# A phase II trial of mTORC1/2 inhibition in STK11 deficient non small cell lung cancer

**DOI:** 10.1038/s41698-025-00838-4

**Published:** 2025-03-11

**Authors:** Gary Middleton, Helen L. Robbins, Peter Fletcher, Joshua Savage, Manita Mehmi, Yvonne Summers, Alastair Greystoke, Nicola Steele, Sanjay Popat, Pooja Jain, James Spicer, Judith Cave, Paul Shaw, David Gilligan, Danielle Power, Dean Fennell, Maya Bajracharya, David J. McBride, Uma Maheswari, Alexander M. Frankell, Charles Swanton, Andrew D. Beggs, Lucinda Billingham

**Affiliations:** 1 University Hospitals Birmingham NHS Foundation Trust, Birmingham, UK; 2 Department of Immunology & Immunotherapy, University of Birmingham, Birmingham, UK; 3 Cancer Research UK Clinical Trials Unit, University of Birmingham, Birmingham, UK; 4 The Christie, Manchester, UK; 5 Newcastle University, Newcastle, UK; 6 Beatson West of Scotland Cancer Centre, Glasgow, UK; 7 The Royal Marsden Hospital, London, UK; 8 St James’s University Hospital, Leeds, UK; 9 King’s College London, Guy’s Hospital, London, UK; 10 Southampton University Hospitals NHS Trust, Southampton, UK; 11 Velindre Cancer Centre, Cardiff, UK; 12 Addenbrooke’s Hospital, Cambridge, UK; 13 Imperial College Healthcare NHS Trust, London, UK; 14 Leicester Royal Infirmary, Leicester, UK; 15 Illumina Cambridge Ltd, Cambridge, UK; 16 Cancer Evolution and Genome Instability Laboratory, The Francis Crick Institute, London, UK; 17 Department of Cancer & Genomic Sciences, University of Birmingham, Birmingham, UK; 18 Present Address: Department of Immunology & Immunotherapy, University of Birmingham, Birmingham, UK

**Keywords:** Non-small-cell lung cancer, Targeted therapies

## Abstract

There are no current stratified medicine options for STK11-deficient NSCLC. STK11 loss mediates mTORC activation, GLUT1 up-regulation and increased glycolysis. This metabolic reprogramming might represent a therapeutic vulnerability targetable with mTORC1/2 inhibition. In arm B2 of the National Lung Matrix Trial 54 patients with NSCLC received vistusertib, of which 49 were STK11-deficient (30 with *KRAS* mutation (B2D), 19 without (B2S)). Objective response (OR) and durable clinical benefit (DCB) rates with 95% credible intervals (CrI) were estimated from posterior probability distributions generated using Bayesian beta-binomial conjugate analysis. In B2D, 2 per-protocol patients obtained OR (estimated true OR rate (95%CrI) 9.8% (2.4–24.3). Estimates of true DCB rate (95%CrI): B2D 24.4% (11.1–42.3), B2S 14.6% (3.6–34.7). Overall, vistusertib cannot be recommended in this context. Longitudinal ctDNA analysis demonstrates enrichment of *SMARCA4* mutations post-treatment. In vitro studies show adaptive resistance to mTORC1/2 inhibition via AKT reactivation. (NCT02664935, ISRCTN38344105, EudraCT 2014-000814-73, 10 June 2015)

## Introduction

Approximately 20–30% of lung adenocarcinomas have mutation or loss of STK11^1–3^, however there are no current targeted therapies for this lung cancer subset. STK11 activates AMPK under conditions of energetic stress, which in turn activates TSC1/2^4^. TSC1/TSC2 represses Rheb, a key activator of mTORC1. AMPK also directly inhibits Raptor (a major mTORC1 component) in an STK11-dependent fashion^5^. Thus, lung adenocarcinomas with loss or inactivation of STK11 have low AMPK activation and high mTOR activity^1^. The resultant increase in mTORC1 signalling drives increased translation of downstream targets: up-regulating genes promoting cell growth and survival, including HIF-1α, due to activation of S6K and inhibition of 4EBP1 through phosphorylation^4,6,7^. HIF-1α drives GLUT1 upregulation, increasing glucose metabolism, and these increases are reversible with the mTORC1 inhibitor rapamycin^7^. As a result, mTOR signalling may represent a targetable therapeutic vulnerability in *STK11*-mutant NSCLC.

Approximately 8% of lung adenocarcinomas have concomitant STK11 loss and KRAS mutation^8,9^, which confers a particularly poor prognosis^10–12^. KRAS and STK11 alterations synergise to drive epigenetic and metabolic re-programming to a greater degree than either mutation alone, a phenomenon reversible by mTORC1/2 inhibition^13,14^. STK11/KRAS co-mutated lung cancer cells demonstrate increased glycolysis, and are more sensitive to glycolysis inhibition or glucose deprivation than either KRAS single mutant or STK11 single mutant cells^15^. A549 (STK11 deficient/KRAS mutant lung adenocarcinoma) cells again demonstrate increased glycolysis and increased HIF1α expression in normoxia, both of which are reduced with either STK11 re-expression or rapamycin treatment^16^. In vivo, STK11 reactivation in an STK11-deficient/*KRAS* mutant lung cancer model markedly reduces tumour burden, abrogates increased glucose avidity that coincides with tumour progression and downregulates glycolysis-related genes and genes relating to mTOR signalling, consistent with its role in inhibiting mTOR in this context^17^. Thus, we hypothesised that cancers harbouring dual *KRAS* mutation/*STK11* inactivation might be particularly vulnerable to targetting mTOR signalling.

Dual mTORC1/2 inhibition may be more effective than mTORC1 inhibition alone. mTORC2 is a central node driving enhanced glycolysis across cancer cell lines, including A549^18^. Rictor knockdown suppresses glycolytic gene expression and glucose uptake mediated by c-Myc in a manner independent of Raptor, HIF1α and Akt. Kockout of Rictor, a key component of the mTORC2 complex in A549 cells reduces glucose uptake to a degree similar to Raptor knockdown^19^. Overall, mTORC2 inhibition appears to be important in effectively switching off aerobic glycolysis. Further, inhibition of mTORC1 alone enhances mTORC2 activation, allowing potential escape through bypass signalling^20^.

MLN0128, clinically developed as sapanasertib, is an ATP-competitive dual mTORC1/2 inhibitor. MLN0128 more potently inhibits pS6K than rapamycin in A549 cells and also inhibits 4EBP1 phosphorylation as well as mTORC2, neither of which are effected by rapamycin^21^. Like phenformin, which increases the AMP/ATP ratio, it is a potent inducer of energetic stress. MLN0128 significantly and durably inhibits HIF1α and GLUT1 in double STK11/KRAS mutant cells in vitro and significantly reduces their glucose consumption and lactate production^21^. Genetically engineered mouse models are ideal systems in which to recapitulate in vivo the complex heterogeneous organ-specific biology of cancer in an immunocompetent setting. The Shackleford group performed in vivo therapeutic studies with MLN0128 using NSCLC models initiated by a conditionally activated K-ras oncogene (LKB1-proficient K_LUC_ mice) and combined with LKB1 deletion in mice also bearing floxed alleles of LKB1 (KL_LUC_ mice), in which lung cancers are induced by intranasal administration of Adeno-Cre^21^. In lung tumour lysates MLN0128 monotherapy causes robust inhibition of HIF1α and GLUT1, translating to a significant and maintained reduction in lung tumour FDG avidity and suppression of tumour growth. FDG avidity and tumour growth suppression with MLN0128 is minimal in LKB1 proficient K_LUC_ tumours. By 8 weeks resistant lung cancer nodules develop which are FDG avid, but these resistant nodules are not adenocarcinoma (the classic histological subtype of human STK11-deficient/KRAS mutant lung cancer) but squamous cell carcinoma. These proof of principle and mechanism studies demonstrate in a relevant lung cancer model a significant susceptibility of KRAS mutant/STK11 deficient lung adenocarcinoma to dual mTORC1/2 inhibition.

Vistusertib (AZD2014), like sapanasertib, is a selective ATP-competitive inhibitor of mTOR kinase^22^. We report the final results of the outcome of patients treated with vistusertib in treatment arm B of the National Lung Matrix Trial (NLMT), a large stratified medicine trial in NSCLC, focusing on the cohort of patients whose NSCLC harbour STK11 loss, with or without concomitant *KRAS* mutations. This is the first study to investigate the impact of mTOR inhibition on targeting STK11-deficient NSCLC. To our knowledge, this is the first prospective precision oncology trial of targeted therapy in this lung cancer sub-type.

## Results

### Clinical trial outcomes

**Fig. 1:**
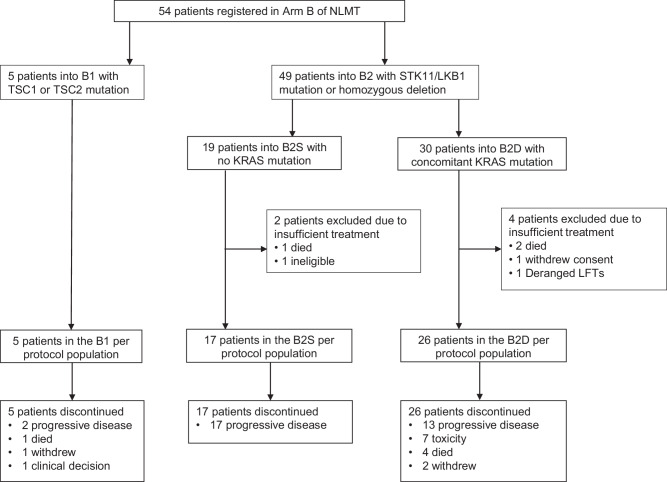
Patient flow diagram for Arm B of the National Lung Matrix Trial.

Between 13 May 2015 and 2 January 2020, 54 patients were registered from 13 UK hospitals to treatment arm B of NLMT, an umbrella phase II platform trial in advanced NSCLC investigating nine experimental targeted drug interventions in 23 different actionable biomarker cohorts (Supplementary Fig. 1). 5 patients had a *TSC1* or *TSC2* mutation and were assigned to cohort B1. 49 patients had an *STK11*/*LKB1* mutation or homozygous deletion and were allocated to cohort B2: 30 had a concomitant *KRAS* mutation and were sub-assigned to cohort B2D; 19 were *KRAS* wild-type (wt) and were sub-assigned to B2S (Fig. 1). All 54 patients were included in the safety analysis of vistusertib. 6 patients were excluded from the *per-protocol* population resulting in 5, 26, and 17 patients included in B1, B2D, and B2S respectively. Recruitment to B2S was closed prematurely because of lack of efficacy, with recruitment to B1 and B2D closed later, again due to lack of efficacy and de-prioritisation of vistusertib from further clinical testing.

In the 54 registered patients, median age was 66 years, 59% were female. 85% of cancers were adenocarcinoma. 67% of patients had stage IV disease. 20% patients were PS0, 70% were PS1. Distribution of baseline characteristics was similar across the 3 cohorts (Supplementary Table 1). Data on previous treatment was available for 52 patients. Patients had received between 1 and 5 lines of previous systemic therapy (median 2). Of these 52 patients, the previous treatment received included platinum-doublet chemotherapy (±immunotherapy/targeted therapies) in 88%, immunotherapy in 25%, targeted therapy in 27%, radiotherapy in 48%, and surgery in 25%.

At the time of data lock (13-October-2023), only 2 patients in the per-protocol (analysis) population were censored for overall survival time, with follow-up times of 322 days and 56 days.

**Fig. 2:**
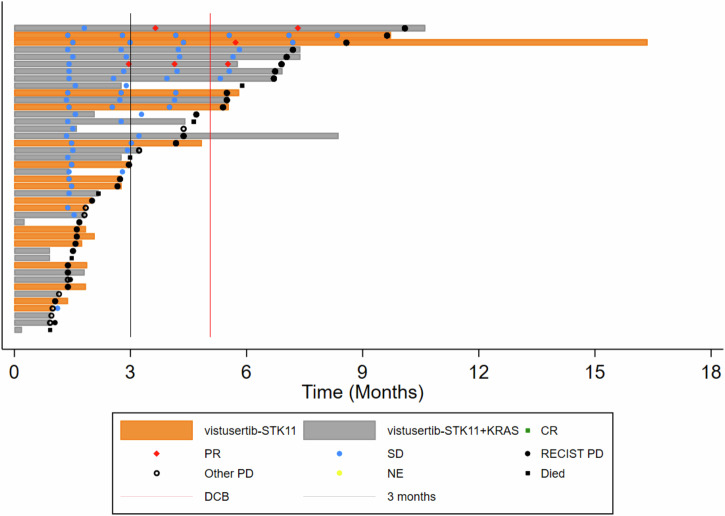
Swimmer plot for participants with STK11+/- KRAS (Cohort B2) as indicated, ordered by progression-free survival time. CT assessments are indicated and coded by Complete or Partial Response, Stable Disease, Progressive Disease or Not Evaluable according to RECIST v1.1. Follow-up time after treatment discontinuation, commencing a post-trial treatment, and death are also indicated. The solid black vertical line indicates 3 months, and the solid red line 22 weeks, being the minimum time that a participant can be counted as reaching the Durable Clinical Benefit threshold.

Across the 48 per-protocol patients, the median number of cycles received was 2 cycles, with patients receiving between 1 and 17 cycles of treatment; 71% of per-protocol patients received between 1 and 4 cycles (including all patients in B1). The most common reason for treatment discontinuation was progressive disease (67%). A swimmer plot for participants with STK11+/- KRAS (Cohort B2) is displayed in Fig. 2. There were 647 AEs (Adverse Events) and 298 ARs (Adverse Reactions) in 53 of the registered patients, but only 10% of these events were reported as Grade 3 or above, with gastrointestinal disorders and fatigue being the most common (Supplementary Table 2), in keeping with previously published AE profiles of vistusertib.

**Fig. 3:**
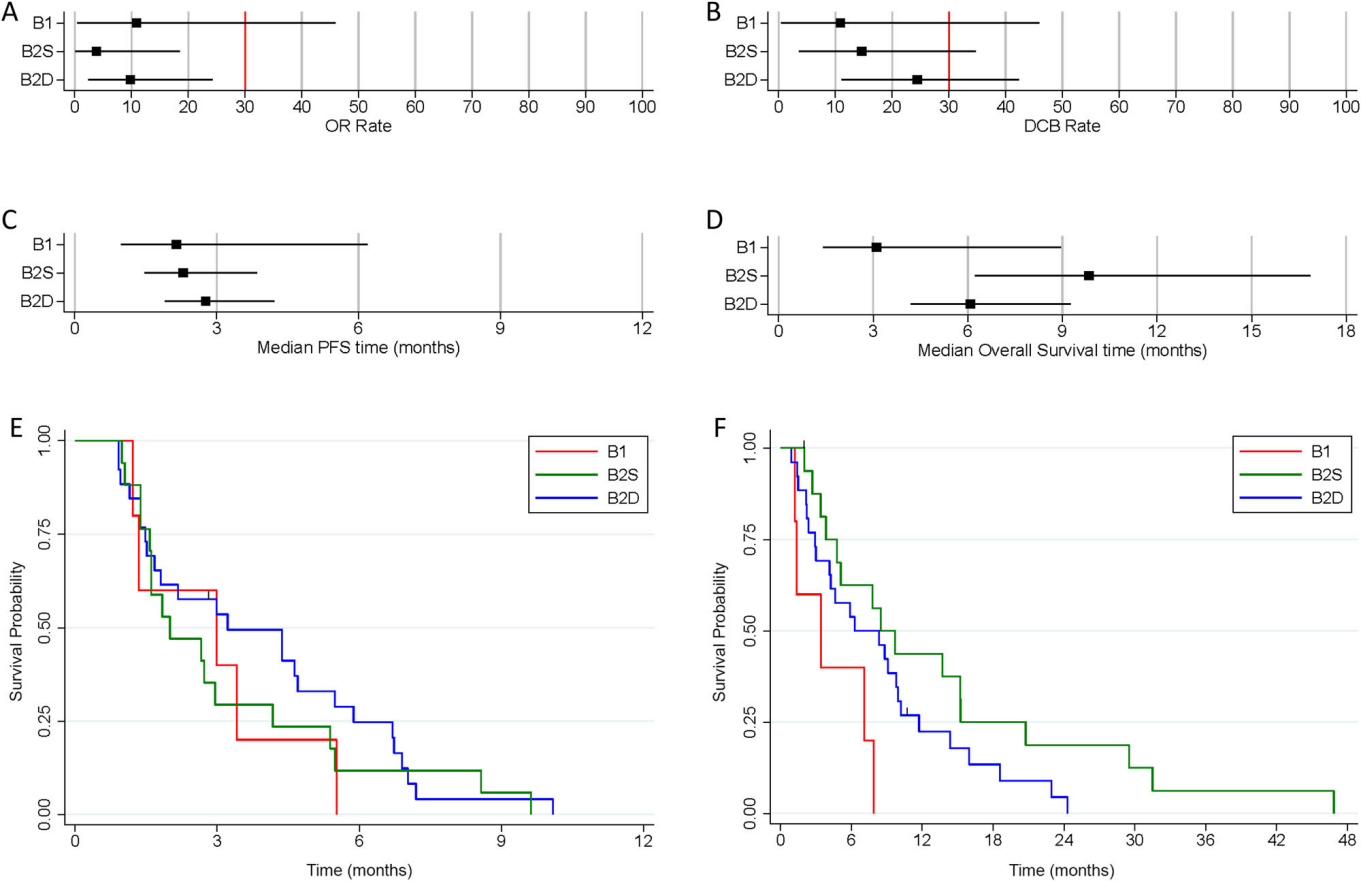
Summarised clinical endpoints (objective response rate, durable clinical benefit rate, progression-free survival, overall survival). Forest plots indicating the median and 95% Credible Interval for (**A**) Objective Response rate, (**B**) Durable Clinical Benefit rate, (**C**) median Progression-free Survival time, and (**D**) median Overall Survival time. The vertical red lines for the co-primary outcomes of OR and DCB indicate the minimum clinically relevant threshold for the true rates that would generate a GO decision. Kaplan-Meier curves for (**E**) Progression-free Survival time, and (**F**) Overall Survival time.

**Fig. 4:**
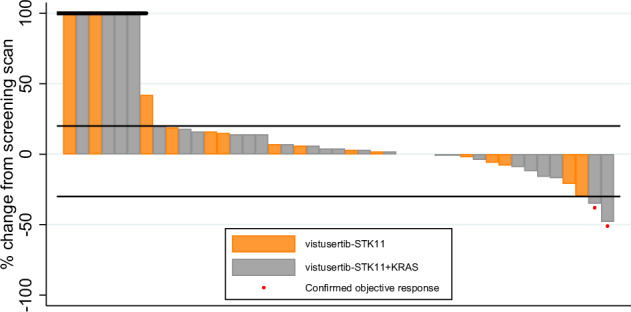
Waterfall plot indicating the Best Percentage Change in sum of target lesion diameters for patients in the B2 cohorts. Per-protocol participants who discontinued without having returned a measurement are included on the left of the plot with a default measurement of +100%.

In terms of co-primary outcome measures, there were only 2 per-protocol patients with OR in treatment arm B, both in cohort B2D (2/26) giving an estimated true OR rate (95%CrI) of 9.8% (2.4–24.3) in that cohort (Figs. 3A and 4; Supplementary Table 3) with a very low probability of a clinically relevant true OR rate (Bayesian posterior probability (PP) < 0.01). Durable clinical benefit (DCB) was defined as progression-free survival at 24 weeks, the time of the fourth on-treatment CT assessment of response. DCB outcomes were somewhat more encouraging with 6/26 in B2D and 2/17 in B2S giving estimates of 24.4% (11.1–42.3) and 14.6% (3.6–34.7), respectively (Figs. 2 and 3B; Supplementary Table 3) but even in B2D the probability of a clinically relevant true DCB rate was still low (PP = 0.26).

In terms of best percentage change in sum of target lesion diameters, the waterfall plot shows no clear segregation between B2D and B2S, with both types of patients distributed across the full range of values (Fig. 4). Median PFS was estimated as less than 3 months for all cohorts (Fig. 3C, E; Supplementary Table 3). Overall survival time was best for B2S with median of 9.8 months (6.2–16.9) with median of 6.1 months (4.2–9.3) for B2D (Fig. 3D, F; Supplementary Table 3).

In the 8 STK11-deficient patients with DCB, the median best percentage change in target lesion sum was −18.5%, ranging from −48% to +3%. In the same cohorts, in the 18 patients with a recorded PFS time of less than 3 months who were evaluable for this outcome, the median percentage change was +10.5%, ranging from −6% to +48% (Fig. 4).

### Longitudinal ctDNA analysis

We analysed longitudinal ctDNA profiles using the Illumina TSO500 platform. Using a conventional *p* < 0.05 for significance, 2 genes were significantly enriched in post-treatment versus baseline samples: top-ranked was SMARCA4 (detectable in 3 patients at baseline (8.3%, 3/36) and detectable in 11 patients post-therapy (40.7%, 11/27, *p* = 0.004), followed by FOXP1 (0 patients at baseline versus 4 patients post-treatment (15%, 4/27), *p* = 0.029). We show that SMARCA4 mutations are positively selected for in STK11 mutant LUAD, unlike FOXP1, and are sub-clonal in 50% of primary samples (Supplementary Fig. 2). All identified SMARCA4 mutations were pathogenic (frameshift, trinucleotide indel, pathogenic splice or CADD > 20^23^, Supplementary Table 4). We wondered whether SMARCA4 deficient clones might be inherently less sensitive to mTOR inhibition due to lower levels of mTOR activation. Supplementary Fig. 3 shows indeed that LUAD harbouring SMARCA4 driver mutations have significantly lower mTOR activation and activation of the mTORC2 substrate AKTSer473.

### In vitro comparison of vistusertib and everolimus in STK11 deficient lung cancer cell lines and evaluation of feedback reactivation of AKT

**Fig. 5:**
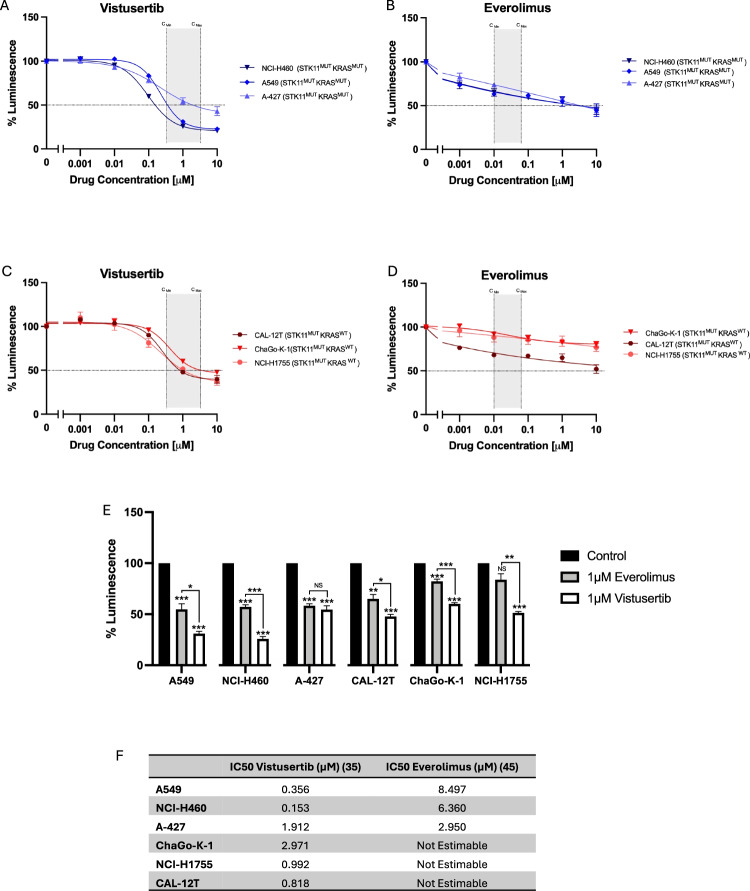
Cell viability of STK11-mutant lung cancer cell lines treated with vistusertib (mTORC1/2 inhibition) or everolimus (mTORC1-only inhibition). **A**–**D** Cell viability of NCI-H460, A-427, NCI-A549 (STK11mut KRASmut) and ChaGoK1, NCI-H1755, CAL-12T (STK11mut KRASwt) after treatment with 0 - 10 µM vistusertib or everolimus for 48 hours, expressed as mean ± SEM from at least 3 independent experiments. Pharmacologically relevant concentrations are indicated in shaded area, calculated from previous publications^63,64^. **E** Relative luminescence for cells treated with 1µM everolimus vs. 1µM vistusertib (data presented from same experiements as described in **A**–**D**). **F** shows calculated IC50 values for everolimus and vistusertib in STK11 mutant NSCLC cell lines.

During the conduct of this study the clinical development of vistusertib was terminated in late 2018 based on inferior outcomes compared with everolimus in randomised trials in both renal and breast cancer^24,25^. In light of this, we compared the sensitivity of STK11-deficient NSCLC cell lines with and without *KRAS* mutations to vistusertib and everolimus treatment in vitro. All 6 cell lines treated with vistusertib show dose-dependent reduction in viability, with IC50 being reached in all lines at pharmacologically relevant doses (Fig. 5). In contrast, IC50 is not reached for any STK11-deficient cells harbouring wild-type *KRAS* when treated with everolimus. Whilst IC50 was reached in STK11-deficient cell lines with concomitant mutant *KRAS*, this is at everolimus concentrations orders of magnitude above pharmacologically achievable concentrations.

**Fig. 6:**
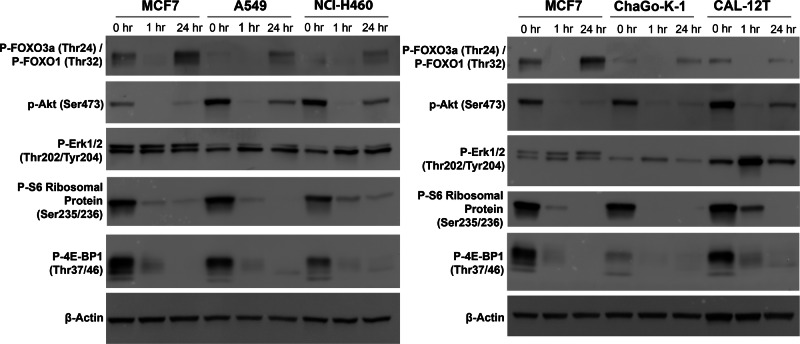
Effect of vistusertib (AZD2014) on cell signalling. MCF7 (control breast cancer cell line), NCI-A549 and NCI-H460 (STK11^MUT^KRAS^MUT^) (left) and MCF7, ChaGo-K-1 and CAL-12T (STK11^MUT^KRAS^WT^) (right) treated with 2 µM vistusertib. Lysates were collected at indicated times. Experiment performed three times on independent biological replicates, representative blots are shown.

An important reason that was postulated for the inferior outcomes compared with everolimus in the renal cancer study was the relief of feedback inhibition of receptor tyrosine kinase signalling through initial AKT inhibition caused by mTORC2 inhibition, leading to subsequent reactivation of AKT and inhibitory downstream phosphorylation of targets including the pro-apoptotic FOXO transcription factors^26,27^. Given STK11 is required for phosphorylation of FOXO by Akt^28^, we had originally hypothesised that this adaptive resistance mechanism may be less relevant in STK11-deficient LUAD. However, in light of the suggestion that inhibition of FOXO might underlie the poor outcomes in renal cancer with vistusertib^27^, we assessed PI3K/AKT pathway signalling at baseline and at 1 and 24 h post-treatment with 2 µM vistusertib (Fig. 6). MCF7 breast cancer cells were used as a positive control, as relief of feedback inhibition by mTORC1/2 is well characterised in this line^26^. As previously reported, we demonstrate clear rebound phosphorylation of FOXO3a/FOXO1 at 24 h after total loss at 1 h in MCF7. In all STK11-deficient cell lines we demonstrate that whilst Akt phosphorylation at Ser473 is reduced at 1 h it becomes re-activated at 24 h, albeit not reaching baseline levels. Baseline FOXO1/3a phosphorylation is low in all STK11-deficient cell lines, with levels further reducing at 1 h, but there is clear rebound phosphorylation at 24 h, to levels exceeding that seen in baseline. All *STK11* mutant cell lines demonstrate increased Erk phosphorylation following treatment with vistusertib, most marked at 1 h. Thus, *STK11* mutant NSCLC cell lines are not exempt from relief of feedback inhibition of receptor tyrosine kinase signalling in response to mTORC1/2 blockade.

## Discussion

Despite strong preclinical rationale supporting the use of mTORC1/2 inhibition in STK11-deficient lung cancer, clinical activity of vistusertib was clearly disappointing, even in cancers with concomitant *KRAS* mutations. Therefore vistusertib cannot be recommended as a potential personalised therapy option in this sub-set of NSCLC. Cell line sensitivity data suggests that alternative clinical testing with everolimus is unlikely to change our conclusion that mTOR inhibition is of limited value in STK11-deficient lung cancer. Indeed, in the phase II trial of everolimus in a pan-cancer cohort of patients with inactivating TSC1/2 or activating mTOR mutations, the response rate and DCB rate was numerically lower than that for cohort B2D in the current report^29^. Sapanisertib (MLN0128) provided strong pre-clinical rationale for trialling mTORC1/2 inhibition in the context of dual STK11 loss and *KRAS* mutation^21^. However, like vistusertib, sapanisertib also failed to improve outcomes over everolimus in both renal and ER+ breast cancer^30,31^. In the former study median OS was 6 months less with sapanisertib compared with everolimus, response rates 0% and 16.7% respectively, and rate of treatment-related AEs leading to discontinuation trebled with sapanisertib^30^. Finally, whilst STK11 appears necessary for competent downstream signalling from AKT to inhibit pro-apoptotic molecules^27^, we show that FOXO3a/FOXO1 still becomes re-phosphorylated at 24 h despite STK11 deficiency. This adaptive resistance may be one reason limiting the impact of mTORC1/2 inhibition in STK11-deficient LUAD, as previously suggested in other contexts^27^. Indeed, activation of AKT was postulated as driving resistance in the squamous cancers that eventually progress in KL_LUC_ mice under dual mTORC1/2 inhibition^21^.

An important initial premise of this study was that the metabolic sequelae of STK11 loss, particularly in combination with *KRAS* mutation, would create a metabolic vulnerability of enhanced glycolysis exploitable using mTORC1/2 inhibition. As described above, data in relevant mouse models of *KRAS* mutant/STK11 deficient adenocarcinoma fully supported this hypothesis^21^. However, subsequent data showed that glycolysis inhibition in the *KRAS* mutation/STK11 deficient genotype constitutes a relatively weak vulnerability compared with inhibition of glycolysis in squamous lung cancers. Whilst A549 cells have a somewhat higher expression of GLUT1 compared to other LUAD lines, levels are much lower than in squamous cell lung cancer (LUSC) lines^32^. A549 *STK11*/*KRAS* co-mutant lung adenocarcinoma cells are more sensitive to GLUT1 knockdown than other LUAD lines, however, they continue to proliferate. In contrast, GLUT1 knockdown markedly suppresses growth of LUSC cells and induces apoptosis. Further, GLUT1 knockdown significantly reduces LUSC growth in vivo but has no effect on A549 xenografts. At first sight, these later studies suggested that the relatively limited effect of vistusertib in STK11 deficient/*KRAS* mutant lung cancer might have been predicted. However, down-regulation of HIF1α and GLUT1 are not the only impacts of mTORC1/2 inhibition: whilst MLN0128 monotherapy has marked effects on tumour growth in the KL_LUC_ model, rapamycin fails to effect tumour growth^21^. A key difference in action of these two drugs is the suppression of inhibitory 4E-BP1 phosphorylation by MLN0128 which is not affected by rapamycin. Whilst the use of a dominant negative 4E-BP1 mutant that binds eIF4E to inhibit cap-dependant translation causes reduction of cell growth in STK11-deficient/*KRAS* mutant cells it has no effect on glycolysis^21^. Indeed, this group had earlier shown that the metabolic stress driven by the glycolysis inhibitor 2DG induces minimal apoptosis in A549 cells unlike the energetic stress caused by phenformin^33^. This suggests that the in vivo efficacy of MLN0128 in the KL_LUC_ models is only partly related to glycolysis inhibition. The inhibition of 4E-BP1 phosphorylation that we also show here using vistusertib in the setting of STK11 deficiency will reduce expression of multiple other key molecules besides HIF-1α/GLUT1 which promote cancer cell proliferation, growth, and survival via inhibition of their cap-dependant translation^34^.

mTOR inhibition as a therapeutic strategy in STK11-deficient LUAD emphasises the role of STK11-dependent AMPK activation. However, AMPK loss does not phenocopy STK11 loss in *KRAS* mutant NSCLC mouse models^35^. Whilst knockout of STK11 greatly accelerates growth as expected, AMPK knockout retards growth. Under glucose depletion, AMPK knockout *KRAS* mutant TP53 conditionally deleted (KP) cells proliferate more slowly than parental AMPK wild-type KP cells. The pro-tumorogenic effects of AMPK appear to be due to the promotion of lysosomal gene expression in response to glucose depletion. Tfe3 is a master transcriptional activator of this programme which under high nutrient conditions is retained in the cytoplasm via phosphorylation enforced by mTOR. Upon glucose deprivation AMPK is activated inhibiting mTOR and de-repressing Tfe3. Finally, the STK11-downstream targets, SIK1 and SIK3, mediate much of the anti-tumour effect of STK11, therefore therapies targeting chronic up-regulation of CREB-dependent transcription which drives IL-6 production could be explored^36^.

*SMARCA4* is the most significantly enriched co-mutation seen at progression. Notably, the rate of detection of STK11 and/or *KRAS* mutations at baseline and progression is very similar, with just one loss and one gain at progression. In *SMARCA4* mutant lung cancers, *STK11* co-mutation is present in 39% and *KRAS* co-mutation in 36% of cases: its presence with either of these 2 mutations confers a significantly worse prognosis, with an additive effect for co-mutation in all 3 genes^37^. We have shown that *SMARCA4* mutation is frequently sub-clonal and it is likely, given that the frequency at progression is 41%, that vistusertib leads to clonal outgrowth of *SMARCA4* mutant sub-clones which thus become detectable on liquid biopsy in much the same way as baseline pre-treatment *EGFR* T790M sub-clones are selected for upon treatment with 1st/2nd generation EGFR TKIs. Our data adds to the existing literature describing both the emergence of SMARCA4 loss at acquired resistance on targeted therapy^38^ but also an important co-mutation at baseline driving primary resistance, including the inhibition of *KRAS* G12C^39,40^ and EGFR mutant lung cancer with Osimertinib^41^.

It is noteworthy that SMARCA4 loss in human lung cancer is associated with enhanced NRF2 target expression; this effect can be recapitulated in vitro where SMARCA4 knockdown inactivates KEAP1, activates NRF2 and enhances haem oxygenase 1 expression^42^. In EGFR mutant cells osimertinb activates ROS which drives mitochondrial fission and apoptosis^43,44^. In response to enhanced ROS the NRF2 pathway is activated with upregulation of haem oxygenase 1 which buffers ROS. Inhibition of this pathway re-establishes high ROS levels leading to enhanced cell death. As mentioned, resistance occurs in the KL_LUC_ model via adeno-squamous transition (AST)^21^. This is not a peculiarity of this GEMM under the evolutionary stress of mTORC1/2 inhibition. Pre-therapy K_c_L models (in which G12C is conditionally mutated and which are LKB1-deficient) uniformly display adenocarcinoma histology: resistance develops upon G12C inhibition again through squamous differentiation and despite maintained repression of RAS signalling^45^. Importantly, a proportion of STK11 deficient human LUAD express squamous cell carcinoma signatures (SCC) and these p63+ lesions express higher levels of the antioxidant transcription factor NRF2 and lower levels of ROS-induced DNA oxidative damage compared with their TTF1+ counterparts^46^. Alleviation of ROS in the KL model significantly reduces AST. Baseline SCC signatures drive clinical resistance to G12C inhibition with adagrasib uniquely in STK11 deficient G12C mutant cases^45^. Thus, it is entirely feasible that the enrichment of SMARCA4 mutation that we here demonstrate on ctDNA analysis represents another means of buffering highly toxic levels of ROS generated by targeted therapy in the context of dual STK11 deficiency and KRAS mutation. Finally we show that SMARCA4 proficient lung adenocarcinoma have higher AKT and mTOR activation. Thus, this may render *SMARCA4* mutant clones inherently less sensitive to inhibition of mTOR than their *SMARCA4* wild type counterparts. *FOXP1* mutation was also significantly enriched at progression. Loss of FOXP1 enhances proliferation and migration of A549 cells^47^ and also impacts mTOR signalling^48,49^.

The NLMT trial leveraged a Bayesian adaptive trial design to screen targeted therapies efficacy. It is acknowledged that patient numbers the arms reported here are relatively low, reflecting arms closing prematurely because of lack of efficacy. It is also important to note that the population was heavily pre-treated and were predominantly White British, potentially limiting generalisability to the global population. Despite these limitations, we are unable to recommend expanding trials of mTORC1/2 inhibition to a larger population of STK11-deficient NSCLC based on the present analysis. Crucially, this work also highlights the value of adaptive and novel trial designs in the era of personalised medicine.

In summary, the modest impact of vistusertib in STK11-deficient NSCLC appears in part due to the selection of *SMARCA4* co-mutated clones on treatment together with adaptive resistance driven by FOXO inactivation. The associated metabolic re-wiring of STK11 deficiency in lung adenocarcinoma appears to represent a limited therapeutic vulnerability. Furthermore, the mechanisms of oncogenicity in STK11-deficient NSCLC appears not to be predominantly through loss of AMPK activation. Given clear evidence that inhibition of glycolysis appears to be a true metabolic and therapeutic vulnerability in LUSC we are shortly initiating recruitment into the KETO-LUNG trial integrating a ketogenic diet alongside chemo-immunotherapy in LUSC.

## Methods

### Trial design and patient selection

Patients were recruited to treatment arm B of NLMT (ClinicalTrials.Gov NCT02664935, ISRCTN38344105, EudraCT 2014-000814-73), with full methodology as previously published^50^, and trial protocol available in supplemental. The trial was submitted and published to ISRCTN on 10th June 2015. Due to a clerical error, this was after the trial opened and the first patient was registered on 13th May 2015. Importantly, at this time this met the Health Research Authority timeframe of a maximum of 6 weeks from opening (https://www.hra.nhs.uk/planning-and-improving-research/research-planning/research-registration-research-project-identifiers/). Only 1 patient of 423 was registered before the trial was on registry, as the 2nd patient was recruited on 16th June 2015. In summary, this is an umbrella phase II platform trial in advanced NSCLC investigating nine experimental targeted drug interventions in 23 different actionable biomarker cohorts (Supplementary Fig. 1).

Patients were screened through the Stratified Medicine Programme 2 as previously reported, an observational pre-screening study for advanced lung cancer^49,50^. All patients gave signed informed consent for the trial and for genomic analysis. Profiling used the custom SMP2v.01 or SMPV2v.02 panel of 28 genes, with genetic variants identified through a custom Nextera rapid Capture sequencing assay (Illumina), with libraries generated from 50 ng of DNA (FFPE for tumour, blood for germline testing) and sequencing on the Illumina MiSeq system. Sequencing reads were aligned to hg19 using isSAAC (Illumina), with variant calling performed using Strelka for single-nucleotide variants^51^, CRAFT (Illumina) for SCNAs, and Mantra for structural variants^52^. Aberrations were classified as tier 1, tier 2 or tier 3, with tier 1 and 2 denoting aberrating conferring eligibility for cohorts within the NLMT.

Treatment arm B of the NLMT investigated treatment with vistusertib in 3 separate cohorts; eligible patients with TSC1 or TSC2 mutation were assigned to cohort B1, and those with STK11/LKB1 mutation or homozygous deletion to cohort B2 with sub-assignment to either cohort B2D (double) if a concomitant KRAS mutation was present or cohort B2S (single) if not; target recruitment for each cohort was 30 patients. At the time of this publication^50^ which was an overview of the entire trial the largest cohort reported here, B2D and the cohort with the most compelling rationale for dual mTORC1/2 inhibition, was still open to recruitment and ultimately reached target recruitment. The earlier overview paper also included no translational data as presented here.

Eligible patients had histologically or cytologically confirmed NSCLC stage III (not suitable for radical radiotherapy or surgery) or stage IV with measurable disease according to response evaluation criteria in solid tumours (RECIST v.1.1) and needed to have received previous anticancer treatment or refused standard-of-care first-line therapy; full eligibility criteria can be found in the trial protocol in supplemental materials of Middleton et al.^50^. All patients gave written informed consent in accordance with Good Clinical Practice and the Declaration of Helsinki’s ethical principles for medical research. The NLMT complied with all regulatory requirements; ethical approval was obtained from South Central—Oxford C Research Ethics Committee; clinical trial authorisation was granted by UK Medicines and Healthcare products Regulatory Agency. The trial was sponsored by the University of Birmingham and run by the Cancer Research UK Clinical Trials Unit.

Vistusertib (AZD2014) was administered twice daily orally on an intermittent schedule at a dose of 125 mg bd 2 days on 5 days off, for 28-day cycles until disease progression; patients could continue on trial treatment after progression if tolerated and there was clinical benefit. Blood for ctDNA was collected pre-dose prior to the first cycle of treatment, and then every 8 weeks (every other cycle).

### Statistical design and analysis

A Bayesian adaptive trial design was used to screen each experimental targeted drug intervention in NLMT for signals of efficacy in each selected molecularly defined cohort. For treatment arm B, the co-primary outcome measures for all 3 cohorts were objective response (OR) defined as the incidence of confirmed complete or partial response according to RECIST v.1.1^53^, and DCB defined as the incidence of remaining free of disease progression at the fourth protocol-defined CT assessment at ~24 weeks from commencing treatment. Secondary outcomes included progression-free survival time (PFS), time to progression (TTP), best percentage change in sum of target lesion diameters, overall survival time (OS) and adverse events (AE).

The OR and DCB rates were each estimated from a posterior probability distribution generated using a Bayesian beta-binomial conjugate analysis with a minimally informative Beta(1,1) prior and reported with 95% credible intervals^54^. Median times for PFS, TTP and OS were estimated from a posterior probability distribution generated using a Bayesian exponential-inverse-gamma (IG) conjugate analysis with a minimally informative IG(0.001, 0.001) prior and reported with 95% credible intervals^55^. Best percentage change in sum of target lesion diameters is reported visually using waterfall plots. Decision guidelines, sample size and operating characteristics are described in Middleton et al.^50^.

AEs were defined as any untoward medical occurrence, not necessarily having a causal relationship with the treatment. Adverse reaction (ARs) were defined as untoward and unintended responses related to any dose of the investigational medicinal product administered (judged as having a causal relationship by the reporting investigator).

### Circulating tumour DNA extraction and library preparation

For the purposes of the present analysis, where available, DNA was extracted at baseline and at progression or treatment discontinuation (Supplementary Fig. 4). Where no discontinuation sample was available the last available sample was extracted. cfDNA was extracted from up to 5 ml of plasma using the QIAamp Circulating Nucleic Acid Kit (QIAGEN, 55114). The 90–270 bp fraction was quantified using the Agilent 22000 TapeStation, using Agilent High Sensitivity D5000 ScreenTape and Reagents (Agilent technologies, 5067-5592 and 5067-5593). Library preparation was completed using TruSight Oncology 500 ctDNA Kit (Illumina, 20039252), with cfDNA inputs of 9–75 ng DNA. Where quantified DNA was <9 ng, input was judged to be insufficient. Samples were pooled 24-plex onto an S4 flowcell and sequenced on a NovaSeq 6000 instrument to a median of 35000× normalised read depth coverage Generated FASTQ files were processed using the appropriate DRAGEN pipeline for Trusight Oncology 500 ctDNA, generating a final set of BAM, variant VCF, TMB, MSI and copy number variants aligned to the hg38 genome. VCF files were converted to MAF file format using vcf2maf^56^, merged and then imported into *maftools v2.16*^57^ via *R/Bioconductor v.4.20/3.17*^58^ for analysis. All processing was carried out on the University of Birmingham BEAR HPC service (http://www.birmingham.ac.uk/bear). Raw data for this cohort is available on the NIH SRA (accession number: SUB13654759).

### Cell culture

NCI-A549 (86012804, RRID:CVCL_0023) and ChaGo-K-1 (6020948, RRID:CVCL_1121) were obtained from ECACC. CAL12T (ACC 443, RRID:CVCL_1105) was obtained from DSMZ; NCI-H460 (HTB-177, RRID:CVCL_0459) and NCI-H1755 (CRL-5982, RRID:CVCL_1492), were obtained from ATCC. MCF7 (RRID: CVCL_0031) and A-427 (RRID: CVCL_1055) were a gift from Professor Jo Morris. All cell lines were authenticated by ATCC using STR typing after experiment completion. Cells were grown in RPMI-1640 with L-Glutamine (Sigma-Aldrich, Missouri, R8758) with 10% Foetal Bovine Serum (Sigma Aldrich, F7524) and 1% Penicillin-Streptomycin (Invitrogen, 15140122). All cell lines were routinely tested for mycoplasma using an EZ-PCR Mycoplasma Test kit (Biological Industries, 20-700-20). At a minimum, all cell lines were confirmed mycoplasma negative before experiments were started and after experiments were completed. All experiments were conducted within 20 passages of cell line receipt. All experiments were performed in triplicate, each repeat performed on independent passages.

### Cell viability

Cells were seeded at 1000–1500 cells per well in opaque 96 well plates (Corning, 3610). After 12 h, cells were treated with vistusertib (AZD2014) (SelleckChem, S2783) or Everolimus (SelleckChem, S1120). DMSO concentration was normalised across all conditions, with a maximum DMSO concentration of 0.1%. After 48 h of treatment, cell viability was measured using CellTiter-Glo 2.0 (Promega, G9241). Results are presented as relative luminescence, with background luminescence subtracted (media with no cells), relative to vehicle control. Best fit curves were plotted with non-linear regression using GraphPad Prism 9 (RRID:SCR_002798). Absolute IC50 was defined as the drug concentration resulting in a 50% reduction in cell viability, with values calculated by Prism.

### Antibodies

All primary antibodies were purchased from cell-signalling: P-FOXO1 (Thr24)/FOXO3a(Thr32) 1:1000 (#9464, RRID: AB_329842); pAkt(Ser473) 1:1000 (#4060, RRID: AB_2315049), pErk1/2 (Thr202/Tyr204) 1:1000 (#9101, RRID: AB_331646), Phospho-S6 Ribosomal Protein 1:1000 (#2211, RRID: AB_331679), p-4EBP1(Thr37/46) 1:1000 #9459, RRID: AB_330985), B-actin 1:1000 (4970, RRID: AB_2223172).

### Western blotting

Cells were seeded in 6 well plates, vistusertib was added the next day and lysates collected at indicated times. Lysates were prepared using RIPA buffer (Thermofisher Scientific, 89901) with Protease/Phosphatase Inhibitor Cocktail (Cell Signalling Technology, 5872) according to standard techniques. Protein was quantified using Pierce Bovine Serum Albumin (BSA) Protein Assay Kit (Thermofisher, 23227). Samples were prepared under reducing conditions using Pierce Lane Marker Reducing Sample Buffer (Thermofisher, 39000) and run on 12% Mini-Protean TGX precast gels (Bio-rad, 4561043). Precision Plus WesternC Standard confirmed molecular weight (Bio-rad, 1610376). Wet transfer was performed at 90 V for 90 min onto PVDF membranes (GE Healthcare, Germany). Membranes were blocked (1 h at RT, 5% dried skimmed milk powder (Marvel) in 1 × Tris-buffered saline, 0.1% Tween 20 (TBST)), and incubated with primary antibodies (diluted in 5% BSA/TBST) overnight at 4 C. Membranes were washed three times in TBST, incubated with secondary antibody for 1 h at RT, and washed a further three times. Chemiluminescence was performed using SuperSignal West Femto Maximum Sensitivity Substrate (Thermofisher, 34095) for pFOXO1 and pAkt, and SuperSignal West Pico Plus (Thermofisher, 34580) for all other targets. All blots were repeated three times using independently collected and drugged samples. Blots were imaged using a Fusion FX6XT Digital Imaging System (Vilber) (raw images of all blots available in Supplementary Figs. 5 and 6).

### TCGA analysis

C-Bioportal (RRID:SCR_014555)^59,60^ was used to explore associations between SMARCA4 alteration status and MTOR and Akt phosphorylation in the TCGA PanCancer Atlas Lung Adenocarcinoma cohort^61^, using RPPA data for Akt Ser473 phosphorylation Analysis was restricted to samples with data for both profiles.

Multiregional whole exome sequencing from lung adenocarcinomas in the TRACERx study was used to determine the rates of clonal and subclonal non-synonymous mutations in SMARCA4, FOXP1 and TRAF2 in either a STK11 mutant or STK11 wildtype context as described in Frankell et al.^62^.

## Supplementary information

Supplementary Material

## Data Availability

For NLMT data, scientifically sound proposals from appropriately qualified Research Groups will be considered for data sharing. Requests should be made by returning a completed Data Sharing Request Form and curriculum vitae of the lead applicant and statistician to newbusiness@trials.bham.ac.uk. The Data Sharing Request Form captures information on the specific requirements of the research, the statistical analysis plan, and the intended publication schedule. The request will be reviewed independently by the Cancer Research UK Clinical Trials Unit (CRCTU) Directors at University of Birmingham in discussion with the Chief Investigator and relevant Trial Management Group and independent Trial Steering Committee. In making their decision the Director’s Committee will consider the scientific validity of the request, the qualifications of the Research Group, the views of the Chief Investigator, Trial Management Group and Trial Steering Committee, consent arrangements, the practicality of anonymizing the requested data and contractual obligations. Where the CRCTU Directors and appropriate Trial Committees are supportive of the request, and where not already obtained, consent for data transfer will be sought from the Sponsor of the trial before notifying the applicant of the outcome of their request. It is anticipated that applicants will be notified of a decision within 3 months of receipt of the original request. The results published here are based in part on data generated by TCGA pilot project established by the NCI and the National Human Genome Research Institute. The data were retrieved through database of Genotypes and Phenotypes (dbGaP) authorisation (accession number: phs000178.v10.p8). TRACERx sequencing datasets used in this study are described in Frankell et al.^62^.
