# Supplementary material for: A phase II trial of mTORC1/2 inhibition in STK11 deficient non small cell lung cancer
Source: NPJ Precis Oncol. 2025 Mar 11;9:67. doi: 10.1038/s41698-025-00838-4 (PMC11897347; doi:10.1038/s41698-025-00838-4)
Supplement: Supplementary file 1 — Supplementary Material [file 41698_2025_838_MOESM1_ESM.pdf]

Supplemental Figures

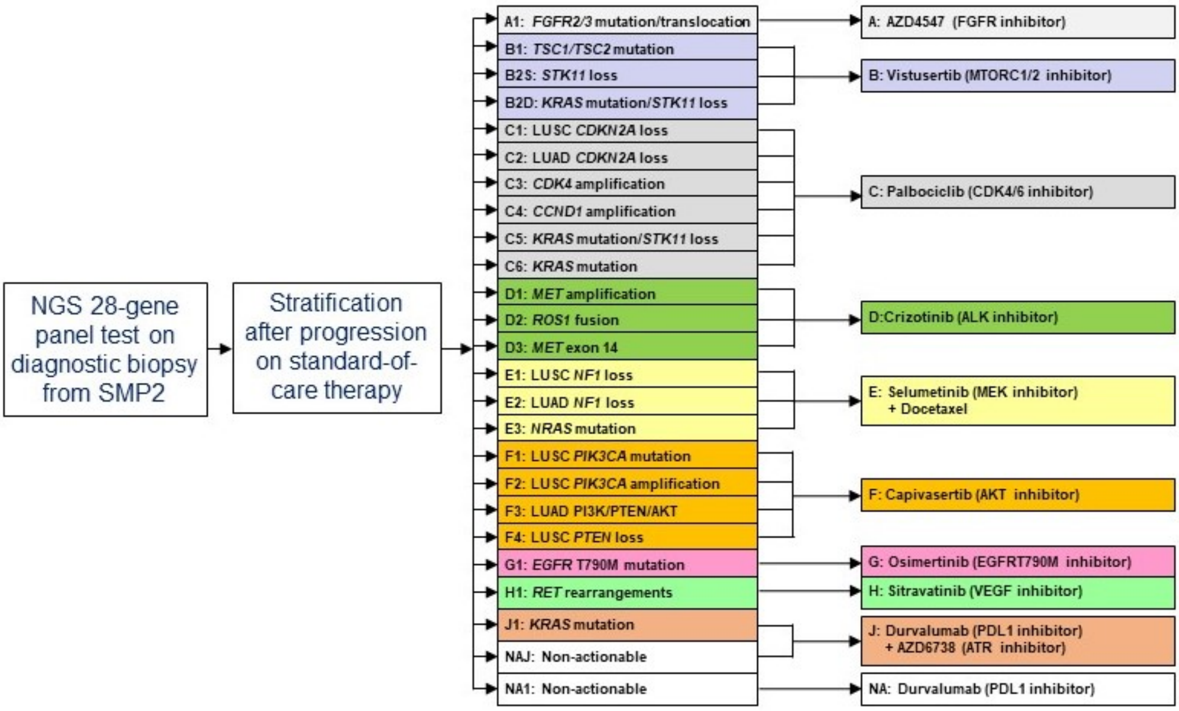

Supplemental Figure 1: Scheme of the National Lung Matrix Trial, showing Arm B with cohorts B1, B2D and B2S.

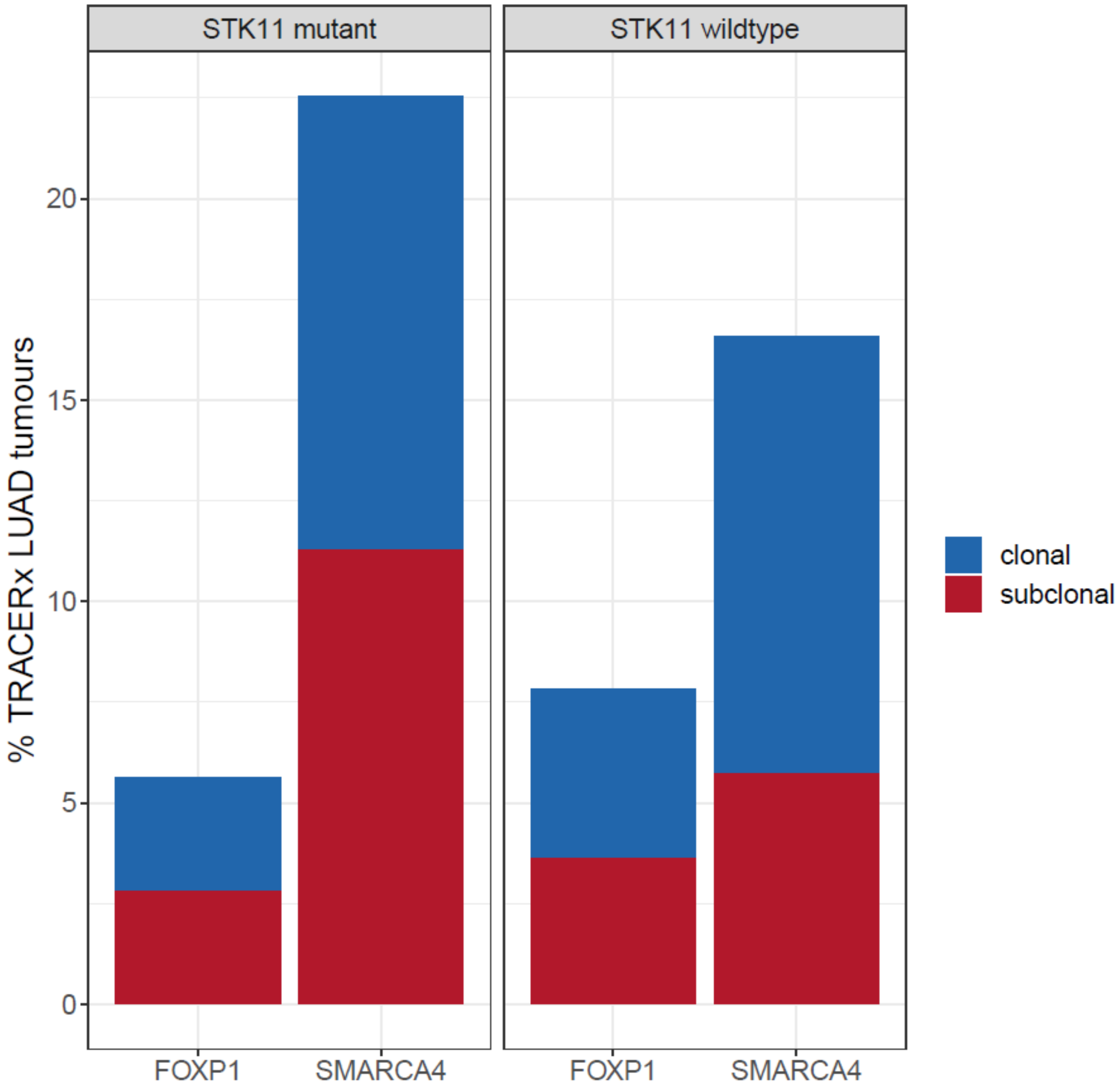

Supplemental Figure 2: Rates of clonal and subclonal non-synonymous mutations in SMARCA4, and FOXP1 from lung adenocarcinomas in the TRACERx study (28) in either an STK11 mutant or STK11 wildtype context.

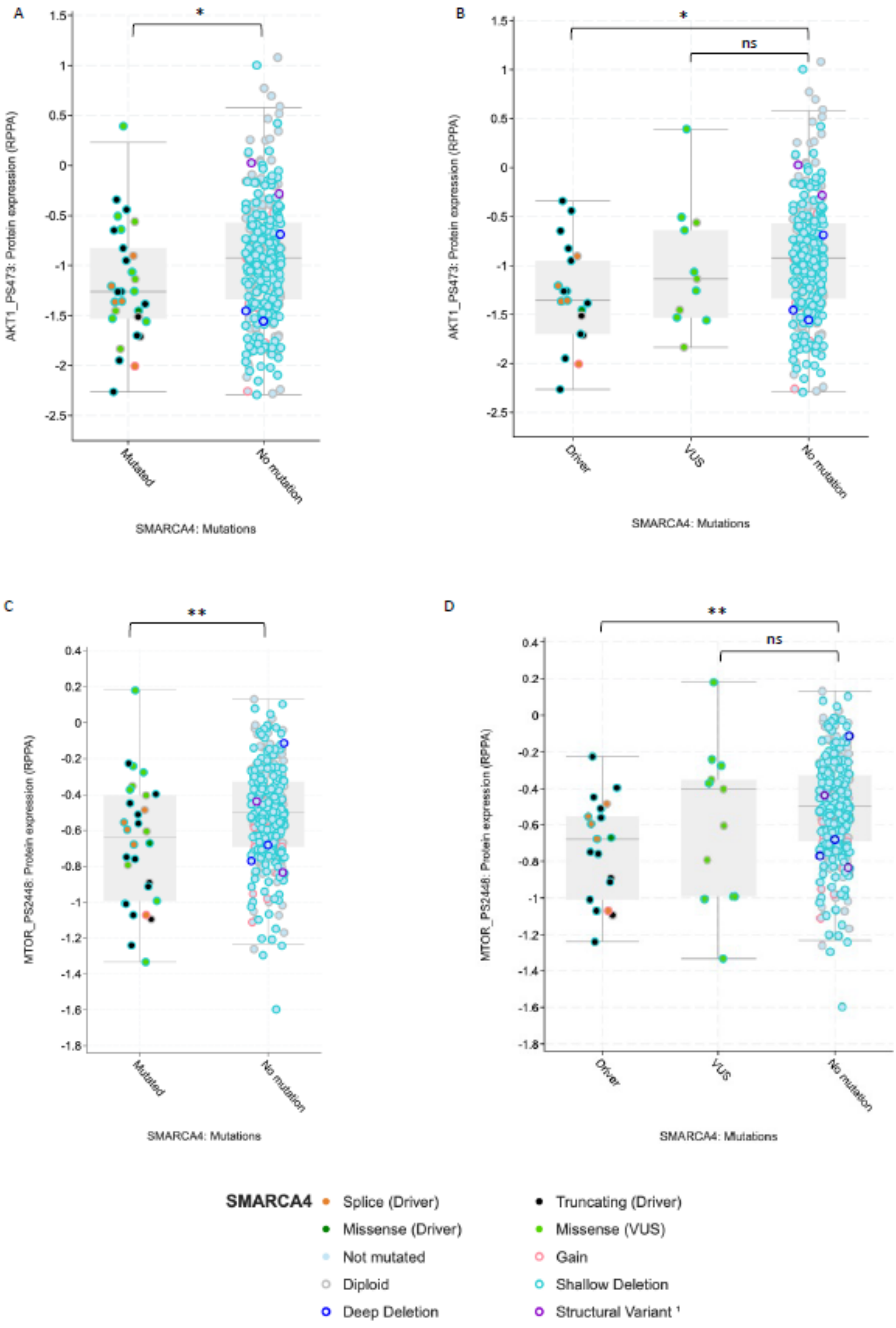

Supplemental Figure 3: Relationship between SMARCA4 status and MTOR phosphorylation at Ser2448 (A-B) and AKT phosphorylation at Ser473 (C-D) in TCGA Lung Adenocarcinoma Cohort, Pan Cancer Atlas (n=356) (28). TCGA lung adenocarcinoma PanCancer Atlas data was accessed via Cbioportal (26,27). Akt pS473 (A-B) and MTOR pS2448 (C-D) levels were assessed according to SMARCA4 mutation status (n=356 for all analyses). Statistical analysis across two groups using unpaired t-test. Statistical analysis across three groups using ordinary one-way ANOVA with multiple comparison testing.

P-Akt: SMARCA4 mutant vs wild-type p = 0.0207; SMARCA4 VUS vs wild-type = 0.8624; SMARCA4 driver mutant vs wild-type = 0.0190

P-MTOR: SMARCA4 mutant vs wild-type p = 0.0035; SMARCA4 VUS vs wild-type p = 0.8009; SMARCA4 driver mutant vs wild-type p = 0.0020.

Supplemental Tables

	B1 (n=5)	B2S (n=19)	B2D (n=30)	All (n=54)
**Age (years)**				
Median	66	67	65	66
Inter-quartile range	61-67	57-71	57-70	57 – 70
Range	59-69	46-86	26-77	26 – 86
**Sex**				
Female	2 (40%)	11 (58%)	19 (63%)	32 (59%)
Male	3 (60%)	8 (42%)	11 (37%)	22 (41%)
**Race**				
White British	5 (100%)	16 (84%)	25 (83%)	46 (85%)
Other White	0 (0%)	3 (16%)	1 (3%)	4 (7%)
White and Black Caribbean	0 (0%)	0 (0%)	1 (3%)	1 (2%)
White and Asian	0 (0%)	0 (0%)	1 (3%)	1 (2%)
African	0 (0%)	0 (0%)	1 (3%)	1 (2%)
Other	0 (0%)	0 (0%)	1 (3%)	1 (2%)
**Performance Status**				
0	0 (0%)	5 (26%)	6 (20%)	11 (20%)
1	5 (100%)	13 (68%)	20 (67%)	38 (70%)
2	0 (0%)	1 (5%)	4 (13%)	5 (9%)
**Histology**				
Adenocarcinoma	1 (20%)	16 (84%)	29 (97%)	46 (85%)
Squamous cell carcinoma	4 (80%)	3 (16 %)	0 (0%)	7 (13%)
Carcinoma NOS	0 (0%)	0 (0%)	1 (3%)	1 (2%)
**Smoking History in pack years (n=46)**				
Never-smoker	0 (0%)	2 (11%)	1 (4%)	3 (7%)
<10	1 (20%)	2 (11%)	4 (16%)	7 (15%)
10-30	0 (0%)	5 (28%)	11 (44%)	16 (35%)
>30	2 (67%)	9 (50%)	9 (36%)	20 (43%)
**Metastases**				
No	1 (20%)	4 (21%)	13 (43%)	18 (33%)
Yes	4 (80%)	15 (79%)	17 (57%)	36 (67%)

Supplemental Table 1: Baseline characteristics for all registered participants in treatment Arm B. Complete smoking history data were available for 46 out of the 54 registered participants.

	AE Grade 3+	AR Grade 3+
**Fatigue**	12 (22%)	7 (13%)
**Vomiting**	4 (7%)	4 (7%)
**Diarrhea**	3 (6%)	3 (6%)
**Lung infection**	3 (6%)	0
**Pulmonary embolism**	2 (4%)	0
**Pneumonia**	2 (4%)	0
**Dyspnea**	2 (4%)	1 (2%)
**Nausea**	2 (4%)	2 (4%)
**Anorexia**	2 (4%)	2 (4%)
**Back pain**	2 (4%)	0
**Mucositis oral**	1 (2%)	1 (2%)
**Non-cardiac chest pain**	1 (2%)	0
**Cough**	1 (2%)	0
**Pneumothorax**	1 (2%)	1 (2%)
**Haemoptysis**	1 (2%)	0
**Chest infection**	1 (2%)	0
**Bronchial infection**	1 (2%)	0
**Cellulitis (left arm)**	1 (2%)	1 (2%)
**Rash maculo-papular**	1 (2%)	1 (2%)
**Abdominal pain**	1 (2%)	1 (2%)
**Small intestinal perforation**	1 (2%)	1 (2%)
**Peritonitis**	1 (2%)	1 (2%)
**Dehydration**	1 (2%)	1 (2%)
**Hyponatremia**	1 (2%)	0
**Hypophosphatemia**	1 (2%)	0
**Aspartate aminotransferase increased**	1 (2%)	0
**Elevated ALT, AST, GGT, Alk Phos**	1 (2%)	0
**Elevated liver function tests**	1 (2%)	0
**ECG changes**	1 (2%)	1 (2%)
**Spinal cord compression**	1 (2%)	0
**Weak legs**	1 (2%)	0
**Shingles**	1 (2%)	0
**Confusion**	1 (2%)	0
**Insomnia**	1 (2%)	0

Supplemental Table 2: Number (%) of patients registered to treatment arm B of NLMT with a least one adverse event (AE) or adverse reactions (AR) reported as Grade 3 or above.

	B1 (n=5)	B2S (n=17)	B2D (n=26)
**Confirmed Objective Response (OR)**			
**Number**	0	0	2
**OR rate (95% CrI)**	10.9% (0.4 - 45.9)	3.8% (0.1 - 18.5)	9.8% (2.4 - 24.3)
**PP(>30%)**	0.12	<0.01	<0.01
**PPoS**	0.10	<0.01	0
**Durable Clinical Benefit (DCB)**			
**Number**	0	2	6
**DCB rate (95% CrI)**	10.9% (0.4 - 45.9)	14.6% (3.6 - 34.7)	24.4% (11.1 - 42.3)
**PP(>30%)**	0.12	0.06	0.26
**PPoS**	0.10	0.01	0.06
**Progression-free Survival time (PFS)**			
**Number of events**	5	17	25
**Median (95% CrI) months**	2.2 (1.0 - 6.2)	2.3 (1.5 - 3.9)	2.8 (1.9 - 4.2)
**Time to Progression (TTP)**			
**Number of events**	3	17	19
**Median (95% CrI) months**	2.9 (1.1-12.3)	2.3 (1.5 – 3.9)	3.3 (2.2 – 5.4)
**Overall Survival time (OS)**			
**Number of events**	5	16	25
**Median (95% CrI) months**	3.1 (1.4 – 9.0)	9.8 (6.2 - 16.9)	6.1 (4.2 - 9.3)

Supplemental Table 3: Estimates of summary measures for primary and secondary outcomes Note: estimates for OR rate and DCB rate are non-zero even when no events observed because of the influence of the prior distribution.

Gene	Chr	Start	End	ProtID	Change	CADD
SMARCA4	chr19	11021767	11021769	P51532	p.K887Y	Trinucleotide Indel so pathogenic by default
SMARCA4	chr19	11035040	11035042	P51532	p.E1364del	Frameshift so pathogenic by default
SMARCA4	chr19	11019680	11019682	P51532	p.K865_D866delinsNG	Frameshift so pathogenic by default
SMARCA4	chr19	11035040	11035042	P51532	p.E1364del	Frameshift so pathogenic by default
SMARCA4	chr19	11007900	11007902	P51532	p.X670_splice	Pathogenic splice as via SpliceVar
SMARCA4	chr19	10986411	10986411	P51532	p.G193V	26.8
SMARCA4	chr19	11035040	11035042	P51532	p.E1364del	Frameshift so pathogenic by default
SMARCA4	chr19	11059837	11059837	P51532	p.E1574*	52
SMARCA4	chr19	11033309	11033309	P51532	p.R1189L	32
SMARCA4	chr19	11007966	11007966	P51532	p.K689M	24.3
SMARCA4	chr19	11033470	11033470	P51532	p.R1243W	24.1
SMARCA4	chr19	11033377	11033377	P51532	p.Glu1212Ter	44

Supplemental Table 4: Summary of SMARCA4 mutations detected on ctDNA from different B2 patients.

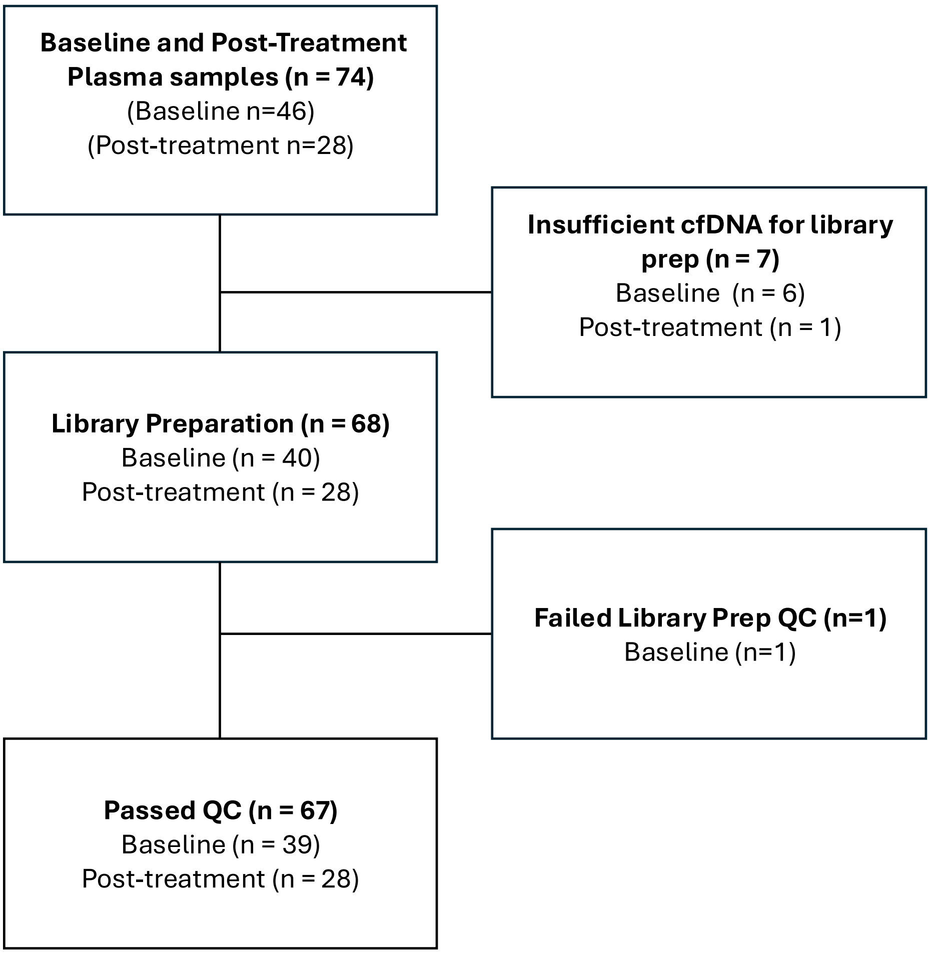

Supplemental Figure 4. Summary of ctDNA processing. cfDNA was extracted using the QIAamp circulating nucleic acid kit, quantified using the Agilent Tapesation, and library prep was completed using the TruSight Oncology 500 ctDNA kit (Illumina). *Note: three baseline samples and one post-treatment sample were excluded from baseline vs post-treatment ctDNA analysis, as patients were not treated with vistusertib, leaving 36 baseline and 27 post-treatment samples for analysis*

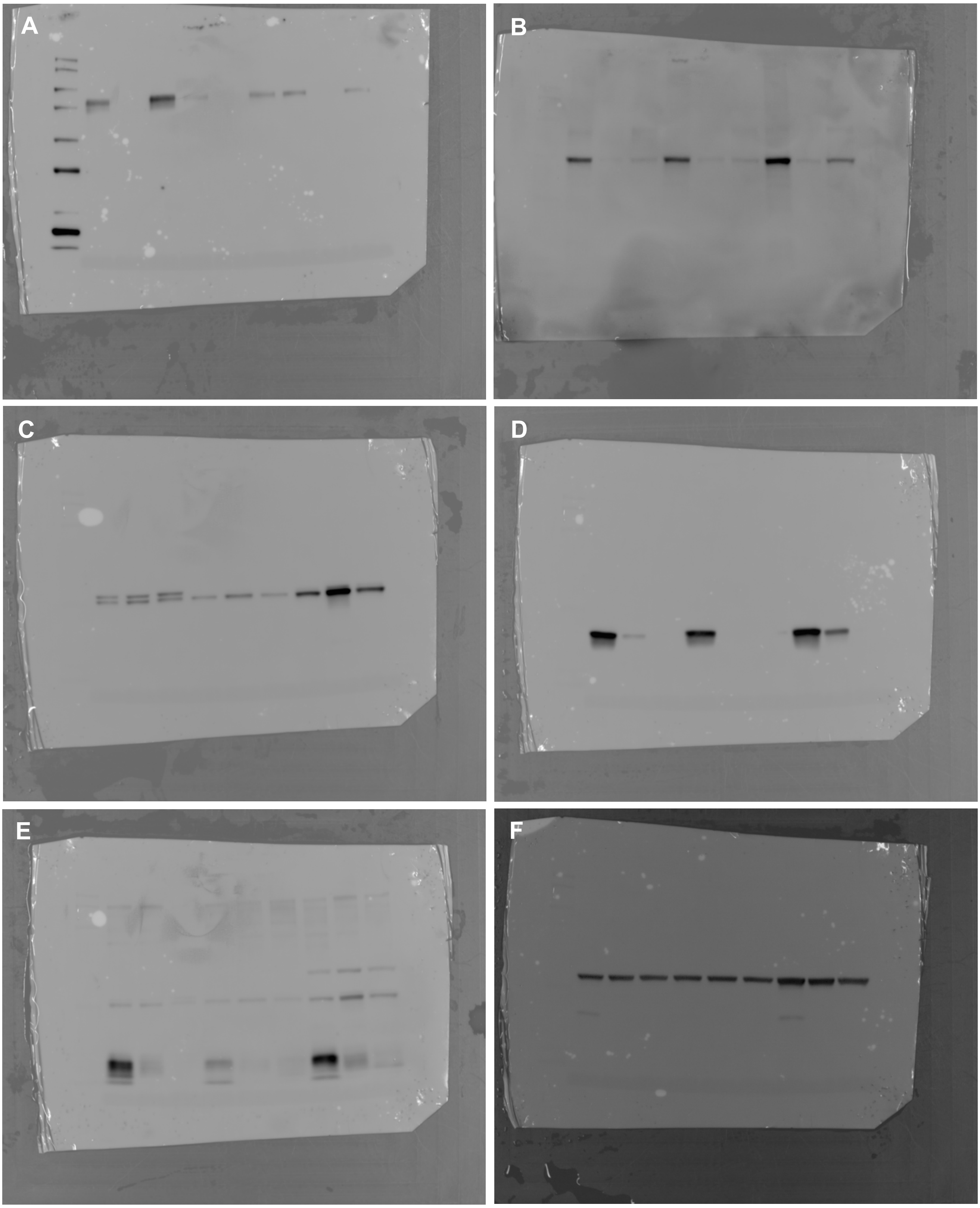

Supplemental Figure 5. Original images of blots examining the effect of vistusertib (AZD2014) on MCF7 (control breast cancer cell line) [Figure 6, right hand blot]. ChaGoK-1 ,CAL-12T. P-FOXO3a/P-FOXO1 (A), p-Akt (B), pERk (C), p-S6 Ribosomal Protein (D), P-4E-BP1 (E), B-Actin (F). MCF7 included as positive and negative internal control. Protein size confirmed using Precision Plus Protein™ WesternC™ Standards (Bio-rad) (developed in A)

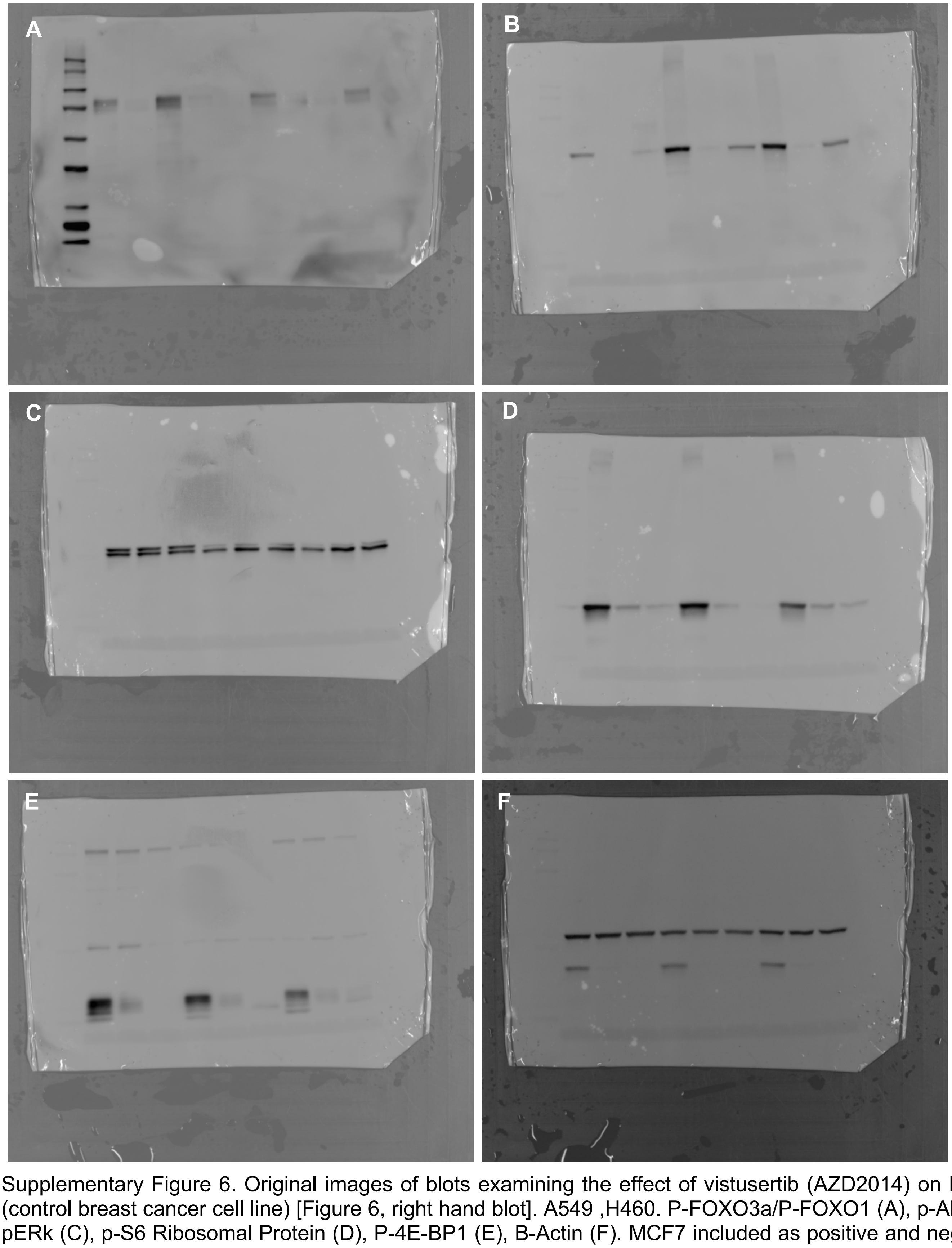

Supplementary Figure 6. Original images of blots examining the effect of vistusertib (AZD2014) on MCF7 (control breast cancer cell line) [Figure 6, right hand blot]. A549 ,H460. P-FOXO3a/P-FOXO1 (A), p-Akt (B), pERk (C), p-S6 Ribosomal Protein (D), P-4E-BP1 (E), B-Actin (F). MCF7 included as positive and negative internal control. Protein size confirmed using Precision Plus Protein™ WesternC™ Standards (Bio-rad) (developed in A)

National Lung Matrix Trial

Multi-drug, genetic marker-directed, non-comparative, multi-centre, multi-arm phase II trial in non-small cell lung cancer

Version 11.0, 08-September-2021

CANCER RESEARCH UK | BIRMINGHAM CANCER RESEARCH UK CLINICAL TRIALS UNIT | UNIVERSITY OF BIRMINGHAM

British Thoracic Oncology Group

EudraCT Number:	2014-000814-73
Sponsor Number:	RG_14-072
CRCTU Number:	LU2007
ISRCTN:	38344105
ClinicalTrials.gov	NCT02664935

TRIAL CONTACTS

Sponsor: University of Birmingham

Chief Investigator: Professor Gary Middleton, Professor of Medical Oncology

Chief Biostatistician: Professor Lucinda Billingham, Professor of Biostatistics

Senior Manager: Dr Joshua Savage, Early Drug Development Team Leader

Senior Trial Coordinator: Dr Manita Mehmi, Early Drug Development Team

Trial Coordinator: Jessica Crook & Sarah Johnson, Early Drug Development Team

Trial Statistician: Peter Fletcher, Biostatistics Team

Lead Investigators:

Arm A – AZD4547<br>*Closed to recruitment*	Professor Gary Middleton	Professor of Medical Oncology, University of Birmingham.
Arm B – Vistusertib (formerly called AZD2014)<br>*Closed to recruitment*	Professor Gary Middleton	Professor of Medical Oncology, University of Birmingham.
Arm C – Palbociclib	Professor Gary Middleton	Professor of Medical Oncology, University of Birmingham.
Arm D – Crizotinib	Professor Sanjay Popat	Consultant Medical Oncologist, Royal Marsden Hospital, London and Honorary Senior Lecturer, Imperial College London.
Arm E – Selumetinib (formerly called AZD6244) & Docetaxel	Professor Gary Middleton	Professor of Medical Oncology, University of Birmingham.
Arm F – AZD5363<br>*Closed to recruitment*	Dr Timothy Yap	Medical Director, Institute for Applied Cancer Science; Associate Professor of Medicine, The University of Texas MD Anderson Cancer Centre, Houston, TX.
Arm G – Osimertinib (formerly called AZD9291)<br>*Closed to recruitment*	Dr Yvonne Summers	Consultant Medical Oncologist, The Christie Hospital and University South Hospital, Manchester.
Arm H – Sitravatinib (formerly called MGCD516)<br>*Closed to recruitment*	Professor Gary Middleton	Professor of Medical Oncology, University of Birmingham.
Arm J - AZD6738 & Durvalumab	Professor Gary Middleton	Professor of Medical Oncology, University of Birmingham.

Cohort NA1 – Durvalumab (formerly called MEDI4736)<br>*Closed to recruitment*	Professor James Spicer	Consultant Medical Oncologist and Reader in Experimental Oncology, King's College, London.

National Lung Matrix Trial Office

Cancer Research UK Clinical Trials Unit (CRCTU), Institute of Cancer and Genomic Sciences, University of Birmingham, Edgbaston, Birmingham, B15 2TT

Enquiries

☎ 0121 414 7611
✉ lungmatrix@trials.bham.ac.uk
www.birmingham.ac.uk/lungmatrix

Registration

☎ 0121 414 7611 or 0121 414 6788 (9.00 am – 5.00 pm Monday to Friday)
✉ lungmatrix@trials.bham.ac.uk

Serious Adverse Event Reporting

✉ reg@trials.bham.ac.uk

SIGNATURE PAGE

National Lung Matrix Trial Protocol version 11.0 dated 8th September 2021

This protocol has been approved by:

**Name:**	Professor Gary Middleton	**Trial Role:**	Chief Investigator
**Signature:**		**Date:**	08 / Sep / 2021

This protocol describes the National Lung Matrix Trial and provides information about procedures for patients taking part in the National Lung Matrix Trial. The protocol should not be used as a guide for treatment of patients not taking part in the National Lung Matrix Trial.

Protocol written using template CRCTU-PRT-QCD-001 version 1.0

AMENDMENTS

The following amendments and/or administrative changes have been made to this protocol since the implementation of the first approved version:

Amendment number (substantial only)	*Date of protocol amendment*	*Protocol version number*	*Type of amendment*	*Summary of amendment*
1	02-Dec-2014	2.0	Substantial	Section 3.1 Core Inclusion Criteria, the ability to swallow oral medication has been removed, and replaced in the relevant Arm Specific Eligibility Criteria for arms with oral IMPs.<br>Section 3.3 Contraception, reference to an exception for the length of time contraception is required post treatment for Arm E has been added.<br>Section 3.3 Contraception, clarification added that total abstinence as an acceptable method of contraception is only if acceptable if this is the patient's usual and preferred lifestyle choice.<br>Arm D Specific Eligibility Criteria, further details around the exclusion of patients at risk of gastrointestinal perforation have been added, and the exclusion of patients requiring medications that prolong QT interval has been added.<br>Arms A, C and D Restrictions & Concomitant Medications, clarified guidance regarding drugs that may cause QT prolongation.<br>Arm D Toxicity Profile, phototoxicity guidelines have been added.<br>Arm D Dose Modifications, a rule has been added that patients must discontinue crizotinib if they develop gastrointestinal perforation.<br>Arm E Specific Eligibility Criteria, the inclusion of patients agreeing to effective contraception for 6 months after treatment with Docetaxel has been added.
N/A	26-Jan-2015	2.0a	Non-substantial	Page vi, Treatment Arms and Cohorts.<br>Arm C: Palbociclib – clarification of the molecular exclusion criteria.
N/A	07-May-2015	2.0b	Non-substantial	Page I, Updates to Trial Contacts.<br>Page viii, Biopsy Flowchart updated to clarify definition of targeted agent and voluntary post-treatment biopsies.<br>General spelling and inconsistencies corrected throughout document.<br>Correcting of inconsistencies between Schedule of Assessment tables and footnotes for all arms.<br>Schedule of Assessments tables updated for all arms to extend period between registration and treatment commencement to 7 days.<br>Arms A, C & D: Clarification on restricted medication criteria for drugs known to cause QT interval prolongation.

Amendment number (substantial only)	*Date of protocol amendment*	*Protocol version number*	*Type of amendment*	*Summary of amendment*
				Arm C: Palbociclib – clarification of the molecular exclusion criteria.<br>Cohort NA1 – error corrected – missing criteria re systemic disease.
2	N/A	N/A	Substantial – REC only	No change to the protocol. Amendment was to incorporate new participating sites and make changes to the patient information.
N/A	04-Jul-2015	2.0c	Non-substantial	Change logo as per University of Birmingham policy.<br>Page iii, errors corrected.<br>Page ix, Biopsy Flowchart error corrected.<br>Page viii: Table updated.<br>Table 1 updated.<br>Arm C: palbociclib – clarification of the molecular exclusion criteria.<br>Cohort NA1 Schedule errors corrected.<br>Spelling and grammar throughout.
3	02-Sep-2015	3.0	Substantial	Addition of ISRCTN number to front page.<br>Throughout protocol:<br>Spelling and grammar errors corrected;<br>Updated to reflect addition of Arms F and G;<br>Listed references in alphabetical order.<br>Page I – Updates to Trial Contacts.<br>Page ii – Updated Trials Unit Address and added second registration fax number.<br>Page vii-viii – Trial Synopsis:<br>Clarification and amendment of key eligibility criteria.<br>Page ix-xiv – Update to Treatment Arms and Cohorts:<br>Figures 1, 2, 3 and 4 amended to align with other changes and improved formatting;<br>Abbreviations list updated.<br>Section 2.1: Clarification regarding repeat biopsies.<br>Section 3: Clarification re Tier 1 and 2 mutations.<br>Section 4: Addition of Molecular Eligibility Rules section applicable to concomitant genetic changes.<br>Section 5: Table 4 amended to reflect cohort changes.<br>Section 6: Core Eligibility Criteria changes.<br>Section 6.4: Addition of guidance for pleural fluid cytology specimens.<br>Section 7: New process of Screening Registration and Informed Consent section updated.<br>Section 8: Additional instructions included for trial entry process.<br>Section 9: Clarification of IMP ordering; clarification added re CT scanning following treatment discontinuation.<br>Section 10: Clarification of AE reporting procedures.<br>Section 11: Clarification of CRF procedures.

Amendment number (substantial only)	*Date of protocol amendment*	*Protocol version number*	*Type of amendment*	*Summary of amendment*
				Section 12: Additional instructions added for registration forms.<br>Section 14: Clarification that it is 30 evaluable patients per cohort; removal of operating characteristics tables and referral to the Statistical Analysis Plan added.<br>Section 15: Clarification of Trial Organisational Structure.<br>Section 16: Additional information included for Feeder Site local approval.<br>Section 17: Clarification of patient details collected at trial entry.<br>Section 19: Reference to policy added.<br>Section 20: References updated.<br>Arms A-E & NA Cohort 1:<br>Updated background; clarification and update to schedules of assessments; additions of Arm-Specific Performance Status inclusion criteria; update and clarification of Arm-specific Eligibility Criteria; updated Restrictions & Concomitant Medications; update to instructions for oral medication (excl. Arm NA Cohort NA1).<br>Arm B Trial Treatment: AZD2014 dosing amended to intermittent schedule.<br>Arm C: Cohort C5 renumbered to C6, and addition of a new cohort C5.<br>Arm D: Addition of cohort D3 (MET exon 14 splice site mutation/deletion); clarification that Crizotinib is a MET/ROS inhibitor.<br>Arm E Trial Treatment: Addition of instructions regarding continuing treatment if either docetaxel or selumetinib are discontinued for reasons other than progressive disease.<br>Arm A, B & D: Update to Dose Modifications for toxicity.<br>Arm NA Cohort NA1: Update to Toxicity Profile.<br>Arm F supplement: New section added for new trial arm.<br>Arm G supplement: New section added for new trial arm.<br>Appendices: renumbering of previous appendices 2, 3, 5, 6, 7, 8, 9.<br>Appendix 3: Definition of Sites Involved in SMP2 and the National Lung Matrix Trial appendix added.<br>Appendix 10: Credible Meds List of Drugs that prolong QT interval (accessed 19th August 2015) appendix added.<br>Appendix 11: AZD2014 Restrictions and Concomitant Medications appendix added.<br>Appendix 14: AZD5363 Concomitant Treatment Cautions and Restrictions added.<br>Appendix 15: AZD9291 Interactions with Concomitant Medications added.

Amendment number (substantial only)	*Date of protocol amendment*	*Protocol version number*	*Type of amendment*	*Summary of amendment*
N/A	19-Nov-2015	3.0a	Non-substantial	Treatment Arms and Cohorts table amended to reflect the rest of the protocol.<br>Minor clarifications/amendments to all Schedule of Assessments tables.<br>Removal of inapplicable information from Table 10.<br>Minor formatting changes throughout.<br>Arm NA Cohort NA1: Clarification of exclusion criterion regarding repeat biopsy result
4	N/A	N/A	Substantial – MHRA only	No changes to the protocol. Amendment to Reference Safety Information (RSI) document following Development Safety Update Report (DSUR) annual submission.
5	N/A	N/A	Substantial – REC only	No changes to the protocol. Amendment was to add new participating sites and change of PI at 2 existing sites.
6	N/A	N/A	Substantial – REC only	No changes to protocol. Amendment was to change a PI at an existing site.
7	12-Dec-2016	4.0	Substantial	<u>Throughout protocol:</u><br>Name changes of AZD2014 to vistusertib, AZD9291 to osimertinib and MEDI4736 to durvalumab.<br>Addition of non-ECMC treatment site references.<br>LKB1 changed to STK11/LKB1.<br>QTc measurements clarified as QTcF.<br>Arm G (osimertinib) closed to recruitment.<br><u>Section changes:</u><br>Trial contacts: updates to contacts, and secondary registration and fax numbers.<br>Treatment arms and cohorts: Wording updated throughout. ADC cohorts to also include not otherwise specified (NOS) NSCLC patients. C5 addition of patients with dual TSC1/2 and KRAS/NRAS/NFI mutations.<br>Figures 1-4 amended to align with other changes.<br>Section 2.1: Addition that patients can register to multiple cohorts sequentially, if eligible.<br>Section 4 and Tables 3 and 4: Updated in line with arms and cohort changes.<br>Section 5.2: ORR renamed as BOR, Toxicity as Adverse Events and addition of a secondary outcome measure, Durable clinical benefit.<br>Section 6: Core eligibility changes, updated contraceptive guidance and updated definition of sample adequate to submit for testing.<br>Addition that locally obtained genetic results for certain genes may be used if SMP2 panel results fail.<br>Section 9.3: Addition that patients who have PD may continue on therapy if PI and CI agree clinical benefit.<br>Section 10: new translational section added.

Amendment number (substantial only)	*Date of protocol amendment*	*Protocol version number*	*Type of amendment*	*Summary of amendment*
				Section 11.1.2.1: Addition that progression or death due to cancer, and hospitalisations for protocol defined treatment/pre-planned elective procedures/treatment for cancer progression do not meet requirements for SAEs.<br>Section 15.4: Cohort NA1 extended to 30.<br>Updates to Appendices 1, 2, 10, 11, 12 and 15.<br><u>All Arms:</u><br>Addition of numbering of sections and standardised order and titling.<br>Cohort Tables updated to reflect changes.<br>Exclusion criteria washouts of radiotherapy, chemotherapy, investigational agents or other anti-cancer treatment moved to arm-specific exclusion criteria.<br>Section added for Treatment delays and discontinuation.<br>All Arms updated in line with latest version of the IB or pharma guidance documents where applicable: Background and Rationale, Inclusion and Exclusion Criteria, Restrictions and Concomitant Medications, Contraception, Investigational Medicinal Product, Toxicity Profile, and Dosing Modifications and Toxicity Management Guidelines.<br><u>All Arms' Schedules of Assessments</u>:<br>Minor clarifications throughout.<br>Discontinuation visits can occur +7 days of last treatment.<br>CT scans to continue until disease progression, not discontinuation.<br>CT scans to be 6 weekly from treatment start date for the 1st year, and 12-weekly thereafter.<br>Whole blood germline DNA sample added at C1D1 (or the next available timepoint).<br>ctDNA and PBMC timepoint flexibility in line with other blood samples.<br><u>Other Schedule of Assessment changes:</u><br>Arms A & F: discontinuation MUGA removed.<br>Arm B: ECG addition 28 day FUP and removal at C1D1; removal of MUGA/ECHOs during treatment and added at 28 FUP if indicated.<br>Arms B & C: Smoking assessment and ctDNA samples are not required at C2D1.<br>Arm D: Addition of MUGA/ECHO if indicated.<br>Arm E: Addition of ophthalmology at 28 FUP if indicated.<br>Arm G: Addition of ophthalmology throughout if indicated.<br>Cohort NA1: Mandatory repeat biopsy can be >28 days before treatment; additional flexibility of some C1D1 assessments; Table 45 updates so CT frequency is in line with other arms.

Amendment number (substantial only)	*Date of protocol amendment*	*Protocol version number*	*Type of amendment*	*Summary of amendment*
8	N/A	N/A	Substantial – MHRA only	No changes to the protocol. Amendment to Reference Safety Information (RSI) document following Development Safety Update Report (DSUR) annual submission.
9	N/A	N/A	Substantial – REC only	No changes to protocol. Amendment was to add 1 new participating treatment site.
10	04-Jul-2017	5.0	Substantial	<u>Throughout protocol:</u><br>MRIs may be used instead of CT scans.<br>Arm F cohorts to also exclude patients with concomitant NF1, NRAS, HRAS or BRAF aberrations or gene failures.<br><u>Section changes:</u><br>ORR clarified as OR, and clarification of definitions of outcomes. 'Evaluable' patients redefined as 'per protocol'.<br>Core inclusion clarified that patients whose disease has increased but does not meet PD as per RECIST 1.1 are eligible. Patients with true stability are not eligible.<br>Examples of approval genes for local testing removed and reference added to a document which will list these.<br>Addition that Grade ≥2 potential sight-threatening and severe vision loss events for Arm D patients must be reported as Aes,<br><u>All arms:</u><br>Addition of Marketing Experience sections.<br>All inclusion criteria updated to refer to 'Use of Local Testing for SMP2/National Lung Matrix Trial' document to see whether a gene is approved for local testing.<br>Arms B, C, D, F and G updated in line with latest version of the IB or pharma guidance documents where applicable.<br>Arms A, B, C, D, F and G updated excluded QT prolonging concomitant medications.<br><u>Appendices changes:</u><br>Appendix 10, 11 and 14 updated in line with latest guidance documents.<br>Appendix 16 added.
11	29-Aug-2017	5.0a	Substantial MHRA amendment ; Non-substantial REC amendment	Following MHRA notice of non-acceptance of amendment SA10, a minor change was made to the protocol to remove a sentence in section 23.3.4.2 for clarification that Arm B dose reduction levels remain unchanged. The protocol and updated IMP labels were then resubmitted to the MHRA.
12	N/A	N/A	Substantial	No changes to the protocol. Amendment to Reference Safety Information (RSI) document in order to implement for the 4th Development Safety Update Report (DSUR) reporting period.
13	27-Apr-2018	6.0	Substantial	<u>Throughout protocol:</u><br>Addition of Arm H – Sitravatinib.<br>Arm NA Cohort NA1 is closed to recruitment.

Amendment number (substantial only)	*Date of protocol amendment*	*Protocol version number*	*Type of amendment*	*Summary of amendment*
				Cohort B2 separate into patients with (open to recruitment) or without (closed to recruitment) KRAS mutation.<br>Arm F molecular exclusions amended.<br>Addition that patients who refuse standard of consent first line treatment can have Matrix treatment first line.<br>Updated biopsy references to indicate that pre-trial and post-trial optional research biopsies are for the purpose of creating PDX models.<br>Addition of 2 paired optional research pharmacodynamics biopsies in Arm B.<br><u>Section changes:</u><br>15.3: DCB added as a co-primary outcome for Arms A, B, D, E and F.<br>Content from previous Appendices 9, 11, 14 and 15 moved into the Arm-specific concomitant medication guidance sections. Appendices 9-12 renumbered accordingly.<br><u>All Arms:</u><br>Concomitant medication exclusion criteria (Arms A-F) and guidance sections (all Arms) updated and clarified for all arms to highlight importance.<br><u>Arm-specific changes:</u><br>Arm B Schedule of Assessments (SoA): addition of INR and aPTT assessments.<br>Arm G ophthalmic guidance updated.<br>Arm NA Cohort NA1 treatment schedule amended; patients no longer have to stop after 12 months.
14	N/A	N/A	Substantial	No changes to the protocol; addressing IMP queries from MHRA for Arm H addition (MHRA only).
N/A	15-Aug-2018	6.0a	Notification	Change in Data Protection Regulations
15	N/A	N/A	Substantial	No changes to the protocol. Amendment to Reference Safety Information (RSI) document in order to implement for the 5th Development Safety Update Report (DSUR) reporting period.
16	18-Jun-2019	7.0	Substantial	Removal of PDX model biopsies and pharmacodynamics biopsies in Arm B.<br>Change in AZD5363 dose formulation (Arm F).<br>Closure of Arm H.<br>Updated Appendix 9 Credible Meds List of Drugs that Prolong QT Interval
17	N/A	N/A	Substantial	No changes to the protocol. Amendment to Reference Safety Information (RSI) document in order to implement for the 6th Development Safety Update Report (DSUR) reporting period.
18	18-Oct-2019	8.0	Substantial	Implementation of Urgent Safety Measure for Arm C: Palbociclib for pulmonary symptoms indicative of ILD/pneumonitis:<br>• 24.2 Updated Arm C Exclusion Criteria<br>• 24.3 Updated sections on Effects on Respiratory Tract, Dose

Amendment number (substantial only)	*Date of protocol amendment*	*Protocol version number*	*Type of amendment*	*Summary of amendment*
				Modifications,Toxicity Management and Treatment Delays & Discontinuation
19	22-Jan-2020	9.0	Substantial	Closure of Arm A & Arm F<br>Closure of Cohort B1, C2 & D2<br>Updated Appendix 9 Credible Meds List of Drugs that Prolong QT Interval<br>Change to frequency of visits in Arm D after 12 months<br>Addition of Arm J (AZD6738 & Durvalumab)<br>Removal of information from Cohort NA1 section with references to Arm J added<br>Updates to Appendix 12 regarding potential Hy's Law cases
20	N/A	N/A	Substantial	No changes to the protocol. Amendment to Reference Safety Information (RSI) document in order to implement for the 7th Development Safety Update Report (DSUR) reporting period.
21	04-Dec-2020	10.0	Substantial	Closure of Arm B throughout protocol.<br>Change in Palbociclib dose formulation from capsules to tablets (Arm C)<br>Update to concomitant medication advice.<br>Covid-19 pandemic information added.<br>All Open Arm Schedule of Assessment tables updated to reflect that where applicable and acceptable in accordance to local practices, visits may be performed by telephone or video call.<br>Clarification of non-requirement of repeat biopsies for Arm J.<br>Correction of 90% pass rates for Arm J eligibility.<br>Information on the use of alternative validated gene panels to SMP2, Clarification that CT/MRI scans may take place 6-weekly, 8-weekly or 12-weekly depending on treatment arm and time from baseline.<br>Clarification on DCB definition.<br>Core Exclusion criteria clarification.<br>Updated Serious Adverse Events reporting procedure.<br>Arms C, D, E & J - Option for visits to be reduced from 6 to 12 weekly following completion of 12 months of treatment at the discretion of the investigator.<br>All Arms (not NA1): Footnotes of all Schedule of Assessment tables updated to clarify optional post-registration biopsy is pre-treament.<br>Arm E - Addition of anaemia and removal of increased blood pressure as expected side effects of selumetinib.<br>Arm G - updated incidences of various AEs & addition of erythema multiforme (EM) and Stevens-Johnson syndrome (SJS) AEs<br>Arm J -Clarification of Arm J inclusion criteria - patients must weigh 30.1 kg or above for trial entry.

Amendment number (substantial only)	*Date of protocol amendment*	*Protocol version number*	*Type of amendment*	*Summary of amendment*
				Updated to include that AZD6738 tablets must be taken whole with water.<br>Clarification of dose calculation for patients weighing <30.1kg during Arm J treatment.<br>Clarification that blood pressure, pulse rate and temperature assessments taken on day 15 will also comprise a single measurement,<br>Clarification that haematology and clinical chemistry, ureas, electrolytes, full blood count, liver function tests can be taken up to 2 days earlier than the actual visit date (where indicated), with the exception of Cycle 1 Day 1.<br>Clarification that AZD6738 may be dispensed within the IWRS up to 2 days prior to the actual visit date.<br>Addition of general guidance regarding discontinuation.<br>Blood count values changed to /L units for consistency.<br>Further information added on the treatment of neutropenia in Arm J with G-CSF<br>Clarification of Arm J treatment restart parameters following treatment interruption.<br>Clarification of Cohort NAJ nomenclature as a non-actionable cohort receving Arm J treatment<br>Appendices:Updated Appendix 9 Credible Meds List of Drugs that Prolong QT Interval
N/A	22-Feb-2021	N/A	Non-substantial REC amendment	No changes to the protocol. Correction of minor typographical errors to Arms C, E and J PIS+ICFs
SA23	08-Sep-2021	11.0	Substantial	Update to the NHS R&D form to reflect the change of Palbociclib to tablets from their previous capsule form.<br>Clarification that the second intervention to be tested in the NA arm is the Arm J combination of drugs (Cohort NAJ). The statistical design for this NAJ cohort is the same as for cohort J1 on Arm J.<br>Changes to protocol including further clarification of Arm J dose modifications and treatment restart parameters following treatment interruption. Flexibility of -1 day for some cycle 1 day 1 assessments in Arm E and J due to Covid-19 associated clinic visit restrictions.<br>Arm D clarification that although patients on beta-blockers are not excluded from crizotinib treatment, these drugs should be used with extreme caution.<br>Arm E clarification that no dose adjustment is recommended in patients with mild hepatic impairment or with mild, moderate, or severe renal impairment, or those with ESRD.<br>Arm C update to include sepsis as an AE

Amendment number (substantial only)	*Date of protocol amendment*	*Protocol version number*	*Type of amendment*	*Summary of amendment*
				Change to the protocol including the addition of a day 22 visit during cycles 1 and 2 in Arm J. Toxicity information has also been updated for Arm J.<br>The Arm J PIS+ICF has been updated to v3.0 to include an additional visit at Day 22 of treatment cycles 1 and 2.<br>Addition of a footnote to the Arm C, D, E and J Schedule of Assessments to clarify that once a patient has completed 12 months of treatment, visits may be reduced to 12 weekly at the discretion of the Investigator.<br>New PIS+ICF for optional archive tissue for future research.<br>Change of PI at Royal Devon and Exeter with Dr Ian Fraser taking over from Dr Elizabeth Toy as the new PI.<br>Closing Leicester Royal Infirmary and Birmingham Heartlands Hospital as trial sites.<br>Trial end date extended to 30-Sep-2022.

TRIAL SYNOPSIS

Title
National Lung Matrix Trial: Multi-drug, genetic marker-directed, non-comparative, multi-centre, multi-arm phase II trial in non-small cell lung cancer (NSCLC).

Trial Design
Multi-centre, multi-arm, phase II trial, each arm testing an experimental drug in a population stratified by multiple pre-specified target biomarkers, using a Bayesian adaptive umbrella design. The trial also includes an arm for the population with no actionable genetic change who will be treated with a sequential pipeline of drugs.

Trial Objectives
This is a rolling phase II trial of molecularly targeted anti-cancer agents for patients with locally advanced or metastatic NSCLC selected for molecular genotype appropriate to the mechanism of action of each agent, with the purpose:

- To detect whether there is a signal of drug activity sufficient to justify further investigation in that molecularly-defined group.
- To collect tissue linked to clinical outcome data for future exploratory analysis to investigate the molecular genotype of tumours in responding versus non-responding patients and to further develop the clinical diagnostic test necessary to support further investigation of the agent in the relevant molecularly-defined group.
- To identify predictive biomarkers for activity of the various drugs used in the various molecular cohorts.
- To determine the mechanisms of resistance to the target therapies utilised.

Outcome Measures
- Objective response (OR)
- Durable clinical benefit (DCB)
- Best percentage change in sum of target lesion diameters (PCSD)
- Time to progression (TTP)
- Progression-free survival time (PFS)
- Overall survival time (OS)
- Adverse events (AE)

Patient Population
Locally advanced or metastatic NSCLC patients, who have previously consented to molecular profiling of their tumours.

Trial Duration
Minimum of 2 years. There is no current limit on trial duration given that this is a rolling adaptive trial design which explicitly allows for the introduction of new drug/biomarker trial arms during the running of the trial.

Sample Size
The target sample size for each drug-(putative)biomarker cohort is 30 patients but the adaptive design allows decisions to be made with any number of patients. The sample size for each sequential cohort in the no actionable genetic change arm will be determined for each drug individually as they are introduced into the sequential pipeline.

Core Key Inclusion Criteria (not exhaustive - refer to Section 6)
- Prior anti-cancer treatment:

- Patients who refuse any standard of care first line therapy, are eligible to receive National Lung Matrix Trial treatment as first line therapy, providing they explicitly consent to this effect.
- Patients who have previously consented to and received standard of care first line therapy must have completed all standard of care therapy that the treating oncologist thinks is appropriate. As a minimum patients must have failed one or more lines of treatment (either radiological documentation of disease progression or due to toxicity). Patients whose disease has increased in size but is not classed as progressive disease as per RECIST criteria, will be eligible. Patients with no change at all in dimension of disease (i.e. true stability) after first line therapy will not be eligible.
- Patients who have progressed after surgical resection and adjuvant therapy will be eligible for entry without the need for the administration of first line metastatic therapy.
- Patients will also be eligible without the necessity for first line regimen if they have relapsed within 6 months of completion of definitive chemoradiation.

- Consented and provided an adequate specimen to adequately characterise the molecular genotype of the tumour in the molecular pre-screening according to the molecular exclusion rules (see Section 6.4 for definition of an adequate sample).
- Histological or cytologically confirmed NSCLC stage III (not suitable for radical radiotherapy or surgery) or stage IV. This includes patients who may not have clear morphology, but IHC strongly support either squamous cell carcinoma (p63 positivity) or adenocarcinoma (Thyroid transcription factor 1 [TTF1] positivity). If a physician and pathologist are convinced after multi-disciplinary review that the patient has stage III or IV NSCLC but where all the IHC is negative and the morphology does not distinguish a specific sub-type, these patients will be eligible for non-histology specific cohorts.
- Computed Tomography (CT) or Magnetic Resonance Imaging (MRI) scan of head, chest and abdomen within 28 days of treatment demonstrating measurable disease as per Response Evaluation Criteria In Solid Tumours (RECIST) version 1.1 (see Appendix 1) (The same imaging modality must be used throughout treatment).
- Adequate organ function.
- Age ≥ 18 years.
- Provision of signed and dated, written informed consent prior to any study specific procedures, sampling and analyses.

Core Key Exclusion Criteria (not exhaustive - refer to Section 6)

- Major surgery (excluding placement of vascular access) within 4 weeks prior to treatment.
- Nausea, vomiting, chronic gastrointestinal diseases (e.g. inflammatory bowel disease) that would preclude adequate absorption.
- Any psychological, familial, sociological or geographical condition hampering protocol compliance.
- Concurrent malignancies or invasive cancers diagnosed within past 3 years except for adequately treated basal cell carcinoma of the skin and in situ carcinoma of the uterine cervix.
- Pregnant and lactating patients (patients of childbearing potential must have a negative pregnancy test prior to registration).

Trial Office Contact Details

National Lung Matrix Trial Office, Cancer Research UK Clinical Trials Unit
Institute of Cancer and Genomic Sciences, University of Birmingham, Edgbaston B15 2TT

Phone: 0121 414 7611
E-mail: lungmatrix@trials.bham.ac.uk
Website: www.birmingham.ac.uk/lungmatrix

Treatment Arms and Cohorts: Open to Recruitment

Arm	Investigational Medicinal Products	Cohort Number	NSCLC Histology	Molecular Cohort*
C	Palbociclib – CDK-4/6 Inhibitor	C1	SCC	p16 (CDKN2A) loss of function with proficient Rb (no loss of Rb function either by mutation or deletion)
		C3	NSCLC	CDK4 amplification with proficient Rb (no loss of Rb function either by mutation or deletion)
		C4	NSCLC	CCND1 amplification with proficient Rb (no loss of Rb function either by mutation or deletion)
		C5	NSCLC	• STK11/LKB1 mutation, STK11/LKB1 homozygous deletion, TSC1 mutation or TSC2 mutation; AND<br>• Activated KRAS/MAPK pathway i.e. concomitant KRAS, NRAS or NF1 mutation; AND<br>• Proficient Rb (no loss of Rb function either by mutation or deletion)
D	Crizotinib – ALK Inhibitor	D1	NSCLC	MET amplification
		D3	NSCLC	MET exon 14 skipping (splice mutation or deletion)
E	Selumetinib – MEK Inhibitor & Docetaxel	E1	SCC	NF1 mutation
		E2	ADC or NOS NSCLC	NF1 mutation
		E3	NSCLC	NRAS mutation
J	AZD6738 – ATR inhibitor + Durvalumab - PD-L1 Inhibitor	J1	NSCLC	KRAS mutation<br>STK11/LKB1 successful test result<br>$\geq$90% core genes pass rate.
NA		NAJ	NSCLC	No actionable genetic change for other trial arms<br>$\geq$90% core genes pass rate.

***Please refer to protocol Abbreviations table for gene and drug definitions**

National Lung Matrix Trial

Treatment Arms and Cohorts: Closed to Recruitment

Arm	Investigational Medicinal Products	Cohort Number	NSCLC Histology	Molecular Cohort*
A	AZD4547 – FGFR Inhibitor	A1	NSCLC	FGFR2 or FGFR3 mutation
B	Vistusertib – MTORC-1/2 Inhibitor	B1	NSCLC	TSC1 or TSC2 mutation
		B2	NSCLC	STK11/LKB1 mutation or STK11/LKB1 homozygous deletion:<br>a. Patients with no concomitant KRAS mutation; OR<br>b. Patients with a concomitant KRAS mutation.
C	Palbociclib – CDK-4/6 Inhibitor	C2	ADC or NOS NSCLC	p16 (CDKN2A) loss of function with proficient Rb (no loss of Rb function either by mutation or deletion)
		C6	NSCLC	KRAS mutation with proficient Rb (no loss of Rb function either by mutation or deletion)<br>(No concomitant STK11/LKB1 mutation or deletion, no PIK3CA mutation or amplification, no PTEN mutation or homozygous deletion, no AKT mutation, no EGFR mutation, no FGFR2 or FGFR3 mutation, no TSC1 or TSC2 mutation, and no HER2 mutation: Any Tier1 or 2 aberrations will exclude the patient, and some Tier3 aberrations may also exclude the patients - these will be checked on a case by case basis by the National Lung Matrix Trials Office with CRUK and Pfizer).
D	Crizotinib – ALK Inhibitor	D2	NSCLC	ROS1 gene fusions
F	AZD5363 - AKT Inhibitor	F1	SCC	PIK3CA mutation & no aberrations in KRAS, NF1, NRAS, (Tiers 1-2) or HRAS or BRAF (Tier 3)
		F2	SCC	PIK3CA amplification & no aberrations in KRAS, NF1, NRAS, (Tiers 1-2) or HRAS or BRAF (Tier 3)
		F3	NSCLC	PIK3CA mutation or PIK3CA amplification & no aberrations in KRAS, NF1, NRAS, (Tiers 1-2) or HRAS or BRAF (Tier 3) (ADC or NOS NSCLC);<br>PTEN mutation or PTEN loss & no aberrations in KRAS, NF1, NRAS, (Tiers 1-2) or HRAS or BRAF (Tier 3) (ADC or NOS NSCLC);<br>AKT mutation & no aberrations in KRAS, NF1, NRAS, (Tiers 1-2) or HRAS or BRAF (Tier 3) (NSCLC)

Arm	Investigational Medicinal Products	Cohort Number	NSCLC Histology	Molecular Cohort*
		**F4**	SCC	PTEN loss or PTEN mutation & no aberrations in KRAS, NF1, NRAS, (Tiers 1-2) or HRAS or BRAF (Tier 3)
**G**	Osimertinib – EGFR mutation positive T790M+ Inhibitor	**G1**	NSCLC	EGFR mutation & T790M mutation
**H**	Sitravatinib – VEGFR Inhibitor	**H1**	NSCLC	RET rearrangements
**NA**	Durvalumab – PD-L1 Inhibitor	**NA1**	NSCLC	No actionable genetic change for other trial arms

***Please refer to protocol Abbreviations table for gene and drug definitions**

Figure 1: Biopsy Flow Chart (see Section 2 for further detail).

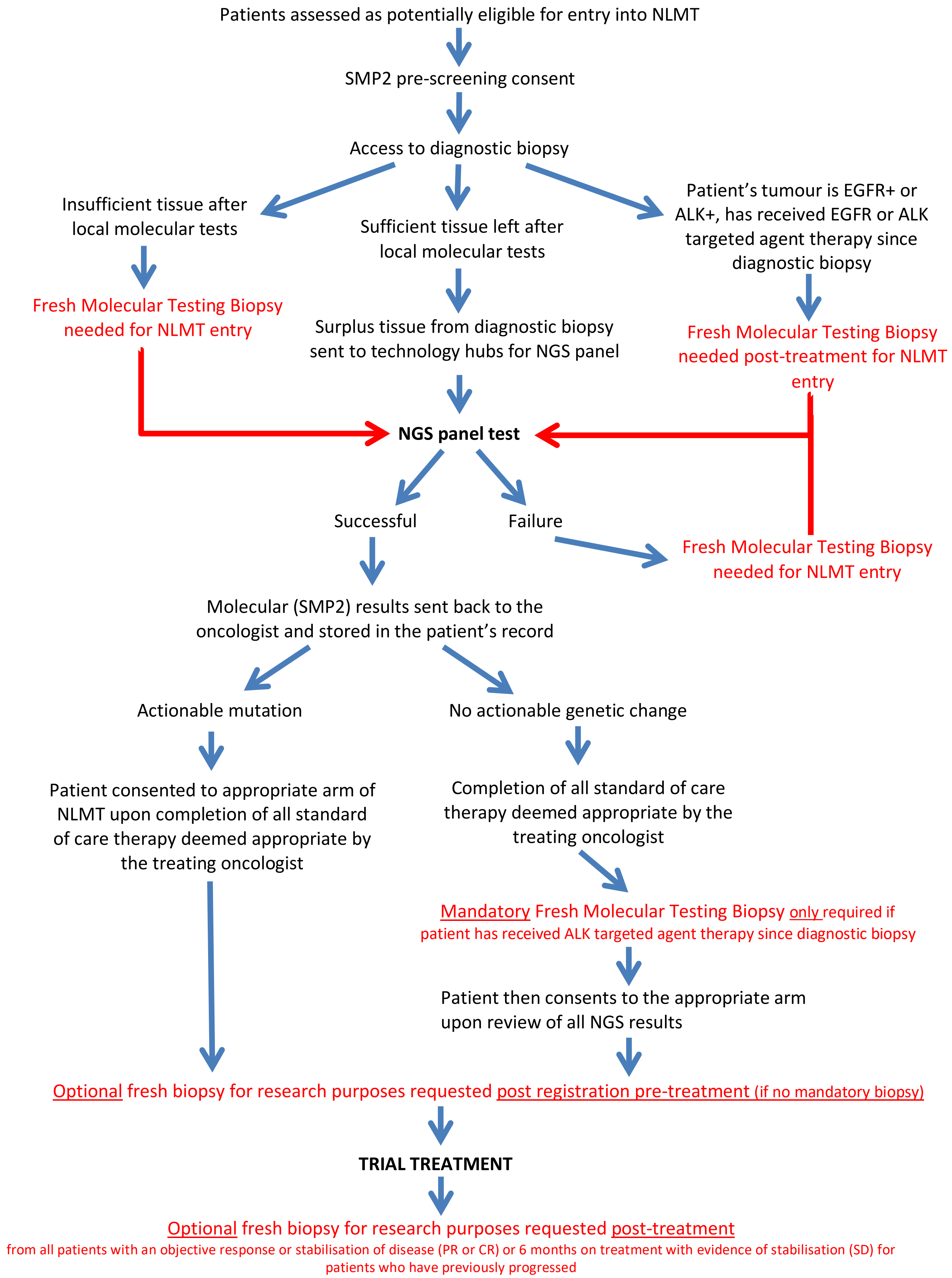

Figure 2: National Lung Matrix Trial Schema

Potentially eligible patient for the National Lung Matrix Trial who has previously consented to diagnostic tissue testing under SMP2

Repeat biopsy required before trial entry:
Biopsy testing failure / not enough tissue left after local testing / patient has received EGFR or ALK targeted agent since diagnostic biopsy / patient has no actionable genetic changes and ≤90% core gene success on NGS panel

Mandatory repeat biopsy collected for molecular testing following patient consent

No repeat biopsy required

Final biopsy result (diagnostic and/or repeat) for National Lung Matrix Trial entry:

- Single actionable target with open treatment arm → Allocated to single treatment arm according to actionable target (if eligible)
- Multiple actionable targets with open treatment arms → Allocated to a single treatment arm according to Molecular Eligibility rules (if eligible). Can be considered for a sequential cohort (if molecularly eligible)
- Actionable targets but no target therapy arms open → Allocated to a no actionable genetic change cohort (if eligible)
- No actionable genetic change → Allocated to a no actionable genetic change cohort (if eligible)

Trial Treatment on one of the following arms:
A (AZD4547), B (Vistusertib), C (Palbociclib), D (Crizotinib), E (Selumetinib & Docetaxel), F (AZD5363), G (Osimertinib), H (Sitravatinib), J (AZD6738 & Durvalumab), NA (Durvalumab)

Figure 3: Squamous cell carcinoma molecular cohorts

Squamous cell carcinoma

Molecular alteration	Cohort	Treatment
FGFR2 or FGFR3 mutation	A1	AZD4547
TSC1 or TSC2 mutation	B1	Vistusertib
STK11/LKB1 mutation or homozygous deletion (without KRAS mutation)	B2	Vistusertib
STK11/LKB1 mutation or homozygous deletion AND KRAS mutation	B2	Vistusertib
p16 (CDKN2A) loss*	C1	Palbociclib
CDK4 amplification*	C3	Palbociclib
CCND1 amplification*	C4	Palbociclib
STK11/LKB1 mutation/homozygous deletion or TSC1/2 mutation AND concomitant KRAS/NRAS/NFI mutation*	C5	Palbociclib
KRAS mutation*	C6	Palbociclib
MET amplification	D1	Crizotinib
ROS1 gene fusions	D2	Crizotinib
MET exon 14 skipping (splice mutation or deletion)	D3	Crizotinib
NF1 mutation	E1	Selumetinib & Docetaxel
NRAS mutation	E3	Selumetinib & Docetaxel
PIK3CA mutation**	F1	AZD5363
PIK3CA amplification**	F2	AZD5363
AKT mutation**	F3	AZD5363
PTEN loss or PTEN mutation**	F4	AZD5363
EGFR mutation & T790M mutation	G1	Osimertinib
RET rearrangements	H1	Sitravatinib
KRAS mutation	J1	AZD6738 & Durvalumab
No actionable genetic change for other trial arms	NAJ	AZD6738 & Durvalumab
No actionable genetic change for other trial arms	NA1	Durvalumab

*Patients must also be Rb wildtype.
**No concomitant aberrations in KRAS, NF1, NRAS, (Tiers 1-2) or HRAS or BRAF (Tier 3).
Note: Greyed out boxes are closed to recruitment.

Figure 4: Adenocarcinoma and NOS molecular cohorts

Adenocarcinoma (ADC) and Not otherwise specified (NOS) non-small cell lung cancer (NSCLC)

<table>
<tr><td>FGFR2 or FGFR3 mutation</td><td>A1</td><td>AZD4547</td></tr>
<tr><td>TSC1 or TSC2 mutation</td><td>B1</td><td rowspan="3">Vistusertib</td></tr>
<tr><td>STK11/LKB1 mutation or homozygous deletion (without KRAS mutation)</td><td rowspan="2">B2</td></tr>
<tr><td>STK11/LKB1 mutation or homozygous deletion AND KRAS mutation</td></tr>
<tr><td>p16 (CDKN2A) loss*</td><td>C2</td><td rowspan="5">Palbociclib</td></tr>
<tr><td>CDK4 amplification*</td><td>C3</td></tr>
<tr><td>CCND1 amplification*</td><td>C4</td></tr>
<tr><td>STK11/LKB1 mutation/homozygous deletion or TSC1/2 mutation AND concomitant KRAS/NRAS/NFI mutation*</td><td>C5</td></tr>
<tr><td>KRAS mutation*</td><td>C6</td></tr>
<tr><td>MET amplification</td><td>D1</td><td rowspan="3">Crizotinib</td></tr>
<tr><td>ROS1 gene fusions</td><td>D2</td></tr>
<tr><td>MET exon 14 skipping (splice mutation or deletion)</td><td>D3</td></tr>
<tr><td>NF1 mutation</td><td>E2</td><td rowspan="2">Selumetinib & Docetaxel</td></tr>
<tr><td>NRAS mutation</td><td>E3</td></tr>
<tr><td>PIK3CA mutation**</td><td rowspan="4">F3</td><td rowspan="4">AZD5363</td></tr>
<tr><td>PIK3CA amplification**</td></tr>
<tr><td>AKT mutation**</td></tr>
<tr><td>PTEN loss or PTEN mutation**</td></tr>
<tr><td>EGFR mutation & T790M mutation</td><td>G1</td><td>Osimertinib</td></tr>
<tr><td>RET rearrangements</td><td>H1</td><td>Sitravatinib</td></tr>
<tr><td>KRAS mutation</td><td>J1</td><td rowspan="2">AZD6738 & Durvalumab</td></tr>
<tr><td>No actionable genetic change for other trial arms</td><td>NAJ</td></tr>
<tr><td>No actionable genetic change for other trial arms</td><td>NA1</td><td>Durvalumab</td></tr>
</table>

*Patients must also be Rb wildtype.
**No concomitant aberrations in KRAS, NF1, NRAS, (Tiers 1-2) or HRAS or BRAF (Tier 3).
Note: Greyed out boxes are closed to recruitment.

ABBREVIATIONS

4E-BP1	4E Binding protein 1
ABPI	Association of British Pharmaceutical Industry
ACC	Acetyl-CoA carboxylase
ADA	Anti-drug antibodies
ADC	Adenocarcinoma
ADL	Activities of daily living
AE	Adverse Event
AESI	Adverse events of special interest
ALK	Anaplastic lymphoma kinase
ALP	Alkaline phosphatase
ALT	Alanine transferase
AML	Acute myeloid leukaemia
AMP	Adenosine monophosphate
AMPK	AMP-activated protein kinase
ANC	Absolute neutrophil count
APC	Antigen presenting cell
APTT	Activated partial thromboplastin time
ASCO	American Society of Clinical Oncology
ASC	Adenosquamous carcinoma
AST	Aspartate transferase
ATP	Adenosine triphosphate
ATV	Atorvastatin
AUC	Area under the curve
BAPN	β-aminoproprionitrite
BCRP	Breast cancer resistance protein
BD/BID	*bis in die*, twice a day
BICR	Blinded independent central review
BP	Blood pressure
bpm	Beats per minute
BSA	Body surface area
BUN	Blood urea nitrogen
CCDC6	Coiled-coil domain-containing protein 6
CD	Cluster of differentiation
CHF	Congestive heart failure
CIOMS	Council for International Organisations of Medical Sciences
CK	Creatine kinase
CLcr	Creatinine clearance
CLR	Renal clearance

Cmax	Maximum concentration
Cmin	Minimum concentration
CNA	Circulating nucleic acid
CNS	Central nervous system
CO	Carbon monoxide
CPK	Creatine phosphokinase
CR	Complete response
CRC	Clinical Research Committee
CRCTU	Cancer Research UK Clinical Trials Unit
CRF	Case Report Form
CRN	Clinical research network
CRUK	Cancer Research UK
CRV	Cerivastatin
CSG	Clinical Studies Group
CT	Computed Tomography
CTC	Circulating tumour cells
ctDNA	Circulating tumour DNA
CTLA	Cytoxic T-lymphocyte antigen
CTX	Circulating tumour cell derived explant tumour
CV	*Curriculum vitae*
CYP	Cytochrome P450
DCA	Data capture aid
DCB	Durable clinical benefit
DCR	Disease control rate
DDI	Drug-Drug interactions
DDLS	Dedifferentiated liposarcoma
DDR2	Discoidin domain-containing receptor 2
DILI	Drug-induced liver injury
DLCO	Carbon monoxide diffusing capacity
DLT	Dose limiting toxicity
DoR	Duration of Response
DTC	Differentiated Thyroid Cancer
EBUS-FNA	Endoscopic bronchial ultrasound-guided fine-needle aspiration
EC_{50}	Concentration that results in 50% effect
ECD	Extracellular domain
ECG	Electrocardiogram
ECHO	Echocardiogram
ECM	Extracellular matrix
ECMC	Experimental Cancer Medicine Centre
ECOG	Eastern Cooperative Oncology Group

EDD	Early Drug Development
EGFR	Epidermal Growth Factor Receptor
EML4	Echinoderm microtubule-associated protein-like 4
EORTC	European Organisation for Research and Treatment of Cancer
EPHA2	Ephrin type-A receptor 2
ER	Oestrogen receptor
ERK	Extracellular signal-regulated kinase
ERBB2	Receptor tyrosine-protein kinase (also known as HER2)
eRDC	electronic Remote Data Capture
ESCRT	Endosomal Sorting Complex Required for Transport
EZE	Ezetimibe
FBC	Full blood count
FDA	Food and Drug Administration
FDG	Fludeoxyglucose
FFPE	Formalin-fixed paraffin embedded
FGFR	Fibroblast growth factor receptor
FISH	Fluorescent *in situ* hybridization
FLT3	fms like tyrosine kinase 3
FLV	Fluvastatin
FOXO3a	Forkhead box O3a
FSH	Follicle stimulation hormone
GAP	GTPase-activating protein
G-CSF	Granulocyte colony stimulating factor
GCP	Good Clinical Practice
GDP	Guanosine diphosphate
GDPR	General Data Protection Regulation
GGT	Gammaglutamyl transferase
GI_{50}	Concentration that results in 50% inhibition of cell growth
GM-CSF	Granulocyte-macrophage colony-stimulating factor
GSK3β	Glycogen Synthase Kinase 3β
GP	General Practitioner
GTP	Guanosine-5'-triphosphate
HbA1C	Glycosylated haemoglobin
HCC	Hepatocellular carcinoma
HER2	Human epidermal growth factor receptor 2 (also known as ERBB2)
HGF	Hepatocyte growth factor
HGFR	Hepatocyte growth factor receptor
HIF	Hypoxia inducible factor
HME	Hot-melt extrusion
HR	Heart rate

HTA	Human Tissue Authority
IB	Investigator Brochure
IC_{50}	Concentration that results in 50% inhibition
IFN	Interferon
Ig	Immunoglobulin
IGF1R	Insulin-like growth factor 1 receptor
IHC	Immunohistochemistry
IMP	Investigational Medicinal Product
IL	Interleukin
ILD	Interstitial lung disease
imAE	Immune-mediated adverse event
INR	International normalised ratio
irRC	Immune-related response criteria
IRS	Insulin receptor substrate
ISF	Investigator site file
ITT	Intent to treat
KDR	Kinase insert domain receptor
KEAP1	Kelch-like ECH-associated protein 1
LCH	Langerhans cell histiocytosis
LC-MS	Liquid chromatography-mass spectrometry
LDH	Lactate dehydrogenase
LFT	Liver Function Test
LH	Luteinizing hormone
LHRH	Luteinizing-hormone-releasing hormone
LKB1	Liver kinase (also known as STK11)
LLN	Lower limit of normal
LoF	Loss of function
LOH	Loss of heterozygosity
LOV	Lovastatin
LOX	Lysyl oxidase
LVEDP	Left ventricular end diastolic pressure
LVEDV	Left ventricular end-diastolic volume
LVEF	Left ventricular ejection fraction
LVESV	Left ventricular end-systolic volume
LWD	Last Weekly Dose
MAPK	Mitogen-activated protein kinase
MCL	Mantle cell lymphoma
MDR1	Multi drug resistance gene 1
MEK	Mitogen activated protein kinase
MHRA	Medicines and Healthcare products Regulatory Agency

MRI	Magnetic resonance imaging
MTD	Maximum tolerated dose
MTORC	Mammalian target of rapamycin complex
MUGA	Multi-gated acquisition scan
NAC	New agents committee
NC	Not calculable
NCI	National Cancer Institute
NCOA4	Nuclear receptor coactivator 4
NCRI	National Cancer Research Institute
NEQAS	National External Quality Assessment Service
NF	Neurofibromin
NGS	Next-generation sequencing
NIHR	National Institute for Health Research
NOAEL	No-observed-adverse-effect level
NOS	Not otherwise specified
NPM	Nucleophosmin
NRAS	Neuroblastoma RAS
NSCLC	Non-small Cell Lung Cancer
NYHA	New York Heart Association
OAE	Other clinically important events
OCT	Optical coherence tomography
OD	*omni die*, once a day
OR	Objective response
ORR	Objective response rate
OS	Overall survival time
PABA	Para-aminobenzoic acid
PBMC	Peripheral blood mononuclear cells
PCSD	Best percentage change in sum of target lesion diameters
PD	Progressive disease
PD-1	Programmed death-1
PDGFR	Platelet-derived growth factor receptor
PDGFRA	Platelet-derived growth factor receptor A
PD-L1	Programmed death ligand-1
PDX	Patient-derived xenograft
pERK	Phosphorylated extracellular signal related kinase
PET	Positron emission tomography
PFS	Progression free survival time
Pgp	P-glycoprotein
PHL	Potential Hy's Law
PK	Pharmacokinetics

PI3K	Phosphatidylinositide 3-kinase
PIK3CA	Phosphatidylinositol-4,5-bisphosphate 3-kinase, catalytic subunit alpha
PR	Partial response
PRAS40	Proline rich AKT substrate of 40kDa
PRP	Platelet rich plasma
PRV	Pravastatin
PS	Performance Status
PST	Potential sight-threatening
PT	Preferred term
PTEN	Phosphatase and tensin homolog
Q2W	Every 2 weeks
QD	*quaque die*, every day
QTc	Corrected QT interval
QTcF	QT interval corrected for heart rate using Fridericia's formula
R&D	Research & Development
RAC	Accumulation rate
RAF	Rapidly accelerated fibrosarcoma
RANKL	Receptor activator of nuclear factor kappa-B ligand
Rb	Retinoblastoma
REC	Research Ethics Committee
RECIST	Response Evaluable Criteria in Solid Tumours
Rheb	Ras homolog enriched in brain
RON	Recepteur d'Origine Nantais
ROV	Rosuvastatin
RP2D	Recommended phase II dose
RPE	Retinal pigmented epithelium
RPED	Retinal pigmented epithelium detachment
RTK	Receptor tyrosine kinase
RVO	Retinal vein occlusion
S6K1	S6 Kinase 1
SAE	Serious adverse event
SAR	Serious adverse reaction
SCC	Squamous cell carcinoma
SCCHN	Squamous cell carcinoma of the head and neck
SCLC	Small cell lung cancer
SD	Stable disease
SGK1	Serum/glucocorticoid regulated kinase 1
sMET	Soluble MET
SMP	Stratified Medicine Programme
SMQ	Standardised MedDRA query

SMV	Simvastatin
SPF	Sun protection factor
StDev	Standard Deviation
STK11	Serine/threonine kinase II (also known as LKB1)
SUSAR	Suspected Unexpected Serious Adverse Reaction
SUV	Standardised uptake values
SVL	Severe Vision Loss
$t_{1/2}$	Half life
TCGA	The Cancer Genome Atlas
TCP	Temporal change parameter
TEAE	Treatment-emergent adverse event
TGI	Tumour growth inhibition
TKB	Tyrosine kinase binding domain
TKI	Tyrosine kinase inhibitors
t_{max}	Median time to maximum plasma concentration
TMG	Trial Management Group
TPGS	Tacopheryl polyethylene glycol 1000 succinate
TSC	Trial Steering Committee
TSC	Tuberous sclerosing complex
TSG	Tumour suppressor gene
TSH	Thyroid stimulating hormone
TTF1	Thyroid transcription factor 1
TTP	Time to progression
ULN	Upper Limit of Normal
US	United States
UV	Ultraviolet
VEGF	Vascular endothelial growth factor
VEGFR	Vascular endothelial growth factor receptor
WBC	White blood cells
WDLS	Well differentiated liposarcoma
Wt	Wildtype

TABLE OF CONTENTS

1 Background and Rationale 1
1.1 Non-Small Cell Lung Cancer 1
1.2 Stratified Medicine Programme 3
2 Trial Biopsies 5
3 Tiered Variants 7
4 Molecular Eligibility Rules 10
4.1 Alternative testing to SMP2 13
5 Trial Design 17
5.1 Overview 17
5.2 Outcome Measures 18
5.2.1 Objective response (OR) 18
5.2.2 Durable clinical benefit (DCB) 18
5.2.3 Best percentage change in sum of target lesion diameters (PCSD) 18
5.2.4 Time to Progression (TTP) 18
5.2.5 Progression-free survival time (PFS) 18
5.2.6 Overall survival time (OS) 19
5.2.7 Adverse Events (AE) 19
6 Core Eligibility Criteria 23
6.1 Core Inclusion Criteria 23
6.2 Core Exclusion Criteria 24
6.3 Contraception 25
6.3.1 Women of childbearing potential 25
6.3.2 Males 26
6.4 Definition of sample adequate to submit for testing 26
7 Screening and Consent 27
7.1 SMP2 Pre-Screening 27
7.2 National Lung Matrix Trial Screening 28
7.2.1 Patients who require a repeat biopsy 28
7.3 Screening Registration Process 28
7.3.1 Patients who do not require a repeat biopsy 29
7.3.2 Patients who require a repeat biopsy 29
7.4 Informed Consent 30
7.4.1 Informed Consent for Mandatory Repeat Biopsies 30
7.4.2 Informed Consent for Trial Participation 31
8 Trial Entry 33
9 Treatment Details 33
9.1 Treatment Compliance 33
9.2 Concomitant Medication 34
9.3 Patient Withdrawal 34
9.4 COVID-19 Pandemic 35
10 Translational research 35
10.1 Germline Blood Sample 35
10.2 ctDNA Samples 36
10.3 PDL1 staining 36
10.4 PBMC samples 36
10.5 Pharmacokinetic samples 36
10.6 Smoking status assessments 37
11 Adverse Event Reporting 37
11.1 Reporting Requirements 37
11.1.1 Adverse Events 37
11.1.2 Serious Adverse Events 37
11.2 Reporting Procedure 39

11.2.1 Site....39
11.2.2 National Lung Matrix Trial Office....40
11.2.3 Reporting to the Competent Authority and main Research Ethics Committee .40
11.2.4 Investigators....41
11.2.5 Trial Management Group (TMG)....41
11.2.6 Trial Steering Committee (TSC)....41
11.2.7 Manufacturer of Investigational Medicinal Product....41
12 Data Handling and Record Keeping....41
12.1 Data Collection....41
12.1.1 Case Report Form....41
12.2 Archiving....42
13 Quality Management....42
13.1 Site Set-up and Initiation....42
13.2 Investigator Meetings....42
13.3 On-site Monitoring....42
13.4 Central Monitoring....43
13.5 Audit and Inspection....43
13.6 Notification of Serious Breaches....43
14 End of Trial Definition....43
15 Statistical Considerations....44
15.1 Statistical Design for Cohorts with Actionable Target (Arms A-J)....44
15.2 Statistical Analysis Plan for Cohorts with Actionable Target (Arms A-J)....44
15.3 Sample Size Justification for Cohorts with Actionable Targets (Arms A-J)....45
15.4 Design, analysis and sample size for no actionable genetic change cohorts (Cohort NA1 & NAJ)....47
15.5 SMP2 Patients who do not enter the National Lung Matrix Trial....47
16 Trial Organisational Structure....47
16.1 Sponsor....47
16.2 Coordinating Centre....47
16.3 Lead Investigators....47
16.4 Trial Management Group....48
16.5 Trial Steering Committee....48
16.6 Independent Peer Review....48
16.7 New Arm Incorporation....48
16.8 Finance....49
17 Ethical Considerations....49
18 Confidentiality and Data Protection....49
19 Insurance and Indemnity....50
20 Publication Policy....50
21 Reference List....50
22 ARM A: AZD4547 – FGFR Inhibitor *(Closed to recruitment)*....52
22.1 Background & Rationale....52
22.1.1 Molecular cohorts....52
22.1.2 Pre-Clinical Rationale....52
22.1.3 Clinical Data....53
22.1.4 Cohort Definition....60
22.2 Specific Eligibility Criteria....63
22.2.1 Inclusion Criteria....63
22.2.2 Exclusion Criteria....63
22.2.3 Restrictions & Concomitant Medications....64
22.3 Trial Treatment....69
22.3.1 Investigational Medicinal Product....69
22.3.2 Schedule of Assessments....71

22.3.3 Toxicity Profile....76
22.3.4 Dosing Modifications and Toxicity Management....82
22.4 Reference List....88
23 ARM B: Vistusertib – MTORC1/2 Inhibitor *(Closed to recruitment)*....89
23.1 Background & Rationale....89
23.1.1 Molecular Cohorts....89
23.1.2 Pre-Clinical Rationale....89
23.1.3 Clinical Data....90
23.1.4 Cohort definition....95
23.2 Specific Eligibility Criteria....99
23.2.1 Inclusion Criteria....99
23.2.2 Exclusion Criteria....99
23.2.3 Restrictions & Concomitant Medications....101
23.3 Trial Treatment....105
23.3.1 Investigational Medicinal Product....105
23.3.2 Schedule of Assessments....107
23.3.3 Toxicity Profile....113
23.3.4 Dose Modifications and Toxicity Management....118
23.4 Reference List....126
24 ARM C: Palbociclib – CDK4/6 Inhibitor....127
24.1 Background & Rationale....127
24.1.1 Molecular Cohorts....127
24.1.2 Pre-Clinical rationale....128
24.1.3 Clinical data....131
24.1.4 Cohort definition....133
24.2 Specific Eligibility Criteria....137
24.2.1 Inclusion Criteria....137
24.2.2 Exclusion Criteria....137
24.2.3 Restrictions & Concomitant Medications....138
24.3 Trial Treatment....141
24.3.1 Investigational Medicinal Product....141
24.3.2 Schedule of Assessments....144
24.3.3 Toxicity Profile....149
24.3.4 Dose Modifications and Toxicity Management....153
24.4 Reference List....160
25 ARM D: Crizotinib – ALK/MET/ROS1 Inhibitor....161
25.1 Background & Rationale....161
25.1.1 Molecular Cohorts:....161
25.1.2 Pre-clinical Rationale....161
25.1.3 Clinical Data....161
25.1.4 Cohort definition....162
25.2 Specific Eligibility Criteria....169
25.2.1 Inclusion Criteria....169
25.2.2 Exclusion Criteria....170
25.2.3 Restrictions & Concomitant Medications....170
25.3 Trial Treatment....174
25.3.1 Investigational Medicinal Product....174
25.3.2 Schedule of Assessments....175
25.3.3 Toxicity Profile....181
25.3.4 Dosing Modifications & Toxicity Management....188
25.4 Reference List....192
26 ARM E: Selumetinib – MEK Inhibitor in combination with Docetaxel....194
26.1 Background & Rationale....194
26.1.1 Molecular cohorts....194

26.1.2 Pre-Clinical Rationale....194
26.1.3 Clinical Data....195
26.1.4 Cohort definition....197
26.2 Specific Eligibility Criteria....198
26.2.1 Inclusion Criteria....198
26.2.2 Exclusion Criteria....199
26.2.3 Restrictions & Concomitant Medications....200
26.3 Trial Treatment....202
26.3.1 Investigational Medicinal Products....202
26.3.2 Non-Investigational Medicinal Products....204
26.3.3 Schedule of Assessments....205
26.3.4 Toxicity Profile....212
26.3.5 Dose Modifications and Toxicity Management....214
26.4 References....229
27 ARM F: AZD5363 - AKT inhibitor (closed to recruitment)....230
27.1 Background & Rationale....230
27.1.1 Molecular cohorts....230
27.1.2 Pre-Clinical Data....230
27.1.3 Clinical Data....231
27.1.4 Cohort Definition....234
27.2 Specific Eligibility Criteria....236
27.2.1 Inclusion Criteria....236
27.2.2 Exclusion Criteria....236
27.2.3 Restrictions & Concomitant Medications....237
27.3 Trial Treatment....246
27.3.1 Investigational Medicinal Product....246
27.3.2 Schedule of Assessments....247
27.3.3 Toxicity Profile....253
27.3.4 Dose Modifications and Toxicity Management....255
27.4 References....260
28 ARM G: Osimertinib – EGFRM+ AND T790M+ Inhibitor *(Closed to recruitment)*....261
28.1 Background & Rationale....261
28.1.1 Molecular cohorts....261
28.1.2 Pre-Clinical Rationale....261
28.1.3 Clinical Data....262
28.2 Specific Eligibility Criteria....263
28.2.1 Inclusion Criteria....263
28.2.2 Exclusion Criteria....263
28.2.3 Restrictions & Concomitant Medications....264
28.3 Trial Treatment....268
28.3.1 Investigational Medicinal Product....268
28.3.2 Schedule of Assessments....269
28.3.3 Toxicity Profile....275
28.3.4 Dose Modifications & Toxicity Management....281
28.4 References....288
29 ARM H: Sitravatinib (MGCD516) - VEGFR Inhibitor *(Closed to Recruitment)*....289
29.1 Background & Rationale....289
29.1.1 Molecular Cohorts....289
29.1.2 Pre-Clinical Rationale....289
29.1.3 Clinical Data....289
29.1.4 Cohort Definition....293
29.2 Specific Eligibility Criteria....294
29.2.1 Inclusion Criteria....294
29.2.2 Exclusion Criteria....295

29.2.3 Restrictions & Concomitant Medications 295
29.3 Trial Treatment...298
29.3.1 Investigational Medicinal Product 298
29.3.2 Schedule of Assessments 299
29.3.3 Toxicity Profile..305
29.3.4 Dose Modifications & Toxicity Management 307
29.4 Reference List 312
30 ARM J & Cohort NAJ: AZD6738 (ATR Inhibitor) & Durvalumab (Anti-PDL1) 313
30.1 Background & Rationale 313
30.1.1 Pre-Clinical Experience 315
30.1.2 Clinical Data 315
30.2 Specific Eligibility Criteria 320
30.2.1 Inclusion Criteria 320
30.2.2 Exclusion Criteria 320
30.2.3 Restrictions & Concomitant Medications 322
30.3 Trial Treatment...327
30.3.1 Investigational Medicinal Product 327
30.3.2 Schedule of Assessments 330
30.3.3 Toxicity Profile..337
30.3.4 Dose Modifications & Toxicity Management 338
30.4 References...376
31 COHORT NA1: Durvalumab – Anti-PDL1 *(Closed to Recruitment)*..........................378
31.1 Background & Rationale 378
31.1.1 Pre-Clinical Rationale 378
31.1.2 Clinical Data 380
31.2 Specific Eligibility Criteria 382
31.2.1 Inclusion Criteria 382
31.2.2 Exclusion Criteria 382
31.2.3 Restrictions & Concomitant Medications 384
31.3 Trial Treatment...385
31.3.1 Investigational Medicinal Product 385
31.3.2 Schedule of Assessments 387
31.3.3 Toxicity Profile..396
31.3.4 Dosing Modifications & Toxicity Management 396
32 Appendix 1: Response Evaluation Criteria in Solid Tumours Version 1.1...............401
33 Appendix 2: Definition of Sites Involved in SMP2 and the National Lung Matrix Trial 405
34 Appendix 3: Common Toxicity Criteria Gradings..406
35 Appendix 4: Cockcroft Gault Formula – Creatinine Clearance.................................407
36 Appendix 5: Incidental Genetic findings policy 408
37 Appendix 6: Definition of Adverse Events 409
38 Appendix 7: WMA Declaration of Helsinki 411
39 Appendix 8: Eastern Cooperative Oncology Group Performance Status Criteria ..414
40 Appendix 9 Credible Meds List of Drugs that Prolong QT Interval 415
41 Appendix 10: New York Heart Association Classification – Stages of Heart Failure 428
42 Appendix 11: Canadian Cardiovascular Society Grading of Angina Pectoris.........429
43 Appendix 12: Actions required in case of increases in liver biochemistry and evaluation of Hy's Law...430
43.1 Introduction 430
43.2 Definitions 430
43.2.1 Potential Hy's Law (PHL):...430

43.2.2 Hy’s Law (HL):..430
43.3 Identification of Potential Hy’s Law (PHL) cases..430
43.4 Follow-up 431
43.5 Review and assessment of Potential Hy’s Law (PHL) cases 431
43.6 References..433
44 Appendix 13: Durvalumab Weight-Based Dose Calculation....................................434

LIST OF TABLES

Table 1: Characteristics of Tier 1 aberrations 7
Table 2: Characteristics of Tier 2 aberrations 9
Table 3: Molecular Eligibility Rules for the National Lung Matrix Trial 14
Table 4: Drug-(putative) biomarker combinations being tested in NSCLC 20
Table 5: Post treatment contraception requirements for female participants 25
Table 6: Post treatment contraception requirements for male participants 26
Table 7: Thresholds for decision-making for each drug 45
Table 8: Tier 1 and Tier 2 actionable mutations for FGFR2 and FGFR3. 61
Table 9: Withdrawal periods for select drugs known to prolong the QT interval 65
Table 10: Withdrawal periods for select drugs known to prolong the QT interval 66
Table 11: Prohibited CYP3A4 or CYP2D6 inhibitors may increase exposure to AZD4547....66
Table 12: Prohibited Inducers of CYP3A4 may reduce exposure to AZD4547......................67
Table 13: Permitted (with caution) moderate Inhibitors of CYP3A4/CYP2D6 may increase exposure to AZD4547...68
Table 14: Prohibited medicines metabolised by CYP3A4 (substrates).................................68
Table 15: Permitted (with caution) medicines metabolised by CYP3A4 (substrates) 69
Table 16: AZD4547 - Schedule of Assessments...71
Table 17: AZD4547 - Frequency of Adverse Events 77
Table 18: AZD4547 - Available dose levels 83
Table 19: Vistusertib Monotherapy (D2270C00001) 90
Table 20: Vistusertib in Combination with fulvestrant (D2270C00005).................................91
Table 21: Prohibited cytochrome P450 and transported inhibitor/inducers..........................102
Table 22: Transport Substrate Restrictions..104
Table 23: Vistusertib - Schedule of Assessments 107
Table 24: Vistusertib Dose Modifications 119
Table 25: Dose modifications and discontinuation criteria for CTCAE Grade 3 or 4 haematological toxicities...119
Table 26: Dose modifications and discontinuation criteria for CTCAE Grade 3/4 non-haematological toxicities* (except liver dysfunction**)..120
Table 27: Palbociclib - Inhibition of Cyclin-Dependent Kinases..128
Table 28: Specific Aberration Rules for Arm C Cohorts. 136
Table 29: Palbociclib - Schedule of Assessments..144
Table 30: ILD/Pneumonitis Management Grades 1-4 155
Table 31: ILD/Pneumonitis Management Following Improvement to Baseline 156
Table 32: Palbociclib - Available Dose Levels..158
Table 33: Palbociclib - Dose Modifications 158
Table 34: NSCLC METEx14 cases reported treated with MET inhibitors with outcomes. ...167
Table 35: Specific aberration rules for Arm D cohorts 169
Table 36: Crizotinib - Schedule of Assessments..175
Table 37: Crizotinib - Available dose levels 189
Table 38: Dose modifications for haematological toxicity[a,b] 190
Table 39: Dose modifications for non-haematological toxicity...190
Table 40: Dose modifications for vision disorder..191
Table 41: Selumetinib - *In vivo* Combination Effects 195
Table 42: Inhibitors of CYP1A2, CYP2C19 or CYP3A4 200
Table 43: Inducers of CYP1A2, CYP2C19 or CYP3A4 200
Table 44: Selumetinib + Docetaxel - Schedule of Assessments 205
Table 45: Selumetinib - Available Dose Levels 215
Table 46: Selumetinib + Docetaxel – Example Topical Steroids and Antibiotics 216
Table 47: CTCAE (version 4) grading for diarrhoea 219
Table 48: Selumetinib + Docetaxel - Assessments recommended for specific AEs 226
Table 49: Selumetinib - Muscular symptoms & CK Elevation...227
Table 50: Duration of treatment for dosing groups of interest 233

Table 51: Frequency of abnormalities in the PI3K/PTEN/AKT pathway in NSCLC 235
Table 52: Prohibited strong CYP3A4 inhibitors may increase exposure to AZD5363 more than 5-fold 238
Table 53: Prohibited potent Inducers of CYP3A4 that may reduce exposure to AZD5363 by more than 5-fold 239
Table 54: Permitted (with caution) moderate Inhibitors of CYP3A4 that may increase exposure to AZD5363............239
Table 55: Prohibited medicines significantly metabolised by CYP3A4 (substrates) 240
Table 56: Permitted (with caution) medicines metabolised by CYP3A4 (substrates) 241
Table 57: Prohibited medicines metabolised by CYP2D6 (substrates) 241
Table 58: Permitted (with caution) medicines metabolised by CYP2D6 (substrates) 242
Table 59: Prohibited medicines metabolised by combined CYP3A4 and CYP2D6 (substrates) 242
Table 60: Permitted (with caution) medicines that are known sensitive substrates of CYP2B6, CYP 2C9 and CYP 2C19............243
Table 61: AZD5363 - Schedule of Assessments............247
Table 62: AZD5363 available dose levels............255
Table 63: Prohibited drugs inducing CYP3A4 265
Table 64: Permitted (with caution) BCRP substrates 266
Table 65: Osimertinib (AZD9291) - Schedule of Assessments 269
Table 66: Dose intervention for osimertinib............281
Table 67: Osimertinib skin effects - rash treatment guidance............282
Table 68: Osimertinib skin effects - dry skin/xerosis treatment guidance 283
Table 69: Osimertinib skin effects - pruritus treatment guidance............283
Table 70: Osimertinib skin effects - paronychia treatment guidance 284
Table 71: Osimertinib related diarrhoea: dietetic measures and pharmacological treatment 286
Table 72: Prohibited & permitted (with caution) sensitive substrates and substrates with a narrow therapeutic index for the indicated CYP enzymes............296
Table 73: Prohibited and permitted (with caution) sensitive substrates and substrates with a narrow therapeutic index for P-gp and BCRP transporters 297
Table 74: Sitravatinib (MGCD516) - Schedule of Assessments (Screening/During Treatment) 299
Table 75: Single Agent Sitravatinib Adverse Drug Reactions............305
Table 76: Sitravatinib dose modifications............308
Table 77: Dose Modifications for Non-Haematological Drug Related Toxicities 309
Table 78: Dose Modifications for Haematological Drug Related Toxicities............309
Table 79: Drugs known to be inhibitors and inducers of CYP3A 323
Table 80: Drugs known to be metabolised by CYP3A4 and have a narrow therapeutic index 323
Table 81: Drugs known to be metabolised by CYP2B6 and have a narrow therapeutic index 323
Table 82: AZD6738 & Durvalumab – Schedule of Assessments............330
Table 83: AZD6738 dose modifications for toxicity management............343
Table 84: General Considerations Regarding Immune-Mediated Reactions............346
Table 85: Durvalumab Specific Immune-Mediated Reactions............348
Table 86: Durvalumab Other-Immune-Mediated Reactions 372
Table 87: Durvalumab Infusion-Related Reactions............374
Table 88: Durvalumab Non - Immune-Mediated Reactions 375
Table 89: Highly Effective Methods of Contraception (<1% Failure Rate)............385
Table 90: Durvalumab (MEDI4736) - Schedule of Assessments............387
Table 91: Durvalumab - Schedule of Assessments: follow up in absence of PD.............395

LIST OF FIGURES

Figure 1: Biopsy Flow Chart (see Section 2 for further detail). xix
Figure 2: National Lung Matrix Trial Schema xx
Figure 3: Squamous cell carcinoma molecular cohorts xxi
Figure 4: Adenocarcinoma and NOS molecular cohorts xxii
Figure 5: Actionable genetic changes in adenocarcinoma of the lung 1
Figure 6: Actionable genetic changes in squamous cell carcinoma of the lung 2
Figure 7: Stratified Medicine Programme 2 workflow 4
Figure 8: Examples of proposed clinical delivery structure 5
Figure 9: Screening registration process flow chart 30
Figure 10: Informed Consent Process Flow Chart 32
Figure 11: FGFR2/3 Tier 1 and Tier 2 mutations 60
Figure 12: AZD4547 - Dose Modifications for Toxicity 84
Figure 13: AZD4547 - Management of Eye Toxicity (Visual Symptoms) 85
Figure 14: AZD4547 - Management of Eye Toxicity (No Visual Symptoms) 87
Figure 15: Vistusertib - AZ GI50 data by TSC1 or 2 status 95
Figure 16: Vistusertib - Sanger data by STK11/LKB1 98
Figure 17: STK11/LKB1 NSCLC xenografts treated with AZD8055 98
Figure 18: Selumetinib - Guidance for the Management of Patients with Rash 217
Figure 19: Selumetinib - Management Guidelines for Patients with Diarrhoea 221
Figure 20: Selumetinib - Management of Asymptomatic LVEF Reduction 222
Figure 21: Selumetinib – Management Guidelines for Patients with Dyspnoea 223
Figure 22: Selumetinib – Management Guidelines for Patients with Visual Symptoms 224
Figure 23: Selumetinib - Management Guidelines for patients with CK elevation 226
Figure 24: Toxicity Management Algorithm 256
Figure 25: AZD5363 - Management of Skin Toxicity 257
Figure 26: AZD5363 - Management of Hyperglycaemia Blood Glucose Intervention Guideline 258
Figure 27: AZD5363 – Management of hepatotoxicity 259
Figure 28: Osimertinib - ILD and Pneumonitis Guidance Flowchart 287
Figure 29: Proof of concept anti-tumour efficacy in NSCLC models exhibiting RTK target alterations (10) 294

1 BACKGROUND AND RATIONALE

Lung cancer is a devastating and very common healthcare problem. It is the most common cancer worldwide with 1.61 million new cases in 2008 (Cancer Research UK Cancer Statistics website). It is also the most common cause of cancer death worldwide with 1.38 million deaths in that same year. In 2010 it was the second most common cancer representing 13% of all cancers in the UK. There were 42,026 new cases and the male to female ratio was almost equal at 12:10. Three quarters of cases are in people over the age of 65. Over the past 25 years the incidence has been steadily decreasing in Europe but in females has gone up by 70% in the same timeframe. The lifetime risk of developing lung cancer is 1 in 14 in men and 1 in 18 in women. The disease usually presents late with less than 25% having stage 1 or 2 disease. In the UK it accounts for 6% of all deaths. It is the most common cause of cancer death representing 22% of cancer death in 2011, where 35,184 people died of the disease. Mortality in men has been decreasing over the past 25 years but in Europe has gone up by 63% and still continues to increase in women.

1.1 Non-Small Cell Lung Cancer

There has been a revolution in the management of NSCLC over the past 10 years. The discovery of the molecular basis of response to Epidermal Growth Factor Receptor (EGFR) tyrosine kinase inhibitors (TKIs) resulted in a paradigm shift in our thinking about this disease, from one dominated by nihilism driven by the experience of the limited activity of toxic chemotherapies to one based on the rational selection of personalised therapy based on the determination of actionable genetic changes. What has been particularly striking is the rapidity of clinical translation in this arena: in 2007 the echinoderm microtubule-associated protein-like (EML)-4-anaplastic lymphoma kinase (ALK) translocation was first described and the phase II data leading to registration of crizotinib appeared in 2011 with comprehensive evaluation of resistance mechanisms just a year later. There was barely a year between the description of RET fusions in a sub-set of NSCLC and the report of responses to RET inhibition in patients harbouring RET fusions. The list of potential actionable genetic changes continues to grow, both in adenocarcinomas and more recently in squamous carcinomas (Figure 5 & Figure 6).

Figure 5: Actionable genetic changes in adenocarcinoma of the lung
(Adapted from Pao & Hutchinson 2012).

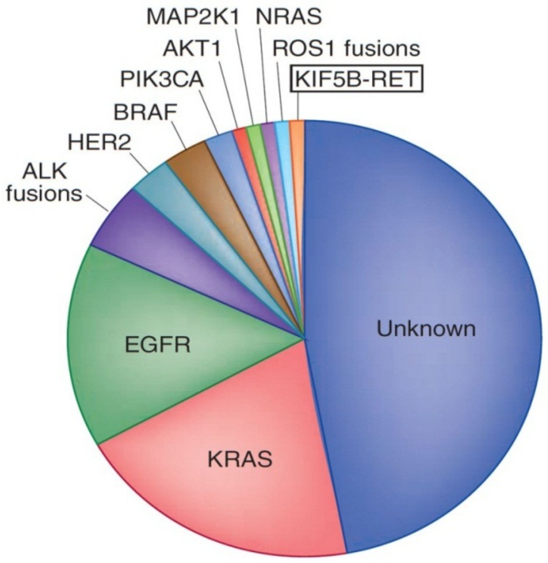

From Figure 5 it will be appreciated that many of these abnormalities each represent small fractions of the overall unstratified population of patients with adenocarcinoma of the lung. ROS1 and RET fusions, for example, each account for less than 2% of non-enriched populations and it is likely that any new actionable genetic changes that are discovered will constitute ever smaller slices of the pie. This makes generating the numbers required for meaningfully powered studies challenging. Furthermore, there are often a number of small molecule inhibitors available that can target any particular pathway or molecule and this multiplies the problem by the number of candidate molecules; diverse agents may target different components within the activated pathway, may be of different drug class, may have different binding specificities and may generate different resistance mechanisms. It is entirely possible that various drugs aimed at the same pathway or molecular target may have very different therapeutic indices and resistance mechanism profiles. All suitable candidates need to be adequately and systematically trialled.

Figure 6 shows that deregulation of both fibroblast growth factor receptor (FGFR)-1 and phosphatidylinositide 3-kinase (PI3K) signalling is a relatively common event in squamous cell carcinoma. PI3K signalling deregulation can be secondary to phosphatidylinositol-4, 5-bisphosphate 3-kinase, catalytic subunit alpha (PIK3CA) mutation, PIK3CA amplification, phosphatase and tensin homolog (PTEN) loss, PTEN mutation or AKT1 mutation. FGFR deregulation can be due to FGFR1 amplification or less frequently FGFR2 or FGFR3 mutation. These diverse mechanisms of deregulation may result in varying sensitivity to any inhibitor and differences in the mechanisms and timing of resistance to that drug. Trialling an inhibitor in a heterogeneous cohort of patients with an actionable deregulated pathway, rather than spanning the entire range of deregulatory mechanisms, may mean that therapeutic differences between sub-sets will be missed, especially with the rarer deregulating abnormalities, which will comprise very few patients in trials which include all patients with pathway deregulation.

Figure 6: Actionable genetic changes in squamous cell carcinoma of the lung (Adapted from Drilon *et al.* 2012).

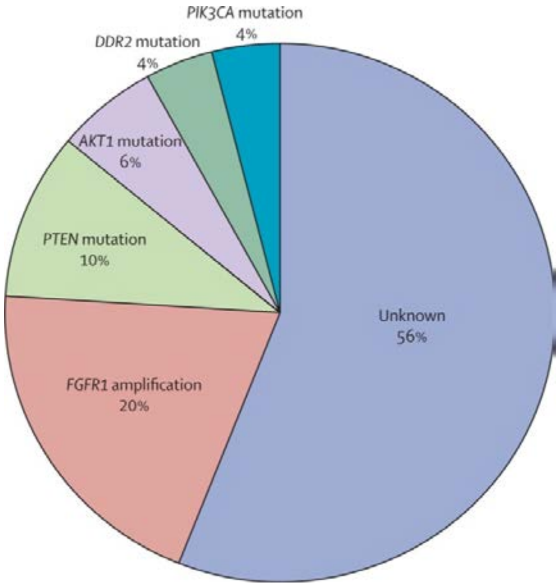

In molecular cohorts for which there are licensed drugs available (gefitinib and erlotinib in those with EGFR mutations and crizotinib in patients with echinoderm microtubule-associated protein-like-4 (EML4)-ALK translocations) resistance is inevitable to these drugs. The mechanisms of resistance are diverse and therapies to abrogate resistance and/or target resistant cell populations, based on post-treatment molecular characterisation, will be mechanism-specific. This again places huge burdens, firstly, in order to define the range of resistance mechanisms specific to any particular mechanism of pathway regulation, for

example those found in patients with FGFR mutations as opposed to those with FGFR amplifications when resistance appears on treatment with FGFR inhibitors. Secondly, in terms of screening for suitable patients, in order to assess the activity of second-line inhibitors or agents added to targeted therapy to reverse resistance, in large enough studies to provide clinically meaningful read-outs, as opposed to anecdotal case reports.

1.2 Stratified Medicine Programme

In 2011 Cancer Research UK set up the Cancer Research UK Stratified Medicine Programme. The first part of the programme (SMP1) was aimed at proving the feasibility of implementing large scale molecular testing in cancer within the NHS whilst receiving consent from patients to use their molecular and routine clinical data for research.

SMP1 officially closed in July 2013: more than 10000 patients among 6 different cancer indications (breast, colorectal, lung, melanoma, ovarian and prostate) were consented across a network of 8 Experimental Cancer Medicine Centres (ECMC) and 26 feeder sites. Slides of formalin-fixed paraffin embedded (FFPE) tumour material and matching blood were then sent to one of three CPA accredited molecular diagnostic labs, or technology hubs. 9010 samples were tested by 3 technology hubs for a range of molecular faults linked to cancer and the results returned to the hospitals with the aim of a turnaround time of 15 working days.

Patients' routine clinical and genetic data was collected from local hospital systems against agreed data standards (COSD, ICD-10, OPCS 4 and SNOMED) and supplied to a central data repository provided by The National Cancer Registration Service – Eastern Office as a managed service. The intention is to make this data available for further research and analysis. The success of SMP1 proved that large volume screening is not only an exciting vision, but an achievable goal in the UK medical landscape.

It is obvious that the greater the number of patients screened, the greater will be the subsequent number of potential participants for trials of inhibitors in small, but important, actionable subsets and for trials of agents to abrogate the multiple routes to resistance of standard of care targeted therapies. This is the simple founding principle underlying the National Lung Matrix Trial aligned to the Cancer Research UK (CRUK) SMP2. This initiative aims to provide large volume national molecular pre-screening integrated with a national clinical trial network to deliver a stratified trial. It builds on the highly successful SMP1 initiative which included 2036 lung cancers, over two years. SMP2 will be only the third truly national screening programme. In the USA the Lung Cancer Mutation Consortium obtains samples from 16 National Cancer Institute (NCI) cancer centres but testing is restricted to 1000 patients per year with a restricted panel of 9 genes analysed per patient. Funding is principally via pharmaceutical partnerships and the programme has demonstrated the ability to enter patients into clinical trials via screening. The French INCa programme has had difficulties with obtaining clinical data sets and with the standardisation of technologies between different pathology departments. SMP2 will be national and will offer a wider gene panel than either the Mutation Consortium or INCa using three genetic technology hubs involved in SMP1 and using an accredited multiplexed technology solution. The workflow is shown diagrammatically in Figure 7. It is proposed to screen 2,000 patients per year with stage IV disease or stage III disease not amenable to surgery or radical radiotherapy using biopsy samples. The Clinical Hubs which will deliver the clinical trial will constitute the ECMC Network and each clinical hub will have a network of feeder sites. Figure 8 gives examples of two clinical delivery networks.

The National Lung Matrix Trial will essentially designate the umbrella structure under which will sit multiple individual trial arms. Each arm will be non-randomised with treatment allocated according to molecular genotype as determined by molecular pre-screening through SMP2 (cohorts). There will be no standard control arm. The exact size of each cohort will vary depending on the nature of the compound (cytostatic versus cytotoxic) and the required level

of activity but will generally be of approximately 30 patients in order to generate the required signal of activity. A key feature will include an optional repeat biopsy at progression for ascertainment of resistance mechanisms and collection of appropriate samples prior to therapy with the targeted agent to reveal potential positive and negative predictive biomarkers for that agent. The study will open at all ECMCs and other UK sites, under a single clinical trial protocol and regulatory submission. The definitions of the sites involved in SMP2 and The National Lung Matrix Trial can be found in Appendix 2: Definition of Sites Involved in SMP2 and the National Lung Matrix Trial.

Figure 7: Stratified Medicine Programme 2 workflow

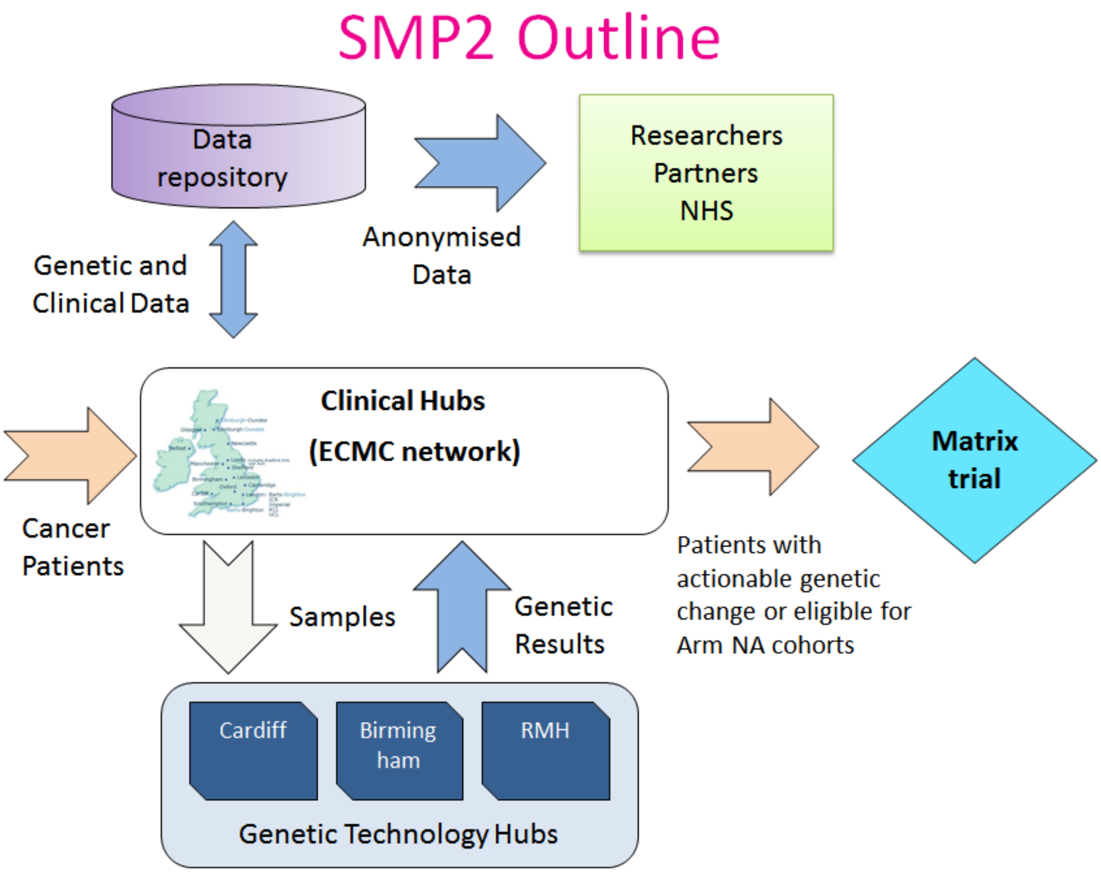

Figure 8: Examples of proposed clinical delivery structure

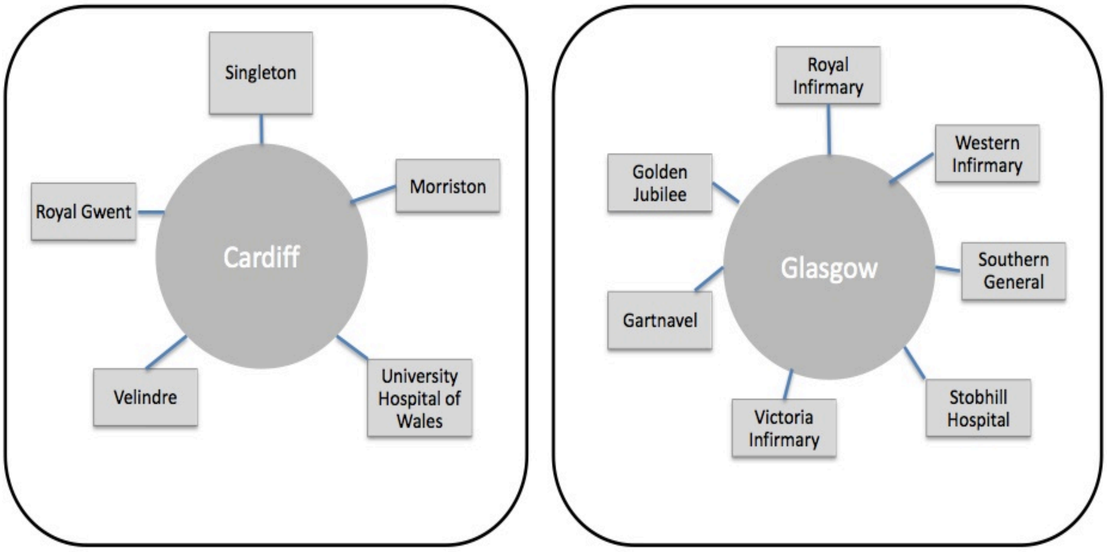

2 TRIAL BIOPSIES

The timing of biopsies that will be performed to identify suitable patients for entry into the National Lung Matrix Trial is summarised in Figure 1 with further detail here.

Entry into the National Lung Matrix Trial will be via two stage consent:

1. Pre-treatment diagnostic biopsy in SMP2
2. After completion of standard of care therapy which the treating oncologist thinks appropriate, and a successful Next Generation Sequencing (NGS) test result

Patients attending an oncology clinic at a feeder site or Clinical Hub with a new diagnosis of metastatic NSCLC or locally advanced disease, where surgery or radiotherapy are deemed not to be appropriate as definitive up-front therapeutic options, and who in the local Investigator's opinion would be potentially suitable for clinical trial entry, will be asked for consent to test their diagnostic (pre-treatment) biopsy on the SMP2 platform.

If the subsequent result shows that they have an actionable target (a genomic change that is an entry criteria for one of the arms open in the National Lung Matrix Trial) they will be informed of this, and when they have completed all standard of care therapy that the treating oncologist thinks is appropriate and at that time satisfy all other eligibility criteria, they will be entered onto the appropriate targeted therapy arm in the trial at the nearest Clinical Hub (or non-ECMC treatment site), without the requirement of a repeat fresh biopsy.

For all other patients who do not have an actionable genetic change for a trial arm in the pre-treatment diagnostic biopsy, or where the genetic analysis of the diagnostic biopsy failed (e.g. insufficient remaining tissue, technical fail; refer to Section 7.3.2), a repeat fresh biopsy for molecular testing upon completion of standard of care therapy will be requested by the local Investigator providing the patient still remains fit for entry into a clinical trial. These repeat biopsies can be undertaken by a NLMT feeder site or at a NLMT treatment site (either a Clinical Hub or non-ECMC treatment site). It is expected that some patients with a successful SMP2 platform analysis of the diagnostic sample but without actionable target, will have developed new genetic aberrations as a result of standard therapy (such as gene copy number changes

or mutations) and will thus become eligible for treatment within one of the cohorts of the National Lung Matrix Trial.

For patients receiving a targeted agent as first line therapy for an actionable target, such as gefitinib for EGFR mutant disease, a new post-first line therapy repeat biopsy for molecular testing is also mandatory for entry onto the National Lung Matrix Trial (see Section 7.3.2).

The design of the trial allows for selected patients to enter multiple cohorts sequentially (see Molecular Eligibility Rules Section 4 of protocol for details). A repeat biopsy for molecular testing may also be required before entering a new treatment cohort due to prior treatment with another targeted therapy within the trial. If no response was seen at the first tumour assessment (6 weeks) when receiving the first trial treatment, a repeat biopsy will not be required. The National Lung Matrix Trial Office should be consulted before any decision is made concerning repeat biopsies for patients being considered for entry in a sequential trial arm.

It is expected that 2000 patients per year as described above will consent to their diagnostic biopsy being tested in SMP2. Some of these patients will fail genetic screening due to inadequate tissue or degraded sample. However, given the impressive range of personalised options available in the National Lung Matrix Trial, it is fully expected that the great majority of these will accept a repeat biopsy for molecular testing, and here all of the tissue obtained will be for testing on the SMP2 platform and not required for diagnostic purposes (e.g. EGFR and ALK testing).

Using data from two recently published randomised maintenance studies we can predict the likely number that will be screened on the diagnostic biopsy but will not be subsequently suitable for consideration of entry into the National Lung Matrix Trial (Paz-Ares *et al.* 2013 and Pe'rol *et al.* 2012). In Paz-Ares 6% did not enter the maintenance phase due to death, 7% due to adverse events and 7% due to subject or Investigator decision, loss to follow up or entry criteria issues. The figures from Pe'rol are highly similar – 7% death, 7% major toxicity and 7% withdrawal of consent, other disease or loss to follow up. Thus 20% of fit patients receiving first line chemotherapy will not be suitable for a second or greater line programme. This leaves 1600 patients from the original 2000. In both studies the progressive disease rate was identical at 23%. It is unlikely that the proportion of initially progressing patients eligible for second or subsequent line therapy will be radically different from those not progressing at this point particularly if a conservative estimate of the likely number that could receive second and later line treatment is made. In Pe'rol the mean salvage therapy usage was 83% and in Paz-Ares 67%. We assume a 60% rate of patients who will be suitable to enter the trial. This is clearly less and it is to be noted that the impetus to deliver and receive later lines of therapy depends critically on what options there are. The National Lung Matrix Trial of personalised care is a scientifically and clinically reasonable option in comparison with other second line therapies currently used. The figure of 60% is very similar to second line usage in Scagliotti, 2008 (mean = 54%): views on subsequent therapy have clearly evolved since that time.

So of 2000 patients entering SMP2 per year we estimate 960 are potentially suitable for the trial. If a one-third to two-thirds split is assumed between squamous and adenocarcinoma (Scagliotti *et al.* 2008) then biomarker prevalence of at least 4.7% and 2.4% relating to squamous and adenocarcinoma respectively will result in recruitment to our target of 30 patients within 2 years.

These figures are clearly for illustrative purposes and details of the projected patient flows for each cohort are included in the Statistical Analysis Plan and these will be monitored and updated as the trial progresses. As well as refining these estimates, the first year of the programme will be highly important in defining failure rates, re-biopsy rates and conversion rates (initially no actionable target on diagnostic biopsy with actionable target on re-biopsy).

3 TIERED VARIANTS

There are 2 groups of aberrations that will be eligible for the National Lung Matrix Trial referred to as 'Tiers'. Aberrations will be classified as Tier 1, Tier 2 or not actionable (Tier 3). For example, Tier 1 aberrations in suppressor genes are considered highly likely to be severely deleterious to gene function either by their nature (e.g. STOP codon) or weight of evidence in the literature or public databases. Tier 1 aberrations are characterised in Table 1. Tier 2 aberrations are considered likely to be deleterious to gene function based on weight of evidence in the literature but with lower certainty than Tier 1. STK11/LKB1 mutation is an example of where there are a significant number of both Tier 1 and Tier 2 aberrations. The majority of the Tier 2 STK11/LKB1 mutations are associated with germline disorder e.g. Peutz-Jeghers rather than being specifically associated with NSCLC. Tier 2 aberrations are characterised in Table 2: Characteristics of Tier 2 aberrations. This rule set will exclude many TCGA aberrations which lack evidence of pathogenic / deleterious effects.

Table 1: Characteristics of Tier 1 aberrations

**Actionable Tier**	**Oncogene (activation)**	**Suppressor (inactivation/Loss of Function [LoF])**<br>*Note: Encompasses any aberration which ablates function of the gene, e.g. point or multi-nucleotide substitutions, insertions and deletions, rearrangements and loss of part of or the entire gene locus, coding or non-coding variants.*
**Tier 1**	**Any mutation/aberration resulting in:**	**Any mutation/aberration resulting in:**
	1. Well characterised activating aberration with demonstrated effects in more than one clinical oncology setting and known to be recurrent.	1. Disruption to a core transcript (preferably with CCDS entry) - frameshift, premature stop, or canonical splice defect (changes to flanking AG and GT recognition sites). Exception is any change beyond a recognised important functional domain (e.g. codon 306 in TP53).
	2. Well characterised aberration with demonstrated clinical evidence of sensitisation to drug with same mode of action.	2. Single exon deletion leading to frameshift.
	3. Coding sequence change known to lead to activation of protein function and/or pathway in at least two different studies using human tumour tissues or *in vivo/ex vivo* models.	3. Single exon deletion (without frameshift) which impacts a known important protein functional domain.
	4. Coding sequence change known to lead to sensitisation to same class of compound in at least two different studies using human tumour tissues or *in vivo/ex vivo* models.	4. Large/gross deletion leading to more than one coding exon being lost.
	5. Coding sequence change reported as "pathogenic" in germline disease setting by at least two independent sources.	5. Change to initiating ATG codon where no other nearby recognised initiation codon is known.

Actionable Tier	Oncogene (activation)	Suppressor (inactivation/Loss of Function [LoF])<br><br>*Note: Encompasses any aberration which ablates function of the gene, e.g. point or multi-nucleotide substitutions, insertions and deletions, rearrangements and loss of part of or the entire gene locus, coding or non-coding variants.*
	**Exceptions:**	6. Well characterized inactivating aberration with demonstrated effects in more than one clinical oncology setting.
	Mutation affecting an alternative exon in a non CCDS transcript where knowledge of function of that transcript is limited.	7. Previously reported as “clinically relevant” or “pathogenic” in germline setting by multiple sources (3 or more reports required).
	Mutation must be VERY RARE (<0.5%) in recognised human variation databases (e.g. 1000 genomes populations).	8. Any variant demonstrated to have dominant negative effect in germline by at least two sources.
		9. Any missense variant with 10 or more reports as **somatic** mutation.
		**Exceptions:**
		Frameshift, truncation, or exon skipping near C-terminus of protein and not affecting a known important functional domain.
		Mutation affecting an alternative exon in a non CCDS transcript where knowledge of function of that transcript is limited.
		Clear single heterozygous loss in a gene where complete loss is believed to be required for abrogation of function (i.e. no obvious dominant negative effect, double hit presumed to be required to drive cancer).
		Mutation must be VERY RARE (<0.5%) in recognised human variation databases (e.g. 1000 genomes populations).

Table 2: Characteristics of Tier 2 aberrations

Actionable Tier	Oncogene (activation)	Suppressor (inactivation/LoF)<br>*Note: Encompasses any aberration which ablates function of the gene, e.g. point or multi-nucleotide substitutions, insertions and deletions, rearrangements and loss of part of or the entire gene locus, coding or non-coding variants.*
**Tier 2**	1. Any mutation fulfilling **at least one** of the following criteria:	1. Missense change with robust evidence for **at least one** of the following effects:
	a. Coding change demonstrated to be sensitizing/activating for the relevant pathway in at least one human subject in an experimental oncology trial.	a. Significant loss of expression of mRNA (>80% reduction in detectable mRNA levels) in human tissue or human cell line.
	b. Coding change in a codon known to be an activation hotspot.	b. Significant loss of expression of protein (>80% reduction in detectable protein) in human tissue or cell line.
	c. Recurrent confirmed somatic coding change observed in at least three different tumour samples (may be from public data sources, commercially acquired samples or samples from other clinical trials).	c. Significant loss of protein function (>80% reduction in protein activity in a recognised functional assay) performed in human tissue or human cell line).
	d. Coding sequence change known to enhance sensitivity to compound in at least two different established experimental models, e.g. xenografts, primary disease explants, organoids or appropriate cell lines.	d. Reported as causative for a recognised germline disorder with dominant negative effect.
	e. Coding sequence change reported to activate/enhance protein function in a relevant *in vitro* functional assay.	e. Significant increase in drug sensitivity for a cell line, xenograft or primary disease explant model harbouring the mutation.
	f. Coding sequence change reported as "pathogenic" in germline disease by one or more sources.	f. Functionally validated splicing mutation within an intron (unless in-frame skipping of an exon not known to be in a protein functional domain).
	g. Coding sequence change reported as "likely pathogenic" by at least two independent sources.	g. Experimentally confirmed dominant negative effect on wild-type protein function.
		h. Missense with five (5) or more independent reports in recognised germline disease database.
	**Exceptions:**	
	Mutation affecting an alternative exon in a non CCDS transcript where knowledge of function of that transcript is limited.<br>Mutation must be VERY RARE (<0.5%) in recognised human variation databases (e.g. 1000 genomes populations).	

4 MOLECULAR ELIGIBILITY RULES

The following strategy will apply to concomitant genetic changes (summarised in Table 3). This strategy cannot be exhaustive due to the complexities of the molecular read-out and biological rationale for stratification.

It is also important to note that patients may have concomitant genetic changes and may be eligible for cohorts in more than one arm. In order to maximise the biological information collected throughout this trial, these patients can be treated sequentially.

Please contact the National Lung Matrix Trial Office for confirmation if applicable. The order of treatment arms, if applicable, will be considered by the Chief Investigator.

The National Lung Matrix Trial Office will assign patients to arms/cohorts during the screening registration process (see Section 7.3).

PI3K mutations
It is to be noted that all concomitant KRAS, NF1, NRAS mutations (Tiers 1-2), or HRAS or BRAF mutations (Tier 3) will be excluded from this group as there is cell line data that demonstrate that KRAS mutation is a negative predictor for sensitivity.

Patients with dual PIK3CA and TSC1/2 mutations are eligible for treatment with vistusertib (cohort B1) and AZD5363 (cohorts F1/3) sequentially. These patients will first be treated with vistusertib (Arm B); mTOR sits downstream of PI3K and therefore is a more natural target for this dual mutation.

PIK3CA amplifications
It is to be noted that all concomitant KRAS, NF1, NRAS mutations (Tiers 1-2), or HRAS or BRAF mutations (Tier 3) will be excluded from this group as there is cell line data that demonstrate that KRAS mutation is a negative predictor for sensitivity.

PTEN aberrations
It is to be noted that all concomitant KRAS, NF1, NRAS(Tiers 1-2), or HRAS or BRAF mutations (Tier 3) will be excluded from this group as there is cell line data that demonstrate that KRAS mutation is a negative predictor for sensitivity.

AKT mutations
It is to be noted that all concomitant KRAS, NF1, NRAS mutations (Tiers 1-2), or HRAS or BRAF mutations (Tier 3) will be excluded from this group as there is cell line data that demonstrate that KRAS mutation is a negative predictor for sensitivity.

FGFR2/3 mutations
There are a minority of FGFR mutations in SCC that have concomitant PIK3CA or KRAS mutations. These patients will all be treated with the FGFR inhibitor AZD4547 (Arm A), and are excluded from entry into cohort C6 (palbociclib). It is entirely feasible that one or other of these mutations by downstream pathway activation may negatively affect activity of AZD4547 but as yet we have not evidence that this is the case.

Patients with dual FGFR2/3 and TSC1/2 mutations are eligible for treatment with vistusertib (B1) and AZD4547 (A1) sequentially. These patients will be treated with vistusertib (Arm B) initially, in view of the impressive anecdotal responses to mTOR inhibitors in patients with TSC1/2 mutations.

STK11/LKB1 mutations
In the TCGA there is no co-occurrence with other actionable mutations for SCC harbouring STK11/LKB1 mutations.

Half of ADC patients with STK11/LKB1 mutations have concomitant KRAS mutations. It is important to note that patients with dual STK11/LKB1 and KRAS mutations are eligible for treatment with vistusertib (B2) and palbociclib (C5).

Patients with dual STK11/LKB1 and NRAS mutations are eligible for treatment with vistusertib (B2 – now closed to recruitment), palbociclib (C5) and selumetinib (E3).

Patients with dual STK11/LKB1 and NF1 mutations are eligible for treatment with vistusertib (B2 – now closed to recruitment), palbociclib (C5), and selumetinib (E1/2).

A STK11/LKB1 successful test result must be available for patients eligible for treatment with AZD6738 & durvalumab (J1).

TSC1/2 mutations
In SCC there is occasional concomitant PI3K mutation. Patients with dual TSC1/2 and PI3KCA mutations are eligible for treatment with vistusertib (B1) and AZD5363 (F1) sequentially. These patients will be treated with vistusertib (B1) initially as mTOR activated by TSC1 loss is downstream of PI3K.

Patients with dual TSC1/2 and KRAS mutations are eligible for treatment with vistusertib (B1) and palbociclib (C5).

Patients with dual TSC1/2 and NRAS mutations are eligible for treatment with vistusertib (B1), palbociclib (C5) and selumetinib (E3).

Patients with dual TSC1/2 and NF1 mutations are eligible for treatment with vistusertib (B1), palbociclib (C5), and selumetinib (E1/2).

KRAS mutations
Dual KRAS and STK11/LKB1 mutants are eligible for treatment with vistusertib (B2) and palbociclib (C5).

Dual KRAS and TSC1/2 mutants are eligible for treatment with vistusertib (B1) and palbociclib (C5).

KRAS mutants are also eligible for treatment with AZD6738 & durvalumab (J1) if a successful test result is also available for STK11/LKB1 and a pass rate of 90%≥ of the core genes (see core genes section below).

This leaves dual PIK3CA/PTEN/AKT and KRAS mutants not eligible for an actionable cohort in the trial. These patients can be considered for entry into Arm NA.

Any patient with a KRAS mutation deemed to be potentially eligible for treatment with palbociclib in cohort C6 must have no mutation resulting in activation of AKT as this inhibits the re-induction of senescence in KRAS – mutant adenocarcinoma with CDK4 knockdown. Patients with dual KRAS mutation with: STK11/LKB1 mutation or homozygous deletion; PIK3CA mutation or amplification; AKT mutation; PTEN mutation or loss; EGFR mutation; HER2 mutation; FGFR2/3 mutation; or TSC1/2 mutation, will not be eligible for treatment with palbociclib in cohort C6.

NRAS mutations
Patients with dual TSC1/2 and NRAS mutations are eligible for treatment with vistusertib (B1), palbociclib (C5) and selumetinib (E3).

Patients with dual STK11/LKB1 and NRAS mutations are eligible for treatment with vistusertib (B2 – closed to recruitment), palbociclib (C5) and selumetinib (E3).

NF1 mutations
In the original TCGA data for adenocarcinoma (Ding *et al.* 2008) there is some overlap of NF1 mutation and KRAS mutation. These patients will be treated with selumetinib/docetaxel given the published data demonstrating activity of this combination in KRAS mutant patients.

Patients with concomitant NF1 and STK11/LKB1 mutations will be eligible for treatment with vistusertib (B2 – closed to recruitment), palbociclib (C5) and selumetinib (E1/2).

Patients with concomitant NF1 and TSC1/2 mutations will be eligible for treatment with selumetinib/docetaxel (E1/2), palbociclib (C5), and vistusertib (B1).

Core genes
Patients being considered for cohort J1 and no actionable genetic change cohorts require a successful SMP2 result, which is defined as ≥90% of core genes* successfully reported in the Technology Hub Report. A successfully reported gene must confirm the presence or absence of a gene change and exclude failed genes. This can be either from the diagnostic sample or a repeat biopsy sample.

The 52 core genes are as follows:

AKT1	CCND3	FGFR2	NRAS	RAD54L
ALK	CCNE1	FGFR3	**NRG1**	RB1
ARID1A	CDK12	Her2	NTRK1	RET
ATM	CDK4	HRAS	PALB2	ROS1
BARD1	CDKN2A	KRAS	PIK3CA	STK11/LKB1
BRAF	CHEK1	MET	**PMS2**	**TP53**
BRCA1	CHEK2	**MLH1**	PPP2R2A	TSC1
BRCA2	**CTNNB1**	**MSH2**	PTEN	TSC2
BRIP1	EGFR	**MSH3**	RAD51B	
CCND1	**ERCC1**	**MSH6**	RAD51C	
CCND2	FANCL	NF1	RAD51D	

Genes highlighted above in **bold** will only be reported in phase 2 of the TST 170 panel release.

*The core genes list will differ depending on which version of the panel is in use by the Technology Hubs at the point of testing. For clarity:

- Samples tested under the original 28 gene panel will require a pass rate of 25 genes (90%).
- In phase 1 of the TST 170 panel release (implemented December 2019) 43 genes are analysed and reported, all of which are core genes, therefore a 90% pass rate of those 43 genes is required (39 genes).
- Samples tested and fully reported under phase 2 of the TST 170 panel (170 genes) require a pass rate of 47 core genes (90% of 52 core genes).

Other
All amplifications and rearrangements will be first treated with the targeted agent appropriate to them (CDK4 and CCND1 amplifications with palbociclib, Met amplifications and ROS fusions with crizotinib, and PIK3CA amplifications with AZD5363), irrespective of concomitant mutations.

4.1 Alternative testing to SMP2

Patients whose samples have been tested on an alternative validated gene panel or single mutation, rearrangement or copy number change (tissue or blood is accepted), and not SMP2, are considered eligible if they meet the molecular eligibility rules stated above. Due to the 90% gene pass rate requirement, only patients who come through SMP2 are eligible for Arm J, unless all the 28 (old panel) or 52 genes listed above are tested. Prior to patient consent and submission of screening registration, evidence of test validation must be supplied to the NLMT Trial Office for approval. Evidence of molecular eligibility and histology must be provided in a printed format for each patient, as is done with SMP2.

Table 3: Molecular Eligibility Rules for the National Lung Matrix Trial

Arm	IMPs	Cohort	Histology	Molecular Cohort	Molecular Eligibility Rules
**A**	AZD4547<br>*Closed to recruitment*	**A1**	NSCLC	FGFR2 or FGFR3 mutation	FGFR and TSC1/2 dual mutations are eligible for treatment with vistusertib (B1) and AZD4547 (A1) sequentially.<br>FGFR and KRAS dual mutations will be treated with AZD4547 (A1). These patients are excluded from treatment with palbociclib (C6).<br>FGFR and PIK3CA dual mutations are eligible for treatment with AZD4547 (A1) and AZD5363 (F1/3) sequentially.
**B**	Vistusertib<br>*Closed to recruitment*	**B1**	NSCLC	TSC1 or TSC2 mutation	TSC1/2 and KRAS dual mutations are eligible for treatment with vistusertib (B1), and palbociclib (C5).<br>TSC1/2 and NRAS dual mutations are eligible for treatment with vistusertib (B1), palbociclib (C5), and selumetinib (E3).<br>TSC1/2 and NF1 dual mutations are eligible for treatment with vistusertib (B1), palbociclib (C5), and selumetinib (E1/2).<br>PIK3CA and TSC1/2 dual mutations are eligible for treatment with vistusertib (B1) and AZD5363 (F1/3) sequentially.
		**B2**	NSCLC	STK11/LKB1 mutation or STK11/LKB1 homozygous deletion:<br>a. Patients with no concomitant KRAS mutation (CLOSED TO RECRUITMENT); OR<br>b. Patients with a concomitant KRAS mutation.	STK11/LKB1 and KRAS dual mutations are eligible for treatment with vistusertib (B2) and palbociclib (C5).<br>STK11/LKB1 and NRAS dual mutations are eligible for treatment with vistusertib (B2 – closed to recruitment), palbociclib (C5), and selumetinib (E3).<br>STK11/LKB1 and NF1 dual mutations are eligible for treatment with vistusertib (B2 – closed to recruitment), palbociclib (C5), and selumetinib (E1/2).
**C**	Palbociclib	**C1**	SCC	p16 (CDKN2A) loss	Rb-proficient (with no loss of Rb function either by mutation or deletion)
		**C2**<br>*Closed to recruit-ment*	ADC or NOS NSCLC	p16 (CDKN2A) loss	Rb-proficient (with no loss of Rb function either by mutation or deletion)
		**C3**	NSCLC	CDK4 amplification	Rb-proficient (with no loss of Rb function either by mutation or deletion)
		**C4**	NSCLC	CCND1 amplification	Rb-proficient (with no loss of Rb function either by mutation or deletion)

Arm	IMPs	Cohort	Histology	Molecular Cohort	Molecular Eligibility Rules
		**C5**	NSCLC	STK11/LKB1 mutation, STK11/LKB1 homozygous deletion, TSC1 mutation or TSC2 mutation with activated KRAS/MAPK pathway	Rb-proficient (with no loss of Rb function either by mutation or deletion) & concomitant KRAS, NRAS or NF1 mutation
		**C6** *Closed to recruit-ment*	NSCLC	KRAS mutation	Rb-proficient (with no loss of Rb function either by mutation or deletion)<br>No concomitant STK11/LKB1 mutation or homozygous deletion, PIK3CA mutation or amplification, no PTEN mutation or homozygous deletion, no AKT mutation, no EGFR mutation, no FGFR2/3 mutation, no TSC1/2 mutation, and no HER2 mutation.<br>KRAS and NF1 dual mutations are eligible for treatment with palbociclib (C6) and selumetinib (E1/2) sequentially.<br>KRAS and NRAS dual mutations are eligible for treatment with palbociclib (C6) and selumetinib (E3) sequentially.
**D**	Crizotinib	**D1**	NSCLC	MET amplification	N/A
		**D2** *Closed to recruit-ment*	NSCLC	ROS1 gene fusions	N/A
		**D3**	NSCLC	MET exon 14 skipping (splice mutation or deletion)	N/A
**E**	Selumetinib & Docetaxel	**E1**	SCC	NF1 mutation	N/A
		**E2**	ADC or NOS NSCLC	NF1 mutation	N/A
		**E3**	NSCLC	NRAS mutation	N/A
**F**	AZD5363 *Closed to recruitment*	**F1**	SCC	PIK3CA mutation & no aberrations in KRAS, NF1, or NRAS (Tiers 1-2), or HRAS or BRAF (Tier 3).	No concomitant aberrations in KRAS, NF1, or NRAS (Tiers 1-2), or HRAS or BRAF (Tier 3).
		**F2**	SCC	PIK3CA amplification & no aberrations in KRAS, NF1, or NRAS (Tiers 1-2), or HRAS or BRAF (Tier 3).	No concomitant aberrations in KRAS, NF1, or NRAS (Tiers 1-2), or HRAS or BRAF (Tier 3).

Arm	IMPs	Cohort	Histology	Molecular Cohort	Molecular Eligibility Rules
		**F3**	NSCLC	PIK3CA mutation or PIK3CA amplification & no aberrations in KRAS, NF1, or NRAS (Tiers 1-2), or HRAS or BRAF (Tier 3). (ADC or NOS NSCLC)<br>PTEN mutation or PTEN loss & no aberrations in KRAS, NF1, or NRAS (Tiers 1-2), or HRAS or BRAF (Tier 3) (ADC or NOS NSCLC)<br>AKT mutation and no aberrations in KRAS, NF1, or NRAS (Tiers 1-2), or HRAS or BRAF (Tier 3). (NSCLC)	No concomitant aberrations in KRAS, NF1, or NRAS (Tiers 1-2), or HRAS or BRAF (Tier 3).
		**F4**	SCC	PTEN loss or PTEN mutation & no aberrations in KRAS, NF1, or NRAS (Tiers 1-2), or HRAS or BRAF (Tier 3).	No concomitant aberrations in KRAS, NF1, or NRAS (Tiers 1-2), or HRAS or BRAF (Tier 3).
**H**	Sitravatinib<br>*Closed to recruitment*	**H1**	NSCLC	RET rearrangements	N/A
**G**	Osimertinib<br>*Closed to recruitment*	**G1**	NSCLC	EGFR mutation & T790M mutation	N/A
**J**	AZD6738 & Durvalumab	**J1**	NSCLC	KRAS mutation	STK11/LKB1 succesful test result.<br>>90% core genes pass rate.

5 TRIAL DESIGN

5.1 Overview

The trial schema is shown in Figure 2, Figure 3 and Figure 4. The trial consists of a series of parallel multi-centre single arm phase II trial arms, each testing an experimental targeted drug in a population stratified by multiple pre-specified actionable target putative biomarkers (see Table 4 for all drug-(putative)biomarker combinations). The primary objective is to evaluate whether there is a signal of activity in each drug-(putative)biomarker cohort separately. A Bayesian adaptive umbrella design is adopted to achieve this objective and statistical details are given in Section 15.

The trial is primarily an enrichment putative biomarker design, including patients who are positive for at least one of the actionable targets included in the trial. Patients who are positive for just one putative biomarker will receive the experimental targeted drug specific for that putative biomarker. Putative biomarkers within each drug cohort have been chosen such that in the majority of cases it is not expected that patients will be positive for two or more putative biomarkers within the same drug. In the rare situation that patients are positive for two or more putative biomarkers relevant across different drugs, treatment will be allocated in accordance with the following strategy:

- All amplifications and rearrangements will be treated with targeted agent appropriate to them irrespective of concomitant mutations. This will yield crucial predictive biomarker information.
- For concomitant mutations decisions will be made by the Chief Investigator on a case-by-case basis and based on close consideration of pathway preference and likely dominance of one signal pathway over another together with any pre-clinical efficacy studies that address the activity of the drugs in the presence of concomitant mutations (see Section 4.). If appropriate, patients can be considered for a sequential cohort if molecularly eligible.

A secondary objective of the trial is to provide the opportunity for industrial partners to test novel agents in the cohort of patients who are not positive for any of the actionable targets in the trial, referred to as no actionable genetic change cohorts. In particular, if interim analysis shows significant activity for one of the targeted drugs in a targeted group then it may be relevant to assess the putative biomarker specificity of this drug by including it as a treatment option as a no actionable genetic change cohort. Such an assessment could be important to inform the design of future trials for that drug. During the course of the trial, any drugs that are selected as a no actionable genetic change cohort will be included in a pipeline of options that become available sequentially. The first drug to be tested is durvalumab (Cohort NA1). No comparison whatsoever will be made between any agents tested in a no actionable genetic change cohort.

The following patients who have consented to SMP2 screening and follow up will not be allocated to any open trial arm but will receive standard treatment or possibly treatment under an alternative trial protocol:

- Their diagnostic biopsy and subsequent repeat molecular testing biopsies did not produce an adequate specimen or the NGS platform was unsuccessful.
- Following failure of certain genes to be sequenced by the SMP2 panel (please refer to Use of Local Test Result for SMP2/Matrix document), the use of local molecular test results could also not be used to provide an aberration result.
- The patient is found to be ineligible following consent to an arm in the National Lung Matrix Trial.

- The patient's tumour has no actionable genetic change as per the current trial protocol and there is no available non-actionable genetic change cohort available to treat them.

These patients could be considered as a concurrent control group and although no formal comparison will be made with patients treated in the National Lung Matrix Trial, outcome measures for these patients will be collected in order to provide relevant concurrent background information.

5.2 Outcome Measures

The trial includes a common set of outcome measures (listed below) that will be measured in all drug-(putative)biomarker cohorts with primary outcome measures selected from the common set specifically for each cohort.

5.2.1 Objective response (OR)

Patients will have regular CT or MRI scans from baseline until disease progression (6-weekly, 8-weekly or 12-weekly depending on treatment arm and time from baseline) . On each occasion, overall tumour burden will be assessed using RECIST version 1.1 (Eisenhauer *et al.* 2009). Best overall response is the best response recorded over the whole period of assessment and could be complete response (CR), partial response (PR), stable disease (SD), progressive disease (PD) or inevaluable for response (for which reasons such as early death due to disease or early death due to toxicity will be specified). Objective response is the occurrence of CR or PR as the best overall response. Objective response will be based on responses confirmed using the subsequent scans but objective response based on unconfirmed responses will also be reported.

5.2.2 Durable clinical benefit (DCB)

A patient will be defined as experiencing DCB if they remain free of disease progression at their scan taken approximately 24 weeks since treatment start date. Patients that discontinue trial treatment, but remain progression-free at their first scan at or after 24 weeks and have not started a non-protocol treatment, will be counted as having achieved DCB. Patients that discontinue trial treatment before the 24 week time point and are either lost to follow-up or begin a non-protocol treatment, will be included in the denominator for the DCB analysis but counted as having not met the threshold for successful DCB.

5.2.3 Best percentage change in sum of target lesion diameters (PCSD)

At each evaluation, the longest diameters of all selected target lesions will be measured and summed and the percentage change from the baseline measurement will be calculated. The best percentage change is the one that reflects either the greatest decrease or the least increase over the whole period of assessment.

5.2.4 Time to Progression (TTP)

This is defined as the time from commencement of trial treatment to the date of the CT or MRI scan when progressive disease first recorded. Patients with no recorded progression at the time of analysis or who die without recorded progression will be censored at the date of the scan when they were last recorded with an evaluable measure that was not progression.

5.2.5 Progression-free survival time (PFS)

This is defined as the time from commencement of trial treatment to the date of CT or MRI scan when progressive disease first recorded or date of death without previously recorded progression. Patients who are alive with no recorded progression at the time of analysis will be censored at the date of the scan when they were last recorded with an evaluable measure that was not progression.

5.2.6 Overall survival time (OS)

This is defined as the time of commencement of trial treatment to the date of death. Patients who are alive at the time of analysis will be censored at the date last seen alive.

5.2.7 Adverse Events (AE)

Adverse events and adverse reactions will be recorded in relation to each cycle of treatment and graded according to Common Terminology Criteria for Adverse Events (CTCAE) (see Appendix 3: Common Toxicity Criteria Gradings and Appendix 6: Definition of Adverse Events).

Table 4: Drug-(putative) biomarker combinations being tested in NSCLC

**Molecular Cohorts and Initial Estimated Prevalence Rates**<br>**See Statistical Analysis Plan for derivation of prevalence rates**		Arm A: AZD4547 *Closed to recruitment*	Arm B: Vistusertib *Closed to recruitment*	Arm C: Palbociclib	Arm D: Crizotinib	Arm E: Selumetinib & Docetaxel	Arm F: AZD5363 *Closed to recruitment*	Arm G: Osimertinib *Closed to recruitment*	Arm H: Sitravatinib *Closed to recruitment*	Arm J: AZD6738 & Durvalumab
A1: FGFR2 or FGFR3 mutation – NSCLC<br>*(Closed to recruitment)*	ADC <1.0%<br>SCC 4.0%	✓								
B1: TSC1 or TSC2 mutation – NSCLC<br>*(Closed to recruitment)*	ADC <1.0%<br>SCC 2.7 %		✓							
B2: STK11/LKB1 mutation or homozygous deletion – NSCLC:<br>a. Patients with no concomitant KRAS mutationOR<br>b. Patients with a concomitant KRAS mutation<br>*(Closed to recruitment)*	ADC 8.8%<br>SCC 1.6%		✓							
C1: p16 loss (CDKN2A) & proficient Rb – SCC	SCC 29.0%			✓						
C2: p16 loss & proficient Rb – ADC and NOS NSCLC<br>*(Closed to recruitment)*	ADC 19.6%			✓						
C3: CDK4 amplification & proficient Rb – NSCLC	ADC 7.0%<br>SCC <1.0%			✓						
C4: CCND1 amplification & proficient Rb – NSCLC	ADC 5.0%<br>SCC 12.0%			✓						
C5: STK11/LKB1 mutation or homozygous deletion, or TSC1 or TSC2 mutation, with activated KRAS/MAPK pathway (i.e. concomitant KRAS, NRAS or NF1 mutation), & proficient Rb - NSCLC	ADC 4.4%<br>SCC 0.8%			✓						

Molecular Cohorts and Initial Estimated Prevalence Rates<br>See Statistical Analysis Plan for derivation of prevalence rates		Arm A: AZD4547<br>*Closed to recruitment*	Arm B: Vistusertib<br>*Closed to recruitment*	Arm C: Palbociclib	Arm D: Crizotinib	Arm E: Selumetinib & Docetaxel	Arm F: AZD5363<br>*Closed to recruitment*	Arm G: Osimertinib<br>*Closed to recruitment*	Arm H: Sitravatinib<br>*Closed to recruitment*	Arm J: AZD6738 & Durvalumab
C6/I1: KRAS mutation & proficient Rb – NSCLC<br>*(Closed to recruitment)*	NSCLC 25.8%*			✓						
D1: MET amplification – NSCLC	ADC 2.7%<br>SCC 1.4%				✓					
D2: ROS1 gene fusions – NSCLC<br>*(Closed to recruitment)*	ADC 1.7%<br>SCC <1.0%				✓					
D3: MET exon 14 skipping (splice mutation or deletion) - NSCLC	NSCLC 3.0%				✓					
E1: NF1 mutation – SCC	SCC 5.8%					✓				
E2: NF1 mutation – ADC and NOS NSCLC	ADC 4.6%					✓				
E3: NRAS mutation – NSCLC	NSCLC 1.0%					✓				
F1: PIK3CA mutation & no aberrations in KRAS, NF1, NRAS (Tiers 1-2), or HRAS or BRAF (Tier 3) – SCC<br>*(Closed to recruitment)*	SCC 11.0%						✓			
F2: PIK3CA amplification & no aberrations in KRAS, NF1, NRAS (Tiers 1-2), or HRAS or BRAF (Tier 3) - SCC<br>*(Closed to recruitment)*	SCC 15.0%						✓			
F3: PI3K/AKT deregulation:	ADC 2.0%						✓			

**Molecular Cohorts and Initial Estimated Prevalence Rates**<br>**See Statistical Analysis Plan for derivation of prevalence rates**		Arm A: AZD4547 *Closed to recruitment*	Arm B: Vistusertib *Closed to recruitment*	Arm C: Palbociclib	Arm D: Crizotinib	Arm E: Selumetinib & Docetaxel	Arm F: AZD5363 *Closed to recruitment*	Arm G: Osimertinib *Closed to recruitment*	Arm H: Sitravatinib *Closed to recruitment*	Arm J: AZD6738 & Durvalumab
PIK3CA mutation or amplification & no aberrations in KRAS, NF1, NRAS (Tiers 1-2), or HRAS or BRAF (Tier 3) (ADC and NOS NSCLC);<br>PTEN mutation or loss & no aberrations in KRAS, NF1, NRAS (Tiers 1-2), or HRAS or BRAF (Tier 3) (ADC and NOS NSCLC);<br>AKT mutation & no aberrations in KRAS, NF1, NRAS (Tiers 1-2), or HRAS or BRAF (Tier 3) (NSCLC)<br>*(Closed to recruitment)*	ADC 3.0%<br><br>ADC 0.5%<br>SCC 0.5%									
F4: PTEN loss or mutation & no aberrations in KRAS, NF1, NRAS (Tiers 1-2), or HRAS or BRAF (Tier 3) (SCC)<br>*(Closed to recruitment)*	SCC 20.0%						✓			
G1: EGFR mutation & T790M mutation – NSCLC<br>*(Closed to recruitment)*	ADC 8.0%<br>SCC <1.0%							✓		
H1: RET rearrangements<br>*(Closed to recruitment)*	NSCLC 1.2%								✓	
J1: KRAS mutation	NSCLC 25.8%									✓

* Prevalence rate is for total KRAS mutant population and does not account for concomitant genetic changes excluded from this cohort.
NSCLC – non small cell lung cancer; ADC – adenocarcinoma; SCC – squamous cell carcinoma

6 CORE ELIGIBILITY CRITERIA

Core inclusion and exclusion criteria are presented below. Additional inclusion/exclusion criteria apply to each arm and are presented in the relevant arm supplement.

6.1 Core Inclusion Criteria

- Prior anti-cancer treatment:
    - Patients who refuse any standard of care first line therapy, are eligible to receive National Lung Matrix Trial treatment as first line therapy, providing they explicitly consent to this effect.
    - Patients who have previously consented to and received standard of care first line therapy must have completed all standard of care therapy that the treating oncologist thinks is appropriate. As a minimum patients must have failed one or more lines of treatment (either radiological documentation of disease progression or due to toxicity). Patients whose disease has increased in size but is not classed as progressive disease as per RECIST criteria, will be eligible. Patients with no change at all in dimension of disease (i.e. true stability) after first line therapy will not be eligible.
    - Patients who have progressed after surgical resection and adjuvant therapy will be eligible for entry without the need for the administration of first line metastatic therapy.
    - Patients will also be eligible without the necessity for first line regimen if they have relapsed within 6 months of completion of definitive chemoradiation.
- Consented and provided an adequate specimen to adequately characterise the molecular genotype of the tumour in the molecular pre-screening according to the molecular exclusion rules (see Section 6.4 for definition of an adequate sample).
- Histological or cytologically confirmed NSCLC stage III (not suitable for radical radiotherapy or surgery) or stage IV. This includes patients who may have abnormal histology, but IHC strongly support either squamous cell carcinoma (p63 positivity) or adenocarcinoma (Thyroid transcription factor 1 [TTF1] positivity). If a physician and pathologist are convinced after multi-disciplinary review that the patient has stage III or IV NSCLC but where all the IHC is negative and the morphology does not distinguish a specific sub-type, these patients will be eligible for non-histology specific cohorts.
- CT or MRI scan of head, chest and abdomen within 28 days of treatment demonstrating measurable disease as per RECIST version 1.1 (see Appendix 1: Response Evaluation Criteria in Solid Tumours Version 1.1). (The same imaging modality must be used throughout treatment).
- Adequate haematological function within 7 days of treatment.
    - Haemoglobin ≥ 90 g/L.
    - Absolute neutrophil count (ANC) ≥ 1.5 x 10^9/L.
    - Platelets ≥ 100 x 10^9/L.
- Adequate hepatic function within 7 days of treatment in patients with no liver metastasis (see arm specific entry criteria for adequate hepatic function in patients **with** liver metastases).
    - Total serum bilirubin ≤ 1.5 x upper limit of normal (ULN). (Note that this will not apply to patients with confirmed Gilbert's syndrome (persistent or recurrent hyperbilirubinemia that is predominantly unconjugated in the absence of evidence of haemolysis or hepatic pathology), who may be allowed inclusion at the discretion of the local Investigator).
    - Alanine transferase (ALT) ≤ 2.5 x ULN.
    - Aspartate transferase (AST) ≤ 2.5 x ULN.
- Adequate renal function within 7 days of treatment.

- Creatinine clearance (CLcr) ≥50 ml/min (measured or calculated by Cockcroft and Gault equation – see Appendix 4: Cockcroft Gault Formula – Creatinine Clearance). If calculated CLcr is <50 ml/min a direct measurement of glomerular filtration rate (GFR) such as EDTA may be performed. If the value is ≥50 ml/min the patient is eligible.
- Age ≥ 18 years.
- Females must agree to use adequate contraceptive measures (as defined in Section 6.3), should not be breast feeding and must have a negative pregnancy test prior to start of dosing if of child-bearing potential or must have evidence of non-child-bearing potential by fulfilling one of the following criteria at screening:
    - Post-menopausal defined as aged more than 50 years and amenorrhoeic for at least 12 months following cessation of all exogenous hormonal treatments
    - Documentation of irreversible surgical sterilisation by hysterectomy, bilateral oophorectomy or bilateral salpingectomy but not tubal ligation.
    - Women aged under 50 years old would be consider postmenopausal if they have been amenorrhoeic for 12 months or more following cessation of exogenous hormonal treatments and with luteinizing hormone (LH) and follicle stimulating hormone (FSH) levels in the post-menopausal range for the institution.
- Provision of signed and dated, written informed consent prior to any study specific procedures, sampling and analyses.

6.2 Core Exclusion Criteria

- Major surgery (excluding placement of vascular access) within 4 weeks prior to treatment.
- Nausea, vomiting, chronic gastrointestinal diseases (e.g. inflammatory bowel disease) that would preclude adequate absorption.
- Any psychological, familial, sociological or geographical condition hampering protocol compliance.
- Concurrent malignancies or invasive cancers diagnosed within past 3 years except for adequately treated basal cell carcinoma of the skin and in situ carcinoma of the uterine cervix.
- Judgement by the local Investigator that the patient should not participate in the study if the patient is unlikely to comply with study procedures, restrictions and requirements.
- Any unresolved toxicity of grade 2, 3 or 4 from previous treatment (excluding alopecia) at Registration (see CTCAE - Appendix 3: Common Toxicity Criteria Gradings). Replacement therapy for immune related adverse events with normalisation of endocrine function will not be excluded,
- Patients who have previous symptomatic brain metastases or spinal cord compression are excluded unless they have had adequate treatment, no evidence of progression or symptoms, and have had no requirement for steroid treatment in the previous 28 days before commencement of trial treatment.
- Patients with asymptomatic brain metastases picked up at screening CT scan are not excluded providing that in the view of the local Investigator they do not require immediate radiotherapy or surgical intervention, and have had no requirement for steroid treatment in the previous 28 days before commencement of trial treatment.
- As judged by the local Investigator, any evidence of severe or uncontrolled systemic diseases, including active bleeding diatheses, or active infection including hepatitis B, hepatitis C and human immunodeficiency virus. Screening for chronic conditions is not required.
- Pregnant and lactating patients (patients of childbearing potential must have a negative pregnancy test prior to registration).

Cardiac exclusion criteria, performance status and prior treatment washout periods are detailed within the National Lung Matrix Trial arm-specific eligibility criteria.

Arm-specific eligibility criteria can be found in the following arm supplements:

Arm A: AZD4547: Section 22.2
Arm B: Vistusertib: Section 23.2
Arm C: Palbociclib: Section 24.2
Arm D Crizotinib: Section 25.2
Arm E Selumetinib & Docetaxel: Section 26.2
Arm F AZD5363: Section 27.2
Arm G Osimertinib: Section 28.2
Arm H Sitravatinib: Section 29.2
Arm J and cohort NAJ: AZD6738 & Durvalumab: Section 30.2
Cohort NA1 Durvalumab: Section 31.2

6.3 Contraception

This section should be read in conjunction with any additional guidance in individual arm supplements.

6.3.1 Women of childbearing potential

Females of childbearing potential are defined as those who are not surgically sterile (ie, bilateral tubal ligation, bilateral oophorectomy, or complete hysterectomy) or post-menopausal.

Women will be considered post-menopausal if they have been amenorrheic for 12 months without an alternative medical cause. The following age-specific requirements apply:

- Women <50 years of age would be considered post-menopausal if they have been amenorrheic for 12 months or more following cessation of exogenous hormonal treatments and if they have luteinizing hormone and follicle-stimulating hormone levels in the post-menopausal range for the institution or underwent surgical sterilization (bilateral oophorectomy or hysterectomy).
- Women ≥50 years of age would be considered post-menopausal if they have been amenorrheic for 12 months or more following cessation of all exogenous hormonal treatments, had radiation-induced menopause with last menses >1 year ago, had chemotherapy-induced menopause with last menses >1 year ago, or underwent surgical sterilisation (bilateral oophorectomy, bilateral salpingectomy or hysterectomy).

Adequate contraception should be used from the time of screening, during the study and for a defined period following completion of treatment:

Table 5: Post treatment contraception requirements for female participants

Arm and IMP	Contraception period post treatment completion
A (AZD4547), D (crizotinib), G (osimertinib), cohort NA1 (durvalumab), J and cohort NAJ (AZD6738/durvalumab)	At least 90 days
C (palbociclib)	At least 21 days
E (docetaxel), H (sitravatinib)	At least 6 months

B (vistusertib), E (selumetinib), F (AZD5363)	At least 4 weeks

Acceptable methods of contraception (except for Arms B, F and cohort NA1 – please see arm-specific guidance) include total abstinence (if this is the patient's usual and preferred lifestyle choice), tubal ligation, combined oral, transdermal or intra-vaginal hormonal contraceptives, medroxyprogesterone injections (e.g. Depo-provera), copper-banded intra-uterine devices; hormone impregnated intra-uterine systems and vasectomised partners. All methods of contraception (with the exception of total abstinence) should be used in combination with the use of a condom by their male sexual partner for intercourse.

NB. Some investigational drugs may have a drug-drug interaction with hormonal contraceptives precluding use of the latter. Specific guidance will be provided in the individual drug supplements which must be adhered to.

6.3.2 Males

Male patients with sexual partners who are pregnant or who could become pregnant (i.e. women of child-bearing potential) should use a condom during sexual intercourse or abstain from sexual intercourse (if this is the patient's usual and preferred lifestyle choice), during the study and for a defined period following completion of treatment:

Table 6: Post treatment contraception requirements for male participants

**Arm and IMP**	**Contraception period post treatment completion**
A (AZD4547), C (palbociclib), D (crizotinib), G (osimertinib), cohort NA1 (durvalumab)	At least 90 days
E (selumetinib)	At least 12 weeks
B (vistusertib), F (AZD5363)	At least 16 weeks
E (docetaxel) , H (sitravatinib), J and cohort NAJ (AZD6738 & durvalumab)	At least 6 months

Further details regarding contraception are available in the arm-specific Contraception sections of the protocol.

6.4 Definition of sample adequate to submit for testing

The tissue sample provided for genetic testing should meet the following criteria:

- Biopsy or paraffin-embedded cytology cell block specimen with surplus tissue available following histopathology and any additional tests required as part of standard care, biobanking activity or participation in a clinical trial. Samples containing less than 20% tumour will not be accepted. Macrodissection for tumour enrichment is recommended.
- Origin from either the primary tumour or a site of metastasis.
- Matching blood sample required. Fresh 4 ml blood sample in EDTA tube can be provided within 48 hours otherwise frozen or extracted DNA of quantity 15 µl of 5 ng/µl.

Sections from the following sample types will be accepted:

- Bronchoscopic, percutaneous or surgical biopsies from primary tumour, lymph node or other metastases including visceral, cerebral and bone (EDTA is an acceptable decalcification method).

- Paraffin-embedded cell blocks from endoscopic bronchial ultrasound fine needle aspiration/biopsy (EBUS FNA/B) samples from tumour/lymph nodes or malignant effusions.
- Resections for late-stage disease (stage IIIA or greater).

DNA remaining after local molecular testing (e.g. for EGFR or ALK) may be acceptable for analysis. Any site intending to submit extracted DNA should in the first instance discuss this with the lead scientist at the relevant SMP2 molecular genetics laboratory/Technology Hub (i.e. Birmingham, Cardiff or Royal Marsden/ICR).

Cytology fluids cannot be accepted for the study, however, DNA extracted from sections derived from a paraffin-embedded cell block of pleural fluid cytology specimens are suitable for testing on the NGS panel. A paraffin block must be made with cut sections and an assessment of tumour percentage. It is important to note that DNA from cytology specimens is not acceptable for patients being considered for Arm NA Cohort NA1 who are undergoing a mandatory repeat biopsy for molecular testing.

Locally obtained genetic results from approved laboratories, may be used to confer molecular eligibility for certain selected genes that fail testing on the SMP2 NGS panel. Approval will be based, in part, on NEQAS performance. Please refer to the current version of the document entitled ‘Use of Local Test Result for SMP2/National Lung Matrix Trial’ for further information on approved laboratories and accepted genes.

7 SCREENING AND CONSENT

7.1 SMP2 Pre-Screening

Patients attending oncology clinic with a diagnosis of metastatic NSCLC or locally advanced disease where surgery or radiotherapy are deemed not to be appropriate as definitive up-front therapeutic options and who in the local Investigator’s opinion would be potentially suitable for clinical trial entry (PS 0-2, absence of serious co-morbidities) will be asked for consent to test their diagnostic (pre-treatment) biopsy on the SMP2 platform. It is to be noted that trial entry patients may have a differing PS to that stipulated in SMP2 Eligibility Criteria. Patients with a PS of 2 may improve following first line chemotherapy, and some arms include patients with a PS 0-2 (see arm-specific eligibility criteria). Therefore their diagnostic sample will be tested. This will include patients with a new diagnosis of lung cancer and those who have commenced therapy. They will also be asked to allow the entry of routine clinical data onto a central pseudonymised database, to be linked to the molecular information derived from their tumour. The approved pre-screening consent form and patient information sheet (or a local consent form and PIS, where used) will be used to obtain consent for SMP2. Clinical Hubs with established biobanks and approved consent forms and PIS to use tissue for research can use these forms, upon review and approval by CRUK.

Patients with cancers of the lung who give consent will have a sample of their diagnostic tumour block extracted, under the supervision of a trained histopathologist, and forwarded to a designated genetic testing laboratory for extraction of nucleic acid and molecular analysis for a wide range of molecular faults using a Next Generation Sequencing Panel. They may also have a 5-10ml blood sample taken for extraction of germline DNA in order to compare this with the tumour DNA taken from the tissue sample, as well as storage of plasma for future ethically approved research studies.

No biobank will be established and if tissue provided is surplus to nucleic acid extraction requirements it will be returned to the Clinical Hub for destruction or other research purposes in accordance with local Human Tissue Authority (HTA) approvals. Nucleic acid extracted from collected tissue or plasma samples as part of the Programme may be stored at the genetic

testing laboratories and will not be returned to Clinical Hubs/sites. The genetic testing laboratories will act as custodians and make nucleic acid samples available for further appropriate ethically-approved research use.

The results of molecular analysis will be made available to clinical teams involved in patient care as well as being stored for potential, ethically-approved future research use. Information that may be relevant to the patient's clinical care for cancer including their eligibility for the National Lung Matrix Trial will be fed back to the patient's clinical care team for use at their discretion, in consultation with the patient, and where appropriate, the local clinical genetics service.

7.2 National Lung Matrix Trial Screening

Once a potentially eligible patient with advanced or metastatic non-small cell lung cancer (NSCLC) has completed all standard of care therapy that the treating oncologist thinks is appropriate, the Technology Hub report of the SMP2 pre-screening result should be consulted. As a minimum patients must have failed one or more lines of treatment, either due to radiological documentation of disease progression or due to toxicity. Alternatively, patients who refuse any standard of care therapy are eligible to receive National Lung Matrix Trial treatment as first line therapy, providing they explicitly consent to this effect.

If the patient has a potentially actionable genetic change in the scope of the National Lung Matrix Trial, or no actionable genetic change but the patient appears to meet core eligibility criteria for the Trial, please contact the National Lung Matrix Trial Office to begin the Screening Registration Process (see Section 7.3 below) and to reserve a slot in the appropriate cohort. Feeder sites may also initiate the Screening Registration Process, but in consultation with their paired National Lung Matrix Trial Participating Site (Clinical Hub) so that a patient can be referred to the Clinical Hub when they are ready to be considered for consent to the relevant arm of the trial.

7.2.1 Patients who require a repeat biopsy

Patients who meet any of the following criteria will require a mandatory repeat biopsy for molecular testing for consideration for entry into the National Lung Matrix Trial:

- There was not enough tissue to perform a test.
- The diagnostic sample did not result in a successful test result (i.e. no actionable mutation detected and <90% successful gene test results).
- The patient was previously treated with a targeted therapy in the context of the National Lung Matrix Trial (i.e. the patient's tumour is EGFR+ or ALK+, has received EGFR or ALK targeted agent therapy since diagnostic biopsy).
- Clinician decision (e.g. significant time lapse since previous collection of biopsy).

Consent for these mandatory repeat biopsies for molecular testing will be obtained under the National Lung Matrix Trial, using the National Lung Matrix Trial Molecular Testing Biopsy Patient Information Sheet and Informed Consent Form. Feeder Sites that send SMP2 samples to their paired Clinical Hub will be able to perform mandatory repeat biopsies for molecular testing locally under the consent of the National Lung Matrix Trial, following the same process as SMP2 pre-screening samples. Note: Adverse events believed to possibly, probably or definitely relate to the biopsy procedure should be collected on the Biopsy Collection Adverse Event Form (see Section 10: Adverse Event Reporting for further details).

7.3 Screening Registration Process

Prior to consenting patients to a repeat biopsy procedure or consent to one of the trial arms, the Principal Investigator (or their designee) for each Feeder Site or Participating Site, should

have returned all required documentation (e.g. R&D approval) to the National Lung Matrix Trial Office, and the site personnel involved with the trial must have received appropriate training (site initiation) from the National Lung Matrix Trial Office.

All patients being considered for trial entry must first have a slot reserved with the National Lung Matrix Trial Office as soon as possible. To do so, a Screening Registration Form must be completed and scanned and emailed (lungmatrix@trials.bham.ac.uk) to the National Lung Matrix Trial Office. The Screening Registration Form should be sent to the National Lung Matrix Trial Office together with the patient's SMP2 pre-screening Technology Hub report, Histology report confirming NSCLC and evidence of locally obtained genetic result where applicable, which should be anonymised and labelled with the patient's Screening Number (SNO).

The SNO should be obtained from a pre-defined list of Screening Numbers on the Patient Screening Registration Log which can be found in section 2 of the Investigator Site File/Feeder Site File. Details of this process should be recorded on the Patient Screening Registration Log.

Please note: performing the Screening Registration Process is **not** confirmation of slot allocation within a trial cohort. Please see further details on slot allocation in Sections 7.3.1 and 7.3.2 below.

The original versions of the Screening Registration Forms that have been emailed to the National Lung Matrix Trial Office should then be sent by post to the National Lung Matrix Trial Office, with a copy kept in the Investigator Site File.

7.3.1 Patients who do not require a repeat biopsy

The National Lung Matrix Trial Office will review the Technology Hub report, Histology report and evidence of locally obtained genetic result where applicable, and aim to confirm within 3 working days a slot allocation (where appropriate) for the relevant cohort by return of a Cohort Allocation Form. This Form will also be used to communicate to Sites where a slot may not be currently available for a given patient on the relevant cohort, and whether they have been placed on a waiting list should a slot become available in the future. Participating Sites can then consent patients appropriately for the relevant treatment arm, and Feeder Sites will be expected to refer patients to Participating Sites during this process.

7.3.2 Patients who require a repeat biopsy

The Participating or Feeder Sites can consent patients to the mandatory repeat biopsy for molecular testing. Please ensure that it is clear on documentation sent to the Technology Hub that the sample is a repeat biopsy and should therefore be prioritised for analysis. Once available an anonymised copy of the repeat biopsy Technology Hub report labelled with the SNO should be emailed to the National Lung Matrix Trial Office with the Screening Registration Form. The National Lung Matrix Trial Office will review the Technology Hub report and aim to confirm within 3 working days a slot allocation for the relevant cohort by return of a Cohort Allocation Form. This Form will also be used to communicate to Sites where a slot may not be currently available for a given patient on the relevant cohort, and whether they have been placed on a waiting list should a slot become available in the future. Participating Sites can then consent patients appropriately for the relevant treatment arm, and Feeder Sites will be expected to refer patients to Participating Sites.

Figure 9: Screening registration process flow chart

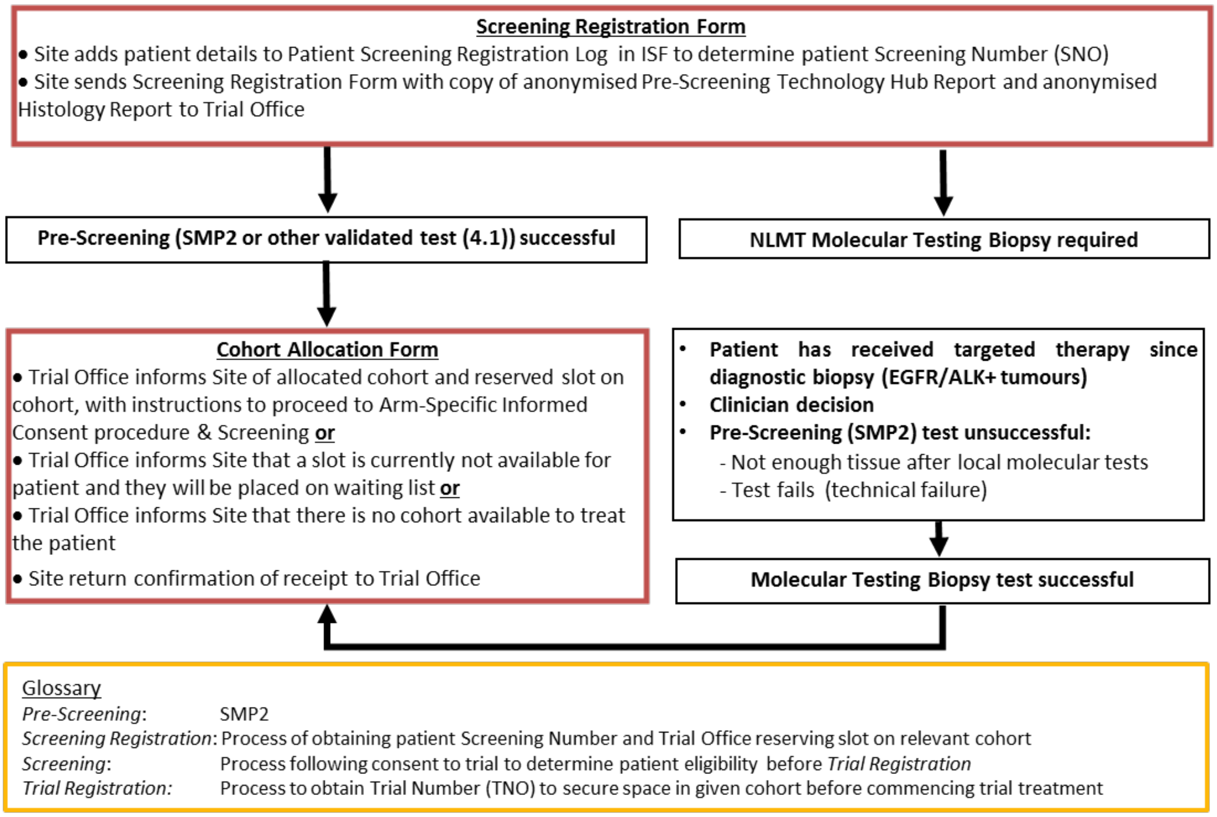

7.4 Informed Consent

7.4.1 Informed Consent for Mandatory Repeat Biopsies

It is the responsibility of the Principal Investigator or designated Sub-Investigator specified on the Site Signature and Delegation Log to obtain written informed consent for each patient prior to performing a mandatory repeat biopsy for molecular testing. A specific Patient Information Sheet – Molecular Testing Biopsy Patient Information Sheet - is provided to facilitate this process. Investigators must ensure that they adequately explain to the patient the aim of the procedure, anticipated benefits and potential hazards of having this procedure. The local Investigator should also stress that the patient is completely free to refuse to take part or withdraw consent for this procedure. The patient should be given ample time (e.g. 24 hours) to read the Patient Information Sheet and to discuss their participation with others outside of the site research team. The patient must be given the opportunity to ask questions which should be answered to their satisfaction. The right of the patient to refuse to undergo a repeat biopsy without giving a reason must be respected.

If the patient expresses an interest in undergoing a repeat biopsy, they should be asked to sign and date the latest approved version of the Molecular Testing Biopsy Informed Consent Form. The Investigator or designate must then sign and date the form. A copy of the Informed Consent Form should be given to the patient, a copy should be filed in the hospital notes, and the original placed in the Investigator Site File (ISF). In addition, if the patient has given explicit consent, a copy of the signed Informed Consent Form must be sent in the post to the National Lung Matrix Trial Office for review if the patient is later registered to the trial.

Details of the informed consent discussions should be recorded in the patient's medical notes, this should include date of, and information regarding, the initial discussion, the date consent

was given, with the name of the trial and the version number of the Molecular Testing Biopsy Patient Information Sheet and Informed Consent Form used.

Electronic copies of the Molecular Testing Biopsy Patient Information Sheet and Informed Consent Form are available from the National Lung Matrix Trial Office or on the National Lung Matrix Trial website and should be printed or photocopied onto the headed paper of the local institution.

Details of all patients approached about the trial should be recorded on the Patient Screening/Enrolment Log.

A copy of the Biopsy for Molecular Testing Informed Consent Form should be sent to the Trial Office following trial registration, for in-house review.

7.4.2 Informed Consent for Trial Participation

It is the responsibility of the Principal Investigator or designated Sub-Investigator specified on the Site Signature and Delegation Log to obtain written informed consent for each patient prior to performing any trial related procedure. Arm-specific Patient Information Sheets are provided to facilitate this process. Local Investigators must ensure that they adequately explain the aim, trial treatment, anticipated benefits and potential hazards of taking part in the trial to the patient. The local Investigator should also stress that the patient is completely free to refuse to take part or withdraw from the trial at any time. The patient should be given ample time (e.g. 24 hours) to read the Patient Information Sheet and to discuss their participation with others outside of the site research team. The patient must be given an opportunity to ask questions which should be answered to their satisfaction. The right of the patient to refuse to participate in the trial without giving a reason must be respected.

If the patient expresses an interest in participating in the trial they should be asked to sign and date the latest version of the specific Informed Consent Form. In the event that an abnormality that might affect other family members is uncovered during genetic testing for the trial, the patient will be asked if they would like their doctor to be informed so that they can be referred to a genetic counsellor if appropriate. This consent is optional. Please see Appendix 5: Incidental Genetic findings policy for the National Lung Matrix Trial Incidental Findings Policy. The local Investigator or designate must then sign and date the form.

A copy of the Informed Consent Form should be given to the patient, a copy should be filed in the hospital notes, and the original placed in the Investigator Site File (ISF). Once the patient is entered into the trial the patient's trial number should be entered on the Informed Consent Form maintained in the ISF. In addition, if the patient has given explicit consent by signing the copy of the Informed Consent Form a copy of the signed Informed Consent Form must be sent in the post to the National Lung Matrix Trial Office for review. Details of the informed consent discussions should be recorded in the patient's medical notes, this should include date of, and information regarding, the initial discussion, the date consent was given, with the name of the trial and the version number of the Patient Information Sheet and Informed Consent Form. Throughout the trial the patient should have the opportunity to ask questions about the trial and any new information that may be relevant to the patient's continued participation should be shared with them in a timely manner. On occasion it may be necessary to re-consent the patient in which case the process above should be followed and the patient's right to withdraw from the trial respected.

Electronic copies of the Patient Information Sheet and Informed Consent Form are available from the National Lung Matrix Trial Office or website and should be printed or photocopied onto the headed paper of the local institution.

Details of all patients approached about the trial should be recorded on the Patient Screening/Enrolment Log and with the patient's prior consent their General Practitioner (GP) should also be informed that they are taking part in the trial. A GP Letter is provided electronically for this purpose.

Figure 10: Informed Consent Process Flow Chart

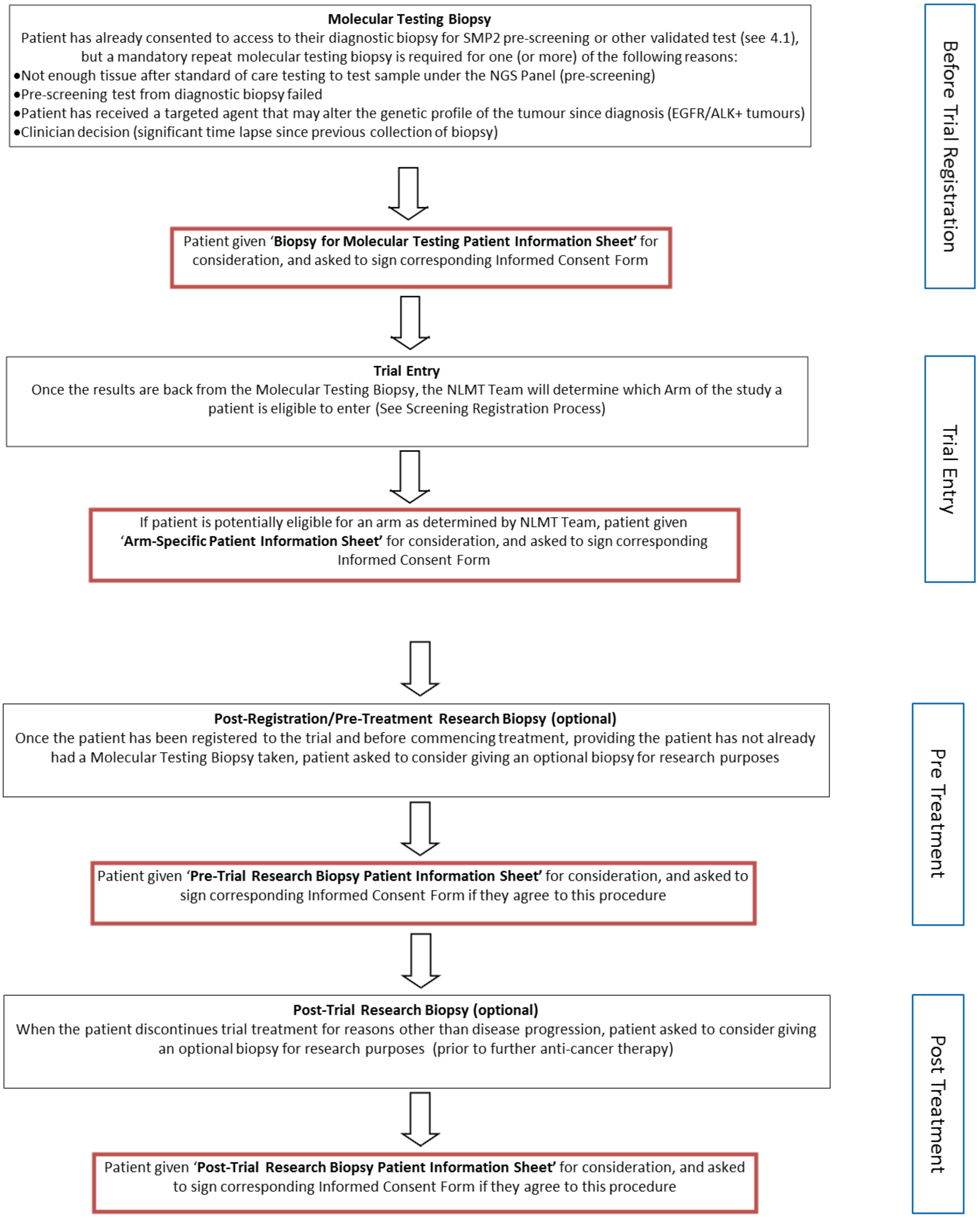

8 TRIAL ENTRY

Prior to trial entry, patients must have a slot reserved on the relevant cohort within the trial, and the National Lung Matrix Trial Office must have determined which cohort the patient is eligible to enter (see Screening Registration section 7.3 above).

The patient's eligibility will be confirmed at registration. Participating Sites must send a copy of the completed Core and Arm-Specific Eligibility Checklists to the National Lung Matrix Trial Office by email. Sites must then telephone the registration line with the details required on the Registration Form, and the patient will be registered over the phone and allocated a Trial Number (TNO). An email to key site personnel (including the Principal Investigator, the person performing the registration and responsible pharmacist) will then be sent confirming the details of registration, and containing a link to the National Lung Matrix Trial electronic Remote Data Capture (eRDC) system, from which site users with the correct permissions and a username may log in and download a trial entry confirmation report. Trial drug prescriptions must include the patient's Trial Number and dose. The registration line will be open during office hours (09:00 – 17:00, Monday-Friday).

Registration line: **0121 414 7611 or 0121 414 6788** (09:00 - 17:00, Monday to Friday).

Registration email: lungmatrix@trials.bham.ac.uk

The original Eligibility Checklists should be returned in the post to the National Lung Matrix Trial Office with a copy retained in the ISF.

9 TREATMENT DETAILS

All AstraZeneca trial investigational medicinal products (IMP) will be supplied, packaged and delivered free of charge to the Pharmacy Hub; Fisher Clinical Services. Pfizer products will be ordered from Pfizer via the Trial Office free of charge, and will be labelled and distributed by Fisher Clinical Services. Please see the Pharmacy Manual for further details of distribution of IMP from the Pharmacy Hub to clinical trial sites.

Specific treatment details are outlined for each arm of the trial and in the Pharmacy Manual.

ARM A: AZD4547 – FGFR Inhibitor *(Closed to Recruitment)*
ARM B: Vistusertib – MTORC1/2 Inhibitor *(Closed to Recruitment)*ARM C: Palbociclib – CDK4/6 Inhibitor
ARM D: Crizotinib – ALK/MET/ROS1 Inhibitor
ARM E: Selumetinib – MEK Inhibitor in combination with Docetaxel
ARM F: AZD5363 - AKT inhibitor *(Closed to recruitment)*
ARM G: Osimertinib – EGFRM+ AND T790M+ Inhibitor *(Closed to recruitment)*
ARM H: Sitravatinib (MGCD516) - VEGFR Inhibitor *(Closed to Recruitment)*
ARM J & Cohort NAJ: AZD6738 (ATR Inhibitor) & Durvalumab (Anti-PDL1)
COHORT NA1: Durvalumab – Anti-PDL1 *(Closed to Recruitment)*

9.1 Treatment Compliance

Treatment compliance will be strictly monitored. For orally administered drugs (Arms A-J) patients will be asked to complete and return patient diary cards every cycle. In addition, patients will be asked to return any unused tablets or capsules at the end of each cycle for reconciliation. Drug returns will be disposed of by Pharmacy according to local hospital

practice, and documented on the Patient Specific Drug Accountability Log. Further information can be found in the Pharmacy Manual (supplied separately).

9.2 Concomitant Medication

Throughout the study any concomitant medication or therapy deemed necessary to provide adequate supportive care may be prescribed and should be recorded within the Case Report form (CRF) on the electronic Remote Data Capture (eRDC) system. The indication for the treatment should be recorded at the same time. Where concomitant medications are prohibited for a specific agent these are detailed in the relevant arm supplement and Investigator Brochure or Summary of Product Characteristics (SPC). A Prohibited Medications List combining records for each IMP can be found in Section 4 of the Investigator Site File.

Some prohibited concomitant medication may be permitted, only if this is considered safe with very close monitoring, patients have given their consent and it is in the best interest of patients. However, this will only be allowed following consultation with the trials office and CI.

9.3 Patient Withdrawal

The local Investigator will make every reasonable effort to keep each patient on treatment. However, if the local Investigator removes a patient from the study treatment or if (s)he declines further participation final assessments will be performed, if possible. All the results of the evaluations and observations, together with a description of the reasons for study withdrawal, must be recorded in the CRF. For patients withdrawing from trial treatment for reasons other than withdrawal of consent or due to disease progression then continue with RECIST scans until documented progression or the patient starts a new anti-cancer therapy as per protocol.

In the event of a patient’s decision to withdraw from the trial, the local Investigator must ascertain from which aspects of the trial the patient wishes to withdraw, and record the details on the appropriate CRF. All patients will continue to be followed-up (if they agree), and all information and tissue samples collected up until the point of retraction, will be retained and analysed.

Following assessment by the Principal Investigator and discussion with the National Lung Matrix Trial Office, patients who meet RECIST criteria for progressive disease (PD) may be continued on trial treatment if the treatment is tolerable and believed to be of clinical benefit.

Patients who are removed from treatment due to adverse events (clinical or laboratory) will be treated and followed-up according to accepted medical practice. All pertinent information concerning the outcome of such treatment must be recorded in the CRF.

The following are justifiable reasons for the local Investigator to withdraw a patient from study:

- Unacceptable toxicity.
- Unforeseen events: any event which in the judgement of the local Investigator makes further treatment inadvisable.
- SAE requiring discontinuation of treatment.
- Withdrawal of consent.
- Serious violation of the study protocol (including persistent patient attendance failure and persistent non-compliance).
- Withdrawal by the local Investigator for clinical reasons not related to the study drug treatment.

9.4 COVID-19 Pandemic

The National Lung Matrix Trial remains open during the Covid-19 pandemic, although individual trusts may make the decision to close. The Covid-19 pandemic may have a significant impact on patient recruitment, trial visits and treatment, including standard of care (SOC). Patient safety is paramount and Investigators are advised to follow local practices and document any deviations from the protocol on a deviation form. During these exceptional circumstances, reasonable deviations would not trigger additional monitoring visits. Due to Covid-19 associated clinic visit restrictions, sites are permitted to conduct telephone and virtual consultations instead of face-to-face consultations. Blood tests may be performed locally at GP surgeries or community-based clinics, if patients are unable to have these required safety assessments at their hospitals. We appreciate that it may not always be possible to obtain these results for the CRF within the normal expected timeframe. Clincial Trial Pharmacies are permitted to prescribe IMP for more than 1 cycle of treatment due to a reduction in patient clinic visits. In accordance with local practice, sites are permitted to post ambient trial medication, if safe delivery can be confirmed. Refrigerated IMP can be couriered to patients if required. Please note that the trial office cannot reimburse sites for the shipment of IMP direct to patients. We ask all sites posting IMP to inform the NLMT Trial Office.

Scans that are missed due to Covid-19 restrictions and reduced scanning capacityshould be recorded on a deviation form. Future scheduled scans, as with all trial procedures should continue per protocol as soon as possible.

10 TRANSLATIONAL RESEARCH

Longitudinal circulating tumour DNA (ctDNA) and tumour tissue samples are key translational elements in understanding mechanisms of resistance in tumour cells and identifying predictive biomarkers for drug activity.

Repeat molecular testing biopsies will be collected in certain circumstances, for the purposes of re-testing on the SMP2 panel (see Section 2: Trial Biopsies and Section 7.2.1: Patients who require a repeat biopsy).

In the non-actionable cohort NA1 *(Closed to recruitment*), peripheral blood mononuclear cells (PBMC) samples will be collected to investigate immune responses. In this cohort it is also important to establish the PDL1 status of the tumour. Therefore all patients being considered for this cohort will have a mandatory repeat molecular testing biopsy, and sufficient tissue from this (>100 tumour cells) should be sent for PDL1 staining.

In Arm H only, pharmacokinetic (PK) samples are to be collected from patients: The PK exposure from this trial may be used in the development of population PK models for sitravatinib. Plasma concentrations will be listed by subject for the PK population. Summary statistics of sitravatinib concentrations will be reported by day and cycle. Only samples with acceptable PK will be included in the summary statistics and a listing of individual data points or subjects excluded from the analysis will be presented.

Smoking status data will be collected from patients on all Arms. Patients will be asked to complete a Smoking Questionnaire at certain timepoints, and carbon monoxide measurements will also be taken at these visits, using a MicroCO monitor provided by the National Lung Matrix Trial Office at site initiation.

10.1 Germline Blood Sample

A whole blood germline DNA sample is to be collected pre-dose on cycle 1 day 1. If the sample is not collected at this timepoint, it should be collected at the next visit. All instructions on collection, handling, processing, shipment and storage of germline blood samples are documented in the current version of the National Lung Matrix Trial Laboratory Manual. These samples will be stored in the Human Biomaterials Resource Centre (HBRC), University of Birmingham and shipped to Inivata and the University of Cambridge for whole exome sequencing.

10.2 ctDNA Samples

ctDNA samples (9-10 ml of blood plasma) are to be collected pre-dose on cycle 1 day 1, then every 8 or 9 weeks depending on cycle length and at discontinuation (Please refer to Schedule of Assessments for each Arm).

ctDNA samples will also be collected in follow up for patients who discontinue treatment for reasons other than Progressive Disease (e.g. toxicity). These samples should be performed at the same visit as follow up CT or MRI scans until disease progression or the patient starts a new anti-cancer therapy (unless the patient withdraws consent to do so). See arm specific Schedule of Events for frequency and timing of sample collection in follow up.

All instructions on collection, handling, processing, shipment and storage of ctDNA samples are documented in the current version of the National Lung Matrix Trial Laboratory Manual. These samples will be stored at the HBRC, University of Birmingham and shipped to Inivata and the University of Cambridge for whole exome sequencing at a later date.

10.3 PDL1 staining

Patients considered for Arm NA Cohort NA1 will be approached to consent for a mandatory repeat biopsy for molecular testing. All instructions on collection, handling, processing, shipment and storage of molecular testing biopsies are documented in the current version of the National Lung Matrix Trial Laboratory Manual. These samples will first be sent for repeat SMP2 testing on the Next Generation Sequencing (NGS) panel, before the remaining tissue (>100 tumour cells) is shipped to the HBRC, University of Birmingham for storage, before being stained for the presence of PDL1.

10.4 PBMC samples

PBMC samples are to be collected from patients registered to Cohort NA1 pre-dose week 0 day 1, week 2, week 6 and at discontinuation. Samples can be taken up to 1-2 days earlier than the actual visit date (please refer to Arm NA Cohort NA1 Schedule of Assessments).

These samples will be collected and shipped **Monday-Thursday only** using a Royal Mail Safebox provided by the Trials Office to the DeSanto/Mussai/Middleton Laboratory at the University of Birmingham for analysis. It is important that the Trials Office and the DeSanto/Mussai/Middleton are given **as much notice as possible** via email that these samples are to be posted as they will be processed immediately on arrival at the laboratory. All instructions on collection, handling, processing, shipment and storage of PBMC samples are documented in the current version of the National Lung Matrix Trial Laboratory Manual.

10.5 Pharmacokinetic samples

Pharmacokinetic samples are to be collected from patients registered to Arm H at the following timepoints:

- Cycle 1 day 1: < 30 minutes pre-dose & 4 hours post-dose

- Cycle 1 day 10: < 30 minutes pre-dose
- Cycle 2 day 1: < 30 minutes pre-dose
- An additional ad hoc sample should be collected within 7 days of a Serious Adverse Reaction or a dose modification

All instructions on collection, handling, processing, shipment and storage of pharmacokinetic samples are documented in the current version of the National Lung Matrix Trial Laboratory Manual.

10.6 Smoking status assessments

Smoking status data (including smoking history, prevalence of continued smoking during treatment and use of smoking cessation aids) will be collected from patients on all Arms. Patients will be asked to complete a Smoking Questionnaire at certain timepoints; pre-dose Cycle 1 Day 1 (or Week 0 Day 1) then every 8 or 9 weeks whilst on treatment depending on cycle length and at discontinuation (Please refer to Schedule of Assessments for each Arm).

The level of carbon monoxide in the patient's exhaled breath will also be measured at these visits, using a MicroCO monitor. Instructions for the use and calibration of MicroCO monitors are documented in the current version of the National Lung Matrix Trial Laboratory Manual. The measurement in parts per million concentration of carbon monoxide should then be documented on the first page of the Smoking Questionnaire.

This sub-study aims to investigate whether quitting smoking after diagnosis in advanced stage patients is associated with improved cancer outcomes using prospectively collected, biochemically validated measures of smoking exposure. Both the MicroCO monitor and calibration kit will be provided by the National Lung Matrix Trial Office prior to site activation, and should be returned following site closure.

11 ADVERSE EVENT REPORTING

The collection and reporting of Adverse Events (AEs) will be in accordance with the Medicines for Human Use Clinical Trials Regulations 2004 and its subsequent amendments. Definitions of different types of AE are listed in Appendix 6: Definition of Adverse Events. The local Investigator should assess the seriousness and causality (relatedness) of all AEs experienced by the patient (this should be documented in the source data) with reference to the IB and/or SPC.

Specific reporting requirements for the pharma partners are listed below.

11.1 Reporting Requirements

11.1.1 Adverse Events

All medical occurrences which meet the definition of an AE (see Appendix 6: Definition of Adverse Events for definition) should be reported. Please note this includes abnormal laboratory findings that are deemed clinically significant by the local Investigator (abnormal laboratory findings that are not clinically significant are not AEs) or any pre-existing AEs that worsen by at least 1 CTCAE grade from baseline. If an AE changes grade, this should be reported as an individual AE.

11.1.2 Serious Adverse Events

Local Investigators should report AEs that meet the definition of an SAE as per the SAE Form Completion Guidelines (see Appendix 6: Definition of Adverse Events for definition).

11.1.2.1 Events that do not require reporting on a Serious Adverse Event Form

The following events should not be reported on an SAE Form:

- Progression or death as a result of the patient's cancer, as this information is captured elsewhere within the eRDC.
- Hospitalisations for:
    - Protocol defined treatment.
    - Pre-planned elective procedures unless the condition worsens.
    - Treatment for progression of the patient's cancer.

11.1.2.2 Hy's Law cases

Abnormal values in AST and/or ALT concurrent with abnormal elevations in total bilirubin that meet the criteria outlined below in the absence of other causes of liver injury are considered possible cases of drug-induced liver injury (Hy's Law cases) and should always be considered important medical events. Cases where a patient shows AST or ALT ≥3 x ULN together with total bilirubin ≥2 x ULN may need to be reported as SAEs. These cases should be reported as SAEs if after evaluation they meet the criteria for a Hy's Law case (FDA Guidance 2009) or if any of the individual liver test parameters fulfil any of the SAE criteria.
Please refer to ARM B: Vistusertib – MTORC1/2 Inhibitor, ARM D: Crizotinib – ALK/MET/ROS1 Inhibitor, COHORT NA1 and Appendix 12: Actions required in case of increases in liver biochemistry and evaluation of Hy's Law.

11.1.2.3 Potential Sight-Threatening and Severe Vision Loss Adverse Events (Arm D – Crizotinib)

For all Arm D patients, the occurrence of a Grade ≥2 Potential Sight Threatening (PST) (except for Visual field defect, for which only Grade ≥3 events are required) or Severe Vision Loss (SVL) event, as outlined below, should be reported as SAEs.

The following MEDdra preferred terms are considered indicative of a PST or SVL event: amaurosis, amaurosis fugax, blindness, blindness cortical, blindness day, blindness night, blindness transient, blindness unilateral, hemianopia, hemianopia heteronymous, hemianopia homonymous, optic atrophy, optic ischaemic neuropathy, optic nerve disorder, optic neuropathy, quadranopia, retinopathy, sudden visual (or vision) loss, toxic optic neuropathy, tunnel vision, visual cortex atrophy, visual field defect, visual pathway disorder, retinal oedema, retinal detachment, maculopathy, iritis, uveitis, visual field test abnormal.

After you report the SAE to the National Lung Matrix Trial Office, a PST/SVL Data Capture Aid (DCA) which contains relevant clinical and diagnostic questions, including information from any ophthalmic examinations, will also be requested. The National Lung Matrix Trial Office will contact you on behalf of Pfizer, to complete the form.

11.1.2.4 Monitoring pregnancies for potential Serious Adverse Events

It is important to monitor the outcome of pregnancies of patients in order to provide SAE data on congenital anomalies or birth defects.

In the event that a patient or their partner becomes pregnant following consent to a trial arm, please complete a Pregnancy Notification Form (providing the patient's details) and return to the National Lung Matrix Trial Office as soon as possible. If it is the patient who is pregnant, provide outcome data on a follow-up Pregnancy Notification Form. Where the patient's partner

is pregnant consent must first be obtained and the patient should be given a Release of Medical Information Form to give to their partner. If the partner is happy to provide information on the outcome of their pregnancy they should sign the Release of Medical Information Form. Once consent has been obtained, provide details of the outcome of the pregnancy on a follow-up Pregnancy Notification Form. If appropriate also complete an SAE Form as detailed below.

11.1.2.5 Reporting period for patients who do not have a pre-trial mandatory repeat biopsy

Details of all AEs will be documented and reported from the date of Informed Consent for trial treatment until 28 days after the administration of the last treatment. If the patient fails screening, AEs are not required to be documented or reported from the point of recognition as a screen failure. SAEs that are judged to be at least possibly related to IMP must still be reported in an expedited manner irrespective of how long after IMP administration the reaction occurred.

11.1.2.6 Reporting period for patients who have a pre-trial mandatory repeat biopsy

Adverse Events relating to the pre-trial mandatory repeat biopsy procedure must be collected on the Biopsy Collection AE Form until 28 days post biopsy procedure, or until the date of informed consent to a trial arm (whichever comes first). Biopsy related AEs which meet the definition of an SAE should be reported on an SAE Form. Biopsy related AEs ongoing at the time of informed consent to a trial arm should be recorded on the generic Adverse Event Form and resolution information supplied. Feeder Sites will also be expected to collect biopsy related AEs and the details of ongoing events should be supplied to the Clinical Hub/Participating Site when the patient is referred for trial arm consent.

11.2 Reporting Procedure

11.2.1 Site

11.2.1.1 Adverse Events

AEs should be reported on a Biopsy Collection AE Form or a generic AE form (and where applicable on an SAE Form - see definitions in 10.1.1 and 10.1.2 above). Where applicable, Feeder Sites will also be expected to collect AEs and report SAEs for patients following consent to mandatory repeat biopsies. For patients later referred to a Clinical Hub/Participating Site, centres should be made aware of any ongoing adverse events at the time of referral. An AE Form should be completed at each visit and returned to the National Lung Matrix Trial Office. See CRF Completion Guidelines for instructions.

AEs will be reviewed using the Common Terminology Criteria for Adverse Events (CTCAE), version 4.0 (see Appendix 3: Common Toxicity Criteria Gradings). Any AEs experienced by the patient but not included in the CTCAE should be graded by a local Investigator and recorded on the AE Form using a scale of (1) mild, (2) moderate or (3) severe. For each sign/symptom, any changes in grade should be documented.

11.2.1.2 Serious Adverse Events

For more detailed instructions on SAE reporting refer to the SAE Form Completion Guidelines contained in the Investigator Site File (ISF).

AEs defined as serious and which require reporting as an SAE should be reported on an SAE Form. When completing the form, the local Investigator will be asked to define the causality and the severity of the AE which should be documented using the CTCAE version 4.0.

On becoming aware that a patient has experienced an SAE, the local Investigator (or delegate) must complete, date and sign an SAE Form. The form should be emailed to the National Lung Matrix Trial Office using the email address below as soon as possible and no later than 24 hours after first becoming aware of the event:

To report an SAE, email the SAE Form to: reg@trials.bham.ac.uk
Include *"National Lung Matrix Trial SAE"* in the subject line

A separate email should also be sent to the Lung Matrix mailbox informing the Trials Office that an SAE has been reported: lungmatrix@trials.bham.ac.uk. Please refer to the SAE Completion Guidelines for further instructions.

On receipt of SAE Forms for events experienced following registration to the trial, the National Lung Matrix Trial Office will allocate each SAE a unique reference number. This number will be sent by email to the site confirming receipt. If confirmation of receipt is not received within 1 working day please contact the National Lung Matrix Trial Office. The SAE reference number should be quoted on all further correspondence and follow-up reports regarding the SAE. Confirmation of receipt provided by the National Lung Matrix Trial Office should be filed with the SAE Form in the ISF.

For SAE Forms completed by someone other than a delegated Investigator of consultant level, a local Investigator will be required to countersign the original SAE Form to confirm agreement with the causality and severity assessments. The original SAE Form should then be filed in the ISF. Local Investigators should also report SAEs to their own Trust in accordance with local practice.

11.2.1.3 Provision of follow-up information

Patients should be followed up until resolution or stabilisation of the serious adverse event. Follow-up information should be provided on a new SAE Form (refer to the SAE Form Completion Guidelines for further information).

11.2.2 National Lung Matrix Trial Office

On receipt of an SAE Form seriousness and causality will be determined independently by a Clinical Coordinator. An SAE judged by the local Investigator or Clinical Coordinator to have a reasonable causal relationship with the trial medication will be regarded as a Serious Adverse Reaction (SAR). The Clinical Coordinator will also assess all SARs for expectedness. If the event meets the definition of a SAR that is unexpected (i.e. is not defined in the relevant IB) it will be classified as a Suspected Unexpected Serious Adverse Reaction (SUSAR).

11.2.3 Reporting to the Competent Authority and main Research Ethics Committee

11.2.3.1 Suspected Unexpected Serious Adverse Reactions

The National Lung Matrix Trial Office will report a minimal data set of all individual events categorised as a fatal or life threatening SUSAR to the Medicines and Healthcare products Regulatory Agency (MHRA) and main Research Ethics Committee (REC) within 7 days. Detailed follow-up information will be provided within an additional 8 days.
All other events categorised as SUSARs will be reported within 15 days.

11.2.3.2 Serious Adverse Reactions

The National Lung Matrix Trial Office will report details of all SARs (including SUSARs) to the MHRA and main REC annually from the date of the Clinical Trial Authorisation, in the form of a Developmental Safety Update Report.

11.2.3.3 Adverse Events

Details of all AEs will be reported to the MHRA on request.

11.2.3.4 Other safety issues identified during the course of the trial

The MHRA and main REC will be notified immediately if a significant safety issue is identified during the course of the trial.

11.2.4 Investigators

Details of all SUSARs and any other safety issue which arises during the course of the trial will be reported to Principal Investigators. A copy of any such correspondence should be filed in the ISF.

11.2.5 Trial Management Group (TMG)

The Trial Management Group will be given the opportunity to review all SAEs.

11.2.6 Trial Steering Committee (TSC)

The Trial Steering Committee will review all SAEs.

11.2.7 Manufacturer of Investigational Medicinal Product

All SAEs will be reported to the manufacturer of the Investigational Medicinal Product within 24 hours of first awareness of the event of the National Lung Matrix Trial Office during the reporting period.

12 DATA HANDLING AND RECORD KEEPING

12.1 Data Collection

12.1.1 Case Report Form

An eRDC system will be used for this trial and paper CRFs will also be available as a backup. The local Investigator and site staff will ensure all data from subject visits are promptly entered into the eRDC in accordance with the study specific eRDC User Manual and CRF Completion Guidelines. The local Investigator must approve the eRDC within the electronic system to verify the integrity of the data recorded.

The CRF must be completed by the local Investigator or an authorised member of the site research team (as delegated on the Site Signature and Delegation Log) within the timeframe specified by the National Lung Matrix Trial Office. There are exceptions to this where several forms will require additional signature/approval by a local Investigator to assert that these have been completed correctly (see CRF Completion Guidelines for details). One particular exception is the SAE Form which must be co-signed by the local Investigator as well as the site staff who completed the form initially (this must be done as soon as possible but can be done after the form has already been emailed to the Trial Office so as not to delay initial reporting). See Adverse Event Reporting Section 10 for further details.

Data reported on each form should be consistent with the source data or the discrepancies should be explained. If information is not known, this must be clearly indicated on the form. All missing and ambiguous data will be queried. All sections are to be completed. In all cases it remains the responsibility of the local Investigator to ensure that the CRF has been completed correctly and that the data are accurate.

The format and questions in the CRF may be amended by the Trial Office, as appropriate, throughout the duration of the trial. Whilst this will not constitute a protocol amendment, sites will be notified of new versions of the form when they are available in the eRDC system, and in the case of the SAE form, new versions of the form must be implemented by participating sites immediately on receipt. Paper CRF copies are available on the National Lung Matrix Trial website.

12.2 Archiving

It is the responsibility of the Principal Investigator to ensure all essential trial documentation and source records (e.g. signed Informed Consent Forms, Investigator Site Files, Pharmacy Files, patients' hospital notes, copies of CRFs etc.) at their site are securely retained for at least 25 years after the end of the trial or following the processing of all biological material collected for research, whichever is the later. Do not destroy any documents without prior approval from the National Lung Matrix Trial Office.

13 QUALITY MANAGEMENT

13.1 Site Set-up and Initiation

All sites will be required to sign a Clinical Study Site Agreement prior to participation. In addition all participating Principal Investigators will be asked to sign the necessary agreements and registration forms; and supply a current curriculum vitae (CV) to the National Lung Matrix Trial Office. All members of the site research team will also be required to complete registration forms and sign the Site Signature and Delegation Log, which should be returned to the National Lung Matrix Trial Office. Prior to commencing recruitment all sites will undergo a process of initiation. Key members of the site research team will be required to attend either a meeting or a teleconference covering aspects of the trial design, protocol procedures, AE reporting, collection and reporting of data and record keeping. Sites will be provided with an Investigator Site File and a Pharmacy File containing essential documentation, instructions, and other documentation required for the conduct of the trial. Feeder Sites will be supplied with a Feeder Site File for this purpose. The National Lung Matrix Trial Office must be informed immediately of any change in the site research team.

13.2 Investigator Meetings

Key members of the site research team will be required to attend regular Principal Investigator meetings or teleconferences covering aspects of the trial design and protocol procedures as new targets are incorporated into the National Lung Matrix Trial protocol.

13.3 On-site Monitoring

Monitoring will be carried out as required following a risk assessment and as documented in the CRCTU Quality Management Plan. Additional on-site monitoring visits may be triggered for example by poor CRF return, poor data quality, low SAE reporting rates, excessive number of patient withdrawals or deviations. If a monitoring visit is required the National Lung Matrix Trial Office will contact the site to arrange a date for the proposed visit and will provide the site with written confirmation. Principal Investigators will allow the National Lung Matrix Trial staff access to source documents as requested.

13.4 Central Monitoring

Trials staff will be in regular contact with the site research team to check on progress and address any queries that they may have. Trials staff will check incoming Case Report Forms for compliance with the protocol, data consistency, missing data and timing. Sites will be sent electronic Data Clarification Forms via the eRDC requesting missing data or clarification of inconsistencies or discrepancies.
Where a patient has given explicit consent sites are requested to send in copies of signed Informed Consent Forms for in-house review.

Sites may be suspended from further recruitment in the event of serious and persistent non-compliance with the protocol and/or the Good Clinical Practice (GCP), and/or poor recruitment. Any major problems identified during monitoring may be reported to the Trial Management Group (TMG) and the relevant regulatory bodies. This includes reporting serious breaches of GCP and/or the trial protocol to the main Research Ethics Committee (REC) and the Medicines and Healthcare products Regulatory Agency (MHRA).

13.5 Audit and Inspection

The Principal Investigator will permit trial-related monitoring, audits, ethical review, and regulatory inspection(s) at their site, providing direct access to source data/documents. Sites are also requested to notify the National Lung Matrix Trial Office of any MHRA inspections.

13.6 Notification of Serious Breaches

In accordance with Regulation 29A of the Medicines for Human Use (Clinical Trials) Regulations 2004 and its amendments the Sponsor of the trial is responsible for notifying the licensing authority in writing of any serious breach of:

a) The conditions and principles of GCP in connection with that trial or;

b) The protocol relating to that trial, within 7 days of becoming aware of that breach.

For the purposes of this regulation, a “serious breach” is a breach which is likely to effect to a significant degree:

c) The safety or physical or mental integrity of the subjects of the trial; or

d) The scientific value of the trial.

Sites are therefore requested to notify the National Lung Matrix Trial Office of a suspected trial-related serious breach of GCP and/or the trial protocol. Where the National Lung Matrix Trial Office is investigating whether or not a serious breach has occurred sites are also requested to cooperate with the National Lung Matrix Trial Office in providing sufficient information to report the breach to the MHRA where required and in undertaking any corrective and/or preventive action.

14 END OF TRIAL DEFINITION

The end of trial will be 6 months after the last data capture. This will allow sufficient time for the completion of protocol procedures, data collection and data input. The National Lung Matrix Trial Office will notify the MHRA and main REC that the trial has ended and will provide them with a summary of the clinical trial report within 12 months of the end of trial.

After closure of the trial with the MHRA the Sponsor is no longer required to notify the main REC of changes of Principal Investigator. However, sites should continue to notify the National

Lung Matrix Trial Office of changes in Principal Investigator by completing and returning (where required) an Investigator Registration Form together with a current signed and dated CV.

15 STATISTICAL CONSIDERATIONS

15.1 Statistical Design for Cohorts with Actionable Target (Arms A-J)

The trial design and outcome measures are detailed in Section 5. The aim of the statistical analysis for the cohorts with an actionable target biomarker receiving an experimental targeted drug is to determine whether there is sufficient signal of activity in any drug-(putative)biomarker combination to warrant further investigation. The trial design for each drug-(putative)biomarker cohort is essentially the same but the primary outcome measures representing signal of activity and the values that determine the go/no go decision at interim and final analysis may differ across the different cohorts and these are summarised in Section 15.3 with further detail in each supplement of the protocol as needed.

Due to the complexity of the study, we have chosen an adaptive design with a Bayesian approach to decision-making that better reflects real life evaluation than traditional designs. This adaptive design will provide flexibility to make conclusions from all patients recruited to any drug-(putative)biomarker combination without fixing the exact number which will be important given the multiple biomarkers and some uncertainty regarding prevalence rates. The design will be adaptive to enable early stopping of recruitment and review of molecular eligibility, to any drug-(putative)biomarker combinations that do not show sufficient promise at any interim analysis to warrant continuation.

15.2 Statistical Analysis Plan for Cohorts with Actionable Target (Arms A-J)

The final analysis will generate the posterior probability distribution for the primary outcome measures to represent the signal of activity for each targeted drug in each targeted cohort. From this, relevant probabilities can be established on which a decision for further research (i.e. the go/no go decision) will be based. Of particular interest will be the probability that the true outcome measure summary statistic is greater than some pre-specified clinically relevant value (see Section 15.3). Secondary outcome measures will also contribute to the decision to continue further research. The trial will aim to recruit 30 per protocol patients per drug-(putative)biomarker combination for this final analysis, as justified in Section 15.3, but the flexible design ensures that this analysis will be applicable for any number of patients.

The Bayesian adaptive trial design allows interim analysis at any point in the trial but a formal interim analysis is planned per cohort after 15 per protocol patients have been recruited in each drug-(putative) biomarker cohort and reached the first scan at 6 weeks (8 weeks for Arm J) at which decisions to stop recruitment early to the cohort for futility will be made based on the posterior probability distribution at that time. The decision criteria are specified in Section 15.3 together with justification for the choice of interim sample size of 15 patients. In addition, interim analyses will be used to indicate the need to review molecular eligibility criteria (see separate Statistical Analysis Plan for further details). In general, recruitment will not be suspended whilst interim analyses are being performed and the Trial Steering Committee (TSC) is reviewing the data. Rapid review will be necessary for fast recruiting cohorts to ensure the utility of interim decisions.

The primary analysis for any drug-(putative)biomarker cohort will be based on non-informative priors but, where appropriate, secondary analysis with informative priors may be carried out to incorporate other relevant information external to the data for that specific drug-(putative)biomarker cohort. In addition, and subject to biological plausibility, Bayesian hierarchical modelling will be employed to ‘borrow strength’ of information about the primary outcome measures across different biomarker cohorts within any single drug to provide

secondary information to aid decision-making, particularly in borderline situations. Such secondary analysis will never be used to negate a primary analysis that shows a potentially positive result.

Further details of the planned statistical analysis can be obtained from the separate Statistical Analysis Plan.

15.3 Sample Size Justification for Cohorts with Actionable Targets (Arms A-J)

At the final analysis for each drug-(putative)biomarker cohort, decision-making regarding go/no go for further research will be based on the posterior probability distribution for the primary outcome measures, together with data from secondary outcome measures. In order to determine an appropriate sample size for the trial, a specific decision criterion needs to be chosen in relation to the primary outcomes, that reflect the likely go/no go decision that will be made. The operating characteristics can then be evaluated for a range of sample sizes to determine the appropriate number. The following generic criterion represents the sort of decision that would be made at the final analysis:

If p (true signal for drug i in biomarker group j > s_1 | current observed data and any relevant prior information) > q_1 (i.e. if there is a high chance that the true signal in the targeted group is greater than some clinically relevant threshold value) then this drug-(putative)biomarker combination will be deemed worthy of further investigation. The threshold values of s_1 and q_1 will be specifically chosen for each drug-(putative)biomarker combination (see Table 7).

At the interim analysis for each drug-(putative)biomarker cohort, the posterior probability distribution for the primary outcome measures at that time will be calculated and the following generic decision criteria will be applied to guide whether recruitment should continue in that particular drug-(putative)biomarker combination. Operating characteristics are evaluated for these decision criteria for a range of interim sample sizes to determine the appropriate number.

If p (true signal for drug i in putative biomarker group j < s_0 | current observed data and any relevant prior information) > q_0 (i.e. if there is a high chance that the true signal in the targeted group falls below a clinically relevant threshold value) then this drug-(putative)biomarker combination may be rejected as not worthy of further investigation and recruitment may be stopped. The threshold values of s_0 and q_0 will be specifically chosen for each drug-(putative)biomarker combination (see Table 7 for pre-specified values).

Table 7: Thresholds for decision-making for each drug

	**Arm A: AZD4547** *Closed to recruitment*	**Arm B: Vistusertib** *Closed to recruitment*	**Arm C: Palbociclib**	**Arm D: Crizotinib**	**Arm E: Selumetinib & Docetaxel**	**Arm F: AZD5363** *Closed to recruitment*	**Arm G: Osimertinib** *Closed to recruitment*	**Arm H: Sitravatinib** *Closed to recruitment*	**Arm J: AZD6738 & Durvalumab**
**Primary outcome variable/ s**	OR & DCB	OR & DCB	PFS	OR & DCB	OR & DCB	OR & DCB	OR	OR & DCB	OR & DCB
**s_1**	Rate = 30%	Rate = 30%	Median = 3 months	Rate = 30%	Rate = 40%	Rate = 30%	Rate = 30%	Rate = 30%	Rate = 30%

	Arm A: AZD4547 *Closed to recruitment*	Arm B: Vistusertib *Closed to recruitment*	Arm C: Palbociclib	Arm D: Crizotinib	Arm E: Selumetinib & Docetaxel	Arm F: AZD5363 *Closed to recruitment*	Arm G: Osimertinib *Closed to recruitment*	Arm H: Sitravatinib *Closed to recruitment*	Arm J: AZD6738 & Durvalumab
q_1 (see Note1)	0.5	0.5	0.5	0.5	0.5	0.5	0.5	0.5	0.5
s_0 (see Note 2)	30%	30%	Median = 3 months	30%	40%	30%	30%	30%	30%
q_0 (see Note3)	0.9	0.9	0.8	0.9	0.9	0.9	0.9	0.9	0.9

Note 1: In all cases 0.5 has been selected for this parameter because intuitively if the observed value for the primary outcome measures is equal to the clinically relevant threshold then the decision is likely to be go and this equates with setting q_1=0.5 in the decision criteria.

Note 2: In all cases the clinically relevant threshold is set to be the same at the interim analysis as it is at the final analysis.

Note 3: This probability is set at a high level to minimise the chance of incorrectly stopping recruitment early but with the levels relaxed slightly in some cases to ensure that there is a high probability of stopping early in situations when there is a lack of efficacy.

For the OR and DCB primary outcome measures, a Beta-Binomial conjugate analysis for the rate was used to evaluate the proposed design for a range of sample sizes. At this design stage, a non-informative Beta(1,1) prior was used. The current standard treatment in these patients is likely to give an OR rate of 10% and DCB rate of 20-25%; therefore 30% has been chosen for both as the critical threshold value in most targeted therapy, single agent cohorts with a higher level of 40% chosen for the combination treatment in Arm E, but 30% for the combination treatment in Arm J given observed rates on durvalumab. Sample sizes considered were based on requiring a minimum of 10 and maximum of 15 for the interim and minimum of 20 and maximum of 40 for the final. Sample size was chosen to minimise the chance of false positive conclusions and maximise the chance of true positive conclusions (further details given in the Statistical Analysis Plan). An interim sample size of 15 and final sample size of 30 provide operating characteristics that satisfy the pre-specified requirements. The operating characteristics that are calculated using exact binomial probabilities rather than simulation are given in the Statistical Analysis Plan and are applicable to each of the drug-(putative)biomarker cohorts that have either OR, DCB or both as their primary outcome measure. The design for Arms A, B, D, E and F were originally based on a single primary outcome measure of OR but this has since been adapted to include an additional more patient-centric primary outcome of DCB alongside the original OR, and operating characteristics have been revised accordingly (see Statistical Analysis Plan for further details).

For the PFS primary outcome measure, a conjugate analysis of the time-to-event outcome based on an exponential-inverse-gamma model (Thall *et al.* 2005) was used for medium PFS to evaluate the proposed design for a range of sample sizes. At this design stage, a non-informative IG(0.001,0.001) prior was used. The current standard treatment in these patients is likely to give a median PFS of 3 months (e.g. Hanna *et al.* 2004) so this is chosen as the critical threshold value. An interim sample size of 15 and final sample size of 30 provide operating characteristics that satisfy the pre-specified requirements. Operating characteristics have been calculated by simulating PFS data for 10,000 trials from exponential distributions under 6 different possible underlying truths (median=1 to median=6) and the results are given in the Statistical Analysis Plan. A second NA cohort has been added to Arm J, with the same

co-primary outcomes and success critiera (including interim analysis after 15 patients) as for the previous NA cohort, and the targeted J1 cohort.

15.4 Design, analysis and sample size for no actionable genetic change cohorts (Cohort NA1 & NAJ).

As described in Section 5, a secondary objective of the trial is to provide the opportunity for industrial partners to test novel agents in the cohort of patients who are not positive for any of the actionable targets in the trial, referred to as the no actionable genetic change cohorts. During the course of the trial, any drugs (including targeted agents) that are selected for allocation to the no actionable genetic change cohorts will be included in a pipeline of options that become available sequentially. No comparison whatsoever will be made between any agents tested on the no actionable genetic change arm. For each drug, the objective will be to evaluate whether there is a signal of activity in this no actionable genetic change cohort. For any targeted agents selected for testing in this no actionable genetic change group, this will provide some initial assessment regarding the biomarker specificity of drug activity. The statistical design and sample size for each drug will be developed specifically for each drug as it becomes selected for the cohort but in general it will take the form of an adaptive Bayesian design in line with the actionable target cohorts.

The first drug to be tested in this arm is durvalumab (Arm NA Cohort NA1). There are two co-primary outcomes to assess efficacy: OR and DCB. Drug availability fixed the initial sample size at 20 patients. Further recruitment will continue to a target of 30 patients in total. Further details are given in the Statistical Analysis Plan. The second intervention to be tested in the NA arm is the Arm J combination of drugs (Cohort NAJ). The statistical design for this NAJ cohort is the same as for cohort J1 on Arm J.

15.5 SMP2 Patients who do not enter the National Lung Matrix Trial

As described in Section 2, patients who have consented to SMP2 screening and follow up but:

- Do not have an actionable mutation as per the current trial protocol
- Are not eligible for a cohort in Arm NA
- Have an actionable target but are not otherwise eligible for the trial

will receive standard treatment or possibly treatment under an alternative trial protocol. There is no formal statistical design for this part of the trial and outcome measures for these patients will be collected in order to provide a descriptive analysis of relevant concurrent background information. Patients consent for this follow up as part of the SMP2 research protocol.

16 TRIAL ORGANISATIONAL STRUCTURE

16.1 Sponsor

The trial sponsor is the University of Birmingham.

16.2 Coordinating Centre

The trial is being conducted under the auspices of the CRCTU, University of Birmingham according to their local procedures.

16.3 Lead Investigators

Lead Investigators have been appointed for specific arms of the protocol (see Trial Contacts section of the protocol for a list). Lead Investigators were initially selected following a call for Expressions of Interest via the National Cancer Research Institute (NCRI) Lung Clinical Studies Group (CSG). Applicants were then selected via interview. Lead Investigators will take day to day responsibility for the running of the trial arm in conjunction with the National Lung Matrix Trial Office, with high level oversight of the Chief Investigator and the Sponsor. During recruitment Lead Investigators will be members of the Trial Management Group, and actively participate in Investigator Meetings and Trial Steering Committee meetings to discuss pharmacovigilance and operational matters.

16.4 Trial Management Group

The TMG will be chaired by Professor Sanjay Popat. Members of the TMG include the CI, CRCTU Trial Management Team Leader, Chief Biostatistician, Senior Trial Coordinator, Trial Coordinator, Trial Statistician and representatives from the following fields and organisations: Medical oncology, clinical oncology, pathology, pharmacy, early information unit, functional imaging, biomarkers, genomic analysis, radiology, CRUK and a patient representative. The TMG will meet or hold a teleconference every approximately 4 months during recruitment, or more often as required.

16.5 Trial Steering Committee

The role of the TSC is to provide overall supervision for the trial on behalf of the Trial Sponsor (University of Birmingham) and Trial Funder (CRUK) and to ensure that the trial is conducted to the rigorous standards set out in the GCP standards. In particular, the TSC will concentrate on progress of the trial, adherence to the protocol, patient safety, evidence on main efficacy outcome measures and the consideration of new information of relevance to the research question(s). The safety and well-being of the trial participants are the most important considerations and should prevail over the interests of science and society. The TSC will provide advice, through its Chair, Professor Richard Kaplan, to the Chief Investigator, the University of Birmingham and CRUK on all appropriate aspects of the trial, particularly whether it is appropriate to continue recruitment to cohorts. Membership of the TSC has been limited and includes an independent Chair, three other independent members (including a patient representative) and two trial Principal Investigators who do not sit on the TMG. The TSC will be asked to comment in detail on substantial changes to the protocol, including the addition of new arms. The TSC will meet at often as required, at least twice per year.

16.6 Independent Peer Review

The protocol has been submitted to an international selection of suitably qualified individuals and patient representatives from the NCRI Lung CSG. Written comments were anonymised and provided alongside the protocol for discussion by an independent expert review panel – the National Lung Matrix Trial Review Panel. The panel consists of members from CRUK funding committees namely the Clinical Research Committee (CRC), New Agents Committee (NAC) and Science Committee. This selection ensures the expertise in both experimental and confirmatory trials as well as biomarker and translational research. A patient representative is also present on the panel. The National Lung Matrix Trial Review Panel has given their approval for the protocol after which ethical approval is to be sought.

16.7 New Arm Incorporation

New study arms will be proposed to the Chief Investigator of the study or Cancer Research UK Strategic Partnerships team. Following initial feasibility assessment, proposals will be developed by the CRCTU with the approval and guidance from the Trial Management Group, and submitted to CRUK for funding review before regulatory and ethical approvals are sought.

16.8 Finance

This is a clinician-initiated and clinician-led trial funded by CRUK. Pharmaceutical companies including but not limited to AstraZeneca, Pfizer and Mirati Therapeutics are providing Investigational Medicinal Products.

Individual payments will be made to NHS Trusts for various research costs associated with the Trial. The trial has been independently peer reviewed and has been adopted by the NIHR Clinical Research Network (CRN) Portfolio.

17 ETHICAL CONSIDERATIONS

The trial will be performed in accordance with the recommendations guiding physicians in biomedical research involving human subjects, adopted by the 18th World Medical Association General Assembly, Helsinki, Finland, June 1964, amended at the 48th World Medical Association General Assembly, Somerset West, Republic of South Africa, October 1996 (website: http://www.wma.net/en/30publications/10policies/b3/index.html) (Appendix 7: WMA Declaration of Helsinki)
The trial will be conducted in accordance with the UK Policy Framework for Health and Social Care Research, the applicable UK Statutory Instruments, (which include the Medicines for Human Use Clinical Trials 2004 and subsequent amendments, the General Data Protection Regulation (GDPR) and Data Protection Act 2018 and Human Tissue Act 2008) and GCP. This trial will be carried out under a Clinical Trial Authorisation in accordance with the Medicines for Human Use Clinical Trials regulations. The protocol will be submitted to and approved by the main REC prior to circulation.

Before any patients are enrolled into the trial, the Principal Investigator at each site is required to obtain local Research & Development (R&D) approval. Sites will not be permitted to enrol patients until written confirmation of R&D approval is received by the National Lung Matrix Trial Office and the site is formally activated. Principal Investigators at Feeder Sites are also responsible for obtaining local R&D approval and will not be permitted to consent patients to performing mandatory repeat biopsies until written confirmation of R&D approval is received by the Trial Office and the site is formally activated.

It is the responsibility of the Principal Investigator to ensure that all subsequent amendments gain the necessary local approval. This does not affect the individual clinicians' responsibility to take immediate action if thought necessary to protect the health and interest of individual patients.

18 CONFIDENTIALITY AND DATA PROTECTION

Personal data recorded on all documents will be regarded as strictly confidential and will be handled and stored in accordance with the General Data Protection Regulation (GDPR) 2016/679 and the Data Protection Act 2018. With the patient's consent, their full name, date of birth, hospital number, NHS/CHI number and general practitioner (GP) details will be collected at trial entry to allow tracing through the Cancer Registries and the NHS Information Centre for Health and Social Care (service formally provided by the Office of National Statistics) and to assist with long-term follow-up via other health care professionals (e.g. patient's GP). Patients will be identified using only their unique trial number and initials on the Case Report Form and correspondence between the National Lung Matrix Trial Office and the participating site. However patients are asked to give permission for the National Lung Matrix Trial Office to be sent a copy of their signed Informed Consent Form which will not be anonymised. This will be used to perform in-house monitoring of the consent process. The local Investigator must maintain documents not for submission to the National Lung Matrix Trial Office (e.g. Patient Identification Logs) in strict confidence. In the case of specific issues

and/or queries from the regulatory authorities, it will be necessary to have access to the complete trial records, provided that patient confidentiality is protected.

The National Lung Matrix Trial Office will maintain the confidentiality of all patients' data and will not disclose information by which patients may be identified to any third party other than those directly involved in the treatment of the patient and organisations for which the patient has given explicit consent for data transfer (e.g. Cancer Registries). Representatives of the National Lung Matrix Trial team may be required to have access to patient's notes for quality assurance purposes but patients should be reassured that their confidentiality will be respected at all times.

19 INSURANCE AND INDEMNITY

In terms of liability at a site, NHS Trust and non-Trust hospitals have a duty to care for patients treated, whether or not the patient is taking part in a clinical trial. Patients not eligible for NHS care cannot be recruited due to indemnity limitations. Compensation is therefore available via NHS indemnity in the event of clinical negligence having been proven.

University of Birmingham employees are indemnified by the University insurers for negligent harm caused by the design or co-ordination of the clinical trials they undertake whilst in the University's employment. The University of Birmingham cannot offer indemnity for non-negligent harm. The University of Birmingham is independent of any pharmaceutical company, and as such it is not covered by the Association of the British Pharmaceutical Industry (ABPI) guidelines for patient compensation.

20 PUBLICATION POLICY

Results of each target-specific trial arm will be submitted for publication in a peer reviewed journal. The manuscript will be prepared by the TMG and authorship will be determined by mutual agreement. The National Lung Matrix Trial publication policy contains further information.

Any secondary publications and presentations prepared by Investigators must be reviewed by the TMG. Manuscripts must be submitted to the TMG in a timely fashion and in advance of being submitted for publication, to allow time for review and resolution of any outstanding issues. Authors must acknowledge that the trial was performed with the support of the University of Birmingham. Intellectual property rights will be addressed in the Clinical Study Site Agreement between Sponsor and site.

21 REFERENCE LIST

- Cancer Research UK Cancer Statistics website, available form URL: http://www.cancerresearchuk.org/cancer-info/cancerstats/. Accessed 3rd March 2014.
- Pao W & Hutchinson KE (2012). Chipping away at the lung cancer genome. *Nat Med* **18(3):**349-51.
- Drilon *et al.* (2012). Squamous-cell carcinomas of the lung: emerging biology, controversies, and the promise of targeted therapy. *Lancet Oncol* **13:** e418-26.
- Ding L, Getz G, Wheeler DA *et al.* (2008). Somatic mutations affect key pathways in lung adenocarcinoma. *Nature* **455(7216):1069-75.**
- Eisenhauer EA, Therasse P, Bogaerts J *et al.* (2009). New response evaluation criteria in solid tumours: Revised RECIST guideline (version 1.1). *Eur J Cancer* **45(2):**228-47.
- Paz-Ares LG *et al.* (2013). PARAMOUNT: Final Overall Survival Results of the Phase III Study of Maintenance Pemetrexed Versus Placebo Immediately After Induction Treatment

With Pemetrexed Plus Cisplatin for Advanced Non-squamous Non–Small-Cell Lung Cancer. *J Clin Oncol* **31(23):**2895-902.

- Pe'rol M *et al.* (2012). Randomized, Phase III Study of Gemcitabine or Erlotinib Maintenance Therapy Versus Observation, With Predefined Second-Line Treatment, After Cisplatin-Gemcitabine Induction Chemotherapy in Advanced Non–Small-Cell Lung Cancer. *J Clin Oncol* **30(28):**3516-24.
- Scagliotti GV *et al.* (2008). Phase III study comparing cisplatin plus gemcitabine with cisplatin plus pemetrexed in chemotherapy-naive patients with advanced-stage non-small-cell lung cancer. *J Clin Oncol* **26(21):**3543-51.Thall PF, Wooten LH & Shpall EJ (2006). A Geometric Approach to Comparing Treatments for Rapidly Fatal Diseases. *Biometrics* **62(1):193-201.**
- Hanna N, Shepherd FA, Fossella FV *et al*. (2004). Randomized Phase III Trial of Pemetrexed Versus Docetaxel in Patients With Non–Small-Cell Lung Cancer Previously Treated With Chemotherapy. *J Clin Oncol.* **22(9):1589-97.**

22 ARM A: AZD4547 – FGFR INHIBITOR *(CLOSED TO RECRUITMENT)*

Lead Investigator: Professor Gary Middleton

22.1 Background & Rationale

22.1.1 Molecular cohorts

Inhibitor: AZD4547

Arm	Investigational Medicinal Product	Cohort Number	NSCLC Histology	Molecular Cohort
**A**	AZD4547 – FGFR Inhibitor	**A1**	NSCLC	FGFR2 or FGFR3 mutation

AZD4547 is a potent inhibitor of FGFR1, 2 and 3 with IC_{50} values of 0.2, 2.5 and 1.8 nmol/L respectively (Gavine *et al.* 2012). It is a highly selective inhibitor: the only other kinase which it has activity against is vascular endothelial growth factor receptor (VEGFR)-2 but it is 120-fold more selective for FGFR compared to VEGFR2 activity. In cellular phosphorylation assays AZD4547 inhibited auto phosphorylation of FGFR 1, 2 and 3 with concentrations that results in 50% inhibition (IC_{50}) of 13, 2 and 40 nmol/L respectively. IC_{50} values for VEGFR2 was 258 nmol/L. In xenograft models known to be sensitive to VEGFR2 inhibition there was no activity of AZD4547.

22.1.2 Pre-Clinical Rationale

There is a strong pre-clinical rationale for testing the activity of AZD4547 in patients with squamous cell NSCLC harbouring FGFR mutations. A recent study analysed the activity of AZD4547 in the context of the FGFR mutations in squamous cell lung cancer collated in The Cancer Genome Atlas (TCGA) (Liao *et al.* 2013). 5.6% of patients had mutations in FGFR2 or 3 with approximately equal numbers for each receptor. Mutation was significantly enriched in the basal expression subtype. 3/10 harboured concomitant PIK3CA mutations, 2 concomitant RAS mutations and 3 concomitant human epidermal growth factor receptor (HER)-2 mutations. NIH-3T3 cells were engineered to stably express each of the individual mutations: the majority were transforming but 2 FGFR2 and 2 FGFR3 mutations were non-transforming. Extracellular domain (ECD) and kinase domain mutations were transforming and 3/4 non-transforming mutations were in neither of these regions. All of the ECD mutations were transforming. Activation of FGFR is caused by covalent receptor dimerization: mutant receptors form intermolecular disulphide dimers via the substitution of a cysteine residue and this was observed in the squamous lung cancer ECD mutants. The observed ECD mutations were sufficient to induce covalent receptor dimerization in the absence of ligand.

The transforming mutations were tumourigenic *in vivo* and FGFR inhibition significantly reduced tumour growth. Ba/F3 cells expressing the transforming FGFR mutations were dependent on FGFR signalling in the presence of FGF and in the absence of interleukin (IL)-3. Importantly, AZD4547 inhibited the proliferation of all of the transfectants with single digit nanomolar IC_{50} in more than a half. These data clearly show that the proliferation of transforming squamous cell FGFR mutant containing cells is sensitive to AZD4547 treatment. The exact nature of the mutation detected is likely to be critical in defining which tumours are likely to be sensitive to such therapy. Finally there is similar frequency of FGFR mutations in adenocarcinoma – FGFR2 and 3 combined 3.3% in the latest iteration of the TCGA dataset.

22.1.3 Clinical Data

Although FGFR amplification is a frequent event in squamous cell lung cancer, on-going core AZ programmes are already assessing the impact of AZD4547 in FGFR amplified squamous cell carcinoma. The current study described herein is specifically targeting FGFR mutation, leveraging the large number of patients undergoing molecular screening to generate the required number of patients.

In addition to the AstraZeneca-sponsored studies with AZD4547, a programme of Investigator-Sponsored Studies is underway.

D2610C00001

- Part A and B (SAD/MAD/Phase I) – completed.
- Part C: Cohort 1 (solid tumours), Cohort 2 (SCC NSCLC), Cohort 3 (Gastric) – completed.

D2610C00002 (Japan)

- Japanese patients (FTIH) Part A (all solid tumours) /B (lung, breast, gastric) – completed.

D2610C00003 (GLOW: Combination - Exemestane/Fulvestrant)

- Strata: FGFR1 amplified population (Fluorescent *in situ* hybridization [FISH] 6 and FISH 4/5).
- Safety Run In (comb with exemestane) – completed.
- Phase IIa (comb with Fulvestrant) ER+ve BC – completed.

D2610C00004 (SHINE: AZD4547 vs. Paclitaxel)

- Randomised Phase II gastric / gastro-oesophageal junction cancer versus paclitaxel.
- Strata: FGFR2 amplified population (FISH 6 and FISH 4/5) – terminated.

SAFIR02 (France)

- Lung: Phase II open-label multi-centre randomised trial for the evaluation of the efficacy of high throughput genome analysis as a therapeutic decision tool for patients with metastatic non-small cell lung cancer.
- Breast: Phase II open-label multi-centre randomised trial for the evaluation of the efficacy of high throughput genome analysis as a therapeutic decision tool for patients with metastatic breast cancer.

Royal Marsden Hospital Paired Biopsy

- Phase II POC study of AZD4547 in patients with FGFR1 or FGFR2 amplified tumours.

RADICAL

- Phase IIa AZD4547 in combination with anastrozole/letrozole + exemestane in ER+ve breast cancer - completed.

FACING

- Phase I/IIa: AZD4547 in combination with cisplatin + capecitabine (1st line gastric cancer, FGFR amplified) - completed.

FIESTA

- Phase Ib: AZD4547 in combination with gemcitabine + cisplatin (Cardiff + CTRU Leeds) Bladder cancer - completed.

LUNG-MAP

- Phase II/III Biomarker-Driven Master Protocol for Second Line Therapy of Squamous Cell Lung Cancer: Lung-MAP (Southwest Oncology Group): S1400 Biomarker-Targeted Second-Line Therapy in Treating Patients With Recurrent Stage IIIB-IV Non-Small Cell Lung Cancer (NCT Number: NCT02154490) – completed.

TARGET

- A Phase I/II, Open-Label, Multicentre Study to Assess The Safety, Tolerability, Pharmacokinetics and Clinical Efficacy of AZD4547 in Patients with glioma positive for an FGFR fusion Relapsed/Refractory

FRAME

- Phase II study of AZD4547 in patients with previously treated malignant pleural mesothelioma.

22.1.3.1 Clinical Pharmacokinetics

Preliminary invalidated PK data are available from Studies D2610C00001 (single and multiple bd dosing of 20 mg to 200 mg AZD4547 oral suspension formulation and 120 mg to 200 mg AZD4547 tablet formulation) and D2610C00003 (a Phase I/IIa AZD4547 study in combination with exemestane or fulvestrant in oestrogen receptor [ER+] breast cancer. Final reported data are available for D2610C00002 (single and multiple bd dosing of 40 to 120 mg and qd dosing of 160 mg AZD4547 oral tablet formulation in Japanese patients) and D2610C00004 (an open label Phase IIa study in AZD4547 monotherapy versus paclitaxel in advanced gastric cancer).

D2610C00001: Preliminary data suggest the administration of AZD4547 by either suspension or tablet formulation results in similar systemic exposures. AZD4547 has a moderate rate of absorption, with a median time to maximum plasma concentration (tmax) of 1 to 4 hours across all the dose levels following single doses and at steady state. Following a single dose of AZD4547, peak plasma concentrations declined with a consistent terminal elimination half-life (t1/2) across the dose-levels; mean value approximately 30 h. Oral clearance and distribution, although variable, were independent of dose and appropriate for the intended treatment regimen. The oral clearance was approximately 50 L/h and the oral volume of distribution was greater than total body water indicating that AZD4547 was well-distributed in the tissues. Multiple-dose data indicated that the accumulation ratio (RAC) was in keeping with the half-life and there was no unexpected time-dependency (the temporal change parameter [TCP] was generally close to unity).

D2610C00002: AZD4547 had a moderate rate of absorption, with a median tmax of 3 to 4 hours across the dose levels following single doses and at steady state. Following a single dose of AZD4547, peak plasma concentrations declined with a consistent t1/2 across the dose levels; mean value approximately 30 hours. The TCP was generally close to unity or slightly higher in most patients, indicating no notable time-dependency with multiple dosing. Comparison of the dose-normalised single dose profiles in Japanese and Western patients indicated that although plasma concentrations ranges were overlapping in the Western and Japanese, they were generally 30% lower in Japanese patients.

D2610C00003: Preliminary steady state AZD4547 data were available from up to 25 breast cancer patients receiving 40 mg or 80 mg bd AZD4547 with 25 mg qd exemestane in the safety run-in period. These data indicate that AZD4547 exposure, when administered in combination

with exemestane, was dose-proportional and consistent with previous monotherapy experience in Study D2610C00001. Although highly variable, the steady state PK of exemestane and 17-hydroxyexemestane following the 25 mg exemestane qd dosing regimen appeared to be largely unaffected by co-administration of AZD4547.

D2610C00004: Preliminary sparse sampled steady state data were available from 14 advanced gastric cancer patients receiving 40 mg or 80 mg bd, on a 2-week on treatment/1-week off schedule. Pharmacokinetic exposure was generally consistent with previous experience from Study D2610C00001.

22.1.3.2 Clinical safety and efficacy

Data reported here are based on a data cut off of 26 June 2015. At the time of data cut-off, recruitment to Study D2610C00001, Study D2610C00002, Study D2610C00003, and Study D2610C00004 is complete; study report is available for all these studies.

Enrolment to Study D2610C00004 was terminated in June 2013; based on the results of an interim analysis, it was concluded that Study D2610C00004 was unlikely to meet its primary objective of demonstrating superiority of AZD4547 monotherapy over paclitaxel. The decision only applied to patients with gastric cancer with tumours that had FGFR2 amplification (FISH score 6) receiving AZD4547 monotherapy and thus enrolment to Part C Cohort 3 of Study D2610C00001 was also terminated (08 July 2013). Patients with gastric cancer may receive AZD4547 in combination with other agents in clinical studies including Investigator Sponsored Studies. Enrolment to Part C Cohort 2 of Study D2610C00001 was subsequently terminated (on 12 December 2013) as the results did not meet the pre-defined efficacy criteria for continuation of the study. Enrolment to Part B of Study D2610C00003 has also been terminated (27 March 2014) as recruitment was much slower than predicted, leading to concerns about the feasibility of completing enrolment in a realistic timeframe. This, combined with the limited evidence of clinical activity observed with AZD4547 monotherapy in FGFR-amplified gastric cancer and squamous non-small cell lung cancer (Study D2610C00004 and Study D2610C00001), resulted in a business decision to terminate enrolment to this study.

The dose of AZD4547 being taken forward for further clinical study is 80 mg bd; specific data relating to this dose are presented for each study:

D2610C00001: A Phase I, Open-Label, Multicentre Study to Assess the Safety, Tolerability, Pharmacokinetics and Preliminary Anti-tumour Activity of Ascending Doses of AZD4547 in Patients with Advanced Solid Malignancies

This study was in 3 parts: Part A was a dose escalation in 8 cohorts (receiving doses from 20 mg to 200 mg bd), Part B was a safety and tolerability expansion and Part C was a safety, tolerability and efficacy expansion phase in 3 cohorts of different characterisations of tumour types. A total of 94 patients had received at least a single dose of AZD4547 in Study D2610C00001.

Cohorts were dosed with the suspension formulation as follows: 20 mg bd (n=3), 40 mg bd (n=5), 80 mg bd (n=6), 150 mg bd (n=7), and 200 mg bd (n=6). Cohorts were dosed with the tablet formulation as follows: 200 mg bd (n=4), 160 mg bd (n=6), and 120 mg bd (n=6). The total number of patients dosed in Part A was 43 patients.

In Part A, dose-limiting toxicities (DLTs) have been reported for 7 patients: raised liver function tests (80 mg bd [suspension]), mucositis (120 mg bd [tablet]), stomatitis (150 mg bd [suspension]), uncontrolled phosphate levels (160 mg bd [tablet]), renal failure (200 mg bd [suspension]), renal failure (160 mg bd [tablet]), and liver enzyme changes (200 mg bd [tablet]). The 160 mg bd dose was declared non-tolerated as 2/6 patients experienced DLTs. The

Safety Review Committee decided that the 120 mg bd dose was not sufficiently tolerated to support chronic dosing, although it did not achieve the protocol definition of a non-tolerated dose.

Part B (expansion phase) of the study has been completed and 6 patients have received AZD4547 80 mg bd as a continuous dose. No patient had a DLT in Part B.

Part C, exploring the safety, tolerability, PK and preliminary anti-tumour activity of AZD4547 in patients with FGFR1 and/or FGFR2 gene amplified tumours, has now completed. There are 3 cohorts for Part C: Cohort 1 – 20 patients with any solid tumour with FGFR1 or FGFR2 amplification (FISH score 6), Cohort 2 – 15 patients with squamous NSCLC that have tumours with FGFR1 amplification (FISH score 6) and Cohort 3 (enrolment terminated)– 10 patients with advanced gastric adenocarcinoma (including adenocarcinoma of the lower third of the oesophagus or the gastro-oesophageal junction) with tumours that have FGFR2 amplification (FISH score 6). A total of 45 patients have been dosed at 80 mg bd (continuous dose). No patient had a DLT in Part C. Enrolment to Cohort 3 in Part C was terminated early due to review of the data from Study D2610C00004.

Overall, the majority of adverse events (AEs) were Common Terminology Criteria for Adverse Events (CTCAE) Grade 1 or 2 in intensity.

The most commonly reported AEs (overall; all doses in all parts of the study [94 patients]) were constipation (43 [45.7%] patients); dry mouth (40 [42.6%] patients), stomatitis (39 [41.5%] patients), diarrhoea (33 [35.1%] patients), alopecia (32 [34.0%] patients), decreased appetite (31 [33.0%] patients), and vomiting (31 [33.0 %] patients).

The most frequently reported AEs, overall, at the AZD4547 80 mg bd dose (57 patients), were dry mouth (25 patients [43.9%]), stomatitis (23 patients [40.4%]; preferred term), constipation (22 patients [38.6%]), decreased appetite (21 patients [36.8%]), diarrhoea (21 patients [36.8%]), vomiting (21 patients [36.8%]), fatigue (19 patients [33.3%]), and nausea (17 patients [29.8%]).

There were 49 SAEs reported in 25 patients; this includes 1 SAE (respiratory distress) in 1 patient that occurred more than 28 days after the last dose in the study. Nineteen SAEs in 12 patients were considered by the reporting Investigator to be related to treatment with AZD4547. Asthenia, blood creatinine increased, chorioretinopathy, dehydration, dyspnoea, general physical health deterioration, renal failure, sepsis, and vomiting are SAE terms reported on more than one occasion.

Four patients have had CTCAE Grade 5 AEs (i.e., AE resulting in death); all were reported in Part C. One patient had a fatal AE of general physical health deterioration 56 days after the start of treatment; the AE was not considered by the reporting Investigator to be related to treatment with AZD4547. One patient had 4 fatal AEs (pericardial effusion, pleural effusion, respiratory failure and sepsis) 46 days after the start of treatment; all 4 events were considered by the reporting Investigator to be related to treatment with AZD4547. The patient had paracentesis for ascites 16 days prior to the events that may have triggered the sepsis with respiratory failure, pericardial effusion and pleural effusion. The patient's underlying metastatic gastric carcinoma with ascites also provides an alternative explanation for the occurrence of the events. One patient died due to euthanasia (this was at the patient's request) 59 days after the start of treatment; the event was not considered by the reporting Investigator to be related to AZD4547. From 2.5 weeks to 4 days prior to their death the patient had an SAE of epilepsy, the Investigator suspected disease progression. The remaining patient died due to respiratory distress; this patient died after database lock for Part C of the study. The event was not considered by the reporting Investigator to be related to treatment with AZD4547.

A total of 20 from 82 eligible patients in Study D2610C00001 have had a best response of partial response (1 patient) or prolonged stable disease (stable disease ≥7 weeks in Part A and Part B [8 patients] and stable disease ≥6 weeks in Part C [11 patients]; total 19 patients) based on tumour assessment by Response Evaluation Criteria In Solid Tumours (RECIST) 1.1. Note that Part C cluster patients are not included in the efficacy analyses. In Part A, 6 patients have had best response of prolonged stable disease; 2 patients in the 160 mg cohort (tablet) and 1 each in the 40 mg (suspension), 80 mg (suspension), 120 mg (tablet), and 200 mg (tablet) cohorts. In Part B, 2 patients (80 mg tablet) have had best response of prolonged stable disease. In Part C of the study, 1 patient has had a best response of partial response (Cohort 1) and 11 patients have had a best response of prolonged stable disease. Bone biomarker data are in the process of being analysed.

D2610C00002: A Phase I, Open-Label, Multicentre Study to Assess the Safety, Tolerability, Pharmacokinetics and Preliminary Anti-tumour Activity of Ascending Doses of AZD4547 in Japanese Patients with Advanced Solid Malignancies.

This study was in 2 parts: Part A was a dose escalation in 4 cohorts (receiving doses from 40 mg to 160 mg bd) with expansion and Part B was a safety, tolerability and efficacy expansion.

A total of 34 patients had received at least a single dose of AZD4547 in the Japan dose escalation study (D2610C00002). In Part A, cohorts were dosed with the tablet formulation as follows: 40 mg bd (n=3), 80 mg bd (n=6), 120 mg bd (n=6) and 160 mg qd (n=15). No DLTs were reported, and a maximum tolerated dose (MTD) was not defined. A total of 4 patients received at least a single dose of AZD4547 80 mg bd in the safety, tolerability and efficacy expansion (Part B) in patients with breast, gastric and squamous cancer with FGFR gene amplified tumours. Thus, a total of 10 patients received at least a single dose of AZD4547 at 80 mg bd in Study D2610C00002.

All but 1 patient receiving AZD4547 in Study D2610C00002 has had at least one AE. The majority of AEs reported have been CTCAE Grade 1 or 2 in intensity. The most commonly reported AEs in Part A were dysgeusia (14 [46.7%] patients), diarrhoea (12 [40.0%] patients), stomatitis (12 [40.0%] patients), hyperphosphataemia (11 [36.7%] patients), dry mouth (10 [33.3%] patients), and dry skin (9 [30.0%] patients). The most commonly reported AEs at the AZD4547 80 mg bd dose from Part A and Part B were stomatitis (6 [60.0%] patients), dry mouth (5 [50.0%] patients), dysgeusia (5 [50.0%] patients), nausea (4 [40.0%] patients), decreased appetite (3 [30.0%] patients), diarrhoea (3 [30.0%] patients), epistaxis (3 [30.0%] patients), hyperphosphataemia (3 [30.0%] patients), malaise (3 [30.0%] patients), neutropenia (3 [30.0%] patients), and vomiting (3 [30.0%] patients).

There have been 4 SAEs reported in 3 patients. In Part A, 1 patient had CTCAE Grade 3 stomatitis (during follow-up) and 1 patient had CTCAE Grade 3 nausea and CTCAE Grade 3 decreased appetite (on-treatment). In Part B, 1 patient had CTCAE Grade 3 decreased appetite on-treatment). The SAEs of nausea and decreased appetite in Part A were considered by the reporting Investigator to be related to treatment with AZD4547; the Investigator also listed morphine as a suspected medication. No deaths due to an AE have been reported during study treatment.

The best objective response observed (in the opinion of the Investigator) was prolonged stable disease (stable disease ≥4 weeks), which was recorded in 22/34 patients.

D2610C00003: A Randomised Double-blind Phase IIa Study (with Combination Safety Run-in) to Assess the Safety and Efficacy of AZD4547 in Combination with Fulvestrant vs. Fulvestrant Alone in ER+ Breast Cancer Patients with FGFR1 Polysomy or Gene Amplification Who Have Progressed Following Treatment with Prior Endocrine Therapy (Adjuvant or First-line Metastatic) (GLOW).

This study is in 2 parts; a safety run-in (Part A: AZD4547 in combination with exemestane), with 4 cohorts: Cohort 1: AZD4547 80 mg bd continuous, Cohort 2: AZD4547 40 mg bd continuous, Cohort 3: AZD4547 80 mg bd on an intermittent schedule of 1 week on/1 week off, Cohort 4: AZD4547 80 mg bd on an intermittent schedule of 2 weeks on/1 week off. The second part (Part B) is a randomised Phase IIa study, using AZD4547 in combination with fulvestrant.

Study D2610C00003 was originally designed to explore the effect of AZD4547 in combination with exemestane, at a time when exemestane was the standard of care in the tested population. However, during the run-in stage of the study, the BOLERO2 study, demonstrated superiority for exemestane in combination with everolimus over exemestane alone, as a result the exemestane plus everolimus combination effectively became the new standard of care. The addition of everolimus to the AZD4547 plus exemestane combination was considered but it was decided that this combination would not be tolerated by patients; therefore, fulvestrant was chosen as an alternative second-line therapy and was taken forward for use in the Phase IIa (Part B) of the study. A protocol amendment was submitted in December 2012 to change the combination endocrine agent/comparator from exemestane to fulvestrant for the Phase IIa part of this study. Hence, the safety run-in part of this study reports the use of AZD4547 plus exemestane, and the latter part of the study using AZD4547 plus fulvestrant is ongoing.

Enrolment to Part B of Study D2610C00003 was terminated on 27 March 2014 as recruitment was much slower than predicted, leading to concerns about the feasibility of completing enrolment in a realistic timeframe. This, combined with the limited evidence of clinical activity observed with AZD4547 monotherapy in FGFR-amplified gastric cancer and squamous non-small cell lung cancer (Study D2610C00004 and Study D2610C00001), resulted in a business decision to terminate enrolment to this study. The study has now been reported.

A total of 31 patients with ER+ breast cancer received at least a single dose of AZD4547 in combination with exemestane 25 mg in the safety run-in period (Part A) of Study D2610C00003. In all cohorts of the safety run-in period patients have received exemestane 25 mg for 7 days prior to co-administration of exemestane 25 mg with AZD4547 bd.

In the first cohort, 5 patients received AZD4547 80 mg bd dosed continuously. No DLTs have been reported. Although the 80 mg bd AZD4547 cohort did not fulfil the protocol definition of a non-tolerated dose (2/6 patients with a DLT within the 21-day combination evaluation period), the Safety Review Committee decided that 80 mg bd dosed continuously was not appropriate for chronic dosing in this patient population due to the emerging tolerability profile. In the second cohort, 5 patients received AZD4547 40 mg bd continuous dosing. Twelve patients were recruited in Cohort 3 (80 mg bd intermittent schedule 1 week on AZD4547 and 1 week off treatment). Nine patients were recruited in Cohort 4 (80 mg bd intermittent schedule 2 weeks on AZD4547 and 1 week off treatment).

A total of 5 patients with ER+ breast cancer have received at least a single dose of AZD4547 (80 mg bd intermittent dosing schedule [2 weeks on, 1 week off])) in combination with fulvestrant 500 mg in the randomised period (Part B) of Study D2610C00003. The majority of AEs reported for patients receiving AZD4547 in Study D2610C00003 have been CTCAE Grade 1 or 2 in intensity. In Part B, in the AZD4547+fulvestrant group, 2 patients discontinued treatment due to objective disease progression, 2 patients due to patient decision and 1 patient due to “Other” (general status decreasing). Overall, the most commonly reported AEs in Part A are: alopecia (19 [61.3%] patients), dry mouth (17 [54.8%] patients), dysgeusia (16 [51.6%] patients), constipation (12 [38.7%] patients), diarrhoea (11 [35.5%] patients), dry skin (11 [35.5%] patients), and nausea (11 [35.5%] patients).

The recommended dose for the Phase IIa Period of Study D2610C00003 (AZD4547 80 mg bd 2 weeks on/ 1 week off) was based on an analysis of data from the safety run-in period. The most commonly reported AEs in the 9 patients receiving AZD4547 80 mg bd on an intermittent schedule of 2 weeks on/1 week off have been: dysgeusia (6 patients), alopecia (5 patients), nausea (5 patients), decreased appetite (4 patients), dry mouth (4 patients), and nail disorder (4 patients; preferred term).

There were a total of 20 SAEs reported in 11 patients in Part A and Part B following treatment with AZD4547. In Part A, 1 patient in Cohort 1 had a single SAE: neutropenic sepsis. Two patients in Cohort 2 had an SAE: anaemia in 1 patient and 1 event of pleural effusion in the remaining patient. Five patients in Cohort 3 had at least 1 SAE: device deposit issue and renal failure (both reported in 1 patient); asthma, dyspnoea, jugular vein thrombosis, and VIIth nerve paralysis (all reported in 1 patient); pyelonephritis (1 patient); dizziness (1 patient), and troponin increased, lower respiratory tract infection viral, oesophageal achalasia, and stomatitis (all reported in 1 patient). One patient in Cohort 4 has had an SAE of diarrhoea. In Part B, where patients have been receiving fulvestrant 500 mg in combination with AZD4547 (80 80 mg bd continuous dosing), 4 SAEs have been reported in 2 patients (dizziness in 1 patient and inflammation, psoriatic arthropathy, and gait disturbance in the remaining patient). Seven of the 16 SAEs in the 10 patients in Part A were considered by the reporting Investigator to be related to treatment with AZD4547 (device deposit issue, diarrhoea, dizziness, oesophageal achalasia, renal failure, stomatitis, troponin increased). The SAE of dizziness was considered by the reporting Investigator to be related to treatment with both AZD4547 and exemestane (reported in 1 patient who received AZD4547 80 mg bd intermittently [1 week on/1 week] off). None of the 4 SAEs reported in Part B were considered by the reporting Investigator to be related to treatment with AZD4547. No patients have died due to an AE in Part A or Part B of the study.

Overall, 12 (38.7%) of Part A patients have discontinued study treatment due to AEs: 2 patients in Cohort 1 (nail discolouration and local swelling [swollen finger tips in 1 patient and depression and neuralgia in the remaining patient); 1 patient in Cohort 2 (chorioretinopathy); 5 patients in Cohort 3 (renal failure [SAE], dizziness [SAE], sub-retinal fluid, and detachment of retinal pigment epithelium [2 cases]), and 4 patients in Cohort 4 ([blood creatinine increased, detachment of retinal pigment epithelium, lethargy, and rash and skin ulcer [both of the latter terms were reported in 1 patient]). Overall, no patients in Part B of the study discontinued study treatment due to AEs.

Five patients in Part A had a dose reduction: 3 patients in Cohort 3 (80 mg bd intermittent schedule 1 week on AZD4547 and 1 week off treatment) due to paraesthesia and palmar-plantar erythrodysaesthesia syndrome (both reported in 1 patient), blister and palmar-plantar erythrodysaesthesia syndrome (both reported in 1 patient), and stomatitis (in 1 patient), and 2 patients in Cohort 4 (80 mg bd intermittent schedule 2 weeks on AZD4547 and 1 week off treatment) due to chorioretinopathy and ulcerative colitis. One patient in the AZD4547+fulvestrant group had a dose reduction due to an AE of palmar-plantar erythrodysaesthesia syndrome.

D2610C00004: A Randomised Open-Label Phase II Study to Assess the Efficacy and Safety of AZD4547 Monotherapy Versus Paclitaxel in Patients With Advanced Gastric Adenocarcinoma (Inc. Adenocarcinoma of the Lower Third of the Oesophagus or the Gastro-Oesophageal Junction)With FGFR2 Polysomy or Gene Amplification (Shine study).

A randomised, open label, multicentre study to compare AZD4547 monotherapy 80 mg on an intermittent schedule of 2 weeks on/1 week off versus paclitaxel in patients with advanced gastro-oesophageal tumours.

A total of 67 patients with advanced gastric cancer received study treatment with AZD4547 (40 patients; 80 mg bd 2 weeks on and 1 week off schedule) or paclitaxel (27 patients; 80 mg/m2).

Thirty-nine patients in the AZD4547 group had discontinued study treatment and 1 patient was ongoing. Most patients discontinued the study due to condition under investigation worsened (31 patients), followed by an AE (3 patients), “Other” (3 patients; death [2 cases] and performance score of 3) and subject decision (2 patients). Note: For 2 of the 5 patients with an AE leading to discontinuation from treatment listed below, a reason of death (both had fatal AEs) was given for discontinuing study treatment on the case report form. The discrepancy may have arisen as there are 2 case report forms, one form for completion/discontinuation from the study or treatment and another for AEs and resulting changes to study treatment.

The majority of AEs reported for patients receiving AZD4547 in Study D2610C00004 have been CTCAE Grade 1 or 2 in intensity. The most commonly reported AEs (of any grade) reported for patients receiving AZD4547 were: decreased appetite (16 [40.0%] patients), asthenia (11 [27.5%] patients), nausea (10 [25.0%] patients), constipation (10 [25.0%] patients), stomatitis (10 [25.0%] patients), abdominal pain (9 [22.5%] patients), abdominal pain upper (9 [22.5%] patients), dry mouth (9 [22.5%] patients), and vomiting (8 [20.0 %] patients).

Twelve on-treatment or post-treatment SAEs were reported for 8 patients receiving AZD4547 and 8 on-treatment or post-treatment SAEs had been reported in 6 patients receiving paclitaxel. Two SAEs were considered by the reporting Investigator to be related to treatment: stomatitis in 1 patient in the AZD4547 group and lower respiratory tract infection in 1 patient in the paclitaxel group.

Three deaths due to AEs were reported; 2 in the AZD4547 group (intestinal haemorrhage and arterial disorder) and 1 in the paclitaxel group (asthenia). None of the 3 events were considered by the reporting Investigator to be related to treatment. The patient who died of intestinal haemorrhage had a history of chronic gastritis.

AEs leading to discontinuation of study treatment were reported for 5 patients and 2 patients in the AZD4547 and paclitaxel groups, respectively. In the AZD4547 group these were: arterial disorder (1 patient; fatal; not considered by the reporting Investigator to be related to treatment), blood bilirubin increased (1 patient), intestinal haemorrhage (1 patient; fatal; not considered by the reporting Investigator to be related to treatment), RPED (1 patient), and RPED and bile duct obstruction (both terms in 1 patient). In the paclitaxel group these were: hypoaesthesia and malaise (both terms in 1 patient) and peripheral sensory neuropathy (1 patient).

Overall, in all randomised patients, the analysis of PFS did not show any statistically significant difference in favour of the AZD4547 arm, compared with the paclitaxel arm (HR 1.57, 80% CI: 1.12, 2.21). Similar results were observed for the FISH 6 strata (HR 1.30; 80% CI: 0.81, 2.12).

22.1.3.3 Marketing experiences

AZD4547 is not currently marketed in any territory.

22.1.4 Cohort Definition

Figure 11 shows the regions of the FGFR2 and 3 genes that are classified as Tier 1 and Tier 2 mutations. Novel mutations that occur in the ‘green’ regions are classified as Tier 2. Mutations that occur in the ‘red’ regions are Tier 3 and therefore ineligible. Table 8 lists the specific regions of the genes that are included in the ‘green’ regions of each gene.

Figure 11: FGFR2/3 Tier 1 and Tier 2 mutations

FGFR2 domains

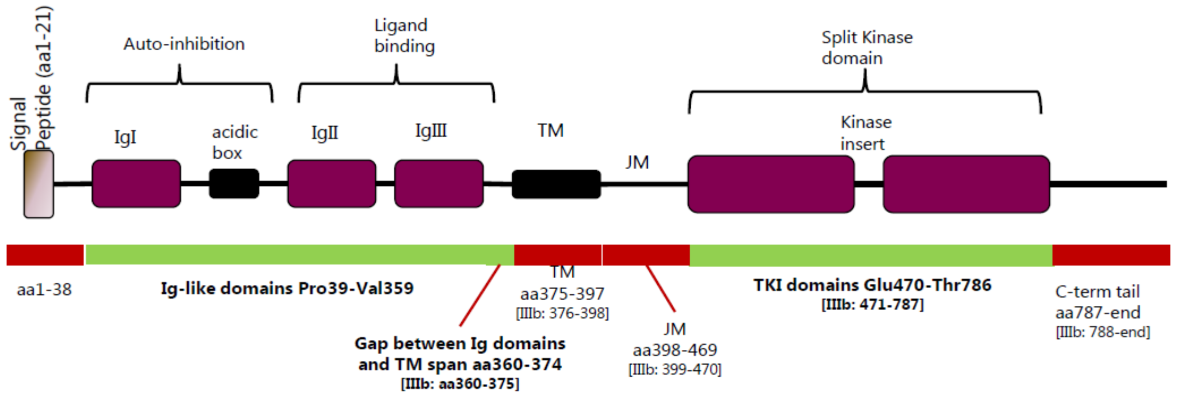

KEY

- Green: Novel mutations in these regions (not listed in TIER1 lookup) are TIER2
- Red: Mutations in this region not eligible (TIER3) unless known pathogenic/activating.

Residue numbering based on IIIc form (IIIb numbering in brackets where applicable).

Known (listed) pathogenic variants (germline data) or activating mutations (functional assay) override this position classification.

DOMAIN info from Ensembl:
IgI domain: Prosite Ig-like domain aa39-125; SMART Ig_sub aa47-125; SMART Ig_sub2 aa53-114.
IgII domain: Prosite Ig-like domain aa154-247; Pfam I-set domain aa172-248; Pfam Ig domain aa173-233
IgIII domain Prosite Ig-like domain aa256-358; Pfam I-set domain aa263-359; Pfam Ig domain aa271-343
TM helix aa375-397
InterPro superfamily Kinase-like domain aa470-786; InterPro Tyr_kinase_cat_domain aa481-757; SerThr dual kinase domain aa481-761; Prosite Prot_kinase_domain aa481-770. SCOP Superfamily Kinase-like domain 470-786. Longest domain definition taken here.
C-terminal tail defined as aa787-821 (IIIc), 822 (IIIb form).

FGFR3 domains

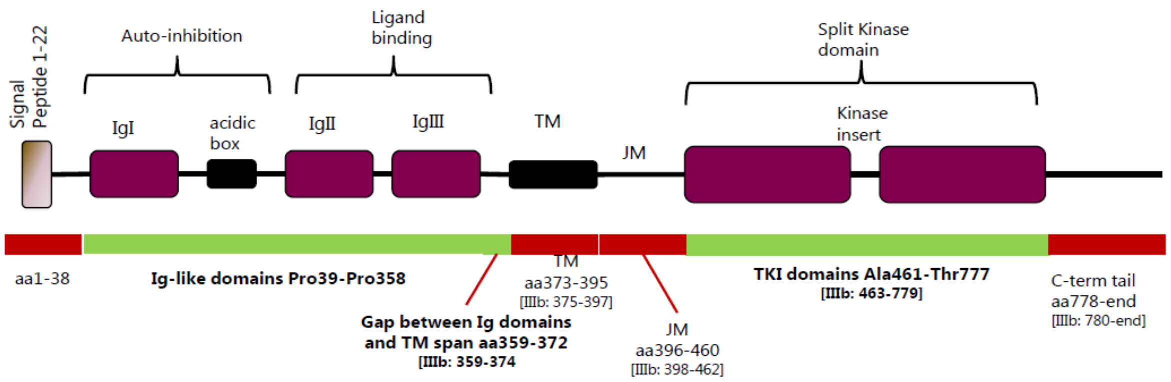

KEY

- Green: Novel mutations in these regions (not listed in TIER1 lookup) are TIER2
- Red: Mutations in this region not eligible (TIER3) unless known pathogenic/activating.

Residue numbering based on IIIc form (IIIb numbering in brackets where applicable).

Known (listed) pathogenic variants (germline data) or activating mutations (functional assay) override this position classification.

DOMAIN info from Ensembl:
IgI domain: Prosite Ig-like domain aa39-110; SMART Ig_sub aa46-126; SMART Ig_sub2 aa52-116.
IgII domain: Prosite Ig-like domain aa151-244; Pfam I-set domain aa167-245; Pfam Ig domain aa170-230
IgIII domain Prosite Ig-like domain aa253-355; Pfam I-set domain aa260-356; Pfam Ig domain aa268-340;
V-set domain reaches to Pro358, took this as final edge of the IgIII domain.
TM helix aa373-395;
Pfam protein kinase domain aa473-747; Prosite Prot_kinase_domain aa472-761; SMART Ser/Thr dual_sp kinase domain aa472-752; Superfamily kinase-like domain aa461-777. Longest domain definition taken here.
C-terminal tail defined as 778-806 (808 for IIIb form)

Table 8: Tier 1 and Tier 2 actionable mutations for FGFR2 and FGFR3.

Actionable Tier	FGFR2	FGFR3
TIER1	Only mutations as listed in the TIER1 lookup list.	Only mutations as listed in the TIER1 lookup list.
TIER2 (green)	Any mutation altering amino acids Pro39 to Val374 (inclusive) or Glu(E)470 to Thr786 (inclusive) and not listed in the TIER1 list are considered eligible as TIER2. Numbering based on IIIc form (ENST00000358487). These regions encompass the Extracellular (Ig-like) domains and the cytosolic TK-domains as well as the junction region between IgIII domain and the TM span.	Any mutation altering amino acids Pro39-Pro372 OR Ala461-Thr777 (inclusive) and not listed in the TIER1 list are considered eligible as TIER2. Numbering based on IIIc form (ENST00000440486). These regions encompass the Extracellular (Ig-like) domains and the cytosolic TK-domains as well as the junction region between IgIII domain and the TM span.

22.2 Specific Eligibility Criteria

22.2.1 Inclusion Criteria

For inclusion in the study, patients should fulfil all of the following criteria:

- Patients must fulfil all the core eligibility criteria.
- Technology hub result (or locally obtained result from an approved Laboratory if applicable.
- Eastern Cooperative Oncology Group (ECOG) Performance Status $\leq$1 with no deterioration over the previous 2 weeks (see Appendix 8: Eastern Cooperative Oncology Group Performance Status Criteria).
- For patients with EGFR wildtype (wt) tumours, prior therapy must include platinum-based chemotherapy. For patients with tumours harbouring an EGFR mutation known to be associated with EGFR TKI sensitivity, prior therapy must include an EGFR tyrosine kinase inhibitor. For patients with ALK-positive tumours, prior therapy must include an ALK inhibitor.
- Ability to swallow oral medication.
- Aged at least 25 years (skeleton maturation is complete).

22.2.2 Exclusion Criteria

Patients should not enter the study if any of the following exclusion criteria are fulfilled:

- Patients who do not fulfil all the core eligibility criteria.
- Treatment with any of the following:
    - Any concomitant medication known to prolong the QT interval within the required washout period prior to the first dose of AZD4547 (see Section 22.2.3.1).
    - Potent inhibitors or inducers of CYP3A4 or 2D6 or substrates of CYP3A4 within the required washout period prior the first dose of AZD4547:
        - Inhibitors: see Table 11 in Section 22.2.3.2 for prohibited medications and relevant washout periods (see Table 13 for inhibitors permitted with caution)
        - Inducers: See Table 12 in Section 22.2.3.2 for prohibited medications and relevant washout periods.
        - Substrates: See Table 14 in Section 22.2.3.2 for prohibited medications and relevant washout periods (see Table 15 for substrates permitted with caution).
    - Nitrosourea or mitomycin C within 6 weeks prior to the first dose of AZD4547.
    - Any prior treatment with any agent with FGFR inhibition as its primary pharmacology.
    - Malaria prophylaxis with chloroquine within 12 months prior to the first dose of AZD4547 (see Table 9 in Section 22.2.3.1.1).
    - Antiarrhythmic medication with amiodarone within 12 months prior to the first dose of AZD4547 (see Table 9 in Section 22.2.3.1.1).
    - Radiotherapy of the primary site with a wide field of radiation within 4 weeks, or radiotherapy with a limited field of radiation for palliation within 2 weeks prior to the first dose of AZD4547.
    - Any other chemotherapy, investigational agents or other anti-cancer therapy within 4 weeks prior to the first dose of AZD4547.
- Corrected calcium >ULN within 7 days of treatment.
    - Corr. Calcium = Total Calcium (mmol/L) + ([40 – Albumin (G/L)] x 0.02)
- Phosphate >ULN within 7 days of the first dose of AZD4547.
- Hepatic function (in patients **with** liver metastasis).

  - Alanine transferase (ALT) and Aspartate transferase (AST) >5 x ULN.
- Any of the following cardiac criteria:
  - Mean resting corrected QT interval (QTcF) ≥470 msec obtained from 3 consecutive ECGs.
  - Any clinically important abnormalities in rhythm, conduction or morphology of resting ECG e.g., complete left bundle branch block, third degree heart block, second degree heart block, first degree heart block.
  - Any factors that increase the risk of QTc prolongation or risk of arrhythmic events such as heart failure, hypokalaemia, congenital long QT syndrome, family history of long QT syndrome, or unexplained sudden death under 40 years of age.
- History of hypersensitivity to active or inactive excipients of AZD4547 or drugs with a similar chemical structure or class to AZD4547.
- Any of the following ophthalmological criteria:
  - Current evidence or previous history of RPED.
  - Previous laser treatment or intra-ocular injection for treatment of macular degeneration.
  - Current evidence or previous history of dry or wet age-related macular degeneration.
  - Current evidence or previous history of retinal vein occlusion (RVO)
  - Current evidence or previous history of retinal degenerative diseases (e.g., hereditary).
  - Current evidence or previous history of any other clinically relevant chorioretinal defect.

22.2.3 Restrictions & Concomitant Medications

Information on any treatment from the date of informed consent until 28 days after the administration of the last treatment dose should be recorded. If medically feasible, patients taking regular medication should be maintained on it throughout the study period. Patient should be advised to inform their treating physicians of all concomitant medications, including prescription medicines, over-the-counter drugs, vitamins, and herbal products.

Nb. These lists are not exhaustive and the absence of a drug from the lists does not imply that its combination with AZD4547 is safe.

22.2.3.1 QT Interval Prolongation

Concomitant medications **known to prolong the QT interval** are prohibited during the active treatment phase. Any patients taking such drugs at or prior to registration should discontinue the drug prior to commencing trial treatment. **Please note the washout period for QT interval prolonging drugs in Arm A differs from other arms of the National Lung Matrix Trial (see Section 22.2.3.1.1)**.

In addition, **a number of permitted drugs with a possible TdP risk have an associated minimum treatment period on medication prior to commencing AZD4547 (see Section 22.2.3.1.2).**

For a list of drugs known to prolong the QT interval, (or with any risk of prolonging the QT interval), please refer to the following database: https://www.crediblemeds.org. Appendix 9 Credible Meds List of Drugs that Prolong QT Interval contains a list exported from this database on 2nd March 2018. **It is important to note that this list is a guide – the database will change with time and therefore needs to be checked in real-time when screening and**

registering a patient, and throughout their treatment. The Trials Office will email all sites when updates are made to the database.

Please note that for AstraZeneca trial Arms, only concomitant medications listed on https://www.crediblemeds.org under the category '**Known risk of TdP'** will exclude patients from entering the trial, as per the eligibility criteria.

It is the responsibility of an Investigator (Consultant level) to review and clinically evaluate all concomitant medications. Please contact the National Lung Matrix Trial Office for clarification regarding any drugs that appear on the database that are not listed in Appendix 9 Credible Meds List of Drugs that Prolong QT Interval.

The drugs listed on this website are taken from information provided by The Arizona Centre for Education and Research on Therapeutics and The Critical Path Institute, Tucson, Arizona and Rockville, Maryland. Important Note - If a patient is being treated with such medication or is taking another medication that may affect QT interval which is not on the database, please contact the National Lung Matrix Trial Office to obtain the recommended withdrawal/ minimum period prior to starting trial treatment.

22.2.3.1.1 Pre-treatment washout period for drugs known to prolong the QT interval

Unlike other arms of the National Lung Matrix Trial, the washout period of 6 half lives plus 1 day prior to initiation of trial treatment for drugs known to prolong the QT interval **does not apply to all concomitant medications**. Washout periods for the drugs listed in Table 9 below must be adhered to prior to the first dose of AZD4547. For any drugs not listed in Table 9, the washout period of 6 half lives plus 1 day applies:

Table 9: Withdrawal periods for select drugs known to prolong the QT interval

**Contraindicated drug**	**Withdrawal period prior to AZD4547 initiation**
Droperidol, Erythromycin, Procainamide	2 days
Cisapride, Clarithromycin, Disopyramide, Dofetilide, Deomperidone, Ibutilide, Sotalol, Sparfloxacin, Thioridazine	7 days
Bepridil, Chlorpromazine, Halofantrine, Haloperidol, Mesoridazine	14 days
Levomethadyl, Methadone, Pimozide	4 weeks
Arsenic trioxide*	6 weeks
Pentamidine	8 weeks
Amiodarone, Chloroquine	1 year

*estimated value as pharmacokinetics of arsenic trioxide has not been studied.

22.2.3.1.2 Minimum treatment period for drugs with a possible risk of TdP

Unlike other arms of the National Lung Matrix Trial, a number of permitted drugs that are possibly associated with QT interval prolongation have a minimum period that the patient must have been taking the concomitant medication prior to commencing treatment with AZD4547. This is to ensure the patient is stable on the therapy prior to trial treatment, and applies only to the drugs listed in Table 10 below:

Table 10: Withdrawal periods for select drugs known to prolong the QT interval

Permitted drug	Minimum treatment prior to AZD4547 initiation
Alfuzosin, Chloral hydrate, Ciprofloxacin, Dolasetron, Foscarnet, Galantamine, Gemifloxacin, Isradipine, Levofloxacin, Mexiletine, Nicardipine, Octreotide, Ofloxacin, Ondansetron, Quetiapine, Ranolazine, Telithromycin, Tizanidine, Vardenafil, Venlafaxine, Ziprasidone	2 days
Amantadine, Amirtriptyline, Amoxapine, Clozapine, Doxepin, Felbamate, Flecainide, Fluconazole, Fosphenytoin, Gatifloxacin, Granisetron, Imipramine, Indapamide, Lithium, Moexipril/Hydrochlorothiazide, Moxiflocacin, Risperidone, Roxithromycin, Sertraline, Trimethoprim-Sulfa, Trimipramine, Voriconazole	7 days
Azithromycin, Citalopram, Clomipramine, Itraconazole, Nortriptyline, Paroxetine, Solifenacin, Tacrolimus	14 days
Fluoxetine	5 weeks
Protriptyline	6 weeks
Tamoxifen	8 weeks

22.2.3.2 Cytochrome P450 (CYP) Related Inhibition and Induction

Concomitant use of medicines significantly metabolised by CYP3A4 will be contraindicated during the course of the trial. Use of other agents less significantly metabolised may be permitted with caution if considered clinically indicated for the welfare of the patients, and patients should be closely monitored for possible drug interactions.

The following restrictions will therefore be put in place in the trial, please see the tables below for listings of relevant drugs:

22.2.3.2.1 Drugs affecting CYP3A or CYP2D6 metabolism AstraZeneca strongly recommend are not combined with AZD4547

Table 11: Prohibited CYP3A4 or CYP2D6 inhibitors may increase exposure to AZD4547

Potent CYP3A4 or CYP2D6 inhibitors may increase exposure to AZD4547 more than 3-fold.

Drug	Minimum washout period prior to AZD4547 initiation
Ketoconazole<br>Ritonavir<br>Saquinavir<br>Indinavir<br>Nefazodone	Minimum of 48 hours washout prior to AZD4547 administration and must avoid for the duration of the trial and for 14 days following discontinuation of AZD4547
Itraconazole<br>Clarithromycin (250mg or 500mg bd)<br>Erythromycin<br>Fluconazole 400mg<br>Quinidine<br>Methimazole<br>Large quantities of grapefruit or Seville oranges (see 22.2.3.4 for permitted quantity)	Minimum of 7 days washout prior to AZD4547 administration and must avoid for the duration of the trial and for 14 days following discontinuation of AZD4547
Diltiazem<br>Paroxetine	Minimum of 14 days washout prior to AZD4547 administration and must avoid for the duration of the trial and for 14 days following discontinuation of AZD4547
Fluoxetine	Minimum of 35 days washout prior to AZD4547 administration and must avoid for the duration of the trial and for 14 days following discontinuation of AZD4547

Table 12: Prohibited Inducers of CYP3A4 may reduce exposure to AZD4547

Potent Inducers of CYP3A4 may reduce exposure to AZD4547 by more than 3-fold.

Drug	Minimum washout period prior to AZD4547 initiation
Barbiturates<br>Carbamazepine<br>Phenytoin<br>Rifampicin, Rifabutin	Minimum of 14 days washout prior to AZD4547 administration and must avoid for the duration of the trial and for 14 days following discontinuation of AZD4547
St John's Wort	Minimum of 21 days washout prior to AZD4547 administration and must avoid for the duration of the trial and for 14 days following discontinuation of AZD4547

There are currently no data confirming that there is a PK interaction between these agents and AZD4547; a potential interaction is considered on the basis of preclinical data only. This list is not intended to be exhaustive, and a similar restriction will apply to other agents that are known to strongly modulate CYP3A4 or CYP2D6 activity. Appropriate medical judgement is required. Please contact the National Lung Matrix Trial Office with any queries you have on this issue.

22.2.3.2.2 Drugs affecting CYP3A metabolism that AstraZeneca considers may be allowed with caution.

Table 13: Permitted (with caution) moderate Inhibitors of CYP3A4/CYP2D6 may increase exposure to AZD4547

Drug	Warning of possible interaction
Duloxetine<br>Nelfinavir<br>Terbinafine<br>Verapamil	Drugs are permitted but caution should be exercised and patients monitored closely for possible drug interactions. Please refer to full prescribing information for all drugs prior to co-administration with AZD4547.
Grapefruit juice<br>Seville oranges (and other products containing Seville oranges)	Patients should abstain from eating large amounts of grapefruit and Seville oranges (and other products containing these fruits e.g., grapefruit juice or marmalade) during the trial (e.g., no more than a small glass of grapefruit juice (120 mL) or half a grapefruit or 1-2 teaspoons (15 g) of Seville orange marmalade daily).

22.2.3.2.3 Medicines that are significantly metabolised by CYP3A4 (substrates) that AztraZeneca strongly recommend are not combined with AZD4547.

Table 14: Prohibited medicines metabolised by CYP3A4 (substrates)

Exposure, pharmacological action and toxicity may be increased by inhibition of CYP3A4 by AZD4547.

Drug	Minimum washout period prior to AZD4547 initiation
Alfentanil<br>Cyclosporin<br>Tacrolimus<br>Atorvastatin<br>Lovastatin<br>Simvastatin	Minimum of 7 days washout prior to AZD4547 administration and must avoid for the duration of the trial and for 14 days following discontinuation of AZD4547
Carbamazepine	Minimum of 14 days washout prior to AZD4547 administration and must avoid for the duration of the trial and for 14 days following discontinuation of AZD4547.

There are currently no data confirming that there is a PK interaction between these agents and AZD4547; a potential interaction is considered on the basis of preclinical data only. This list is not intended to be exhaustive, and a similar restriction will apply to other agents with narrow therapeutic windows that are known to depend on CYP3A4 for metabolism. Appropriate medical judgement is required. Please contact the National Lung Matrix Trial Office with any queries you have on this issue.

22.2.3.2.4 Medicines that are significantly metabolised by CYP3A4 that AztraZeneca considers may be allowed with caution.

Table 15: Permitted (with caution) medicines metabolised by CYP3A4 (substrates)

Exposure, pharmacological action and toxicity may be increased by inhibition of CYP3A4 by AZD4547.

Drug	Warning of possible interaction:
Alprazolam<br>Midazolam<br>Triazolam<br>Felodipine<br>Isradipine<br>Nifedipine<br>And possibly other calcium antagonists<br>Methylprednisolone	Drugs are permitted but caution should be exercised and patients monitored closely for possible drug interactions. Please refer to full prescribing information for all drugs prior to co-administration with AZD4547.

22.2.3.3 Concomitant Radiotherapy

If a patient requires palliative radiotherapy, consideration should be given to suspending trial treatment based on local practice and following discussion with the National Lung Matrix Trial Office.

22.2.3.4 Food Restrictions

Patients should abstain from eating large amounts of grapefruit and Seville oranges (and other products containing these fruits e.g., grapefruit juice or marmalade) during the trial (e.g., no more than a small glass of grapefruit juice (120 mL) or half a grapefruit or 1-2 teaspoons (15 g) of Seville orange marmalade daily).

22.2.3.5 Other Restrictions

St John's Wort is not permitted during treatment with AZD4547. See Table 12 in Section 22.2.3.2.1 for pre-treatment washout period.

22.2.3.6 Contraception

No reproductive toxicology or teratogenic studies have been conducted with AZD4547 to date, and it is unknown whether the drug is excreted in human milk. Therefore, women of childbearing potential and men should agree to use adequate contraception prior to trial entry and for the duration of trial participation and until at least 90 days after completion of treatment, and women who are breast feeding are excluded from any trial. A definition of females of childbearing potential and females of non-childbearing potential and acceptable methods of contraception are described in Section 6.3.

Both women and men should be fully informed of the lack of reproductive toxicity testing, and women must have a negative pregnancy test prior to enrolment.

22.3 Trial Treatment

22.3.1 Investigational Medicinal Product

A cycle of trial treatment will be defined as 21 days.

AZD4547 is a potent and selective inhibitor of fibroblast growth factor receptor (FGFR)-1, 2 and 3 receptor tyrosine kinases (enzyme and cellular phosphorylation endpoints), and has a

significantly lower potency for inhibition of insulin-like growth factor 1 receptor (IGF1R) and kinase insert domain receptor (KDR). The tablet is presented as two strengths: 20 or 80 mg.

AZD4547 will be supplied free of charge by AstraZeneca. AZD4547 has been classified as a Schedule 1 Controlled Drug by the Home Office, and is therefore only open to recruitment at select sites with a specific Schedule 1 Controlled Drug license for the purposes of the National Lung Matrix Trial. Please refer to the Pharmacy Manual, or contact the National Lung Matrix Trial Office for a complete list of sites to which patients eligible for Arm A should be referred for trial treatment.

The dosing schedule will be continuous dosing at 80 mg bd, twice daily doses should be taken approximately 12 hours apart. Where possible all doses of AZD4547 should be taken at approximately the same times each day.

With regard to missed doses, patients should try their best to take the test compound as directed however, the kinetics of AZD4547 (30h half-life) mean that PK profiles are generally quite flat and will tolerate occasional lapses in compliance. A missed dose can be taken within 6 hours of the scheduled time. It is important that there are no patient double doses (i.e. takes 2 doses simultaneously). Please ensure that any compliance failures are recorded so that we can estimate the degree of compliance in the trial subjects. If vomiting occurs within 30 minutes after AZD4547 dosing, or later if the tablet(s) can be identified in the vomit content, the patient can re-take new tablet(s). Both the Arm A Patient Information Sheet and Arm A Patient Diary contain more specific instructions for patients to follow regarding how to take their medication.

Please note patients who meet RECIST criteria for progressive disease (PD) may be continued on trial treatment if the treatment is tolerable and the Investigator believes it to be of clinical benefit; see Section 9.3.

Please also refer to the Pharmacy Manual for further details.

22.3.2 Schedule of Assessments

Table 16: AZD4547 - Schedule of Assessments

	**Screening**	**Treatment 80 mg bd (21 day cycles)**					**Discontinuation** (+ 7 days)**	**28 day follow up visit[t]** (+ 7 days)**	**Post-28 day follow up** (± 7 days)***
	Within 28 days of treatment (unless otherwise stated)	Cycle 1			Cycle 2	Cycle 3 onwards			
		Day 1	Day 8	Day 15	Day 1 (± 2 days)*	Day 1 (± 2 days)*			
Informed consent[a]	X								
Demography & baseline characteristics[b]	X								
Medical history[c]	X								
Inclusion / exclusion criteria[d]	X								
Physical examination[e]	X	X			X	X	X		
ECOG performance status	X (within 14 days of treatment)	X			X (± 7 days)	X (± 7 days)	X		
Vital signs (inc. weight)[f]	X	X	X		X	X	X		
ECG[g]	X		X		X	X	X		
Multi-gated acquistion scan (MUGA) / Echocardiogram (ECHO)[h]	X				X (± 7 days)	X (every 12 weeks ± 7 days)			
Ophthalmology examination[i]	X	Monthly for first 3 months, then every 8 weeks (± 7 days)							
Haematology, Clinical chemistry, Urinalysis[j]	X (within 7 days of treatment)	X	X	X	X (- 2 days)	X (- 2 days)	X		

	Screening	Treatment 80 mg bd (21 day cycles)					Discontinuation (+ 7 days)**	28 day follow up visit[t] (+ 7 days)**	Post-28 day follow up (± 7 days)***
	Within 28 days of treatment (unless otherwise stated)	Cycle 1			Cycle 2	Cycle 3 onwards			
		Day 1	Day 8	Day 15	Day 1 (± 2 days)*	Day 1 (± 2 days)*			
Pregnancy test[k]	X	X					X		
Tumour assessments[l]	X	Every 6 weeks during year 1 (± 7 days) [except 1st scan + 7days only]							X ◊
Adverse events & Concomitant Medications	X	X	X	X	X	X	X	X	
Dispense trial drug[m]		X			X	X			
Administer trial drug[n]		BD Dosing							
Smoking status[o]		X				X (every 9 weeks)	X		
Germline DNA sample[p]		X							
ctDNA samples[q]		X				X (every 9 weeks) (- 2 days)	X		X $
Optional research biopsy[r]		X (post-reg, pre-tx)					X		
Survival status[s]									X

* Visit may occur ± 2 days of the planned visit date. Individual assessments may occur independently of the visit date where indicated in the table above

** Visit may occur + 7 days of the planned visit date

*** Visit may occur ± 7 days of the planned visit date

a Prior to the start of any trial specific procedures, each patient must provide signed informed consent.

b Demography must be captured for all patients. Demographic data and other characteristics will include: date of birth, gender, race/ethnicity.

c A standard medical and surgical history will be obtained, including prior cancer treatment.

d Patients must not be registered unless all eligibility criteria have been fully met.

e Physical examination includes general appearance, respiratory, cardiovascular, skin, head and neck (including ears, eyes, nose and throat), lymph nodes, thyroid, abdomen, musculo-skeletal (including spine and extremities) and should be performed at Screening, prior to dosing on day 1 of every cycle and at discontinuation.

f Single measurements of supine blood pressure (BP) and pulse will be recorded on each occasion after 10 minutes rest. Vital signs to be taken at screening, **pre-dose** cycle 1 day 1 and day 8, cycle 2 day 1 and cycle 3 day 1. On day 1 of subsequent cycles and discontinuation vital signs can be taken at any time of day.

g ECG to be taken at screening, pre-dose cycle 1 day 8, cycle 2 day 1 and cycle 3 day 1. On day 1 of subsequent cycles and discontinuation ECG can be taken at any time of day. Twelve-lead ECGs will be obtained after the patient has been resting semi-supine for at least 10 minutes prior to times indicated. All ECGs should be recorded with the patient in the same physical position. For each time point unless specifically stated above, three ECG recordings should be taken at a minimum of 5 minute intervals (all three ECGs must be collected within 30 minutes). A standardised ECG machine should be used and the patient should be examined using the same machine throughout the trial if possible. After paper ECGs have been recorded, the Investigator or designated physician will review each of the ECGs and may refer to a local cardiologist if appropriate. A paper copy should be filed in the patient's medical records. If an abnormal ECG finding at screening or cycle 1 day 1 is considered to be clinically significant by the Investigator, it should be reported as a concurrent condition. For all ECGs details of rhythm, ECG intervals (R-R, PR, QT and QRS) and an overall evaluation will be recorded.

h A scan carried out within 3 months prior to starting trial treatment can be used for eligibility at screening, however, for patients who experience a significant cardiac event or who have had cardiotoxic medication within this interval, the MUGA/ECHO scan should be repeated prior to starting trial treatment. MUGA/ECHO should be performed on cycle 2 day 1 (± 7 days) then every 12 weeks - completion of every 4 cycles until discontinuation and as clinically indicated. The modality of the cardiac function assessments must be consistent within patient and the same machine operator is to be used where possible.

i Ophthalmology examination to be performed at screening, monthly for the first 3 months then every 8 weeks (± 7 days) whilst on treatment. At any other time, abnormal visual symptoms or signs will trigger a full ophthalmological review. The ophthalmic assessment should be performed on each occasion by the same ophthalmic expert where possible.

The following assessments will be performed in the order stated:

- Visual acuity (best corrected) including near and far vision for each eye separately
- Corneal examination (Fluorescein staining)
- Amsler grid
- Schirmer's test without anaesthesia – read after 5 minutes (this test should be done before instillation of stains or dilatory agents)
- Slit lamp examination
    - Apply 1 drop of 2% fluorescein followed by 1 drop of normal saline
    - Apply Lissamine Green (only applicable if Lissamine Green is used as local standard practice)
    - Measure intra-ocular pressure

  - Photograph any abnormalities
- Slit lamp fundoscopy and lens examination following pupil dilatation should be performed using binocular equipment and a 78-dioptre lens (or nearest available equivalent lens).

Optical Coherence Tomography (OCT) scans of the macula area of both eyes should be performed at baseline and monthly for the first 3 months on trial treatment. After this time, an OCT scan should be performed on the occurrence of clinical symptoms or signs suggestive of RPED. OCT is the AstraZeneca preferred methodology for diagnosis of RPED. If OCT is not available as part of local clinical practice, an equivalent alternative diagnostic methodology to screen for RPED should be used.

j Samples to be collected at screening (within 7 days of treatment), **pre-dose** cycle 1 day 1, 8 and 15. Samples for subsequent cycles can be taken up to 2 days earlier than the actual visit date (commencement of cycle). On day 1 of subsequent cycles and discontinuation, blood sample can be taken at any time of day.

Clinical Chemistry: Albumin, AST, ALT, Alkaline phosphatase (ALP), bilirubin (total), calcium (total), creatinine, glucose, magnesium, phosphate, sodium, troponin I or T, urea nitrogen, potassium and total protein.

If a patient experiences a doubling of phosphate from baseline or a corrected calcium:phosphate product >4.5 mmol^2/L^2 and phosphate chelation therapy (non-calcium containing agent) should be initiated. Management of patients will be according to the corrected calcium result provided from the Investigational site laboratory. Additional clinical chemistry samples will be taken weekly until resolution of the parameter to below the intervention limit.

Haematology: Full Blood Count (FBC).

Urinalysis: Protein, glucose, blood. If urinalysis abnormal, perform microscopy – red blood cells, white blood cells, bacteria, casts, crystals.

Laboratory values that meet the criteria for CTCAE grade 3 or have changed significantly from baseline and are considered to be of clinical concern will be repeated/confirmed within 7 days and followed up as appropriate.

Laboratory tests do not need to be repeated at cycle 1 day 1 if within 2 days of the screening sample.

k Investigator should assess the patient’s compliance to contraceptive measures and perform a test if required. Female patients of child-bearing potential only. A serum or urine pregnancy test is to be performed at screening, pre dose on cycle 1 day 1 and at discontinuation. In the event of suspected pregnancy during the trial, the test should be repeated and, if positive, the patient discontinued from trial treatment immediately.

l CT or MRI scan of head, chest and abdomen to be performed at screening. CT or MRI scans of chest and abdomen to be performed until discontinuation. Tumour assessments should be performed relative to the date of start of treatment (cycle 1 day 1) every 6 weeks for the first year, then every 12 weeks. Scans should be performed ± 7 days (except 1st scan + 7 days only). If brain metastases are identified at screening or if clinically indicated, head scanning will also be performed throughout treatment at the same time points. **The same imaging modality must be used consistently throughout the course of the trial for each patient.**

◊

Tumour assessments will be performed in follow up for patients who discontinue treatment for reasons other than Progressive Disease (e.g. toxicity). These scans should continue to be performed on a 6-weekly basis for the first year relative to the start date of treatment, then every 12 weeks until disease progression or the patient starts a new anti-cancer therapy (unless the patient withdraws consent to do so). Scans should be of the chest and abdomen, and only include the head where brain metastases are identified at screening, or if clinically indicated. All scans to be reported using RECIST 1.1.

m AZD4547 must be dispensed within the IWRS Cenduit system. Refer to the Pharmacy Manual for further details. AZD4547 may be dispensed within the IWRS up to 2 days prior to the actual visit date.

n Cycle 1 day 1: Treatment must commence within 7 days of trial registration.

o Smoking status data will be collected through questions and carbon monoxide (CO) monitoring **pre-dose** cycle 1 day 1, cycle 4 Day 1 then every 9 weeks (Day 1 of every third cycle) and at discontinuation.

p A whole blood germline DNA sample is to be collected **pre-dose** on cycle 1 day 1. If the sample is not collected at this time point, it should be collected at the next visit. Refer to the Laboratory Manual for sample processing guidelines.

q ctDNA samples to be collected at **pre-dose** cycle 1 day 1, cycle 4 Day 1 then every 9 weeks (Day 1 of every third cycle) and at discontinuation. Samples can be taken up to 2 days earlier than the actual visit date (commencement of cycle) where indicated. Refer to Laboratory Manual for sample processing guidelines.

$ ctDNA samples will be collected in follow up for patients who discontinue treatment for reasons other than Progressive Disease (e.g. toxicity). These samples should be performed at the same visit as follow up CT or MRI scans until disease progression or the patient starts a new anti-cancer therapy (unless the patient withdraws consent to do so). Samples should be collected on a 6-weekly basis for the first year relative to the start date of treatment then every 12 weeks.

r An optional fresh metastatic/recurrent tumour biopsy sample should be collected (if patient consents) post–registration (pre-treatment) and at the end of treatment visit for patients who discontinue treatment for reasons other than disease progression (origin from either the primary tumour of site of metastasis). An optional pre-treatment biopsy should not be performed in cases where the patient has already had a mandatory biopsy for molecular testing (Note - a mandatory repeat SMP2 biopsy will be performed if the patient has had targeted therapy e.g. ALK inhibitor). The discontinuation biopsy must be performed prior to commencing further anti-cancer therapy. A post-treatment biopsy will only be requested from patients with an objective response or stabilisation of disease (PR or CR), or 6 months on treatment with evidence of stabilisation (SD) for patients who have previously progressed. The tumour tissue will be used to determine possible mechanisms of resistance to study treatment. Refer to the Laboratory Manual for sample processing instructions.

s Survival status will be collected every 12 weeks (± 7 days) post-permanent discontinuation of AZD4547 until death.

t 28 day follow up visit should be carried out 28 days (+ 7 days) post-permanent discontinuation of AZD4547.

22.3.3 Toxicity Profile

A consistent safety and tolerability profile has been seen across the AZD4547 clinical programme.

22.3.3.1 Expected Adverse Events

The information below summarises emerging safety information for AZD4547 80mg bd continuous (n=62) and presents those events (specific MedDRA preferred terms that characterise each event are provided) that are to be regarded as expected for safety reporting purposes. Further detail is contained in Table 17.

- Blood creatinine increased: analysis of the laboratory values for serum creatinine showed that 3.0% of patients have had an abnormal serum creatinine value while taking AZD4547 or during the follow-up period. Creatinine levels are routinely and closely monitored throughout all studies. There has been 1 CTCAE grade 3 with one patient discontinuing.
- Diarrhoea: Diarrhoea, faecal incontinence, frequent bowel movement.
- Dysgeusia (altered taste) - mostly CTCAE grade 1 or 2.
- Epithelial and mucosal dryness (conjunctivitis, dry eye, dry mouth, dry skin, dry throat, keratitis, lacrimation decreased, lip dry, mucosal dryness, vulvovaginal dryness, xeroderma, xerophthalmia): mostly CTCAE grade 1 or 2.
- Hair and Eyelash disorders: distichiasis, eyelash thickening, growth of eyelashes, hair colour changes, hair disorder, hair growth abnormal, hair texture abnormal, madarosis, trichiasis, trichomegaly - mostly CTCAE grade 1 or 2.
- Hyperphosphataemia: Blood phosphorus increased, calcium phosphate product increased, hyperphosphataemia - mostly CTCAE grade 1 or 2 and managed, if necessary, with chelation therapy based on the patient's calcium phosphate product.
- Nail Disorder: Nail atrophy, nail bed bleeding, nail bed disorder, nail disorder, nail discolouration, nail growth abnormal, nail pigmentation, nail ridging, onychalgia, onychomadesis, paronychia - mostly CTCAE grade 1 or 2 with one patient discontinued.
- RPED: detachment of macular retinal pigment epithelium, detachment of retinal pigment epithelium, sub-retinal fluid and serous retinal detachment - mostly CTCAE grade 1 or 2 with one patient experiencing CTCAE grade ≥3 and one patient discontinuing therapy. RPED is now monitored using OCT and RPED management guidelines are in place for all studies. Patients can now be safely dosed through to resolution of these lesions.
- Stomatitis: Aphthous stomatitis, glossitis, mouth ulceration, mucosal inflammation, oral mucosa erosion, oral mucosal erythema, oral pain, stomatitis, tongue disorder – mostly CTCAE grade 1 or 2.
- Transaminases increased: ALT increased, AST increased, hypertransaminasemia, transaminases increased (CTCAE grade 1-3) – increase in transaminases from baseline has been seen and are routinely monitored throughout all studies. No Hy's law patient to date.

Table 17: AZD4547 - Frequency of Adverse Events

Number of patients who had at least 1 AE having received at least 1 dose of AZD4547 in the 80 mg twice daily continuous schedules (Study D2610C00001 and Study D2610C00002)

Medical Concept	Total frequency Number (%) N=62[a]	AEs with severity of CTCAE ≥3	AEs leading to dose discontinuation[b]	AEs leading to dose interruption[b]	AEs leading to dose reduction[b]
Epithelium and mucosal dryness	42 (62.7)	2 (3.0)	1 (1.5)	2 (3.0)	0
Stomatitis	34 (50.1)	3 (4.5)	0	3 (4.5)	0
Dysgeusia	20 (29.9)	0	0	1 (1.5)	0
Nail disorder	18 (26.9)	2 (3.0)	0	2 (3.0)	0
Hyperphosphataemia	17 (25.4)	0	0	0	1 (1.5)
Alopecia	11 (16.4)	Not applicable	0	0	0
RPED[c]	14 (20.9)	2 (3.0)	3 (4.5)	6 (9.0)	1 (1.5)
Transaminases increased	6 (9.0)	3 (4.5)	1 (1.5)	2 (3.0)	0
Hair and eyelash disorders	6 (9.0)	0	0	0	0
Blood creatinine increased	2 (3.0)	1 (1.5)	1 (1.5)	1 (1.5)	0

a The frequency of patients with events presented in the table are based on reported AEs. Therefore, the frequencies of increased blood creatinine, increased transaminases and hyperphosphataemia do not include laboratory changes that have not been reported as AEs.

b The last action taken for an event is presented (i.e., if a patient had an interruption followed by a discontinuation, the event is presented as a discontinuation). If a patient had the same event on more than 1 occasion (with differing start and stop dates) the last action taken for each event is presented; thus, the patient could be reported in the interruption, reduction and discontinuation columns.

c In this table, RPED is described by the following MedDRA Preferred Terms (PTs): chorioretinopathy, detachment of macular retinal pigment epithelium, detachment of retinal pigment epithelium, and retinal detachment. Henceforth, RPED will be described by the following amended list of MedDRA PTs: detachment of retinal pigment epithelium, detachment of macular retinal pigment epithelium, subretinal fluid, and serous retinal detachment (a MedDRA lowest level term that codes to PT: 'retinal detachment'). This amendment has not been applied retrospectively to the clinical trial databases.

Data cut-off 26 June 2015.

22.3.3.1.1 Ocular toxicity

Eye changes such as conjunctivitis and corneal atrophy have been seen in rats and dogs with AZD4547. In the clinical studies conducted to date the AEs reported regarding the anterior aspect of the eye are consistent with the pathological changes that were seen pre-clinically. At the AZD4547 80 mg bd dose in each of the 4 studies CTCAE Grade 1 or 2 dry eyes have been reported.

It is anticipated that patients will report any visual disturbances or discomfort relating to the eye in advance of any significant pathology such as ulceration occurring. The decision to continue on trial treatment if mild corneal changes in the eye examination are observed will be

left to the local Investigator's discretion, since a patient may indicate a wish to tolerate minor discomfort if there is perceived clinical benefit from the therapy. Any such events must be clinically managed to prevent secondary consequences, e.g., secondary infections following corneal abrasions. Lubricating eye drops/replacement tears should be used; if there is any indication of extra eyelash growth or eyelashes rubbing on the cornea then these eyelashes should be removed. A patient should be immediately discontinued from trial treatment if corneal ulceration occurs, and appropriate expert ophthalmological consultation should be initiated.

RPED and subretinal fluid accumulation (SRF) are both features of central serous retinopathy, in which fluid accumulates in different layers of the retina. In RPED, fluid accumulates between the retinal pigmented epithelium (RPE) and Bruch's membrane; in SRF, fluid accumulates between the neurosensory retina and the RPE. The accumulation of fluid gives the appearance of a 'detachment' of the layers of the retina. Symptoms of RPED/SRF are apparent when the central retina (macula) is affected. Such symptoms include blurred or distorted vision (metamorphosia), a blurred or grey spot in the central visual field and reduced visual acuity. Review of the current data suggests it is RPED that has been seen with ADZ4547. RPED/SRF may be seen on ophthalmoscopy; however, high resolution OCT is required for a diagnosis and accurate differentiation of the 2 conditions.
Regular OCT monitoring was implemented after the first 4 cases of RPED were diagnosed by OCT scans following patients' reports of 'decreased vision' in Study D2610C00001.

RPED has been identified in clinical studies with AZD4547. RPED and subretinal fluid accumulation (SRF) are both features of central serous retinopathy, in which fluid accumulates in different layers of the retina. In RPED, fluid accumulates between the retinal pigmented epithelium (RPE) and Bruch's membrane; in SRF, fluid accumulates between the neurosensory retina and the RPE. The accumulation of fluid gives the appearance of a 'detachment' of the layers of the retina. Symptoms of RPED/SRF are apparent when the central retina (macula) is affected. Such symptoms include blurred or distorted vision (metamorphosia), a blurred or grey spot in the central visual field and reduced visual acuity. Review of the current data suggests it is RPED that has been seen with ADZ4547. RPED/SRF may be seen on ophthalmoscopy; however, high resolution OCT is required for a diagnosis and accurate differentiation of the 2 conditions. Regular OCT monitoring was implemented after the first 4 cases of RPED were diagnosed by OCT scans following patients' reports of 'decreased vision' in Study D2610C00001.

Regular OCT monitoring was implemented after the first 4 cases of RPED were diagnosed by OCT scans following patients' reports of 'decreased vision' in Study D2610C00001. Following implementation of regular OCT monitoring, more than 40 cases of RPED (at any dose in any study) were diagnosed on the scheduled OCT scans in the AZD4547 programme. Of these, more than 26 patients have had an event of RPED following continuous or intermittent dosing with AZD4547 80 mg bd. In addition to the RPED events, 3 cases of patients with other retinal abnormalities including macular oedema and retinal oedema) were reported based upon OCT scans following AZD4547 80 mg continuous dosing in Study D2610C00001 and Study D2610C00002.

The majority of these events of RPED and other posterior eye changes were diagnosed after 21 days of treatment. Where OCT follow-up scans were available, most cases had partial or complete remission within 2 weeks of stopping treatment. In the patients, who went on to recommence treatment at a lower dose after the initial AE of RPED/related conditions had resolved, the condition recurred. After recurrence of the events, the study treatment was permanently discontinued and the events resolved without sequelae. Analysis of the data to date has not identified a relationship between the incidence of RPED and the AUC, C_{max} or minimum plasma concentration (C_{min}) of AZD4547.

22.3.3.1.2 Mineralisation, particularly in the heart

The cardiac mineralisation identified in both pre-clinical species is thought to be as a direct consequence of elevated serum phosphate levels. The increase in phosphate levels are thought to be pharmacological as a consequence of inhibition of FGF23 modulated phosphate homeostasis in the kidney (Razzaque and Lanske 2007). In the dog, the increase in phosphate level occurred prior to mineralisation, and at lower doses where no mineralisation occurred. Mineralisation was of low incidence, and was not present following 4 weeks off dose. The clinical studies to date have confirmed the pre-clinical finding of increases in serum phosphate and following review of the data hyperphosphataemia is considered to be an expected event in patients treated with AZD4547. There have though been no reports, and no evidence of any soft tissue, including cardiac, mineralisation clinically. However based upon the presumption that increases in phosphate precede mineralisation, patients should be excluded from the trial if they have phosphate or calcium levels above the ULN prior to entry. Serum phosphate and calcium should be included in the standard clinical chemistry safety bloods which should be assessed on a regular basis as per the individual trial plan. Any patient who experiences a doubling of phosphate from baseline or a corrected calcium:phosphate product >4.5 mmol/L should have phosphate chelation therapy initiated, and weekly clinical chemistry assessments performed until resolution of the parameter to below the intervention limit. Investigators must seek appropriate specialist medical consultation (renal or metabolic) to advise on the prescription and titration of phosphate chelation agents, and to raise the patients' awareness of low phosphate diets.

Mineralisation occurring within the heart will result in functional changes prior to any gross structural changes being apparent by specific imaging technology. Therefore, patients should have regular troponin I measurements at the same time as the clinical chemistry safety blood measurements; in addition ECGs should be assessed regularly as detailed in the individual trial plan and MUGA scans or echocardiograms for assessment of Left ventricular ejection Fraction (LVEF) should be performed as specified in 22.3.2 in order to identify functional changes. The protocol should also include standard exclusion criteria for unstable cardiac conditions and risk factors for QTc prolongation.

22.3.3.1.3 Renal toxicity

Creatinine is a breakdown product of creatine phosphate in muscle, and is usually produced at a fairly constant rate by the body (depending on muscle mass). Creatinine is removed from the blood chiefly by the kidneys, primarily by glomerular filtration, but also by proximal tubular secretion. Little or no tubular reabsorption of creatinine occurs. If the filtration in the kidney is deficient, creatinine blood levels rise. Therefore, elevation of serum creatinine is often taken to indicate an impairment in renal function. However, in theory, elevations in serum creatinine may occur if the renal handling of creatinine is affected without indicating an impairment in renal function. Pre-clinically, mild to moderate elevations in serum creatinine levels were noted sporadically in rats, with no clear dose relationship across the dose levels. Mineralization in the kidney tubules was also seen in rats at high doses, although not present in the 1-month study, and was considered to be due to hyperphosphataemia.

Elevations in serum creatinine (mainly Grade 1 or 2) have been commonly observed in patients on AZD4547 treatment or during the follow-up period. Serum creatinine levels improved when AZD4547 was interrupted or permanently discontinued. Increased serum creatinine is considered as an expected event in patients treated with AZD4547. Hyperphosphatemia, which is reversible and can be managed with chelation therapy, has been reported in clinical studies with AZD4547 and is considered to be a drug-related effect.

In some patients, the increase in serum phosphate occurred with increases in serum creatinine. The magnitude of change is not the same but the general trend upwards or

downwards is matched. Intermittent dosing schedules show the effect more clearly, and the pattern can be seen in patients with normal serum creatinine values, as well as those whose creatinine rises above the ULN. It is not clear whether elevations in serum creatinine in patients receiving AZD4547 is indicative of an impairment of renal function. The medical/scientific literature describes the action of FGF23 as a phosphaturic ligand of FGFR1 in the kidney tubules. Data from other FGFR inhibitors, mixed tyrosine kinase inhibitors that include FGFR inhibition amongst other targets, have reported patients with renal failure; however, this is confounded by the advanced cancer populations studied. In clinical studies of AZD4547 in advanced cancer populations there have been 5 reports of renal failure (all SAEs) in 5 patients. Each case contains evidence of possible alternative explanations for the events, such as the presence of a renal tumour, adrenal metastases, advanced cancer and concomitant drugs.

All patients should be closely monitored for any signs of impaired renal function. Patients should be excluded from studies if they have serum creatinine >1.5 times the ULN concurrent with creatinine clearance <50 mL/min (measured or calculated by Cockcroft and Gault equation – see Appendix 4: Cockcroft Gault Formula – Creatinine Clearance). Serum creatinine and blood urea nitrogen should be included in the standard clinical chemistry safety bloods and should be assessed on a regular basis as per the schedule of assessments.

22.3.3.1.4 Bone turnover

Histopathological changes in bone structure have been identified in the rat but not the dog. Similar bone changes have been reported in the literature following administration of another FGFR inhibitor to rats (Brown *et al.* 2005), and have been considered due to a pharmacological effect on growing bones. Patients born with mutations in FGFR genes develop a range of skeletal disorders during childhood such as osteoglyphonic dysplasia, Apert syndrome and hypochondroplastic dwarfism (White *et al.* 2005). Therefore, patients under the age of 25 should not be permitted to enter any study, in order to exclude individuals who have not completed maturation of their skeleton. Bone AEs should be reviewed on a case-by-case basis as it is not possible to provide specific stopping criteria given the background of extensive metastatic disease seen with the advanced cancer patient population, which might result itself in pathological fractures and bone pain. Regular monitoring of the serum calcium and phosphate levels is described above with regards to mineralisation. A sample of blood for the measurement of specific bone turnover biomarkers (including PTH, Vitamin D3, βCTX, PINP and osteocalcin) may be taken at baseline and at intervals during the study to evaluate the effect of study treatment on bone homeostasis mechanisms. Patients who have received bisphosphonate therapy in the 6 months prior to the start of a study, or who are still receiving them, should not have the bone biomarker sample taken due to the action of these drugs on the parameters of interest.

22.3.3.1.5 Mouth-related conditions

Events of ageusia, dry mouth, dysgeusia, and stomatitis/oral mucositis have been reported in the clinical studies to date and following review of the data are considered to be expected events in patients treated with AZD4547. In cases of stomatitis particular attention should be given to prophylaxis, maintaining a high standard of oral hygiene with the regular use of antibacterial mouthwashes during the study. Saline nasal sprays may help nasal mucosal dryness and so reduce the incidence of epistaxis.

22.3.3.1.6 Dermatological toxicity

There have been a number of events reported in patients receiving treatment with AZD4547 involving the skin and associated appendages. These include events of dry skin, hair changes, trichomegaly and changes to the nails and nail beds. Following review of the data these topics are considered to be expected events in patients treated with AZD4547.

22.3.3.1.7 Diarrhoea

Diarrhoea has been commonly reported in patients receiving AZD4547 across all AstraZeneca sponsored studies, the majority of which have been non-serious and CTCAE Grade 1. 1 patient (receiving AZD4547 in combination with fulvestrant) has required a dose modification (interruption); no patients have required dose discontinuation at the AZD4547 80 mg dose. Following review of the data, diarrhoea is considered as an expected event in patients treated with AZD4547. Patients have responded to symptomatic treatment, for example with loperamide.

22.3.3.1.8 Neutropenia

Neutropenia and febrile neutropenia are common risk factors for cancer patients receiving chemotherapy treatment. A review of the individual laboratory data for patients enrolled in AZD4547 clinical studies has not identified any dose relationship with neutropenia, most events are CTCAE Grade 1 or Grade 2 and were not considered by the reporting investigator to be related to treatment with AZD4547. Events of CTCAE Grade 4 neutropenia have been reported; treatment for neutropenia was given, as appropriate. One SAE (neutropenic sepsis in Study D2610C00003) has been reported 28 days after the last dose of AZD4547; the event resolved (duration of 5 days) and was not considered by the reporting Investigator to be related to treatment with AZD4547.

Neutropenia has been reported with dovitinib (multi-kinase inhibitor), TSU-6 (mixed tyrosine kinase inhibitor), and BIBF-1120 (multi-kinase inhibitor). Most of the changes reported with these agents have been Grade 1 and Grade 2 with a few patients having a slight shift towards Grade 4 changes (André *et al*. 2013, Bousquet *et al*. 2011, du Bois *et al*. 2010, Kim *et al*. 2011, Okamoto *et al*. 2012, Shin *et al*. 2012). One patient who received dovitinib died due to treatment emergent neutropenia and infection after 51 days of treatment (André *et al*. 2013). Neutropenia was the most frequently reported treatment-related AE for TSU-68 (Kim *et al.* 2011, Okamoto *et al*. 2012, Shin *et al*. 2012) and a common Grade 3/4 AE with BIBF-1120 (Bousquet *et al.* 2011, du Bois *et al*. 2010). However, neutropenia is not included in the list of most frequently reported AEs for BGJ398 or JNJ-42756493 in the advanced solid tumour population; BGJ398 and JNJ-42756493 have a similar safety profile to AZD4547 (Bahleda *et al.* 2014, Dienstmann *et al*. 2014, Sequist *et al.* 2014). There is no mechanism discussed in the literature regarding the FGFR inhibitory effects on bone marrow that could result in neutropenic events in AZD4547 clinical studies. Neutropenia and febrile neutropenia are common risk factors for cancer patients undergoing chemotherapy, and a compromised haematological profile is a confounding factor.

Any AE of neutropenia should be managed as deemed appropriate by the Investigator with close follow up and interruption of study drug if CTCAE Grade 3 or worse neutropenia occurs. If a patient develops febrile neutropenia, study treatment should be stopped and appropriate management including granulocyte-colony stimulating factor should be given according to local hospital guidelines.

22.3.3.1.9 Increases in transaminases

Increases in transaminases have been reported in the clinical studies to date and following review of the data are considered to be expected events in patients treated with AZD4547. Most of these increases were CTCAE Grade 1 or Grade 2. There was no clear relationship between the dose of AZD4547 and the incidence or severity of the increase in transaminases. Intermittent dosing schedules have allowed examination of the effect of dechallenge and rechallenge with AZD4547, and while there was some evidence of an effect with AZD4547 in a few patients, overall, there was little difference in the data obtained from dosing with continuous or intermittent schedules. Liver function test abnormalities were most common in

patients with liver metastases and in patients with progression of their underlying cancer; multiple concomitant medications in these patients is also a confounding factor.

Studies of AZD4547 include regular measurements of ALT, AST and other hepatic biochemistry parameters. Health care professionals involved in clinical studies with AZD4547 should look for signs of liver toxicity in patients receiving study drug. All patients with an ALT, AST or bilirubin value above the upper limit of normal at the time of the last dose of AZD4547 should have a further liver chemistry profile (AST, ALT, bilirubin and ALP) performed 30 days after permanent discontinuation of AZD4547 in order to evaluate reversibility.

22.3.3.1.10 Asthenic conditions

Reported incidence rates for cancer-related fatigue in the clinical trial setting tend to be in the range of 70% to 80%. The prevalence of fatigue in the palliative care setting is in the range of 48 to 78% (Lawrence 2004). Literature for multi-targeted kinases suggests there may be an association between asthenic events and the use of FGFR and VEGF inhibitors; however, it is difficult to suggest there is an association directly with FGFR inhibitors. There is also a suggestion from pre-clinical findings that FGF may modulate some of the metabolic processes such as FGF-19 (ligand for FGFR4) and FGF21 (ligand for FGFR 1, 2, 3, and 4) that can regulate glucose, lipid, and energy metabolism and cause changes in energy expenditure; this may provide a mechanism for why asthenic events occur with FGFR inhibitors (Tomlinson *et al*. 2002).

The incidence of asthenic reported terms were very commonly reported for patients receiving AZD4547 80 mg bd continuously and for patients on intermittent schedules of AZD4547 80 mg bd (1 week on/1 week off and 2 weeks on/1 week off), although fewer patients had asthenic events in the intermittent schedules. Analysis of data from Study D2610C00004 shows a lower percentage of patients treated with AZD4547 had asthenic events (45.0%) compared with patients receiving paclitaxel (55.6%). The Summary of Product Characteristics for paclitaxel lists asthenia and malaise as rare events (1:1000 to 1:10000) suggesting that the background disease (gastric cancer) could provide an alternative cause for the high incidence of asthenic reported terms in both arms in this study. Confounding factors such as disease progression, low haemoglobin values, comorbidities such as hypothyroidism, chronic obstructive pulmonary disease, infections and multiple concomitant medications (opiates, antihistamines, anti-hypertensive, anti-nausea) are alternative explanations for asthenic events, but no single factor can explain all cases. Common treatable causes of asthenic conditions (e.g., iron, vitamin B12 or folate deficiencies and hypothyroidism) should be investigated in patients with asthenia, fatigue or malaise.

22.3.4 Dosing Modifications and Toxicity Management

22.3.4.1 Treatment delays and discontinuation

For patients experiencing treatment delays due to toxicity that exceed 3 weeks, the Investigator should consider permanent treatment discontinuation on consultation with the National Lung Matrix Trial Office, and the patient should be observed until resolution of the toxicity (see Figure 12: AZD4547 - Dose Modifications for Toxicity and Figure 13: AZD4547 - Management of Eye Toxicity (Visual Symptoms).

In the event of a treatment interruption for reasons other than treatment related toxicity (e.g. non-cancer related surgery) lasting >3 weeks, treatment resumption will be decided on consultation with the National Lung Matrix Trial Office.

It is advised that treatment delays should not exceed 6 weeks.

22.3.4.2 Dose Reductions

Proposed dose reductions:
Dose reduction 1: 60mg BD continuously
Dose reduction 2: 40mg BD continuously

Table 18: AZD4547 - Available dose levels

Dose level	AZD4547 dose
Starting dose	80 mg BD
-1	60 mg BD
-2	40 mg BD
N/A	Discontinue trial treatment

22.3.4.3 Evaluation, Management and Treatment of Toxicities

Dose modifications for toxicity are outlined in Figure 12. Management of eye toxicity is outlined in Figure 13 (visual symptoms) and Figure 14 (no visual symptoms).

22.3.4.3.1 Ocular toxicity

Patients with uncontrolled glaucoma or intra-ocular pressure ≥ 21mm Hg at screening should be referred for ophthalmological management and the condition controlled prior to first dose. Clinically significant abnormalities detected during ophthalmic assessments should be reported as AEs. The patient should be managed under the care of a competent ophthalmologist with appropriate medication and followed up until the condition has resolved. The eye toxicity management guidelines (Figure 13 and Figure 14) differentiate the approach to be taken based on whether or not there are visual symptoms and/or involvement of the macula. For patients who report visual symptoms that are associated with RPED in the macula diagnosed by OCT, dosing with AZD4547 will be stopped for a period of up to 3 weeks. If there is complete resolution, treatment may be recommenced at a lower dose but only 1 dose reduction will be allowed. For patients who are found to have RPED via routine OCT monitoring, which is asymptomatic and outside the macula, provided that the local Investigator and ophthalmologist agree, the patient may be permitted to continue receiving AZD4547, subject to ongoing close ophthalmological observation (please refer to the management guidelines in the study protocol). In either case, if RPED progresses, the patient will permanently discontinue AZD4547.

Figure 12: AZD4547 - Dose Modifications for Toxicity

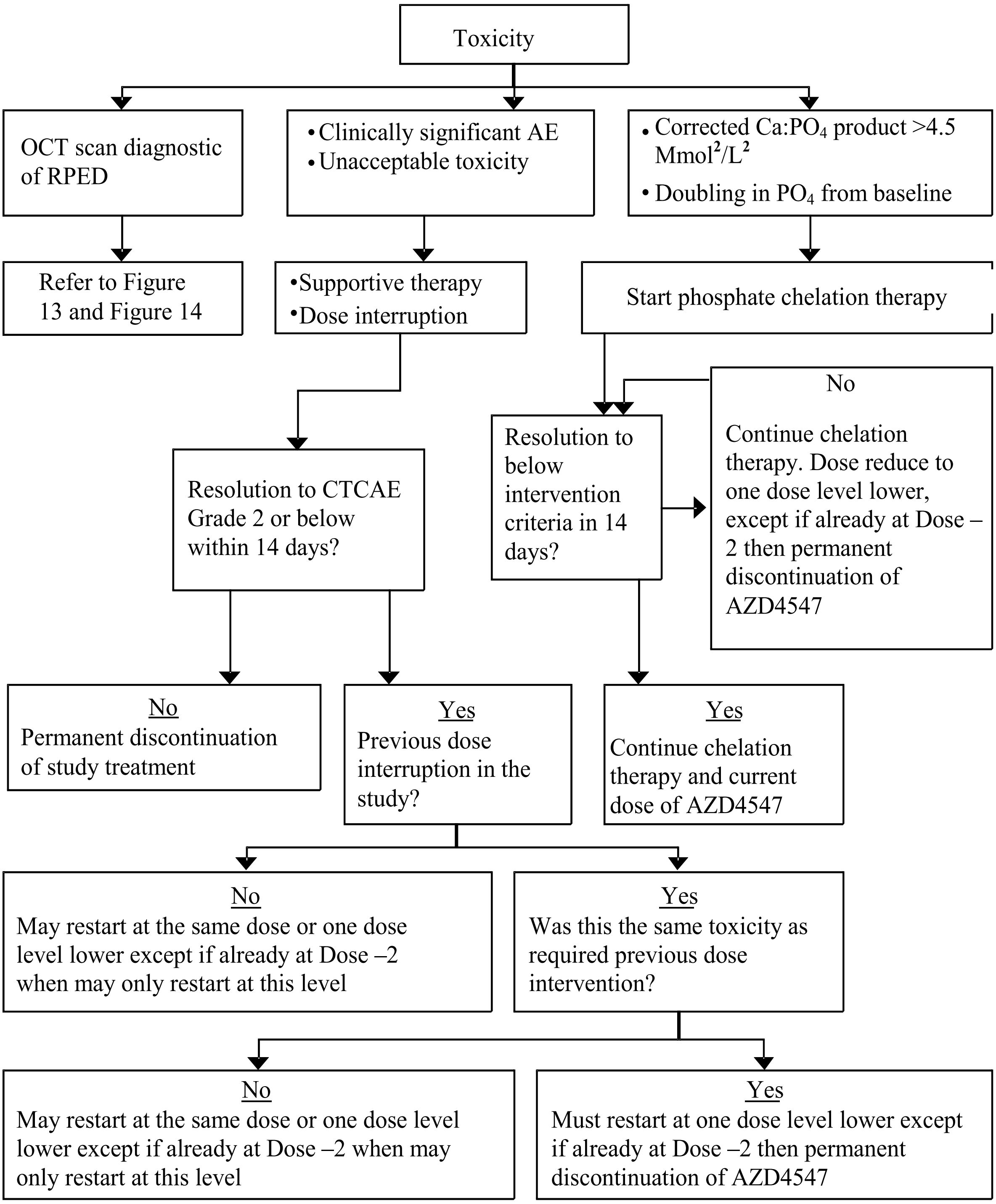

*Chelation therapy should be a non-calcium containing agent. Investigators must seek appropriate specialist medical consultation (renal or metabolic) to advise on the prescription and titration of phosphate chelation agents. Investigators should also raise patient's awareness of low phosphate diets.

Figure 13: AZD4547 - Management of Eye Toxicity (Visual Symptoms)

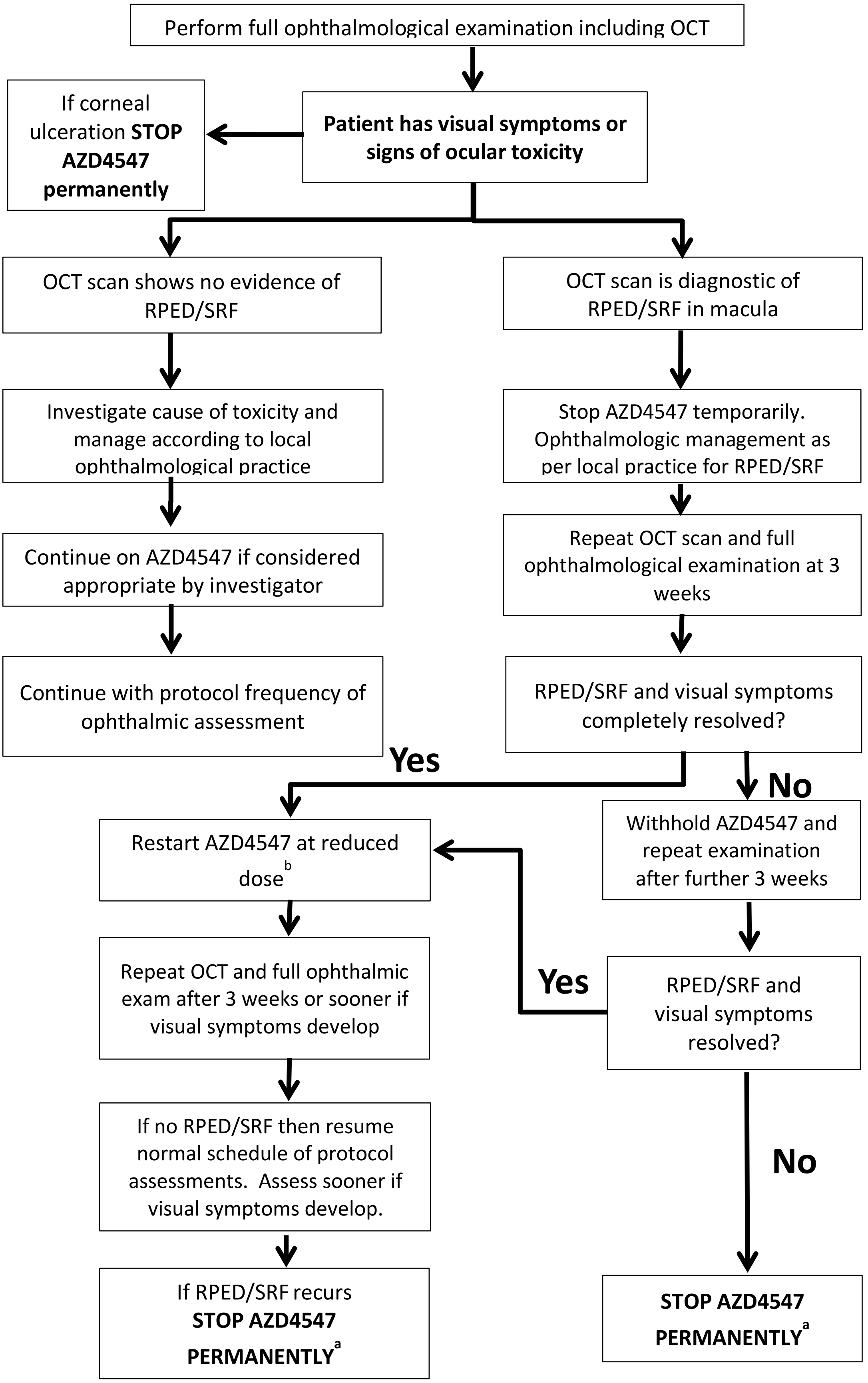

Footnotes:

a: After permanent discontinuation of AZD4547 due to ocular toxicity, patients should be managed according to local clinical practice

b: Only 1 dose reduction allowed for management of RPED or SRF.

MedDRA Medical Dictionary for Regulatory Activities; OCT Optical-coherence-tomography; RPED or SRF This grouped term includes RPED (MedDRA preferred terms of detachment of retinal pigment epithelium and detachment of macular retinal pigment epithelium), MedDRA preferred term subretinal fluid, MedDRA preferred term serous detachment, MedDRA preferred term retinal detachment (MedDRA lower level term: serous retinal detachment); SRF Subretinal fluid.

Figure 14: AZD4547 - Management of Eye Toxicity (No Visual Symptoms)

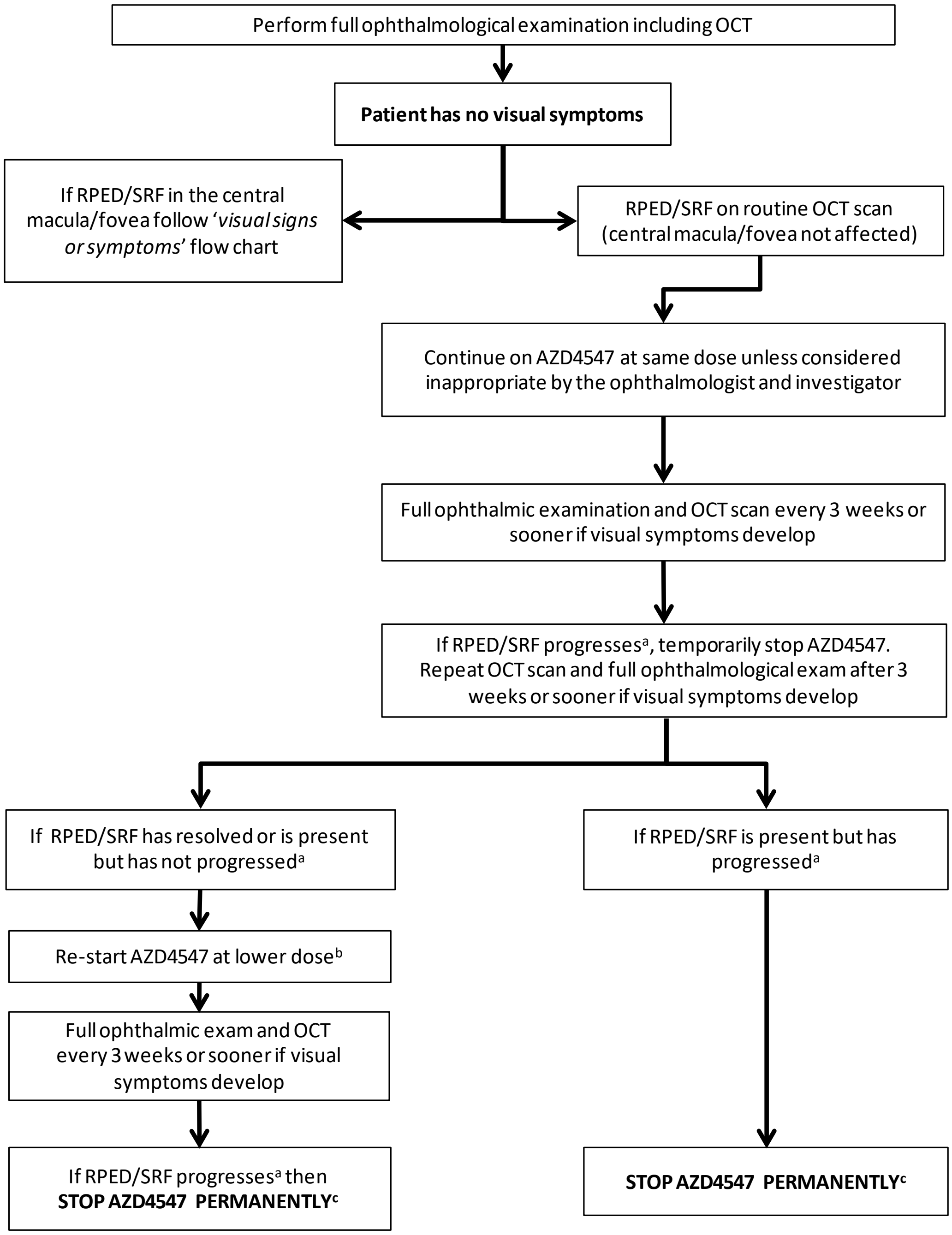

Footnotes:

a: Progression of RPED or SRF is defined as development of visual symptoms, extension from para-macular to macula, or increase in the number of lesions.

b: Only 1 dose reduction allowed for management of RPED or SRF.

c: After permanent discontinuation of AZD4547 due to ocular toxicity, patients should be managed according to local clinical practice.

MedDRA Medical Dictionary for Regulatory Activities; OCT Optical-coherence-tomography; RPED or SRF This grouped term includes RPED (MedDRA preferred terms of detachment of retinal pigment epithelium and detachment of macular retinal pigment epithelium), MedDRA preferred term subretinal fluid, MedDRA preferred term serous detachment, MedDRA preferred term retinal detachment (MedDRA lower level term: serous retinal detachment); SRF Subretinal fluid.

22.4 Reference List

- André F, Bachelot T, Campone M *et al* (2013). Targeting FGFR with dovitinib (TKI258): preclinical and clinical data in breast cancer. *Clin Cancer Res* **19(13):**3693-702.
- Bahleda R, Dienstmann R, Adamo B, Gazzah A, Infante JR, Zhong B, *et al.* (2014). Phase 1 study of JNJ-42756493, a pan-fibroblast growth factor receptor (FGFR) inhibitor, in patients with advanced solid tumors. *J Clin Oncol* **32(5s),** (suppl; abstr 2501).
- Bousquet G, Alexandre J, Le Tourneau C *et al.* (2011) Phase I study of BIBF 1120 with docetaxel and prednisone in metastatic chemo-naïve hormone-refractory prostate cancer patients. *Br J Cancer* **105(11):**1640-5.
- Brown AP, Courtney CL, King LM *et al.* (2005). Cartilage dysplasia and tissue mineralization in the rat following administration of a FGF receptor tyrosine kinase inhibitor. *Toxicologic Pathology* **33:**449-55.
- Dienstmann R, Bahleda R, Adamo B, Rodon J, Varga A, Gazzah A (2014). First in human study of JNJ-42756493, a potent pan fibroblast growth factor receptor (FGFR) inhibitor in patients with advanced solid tumors. Presented at the AACR Annual Meeting 2014 (8 April): Abstract CT325.
- du Bois A, Huober J, Stopfer P *et al.* (2010). A phase I open-label dose-escalation study of oral BIBF 1120 combined with standard paclitaxel and carboplatin in patients with advanced gynaecological malignancies. Ann Oncol **21(2):**370-5.
- Gavine PR, Mooney L, Kilgour E *et al.* (2012). AZD4547: an orally bioavailable, potent, and selective inhibitor of the fibroblast growth factor receptor tyrosine kinase family. *Cancer Res* **72(8):**2045-56.
- Kim S, Ahn JH, Ro J *et al.* (2004). Randomized Phase II Multicenter Trial of Oral Antiangiogenic Agent TSU-68 in Combination with Docetaxel versus Docetaxel. *Eur J Cancer* **47:S347** (suppl.1).
- Lawrence DP, Kupelnick B, Miller K *et al.* (2004). Evidence report on the occurrence, assessment, and treatment of fatigue in cancer patients. *J Natl Cancer Inst Monogr.* **32:**40-50.
- Liao RG, Jung J, Tchaicha J *et al.* (2013). Inhibitor-sensitive FGFR2 and FGFR3 mutations in lung squamous cell carcinoma. *Cancer Res* **73(16):**5195-205.
- Okamoto I, Yoshioka H, Takeda K *et al.* (2012) Phase I clinical study of the angiogenesis inhibitor TSU-68 combined with carboplatin and paclitaxel in chemotherapy-naive patients with advanced non-small cell lung cancer. *J Thorac Oncol* **7(2):**427-33.
- Razzaque MS, Lanske B (2007). The emerging role of the fibroblast growth factor-23-klotho axis in renal regulation of phosphate homeostasis. J Endocrinol **194:**1-10.
- Sequist LV, Cassier P, Varga A, Tabernero J, Schellens JHM, Delord J, *et al.* (2014). Phase I study of BGJ398, a selective pan-FGFR inhibitor in genetically preselected advanced solid tumors. Presented at the AACR Annual Meeting 2014 (8 April): Abstract CT326.
- Shin SJ, Jung M, Jeung HC *et al.* (2012). A phase I pharmacokinetic study of TSU-68 (a multiple tyrosine kinase inhibitor of VEGFR-2, FGF and PDFG) in combination with S-1 and oxaliplatin in metastatic colorectal cancer patients previously treated with chemotherapy. Invest New Drugs **30(4):**1501-10.
- Tomlinson E, Fu L, John L *et al.* (2002). Transgenic mice expressing human fibroblast growth factor-19 display increased metabolic rate and decreased adiposity. *Endocrinol* **143(5):**1741-7.
- White KE, Cabral JM, Davis SI *et al.* (2005). Mutations that cause osteoglophonic dysplasia define novel roles for FGFR1 in bone elongation. *Am J Hum Genet* **76:**361-7.

23 ARM B: VISTUSERTIB – MTORC1/2 INHIBITOR *(CLOSED TO RECRUITMENT)*

Lead Investigator: Professor Gary Middleton

23.1 Background & Rationale

23.1.1 Molecular Cohorts

Inhibitor: Vistusertib (formerly called AZD2014)

Arm	Investigational Medicinal Product	Cohort Number	NSCLC Histology	Molecular Cohort
B	Vistusertib – MTORC1/2 Inhibitor	B1	NSCLC	TSC1 or TSC2 mutation
		B2	NSCLC	STK11/LKB1 mutation or STK11/LKB1 homozygous deletion:<br>a. Patients with no concomitant KRAS mutation (CLOSED TO RECRUITMENT); OR<br>b. Patients with a concomitant KRAS mutation.

Vistusertib is an adenosine triphosphate (ATP)-competitive, selective mTOR kinase inhibitor targeting both mTORC1 (rapamycin-sensitive) and mTORC2 (rapamycin insensitive) complexes. Vistusertib is molecularly different from rapalogues and achieves more profound mTORC1 inhibition, in particular inhibiting phosphorylation of the rapamycin insensitive site on 4E-binding protein 1 (4E-BP1) (T37/46). Vistusertib also inhibits mTORC2 and has a broader range of growth inhibitory activity *in vitro* across tumour types compared to rapalogues. As such, dual TORC1/TORC2 inhibitors like vistusertib that inhibit both mTOR complexes may offer therapeutic advantages to rapalogues.

23.1.2 Pre-Clinical Rationale

Vistusertib is a specific inhibitor of mTORC1 and mTORC2 in enzyme assays and inhibits the phosphorylation of AKT substrates in cells (full details of the pre-clinical assessment of vistusertib are available in the IB – this has been partly presented by Guichard *et al.* 2012). Vistusertib inhibits the proliferation of a range of cell lines derived from solid and haematological tumours. Vistusertib shows dose dependent pharmacodynamic (PD) and antitumour activity in xenografts at well-tolerated doses. Studies *in vitro* show vistusertib to be a potent inhibitor of mTOR (IC_{50} value of 2.81 nM using a truncated FLAG-tagged mTOR alpha screen assay). In a counter screen against 220 other kinases, vistusertib was inactive, including PI3Kα-δ using recombinant PI3 kinases with the lipid phosphatidylinositol bisphosphate as substrate. The IC50 for the different PI3Ks tested were all greater than 3 μM indicating at least a 1000-fold selectivity.

In cell lines, vistusertib inhibited downstream targets of both mTORC1 (phosphorylation of pS6 at serine 235/236 and serine 240/244; phosphorylation of 4EBP1 at tyrosine 37/46 and serine 65) and mTORC2 (phosphorylation of AKT at serine 473) in a dose- and time-dependent manner. In a panel of ER-positive breast cancer cell lines, vistusertib induced a concentration-dependent, sustained decrease of pAKT (S473), pS6 (S240/244 and S235/236) and p-4EBP1 (S65 and T37/46), confirming substantial activity against mTORC1 and mTORC2 downstream

targets. In contrast, RAD001 potently inhibited S6 phosphorylation, but had only a modest effect on the phosphorylation of 4EBP1 at S65 (normally rapamycin-sensitive) and no apparent activity against the phosphorylation of 4EBP1 at T37/46 (rapamycin-resistant). The fact that vistusertib more potently inhibited 4EBP1 phosphorylation than RAD001 was reflected in the observation that vistusertib blocked more efficiently translation initiation and had a greater impact on protein expression of Cap-dependent genes such as Mcl-1, c-Myc and cyclin D1. Finally, RAD001 increased pAKT S473 and pAKT T308 suggesting that the feedback loop between S6K and insulin receptor substrate (IRS1) is functional in these cells, whereas it was effectively inhibited with vistusertib due to its mTORC2 activity.

Both, vistusertib and rapamycin inhibited the proliferation of the majority of cell lines within a large cell panel of solid tumour and haematological cell lines, including 26 lung cancer cell lines, in a dose-dependent manner. Fifty percent growth inhibition (GI_{50}) was observed at sub-micromolar concentrations in 72 out of 82 cancer cell lines with vistusertib. In similar conditions, GI_{50} lower than 1 μM were observed in only 38 out of 83 cell lines with rapamycin. In these studies, vistusertib induced more complete growth inhibition than rapamycin.

23.1.3 Clinical Data

At the data cut-off of 5th October 2015, 547 patients have been enrolled in studies with vistusertib; 252 in AZ sponsored studies, and 213 in externally sponsored research (ESR) studies. A total of 465 have received vistusertib.

Two AstraZeneca-sponsored Phase 1 studies were designed to assess the safety, tolerability, pharmacokinetics and preliminary efficacy of vistusertib; D2270C00001 (monotherapy in patients with advanced cancer); and D2270C00005 (in combination with fulvestrant in patients with metastatic breast cancer). Study D2270C00001 is now complete and Clinical Study Report is available, whilst study D2270C00005 is ongoing.

In addition, vistusertib has been administered as monotherapy to Japanese patients with advanced solid malignancies in Study D2270C00005 (Japan PK) and to patients with relapsed or refractory squamous non-small cell lung cancer (sqNSCLC) after at least one line of therapy in combination with paclitaxel in Study D2274C00001 (STORK).

Several doses and dosing schedules of vistusertib have been explored in both the monotherapy (D2270C00001, see Table 19) and combination (D2270C00005, see Table 20) studies:

Table 19: Vistusertib Monotherapy (D2270C00001)

Dosing schedule	Doses explored	Defined MTD
Intermittent weekly BD dosing (2 consecutive days, 2 days on, 5 days off)	100 mg, 125 mg 170 mg, 225 mg	125 mg (2 consecutive days, 2 days on, 5 days off)
Continuous BD dosing	25 mg, 50 mg, 70 mg, 100 mg	50 mg
Continuous QD dosing	75 mg, 100 mg, 125 mg, 175 mg	100 mg

BD- twice daily dosing, QD- once daily dosing, MTD- maximum tolerated dose.

Table 20: Vistusertib in Combination with fulvestrant (D2270C00005)

Dosing schedule	Doses explored	Recommended dose/ defined MTD
Intermittent BD dosing (2 consecutive days, 2 days on 5 days off)	125 mg, 170 mg	125 mg (2 consecutive days, 2 days on 5 days off), under fasting and fed conditions
Continuous BD dosing	35 mg, 50 mg	50 mg
Continuous QD dosing	75 mg, 100 mg	75 mg

BD- twice daily dosing, QD- once daily dosing, MTD- maximum tolerated dose

Vistusertib is available for administration as a tablet for use in clinical studies and is presented as three strengths: 10, 25 or 50 mg. The intermittent dosing schedule (either 2 consecutive days, 2 days on, 5 days off or 3 consecutive days, 3 days on, 4 days off) has been explored and has shown an improved safety profile compared to the continuous dosing schedule and as a result, the intermittent schedule will now be prioritised for use in all new vistusertib studies although the continuous dosing will still be explored in some new vistusertib single agent or combination studies. Details of all vistusertib studies, including 10 ESCRs, are summarised in Tables 2 and 4 in Section 5 of the IB edition 6.

23.1.3.1 Efficacy

Note that vistusertib remains under investigation in multiple clinical studies.

In the monotherapy study (D2270C00001) at the 50 mg BD continuous dose in a total of 40 patients, two patients had an objective response; 1 patient with pancreatic acinar cell type cancer and 1 patient with ER+ breast cancer had confirmed partial responses (PR) according to RECIST and received treatment for 175 days and 206 days, respectively. In addition, 12 patients in the 50 mg BD group, and 4 patients each in the 100 mg QD and 125 mg intermittent cohort achieved stable disease.

In the combination study with fulvestrant (study D2270C00005) information on the best objective responses in all patients and patients with measurable disease at baseline, is included in Table 20 in Section 5 of the IB edition 6. Based on data from 5th October 2015, vistusertib in combination with fulvestrant has demonstrated encouraging response rate data in this Phase I setting at MTDs for both the continuous and intermittent dosing schedules, there were partial responses (confirmed and unconfirmed) at every MTD dose group, 3/11 (27.3%) with a confirmed response rate of 2/11 (18.2%) in the 50 mg BD continuous dosing group, 3/13 (23.1%) with a confirmed response rate of 2/13 (15.4%) in the 75 mg QD continuous dosing group, and 3/20 (15.0%) with a confirmed response rate of 1/20 (5.0%) in the 125 mg BD intermittent dosing group.

23.1.3.2 Safety

The overall safety findings at vistusertib MTDs in Phase 1 trials in monotherapy and in combination with fulvestrant were very similar and the main AEs observed are consistent with AEs already reported for other mTOR inhibitors: rash, pruritus, mucositis, fatigue, nausea, vomiting, diarrhoea, constipation and decreased appetite. However, at intermittent dosing schedules, vistusertib showed a different safety profile compared to continuous dosing schedules. At intermittent dosing schedules (125 mg BD 2 days on and 5 days off) the main AEs reported, which also led to dose discontinuation in 1 patient, were nausea and vomiting. There was also a marked reduction in rash at intermittent dosing schedules (from 69.2% to 16.2% in combination and 48.8% to 15.4% in monotherapy), pruritus (61.5% to 13.5% in

combination and 34.1% to 0% in monotherapy) and mucositis (from 69.2% to 29.7% in combination therapy and 43.9% to 30.8% in monotherapy). Interestingly, there were no cases of pneumonitis observed with this intermittent monotherapy schedule (compared with 2.4% and 7.7% in the continuous monotherapy and combination therapies respectively). Thus, the intermittent dosing schedules, showing similar efficacy at least in combination with fulvestrant offer an important new development option for a mTOR kinase inhibitor with a different safety profile, especially for combinations with other agents.

23.1.3.2.1 Monotherapy

In Study D2270C00001, 86.7% of patients experienced at least 1 adverse event (AE) considered related to vistusertib by the reporting investigator. The most common AEs related to study treatment (occurring in ≥15 % patients overall across all cohorts) were fatigue (58.5 %), nausea (48.1 %), mucositis (29.6 %), diarrhoea (28.1 %), rash (27.4 %), decreased appetite (22.2 %), vomiting (21.5 %) and hyperglycaemia (15.6 %). Dose limiting toxicities (DLTs) reported at non-tolerated doses were fatigue, diarrhoea, mucositis, nausea, vomiting and rash. During the study, 55/135 (40.7%) patients had an SAE, and 24/135 (17.8%) had a treatment-related SAE. One patient died due to an AE of pulmonary embolism during the study; this was deemed unrelated to treatment by the Investigator. Twenty-six (19.3%) patients experienced an AE leading to the permanent discontinuation of vistusertib. 26 patients (19.3 %) reported at least 1 AE leading to discontinuation. The most common AEs leading to discontinuation of vistusertib (reported in ≥4 patients overall) were fatigue (7.4 %), nausea (4.4 %), decreased appetite (3.0 %), diarrhoea (3.0 %), mucositis (3.0 %), rash (3.0 %), and vomiting (3.0 %).

Although most patients experienced an AE, the drug was considered to be well tolerated with easily manageable side effects by the investigators. A total of 68 patients (50.4 %) experienced an AE of Common Terminology Criteria for Adverse Events (CTCAE) grade 3, 1 patient (1.3 %) had a CTCAE grade 4 AE, and 1 patient (1.3 %) had a CTCAE grade 5 AE (incidence rates include DLTs at non-tolerated doses). 15 (12 %) patients on doses below the MTD experienced AEs that led to the discontinuation of vistusertib and no patients on a dose below the MTD for the QD continuous and intermittent (2 consecutive days, 2 days on, 5 days off) dosing schedules reported any AEs which led to discontinuation. AEs that defined the drug's non-tolerability were generally reversible within a week of stopping treatment with vistusertib. 105 patients (77.8 %) had a duration of treatment (including periods of dose interruption) of >3 months. 1 patient had duration of treatment of 18 months.

The change from the continuous dosing at a dose of 50 mg BD to the intermittent (2 consecutive days, 2 days on, 5 days off) dosing of vistusertib at a dose of 125 mg BD was accompanied by an increase in the incidence of nausea (from 20/41 [48.8 %] to 9/13 [69.2 %]) and diarrhoea (from 18/41 [44.0 %] to 9/13 [69.2 %]), however, it reduced the incidence of rash (from 20/41 [48.8 %] to 2/13 [15.4 %]) and mucositis (from 18/41 [43.9 %] to 4/13 [30.8 %]) . The 125 mg 2 consecutive days, 2 days on, 5 days off schedule did not have any AE's associated with discontinuation with vistusertib, compared to the continuous 50 mg BD schedule, for which 18/41(43.9 %) patients discontinued treatment as a result of an AE.

T wave changes on the ECG have been observed in 9 selected patients of the total 135 included in the study 1: 4 patients had AE of T wave inversion reported by Investigator. Additional 5 patients' ECG were assessed by GSP/MSD as potentially significant as the T wave changes were persistent on treatment and did not resolve at end of study. However, the above mentioned ECG findings do not indicate a clinically significant ECG abnormality when the pre-clinical CV safety profile of the study compound and the clinical context of the observed ECG changes are taken into the final clinical consideration.

23.1.3.2.2 Combination with fulvestrant

At the Study D2770C00005, MTDs overall, the most frequently reported AEs related to vistusertib (reported in ≥15% patients overall) were fatigue, nausea, rash, diarrhoea, decreased appetite, vomiting, hyperglycaemia, mucositis, pruritus, headache, anaemia, asthenia, dry skin, constipation, dizziness and skin hyperpigmentation. Due to low numbers in Study D2270C00005 50 mg BD continuous dosing with fulvestrant group, headache, asthenia, constipation, dizziness and skin hyperpigmentation are included in this list despite only affecting 2 patients (2/13, 15.4% of the group).

The most frequently reported AEs of CTCAE Grade 3 or higher (reported in >1 patient at an MTD, irrespective of causality) were rash, fatigue, nausea, infections, mucositis, vomiting, anaemia, diarrhoea, hyperglycaemia, and hypophosphataemia.

At the Study D2270C00005 (Study 5, combination with fulvestrant) MTD doses, no SAE MedDRA term was reported in more than 1 patient. The SAEs in the continuous dosing groups of 50 mg BD plus fulvestrant and 75 mg QD plus fulvestrant were pulmonary embolism, infections, neutropenia, renal impairment, and spinal compression fracture. The SAEs in the intermittent dosing group of 125 mg BD plus fulvestrant were vomiting, nausea, hypercalcaemia, diarrhoea, infections, colitis, enteritis and subdural haematoma. Two patients had causally-related SAEs: 1 patient (75 mg QD) had CTCAE Grade 4 febrile neutropenia; and 1 patient (125 mg BD) had CTCAE Grade 3 nausea, Grade 3 vomiting, and Grade 3 diarrhoea. All these SAEs were considered by the investigator to be related to vistusertib but not fulvestrant.

There was one death due to an AE in the Study D2270C00005 (Study 5, combination with fulvestrant) MTD of 75 mg QD + fulvestrant (continuous dosing). This was an AE of sepsis of unknown aetiology which was deemed unrelated to treatment by the Investigator.

In the PASTOR study, the most common causally related AEs (occurring in >40% patients) were neutropenia 64.8%, fatigue 51.9% and nausea 44.4%. AEs occurring in >10% of patients that were causally related to vistusertib (regardless of whether also causally related to fulvestrant, but not causally related to palbociclib) were diarrhoea (14.8%, vomiting 13.0% and nausea 11.1%). The most common events (reported in ≥20% patients overall) were neutropenia (64.8%), fatigue (61.1%), nausea (46.3%), diarrhoea (42.6%), rash (31.5%), anaemia (27.8%), cough (24.1%), vomiting (24.1%), back pain (20.4%) mucosal inflammation (20.4%]), and urinary tract infection (20.4%). Of interest, 2 cases of interstitial lung disease were reported: 1 patient with pneumonitis and 1 with lung infiltration. The most common causally related AEs (occurring in >40% patients) were neutropenia (64.8%), fatigue (51.9%) and nausea (44.4%). AEs occurring in >10% of patients that were causally related to vistusertib (regardless of whether also causally related to fulvestrant, but not causally related to palbociclib) were diarrhoea (14.8%), vomiting (13.0%) and nausea (11.1%).
At the data cut-off (30 March 2018), 10 patients experienced 14 SAEs on study; all SAEs affected 1 patient only apart from febrile neutropenia (reported in 3/54 [5.6%] patients). Overall, 2 patients experienced SAEs that were considered to be causally related to vistusertib: 1 patient with febrile neutropenia, and 1 patient with febrile neutropenia and rash. The 2 events of febrile neutropenia were determined to be related to vistusertib and palbociclib, and 1 event of rash was determined to be related to vistusertib. Eleven patients died on study. All patient deaths were due to disease progression. There were no treatment-related deaths in this study. Two patients overall had AEs leading to discontinuation of vistusertib; Grade 3 rash (1.9%) and Grade 2 vitreous haemorrhage (1.9%].

Based on these preliminary unvalidated data, the most commonly occurring adverse events (AEs) are in keeping with those expected in patients enrolled in Phase 1 oncology studies or in patients receiving rapamycin analogues. A total of 65/211 (30.8%) patients had at least 1

SAE in both the monotherapy and combination studies, of the patients who had an SAE, the majority (39/65, 60%) had an SAE that was considered by the Investigator to be unrelated to vistusertib treatment. The introduction of intermittent dosing of vistusertib at a dose of 125 mg BD was accompanied by a change in the safety profile of the drug particularly reducing the incidence of rash, hyperglycaemia and mucositis. This change led to a reduction in the number of patients permanently discontinuing treatment when compared with continuous dosing schedules. Both dosing schedules of 50 mg BD continuous and 125 mg BD intermittent (2 days on, 5 days off) have an acceptable safety profile for further investigation in the clinical programme for vistusertib. Preliminary unvalidated data suggest that the overall safety profile of vistusertib in combination with fulvestrant is broadly similar to the known individual safety profile of vistusertib monotherapy.

23.1.3.3 Pharmacokinetics

The key findings from the preliminary single-dose and multiple dose pharmacokinetic (PK) data are summarised below (for details and discussion see IB section 5.1):

The pharmacokinetics of vistusertib have been studied following single and repeat administration of a solution and tablet formulation in cancer patients. QD, BD and intermittent dosing (2 consecutive days, 2 days on, 5 days off) have been investigated. In summary, vistusertib is orally available and rapidly absorbed, when administered as a tablet and has a short termination half-life. There is a greater than dose proportional increase in exposure (AUC) to vistusertib across the dose range investigated with a corresponding increase in terminal half-life. The mechanism for this and clinical relevance has not been fully elucidated.

Absorption of vistusertib is delayed following administration with food (delayed Tmax and reduction in Cmax) relative to the fasted state, but the extent of exposure (AUC) appears comparable.

There is no change in exposure to vistusertib or fulvestrant when both are administered concomitantly.

23.1.3.4 Pharmacodynamics

At a dose of 50 mg BD, vistusertib reduced cytoplasmic pS6 (S235/236) IHC staining in 8 out of 8 tumour biopsies obtained after 1 to 5 hours of therapy indicating that the drug has mTORC1 activity. Phosphorylation of 4E-BP1 (T37/46) was decreased post treatment in 3 out of 7 evaluable paired biopsies obtained after 1 to 5 hours of therapy.

In monocytes, the median % change in p4E-BP1 at 2 hours after a single dose of vistusertib was -45.7% (n = 19 range -96.9% to +37.3%) at 50 mg BD and -48.3% (n = 2 range -87.7% to -8.9%) at 125 mg intermittent weekly dosing schedule (2 days on, 5 days off). By 6 to 8 hours, the median % change in p4E-BP1 was - 45.9% (n = 18, range -93.9% to +32.7%) at 50 mg BD and -11.7% (n = 2, range -50.1% to +26.8%) at 125 mg intermittent weekly dosing schedule in D2270C00001. Phosphorylation of 4E-BP1 was also reduced following treatment at the lower dose of 100 mg on the intermittent weekly dosing schedule in study D2270C00001.

Phosphorylation of AKT (S473) was inhibited in platelet rich plasma (PRP) providing evidence for TORC2 inhibition in surrogate tissue. At 2 hours after a single dose of vistusertib the median % change was -62.5% (n = 43 range -98.7% to +13.5%) at 50 mg BD and -63.4% (n = 5 range -92.1% to -7.0%) at 125 mg intermittent weekly dosing schedule in D2270C00001. By 6 to 8 hours, the median % change was -43.6% (n = 45 range –93.1% to +62.7%) at 50 mg BD and -34.7% (n = 5 range -83.1% to +7.4%) at 125 mg intermittent weekly dosing schedule. Phosphorylation of AKT was also reduced following treatment at the lower doses of 25 mg BD, 35 mg BD and 100 mg on the intermittent weekly dosing schedule.

There was no evidence for an elevation in cytoplasmic pAKT (S473) (mTORC2 phosphorylation site) in tumour biopsies indicating that vistusertib may be differentiated from everolimus which has been reported to increase phosphorylation on this site in approximately 50% of cases. In tumour biopsies, phosphorylation of AKT (S473) was lower in the post-treatment biopsies in 3 out of 4 evaluable samples obtained after 1 to 5 hours of therapy. A lower level of pAKT (T308) was also observed after treatment in 3 out of 6 tumour biopsies taken 1 to 5 hours after dosing.

23.1.3.5 Marketing Experiences

Vistusertib is not currently marketed in any territory.

23.1.4 Cohort definition

23.1.4.1 B1 - NSCLC harbouring TSC1/2 mutations

The TSC1-TSC2 heterodimeric complex is a major negative regulator of mTORC1 activity. mTORC1–induced phosphorylation of S6 Kinase-1 (S6K1) and 4E-BP1 is inhibited by TSC1-TSC2. Elevated mTORC1 activity is ubiquitous in all TSC deficient tumour cell lines. The guanosine-5'-triphosphate (GTP)ase-activating protein (GAP) domain of TSC2 in the complex stimulates the GTPase activity of Ras homolog enriched in brain (Rheb) resulting in its conversion to Rheb-GDP thus preventing its activation of mTORC1.

Both complete and haploid loss of TSC1 significantly co-operated with activating KRAS mutation on the KRAS adenocarcinoma mouse model (Liang *et al.* 2010). TSC2 protein levels were reduced in these tumours consistent with the chaperone effect of TSC1 in TSC2. pS6 levels were increased and pAKTS473 levels decreased in TSC knockout mice compared to KRAS mutant mice with intact TSC1. Crucially, whilst LSL-Kras ^{G12D} mice showed stable disease on treatment with rapamycin, LSL-Kras^{G12D}Tsc$^{L/L}$ mice showed responses to rapamycin and a striking improvement in survival. There was greater reduction in proliferation and increased apoptosis induction in LSL-Kras^{G12D}Tsc$^{L/L}$ mice compared to LSL-Kras ^{G12D} mice treated with rapamycin. These data clearly showed that TSC knockdown lung cancer was highly sensitive to treatment with rapamycin.

Most TSC mutant cell lines appear highly sensitive to vistusertib (Figure 15). All TSC mutant cell lines had a GI_{50} of <1 μM and 8/10 TSC1 mutants had a GI_{50}<0.2 μM (p=0.004) which represents high levels of activity of the drug against these cells.

Given the pivotal role of TSC in regulating mTOR activity and the pre-clinical data targeting TSC mutant lung cancer with vistusertib is clearly appropriate. The TCGA data demonstrate a TSC1 mutation rate of 1.5% in adenocarcinoma and 2.8% in squamous cell cancer and a TSC2 mutation rate of 1.8% and 2.8% respectively. Thus the TSC mutation rate in adenocarcinoma is not dissimilar to the ALK fusion rate in non-selected adenocarcinoma whilst the rate in squamous cell cancer >5%. These represent small but important cohorts.

Figure 15: Vistusertib - AZ GI50 data by TSC1 or 2 status

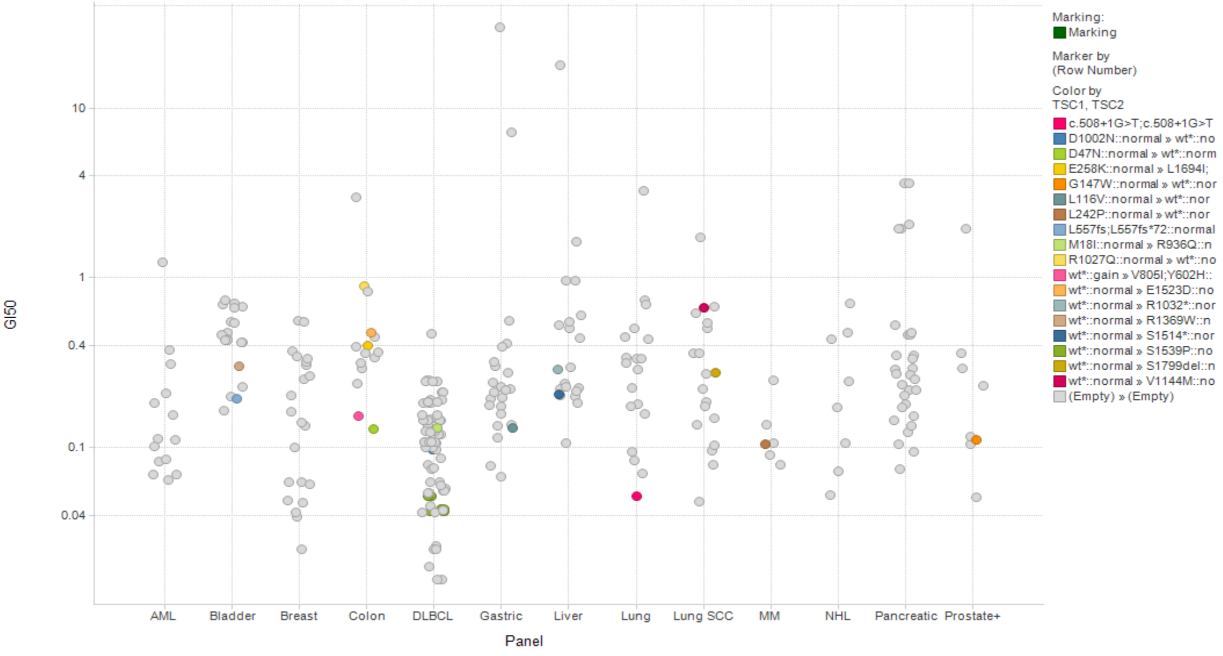

10
4
1
0.4
0.1
0.04
GI50
AML
Bladder
Breast
Colon
DLBCL
Gastric
Liver
Lung
Lung SCC
MM
NHL
Pancreatic
Prostate+
Panel
Marking:
Marking
Marker by
(Row Number)
Color by
TSC1, TSC2
c.508+1G>T;c.508+1G>T
D1002N::normal » wt*::no
D47N::normal » wt*::norm
E258K::normal » L1694I;
G147W::normal » wt*::nor
L116V::normal » wt*::nor
L242P::normal » wt*::nor
L557fs;L557fs*72::normal
M18I::normal » R936Q::n
R1027Q::normal » wt*::no
wt*::gain » V805I;Y602H::
wt*::normal » E1523D::no
wt*::normal » R1032*::nor
wt*::normal » R1369W::n
wt*::normal » S1514*::nor
wt*::normal » S1539P::no
wt*::normal » S1799del::n
wt*::normal » V1144M::no
(Empty) » (Empty)

23.1.4.2 B2 - NSCLC harbouring STK11/LKB1mutations and homozygous deletion

Lysyl oxidase (LOX) is associated with hypoxia-induced metastasis, via oxidisation of lysine residues in collagen and elastin which stabilises the extracellular matrix. High LOX expression is seen in STK11/ LKB1 mutant NSCLC cell lines but not in most wild type cells (Gao *et al.* 2010). Ectopic expression of STK11/LKB1 in STK11/LKB1 deficient lung cancer cells reduced LOX mRNA levels and STK11/LKB1 knockdown in wild type cells increased LOX message. STK11/LKB1 regulation of LOX was via repression of mTOR which resulted in decreased hypoxia inducible factor (HIF)-1α activity. Ectopic expression of HIF-1α rescued the inhibition of LOX activity upon rapamycin treatment in A549 which are STK11/LKB1 deficient. LOX knockdown in A459 cells significantly reduced anchorage-independent cell growth and migration. β-aminopropionitrile (BAPN) is a LOX inhibitor. In an STK11/LKB1 deficient mouse model BAPN significantly reduced tumour number and volume but had no effect in wild type models. It was particularly effective in reducing the number of large STK11/LKB1 deficient tumours. STK11/LKB1 deficient tumours were characterised by active extracellular matrix (ECM) remodelling with collagen-rich fibrotic foci and this increased collagen density enhanced the invasiveness of A549 cells.

Thus, STK11/LKB1 loss results in a lack of hypoxia-induced mTOR repression and mTOR-induced LOX over-expression and thus mTOR inhibition might be expected to be more efficacious in patients with STK11/LKB1-deficient tumours. Additional supporting evidence for this came in the biomarker analysis of the TAMRAD study where patients with low STK11/LKB1 protein expression obtained much more benefit from the addition of everolimus to tamoxifen than those with high STK11/LKB1 expression (Treilleux *et al.* 2013).

A third mechanism of how loss of STK11/LKB1 may promote lung tumorigenesis and progression has been recently described (Okon *et al.* 2014). Growth factor stimulation of STK11/LKB1 deficient A549 cells led to phosphorylation of a large range of receptor tyrosine kinase (RTKs) including EGFR, HER2, Met, VEGFR3, RET, FGFR and EphA2. This was abrogated by forced expression of STK11/LKB1. In STK11/LKB1 proficient lung cancer cells STK11 ablation enhanced EGFR phosphorylation upon EGF stimulation. STK11/LKB1 appeared to enhance the phosphatase activity of SHP-2 and other phosphatases which dephosphorylate activated RTKs.

There is supportive pre-clinical data using vistusertib.

Figure 16 below shows a sensitivity plot of the Sanger cell collection treated with vistusertib with potency on the y axis expressed as ln (natural log) IC_{50}. 6 out of 8 STK11/LKB1 mutant NSCLC lines had IC_{50}<1 µM (ln=0) and 3 had IC_{50} of 200 nM or less (ln=-1.6).

Figure 16: Vistusertib - Sanger data by STK11/LKB1

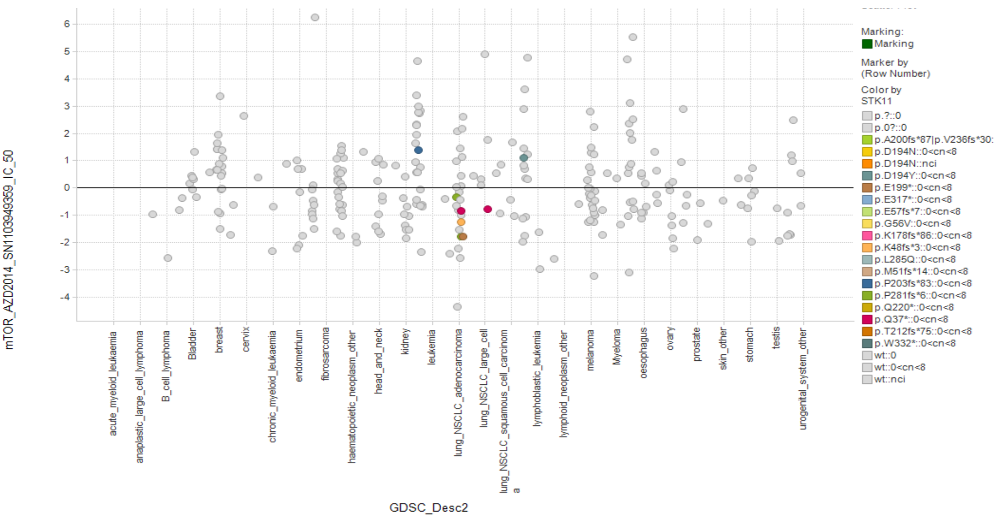

Figure 17: STK11/LKB1 NSCLC xenografts treated with AZD8055

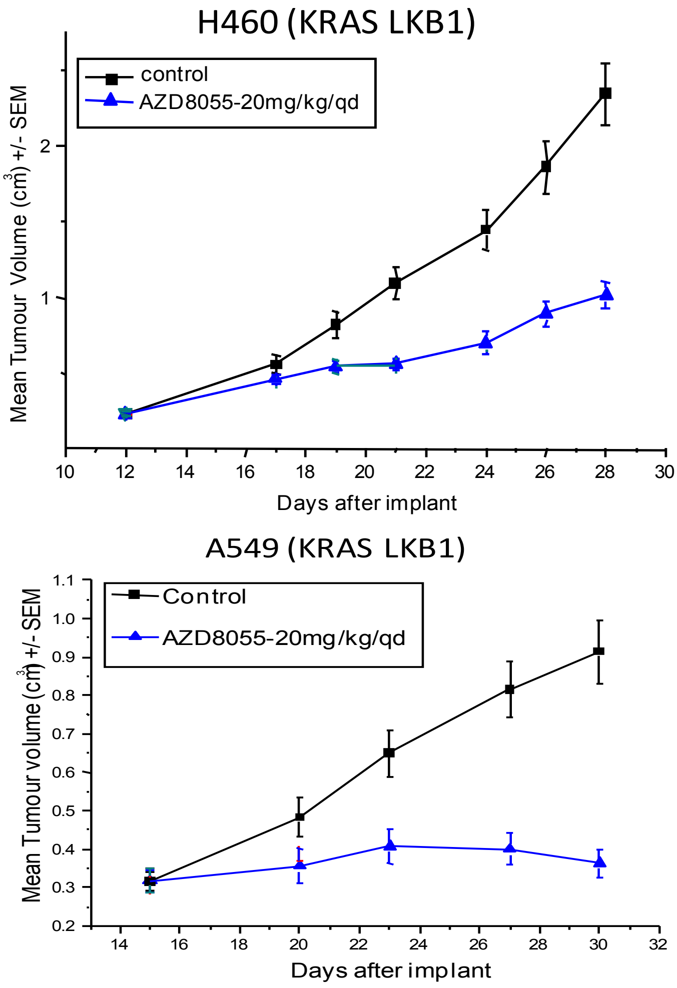

Figure 17 shows 3 separate STK11/LKB1 mutant xenograft models (H460 and A549) treated with AZD8055, an earlier dual mTORC1/2 compound with similar *in vivo* potency. All tumours showed reduction in growth upon therapy.

All STK11/LKB1 mutants harbour concomitant KRAS mutations.On 18th September 2018, the decision was made to restrict future recruitment to the B2 cohort to only patients harbouring both KRAS and STK11/LKB1 concomitant aberrations.

In a recent comprehensive analysis of STK11/LKB1 inactivating mutations in NSCLC there was a 11.3% mutation rate in tumours with loss of heterozygosity (LOH) of microsatellite markers flanking STK11/LKB1 (Gill *et al.* 2011). This is very similar to the mutation rate in adenocarcinoma in the latest iteration of the TCGA. The mutation rate in squamous cell cancer is much lower at 1.7%. A further 28% had homozygous deletion when analysed by CISH for an overall inactivation rate of 39%. The higher rate of mutation in adenocarcinomas has been reported in other series which also showed that the frequency of mutation is higher in Caucasian than oriental populations (17% vs 5% respectively) (Koivunen *et al.* 2008).

23.2 Specific Eligibility Criteria

23.2.1 Inclusion Criteria

- Patients must fulfil all the core eligibility criteria.
- Technology hub result (or locally obtained result from an approved Laboratory if applicable.
- Eastern Cooperative Oncology Group (ECOG) Performance Status ≤2 with no deterioration over the previous 2 weeks (see Appendix 8: Eastern Cooperative Oncology Group Performance Status Criteria).
- For patients with EGFRwt tumours, prior therapy must include platinum-based chemotherapy. For patients with tumours harbouring an EGFR mutation known to be associated with EGFR TKI sensitivity, prior therapy must include an EGFR tyrosine kinase inhibitor. For patients with ALK-positive tumours, prior therapy must include an ALK inhibitor.
- Ability to swallow oral medication.
- No history of non-autologous bone marrow transplant.

23.2.2 Exclusion Criteria

Patients must not enter the trial if any of the following exclusion criteria are fulfilled:

- Patients who do not fulfil all the core eligibility criteria.
- Treatment with any of the following:
    - Concomitant use of medicines known to prolong QT interval within 6 half-lives plus 1 day prior to the first dose of vistusertib (see Section 23.2.3.1).
    - Exposure to strong or moderate inhibitors or inducers of CYP3A4/5, Pgp (MDR1) and BCRP if taken within the stated washout periods prior to the first dose of vistusertib (see Section 23.2.3.2 & Table 21).
    - Exposure to sensitive or narrow therapeutic range substrates of the drug transporters OATP1B1, OATP1B3, MATE1 and MATE2K within the appropriate wash-out period (a minimum of 5 x reported elimination half-life) prior to the first dose of vistusertib (see Section 23.2.3.2 &
    - Table 22).

- Any haemopoietic growth factors (e.g., filgrastim [granulocyte colony-stimulating factor; G-CSF], sargramostim [granulocyte-macrophage colony-stimulating factor; GM-CSF]) within 2 weeks prior to the first dose of vistusertib.
  - Previous treatment with any mTOR inhibitor.
  - Prior use of an investigational monoclonal antibody therapy within 3 months of the first dose of vistusertib.
  - Radical radiotherapy within 4 weeks prior to the first dose of vistusertib, or palliative radiotherapy to focal sites within 7 days of treatment. Patients must have recovered from acute toxicity due to radiotherapy.
  - Any other chemotherapy, investigational agents or other anti-cancer therapy within 4 weeks prior to the first dose of vistusertib.
- Recent history (past 12 months) of drug abuse or alcohol abuse, as judged by the local Investigator.
- Hepatic function (in patients **with** liver metastases).
  - Alanine transferase (ALT) and Aspartate transferase (AST) $\leq$5 x ULN.
- As judged by the local Investigator, any evidence of severe or uncontrolled systemic diseases (e.g., severe hepatic impairment, interstitial lung disease [bilateral, diffuse, parenchymal lung disease], uncontrolled chronic renal diseases (glomerulonephritis, nephritic syndrome, Fanconi Syndrome or Renal tubular acidosis), or current unstable or uncompensated respiratory or cardiac conditions, or uncontrolled hypertension, active bleeding diatheses or active infection including hepatitis B, hepatitis C, and human immunodeficiency virus. Screening for chronic conditions is not required.
- Patients who have experienced any of the following procedures or conditions currently or in the preceding 6 months:
  - Coronary artery bypass graft
  - Angioplasty
  - Vascular stent
  - Myocardial infarction
  - Angina pectoris
  - Congestive heart failure New York Heart Association (NYHA) Grade ≥2
  - Ventricular arrhythmias requiring continuous therapy (see Appendix 10: New York Heart Association Classification – Stages of Heart Failure)
  - Supraventricular arrhythmias including atrial fibrillation, which are uncontrolled
  - Haemorrhagic or thrombotic stroke, including transient ischaemic attacks or any other central nervous system bleeding.
- Abnormal ECHO or MUGA at baseline (LVEF <50% and shortening fraction <15%). Appropriate correction to be used if a MUGA is performed.
- Mean resting QT interval (QTcF) >470 msec as per local reading obtained from 3 ECGs.
- Family or personal history of long or short QT syndrome, Brugada syndrome or known history of QTc prolongation or Torsades de Pointes within 12 months of registration.
- Laboratory values as listed below:
  - PT/INR >1.5 x ULN and PTT (aPTT) >1.5 x ULN
  - Reproducible proteinuria > 1+ on dipstick testing (if 2+ seen on first test, re-test at least 24 hours later)
- Pre-existing renal disease including glomerulonephritis, nephritic syndrome, Fanconi Syndrome or renal tubular acidosis.
- Patients with uncontrolled Diabetes Type 1 or Type 2 as judged by the local Investigator.
- Current refractory nausea and vomiting, malabsorption syndrome, disease significantly affecting gastrointestinal function, resection of the stomach or small bowel, symptomatic inflammatory bowel disease or ulcerative colitis, or partial or complete bowel obstruction.

- History of hypersensitivity to active or inactive excipients of vistusertib or drugs with a similar chemical structure or class to vistusertib.
- Vaccinated with live, attenuated vaccines within 4 weeks of the first dose of vistusertib.

23.2.3 Restrictions & Concomitant Medications

Information on any treatment from the date of informed consent until 28 days after the administration of the last treatment dose should be recorded. If medically feasible, patients taking regular medication should be maintained on it throughout the study period. Patient should be advised to inform their treating physicians of all concomitant medications, including prescription medicines, over-the-counter drugs, vitamins, and herbal products.

Nb. These lists are not exhaustive and the absence of a drug from the lists does not imply that its combination with vistusertib is safe.

23.2.3.1 QT Interval Prolongation

Concomitant medications **known to prolong the QT interval** are prohibited during the active treatment phase. Any patients taking such drugs at or prior to registration should discontinue the drug 6 half-lives plus 1 day prior to commencing trial treatment.

For a list of drugs known to prolong the QT interval, (or with any risk of prolonging the QT interval), please refer to the following database: https://www.crediblemeds.org. Appendix 9 Credible Meds List of Drugs that Prolong QT Interval contains a list exported from this database on 2nd March 2018. **It is important to note that this list is a guide – the database will change with time and therefore needs to be checked in real-time when screening and registering a patient, and throughout their treatment. The Trials Office will email all sites when updates are made to the database.**

Please note that for AstraZeneca trial Arms, only concomitant medications listed on https://www.crediblemeds.org under the category '**Known risk of TdP'** will exclude patients from entering the trial as per the eligibility criteria.

It is the responsibility of an Investigator (Consultant level) to review and clinically evaluate all concomitant medications. Please contact the National Lung Matrix Trial Office for clarification regarding any drugs that appear on the database that are not listed in Appendix 9 Credible Meds List of Drugs that Prolong QT Interval0.

The drugs listed on this website are taken from information provided by The Arizona Centre for Education and Research on Therapeutics and The Critical Path Institute, Tucson, Arizona and Rockville, Maryland. Important Note - If a patient is being treated with such medication or is taking another medication that may affect QT interval which is not on the database, please contact the National Lung Matrix Trial Office to obtain the recommended withdrawal/ minimum period prior to starting trial treatment.

23.2.3.2 Cytochrome P450 (CYP) Related Inhibition and Induction

CYP3A5 and CYP3A4 have been identified *in vitro* as the principal P450s responsible for human metabolism of vistusertib. Vistusertib has also been identified *in vitro* as a substrate for the drug transporters Pgp (MDR1) and BCRP. Co-administration of CYP3A4/5, Pgp (MDR1) or BCRP inhibitors may increase exposure to vistusertib. In addition, co-administration of CYP3A4/5, Pgp (MDR1) or BCRP inducers may decrease the exposure to vistusertib.

Co-medications which are moderate or strong inhibitors or inducers of CYP3A4/5, Pgp (MDR1) and BCRP, or specific substrates of the drug transporters OATP1B1, OATP1B3, MATE1 and

MATE2K are excluded whilst receiving treatment with Vistusertib*, and not permitted within the appropriate wash-out periods before the first dose of study treatment. Listings of such co-medications are given in the tables below.

*Whilst patients may not enter this trial arm if they have taken within the stated washout periods prior to trial start any of the CYP3A4/5, Pgp (MDR1) or BCRP inhibitors or inducers detailed in the tables below, it could be possible to allow their short-term administration during the trial after the end of Cycle 1 under the following circumstances:

- If a patient requires short-term administration of a restricted CYP3A4/5, Pgp (MDRI) or BCRP inhibitor (see Table 21), vistusertib treatment must be withheld for 3 days prior to the first dose and not restarted until the concomitant therapy has been discontinued for the appropriate washout period described in the table.
- If a patient requires short-term administration of a restricted CYP3A4/5, Pgp (MDR1) or BCRP isoenzyme inducer (see Table 21) this should be clearly documented on the eRDC and may be permitted, but the local Investigator should be aware that this could lead to lower levels of study drug and a potential reduction in clinical efficacy.

All patients should avoid concomitant use of specific substrates of the drug transporters OATP1B1, OATP1B3, MATE1 and MATE2K for the wash-out periods detailed in

Table 22 from the time they enter the screening period until 2 weeks after the last dose of treatment, ensuring no study treatment is started until the wash-out period appropriate for each drug has elapsed (at least 5 x elimination half-life). However it could be possible to allow their short-term administration during the study under the following circumstances:

If a patient requires short term administration of restricted substrates of OATP1B1, OATP1B3, MATE1 or MATE2K (see

Table 22), Vistusertib treatment must be withheld for 3 days prior to the first dose and not restarted until the concomitant therapy has been discontinued for the appropriate washout period described in the table.

Table 21: Prohibited cytochrome P450 and transported inhibitor/inducers

**CYP Enzyme Category***	**Drugs**	**Minimum drug wash-out period prior to vistusertib initiation**
CYP3A4/5 Strong Competititve inhibitors	Large quantities of grapefruit juice (see Section 23.2.3.4 for permitted quantity), indinavir, itraconazole, ketoconazole, nefazodone, nelfinavir, saquinovir, telithromycin, troleandomycin and voriconazole	1 week
	Idelalisib	2 weeks
CYP3A4/5 Strong Time dependent inhibitors	Bocepravir, clarithromycin, cobicistat, danoprevir, elvitegravir, LCL161 , lopinavir, **mibefradil****, posaconazole, ritonavir, telaprevir and tipranivir	2 weeks
CYP3A4/5 Strong inhibitors (classification unknown)	Conivaptan	1 week

CYP Enzyme Category*	Drugs	Minimum drug wash-out period prior to vistusertib initiation
CYP3A4/5 Moderate Competititve inhibitors	Large quantities of Seville oranges (see Section 23.2.3.4 for permitted quantity), Amprenavir, aprepitant, atazanavir, cimetidine, cyclosporine, fluconazole, imatinib and netupitant	1 week
CYP3A4/5 Moderate Time dependent inhibitors	ACT-178882, casopitant, crizotinib, darunavir, diltiazem, erythromycin, ledipasvir, lomitapide, tofisopam and verapamil	2 weeks
	FK1706	Half-life not found
CYP3A4/5 Moderate inhibitors (classification not known)	Ciprofloxacin and dronedarone	1 week
	Schisandra sphenanthera	Half-life not found
CYP3A4/5 Strong inducers	Carbamazepine, phenytoin, rifabutin, rifampicin and St. John's Wort	3 weeks
	Enzalutamide and phenobarbital	5 weeks
	Mitotane	114 weeks
	Avasimibe	Half-life not found
CYP3A4/5 Moderate inducers	Bosentan, genistein, lersivirine, lopinavir, modafinil, nafcillin, ritonavir, semagacestat,thioridazine and tipranavir	1 week
	Etravirine	2 weeks
	Efavirenz	3 weeks
	Talviraline	Half-life not found
Pgp (MDR1) inhibitors	Dronedarone, erythromycin, indinavir, itraconazole, ketoconazole, lapatinib, lopinavir, ritonavir, quinidine and verapamil	1 week
	Vorapaxer	10 weeks
	Valspodar (PSC 833)	Half-life not found
Pgp (MDR1) inducers	Carbamazepine and rifampin	3 weeks
BCRP inhibitors	Atazanavir, cyclosporine, lopinavir, ritonavir and tipranavir	1 week

Reference: University of Washington database

* CYP Enzyme Category:

- Strong inhibitor (yielding AUC ratio ≥5)
- Moderate inhibitor (yielding AUC ratio ≥ 2 and <5)
- Strong inducers (AUC decreased by ≥ 80% or CL increased by more than 5 fold (400%)
- Moderate inducers (AUC decreased by 50 - 80% or CL increased by more than 2-5 fold (100 - 400%)

** discontinued

Table 22: Transport Substrate Restrictions

Transporters	Substrates	Minimum drug wash-out period prior to vistusertib initiation
OATP (1B1 or 3)	Bosentan, fexofenadine, glybutride, pitavastatin, pravastatin, **repaglinide***, rosuvastatin	5 x reported terminal half-lives
MATE (1 or 2K)	Cisplatin	

Reference: Expert Opin. Drug Metab. Toxicol. (2013) 9(6):737-751
* Substrate has a narrow therapeutic index

23.2.3.3 Concomitant Radiotherapy

Concurrent radiotherapy is not permitted. If palliative radiotherapy is indicated, the patient should stop taking vistusertib on the day of radiation and should not recommence trial treatment for a minimum of 5 days after radiation. This should be decided on consultation with the National Lung Matrix Trial Office.

23.2.3.4 Food Restrictions

Sugary and fatty foods should be kept to a minimum in the meals prior to taking a dose.

Patients should be advised to avoid large amounts of grapefruit and Seville oranges (and other products containing these fruits, e.g., grapefruit juice or marmalade) during the study.
No more than a small glass of grapefruit juice (120 mL) or half a grapefruit or 1 to 2 teaspoons (15 g) of Seville orange marmalade daily is allowed. For patients who have potentially consumed large quantities prior to trial entry, see Table 21 for required washout period.

23.2.3.5 Sunlight-Protection Measures

Patients should be advised to avoid use of sun beds or tanning booths (including dye-based tanning booths) during the course of the study and for 3 months after the last dose and use sunglasses and sun blocker (with SPF >30 to ultraviolet (UV) B and a high degree of protection against UVA, if exposed to sunlight during this period of time.

23.2.3.6 Other Restrictions

Patients who begin Coumadin therapy should be advised to have their anticoagulation monitored more frequently when receiving vistusertib. In the event of thrombocytopenia, the guidelines in Section 23.3.4 should be followed.

Patients may continue to receive therapeutic bisphosphonates and erythropoietin preparations (Procrit, Epogen, Aranesp), if they were receiving them prior to beginning study treatment.

Blood transfusions are allowed during the trial.

Other medication that is considered necessary for the patient’s safety and well-being may be given at the discretion of the Investigators.

23.2.3.7 Contraception

Female patients must be willing to use 2 forms of highly effective contraception (per institution standards) (1 highly effective method and 1 barrier method) from the time of screening until 4 weeks after discontinuing the trial treatment, must not be breast feeding and must have a negative pregnancy test prior to start of dosing if of child bearing potential, or must have evidence of non-childbearing potential (see Section 6.3.1 for a definition of females of childbearing potential and females of non-childbearing potential).

Acceptable methods of highly effective contraception include:

- Placement of an intrauterine device or intrauterine system
- Male partners sterilisation (with the appropriate post-vasectomy documentation of the absence of sperm in the ejaculate)
- True abstinence.

Please note: use of oral, injected, or implanted hormonal methods of contraception cannot be considered highly effective as it is currently unknown whether vistusertib may reduce their effectiveness.

Acceptable barrier methods of contraception include: condom or occlusive cap (diaphragm or cervical/vault caps) with spermicidal foam/gel/film/cream/suppository

Male patients should either be surgically sterile or willing to use an effective barrier method of contraception during the study and for 16 weeks following the last dose of study treatment if sexually active with a female of childbearing potential. Storage of sperm prior to receiving vistusertib will be advised to male patients with a desire to have children.
Male patients should abstain from sperm donation during exposure to vistusertib.

It is not currently known whether vistusertib affects fertility in humans.

23.3 Trial Treatment

23.3.1 Investigational Medicinal Product

Vistusertib is available for administration as a tablet for use in clinical trials. The tablet is presented as one strength: 25 mg. Vistusertib will be provided by AstraZeneca free of charge.

Vistusertib will be administered twice daily orally on an intermittent schedule at a dose of 125 mg BD 2 continuous days out of 7. Intermittent dosing is recommended because of improved tolerability with fewer patients stopping treatment as a consequence of toxicity. The safety profile of intermittent dosing with vistusertib differs from that of continuous dosing. The incidence of vomiting, AST increase, ALT increase and oral pain appears greater in patients receiving 125 mg BD according to the intermittent schedule when compared with patients receiving 50 mg BD continuously. In contrast the incidence of mucositis, rash, pruritus, hyperglycaemia, anaemia, dry skin, constipation and dehydration is higher in patients receiving a dose of 50 mg BD continuously. The rate of treatment discontinuation due to toxicity is higher amongst patients receiving continuous dosing. Anticancer activity is evident amongst patients treated on both schedules.

Where possible all doses of vistusertib should be taken at approximately the same times each day. Twice daily doses should be taken approximately 12 hours apart. Vistusertib can be taken with or without food. If vomiting occurs within 30 minutes after vistusertib dosing, or later if the tablet(s) can be identified in the vomit content, the patient can re-take a new tablet(s).

Should a patient miss a scheduled dose, the patient will be allowed to take the dose up to a maximum of 2 hours after the scheduled dose time. If greater than 2 hours after the scheduled dose time, the missed dose should not be taken and the patient should take their allotted dose at the next scheduled time. If a patient needs to take the dose earlier for whatever reason, the patient can take the dose up to 2 hours earlier than the scheduled dose time. The patient should make every reasonable effort to take the vistusertib tablet(s) on time.

Please note patients who meet RECIST criteria for progressive disease (PD) may be continued on trial treatment if the treatment is tolerable and the Investigator believes it to be of clinical benefit; see Section 9.3.

Both the Arm B Patient Information Sheet and Arm B Patient Diary contain more specific instructions for patients to follow regarding how to take their medication.

Please also refer to the Pharmacy Manual for further details.

23.3.2 Schedule of Assessments

Table 23: Vistusertib - Schedule of Assessments

	**Screening**	**Treatment 125 mg bd 2 continuous days/7 (28 day cycles)**		**Discontinuation** (+ 7 days)**	**28 day follow up visit[s]** (+ 7 days)**	**Post-28 day follow up** (± 7 days)***
	Within 28 days treatment (unless otherwise stated)	Cycle 1 Day 1	Day 1 of subsequent cycles (± 2 days)*			
Informed consent[a]	X					
Demography & baseline characteristics[b]	X					
Medical history[c]	X					
Inclusion / exclusion criteria[d]	X					
Physical examination[e]	X	X	X	X		
ECOG performance status	X (within 14 days of treatment)	X	X	X		
Vital signs (inc. weight)[f]	X	X	X	X		
ECG[g]	X		X (every 8 weeks beginning cycle 2)	X	X	
MUGA / ECHO[h]	X	X (if clinically indicated)		X	X (if clinically indicated)	
Haematology, Clinical chemistry, Urinalysis[i]	X (within 7 days of treatment)	X (- 2 days)	X (- 2 days)	X		
Fasted glucose	X					
Pregnancy test[j]	X	X		X		

	Screening	Treatment 125 mg bd 2 continuous days/7 (28 day cycles)		Discontinuation (+ 7 days)**	28 day follow up visit[s] (+ 7 days)**	Post-28 day follow up (± 7 days)***
	Within 28 days treatment (unless otherwise stated)	Cycle 1 Day 1	Day 1 of subsequent cycles (± 2 days)*			
Tumour assessments[k]	X	Every 6 weeks during year 1 (± 7 days) except 1st scan + 7 days only)				X ◊
Adverse events & Concomitant Medications	X		X	X	X	
Dispense/administer study drug[l]		X (- 2 days)	X (- 2 days)			
Administer study drug[m]		X	X			
Smoking status[n]		X	X (every 8 weeks beginning cycle 3)	X		
Germline DNA sample[o]		X (- 2 days)				
ctDNA samples[p]		X (- 2 days)	X (every 8 weeks beginning cycle 3) (- 2 days)	X		X $
Optional research biopsy[q]		X (post-reg, pre-tx)		X		
Survival status[r]						X

* Visit may occur ± 2 days of the planned visit date. Individual assessments may occur independently of the visit date where indicated in the table above

** Visit may occur + 7 days of the planned visit date

*** Visit may occur ± 7 days of the planned visit date

a Prior to the start of any study specific procedures, each patient must provide signed informed consent.

b Demography must be captured for all patients. Demographic data and other characteristics will include: date of birth, gender, race/ethnicity.

c A standard medical and surgical history will be obtained, including prior cancer treatment.

d Patients must not be registered unless all eligibility criteria have been fully met.

e Physical examination includes general appearance, respiratory, cardiovascular, skin, head and neck (including ears, eyes, nose and throat), lymph nodes, thyroid, abdomen, musculo-skeletal (including spine and extremities) and neurological systems and should be performed at screening, day 1 of each cycle and at discontinuation. Constitutional symptoms will be collected during screening and pre-dose at all other visits. Constitutional symptoms will include the presence/absence of pruritus, night sweats, recurrent fever ≥38.0ºC, fatigue, weakness and nocturia (a history of weight loss is to be collected at screening only).

f Vital signs (HR, systolic and diastolic BP, oxygen saturation (pulse oximetry), respiration rate, weight, height (at screening) and temperature). Single measurements of supine BP and pulse will be recorded on each occasion after 10 minutes rest. Vital signs to be taken at screening, day 1 of every cycle and discontinuation.

g ECG to be taken at screening then **pre-dose**, 2 hours and 6-8 hours post dose on cycle 2 day 1. Further ECGs assessments should be performed **pre-dose (**where applicable) every 8 weeks (day 1 of every other cycle), at discontinuation and 28 day follow up. Twelve-lead ECGs will be obtained after the patient has been resting semi-supine for at least 10 minutes prior to times indicated. All ECGs should be recorded with the patient in the same physical position. For each time point unless specifically stated above, three ECG recordings should be taken at a minimum of 5 minute intervals (all three ECGs must be collected within 30 minutes). A standardised ECG machine should be used and the patient should be examined using the same machine throughout the study if possible. After paper ECGs have been recorded, the Investigator or designated physician will review each of the ECGs and may refer to a local cardiologist if appropriate. A paper copy should be filed in the patient's medical records.

If an abnormal ECG finding at screening or cycle 2 day 1 is considered to be clinically significant by the Investigator, it should be reported as a concurrent condition. During the study, clinically significant abnormal ECG findings not present at baseline should be reported as an AE. If present, the clinical signs and symptoms associated with the abnormal finding should be reported as the AE with the ECG abnormality given as explanatory information. The modality of the cardiac function assessments must be consistent within patient and the same machine operator is to be used where possible. An ECG should be performed at any cardiac event with symptoms that may be due to cardiac ischemia, or arrhythmia (such as chest pain or palpitations). An ECG will also be captured in all cases of dyspnoea and pulmonary oedema and additionally at the discretion of the Investigator if clinically indicated. For all ECGs details of rhythm, ECG intervals (R-R, PR, QT and QRS) and an overall evaluation will be recorded.

h A scan carried out within 12 weeks prior to starting study treatment can be used for eligibility at screening, however, for patients who experience a significant cardiac event or who have had cardiotoxic medication within this interval, the MUGA/ECHO scan should be repeated prior to starting study treatment. MUGA/ECHO should be performed during treatment (if clinically indicated only), at discontinuation and 28 day follow up (where abnormal LV function was previously diagnosed). Echocardiography will also be carried out if a patient develops signs and/or symptoms suggestive of a deterioration in left ventricular function or in case of the pre-specified ECG finding such as T-wave inversions.. The modality of the cardiac function assessments must be consistent within patient and the same machine operator is to be used where possible.

i Samples to be collected at screening (within 7 days of treatment), day 1 of each cycle and at discontinuation. Samples for cycles can be taken up to 2 days earlier than the actual visit date (commencement of cycle). Clinical Chemistry: Albumin, AST, ALT, ALP, bilirubin (total), calcium (total), creatinine, glucose, magnesium, phosphate, sodium, urea nitrogen, potassium, total protein, triglycerides and cholesterol.

Cardiac markers creatine kinase (CK), Troponin (isoform as per institutional norm), lactate dehydrogenase (LDH) and AST should be assessed at screening and pre-dose on first day of treatment.

INR monitoring and aPTT assessments should be performed at screening, day 1 of every other cycle (commencing Cycle 2 Day 1), and when clinically indicated. If there are no findings after repeated measurements, the assessments can be stopped from Cycle 6 Day 1 onwards, and only performed when clinically indicated.

Troponin (additionally other cardiac markers, i.e. CK, AST and LDH depending on the Investigators decision if clinically indicated) should also be assessed on identification of abnormal ECG findings, e.g. new repolarisation abnormalities, found to be possibly clinically significant by Investigators judgement. A repeat cardiac marker assessment should also be performed 24 hours later if such changes have been observed. The same Troponin isoform should be assessed at each of the visits.

An unscheduled serum urea and creatinine test should be performed in every case of an SAE of diarrhoea.

Haematology: FBC, Hba1c

Urinalysis: glucose and blood will only be measured at screening and if clinically indicated. Proteinuria assessments should be performed at screening, day 1 of every other cycle (commencing Cycle 2 Day 1), and when clinically indicated. If there are no findings after repeated measurements, the assessments can be stopped from Cycle 6 Day 1 onwards, and only performed when clinically indicated.

Additional assessments may be performed at the discretion of the Investigator if clinically indicated.

All patients with clinically significant abnormal laboratory results at treatment completion or study drug discontinuation visit are to be followed until the results return to normal (or patient's baseline), or until a valid reason, other than a drug-related effect, is identified. Patients with an unresolved AE or SAE event at treatment completion or study drug discontinuation will be contacted by the Investigator or his or her designee to determine the status of the event until the event is resolved or stabilised, the patient is lost to follow up, or it has been determined that the study treatment or participation is not the cause of the event.

j Investigator should assess the patient's compliance to contraceptive measures and perform a test if required. Female patients of child-bearing potential only. A serum or urine pregnancy test is to be performed at screening, pre dose on cycle 1 day 1 and at discontinuation. In the event of suspected pregnancy during the study, the test should be repeated and, if positive, the patient discontinued from study treatment immediately.

k CT or MRI scan of head, chest and abdomen to be performed at screening. CT or MRI scans of chest and abdomen to be performed until discontinuation. Following screening the first tumour assessment should be performed 6 weeks after cycle 1 day 1, then every 6 weeks thereafter for the first year, later reducing to every 12 weeks. Scans should be performed ± 7 days (except 1st scan + 7 days only). If brain metastases are identified at Screening or if clinically indicated, head scanning will also be performed throughout treatment at the same time points**. The same imaging modality must be used consistently throughout the course of the trial for each patient.**

◊ For any new respiratory symptoms (cough, dyspnoea, lower respiratory infection) not clearly explained by other factors (e.g., dyspnoea associated with substantial drop in haemoglobin), patients should have oxygen saturation measured. If <92%, the high resolution CT or MRI scan of the chest should be repeated and pulmonary function tests should be performed.

Tumour assessments will be performed in follow up for patients who discontinue treatment for reasons other than Progressive Disease (e.g. toxicity). These scans should continue to be performed on a 6-weekly basis for the first year relative to the start date of treatment, then every 12 weeks until disease progression or the patient starts a new anti-cancer therapy (unless the patient withdraws consent to do so). Scans should be of the chest and abdomen, and only include the head where brain metastases are identified at screening, or if clinically indicated. All scans to be reported using RECIST 1.1.

l Vistusertib must be dispensed within the IWRS Cenduit system. Refer to the Pharmacy Manual for further details. Vistusertib may be dispensed within the IWRS up to 2 days prior to the actual visit date.

m Cycle 1 day 1: Treatment must commence within 7 days of trial registration.

n Smoking status data will be collected through questions and CO monitoring **pre-dose** cycle 1 day 1, cycle 3 day 1 then every 8 weeks (day 1 of every other cycle) and at discontinuation.

o A whole blood germline DNA sample is to be collected **pre-dose** on Cycle 1 day 1. The sample can be taken up to 2 days earlier than the actual visit date (commencement of cycle). If the sample is not collected at this timepoint, it should be collected at the next visit. Refer to the Laboratory Manual for sample processing guidelines.

p ctDNA samples to be collected at **pre-dose** cycle 1 day 1, then every 8 weeks (day 1 of every other cycle) beginning cycle 3 and at discontinuation. ctDNA samples can be taken up to 2 days earlier than the actual visit date (commencement of cycle). Refer to the Laboratory Manual for sample processing instructions.

$ ctDNA samples will be collected in follow up for patients who discontinue treatment for reasons other than Progressive Disease (e.g. toxicity). These samples should be performed at the same visit as follow up CT or MRI scans until disease progression or the patient starts a new anti-cancer therapy (unless the patient withdraws consent to do so). Samples should be collected on a 6-weekly basis for the first year relative to the start date of treatment then every 12 weeks.

q An optional fresh metastatic/recurrent tumour biopsy sample should be collected (if patient consents) post–registration (pre-treatment) and at the end of treatment visit for patients who discontinue treatment for reasons other than disease progression (origin from either the primary tumour of site of metastasis). An optional pre-treatment biopsy should not be performed in cases where the patient has already had a mandatory biopsy for molecular testing (Note - a mandatory repeat SMP2 biopsy will be performed if the patient has had targeted therapy e.g. ALK inhibitor). The discontinuation biopsy must be performed prior to commencing further anti-cancer therapy. A post-treatment biopsy will only be requested from patients with an objective response or stabilisation of disease (PR or CR), or 6 months on treatment with evidence of stabilisation (SD) for patients who have previously progressed. The tumour tissue will be used to determine possible mechanisms of resistance to study treatment. Refer to the Laboratory Manual for sample processing instructions.

r Survival status will be collected every 12 weeks (± 7 days) post-permanent discontinuation of vistusertib until death.

s 28 day follow up visit should be carried out 28 days (+ 7 days) post-permanent discontinuation of vistusertib.

23.3.3 Toxicity Profile

23.3.3.1 Safety issues

Although the incidence rate of some AEs seems to be common to frequent, vistusertib is considered to be generally well tolerated with most of the AEs classified as CTCAE grade 1 and 2, AEs disappearing quickly after short periods of dose interruptions, which allowed patients to stay on the drug for long periods of time (up to over 1 year). Fatigue, mucositis, rash, nausea, vomiting, diarrhoea, hyperglycaemia and decreased appetite were the AEs consistently amongst the most frequently reported overall, and those reported as related to vistusertib by the Investigators. These AEs were also those reported with the highest severity grade and were those AEs most commonly leading to dose interruption and/or permanent discontinuation of vistusertib. These events were generally reversible within 1 week by cessation of vistusertib.

Overall, no clinically significant changes were observed in HR or BP. Single intermittent systolic or diastolic BP values above and below normal values were reported in a few patients. Intermittent higher HR >100 bpm were observed, and tachycardia in some patients.

Overall, no clinically significant changes were observed in vital signs or laboratory data. Review of emerging laboratory data did not reveal clinically significant changes in transaminases, ALP or bilirubin considered related to vistusertib. Transaminase increases (ALT or AST) were reported as AEs in some patients; the majority were CTCAE Grade 1 and were not considered related to vistusertib by the reporting Investigator. Laboratory data suggest that for most patients who do have reductions in cell counts, values return to patients' baseline on stopping vistusertib. The clinical relevance of these changes appears low given that there was no excess of related AEs such as infection, fungal disease, viral disease, dyspnoea, or bleeding within these patients when compared with patients not noted as having reductions, and there were no events of greater severity.
ECGs repolarisation changes were seen in some patients. However, there were no associated significant QRS morphology changes and the clinical significance of these changes is also unknown.

23.3.3.2 Expected Adverse Events – Important Identified Risks

23.3.3.2.1 Fatigue or lethargy

Fatigue was amongst the most common AEs reported with a single dose of vistusertib, and at doses of 70 mg BD and 100 mg BD monotherapy was amongst those events considered as DLT. Fatigue is generally reversible with AEs improving in severity of resolving completely within 1 week after vistusertib is stopped or dose reduced.

At the time of data cut-off, the incidence of fatigue events is 73%, 65%, 15% 36%, 0%, 61% and 43% in trial D2270C00001, trial D2270C00005, trial D2270C00008, trial D2274C00001, D2270C00015, D2270C00020 and trial D4620C00001 respectively. Fatigue events include the MedDRA preferred terms of Fatigue and Lethargy.

23.3.3.2.2 Mucosal Inflammation (Including Stomatitis)

Mucositis was amongst the most common AEs reported with a single dose of vistusertib, and at doses of 70 mg BD and 100 mg BD monotherapy was amongst those events considered as DLT. Mucositis is generally reversible with AEs improving in severity of resolving completely within a few days of (1 day to 1 week) after vistusertib is stopped or dose reduced.

As mucositis is reported as a possible class effect of mTOR inhibitors appropriate management guidance has been put in place for all vistusertib studies.

At the time of data cut-off, the incidence of mucosal inflammation events is 35%, 50%, 30% 18%, 75%, 30% and 6.7% in trial D2270C00001, trial D2270C00005, trial D2270C00008, trial D2274C00001, D2270C00015, D2270C00020 and trial D4620C00001 and trial D2274C00001, respectively. Mucosal inflammation events include the MedDRA PTs of Cheilitis, Mucosal inflammation, Mucositis, Mouth ulceration, Oral mucosal erythema, Stomatitis, Tongue ulceration.

23.3.3.2.3 Gastrointestinal events (Nausea and/or Vomiting)

Nausea, vomiting and diarrhoea were amongst the most common AEs reported after a single dose of vistusertib.

At the time of data cut-off, the incidence of nausea is 60%, 68%, 48%, 0%, 75%, 46% and 53%, in trial D2270C00001, trial D2270C00005, trial D2270C00008, trial D2274C00001, D2270C00015, D2270C00020 and trial D4620C00001 respectively.

The incidence of vomiting is 36%, 46%, 26%, 9%, 75%, 24% and 27% in trial D2270C00001, trial D2270C00005, trial D2270C00008, trial D2274C00001, D2270C00015, D2270C00020 and trial D4620C00001, respectively.

23.3.3.2.4 Rash

In study D2270C00001 Part B expansion at 50 mg BD, 24 (47.1%) patients experienced rash of any type. The majority (39.2) were Grade 1.
At the time of data cut-off, the incidence of rash events is 39%, 41%, 33%, 9%, 0%, 37%10% and 9% in trial D2270C00001, trial D2270C00005, trial D2270C00008, trial D2274C00001, D2270C00015, D2270C00020 and trial D4620C00001, respectively. Rash events include the MedDRA PTs of Dermatitis acneiform, Eczema, Erythema, Exanthema, Rash, Rash erythematous, Rash generalised, Rash macular, Rash maculo-papular, Rash papular, Rash pruritic, Rash pustular, Rash vesicular, Urticaria.

23.3.3.2.5 Glucose Homeostasis effects (Hyperglycaemia)

Emerging laboratory data suggest a trend towards increase in blood glucose over time. Insulin and blood glucose elevations have been observed in both monotherapy and in combination therapy studies, a finding frequently described with other mTOR inhibitors as well.
At the time of data cut-off, the incidence of hyperglycaemia events is 22%, 16%, 4%, 9%, 0%, 9%, and 10% in trial D2270C00001, trial D2270C00005, trial D2270C00008, trial D2274C00001, D2270C00015, D2270C00020 and trial D4620C00001, respectively. Hyperglycaemia events include the MedDRA PTs of Hyperglycaemia, Blood glucose increased.

There have been reports of SAEs of hyperglycaemia reported with vistusertib, including patients requiring in patient hospitalisation and treatment with insulin to achieve glycaemic control. A patient receiving vistusertib 225mg BD intermittent weekly schedule monotherapy developed grade 3 hyperglycaemia, requiring hospitalisation and has to be treated with insulin to achieve glycaemic control and this was declared a DLT.

As hyperglycaemia is reported as a possible class effect of mTOR inhibitor, appropriate inclusion/exclusion criteria, and monitoring of patients, has been put in place for all vistusertib studies.

Should patients develop severe hyperglycaemia, patients may require treatment in an intensive care unit. Due to the short half-life of vistusertib, only a short period of insulin resistance is expected. Therefore early treatment with high doses of insulin should be carefully evaluated and blood sugars monitored as per standard clinical practice.

23.3.3.2.6 Adrenal effects (Hypokalaemia)

In the rat, reversible diffuse vacuolation of the zona glomerulosa (sighting and pivotal study) and mild cortical atrophy of the adrenal gland (1 month study) were observed.

At the time of data cut-off, the incidence of hypokalaemia is 14%, 15%, 4%, 9%, 0%, 4% and 7% in trial D2270C00001, trial D2270C00005, trial D2270C00008, trial D2274C00001, D2270C00015, D2270C00020 and trial D4620C00001, respectively. Hypokalaemia events include the MedDRA PTs of Hypokalaemia, blood potassium decreased.

23.3.3.3 Expected Adverse Events – Other Identified Risks

23.3.3.3.1 Hypophosphataemia

Increases in ALP, without associated liver histopathology, of uncertain toxicological significance have been seen in rats.

At the time of data cut-off, the incidence of hypophosphataemia is 11%, 3%, 0% and 0% in trial D2270C00001, trial D2270C00005, trial D2270C00008, and trial D2274C00001, respectively.

23.3.3.3.2 Decreased Appetite

In rats inhibition of intestinal transit time and gastric emptying has been observed, as has a reduction in food consumption and weight loss. In dogs DLTs have included reduced food consumption and weight loss.

At the time of the data cut-off, the incidence of decreased appetite is 47%, 29%, 18% and 0% in trial D2270C00001, trial D2270C00005, trial D2270C00008, and trial D2274C00001, respectively.

23.3.3.3.3 Pneumonitis and interstitial lung disease (ILD)

At the time of data cut-off, the incidence of non-infectious pneumonitis events is 2%, 3%, 0%. 0%, 0%, and 2% in trial D2270C00001, trial D2270C00005, trial D2270C00008, trial D2274C00001, D2270C00015, D2270C00020 and trial D4620C00001, respectively. One patient experienced an SAE of pneumonitis in Study D4620C00001.
As pneumonitis is reported as a possible class effect of mTOR inhibitors appropriate inclusion/exclusion criteria, and monitoring of patients, has been put in place for all vistusertib studies. Patients undergo scans at baseline, and all reported pulmonary events are analysed retrospectively in detail in regard to signs of pneumonitis. In review of reported cases of pneumonitis, radiological assessments are confounded by other factors, like anaemia, lung metastasis and infections and/or chest wall infiltration and a causal association has not been established.

23.3.3.4 Expected Adverse Events – Other Potential Risks

23.3.3.4.1 Cardiac Effects including ECG (repolarisation) Changes

In pre-clinical Safety Pharmacology studies, increases in HR, BP, myocardial contractility, coronary flow and minor QTc prolongation were observed. Study D2270C00001 (study 1) and

Study D2270C00005 (study 5) were therefore designed with appropriate monitoring including triplicate ECG assessments at screening, during the studies, at study drug discontinuation, and at the 30-day follow-up visit. MUGA or echocardiography was performed at screening, at discontinuation and during the study when clinically indicated.

As of 2nd December 2013 over 155 patients had received at least one dose of vistusertib in Study D2270C00001 or Study D2270C00005. All ECGs were reviewed centrally by a contract vendor. Repolarisation abnormalities (T wave flattening and or T wave inversion) were identified in 11 patients; 8 from study D2270C00001 and 3 patients from study D2270C00005.

All ECG repolarisation changes from first 54 patients in study D2270C00001 were reviewed by an independent external cardiologist, who identified ECG repolarisation changes comprising progressive T-wave flattening in 4 of the 54 patients. In 3 of those patients who continued on treatment the flattening progressed to T-wave inversion. These findings occurred slowly, starting from minimal T-wave flattening to inversions over several weeks, stayed at negative values for several weeks and returned back to low/normal over a period of weeks while on treatment. The patients with these findings showed no clinical signs and symptoms of compromised cardiac function or any elevations in cardiac enzymes. Thirty-day follow-up ECGs were available for 2 patients. ECGs of one patient returned back to normal values and ECGs of another patient the limb leads were very similar to baseline but the chest leads stayed generally smaller in amplitude and remained inverted in V2 and V3.

In the monotherapy trial, study D2270C00001, another 4 patients developed similar findings, mostly however T-wave flattenings without inversions (except 1 patient, who showed both, flattening and inversions). The patient with both findings, experienced recovery from both T-wave abnormalities while on treatment. For the 3 other patients, the T-wave flattening occurred irregularly during the treatment periods with intermittent normal ECGs, however, resolved completely after stopping the drug permanently. In the combination trial, (Study D2270C00005) T-wave flattenings were observed in 3 patients intermittently and resolved while on treatment.

None of these 11 patients showed any cardiac signs or symptoms at the time of the ECG changes and 1 of these patients has had continuous electrolyte balances, which may have contributed to the findings.

The external cardiologist's opinion regarding these changes is as follows:

- There are no significant QRS morphology changes during the trial.
- Repolarisation changes are therefore primary rather than secondary to a change in depolarisation.
- These 4 patients showed stereotyped progressive repolarisation abnormalities with T wave flattening and inversion most marked in the lateral chest leads.
- The mechanism of these changes is unknown
    - their progressive development over weeks is not compatible with an ischaemic insult
    - their variable onset and progression suggests that on-going screening during dosing is required
    - their reduction during on-going drug therapy is not compatible with a purely dose dependent phenomenon
    - no risk factor for the development of these repolarisation changes has been identified
    - as T wave morphology is thought to be determined by differential depolarisation across the myocardial wall, *in vitro* study may be helpful
    - although inter-species differences may hinder interpretation.
- The clinical significance of these changes is also unknown although no clinical events related to left ventricular function or arrhythmia have been recorded.

Patients who develop persistent, confirmed T wave repolarization abnormalities (inversion or flattening) on regularly scheduled ECGs should have a follow up ejection fraction determined using the same technology used at baseline (ECHO or MUGA), as well as a troponin measurement.

23.3.3.4.2 Left ventricular ejection fraction

Of patients who underwent ECHO to estimate left ventricular ejection fraction, there were no significant changes in LVEF measurements across all patients. All values in patients who underwent multi-gated acquisition (MUGA) scans were within the normal range for the institution.

23.3.3.4.3 Haematological effects

Changes in bone marrow (hypocellularity and/or increases in adipose tissue) and secondary lymphoid tissues have been seen in rats and dogs. Associated changes in peripheral haematological parameters (notably reductions in lymphocytes, neutrophils and/or reticulocyte counts) have also been seen. All changes showed evidence of reversibility.

At the time of data cut-off, the incidence of anaemia ranges from 14% to 50% in Studies D2270C00001, D2270C00005, D2270C00015, and D2274C00001, D2270C00020b, and D4620C00001c.

At the time of data cut-off, the incidence of neutropenia ranges from 0% to 25% in Studies D2270C00001, D2270C00005, D2270C00015 and D2274C00001. Note: the upper range of 25% was driven by 1 patient in Study D2270C00015, where patient numbers were low.

At the time of data cut-off, the incidence of thrombocytopenia ranges from 0% to 5% in Studies D2270C00001, D2270C00005, D2270C00015, and D2274C00001. The incidence of thrombocytopenia in D2270C00020b was 13%.

23.3.3.4.4 Photosensitivity

Phototoxicity has been classified as a potential risk for vistusertib; however there have been no relevant clinical findings so far. Patient should be advised of the need for sunlight protection measures such as use of sunscreen and sunglasses during treatment with vistusertib, and should be advised to adopt such measures for a period of 3 months after receiving their final dose of vistusertib.

23.3.3.4.5 Renal effects

Renal effects have been classified as a potential risk for vistusertib. However, no clinically significant renal findings were reported at any dose of vistusertib so far. Clinical study protocols exclude patients with impaired renal function or concurrent specific renal disease from participation in studies using vistusertib.

23.3.3.4.6 Transaminases increased

Reversible increases in ALP and ALT have been seen in rats. In the absence of liver histopathology these findings are of uncertain toxicological significance.

At the DCO, 25.1% of patients had a maximum CTCAE Grade 1 or 2 and 2.2% had Grade 3 or 4 in ALT in trial D2270C00001. At the DCO, 60.4% of patients had a maximum CTCAE Grade 1 or 2 and 4.2% had Grade 3 or 4 in ALT in trial D2270C00005.

At the DCO, 37.0% of patients had a maximum CTCAE Grade 1 or 2 and 0.7% had Grade 3 or 4 in AST in trial D2270C00001. At the DCO, 68.8% of patients had a maximum CTCAE Grade 1 or 2 and 13.5% had Grade 3 or 4 in AST in trial D2270C00005.

At the DCO, 21.5% of patients had a maximum CTCAE Grade 1 or 2 and 2.9% had Grade 3 or 4 in bilirubin in trial D2270C00001. At the DCO, 12.6% of patients had a maximum CTCAE Grade 1 or 2 and 1.0% had Grade 3 or 4 in bilirubin in trial D2270C00005.

23.3.3.4.7 Infection

Not observed in pre-clinical studies. At the DCO, the incidence of infections ranges from 36% to 54% in Studies D2270C00001, D2270C00005, D2270C00015, and D2274C00001.

23.3.4 Dose Modifications and Toxicity Management

Vistusertib is expected to be well tolerated. Substantial acute toxicities should be managed as medically indicated and with temporary suspension of study drug, as appropriate. Dose reductions or holds and initiation of supportive care are allowed as clinically indicated by the treating physician. For each patient, a maximum of 3 dose reductions will be allowed.

23.3.4.1 Treatment delays and discontinuation

In general, if a patient experiences a Grade 1 or Grade 2 haematological or non-haematological toxicity, no dose modification is required.

If a patient experiences a Grade 3, Grade 4 or otherwise clinically significant toxicity not attributable to the disease or disease-related processes under investigation, dosing will be interrupted and/or the dose reduced (see Table 24: Vistusertib Dose Modifications and Table 25: Dose modifications and discontinuation criteria for CTCAE Grade 3 or 4 haematological toxicities) and supportive therapy administered as required (see Section 23.3.4.4).

If the toxicity resolves or reverts to CTCAE Grade 2 or less within 2 treatment weeks, and the patient is showing clinical benefit, treatment with vistusertib may be restarted.

If the toxicity does not resolve to ≤ CTCAE Grade 2 after 2 weeks off treatment, and the patient does not show clinical benefit, then the Investigator should consider permanent treatment discontinuation on consultation with the National Lung Matrix Trial Office, and the patient should be observed until resolution of the toxicity. Maximal drug holiday allowed is 2 treatment weeks, therefore the maximum number of days allowed is 19 days (14 days + 5 from the drug holiday in Week 1, which still counts as "treatment week").

If the toxicity does not resolve to ≤CTCAE Grade 2 after 2 weeks off treatment but the patient does show clinical benefit, treatment with vistusertib may be restarted after discussion with the National Lung Matrix Trial Office.

23.3.4.2 Criteria for recommencement of treatment

The following parameters should be met prior to recommencement of treatment: 1) at the start of every new cycle and; 2) following a treatment interruption for treatment related toxicity:

- Platelet count ≥ 50,000/µL
- ANC ≥ 1000/µL
- No drug-related non-haematological Grade ≥3 toxicity on day 1.

If these criteria are not met (but the patient derives clinical benefit), or in the event of a treatment interruption for reasons other than treatment related toxicity (e.g., non-cancer related surgery) lasting >3 weeks, it is advised that the entire cycle is only delayed to a maximum of 4 treatment weeks (33 days [4 weeks i.e. 28 days + 5 from the drug holiday in previous week, which still counts as a “treatment week”] for vistusertib). Treatment resumption will be decided on consultation with the National Lung Matrix Trial Office, and once the results of repeat assessments indicate that these criteria have been met.

23.3.4.3 Dose reductions

Table 24: Vistusertib Dose Modifications

**Dose level**	**Vistusertib Monotherapy dose (2 days on, 5 days off)**
Starting Dose Vistusertib	125 mg BD
-1 Dose Level	100 mg BD
-2 Dose Level	75 mg BD
-3 Dose Level	50 mg BD
N/A	Discontinue trial treatment

BD: Twice daily, N/A: not applicable

Dose reduction and discontinuation guidelines for haematologic and non-haematologic toxicities are shown in Table 25 and Table 26. Dose reductions of vistusertib should only be considered if the toxicity is considered to be related to vistusertib, i.e. in monotherapy studies or in combination studies if relationship cannot be wholly attributed to the combination agent (each combination agent should be considered on an individual basis).

In general, if a patient experiences a Grade 1/2 haematological or non-haematological toxicity, no dose modification is required.

If a patient experiences a Grade 3 or 4 toxicity (including haematological toxicities occurring in monotherapy studies), not attributable to the disease or disease-related processes under investigation, dosing will be interrupted and/or the dose reduced, see Table 26 below, and supportive therapy administered as required. For Grade 3/4 haematological toxicities occurring in combination studies see Table 25 below.

Dose re-escalation is not permitted.

Table 25: Dose modifications and discontinuation criteria for CTCAE Grade 3 or 4 haematological toxicities

**Toxicity Grade**	**Vistusertib Action**
**Febrile neutropenia** Grade 3 or 4	Withhold vistusertib until infection is resolved, antibiotics no longer required and ANC Grade ≤2 or baseline<br>• Vistusertib restart with 1st dose reduction (see Table 24).

Toxicity Grade	Vistusertib Action
2nd episode of **febrile neutropenia** Grade 3 or 4	Withhold vistusertib until infection is resolved, antibiotics no longer required and ANC Grade ≤2 or baseline<br>• Vistusertib restart with 2nd dose reduction (see Table 24).
3rd episode of **febrile neutropenia** Grade 3 or 4	Discontinue vistusertib
**Non-febrile neutropenia** Grade 4 lasting >7 days despite growth factor support	Withhold vistusertib until Grade ≤2 or baseline<br>• Vistusertib restart with 1st dose reduction (Table 24).
2nd episode of **non-febrile neutropenia** Grade 4 lasting >7 days despite growth factor support	Withhold vistusertib until Grade ≤2 or baseline<br>• Vistusertib restart with 2nd dose reduction (see Table 24)
3rd episode of **non-febrile neutropenia** Grade 4 lasting >7 days despite growth factor support	Withhold vistusertib until Grade ≤2 or baseline<br>• Vistusertib restart with 3rd dose reduction (see Table 24)
4th episode of **non-febrile neutropenia** Grade 4 lasting >7 days despite growth factor support	Discontinue vistusertib
**Thrombocytopenia**, Grade 4 without bleeding requiring red blood cell (RBC) transfusion	Withhold vistusertib until Grade ≤2 or baseline<br>• Vistusertib restart with 1st dose reduction (Table 24).
2nd episode of **thrombocytopenia**, Grade 4 without bleeding requiring RBC transfusion	Withhold vistusertib until Grade ≤2 or baseline<br>• Vistusertib restart with 2nd dose reduction (Table 24)
3rd episode of **thrombocytopenia**, Grade 4 without bleeding requiring RBC transfusion	Withhold vistusertib until Grade ≤2 or baseline<br>• Vistusertib restart with 3rd dose reduction (Table 24)
4th episode of **thrombocytopenia**, Grade 4 without bleeding requiring RBC transfusion	Discontinue vistusertib
**Thrombocytopenia**, Grade 3 or 4 **with bleeding** requiring RBC transfusion	Withhold vistusertib until Grade ≤2 or baseline<br>• Vistusertib restart with 1st dose reduction (Table 24).
2nd episode of **thrombocytopenia**, Grade 3 or 4 **with bleeding** requiring RBC transfusion	Withhold vistusertib until Grade ≤2 or baseline<br>• Vistusertib restart with 2nd dose reduction (Table 24)
3rd episode of **thrombocytopenia**, Grade 3 or 4 **with bleeding** requiring RBC transfusion	Discontinue vistusertib

Table 26: Dose modifications and discontinuation criteria for CTCAE Grade 3/4 non-haematological toxicities* (except liver dysfunction**)

Occurrence	Vistusertib Action
**First**	Withhold vistusertib until recovery to Grade ≤2 or baseline<br>• Vistusertib restart with 1st dose reduction (see Table 24)

Occurrence	Vistusertib Action
**Second**	Withhold vistusertib until recovery to Grade ≤2 or baseline<br>• Vistusertib restart with 2nd dose reduction (see Table 24)
**Third**	Withhold vistusertib until recovery to Grade ≤2 or baseline<br>• Vistusertib restart with 3rd dose reduction (see Table 24)
**Fourth**	Vistusertib - Withhold until recovery to Grade ≤2 or baseline; restart with 4th dose reduction (or discontinue if lowest level already reached, see Table 24)
**Fifth**	Vistusertib - discontinue

* Table to be used for all CTCAE Grade 3/4 toxicities, including haematological toxicities occurring in monotherapy studies. Note that asymptomatic Grade 3/4 neutropenia with a duration of < 7 days does not require a dose reduction. Dose reduction will be required for asymptomatic Grade 3/4 neutropenia which lasts for ≥ 7 days, or for a second occurrence of Grade 3/4 neutropenia. Dose reduction should be considered for all cases of CTCAE Grade 3/4 febrile neutropenia.

** For guidance on liver toxicity, please refer to Table 21 of the Investigator's Brochure and to Appendix 12: Actions required in case of increases in liver biochemistry and evaluation of Hy's Law.

23.3.4.4 Evaluation, management & treatment of toxicities

Generally, **Grade 1 or 2** non-haematological and/or haematological toxicities do not require vistusertib dose reductions and should be managed as medically indicated (with or without short dose interruptions, see Section 23.3.4.1 for haematological and non-haematological toxicities) by the treating physician.

Grade 3 and 4 toxicities require dose modifications, temporary treatment interruptions or discontinuation of vistusertib see Section 23.3.4.3.

Specific management guidelines (dose/evaluation/treatment) for the events listed below are described in the sections to follow:

- Stomatitis/oral mucositis/mouth ulcers
- Rash/skin toxicity
- Hyperglycaemia
- Electrolyte changes (including hypokalaemia and hypophosphataemia)
- Interstitial Lung Disease/pneumonitis
- ECG Changes
- Severe fatigue
- Nausea & vomiting
- Diarrhoea
- Decreased appetite
- Renal effects
- Liver function test abnormalities
- Infections
- Haematological findings
- Reproductive organs
- Phototoxicity

23.3.4.4.1 Recommendations for Evaluation and Treatment of Severe Fatigue

Routine clinical work-up to exclude reasons other than the underlying disease and/or vistusertib treatment may be performed, including laboratory analyses to rule out metabolic (acidosis, hyperglycaemia) or cardiac problems.

Please refer to Table 26 for dose modifications required for ≥ Grade 3 fatigue.

23.3.4.4.2 Recommendations for Treatment of Stomatitis/Oral Mucositis/Mouth Ulcers

For mild toxicity (Grade 1), use conservative measures such as non-alcoholic mouth wash or salt water (0.9%) mouth wash several times immediately after drug administration (1-3 hours) and during the day as required until resolution.

For more severe toxicity (Grade 2 or 3), the suggested treatments are topical analgesic mouth treatments (i.e., local anesthetics such as benzocaine, butyl aminobenzoate, tetracaine hydrochloride, menthol, or phenol), with or without topical corticosteroids, such as triamcinolone oral paste 0.1% (e.g., Kenalog in Orabase®) or alcohol free 0.5 to 2 mg/5 mL dexamethasone oral solution (e.g. Dexsol®). **Most importantly,** patients must be instructed to swish and expectorate the mouth rinse to avoid systemic exposure to dexamethasone. Agents containing hydrogen peroxide, iodine, and thyme derivatives may worsen mouth ulcers. It is preferable to avoid these agents.

For Grade 3 stomatitis/oral mucositis/mouth ulcers, systemic pain killers (e.g. oral or subcutaneous morphine) are indicated and dose modification as described in Table 26.

23.3.4.4.3 Recommendations for Treatment of Rash/Skin toxicity

Early identification and intervention is critical for the optimal management of rash. Preliminary clinical evidence suggests, that antihistminergic drugs may ameliorate occurence/severity of rash. Therefore patients, who are developing Grade 1 or 2 changes in their skin conditions should be treated with the local Investigator’s choice of antihistaminergic drugs, over the counter moisturising cream or ointment, local antihistamines and/or topical or systemic steroids. If bacterial infection is suspected, local and/or systemic antibiotics may be added (non sedative antihistamines may be prefered due to side effects of fatigue, known for vistusertib).

For Grade 3 rash, topical and/or systemic steroids with or without topical and/or systemic antibiotics (to be considered if bacterial infection is suspected) are indicated, together with dose modifications as described in Table 26. Short courses (≤14 days) of corticosteroid treatment at doses that do not exceed 100 mg per day of prednisone or equivalent may be given.

Some example treatments are listed below:

- Topical steroids: triamcinolone acetonide 0.025%; desonide 0.05%; fluticasone proprionate 0.05%, aclometasone 0.05%
- Topical antipruritics: pramoxine 1%; doxepin 5% cream
- Oral antihistamines: loratidine, cetirizine, fexofenadine; diphenhydramine 25-50 mg every 8h; hydroxyzine 25 mg every 8h
- Topical antibiotics: clindamycin 1-2%; erythromycin 1-2%; metronidazole 1%; silver sulphadiazine1%
- Oral antibiotics: doxycycline 100 mg BD; minocycline 100 mg BD; oxytetracycline 500 mg

23.3.4.4.4 Recommendations for Treatment of Hyperglycaemia

In general management of hyperglycaemia should be performed according to local standards at the discretion of the local Investigator.

Due to the predicted short half-life of vistusertib, only a short period of hyperglycaemia with insulin resistance might be expected. Therefore early treatment with insulin and/or oral anti-diabetes medication should be carefully evaluated and blood sugars and hypokalaemia monitored as per standard clinical practice. If blood glucose levels are < 250 mg/dl (Grade 2), generally no medical treatment is required. Dietary modification may be initiated.

For ≥ Grade 3 hyperglycaemia, dose modifications are required, see Table 26.

23.3.4.4.5 Recommendations for Evaluation and Treatment of Electrolyte changes including Hypokalaemia and Hypophosphataemia

Vistusertib, like other mTOR inhibitors, inhibits pump mechanisms in renal tubules leading to hypokalaemia and hypophosphataemia in a small proportion of patients. The presence of biochemical abnormalities should be monitored as per the protocol and electrolyte abnormalities should be corrected using oral supplements. The Investigator should also consider whether other medication the patient may be receiving, such as diuretics may have contributed to these abnormalities.

23.3.4.4.6 Recommendations for Evaluation and Treatment of Interstitial Lung Disease

If required, a high resolution CT scan will be performed for retrospective analysis and comparison with the baseline CT of the thorax when symptoms occur during trial conduct.

Should a patient experience any new respiratory symptoms including cough, dypsnoea, lower respiratory tract infections not clearly explained by other factors such as disease progression or anaemia, a high resolution CT scan and pulmonary function tests should be performed, including 3 forced expiratory volumes, forced vital capacity, and carbon monoxide diffusing capacity (DLCO% & DLCO). A recent haemoglobin measurement should also be available at the time of the DLCO evaluation. If these investigations are suggestive of pneumonitis or interstitial lung disease and causality with the study drug cannot be excluded, treatment should be interrupted. In more severe cases treatment with corticosteroids should be considered as per reference Willemsen et al 2016.

23.3.4.4.7 Recommendations for Evaluation and Management of ECG Changes

Patients who develop persistent, confirmed T wave repolarization abnormalities (inversion or flattening) on regularly scheduled ECGs may be referred for a cardiology opinion and should have the following assessments performed:

- ECHO or MUGA as clinically indicated and end of trial and/or safety follow up visit if any abnormal LV function was diagnosed.
- ECG at safety follow-up visit.
- Troponin measurement as clinically indicated and at end of trial.

23.3.4.4.8 Recommendations for Treatment of Nausea and Vomiting

Not all patients require antiemetics and therefore they should not be given prophylactically. However, once a patient has experienced nausea and vomiting, serotonin (5-HT3) antagonists should be administered on subsequent vistusertib dosing days, e.g.:

- Dolasetron 100 mg by mouth daily
- Granisetron 2 mg by mouth daily or 1 mg by mouth BD

Aprepitant should **not** be used as it is a moderate CYP3A4/5 inhibitor.

Please refer to Table 26 for dose modifications required for ≥ Grade 3 nausea and/or vomiting.

If nausea and vomiting are not being managed with the regimen above, start a breakthrough treatment with the addition of one agent of a different drug class, e.g.:

- Dexamethasone 8 mg by mouth at day 1 of the vistusertib dosing period, or
- Metoclopramide 10-40 mg by mouth, or
- Olanzapine 5-10 mg by mouth, or
- Promethazine (Phenergan) 12.5-25 mg every 6 hours

on the vistusertib dosing days prior to dose.

If this is still not managing the nausea and vomiting sufficiently, add Lorazepam 0.5-2 mg by mouth or sublingual every 4-6 hours as needed, but only on the vistusertib dosing days.

Should upper abdominal pain develop, a H2 blocker or proton pump inhibitor can be added.

23.3.4.4.9 Recommendations for Management and Treatment of Diarrhoea

Patients should be made aware of the risk of diarrhoea while receiving treatment with vistusertib. Patients should be advised to drink sufficient fluids and have a supply of loperamide available throughout treatment; however, loperamide should not be administered prophylactically.

As soon as the first liquid stool occurs, patients should start treatment with loperamide immediately and also take electrolyte-containing fluids, and inform the study team. Loperamide should be administered as per package information and usual clinical practice. It should not be administered for more than 48 consecutive hours.

Hospitalisation is recommended for management of diarrhoea under the following circumstances:

- Diarrhoea associated with fever
- Diarrhoea requiring intravenous hydration
- Diarrhoea persisting beyond 48 hours following the initiation of high-dose loperamide.

Please refer to Table 26 for dose modifications required for ≥ Grade 3 diarrhoea.

23.3.4.4.10 Recommendations for Treatment of Decreased Appetite

Decreased appetite should be treated according to local practice. Dietary review is recommended.

23.3.4.4.11 Recommendations for Evaluation and Treatment of Renal effects

Renal effects have been classified as a potential risk for vistusertib. However, no clinically significant renal findings were reported at any dose of vistusertib so far.

Clinical study protocols exclude patients with impaired renal function or concurrent specific renal disease from participation in studies of vistusertib.

If ≥ Grade 3 renal dysfuction develops while the patient is on study, follow dose modification advice shown in Table 26.

23.3.4.4.12 Recommendations for Evaluation and Treatment of Liver function tests abnormalities

Evidence of abnormal liver function should be monitored as per the Schedule of Assessments. Increased levels of AST, ALT, or serum bilirubin should trigger an investigation of the cause

which may include viral infection or disease progression with liver infiltration. The local Investigator should consider whether the abnormal liver function meets the criteria for expedited reporting (see Section 11.1.2.2 Hy's Law cases).

Vistusertib is metabolised in the liver. For subjects who develop mild liver impairment while on study (Child-Pugh Class A), the recommended dose for vistusertib is 2 dose levels lower than the starting dose (see Table 24: Vistusertib Dose Modifications). If 2 dose levels lower than the starting dose is not available, vistusertib should be discontinued. Subjects who develop moderate or severe hepatic impairment (Child-Pugh Class B or C) must hold study drug until resolved to mild impairment (Child-Pugh Class A) or better and will be re-treated at 2 dose levels lower than the starting dose for vistusertib (or discontinued if applicable) (see Table 24).

23.3.4.4.13 Recommendations for Evaluation and Treatment of Infections

Patients receiving treatment with vistusertib may be at an increased risk of infection. This should be managed as per clinical practice. In case of Grade 3 infections, the guidelines provided in Table 26 should be followed.

23.3.4.4.14 Recommendations for Evaluation and Treatment of Haematological Findings

See Table 25 and Table 26 for dose modifications required for Grade 3 and 4 haematological toxicities.

For vistusertib in monotherapy or in combination with agents which do not affect the bone marrow:

- No specific monitoring measures must be put in place beside standard monitoring in clinical trials, including regular blood checks as per protocol and if findings should be reported, blood checks should be amended accordingly.
- In general treatment with prophylactic haematopoetic growth factors is not allowed and should only be permitted during drug holidays as per Food and Drugs Administration (FDA) guidance (Smith et al 2006).

23.3.4.4.15 Reproductive organs:

Reproductive toxicity has been classified as an important potential risk for vistusertib.

Any reports of pregnancy in patients, or partners of patients will be followed up as described in the study protocols. Contraceptive measures during treatment with vistusertib are summarised in Section 23.2.3.7.

23.3.4.4.16 Potential for Phototoxicity

Phototoxicity has been classified as a potential risk for vistusertib; however there have been no relevant clinical findings so far. Sunlight protection measures, including sun glasses should be adopted during treatment with vistusertib.

23.4 Reference List

- Carretero J, Medina PP, Blanco R *et al.* (2007). Dysfunctional AMPK activity, signalling through mTOR and survival in response to energetic stress in LKB1-deficient lung cancer. *Oncogene* 26(11):1616-25.
- Gao Y, Xiao Q, Ma H *et al.* (2010). LKB1 inhibits lung cancer progression through lysyl oxidase and extracellular Matrix remodelling. Proc Natl Acad Sci U S A.107(44):18892-7.
- Gill RK, Yang SH, Meerzaman D *et al.* (2011). Frequent homozygous deletion of the LKB1/STK11 gene in non-small cell lung cancer. *Oncogene* 30(35):3784-91.
- Guichard SM, Howard Z, Heathcote D *et al.* (2012). AZD2014, a dual mTORC1 and mTORC2 inhibitor is differentiated from allosteric inhibitors of mTORC1 in ER+ breast cancer2. Aims, Objectives & Outcome Measures. AACR, 2012 Abstract 917
- Koivunen JP, Kim J, Lee J *et al.* (2008). Mutations in the LKB1 tumour suppressor are frequently detected in tumours from Caucasian but not Asian lung cancer patients. *Br J Cancer* 99(2):245-52.
- Liang MC, Ma J, Chen L *et al.* (2010). TSC1 loss synergizes with KRAS activation in lung cancer development in the mouse and confers rapamycin sensitivity. *Oncogene* 29(11):1588-97.
- Okon IS, Coughlan KA & Zou MH (2014). Liver kinase B1 expression promotes phosphatase activity and abrogation of receptor tyrosine kinase phosphorylation in human cancer cells. *J Biol Chem* 289(3):1639-48.
- Smith TJ, Khatcheressian J, Lyman GH et al. Update of Recommendations for the use of white blood cell growth factors: an evidence-based clinical practice guideline. American Society of Clinical Oncology 2006; 24: 3187-3205.
- Treilleux I, Arnedos M, Cropet C (2013). Predictive markers of everolimus efficacy in hormone receptor positive (HR+) metastatic breast cancer (MBC): Final results of the TAMRAD trial translational study. *J Clin Oncol* 31(suppl; abstr 510).
- Turina M, Christ-Crain M & Polk Jr HC (2006). Diabetes and hyperglycaemia: Strict glycaemic control. *Crit Care Med* 34(9):S291-30.
- Willemsen A.E.C.A.B, Grutters J.C, Gerritsen W.R, van Erp N.P, van Herpen C.M.L and Tol J. mTOR inhibitor-induced interstitial lung disease in cancer patients: Comprehensive review and a practical management algorithm. Int. J. Cancer: 138, 2312–2321 (2016)

24 ARM C: PALBOCICLIB – CDK4/6 INHIBITOR

24.1 Background & Rationale

Lead Investigator: Professor Gary Middleton

24.1.1 Molecular Cohorts

Inhibitor: Palbociclib

Arm	Investigational Medicinal Product	Cohort Number	NSCLC Histology	Molecular Cohort
C	Palbociclib – CDK4/6 Inhibitor	C1	Squamous cell carcinoma (SCC)	p16 (CDKN2A) loss of function with proficient Rb (no loss of Rb function either by mutation or deletion)
		C2<br>*Closed to recruitment*	Adenocarcinoma (ADC) or not otherwise specified (NOS) NSCLC	p16 (CDKN2A) loss of function with proficient Rb (no loss of Rb function either by mutation or deletion)
		C3	NSCLC	CDK4 amplification with proficient Rb (no loss of Rb function either by mutation or deletion)
		C4	NSCLC	CCND1 amplification with proficient Rb (no loss of Rb function either by mutation or deletion)
		C5	NSCLC	• STK11/LKB1 mutation, STK11/LKB1 homozygous deletion or TSC1 mutation or TSC2 mutation AND<br>• activated KRAS/MAPK pathway i.e. concomitant KRAS, NRAS or NF1 mutation AND<br>• Proficient Rb (no loss of Rb function either by mutation or deletion)
		C6<br>*Closed to recruitment*	NSCLC	KRAS mutation with proficient Rb (no loss of Rb function either by mutation or deletion)<br><br>(No concomitant STK11/LKB1 mutation or deletion, no PIK3CA mutation or amplification, no PTEN mutation or homozygous deletion, no AKT mutation, no EGFR mutation, no FGFR2/3 mutation, no TSC1/2 mutation and no HER2 mutation.<br>Any TIER1 or TIER2 aberrations will exclude the patient from this cohort. Some TIER3 aberrations may also exclude the patients; these will be checked on a case by case basis by the National Lung Matrix Trials Office with CRUK and Pfizer.)

24.1.2 Pre-Clinical rationale

24.1.2.1 Inhibition of CDK4/CDK6 and Enzyme Selectivity

Palbociclib is a highly selective inhibitor of CDK4/cyclinD1 kinase activity (IC_{50} = 11 nM; K_i = 2 nM. Palbociclib has selectivity for CDK4/6, with little or no activity against a large panel of 34 other protein kinases including other CDKs and a wide variety of tyrosine and serine/threonine kinases (Fry *et al*. 2004). CDK6, another enzyme that also complexes with cyclin-D subunits, is also commonly expressed in mammalian cells and tumours. CDK6 is highly homologous to CDK4 and can perform the same function by phosphorylating Rb, thus potentially creating a redundant mechanism to promote cell cycle progression. Consequently, inhibition of both enzymes is necessary to ensure complete suppression of Rb phosphorylation and the greatest possible spectrum of antitumour activity. Results indicate that palbociclib inhibits CDK6 with equivalent potency to CDK4.

Table 27: Palbociclib - Inhibition of Cyclin-Dependent Kinases

CDK	IC_{50} (µM)[a]	K_i (µM)[b]
CDK4/cyclinD1	0.011	0.002
CDK4/cyclinD3	0.009	0.001
CDK6/cyclinD2	0.015	ND
CDK2/cyclinA	>5	ND
CDK1/cyclinB	>5	ND
CDK5/p25	>5	ND

IC_{50} = 50% inhibitive concentration; K_i = inhibitor constant; ND = not determined.
a Concentration of palbociclib necessary to inhibit activity by 50%.
b Kinetic inhibition constant calculated by tight binding inhibition analysis.

24.1.2.2 Inhibition of Retinoblastoma Phosphorylation in Tumour Cells

The only known natural substrate for CDK4/cyclinD1 is the retinoblastoma gene product; Rb. Specific CDK4 phosphorylation sites on Rb include serine-780 and serine-795. Therefore, the phosphorylation status of Rb at these specific sites in treated tumours can serve as an appropriate biomarker for target modulation by palbociclib. The IC_{50} for reduction of Rb phosphorylation at serine-780 in the MDA-MB-435 breast carcinoma cell line was 0.066 µM. Palbociclib was equally effective at reducing Rb phosphorylation at serine-795in this tumour cell line with an IC_{50} of 0.063 µM. Similar effects on serine-780 and serine-795 phosphorylation were obtained in the Colo-205 colon carcinoma cell line.

24.1.2.3 Anti-proliferative Effects of Palbociclib

Palbociclib inhibits cellular proliferation and prevents cellular DNA synthesis by preventing cells from entering S phase of the cell cycle. Palbociclib inhibited thymidine incorporation into the DNA of a panel of Rb-positive human breast, colon, and lung carcinomas, with IC_{50} values ranging from 0.040 to 0.17 µM. Palbociclib was also effective in preventing cell cycle progression in human leukaemias and in non-transformed human epithelial cells and fibroblast and was equally effective in suppressing cell division in human tumour cell lines.

A selective CDK4/cyclinD inhibitor should cause a specific accumulation of cells in G1, but have no effect on other phases of the cell cycle, in which cells should continue to progress and eventually decline in number. MDA-MB-453 breast carcinoma cells that were exposed to various concentrations of palbociclib for 24 hours show a significant increase in the percentage

of cells in G1 in the presence of as little as 0.04 µM palbociclib with a concomitant decline in other phases of the cell cycle.

Finally, to provide further evidence of the selectivity of palbociclib, the compound was tested against Rb-negative tumour cells, which should not be sensitive to a specific CDK4 inhibitor. Palbociclib was tested against the MDA-MB-468 human breast carcinoma and the H2009 human non-small cell lung carcinoma, both of which have deleted Rb. The compound had no anti-proliferative activity on these cells when assayed at 3 µM (highest concentration tested), which is 1 to 2 orders of magnitude higher than the concentration necessary to inhibit Rb-positive tumour cells.

24.1.2.4 Additional Nonclinical Breadth of Efficacy Studies

Published reports demonstrate palbociclib produced a potent G1 cell cycle arrest and induced senescence in a panel of 16 Rb+ glioblastoma (GBM) cell lines, whereas 5 Rb- cell lines were resistant (Michaud, et al, 2010). shRNA knockdown of Rb expression conferred resistance of GBM cells to palbociclib. Palbociclib was effective against intracranial GBM xenograft tumors, including those that had recurred after temozolomide therapy. Palbociclib combined favourably with radiation against GBM xenografts (Michaud, et al, 2010).

A comprehensive survey of molecular alterations was conducted in 207 patient samples representing seven major subtypes of soft tissue sarcoma (Barretina, et al, 2010). Among prominent alterations discovered were mutations in PIK3CA, TP53, and NF1, along with a complex pattern of amplification of chromosome 12q in 90% of dedifferentiated liposarcomas (DDPLS). The most strongly amplified gene among these 12q amplifications in DDLPS was CDK4. shRNA knockdown of CDK4 inhibited proliferation of two DDLPS cell lines, LPS141 and DDLS8817, as did treatment with palbociclib.

24.1.2.5 In Vivo Target Suppression and Antitumour Efficacy

Palbociclib exhibits significant antitumour efficacy against multiple human tumour xenograft models. The Colo-205 model is exquisitely sensitive to palbociclib. At doses as low as 12.5 mg/kg, a 13-day growth delay was obtained, indicating a 90% inhibition of tumour growth rate. Palbociclib was inactive against the H23 lung and the SW-620 colon carcinomas. The lack of response may be associated with the presence of oncogenic K-RAS mutations in SW-620 and H23; none of the xenografts sensitive to palbociclib had such mutations.

Further evidence that the anti-tumour activity observed in Rb-positive tumours is due to inhibition of CDK4/CDK6 protein kinase activity was obtained by testing palbociclib in the MDA-MB-468 breast carcinoma and the DU-145 prostate tumour models. These are Rb-negative tumours; neither of which responded to this compound. The lack of efficacy in Rb-negative tumours is consistent with the lack of anti-proliferative activity observed *in vitro*. Taken together, these results support the proposed mechanism of palbociclib (inhibition of CDK4/6-mediated Rb phosphorylation) and the specificity of the compound demonstrated in enzyme activity tests.

24.1.2.6 <u>Schedule Dependence Studies for Palbociclib</u>

Further studies investigated whether continuous daily dosing of palbociclib was needed for optimal efficacy. Four dosing schedules were employed against the MDA-MB-435 breast carcinoma model over 14 days of treatment, including continuous daily, every other day, every third day, and 3 courses of 3 days dosing followed by 4-day drug holidays. The design of this experiment was such that the total compound administered over the 2-week period was identical for each treatment schedule. The results show that a similar degree of efficacy was attained with all schedules, implying that an intermittent regimen is feasible without

compromising activity. Similar experiments were conducted against the Colo-205 colon carcinoma model. Again, intermittent schedules were as efficacious as daily dosing, with tumour regressions occurring during all dosing regimens.

24.1.2.7 Potential to Develop Acquire Resistance to Palbociclib

During the 14-day treatment period employed for most of the efficacy experiments, no cures were documented, and the tumours grew back after therapy. It is possible that a tumour variant had selectively grown back and acquired resistance to the compound. To address this possibility, Colo-205 colon tumours that had initially significantly regressed in response to treatment with palbociclib were harvested and reimplanted into naive mice. After the tumours grew to 100 to 150 mg, these tumour-bearing mice were treated with palbociclib with a dose and dosing schedule identical to the original experiment. The tumours responded with equal sensitivity to the drug and fully regressed, indicating that no resistance had developed during the initial treatment. A similar result was observed with retreated MDA-MB-435 tumours.

24.1.2.8 Reduction of Tumour Retinoblastoma Phosphorylation in Vivo and Correlation with Efficacy

In parallel with the *in vivo* efficacy tests, tumours were harvested for PD analysis to ensure that anti-tumour activity correlated with modulation of the target and to provide confidence that the proposed biomarker would predict for efficacy. Efficacious and non-efficacious doses of palbociclib were given to mice bearing the MDA-MB-435 breast carcinoma and the phosphorylation status of serine-780 on Rb in tumour tissue was monitored over time. The results show that while all doses caused a reduction in the biomarker shortly after drug administration, phosphorylation returned at the non-efficacious doses (12.5 and 37.5 mg/kg) over the 24-hour interval before the next dose. However, the highly efficacious dose of 150 mg/kg suppressed Rb serine-780 phosphorylation during the full 24-hour period. These data suggest that complete suppression of Rb phosphorylation needs to be maintained between drug doses to achieve significant efficacy against this particular tumour model. Because the lower doses result in transient target modulation, more frequent dosing at these doses might result in improved efficacy by maintaining suppression of phosphorylation.

Similar experiments with the Colo-205 colon carcinoma xenografts, which are exquisitely sensitive to palbociclib, shows that complete suppression of Rb phosphorylation between doses was found to be unnecessary for producing growth inhibition. However, for maximal effects against Colo-205 tumours (i.e. regression), total inhibition had to be maintained. Comparing results of the efficacy experiments between the highly sensitive colon tumour and the moderately sensitive breast tumour reveals a 7 to 8-fold difference in the dose necessary to produce comparable efficacy. The PD results show a similar difference between the 2 tumours in dose requirements necessary to suppress serine-780 phosphorylation on Rb, indicating that efficacy correlates with modulation of this biomarker.

Ki-67, a common marker of cell proliferation, was monitored in xenografts by immunohistochemical staining with the MIB-1 antibody. MDA-MB-435 and Colo-205 xenografts were treated with an efficacious but nontoxic dose of palbociclib (130 mg/kg) daily for 7 and 4 days, respectively. Tumour samples were harvested at various times after the last dose and analysed for Rb phosphorylation and Ki-67 expression. Relative to vehicle-treated controls, Rb phosphorylation and Ki-67 expression were both strongly inhibited from 1 to 24 hours after dosing. At 48 and 72 hours after the last dose, Rb phosphorylation and Ki-67 returned to control levels in MDA-MB-435. In Colo-205, Rb phosphorylation and Ki-67 expression remained partly suppressed through 72 hours. These results are consistent with the anti-proliferative mechanism expected for a specific Cdk4/6 inhibitor. They also provide support for the use of Ki-67 as a biomarker of cell proliferation in conjunction with palbociclib.

24.1.3 Clinical data

24.1.3.1 Patient Exposure

As of 31 August 2016, 35 studies evaluating the safety, efficacy, pharmacodynamics and pharmacokinetics of palbociclib as a single agent or in combination have started.

24.1.3.2 Pharmacokinetics

The pharmacokinetics of palbociclib were characterised in patients with solid tumors including advanced breast cancer and in healthy subjects. The mean Cmax of palbociclib is generally observed between 6 to 12 hours (time to reach maximum concentration, Tmax) following oral administration. The mean absolute bioavailability of palbociclib after an oral 125 mg dose is 46%. In the dosing range of 25 mg to 225 mg, the AUC and Cmax increased proportionally with dose in general. Steady state was achieved within 8 days following repeated once daily dosing. With repeated once daily administration, palbociclib accumulated with a median accumulation ratio of 2.4 (range 1.5-4.2).

Food effect: Palbociclib absorption and exposure were very low in approximately 13% of the population under the fasted condition. Food intake increased the palbociclib exposure in this small subset of the population, but did not alter palbociclib exposure in the rest of the population to a clinically relevant extent. Therefore, food intake reduced the intersubject variability of palbociclib exposure, which supports administration of palbociclib with food. Compared to palbociclib given under overnight fasted conditions, the population average AUCinf and Cmax of palbociclib increased by 21% and 38%, respectively, when given with high-fat, high-calorie food (approximately 800 to 1000 calories with 150, 250, and 500 to 600 calories from protein, carbohydrate and fat, respectively), by 12% and 27%, respectively, when given with low-fat, low-calorie food (approximately 400 to 500 calories with 120, 250, and 28 to 35 calories from protein, carbohydrate and fat, respectively), and by 13% and 24%, respectively, when moderate-fat, standard calorie food (approximately 500 to 700 calories with 75 to 105, 250 to 350 and 175 to 245 calories from protein, carbohydrate and fat, respectively) was given one hour before and two hours after palbociclib dosing. Binding of palbociclib to human plasma proteins *in vitro* was approximately 85%, with no concentration dependence over the concentration range of 500 ng/mL to 5000 ng/mL. The geometric mean apparent volume of distribution (Vz/F) was 2583 L (26% CV). *In vitro* and *in vivo studies* indicated that palbociclib undergoes hepatic metabolism in humans. Following oral administration of a single 125 mg dose of [14C]palbociclib to humans, the primary metabolic pathways for palbociclib involved oxidation and sulfonation, with acylation and glucuronidation contributing as minor pathways. Palbociclib was the major circulating drug-derived entity in plasma (23%). The major circulating metabolite was a glucuronide conjugate of palbociclib, although it only represented 1.5% of the administered dose in the excreta. Palbociclib was extensively metabolized with unchanged drug accounting for 2.3% and 6.9% of radioactivity in faeces and urine, respectively. In faeces, the sulfamic acid conjugate of palbociclib was the major drug-related component, accounting for 26% of the administered dose. *In vitro* studies with human hepatocytes, liver cytosolic and S9 fractions, and recombinant SULT enzymes indicated that CYP3A and SULT2A1 are mainly involved in the metabolism of palbociclib.

The geometric mean apparent oral clearance (CL/F) of palbociclib was 63.1 L/hr (29% CV), and the mean (± standard deviation) plasma elimination half-life was 29 (±5) hours in patients with advanced breast cancer. In 6 healthy male subjects given a single oral dose of [14C]palbociclib, a median of 91.6% of the total administered radioactive dose was recovered in 15 days; faeces (74.1% of dose) was the major route of excretion, with 17.5% of the dose recovered in urine. The majority of the material was excreted as metabolites. Based on a population pharmacokinetic analysis in 183 patients with cancer (50 male and 133 female patients, age range from 22 to 89 years, and body weight range from 37.9 to 123 kg), gender

had no effect on the exposure of palbociclib, and age and body weight had no clinically important effect on the exposure of palbociclib.

24.1.3.3 Clinical Efficacy

Study 1 was a randomised, open-label, multicentre study of palbociclib plus letrozole versus letrozole alone conducted in postmenopausal women with ER-positive, HER2-negative advanced breast cancer who had not received previous systemic treatment for their advanced disease. A total of 165 patients were randomized in Study 1. Randomization was stratified by disease site (visceral versus bone only versus other) and by disease-free interval (>12 months from the end of adjuvant treatment to disease recurrence versus ≤12 months from the end of adjuvant treatment to disease recurrence or de novo advanced disease). Palbociclib was given orally at a dose of 125 mg daily for 21 consecutive days followed by 7 days off treatment. Patients received study treatment until progressive disease, unmanageable toxicity, or consent withdrawal.

Patients enrolled in this study had a median age of 63 years (range 38 to 89). The majority of patients were Caucasian (90%) and all patients had an Eastern Cooperative Oncology Group (ECOG) performance status (PS) of 0 or 1. Forty-three percent of patients had received chemotherapy and 33% had received antihormonal therapy in the neoadjuvant or adjuvant setting prior to their diagnosis of advanced breast cancer. Forty-nine percent of patients had no prior systemic therapy in the neoadjuvant or adjuvant setting. The majority of patients (98%) had metastatic disease. Nineteen percent of patients had bone only disease and 48% of patients had visceral disease.

The major efficacy outcome measure of the study was Investigator-assessed PFS evaluated according to Response Evaluation Criteria in Solid Tumors Version 1.0 (RECIST). Consistent results were observed across patient subgroups of, disease-free interval, disease site and prior therapy. The treatment effect of the combination on PFS was also supported by a retrospective independent review of radiographs with an observed hazard ratio (HR) of 0.621 (95% CI: 0.378, 1.019). Overall response rate in patients with measurable disease as assessed by the Investigator was higher in the palbociclib plus letrozole compared to the letrozole alone arm (55.4% versus 39.4%). At the time of the final analysis of PFS, overall survival (OS) data was not mature with 37% of events.

24.1.3.4 Marketing Experience

Palbociclib has been authorized for marketing in the U.S. On 03 February 2015 the U.S. Food and Drug Administration (FDA) approved IBRANCE® (palbociclib) capsules in combination with letrozole for the treatment of postmenopausal women with oestrogen receptor (ER)-positive, human epidermal growth factor receptor 2 (HER2)-negative advanced breast cancer as initial endocrine-based therapy for their metastatic disease. This indication is approved under accelerated approval based on progression-free survival (PFS).

On 19 February 2016, the US FDA granted full approval to palbociclib in combination with fulvestrant (with or without goserelin) for the treatment of women with hormone receptor-positive, HER2-negative advanced breast cancer whose disease progressed after prior endocrine therapy based on the favourable benefit/risk profile observed in Study 1023.

On 9 November 2016, the EU Commission Decision was issued for IBRANCE for the following indications:

IBRANCE is indicated for the treatment of hormone receptor (HR)-positive, human epidermal growth factor receptor 2 (HER2)-negative locally advanced or metastatic breast cancer:

- in combination with an aromatase inhibitor;

- in combination with fulvestrant in women who have received prior endocrine therapy.

In pre- or peri-menopausal women, the endocrine therapy should be combined with a luteinizing hormone-releasing hormone (LHRH) agonist.

Additionally, IBRANCE is approved in more than 50 countries globally.

24.1.4 Cohort definition

See Table 28 for the specific aberration rules for Arm C cohorts.

24.1.4.1 C1 - Squamous cell lung cancer with proficient Rb and p16 (CDKN2A) loss

24.1.4.2 C2 – Adenocarcinoma or NOS NSCLC with proficient Rb and p16 (CDKN2A) loss

The only known natural substrate for CDK4/6 kinase activity is the Rb protein. Thus, tumours that do not express Rb are not expected to respond to palbociclib. In most common tumours, however, other genetic and epigenetic changes lead to increases in CDK4/6 activity contributing to tumour cell growth. P16INK4A negatively regulates assembly of active CDK4/6-cyclin D complexes. Recently published TCGA data has shown a high frequency of inactivation of CDKN2A the gene that encodes p16 in squamous cell carcinoma of the lung (TCGA 2012). Loss of p16 expression was seen in tumours with homozygous deletion of CDKN2A (29% of cases) and epigenetic silencing by methylation (18% of cases). 18% harboured CDKN2A mutations but low p16 expression was relatively infrequent in this group: 45% of the cases with high p16INK4 and ARF expression had CDKN2A mutations. Only occasional cases of p16 loss had Rb inactivation but given these occur a combined p16 and Rb score seems appropriate. In an extensive panel of ovarian cancer cell lines (where the commonest mechanism for CDKN2A inactivation is either homozygous loss or epigenetic silencing) Rb proficient cells with low p16 expression (by message or protein expression) were the most sensitive to palbociclib and no other analysed biomarkers pertaining to CDK4/6 signalling were informative (Konecny *et al.* 2011). Cells with high expression of p16 were resistant. Expression profiles of cells classified by response to palbociclib demonstrated 117 differentially expressed genes between sensitive and resistant lines: CDKN2A was the most significant gene. P53 mutation appeared to be a resistance marker underscoring the importance of its analysis in patients selected for therapy with the drug. Palbociclib caused G1 arrest as expected in sensitive cell lines with no lethality suggesting primarily a cytostatic mode of action. Given this very strong proof of principle that cancer cells which lack p16 expression with have proficient Rb are those that are likely to be most sensitive to the cytostatic effects of palbociclib and the high frequency of p16 loss in squamous cell cancer principally as a result of homozygous deletion or epigenetic silencing, this cohort is a very obvious one to test the impact of palbociclib in. Homozygous deletion of CDKN2A is the most significant deletion in both oncogene positive and negative adenocarcinoma of the lung, with an overall frequency in the TCGA dataset of 19.6% (TCGA, 2014). It is independent of age, sex and smoking history (Iwakawa *et al.* 2008). It appears to be an early event during progression of adenocarcinoma of the hung and occurs with equal frequency in those with and without EGFR, KRAS and p53 mutations. It is present in around half of the recently described KC variant of KRAS mutant adenocarcinoma, with an overall prevalence of 15.6% in KRAS mutant disease (Skoulidis *et al.* 2015). KC tumours are typically TTF-1 negative and of invasive mutinous histology with low levels of mTORC1 activation. Explicitly testing the activity of palbociclib in adenocarcinoma of the lung is important as the Master Protocol in the US is testing it only in patients with squamous cell lung cancer. The presence of concomitant mutations will not be a barrier to testing in this cohort as the pattern and type of these may be key predictive biomarkers in this cohort.

24.1.4.3 C3 – NSCLC harbouring CDK4 amplifications and proficient Rb

24.1.4.4 C4 - CCND1 amplified NSCLC and proficient Rb

CDK4 amplification occurring as a relatively discrete event with little other evidence of accompanying gene rearrangement by CGH was first described in 2005 where a rate of 4.3% was detected (Wikman *et al.* 2005): amplification was associated with increased CDK4 message and protein expression as would be expected. In the most recent iteration of the TCGA (using GISTIC methodology) 0/178 squamous cell cancers harboured CDK4 amplifications but 7% adenocarcinoma were amplified.

Well differentiated/dedifferentiated liposarcoma (WDLS/DDLS) is the paradigm malignancy characterised by CDK4 amplification and palbociclib has demonstrated growth inhibition in WDLS/DDLS cells *in vitro* and in xenograft models. Proof of principle that targeting CDK4 amplified cancer with palbociclib is important therapeutically came in a phase II trial of palbociclib in WDLS/DDLS (Dickson *et al.* 2013). All patients had received at least one previous line of therapy. Primary end-point was 12 week PFS: on the basis of historical data a PFS > 40% at this point was considered promising. In 29 evaluable patients the 12 week PFS was 66% and median PFS 17.9 weeks. 1 patient responded and 3 others had a decrease in tumour size of greater than 10%. These responses were very gradual occurring over many months: stabilisation rather than response is characteristic of a cell cycle inhibitor.

Cyclin D1 forms a complex with CDK4/6 and as such the biological sequelae of CCND1 and CDK4 amplification would be expected to be similar. In a census of amplified and overexpressed genes in cancer, CCND1 was one of only 14 class III genes (significant evidence) implicated in NSCLC (Santarius *et al.* 2010). In the TCGA dataset CCND1 amplification was seen in 5% adenocarcinoma and 12% squamous cell, although the latter often occur in association with other abnormalities of the p16/CDK4/Rb pathway. Whilst there is no data that we are aware of data analysing the pre-clinical or clinical activity of palbociclib in CCND1 amplified NSCLC, cyclin D1 amplification is clearly a pertinent target for this drug. CCND1 amplification is seen in up to 20% breast cancer and is more associated with the ER+ sub-type. In a panel of breast cancer cell lines luminal ER+ sup-type was the most sensitive to Palbociclib and cyclin D1 was over-expressed in the most sensitive cell lines (Finn *et al.* 2009). The t(11;14)(q13;q32) translocation is characteristic of mantle cell lymphoma (MCL) driving high levels of cyclin D1 expression, the majority of which is found complexed to CDK4. Significant reductions in positron emission tomography (PET) standardised uptake values (SUV), pRB expression and Ki-67 staining was seen in the majority of 17 MCL patients treated with palbociclib (Leonard *et al.* 2012). 5 patients were progression free at 12 months and 3 patients achieved an objective response including one complete response.

24.1.4.5 C5 – STK11/LKB1 mutation or homozygous deletion, TSC1/2 mutation, proficient Rb and activated KRAS/MAPK de-regulation pathway in NSCLC

Mutation, deletion and under-expression of STK11/LKB1 is commonly associated with KRAS mutation and is a feature of the proximal proliferative transcriptional subtype (TCGA, 2012). Concomitant mutation of Kelch-like ECH-associated protein 1 (KEAP1) is also a feature of this expression subtype. STK11/LKB1 expression loss as determined immunohistochemically was seen in 30% of KRAS mutant NSCLC most commonly in smokers (35%) and in tumours with KRAS transversions (Calles, 2015). Patients harbouring double mutant tumours presented with a greater number of involved metastatic sites and a higher incidence of extrathoracic metastases and brain metastases compared with patients with KRAS mutant tumours without concomitant STK11/LKB1 loss. There was a trend towards inferior survival in double mutated patients. These double mutant tumours have been classified as the KL subtype of KRAS mutant NSCLC with frequent co-mutation of KEAP1 as previously described and additionally ATM (Skoulidis, 2015). As to be expected from the mutation of KEAP1, KL tumours were

associated with an NRF2 mediated anti-oxidant and cell protective programme. KL tumours had low levels of immune infiltration possibly related to increased production of HIF1α related to mTOR activation. Finally KL cell lines demonstrated increased sensitivity to HSP90 inhibition, in contradistinction to the reduced sensitivity to docetaxel/selumetinib demonstrated in double mutant models compared with KRAS models lacking dual STK11/LKB1 mutations (Chen, 2012). The rationale for trialing palbociclib in this additional discrete KRAS mutant cohort (in addition to KRAS mutant NSCLC without AKT activation) is based on preclinical data showing high levels of activity in mouse models using palbociclib in KRAS models with concomitant STK11/LKB1 mutation (personal communication). Patients with dual NF1/ STK11/LKB1 mutations will also be eligible given that NF1 loss phenocopies KRAS mutation. The same will apply to STK11/NRAS double mutants. These patients with double mutant tumours are also eligible for treatment with vistusertib.

24.1.4.6 C6 - KRAS mutations and proficient Rb in NSCLC

There is very strong evidence supporting the testing of palbociclib in KRAS mutant adenocarcinoma of the lung, an abnormality present in 25.8% of cases (TCGA). K-Ras+/LSLG12Vgeo; RERT ert/ert mice treated at weaning with 4OHT develop KRAS driven adenomas and NSCLC which recapitulate the human disease. Activation of mutant KRAS in K-Ras+/LSLG12Vgeo; RERT ert/ert mice MEFs bypasses replicative senescence in these cells and this was restored by ablating any of the interphase CDKs (Puyol *et al*. 2010). Ablation also prevented these cells from growing under serum limiting conditions. Knockdown of CDK2, CDK4 and CDK6 blocked proliferation in 4/5 KRAS mutant containing NSCLC cells. The 5th cell line expressed very low levels of KRAS protein. The 5 cell lines containing wild type KRAS were unaffected by loss of CDK expression. There was a significant reduction in tumour burden in K-Ras+/LSLG12Vgeo; RERT ert/ert;CDK4-/- mice compared to their K-Ras+/LSLG12Vgeo; RERT ert/ert;CDK4+/+ littermates 6 months after mutant KRAS activation and all tumours in the CDK4 null mice were benign lesions. There was a significant reduction in the number of mutant KRAS expressing cells in the lung parenchyma of CDK4 null mice due to the induction of senescence which only occurred with the combination of mutant KRAS expression and CDK4 loss. Strikingly, this response was specific to lung cells as this phenomenon was not seen in other tissues including colorectal and pancreatic which expressed mutated KRAS. Replacement of a CDK4 null allele with a conditional allele allowed ablation of CDK4 after the development of KRAS driven tumours. CDK4 ablation resulted in a significant reduction in the size of the tumours and number of high grade lesions. Reduction of lung CDK4 expression was accompanied by up-regulation of senescence-associated markers and the infiltration of lesions by T cells and granulocytes.

The therapeutic impact of palbociclib was tested in this model. K-Ras+/LSLG12Vgeo; RERT ert/ert mice were treated at weaning with 4OHT and monitored by CT for 4 months. Mice that had not developed tumours at this time were treated with vehicle or palbociclib: 3/4 vehicle treated mice developed CT detected lesions compared with only 2/12 palbociclib treated mice. Mice that had developed detectable lung tumours were also randomised. There was a decrease in the number of tumours and a significant decrease in tumour burden in palbociclib treated animals. PD assays showed that palbociclib diminished Rb phosphorylation.

Table 28: Specific Aberration Rules for Arm C Cohorts.

Drug	Arm	Molecular Cohort	Specific Aberration	Rule
Palbociclib	C1	p16 loss (CDKN2A)/Rb WT<br>(SCC)	For Rb, no mutations that introduce STOP, frameshift, splice variants or missense mutations	Concomitant lack of mutations in Rb (proficient Rb (with no loss of Rb function either by mutation or deletion) and loss of p16
	C2	p16 loss (CDKN2A)/Rb WT<br>(ADC or NOS NSCLC)	For Rb, no mutations that introduce STOP, frameshift, splice variants or missense mutations	Concomitant lack of mutations in Rb (proficient Rb (with no loss of Rb function either by mutation or deletion) and loss of p16
	C3	CDK4 amplification/Rb WT	Amplification > 3 copies	Proficient Rb (with no loss of Rb function either by mutation or deletion)<br>Focal > 3, otherwise >4
	C4	CCND1 amplification/Rb WT	Amplification > 3 copies	Proficient Rb (with no loss of Rb function either by mutation or deletion)<br>Focal > 3, otherwise >4
	C5	STK11/LKB1 mutation or homozygous deletion, or TSC1/2 mutation, with activated KRAS/MAPK pathway/Rb WT	All STK11/LKB1 mutations or homozygous deletions, or TSC1/2 mutations, with concomitant KRAS mutation (codons 12, 13, 61, 68), NRAS mutation or NF1 mutation	Proficient Rb (with no loss of Rb function either by mutation or deletion)

Drug	Arm	Molecular Cohort	Specific Aberration	Rule
	C6	KRAS mutation/Rb WT	All KRAS mutations - codons 12, 13, 61, 68.<br><br>No Tier 1 or Tier 2 concomitant genetic change (Some Tier 3 aberrations may also exclude patients; these will be queried by the Trial Office with CRUK during the Screening Registration Process):<br>STK11/LKB1mutation or homozygous deletion, PIK3CA mutation or amplification, PTEN mutation or homozygous deletion, AKT mutation, EGFR mutation, HER2 mutation, TSC1/2 mutation, or FGFR2/3 mutation.	Proficient Rb (with no loss of Rb function either by mutation or deletion).<br>All single KRAS mutations eligible.

24.2 Specific Eligibility Criteria

24.2.1 Inclusion Criteria

For inclusion in the study, patients must fulfil all of the following criteria:

- Patients must fulfil all the core eligibility criteria.
- Technology hub result (or locally obtained result from an approved Laboratory if applicable.
- Eastern Cooperative Oncology Group (ECOG) Performance Status <u><</u>2 with no deterioration over the previous 2 weeks (see Appendix 8: Eastern Cooperative Oncology Group Performance Status Criteria).
- Ability to swallow oral medication.
- Adequate hepatic function in patients **with** liver metastases: AST and/or ALT ≤ 5.0 x ULN

24.2.2 Exclusion Criteria

Patients must not enter the trial if any of the following exclusion criteria are fulfilled:

- Patients who do not fulfil all the core eligibility criteria.
- Treatment with any of the following:
    - Concomitant use of medicines known to prolong QT interval within 6 half-lives plus 1 day prior to the first dose of palbociclib (see Section 24.2.3.1.1).
    - Food or drugs known to be CYP3A4 inhibitors or inducers within 7 days prior to the first dose of palbociclib (see Section 24.2.3.1.2).
    - Radical or palliative radiotherapy within 4 weeks prior to the first dose of palbociclib.

    - Any other chemotherapy, investigational agents or other anti-cancer therapy (including targeted therapy, biological response modifiers, immunotherapy, or endocrine therapy (with the exception of exemestane)) within 4 weeks prior to the first dose of palbociclib.
- Any of the following cardiac related events within 6 months of registration:
    - severe or unstable angina pectoris, ongoing cardiac dysrhythmias of CTCAE version 4.0 Grade 2 or higher, atrial fibrillation of any grade, coronary/peripheral bypass graft, symptomatic congestive heart failure, cerebrovascular accident including transient ischemic attack, or symptomatic pulmonary embolism.
- Current malabsorption syndrome significantly affecting gastrointestinal function, resection of the stomach or small bowel, or active inflammatory bowel disease or chronic diarrhoea.
- History of non-infectious pneumonitis requiring steroids or has active pneumonitis or significantly reduced transfer coefficient (KCO).
- **C6 – KRAS cohort only:** No result available, or confirmation of any of the following concomitant genetic changes (Tier 1 or Tier 2, and some Tier 3 changes [see Table 28]):
    - PIK3CA mutation or amplification.
    - STK11/LKB1 mutation or homozygous deletion.
    - PTEN mutation or homozygous deletion.
    - AKT mutation.
    - EGFR mutation.
    - HER2 mutation.
    - FGFR2/3 mutation.
    - TSC1/2 mutation.

24.2.3 Restrictions & Concomitant Medications

Information on any treatment from the date of informed consent until 28 days after the administration of the last treatment dose should be recorded. If medically feasible, patients taking regular medication should be maintained on it throughout the study period. Patient should be advised to inform their treating physicians of all concomitant medications, including prescription medicines, over-the-counter drugs, vitamins, and herbal products.

Nb. These lists are not exhaustive and the absence of a drug from the lists does not imply that its combination with palbociclib is safe.

24.2.3.1 Prohibited Medications

The following treatments are prohibited throughout the duration of the active treatment phase:

24.2.3.1.1 QT interval prolongation

Concomitant medications **known to prolong the QT interval**, or **with any risk of prolonging the QT interval** are prohibited during the active treatment phase. Any patients taking such drugs at or prior to registration should discontinue the drug 6 half-lives plus 1 day prior to commencing trial treatment.

For a list of drugs known to prolong the QT interval, or with any risk of prolonging the QT interval, please refer to the following database: https://www.crediblemeds.org. Appendix 9 Credible Meds List of Drugs that Prolong QT Interval contains a list exported from this database on 2nd March 2018. **It is important to note that this list is a guide – the database will change with time and therefore needs to be checked in real-time when screening and registering a patient, and throughout their treatment. The Trials Office will email all sites when updates are made to the database.**

Please note that for Pfizer trial Arms, all concomitant medications listed on https://www.crediblemeds.org under the following 3 categories will exclude patients from entering the trial as per the eligibility criteria, unless an exception applies*:

- **'Known risk of TdP'**
- **'Possible risk of TdP'**
- **'Drugs to Avoid in Congenital Long QT'**

*The following exceptions may apply:

- The category '**Drugs to Avoid in Congenital Long QT**' includes drugs that should be avoided in patients with diagnosed or suspected congenital long QT syndrome. Patients without any evidence of these conditions may continue taking drugs allocated to this category alone and would be therefore eligible for inclusion in the trial.
  Important note: '**Drugs to Avoid in Congenital Long QT'** category may also be assigned to drugs in parallel with either of the following 2 categories '**Known risk of TdP**' and '**Possible risk of TdP'**; these concomitant medications are still prohibited for all trial patients and therefore patients would be ineligible for inclusion in the trial.
- Patients receiving treatment with a concomitant medication within the risk category **'Conditional Risk of TdP'** are **not** excluded from trial entry into Arm D, regardless of whether they are also assigned the **'Drugs to Avoid in Congenital Long QT'** category in addition. (Medications within this category are not listed within Appendix 9 Credible Meds List of Drugs that Prolong QT Interval but will still appear on https://www.crediblemeds.org).

It is the responsibility of an Investigator (Consultant level) to review and clinically evaluate all concomitant medications. Please contact the National Lung Matrix Trial Office for clarification regarding any drugs that appear on the database that are not listed in Appendix 9 Credible Meds List of Drugs that Prolong QT Interval.

The drugs listed on this website are taken from information provided by The Arizona Center for Education and Research on Therapeutics and The Critical Path Institute, Tucson, Arizona and Rockville, Maryland. Important Note - If a patient is being treated with such medication or is taking another medication that may affect QT interval which is not on the database, please contact the National Lung Matrix Trial Office to obtain the recommended withdrawal/ minimum period prior to starting trial treatment.

24.2.3.1.2 CYP3A

In vitro data indicate that CYP3A and sulfotransferase (SULT) enzyme SULT2A1 are mainly involved in the metabolism of palbociclib. Palbociclib is a weak time-dependent inhibitor of CYP3A following daily 125 mg dosing to steady state in humans. In vitro, palbociclib is not an inhibitor of CYP1A2, 2A6, 2B6, 2C8, 2C9, 2C19, and 2D6, and is not an inducer of CYP1A2, 2B6, 2C8, and 3A4 at clinically relevant concentrations.

- CYP3A Inhibitors: Data from a drug-drug interaction (DDI) study in healthy subjects indicate that coadministration of multiple 200 mg doses of itraconazole with a single 125 mg palbociclib dose increased palbociclib total exposure (area under the curve, AUC_{inf}) and the peak exposure (C_{max}) by approximately 87% and 34%, respectively, relative to a single 125 mg palbociclib dose given alone. The concomitant use of CYP3A inhibitors including, but not limited to: amprenavir, atazanavir, boceprevir, clarithromycin, conivaptan, delavirdine, diltiazem, erythromycin, fosamprenavir, indinavir, itraconazole, ketoconazole, lopinavir, mibefradil, miconazole, nefazodone, nelfinavir, posaconazole, ritonavir, saquinavir, telaprevir, telithromycin, verapamil, voriconazole, and grapefruit, grapefruit juice or any product containing grapefruit, should be avoided during treatment and within 7 days prior to the first dose of palbociclib.

- CYP3A Inducers: Data from a DDI study in healthy subjects indicate that coadministration of multiple 600 mg doses of rifampin, a strong CYP3A inducer, with a single 125 mg palbociclib dose decreased palbociclib AUC_{inf} and C_{max} by 85% and 70%, respectively, relative to a single 125 mg palbociclib dose given alone. The concomitant use of CYP3A inducers including, but not limited to: carbamazepine, enzalutamide, felbamate, nevirapine, phenobarbital, phenytoin, primidone, rifabutin, rifampin, rifapentin, and St. John's wort, should be avoided during treatment and within 7 days prior to the first dose of palbociclib. Moderate CYP3A inducers may be permitted (see Section 24.2.3.2).

24.2.3.2 Medications Not Recommended

The following treatments are not recommended throughout the duration of the active treatment phase. Alternative therapies should be considered whenever possible. If usage of the following treatments is deemed necessary, consultation and agreement with the National Lung Matrix Trial Office is required prior to treatment initiation.

- Chronic immunosuppressive therapies should be avoided, including systemic corticosteroids. Steroids given for physiological replacement, as anti-emetics or inhaled as well as short course of oral/topical steroids given for allergic reactions or asthma flares are allowed.
- Moderate CYP3A Inducers: Data from a DDI study in healthy subjects (N = 14) indicate that coadministration of multiple 400 mg daily doses of modafinil, a moderate CYP3A inducer, with a single 125 mg palbociclib dose decreased palbociclib AUC_{inf} and C_{max} by 32% and 11%, respectively, relative to a single 125 mg palbociclib dose given alone. The concurrent use of moderate CYP3A inducers (e.g. bosentan, efavirenz, etravirine, modafinil and nafcillin) can be used concurrently with palbociclib when it cannot be avoided. No dosing adjustments are required.

24.2.3.3 Concurrent Radiotherapy

Concurrent radiotherapy is not permitted. If palliative radiotherapy is indicated, the patient should stop taking palbociclib the day before radiation commences and should not recommence trial treatment for a minimum of 7 days. This should be decided on consultation with the National Lung Matrix Trial Office.

24.2.3.4 Permitted Medications

The following treatments are permitted throughout the duration of the active treatment phase:

- Standard therapies for pre-existing medical conditions, medical and/or surgical complications, and palliation. Any medication intended solely for supportive care (e.g., analgesics, antidiarrhoeals, antidepressants) may also be used at the Investigator's discretion.
- Bisphosphonates and receptor activator of nuclear factor kappa-B ligand (RANKL) inhibitors for the treatment of osteoporosis or management of existing bone metastases may be continued for patients who have been receiving them at a stable dose for at least 2 weeks prior to registration. However the need to initiate or increase the dose of these therapies during the study will be considered as indicative of disease progression leading to the discontinuation of patient from the active treatment phase unless disease progression can be completely ruled out and the exact reason for the use of these therapies clearly documented in the patient's source documentation.
- Haematopoietic growth factors (e.g., G-CSF, GM-CSF): Primary prophylactic use of granulocyte-colony stimulating factors is not permitted but they may be used to treat treatment-emergent neutropenia as indicated by the current American Society of

Clinical Oncology (ASCO) guideline. If neutropenic complications are observed in a cycle in which primary prophylaxis with CSFs was not received, secondary prophylaxis may be given at the discretion of the Investigator, but only if dose reduction or delay are not considered being a reasonable alternative.
- Erythropoietin may be used at the Investigator's discretion for the supportive treatment of anaemia.

24.2.3.5 Food Restrictions

Grapefruit, grapefruit juice or any product containing grapefruit, should be avoided during treatment and for at least 7 days prior to the first dose of palbociclib.

24.2.3.6 Other Restrictions

The use of herbal medicine is not recommended during the active treatment phase. St John's Wort must be discontinued at least 7 days prior to the first dose of palbociclib (see Section 24.2.3.1.2).

24.2.3.7 Contraception

There were no effects on oestrous cycle (female rats) or mating and fertility in female rats in nonclinical studies. However, no clinical data have been obtained on fertility in human females.

Palbociclib is considered to have the potential to impair reproductive function and fertility in male humans based on nonclinical findings in rats and dogs. In a male rat fertility study, there were no effects on mating or fertility, but palbociclib-related findings in the testis and epididymis were consistent with repeat-dose toxicity findings, and correlated with lower sperm motility and density. Men should consider sperm preservation prior to beginning therapy with palbociclib.

There are no adequate and well-controlled studies using palbociclib in pregnant women. Based on findings in animals and mechanism of action, palbociclib can cause foetal harm when administered to a pregnant woman. In animal studies, palbociclib was fetotoxic at maternally-toxic doses.

Advise women of childbearing potential to avoid becoming pregnant while receiving palbociclib. Females of childbearing potential who are receiving this drug should use adequate contraceptive methods during therapy and for at least 21 days after completing therapy.

Male patients must be surgically sterile or must agree to use effective contraception during the on-treatment period of the study and for at least 90 days after completion of treatment.

A definition of females of childbearing potential and females of non-childbearing potential and acceptable methods of contraception are described in Section 6.3.

24.3 Trial Treatment

24.3.1 Investigational Medicinal Product

Palbociclib has been tested in a Phase 1 dose escalation Study (A5481001) in 74 patients with advanced cancer. Two dosing schedules were evaluated: Schedule 3/1 (3 weeks on treatment/1 week off treatment) and Schedule 2/1 (2 weeks on treatment/1 week off treatment).

All DLT observed in this study were related to myelosuppression and mainly consisted of Grade 3 neutropenia lasting more than 7 days after the end of the treatment cycle. However, neutropenia was reversible and non-cumulative. The most common non-haematological

adverse events included fatigue, diarrhoea, constipation, vomiting and dyspnoea, all with mild to moderate severity. A greater proportion of patients on the 2/1 schedule had treatment-related TEAEs during and after Cycle 1 than patients on the 3/1 schedule although the proportion of patients with treatment-related neutropenia was similar with respect to the 2 dosing schedules, both during and after Cycle 1. One partial response was reported in a patient with testicular cancer. A total of 13/37 patients treated with Schedule 3/1 evaluable for efficacy experienced stable disease (SD), including 6 patients with SD lasting 40 weeks or longer. One of these patients was a woman with ER+ breast cancer who had previously received 7 lines of treatment for her disease. This patient remained on treatment for 80 weeks (7 cycles at 50 mg/d and 13 cycles at 75 mg/d) and eventually discontinued treatment due to disease progression. Based on the relatively improved safety profile of Schedule 3/1, and the efficacy results from this study, the Schedule 3/1 was selected for further clinical development and the RP2D for this schedule was determined to be 125 mg/d. Therefore Palbociclib will be administered at a dose of 125mg PO daily on Day 1 to Day 21 following a 1 week of rest period, given as 4 weeks cycles.

Palbociclib, an orally active pyridopyrimidine, is a potent and highly selective reversible inhibitor of cyclin-dependent kinase (CDK) 4 and CDK6. The compound prevents cellular DNA synthesis by prohibiting progression of the cell cycle from G1 into the S phase, as demonstrated in laboratory models and early clinical trials. Palbociclib preclinical data indicate that it may be expected to have direct effect on growth arrest as well as potential secondary cytoreductive activity. Treatment of cultured tumour cells with palbociclib causes growth arrest that is accompanied by the inhibition of specific retinoblastoma (Rb) phosphorylation by CDK4 or CDK6 on residues serine -780 and -795 of Rb. Consequently, the phosphorylation status of these sites serves as specific biomarkers of CDK4/6 inhibition by palbociclib.

Indications currently under investigation include breast cancer in combination with endocrine therapy (e.g. aromatase inhibitors, fulvestrant, tamoxifen), as well as recurrent/metastatic squamous cell carcinoma of the head neck in combination with cetuximab, and metastatic pancreatic ducta adenocarcinoma in combination with nab-paclitaxel.

Palbociclib will be provided free of charge by Pfizer.

Patients should be instructed to swallow palbociclib tablets whole and not to chew them prior to swallowing. No tablet should be ingested if it is broken, halved, cracked, crushedor otherwise not intact. The tablet should also not be dissolved for any reason. Patients should be encouraged to take their dose at approximately the same time each day. Patients should be instructed to record daily administration of the study drugs in a patient diary.

Patients may take palbociclib with or without food. Palbociclib will be administered 125 mg orally once a day for 21 days of every 28 day cycle followed by 7 days off treatment. Patients experiencing investigational product related toxicity may have their dose modified (see dose modification Section 24.3.4).

Patients who miss a day's dose entirely must be instructed NOT to "make it up" the next day. Patients who vomit any time after taking a dose must be instructed NOT to "make it up," and to resume treatment the next day as prescribed. Patients who inadvertently take 1 extra dose during a day must be instructed to skip the next day's dose. Both the Arm C Patient Information Sheet and Arm C Patient Diary contain more specific instructions for patients to follow regarding how to take their medication.

Please note patients who meet RECIST criteria for progressive disease (PD) may be continued on trial treatment if the treatment is tolerable and the Investigator believes it to be of clinical benefit; see Section 9.3.

Please also refer to the Pharmacy Manual for further details.

24.3.2 Schedule of Assessments

Table 29: Palbociclib - Schedule of Assessments

	**Screening**	**Treatment 21 days on, 7 days off – 125mg once daily (28 day cycles)**			**Discontinuation** (+ 7 days)**	**28 day follow up visit[r]** (+ 7 days)**	**Post-28 day follow up** (± 7 days)***
	Within 28 days of trial treatment (unless specified)	Cycles 1 & 2		Cycle 3 onwards (± 2 days)*			
		Day 1 (± 2 days cycle 2 only)*	Day 15 (± 2 days)*	Day 1 (± 2 days)*			
Informed consent[a]	X						
Demography & baseline characteristics[b]	X						
Medical history[c]	X						
Inclusion / exclusion criteria[d]	X						
Physical examination[e]	X	X		X	X		
ECOG performance status	X (within 14 days of treatment)	X	X	X	X		
Vital signs (inc. weight)[f]	X	X		X	X		
ECG[g]	X	X	X	X	X		
Haematology, Clinical chemistry, Urinalysis[h]	X (within 7 days of trial treatment)	X (- 2 days cycle 2 only)	X (- 2 days)	X (- 2 days)	X		
Glycosylated Haemoglobin (HbA1c)[i]	X			X (every 3rd cycle)		X	

	Screening	Treatment 21 days on, 7 days off – 125mg once daily (28 day cycles)			Discontinuation (+ 7 days)**	28 day follow up visit[r] (+ 7 days)**	Post-28 day follow up (± 7 days)***
	Within 28 days of trial treatment (unless specified)	Cycles 1 & 2		Cycle 3 onwards (± 2 days)*			
		Day 1 (± 2 days cycle 2 only)*	Day 15 (± 2 days)*	Day 1 (± 2 days)*			
Pregnancy test[j]	X	X (cycle 1 only)			X		
Tumour assessments[k]	X	Every 6 weeks during year 1 (± 7 days) [except 1st scan + 7days only]					X ◊
Adverse events & Concomitant Medications	X	X	X	X	X	X	
Dispense study drug		X		X			
Administer study drug[l]		OD dosing 21 days on, 7 days off					
Smoking status[m]		X (cycle 1 only)		X (every other cycle)	X		
Germline DNA sample[n]		X (cycle 1 only) (- 2 days)					
ctDNA samples[o]		X (cycle 1 only) (- 2 days)		X (every 8 weeks beginning cycle 3) (- 2 days)	X		X $
Optional research biopsy[p]		X (post-reg, pre-tx)			X		
Survival status[q]							X

- * Visit may occur ± 2 days of the planned visit date. Individual assessments may occur independently of the visit date where indicated in the table above. Where applicable and acceptable in accordance to local practices, visits may be performed by telephone or video call.
- ** Visit may occur + 7 days of the planned visit date
- *** Visit may occur ± 7 days of the planned visit date

a Prior to the start of any study specific procedures, each patient must provide signed informed consent.

b Demography must be captured for all patients. Demographic data and other characteristics will include: date of birth, gender, race/ethnicity.

c A standard medical and surgical history will be obtained, including prior cancer treatment.

d Patients must not be registered unless all eligibility criteria have been fully met.

e Physical examination includes general appearance, respiratory, cardiovascular, skin, head and neck (including ears, eyes, nose and throat), lymph nodes, thyroid, abdomen, musculo-skeletal (including spine and extremities) and neurological systems and should be performed at screening, pre-dose on Day 1 of every cycle and at discontinuation.

f Vitals signs are to be recorded are height (at screening only), weight, BP and pulse; required at screening, pre-dose on day 1 of every Cycle and at discontinuation.

g 12-lead ECG required at screening, pre-dose on day 1 of every cycle, day 15 of cycles 1 & 2 and at discontinuation.

Twelve-lead ECGs will be obtained after the patient has been resting semi-supine for at least 10 minutes prior to times indicated. All ECGs should be recorded with the patient in the same physical position. For each time point unless specifically stated above, three consecutive ECG recordings should be performed approximately 2 minutes apart to determine the mean QTcF interval. A standardised ECG machine should be used and the patient should be examined using the same machine throughout the study if possible. After paper ECGs have been recorded, the Investigator or designated physician will review each of the ECGs and may refer to a local cardiologist if appropriate. A paper copy should be filed in the patient's medical records. If an abnormal ECG finding at screening or cycle 1 day 1 is considered to be clinically significant by the Investigator, it should be reported as a concurrent condition. For all ECGs details of rhythm, intervals (R-R, PR, QT and QRS) and an overall evaluation will be recorded.

h Samples to be collected at screening, day 1 and day 15 cycles 1 and 2, day 1 of subsequent cycles and discontinuation. Samples can be taken up to 2 days earlier than the actual visit date (where indicated). Clinical Chemistry, Haematology to be performed at screening, pre-dose on day 1 of every cycle, day 15 in cycles 1 & 2 and at discontinuation. Where applicable and acceptable in accordance to local practices, blood tests can be performed locally in GP surgeries or in community based clinics.

Clinical Chemistry: Albumin, AST, ALT, ALP, bilirubin (total), calcium (total), creatinine, magnesium, sodium, urea nitrogen and potassium.

Haematology: FBC

Urinalysis: Protein, glucose, blood. If urinalysis abnormal, perform microscopy – red blood cells, white blood cells, bacteria, casts, crystals.

i Glycosylated haemoglobin (HbA1c) to be collected at screening, day 1 of every third cycle and at the 28 day follow up visit.

j Investigator should assess the patient's compliance to contraceptive measures and perform a test if required. Female patients of child-bearing potential only. A serum or urine pregnancy test is to be performed at screening, pre dose on cycle 1 day 1 and at discontinuation. In the event of suspected pregnancy during the study, the test should be repeated and, if positive, the patient discontinued from study treatment immediately.

k CT or MRI scan of head, chest and abdomen to be performed at screening. CT or MRI scans of chest and abdomen to be performed until discontinuation. Following screening, the first tumour assessment should be performed 6 weeks after cycle 1 day 1, then every 6 weeks thereafter for the first year, later reducing to every 12 weeks. Scans should be performed ± 7 days (except 1st scan + 7 days only). If brain metastases are identified at Screening or if clinically indicated, head scanning should also be performed throughout treatment at the same time points. **The same imaging modality must be used consistent throughout the course of the trial for each patient.**

◊ Tumour assessments will be performed in follow up for patients who discontinue treatment for reasons other than Progressive Disease (e.g. toxicity). These scans should continue to be performed on a 6-weekly basis for the first year relative to the start date of treatment, then every 12 weeks until disease progression or the patient starts a new anti-cancer therapy (unless the patient withdraws consent to do so). Scans should be of the chest and abdomen, and only include the head where brain metastases are identified at screening, or if clinically indicated. All scans to be reported using RECIST 1.1.

l Cycle 1 Day 1: Treatment must commence within 7 days of trial registration.

m Smoking status data will be collected through questions and CO monitoring at **pre-dose** cycle 1 day 1, then every 8 weeks (day 1 of every other cycle) and at discontinuation.

n A whole blood germline DNA sample is to be collected **pre-dose** on Cycle 1 day 1. The sample can be taken up to 2 days earlier than the actual visit date (commencement of cycle). If the sample is not collected at this timepoint, it should be collected at the next visit. Refer to the Laboratory Manual for sample processing guidelines.

o ctDNA samples to be collected at **pre-dose** cycle 1 day 1, then every 8 weeks (day 1 of every other cycle) beginning cycle 3 and at discontinuation. Samples can be taken up to 2 days earlier than the actual visit date (where indicated). After 12 months of treatment, ctDNA sample collection may be reduced to 12 weekly in line with adjusted visit timing (see footnote 's'). Refer to the Laboratory Manual for sample processing instructions.

$ ctDNA samples will be collected in follow up for patients who discontinue treatment for reasons other than Progressive Disease (e.g. toxicity). These samples should be performed at the same visit as follow up CT or MRI scans until disease progression or the patient starts a new anti-cancer therapy (unless the patient withdraws consent to do so). Samples should be collected on a 6-weekly basis for the first year relative to the start date of treatment then every 12 weeks.

p An optional fresh metastatic/recurrent tumour biopsy sample should be collected (if patient consents) post–registration (pre-treatment) and at the end of treatment visit for patients who discontinue treatment for reasons other than disease progression (origin from either the primary tumour of site of metastasis). An optional pre-treatment biopsy should not be performed in cases where the patient has already had a mandatory biopsy for

molecular testing (Note - a mandatory repeat SMP2 biopsy will be performed if the patient has had targeted therapy e.g. ALK inhibitor). The discontinuation biopsy must be performed prior to commencing further anti-cancer therapy. A post-treatment biopsy will only be requested from patients with an objective response or stabilisation of disease (PR or CR), or 6 months on treatment with evidence of stabilisation (SD) for patients who have previously progressed. The tumour tissue will be used to determine possible mechanisms of resistance to study treatment. Refer to the Laboratory Manual for sample processing instructions.

q Survival status will be collected every 12 weeks (± 7 days) post-permanent discontinuation of palbociclib until death.

r 28 day follow up visit should be carried out 28 days (+ 7 days) post-permanent discontinuation of palbociclib.

s Once a patient has completed 12 months of treatment (approximately 13 cycles), visits may be reduced to 12 weekly at the discretion of the Investigator.

24.3.3 Toxicity Profile

For a comprehensive overview of the safety profile please refer to the most current IB.

24.3.3.1 Expected Adverse Events

Clinical studies to date suggest that toxicity associated with palbociclib is largely limited to uncomplicated neutropenia, fatigue, diarrhoea and anaemia, and nausea. The observed neutrophil nadir of 3 weeks will be managed by a 3 weeks on/1 week off schedule.

Data from pre-clinical studies has indicated that palbociclib has the potential to delay cardiac repolarization as measured by prolongation of the QT interval on ECG. *In vitro* (hERG) and *in vivo* (dog telemetry) studies revealed a potential for QT prolongation at unbound concentrations ≥14-fold the unbound steady-state Cmax associated with the clinical dose of 125 mg QD (refer to the palbociclib IB for additional details).

A preliminary PK/PD analysis has been conducted to explore the QT/QTc and plasma PD-032991 concentration relationship for Study A5481001 (FIH study) by using graphical methods and mixed effects linear modelling (NONMEM). Data from 73 patients were used for the analysis, and an analysis of the QTcF and QTcB data demonstrated that QTcF was the more appropriate correction method based on plots of the QTc versus RR interval. No patient had a maximum on treatment QTcF value of ≥ 500 msec. The QTcF changes from the baseline at the mean Cmax calculated for 200 mg dose were simulated for 10000 patients. The mean and upper 95% confidence interval of QTcF change from the baseline were 5.8 and 9.4 msec, respectively.

24.3.3.2 Special Adverse Events of Note

24.3.3.2.1 Effects on Bone Marrow:

Based on the nonclinical studies performed to date, bone marrow is the main target organ for toxicity. Consistent with nonclinical observations, patients enrolled to the clinical studies of palbociclib have experienced treatment-related changes in their blood counts such as neutropenia, leukopenia, anaemia, and thrombocytopenia. In general, myelosuppression was reversible and manageable.

During clinical studies with palbociclib, patients' complete blood counts should be closely monitored. In case of clinically significant toxicities, treatment with palbociclib should be interrupted and the dose of palbociclib modified as defined in the protocol. Myelosuppression and associated events should be managed based on the judgment of the investigator and according to best clinical practice. Erythropoietin may be used for the supportive treatment of anaemia. Granulocyte-colony stimulating factors should not be used prophylactically but they may be used for treatment-emergent neutropenia as per current American Society of Clinical Oncology (ASCO) guidelines.

24.3.3.2.2 Effects on the Cardiovascular System:

Data from in vitro and in vivo nonclinical studies indicated that palbociclib has the potential to delay cardiac repolarization as measured by prolongation of the QT interval on the electrocardiogram (ECG).

The effect of palbociclib on the QT interval corrected for heart rate (QTc) was evaluated using time-matched electrocardiograms (ECGs) evaluating the change from baseline and corresponding pharmacokinetic data in 77 patients with breast cancer. Palbociclib did not

prolong QTc to any clinically relevant extent at the recommended dose of 125 mg daily (Schedule 3/1) (Section 6.1.5.2 of the IB).

Based on the data from open-label studies no patient has experienced a QTcF interval >500 ms across the program. No significant changes in blood pressure, pulse rate and body weight have been observed in the 2 completed Phase 1 clinical studies in advanced cancers (A5481001 and A5481002; see Section 6.2.2.1 of the IB). In Study A5481001, an analysis of the corrected QT interval using Fridericia's formula (QTcF) and corrected QT interval using Bazett's formula (QTcB) data from 73 patients in Study A5481001 demonstrated that QTcF was the more appropriate correction method based on plots of the QTc versus RR interval. Using QTcF, 46 of 73 patients had a maximum increase from baseline of <30 ms and no patient had a maximum on treatment value of >500 ms. Notably, 1 female patient who had received palbociclib 75 mg once daily (QD) on Schedule 3/1, had a maximum QTcF increase of 67 ms from baseline to Cycle 1. Additionally, QTcF increases ranging from 39 to 51 ms compared to baseline persisted throughout her ECG collection period of 5 subsequent cycles. After 7 cycles, the dose of palbociclib was increased to 100 mg QD. The patient remained on treatment for a total of 39 cycles with no cardiac-related adverse events.

QT data analysis for Study A5481002 indicated no clinically significant mean changes in ECGs. Using QTcF in Study A5481002, all 17 patients in the analysis had a maximum increase from baseline of <30 ms and a maximum post-baseline value for QTc of <500 ms, no subject had a maximum on-study QTcF greater than Grade 1.

In the studies of breast cancer (A5481003) and multiple myeloma (A5481004), no significant changes in blood pressure, pulse rate and body weight have been observed (see Section 6.2.2.2 and Section 6.2.2.3 of the Investigator's Brochure). No patients experienced QTcF intervals >500 ms in either study. However, 3 patients in A5481004 and 1 patient in A5481003 treated with palbociclib had a maximum change from baseline in QTcF of >60 ms.

In Study A5481023 in women with breast cancer treated with palbociclib or placebo in combination with fulvestrant, no notable differences were seen between baseline and end-of treatment in the mean and median values for RR interval, heart rate, QT interval, QTcB interval, and QTcF interval, in either treatment arm. There were also no relevant differences between treatment arms for any of the ECG parameters. The mean and median changes from baseline to any post-baseline time point were generally minor for all parameters and did not show any relevant differences between treatment arms. None of the patients in either treatment arm showed post-baseline ECG findings of a maximum QT interval ≥500 ms. The percentage of patients with post baseline QTcB interval ≥500 ms was slightly higher in the palbociclib plus fulvestrant arm than in the placebo plus fulvestrant arm (5.5% vs 1.4%). Post-baseline QTcF intervals ≥500 ms were not observed in any patient in the palbociclib plus fulvestrant arm, and were observed in 1 (1.4%) patient in the placebo plus fulvestrant arm.

Appropriate monitoring of patients enrolled in clinical studies should include clinical examinations, vital signs measurements, routine ECGs, and AE monitoring. In case of QTc prolongation, concomitant conditions such as electrolyte unbalances or use of medications affecting the QT interval should be ruled out or corrected. In case of clinically significant toxicities, palbociclib administration should be interrupted and the dose reduced as indicated in Section 24.3.4.

24.3.3.2.3 Effects on the Respiratory Tract:

Respiratory effects seen in animals are not considered to represent a significant safety concern for clinical studies given the short-term and limited effects in rats and likely anaesthetic complications contributing to respiratory findings observed in dogs. Although Dyspnoea, Upper respiratory tract infection and Cough were very commonly reported events in clinical studies,

most such events were mild and self-limited. Serious adverse respiratory events reported to date include cases of dyspnoea, upper respiratory tract infection, and pneumonia.

Although no cases of pneumonitis or interstitial lung disease have been reported in Studies A5481003 or A5481023, FDA reviewed cases of interstitial lung disease (ILD) and pneumonitis with cyclin-dependent kinase 4/6 (CDK 4/6) inhibitors were identified in the manufacturers' completed and ongoing clinical trials and their postmarket safety databases. Although rare, there were serious cases and/or deaths with palbociclib, abermaciclib and ribociclib. Across clinical trials of the three CDK 4/6 inhibitors, 1-3% of cases of any grade were reported and less than 1% had fatal outcomes. At the time of writing, there were no fatalaties reported in patients taking palbociclib. Among patients who developed ILD/pneumonitis, including fatal cases, there were patients who had no risk factors for lung disease but some patients had at least one risk factor.

Patients should be monitored regularly for pulmonary signs or symptoms indicative of ILD/pneumonitis. Symptoms may include hypoxia, cough, dyspnoea, or interstitial infiltrates on radiological examinations in patients in whom infectious, neoplastic or other causes have been excluded. In patients who have new or worsening respiratory symptoms or are suspected to have developed pneumonitis, treatment should be interrupted immediately.
See section 24.3.4.1 for further details on the management and treatment of suspected ILD/pneumonitis.

24.3.3.2.4 Pulmonary Embolism:

Pulmonary embolism was reported in 15/872 (1.7%) of palbociclib treated patients in Study A5481003, A5481008, and A5481023, and in 5/471 (1.1%) of patients treated in the comparator arms of these studies. These incidences are consistent with epidemiological data on the frequency of pulmonary embolism in the advanced breast cancer population, and the higher incidence for pulmonary embolism in the palbociclib treated patients may be explained by the increased median treatment duration on the palbociclib arms of these studies.

Pulmonary embolism and other venous thromboembolic events will continue to be monitored but are not currently considered to be adverse drug reactions of palbociclib.

24.3.3.2.5 Effects on the Gastrointestinal Tract:

Non-adverse palbociclib-related gastrointestinal (GI) tract findings were noted in nonclinical toxicity studies. Treatment-related GI tract events have been commonly observed in patients who receive palbociclib as a single agent or in combination with other anticancer therapies. The most frequent gastrointestinal treatment-related events were nausea, vomiting, diarrhoea, and decreased appetite. Most occurrences were mild (Grade 1).

Supportive treatment measures may be initiated as deemed necessary by the treating physician in agreement with best clinical practices. In case of clinically significant toxicities, palbociclib administration should be interrupted and the dose reduced as indicated in Section 24.3.4.

24.3.3.2.6 Effects on Testes:

Palbociclib caused testicular degeneration in rats and dogs. The incidence and severity was dose related and correlated with decreases in testicular weight in the rat. Reversibility of the testicular degeneration was demonstrated following a 12-week non-dosing period in rats and dogs (also following a 4-week recovery period in rats). Patients should consider sperm preservation prior to beginning therapy with palbociclib.

CDK4 has shown expression in normal human spermatogonia (Bartkova et al, 2003). In addition, pathways involving CDKs appear to play a role in spermatogenesis (Ravnik and Wolgemuth, 1999). Testicular degeneration produced by palbociclib is consistent with CDK inhibition and alterations in cell cycle kinetics.

Male patients must be surgically sterile or must agree to use effective contraception during the on-treatment period of the study and for at least 90 days after completion of treatment.

24.3.3.3 Special Warnings and Precautions for Use

24.3.3.3.1 Nonclinical Findings

The primary palbociclib toxicities in preclinical studies are to the bone marrow, lymphoid tissues, and testes. These toxicities occurred in both rats and dog and are consistent with cell cycle inhibition produced by the intended pharmacology of the drug. Bone marrow pancytopenia resulted in decreases in various haematology parameters; however, the changes were reversible following cessation of dosing. Reversible myelosuppression is anticipated in clinical studies and may be dose-limiting. Palbociclib demonstrated a potential for aneugenicity in the in vitro and in vivo micronucleus assays. In addition, palbociclib was determined non-phototoxic.

Acute intravenous (IV) administration of palbociclib to anesthetized dogs resulted in significant pulmonary effects, including apnoea, which were reversible. Effects were transient, appeared related to peak plasma concentrations (>2040 ng/mL), and consistent with centrally-mediated respiratory depression. No changes in pulmonary function occurred at plasma drug concentrations <414 ng/mL. Pulmonary changes were observed in rats to which palbociclib was administered orally, which included rales, dyspnoea, and atrophy of tracheal epithelium. Results of the Purkinje fibre and human ether-a-go-go related (hERG) in vitro assays, and cardiovascular study in dogs have indicated a potential for prolongation of the QT interval.

Hyperglycaemia/glycosuria in relationship to pancreatic islet cell vacuolation was observed in rats only, with secondary findings of lens degeneration/cataracts, vacuolation of renal tubular epithelial cells, and adipose tissue following 15 and/or 27 weeks of intermittent dosing. These findings were not reversible following a 3-month recovery period (though partial reversal of adipose atrophy was observed). The dysregulation of glucose appears to be the result of diabetes secondary to the demise of pancreatic beta cells. Investigatory work in aged rats (with similar beta cell proliferation rates to humans) did not identify alterations in glucose metabolism or pancreatic microscopic findings at exposures where effects were previously identified in young rats, suggesting a susceptibility of young rats to the development of endocrine/metabolic effects. Consistent with the lack of glucose dysregulation, effects considered of secondary relationship including lens degeneration/cataracts were not observed in aged rats. Neither hyperglycaemia nor cataracts were observed in dogs following exposure to palbociclib for up to 9 months. The potential for palbociclib to cause pancreatic islet beta cell loss, subsequent dysregulation of glucose, and cataracts in humans is therefore considered low.

In the first in human study (A5481001) in which close monitoring of glucose was performed, no relationship of either dose level or duration of palbociclib treatment with elevated glucose levels was observed.

In Studies A5481003, A5481008, and A5481023, no measurements of glucose were obtained, however, there was no imbalance in the frequency of adverse event reports consistent with hyperglycaemia or diabetes between the palbociclib treated patients and the patients treated in the comparator arms. The measurement of glycosylated haemoglobin A1c which was

introduced per amendment into Studies A5481008 and A5481023 also did not indicate that palbociclib treated patients had a higher risk of developing hyperglycaemia.
Cataract was reported in 2.0% of 884 palbociclib treated metastatic breast cancer patients and in 0.2% of patients treated in the comparator arms [N = 471]. Further, Cataract nuclear was reported in 2 palbociclib treated patients (0.2%), with no reports of Cataract nuclear in the comparator arms. Ophthalmologic monitoring was introduced into Studies A5481008 and A5481023 per amendment and with few exceptions, no baseline ophthalmologic examinations were available. Many patients diagnosed with cataract were elderly and some had a history of diabetes. Cataract is not currently considered to be an adverse drug reaction of palbociclib.

Although there is currently no clinical evidence that patients treated with palbociclib are at increased risk of developing cataract, hyperglycaemia or diabetes, the potential for increased risk of hyperglycaemia and ophthalmological changes will be examined in the ongoing Asian Study A5481027.

24.3.3.3.2 Clinical Findings

Neutropenia:
In all clinical studies, neutropenia has been the most frequently reported adverse event. In Phase 1/2 study A5481003, neutropenia was reported in 73/95 (76.8%) patients receiving palbociclib and letrozole; in Study A5481004 (palbociclib in combination with dexamethasone and boretezomib patients with multiple myeloma) in 26/51 (51.0%) patients; in Study A5481008 (palbociclib in combination with letrozole) in 294/444 (66.2%) patients: in Study A5481023 (palbociclib in combination with fulvestrant) in 220/345 (63.8%) patients: and in Study A5481034 (palbociclib in combination with letrozole) in 87/236 (36.9%) patients. Across Studies A5481003, A5481008, and A5481023, the median time to first episode of any grade neutropenia was 15 days (range: 12-700 days) and the median duration of Grade ≥3 neutropenia was 7 days across 3 randomized clinical studies.

Febrile neutropenia has been reported in 5/51 (9.8%) palbociclib-treated patients in Study A5481004, in 1/17 (5.9%) palbociclib-treated patients treated in Study A5481002, in 8/444 (1.8%) palbociclib-treated patients in Study A5481008, in 3/345 (0.9%) patients in Study A5481023, and in 5/236 (2.1%) patients in Study A5481034.

Guidance for monitoring neutrophil counts and dose modification in response to neutropenia is detailed in Section 24.3.4.

Infections:
Since palbociclib has myelosuppressive properties, it may predispose to infections. Infections of any grade have been reported at a higher rate in patients treated with palbociclib plus letrozole or fulvestrant (54.8%) compared to patients treated in the respective comparator arms (36.9%). Grade 3 and 4 infections occurred in 4.4% and 0.7%, respectively, in patients treated with palbociclib in either combination compared to in 2.5% and 0%, respectively, of patients treated in the comparator arms. Monitor patients for signs and symptoms of infection and treat as medically appropriate. Physicians should inform subjects to promptly report any episodes of fever. In studies A5481003, A5481008 and A5481023 sepsis has been reported as a Serious Adverse Reaction in 0.5% of patients taking Palbociclib.

24.3.4 Dose Modifications and Toxicity Management

24.3.4.1 Interstitial Lung Disease (ILD)/Pneumonitis

In the case of documented or high suspicious treatment-related ILD or pneumonitis, immunosuppressive treatment should be started immediately. Ideally, an infection should be ruled out by bronchoscopy, especially in the case of grade 2 pneumonitis, in order to be able

to safely introduce trial treatment. If the infectious status cannot be reliably assessed, most algorithms advocate the administration of oral or i.v. broad spectrum antibiotics in parallel to the immunosuppressive treatment in grade 3 pneumonitis (see Table 30: ILD/Pneumonitis Management Grades 1-4 below).

In grade 1 to 2 pneumonitis, treatment consists of oral steroids with prednisone 1 mg/kg daily or equivalent. Patients should be clinically assessed every 2–3 days initially and, ideally, also radiologically in grade 2 pneumonitis. Steroids should be tapered over 4–6 weeks after recovery and reintroduction of palbociclib should be delayed until the daily dose of steroids equals 10 mg of oral prednisone per day or less (see Table 31).

In grade 3 to 4 moderate to severe cases, the patient should be hospitalised and treatment should consist of high-dose i.v corticosteroids [(methyl)prednisolone 2–4 mg/kg/day or equivalent], and trial treatment permanently discontinued. Where the patient's condition does not improve or there is no imaging improvement after 2 days, additional immunosuppressive strategies should be implemented. The addition of infliximab, mycophenolate mofetil (MMF) or cyclophosphamide are possible options. Tapering of steroids should be very slow and careful, over 6 weeks or more; relapses of pneumonitis during steroid tapering have been reported, adding considerations about recurrence in patients who rechallenge immunotherapy.

Table 30: ILD/Pneumonitis Management Grades 1-4

Grade & Symptoms	Palbociclib Action	Assessment & Investigations	Monitoring	Supportive Care
Grade 1:<br>Radiographic changes only<br>Ground glass change, non-specific interstitial pneumonia	Consider delay of treatment	Baseline indications:<br>Physical exam<br>Chest X-ray<br>Bloods [FBC/UEC/LFTs/TFTs/Ca/ESR/CRP/ ILD markers (KL-6, SP-D) and β-D-glucan [where available)]<br>Consider sputum sample & screening for viral, opportunistic or specific bacterial (Mycoplasma, Legionella) infections depending on the clinical context	Monitor symptoms every 2-3 days<br>If worsens, treat as Grade 2 or 3-4	
Grade 2:<br>Mild/moderate new symptoms<br>Dyspnoea, cough, chest pain	Withhold	Baselin indications as above plus: Repeat chest X-ray & bloods weekly<br>Lung function tests including TCLO<br>High-resolution CT +/- bronchoscopy & BAL pending appearances	Daily outpatient monitoring	Start antibiotics if suspicious of infection (fever, CRP, neutrophil counts)<br>If no evidence of infection or no improvement with antibiotics after 48h add in prednisolone 1 mg/kg/day orally<br>Consider pneumocystis prophylaxis depending on the clinical context<br>If no improvement after 48h oral prednisolone, manage as Grade 3

Grade & Symptoms	Palbociclib Action	Assessment & Investigations	Monitoring	Supportive Care
Grade 3 or 4:<br>Severe new symptoms<br>New/worsening hypoxia<br>Life threatening<br>Difficulty in breathing, acute respiratory stress syndrome (ARDS)	Permanently discontinue	Baseline tests as above (grade 2)<br>High-resolution CT & respiratory review +/- bronchoscopy & BAL pending appearances	Admit patient<br>Discuss escalation and ventilation	Cover with empiric antibiotics<br>*If no improvement or worsening after 48 hours:*<br>Add infliximab 5 mg/kg or MMF if concurrent hepatic toxicity<br>Continue with i.v. steroids – wean as clinically indicated

BAL: bronchoalveolar lavage, ESR: erythrocyte seimentation rate, MMF: mycophenolate mofetil, TCLO: transfer factor for carbon monoxide, TFT: thyroid function tests, UEC: urea, electrolytes, creatinine.

Table 31: ILD/Pneumonitis Management Following Improvement to Baseline

Grade	Supportive Care	Palbociclib Action
Grade 2	Wean oral steroids over at least 6 weeks, titrate to symptoms<br>Steroid considerations:<br>Calcium & Vitamin D supplementation as per local guidelines<br>Pneumonocystis prophylaxis – cotrimoxazole 480 mg bd Mon/Wed/Fri or inhaled pentamidine if cotrim allergy	Reintroduce once the daily dose of steroids equals 10 mg of oral prednisolone per day or less
Grade 3/4	Wean steroids over at least 8 weeks<br>Steroid considerations as above<br>Pneumonocystis prophylaxis as above	N/A – permanently discontinued

24.3.4.2 Treatment Delays and Discontinuation

Patients experiencing the following adverse events within a treatment cycle should have their treatment with palbociclib interrupted (delayed) until criteria for re-commencement of treatment are met:

- Uncomplicated grade 3 or 4 neutropenia (ANC<1000/mm^3);
- Grade 3 or 4 neutropenia (ANC<1000/mm^3) associated with a documented infection or fever ≥38.5°C;
- Grade 3 or grade 4 thrombocytopenia (platelet count <50,000/mm^3);
- Non-haematological toxicity persisting despite optimal medical treatment if either grade 2 and lasting more than 3 weeks or grade ≥3
- Grade 3 QTc prolongation (QTcF ≥501 msec on at least two separate ECGs).
- Grade 2 interstitial lung disease (ILD)/pneumonitis. Grade 1 events may also be considered for treatment interruption (see Section 24.3.4.1).

If a patient experiences concurrent >3xULN ALT and 2xULN bilirubin treatment should be withheld while the cause is investigated.

Appropriate follow up assessments should be done until adequate recovery occurs as assessed by the Investigator.

Doses may be held as needed until toxicity resolution. Depending on when the adverse event resolved, a treatment interruption may lead to the patient missing all subsequent planned doses within that same cycle or even to delay the initiation of the subsequent cycle.
If the adverse event that led to the treatment interruption recovers within the same cycle, then re dosing in that cycle is allowed. Doses omitted for toxicity are not replaced within the same cycle. The need for a dose reduction at the time of treatment resumption should be based on the criteria found below. If a dose reduction is applied in the same cycle, the patient will need to return to the clinic to receive new drug supply.

If a treatment delay results from a decline in haematological parameters, the frequency of blood count assessments should be adjusted as clinically indicated.

If the re-treatment parameters (see criteria below) are met within 3 weeks of treatment interruption, palbociclib may be resumed.

If these parameters have not been met within 3 weeks of treatment interruption (including the scheduled 1 week off treatment), permanent discontinuation of trial treatment should be considered in consultation with the National Lung Matrix Trial Office.

In the event of a treatment interruption for reasons other than treatment related toxicity (e.g., non-cancer related surgery) lasting >3 weeks, treatment resumption will be decided on consultation with the National Lung Matrix Trial Office. It is advised that treatment delays should not exceed 6 weeks.

Patients who become pregnant are not allowed to continue treatment.

24.3.4.3 Criteria for Recommencement of Treatment

The following parameters should be met prior to recommencement of treatment: 1) at the start of every new cycle and; 2) following a treatment interruption for treatment related toxicity:

- Platelet count ≥ 50,000/mm^3;
- ANC ≥ 1000/mm^3 and no fever;

- ≥grade 3 treatment-related non-haematological AEs considered related to palbociclib (including, nausea, vomiting, diarrhoea, and hypertension only if persisting despite optimal medical treatment) have recovered to ≤grade 1 or baseline.
- QTcF ≤480 msec and potential reversible causes (e.g. electrolyte imbalance, concomitant medications known to prolong QTcF) corrected. If QTcF remains above 480msec, a cardiologist should be consulted and ECG should be monitored more frequently until QTcF ≤480 msec.
- In the case of Grade 2 ILD/pneumonitis, trial treatment may be resumed once the AE has been reduced to baseline and the daily dose of steroids equals 10 mg of oral prednisolone per day or less (see Table 31).

24.3.4.4 Dose Reductions

Following dose interruption or cycle delay the Palbociclib dose may need to be reduced when treatment is resumed.

No specific dose adjustments are recommended for Grade 1/2 treatment-related toxicity. However, Investigators should always manage their patients according to their medical judgement based on the particular clinical circumstances.

Dose reduction of Palbociclib by 1 and, if needed, 2 dose levels (Table 32) will be allowed depending on the type and severity of toxicity encountered. Patients requiring more than 2 dose reductions will be discontinued from the study and entered into the follow-up phase.

Once a dose has been reduced for a given patient, all subsequent cycles should be administered at that dose level, unless further dose reduction is required. Dose re-escalation is not allowed.

Table 32: Palbociclib - Available Dose Levels

**Dose Level**	**Palbociclib Dose**
Starting dose	125 mg/d
-1	100 mg/d
-2	75 mg/d*
-3	Discontinue Study Treatment

* Palbociclib dose de-escalation below 75 mg/d is not allowed.

24.3.4.5 Evaluation, Management and Treatment of Toxicities

Palbociclib recommended dose modifications for treatment related toxicities requiring treatment interruption/delay or persisting despite optimal medical treatment are described in Table 33.

Table 33: Palbociclib - Dose Modifications

**Toxicity**	**Restart Palbociclib Treatment at:**
Uncomplicated Grade 3 neutropenia (ANC<1000/mm^3)	Same dose level

Toxicity	Restart Palbociclib Treatment at:
Grade 3 neutropenia (ANC<1000/mm^3) associated with a documented infection or fever ≥38.5°C	↓ 1 Dose Level
Grade 4 neutropenia (ANC<500/mm^3)	↓ 1 Dose Level
Grade 4 thrombocytopenia (Platelet count <25,000/mm^3)	↓ 1 Dose Level
Grade ≥3 non-hematologic toxicity (including, nausea, vomiting, diarrhoea, and hypertension only if persisting despite optimal medical treatment)	↓ 1 Dose Level

24.3.4.6 Missed Doses

Patients who miss a day's dose entirely should resume their usual schedule the following day. They should not be advised to take a higher dose the next day.

Patients who vomit any time after taking a dose should not repeat that dose. The patient should take the next dose at the scheduled time.

24.4 Reference List

- Cancer Genome Atlas Research Network (2012). Comprehensive genomic characterization of squamous cell lung cancers. *Nature* **489(7417):**519-25
- Calles A, Sholl LM, Rodig SJ *et al.* (2014). Immunohistochemical Loss of LKB1 Is a Biomarker for More Aggressive Biology in KRAS-Mutant Lung Adenocarcinoma. *Clin Cancer Res*, **21(12):** 2851–60.
- Chen Z, Cheng, K, Walton Z *et al.* A murine lung cancer co-clinical trial identifies genetic modifiers of therapeutic response. *Nature* **483:**613-617.
- Dickson MA, Tap WD, Keohan ML *et al.* (2013). Phase II trial of the CDK4 inhibitor PD0332991 in patients with advanced CDK4-amplified well-differentiated or dedifferentiated liposarcoma. *J Clin Oncol* **31(16):**2024-8.
- Finn RS, Dering J, Conklin D *et al.* (2009). PD 0332991, a selective cyclin D kinase 4/6 inhibitor, preferentially inhibits proliferation of luminal estrogen receptor-positive human breast cancer cell lines *in vitro*. *Breast Cancer Res* **11(5):**R77.
- Flaherty KT, Lorusso PM, Demichele A *et al.* (2012). Phase I, dose-escalation trial of the oral cyclin-dependent kinase 4/6 inhibitor PD 0332991, administered using a 21-day schedule in patients with advanced cancer. *Clin Cancer Res* **18(2):**568-76.
- Fry DW, Harvey PJ, Keller PR *et al.* (2004). Specific inhibition of cyclin-dependent kinase 4/6 by PD 0332991 and associated antitumour activity in human tumour xenografts. *Mol Cancer Ther* **3(11):**1427-38.
- Konecny GE, Winterhoff B, Kolarova T *et al.* (2011). Expression of p16 and retinoblastoma determines response to CDK4/6 inhibition in ovarian cancer. *Clin Cancer Res* **17(6):**1591-602.
- Leonard JP, LaCasce AS, Smith MR *et al.* (2012). Selective CDK4/6 inhibition with tumour responses by PD0332991 in patients with mantle cell lymphoma. *Blood* **119(20):**4597-607.
- Puyol M, Martín A, Dubus P (2010). A synthetic lethal interaction between K-Ras oncogenes and CDK4 unveils a therapeutic strategy for non-small cell lung carcinoma. *Cancer Cell* **18(1):**63-73.
- Santarius T, Shipley J, Brewer D *et al.* (2010). A census of amplified and overexpressed human cancer genes. *Nat Rev Cancer* **10(1):**59-64.
- Schwartz GK, LoRusso PM, Dickson MA (2011). Phase I study of PD 0332991, a cyclin-dependent kinase inhibitor, administered in 3-week cycles (Schedule 2/1). *Br J Cancer* **104(12):**1862-8.
- Wikman H, Nymark P, Väyrynen A *et al.* (2005). CDK4 is a probable target gene in a novel amplicon at 12q13.3-q14.1 in lung cancer.
- *Genes Chromosomes Cancer* **42(2):**193-9.
- Haanen JB, Carbonnel F, Robert C *et al* (2017). Management of toxicities from immunotherapy: ESO Clinical Practice Guidelines for diagnosis, treatment and follow-up. *Annals of Oncology* **28(4)**: i119-i142. https://www.esmo.org/Guidelines/Supportive-and-Palliative-Care/Management-of-Toxicities-from-Immunotherapy

25 ARM D: CRIZOTINIB – ALK/MET/ROS1 INHIBITOR

Lead Investigator: Professor Sanjay Popat

25.1 Background & Rationale

25.1.1 Molecular Cohorts:

Inhibitor: Crizotinib

Arm	Investigational Medicinal Product	Cohort Number	Histology	Molecular Cohort
**D**	Crizotinib – ALK/MET/ROS1 Inhibitor	**D1**	NSCLC	MET amplification
		**D2** *Closed to recruitment*	NSCLC	ROS1 gene fusions
		**D3**	NSCLC	MET exon 14 skipping (splice mutation or deletion)

25.1.2 Pre-clinical Rationale

Crizotinib (PF-02341066) is a selective ATP-competitive small-molecule oral inhibitor of the ALK, c-Met/hepatocyte growth factor receptor (HGFR), Recepteur d'Origine Nantais (RON), and ROS1 receptor tyrosine kinases and their oncogenic variants (e.g. c-Met/HGFR mutations and ALK or ROS1 fusion proteins). Consistent with this mechanism of action, crizotinib inhibited phosphorylation of c-Met/HGFR and selected ALK fusion or mutant variants in tumour cells both *in vitro* and *in vivo* and RON and ROS1 *in vitro*.

Crizotinib exhibited potent and selective growth inhibitory activity against tumour cells exhibiting translocation/inversion or selected mutations involving the ALK gene locus (i.e., EML4-ALK or nucleophosmin (NPM)-ALK fusion variants), exhibiting translocation of the ROS1 gene locus, or amplification of the c-Met/HGFR gene locus.

Crizotinib demonstrated antitumour efficacy, including marked cytoreductive antitumour activity, in multiple tumour models implanted in athymic mice that expressed activated crizotinib targets providing further rationale for study in clinical trials.

25.1.3 Clinical Data

25.1.3.1 Marketing Experience

Currently, crizotinib is already licensed in the European Union and approved in more than 90 countries worldwide for the first-line treatment of adults with ALK positive advanced NSCLC; the treatment of adults with previously treated ALK-positive advanced NSCLC.

Approvals for the treatment of adults with ROS1-positive advanced NSCLC were granted in the US and EU in March 2016 and August 2016 respectively. Currently, crizotinib is approved in more than 40 countries worldwide, and marketing applications for this indication are planned or currently in review in several countries worldwide.

25.1.4 Cohort definition

25.1.4.1 D1 - MET amplified NSCLC

Crizotinib was demonstrated to be a potent ATP-competitive inhibitor of recombinant human ALK and c-Met/HGFR RTKs in biochemical kinase assays. Crizotinib demonstrated a mean Ki value of 0.5 nM for inhibition of recombinant human ALK and a mean Ki value of 0.62 nM for inhibition of recombinant human c-Met/HGFR. Crizotinib inhibited hepatocyte growth factor (HGF)-stimulated or constitutive total tyrosine phosphorylation of wild type c-Met/HGFR with a mean IC_{50} value of 11 nM across a panel of human tumour cell lines and demonstrated similar values in mouse or canine epithelial cells.

In a series of cell-based functional assays, crizotinib potently inhibited human GTL-16 gastric carcinoma cell (Met amplified, non-mutated) growth (IC_{50} = 9.7 nM), HGF-stimulated human NCI-H441 lung carcinoma cell migration and invasion through a matrigel Matrix (IC_{50} values of 11 nM and 6.1 nM, respectively), and HGF-stimulated MDCK cell motility/scattering (IC_{50} = 16 nM). To also investigate potential antiangiogenic activity, crizotinib inhibited HGF-mediated HUVEC endothelial cell survival (IC_{50} = 11 nM) and matrigel invasion (IC_{50} = 35 nM) as well as HMVEC endothelial cell tubulogenesis in fibrin gels (IC_{50} = ~80 nM). These data suggest that antitumour efficacy of crizotinib may be mediated by both direct effects on tumour cell growth or survival as well as antiangiogenic mechanisms.

The antitumour efficacy of crizotinib was evaluated in a variety of human tumour xenograft models representative of cancer indications in which dysregulation of c-Met/HGFR is implicated. Crizotinib demonstrated potent cytoreductive activity in both the GTL-16 gastric carcinoma (mean 60% regression) and the NCI-H441 non-small cell lung carcinoma (75% cells contain 3-5 copies of non-mutated MET on FISH) (mean 48% regression) models, suggesting the potential to monitor patient objective response as a means of assessing clinical efficacy. Crizotinib also demonstrated near complete tumour growth inhibition in both the U87MG glioblastoma (97% growth inhibition) and PC-3 prostate carcinoma models (87% growth inhibition) at 50 mg/kg/day. In models in which multiple dose levels were evaluated, (GTL-16, U87MG, and NCI-H441), dose-dependent inhibition of tumour growth that correlated with dose-dependent inhibition of c-Met/HGFR phosphorylation was demonstrated. In these studies it was observed that complete inhibition of c-Met/HGFR was achieved for the full dosing interval at 50 mg/kg/day and that dose levels greater than 50 mg/kg/day did not demonstrate a further improvement in antitumour efficacy. In addition, crizotinib was generally well tolerated with no overt toxicity, weight loss, or target organ histopathology observed up to a 200-mg/kg/day dose level for up to 30 days of administration in any of the studies.

To evaluate the PD inhibition of c-Met/HGFR by crizotinib, GTL-16 gastric carcinoma tumours were harvested at several time points following oral administration of crizotinib in both single-dose and repeat-dose (steady-state) studies. c-Met/HGFR phosphorylation status in tumours was quantitated by ELISA over a range of doses. With focus on steady-state PD studies (11-day administration) to draw a correlation with tumour growth inhibition, crizotinib demonstrated the following:

At 50 mg/kg/day QD: 100% tumour growth inhibition correlated with complete inhibition of c-Met/HGFR phosphorylation in GTL-16 tumours sustained for 24 hours (25 mg/kg: near complete inhibition of both phosphorylation and tumour growth). At 12.5 mg/kg/day QD: 60% tumour growth inhibition correlated with 80% to 90% inhibition of c-Met/HGFR phosphorylation at 1 to 8 hours which decreased to 50% to 60% inhibition by 16 to 24 hours.

At 6.25 mg/kg/day QD: non-significant trend toward tumour growth inhibition correlated with 30 to 50% inhibition of c-Met/HGFR phosphorylation at 1 to 8 hours with full recovery by 16 hours.

When defining the relationship of target PD to efficacy in the GTL-16 model, the following conclusions were apparent:

> 1) Complete inhibition of c-Met/HGFR activity for 24 hours is consistent with complete inhibition of tumour growth (50 mg/kg/day, 100% TGI),
>
> 2) Potent inhibition of c-Met/HGFR activity for only a portion of the schedule is consistent with suboptimal efficacy (12.5 mg/kg/day, 60% TGI), and 3) inability to achieve >50% inhibition of c-Met/HGFR activity (3.125, 6.25 mg/kg/day) is consistent with lack of significant tumour growth inhibition.

These findings suggest that the duration of c-Met/HGFR inhibition is important to maximize antitumour efficacy of crizotinib. Furthermore, a similar dose-dependent effect of crizotinib on tumour growth and c-Met/HGFR phosphorylation was observed utilizing other tumour models (NCI-H441, U87MG), further these supporting observations. The importance of maximizing the duration of HGFR inhibition is also illustrated by comparison of QD and BID administration of crizotinib in the GTL-16 model. The degree of GTL-16 growth inhibition observed at A) 12.5 mg/kg twice daily was comparable to that observed at dose levels of 25 or 50 mg/kg administered once daily and at B) 6.25 mg/kg twice daily was comparable to that observed at dose levels of 12.5 mg/kg administered once daily. Collectively, these studies suggest that near complete inhibition of c-Met/HGFR phosphorylation (>90% inhibition) for the duration of the administration schedule is necessary to maximize therapeutic benefit.

Proof of principle that crizotinib could be a highly effective therapy for patients with MET-amplified NSCLC have come in two published case reports. In the first, a patient with stage IV adenocarcinoma with high-level MET amplification (ALK rearrangement negative) was treated with crizotinib second line whilst progressing on bevacizumab maintenance after first line gem/carbo/bevacizumab, in the A8081001 trial MET-enrichment cohort (Ou *et al.* 2011). The entry criteria for this cohort was MET/CEP7 ratio >2.2 but not polysomy. The patient had a sustained partial response to therapy with grade I bradycardia and grade I visual disturbance as only side effect. More recently a chemotherapy naïve patient with squamous cell cancer with Met amplification defined as above had a near CR to crizotinib (Schwab *et al.* 2014). Camidge presented data at ASCO 2014 using crizotinib in 14 MET amplified patients. In those with high amplification (>5 MET/CEP7 ratio) there was a 67% response rate. In the 6 patients with >2.2-5 fold amplication there was one confirmed response and 2 unconfirmed responses (Camidge *et al.* 2014).

In a series of 435 surgically resected NSCLC cases the frequency of true MET amplification was 4.1% and there was no association with histology (Cappuzzo *et al.* 2009). This is very similar to the 3.9% amplification rate in a Korean surgical series, where 6/7 amplified patients had squamous cell cancer (Go *et al.* 2010). In the A8081001 screening programme 2/94 patients were found to have de novo amplification, but screening was skewed towards screening mostly never smokers with adenocarcinoma. Although the figure of FISH positivity can be significantly increased by adding high polysomy patients it is worth noting that in the first case report above a patient with high MET polysomy but not amplified treated with crizotinib was described – the patient had progressive disease at 6 weeks. Finally, initial treatment with chemotherapy appears to increase Met gene dosage (high Met gene dosage in 35.7% stage III/IV NSCLC patients with prior chemotherapy versus 7.4% without): thus patients negative for an actionable biomarker on the original biopsy may demonstrate acquisition of MET amplification on repeat post-first line biopsy (Chen *et al.* 2011).

25.1.4.2 D2 - NSCLC harbouring ROS1 gene fusions

Crizotinib demonstrated inhibitory activities against total tyrosine autophosphorylation of the related ROS1 RTK with a mean IC_{50} value of 47 nM in HCC78 lung adenocarcinoma cells engineered expressing the SLC34A-ROS fusion RTK. Crizotinib was evaluated for its ability to inhibit ROS1-dependent phenotypes including cell proliferation and cell survival at concentrations consistent with inhibition of ROS1 phosphorylation. Crizotinib inhibited cell proliferation (Concentration that results in 50% effect [EC_{50}] = 59 nM) at concentrations comparable to those required to inhibit ROS1 phosphorylation (EC_{50} = 44 nM) in HCC78 lung adenocarcinoma cells expressing the SLC34A-ROS fusion variant.

To evaluate the antitumour activity of crizotinib in ROS1-driven tumour models, athymic mice bearing established NIH-3T3 tumours expressing selected ROS1 fusion variants were orally administered crizotinib at 75 mg/kg twice a day for 11 to 17 days. Near complete tumour regression was observed following 5-7 days of crizotinib treatment for each of the five NIH-3T3-ROS models. Two independent dose-response studies were conducted in NIH-3T3-CD74-ROS and NIH-3T3-SLC34A2-ROS(L) models to investigate the effect of crizotinib on 1) inhibition of ROS1 phosphorylation, 2) tumour growth, and 3) the relationship of crizotinib plasma concentration to inhibition of tumour growth. Crizotinib demonstrated dose-dependent inhibition of ROS1 phosphorylation in each of the NIH-3T3-CD74-ROS1 and NIH-3T3-SLC34A2-ROS1(L) xenograft models in athymic mice. Dose-dependent inhibition of ROS1 phosphorylation in tumours following crizotinib administration was correlated with dose dependent tumour growth inhibition and tumour regression in these two studies. Crizotinib demonstrated marked tumour regression (84% to 95%) in the NIH-3T3-CD74-ROS1 and NIH-3T3-SLC34A2-ROS1(L) tumours following 9-10 days of oral administration at the 80-160 mg/kg/day dose levels, and significantly inhibited the tumour growth by 34%-43% and 78%-91% at the 20 mg/kg/day and 40 mg/kg/day dose levels in each tumour xenograft model. There was no body weight loss observed in either study following crizotinib treatment. PK/PD relationships between crizotinib plasma concentrations and the dose-dependent tumour growth inhibition from the NIH-3T3-CD74-ROS1 study and the NIH-3T3-SLC34A2-ROS1(L) study were quantitatively modelled by tumour growth inhibition models. Based on the modelling results, a free plasma concentration which was correlated to the effect of tumour stasis--Cstasis (100% tumour growth inhibition [TGI]) was estimated to be 84 to 99 nM. Collectively, the combination of studies in the NIH-3T3-CD74-ROS1 and the NIH-3T3-SLC34A2-ROS1(L) xenograft models indicated that the extent of ROS1 phosphorylation inhibition was directly linked to the level of anti-tumour efficacy and that significant inhibition of ROS1 kinase activity during the entire treatment period was necessary to achieve robust anti-tumour activity (i.e., complete TGI). Based on these results, the concentrations associated with tumour stasis—Cstasis of 84 nM to 99 nM is required to achieve significant antitumour efficacy (100% TGI) in the NIH-3T3-CD74-ROS1(s) and the NIH-3T3-SLC34A2-ROS1(L) xenograft models.

Proof of principle that crizotinib could be a useful therapy for ROS1 rearranged adenocarcinoma came in large analysis of the frequency and clinical characteristics of ROS1 rearranged adenocarcinoma. Of 1073 tumours assessed by FISH, 1.7% demonstrated ROS1 rearrangements with no case harbouring a concomitant ALK rearrangement (Bergethon *et al*. 2012) The clinical characteristics of patients with ROS1 rearrangements were similar to those with ALK rearrangements – median age 50, principally never smokers and all with adenocarcinoma. A 31 year old patient with ROS1 rearranged BAC was described: 8 weeks therapy with crizotinib resulted in a near CR with no sign of relapse at 6 months. This group have updated their results at ASCO 2013 (Ou *et al.* 2013). A total of 50 ROS1 rearranged patients treated with crizotinib were evaluable for response. The objective response rate was 72% (95% confidence interval [CI], 58 to 84), with 3 complete responses and 33 partial responses. The median duration of response was 17.6 months (95% CI, 14.5 to not reached). Median progression-free survival was 19.2 months (95% CI, 14.4 to not reached), with 25 patients (50%) still in follow-up for progression. Among 30 tumours that were tested, we identified 7 ROS1 fusion partners: 5 known and 2 novel partner genes. No correlation was observed between the type of *ROS1* rearrangement and the clinical response to crizotinib. The

safety profile of crizotinib was similar to that seen in patients with *ALK*-rearranged NSCLC. The National Lung Matrix Trial ROS1 cohort D2 will generate data to complement this programme and a third study being performed in East Asia.

25.1.4.3 D3 - MET exon 14 skipping (splice mutation or deletion) - NSCLC

Somatic mutations affecting exon 14 of *MET* (*METex14*) were initially reported in NSCLC and SCLC specimens and cell lines (Ma *et al.* 2003; Ma *et al.* 2005; Kong-Beltram *et al.* 2006). This and other somatic aberrations that result in exon 14 loss, result in MET kinase activation. *MET* exon 14 encodes a number of prominent amino acids including Y1003 in a DpYR motif, which is required for efficient recruitment of the ubiquitin ligase CBL that functions to target MET for ubiquitin-mediated degredation (Peschard *et al.* 2001). MET exon 14 loss by splicing mutations maintain the reading frame and results in increased MET stability and prolonged signalling via HGF stimulation, and therefore oncogenic potential (Frampton 2015).

Preclinical models have confirmed that *METex14* variants have increased MET activity (Frampton *et al.* 2015) are transforming, similar to CBL mutant models (Peschard *et al.* 2001), and are sensitive to MET inhibitors *in vitro*.

The largest comprehensive analysis of *METex14* variants comes from the Foundation Medicine laboratory (Frampton *et al.* 2015). Here, 38,028 specimens from malignancy undergoing panel-based sequencing for routine clinical care were examined for *METex14* alterations. 224 distinct *METex14* alterations were identified from 221 specimens consisting of 126 different genomic sequence variants including substitutions and indels at splice acceptor sites, in the ~25bp intronic non-coding region immediately adjacent to the splice acceptor site, as well as whole exon 14 deletions. 193 alterations were identified from lung NSCLCs; with a prevalence of 3% in adenocarcinomas (n=131). Other lung histologies identified included adenosquamous (n=6), large cell carcinoma (n=2), carcinosarcoma (n=1), sarcomatoid carcinoma (n=3), NOS (n=33), squamous cell (n=15). Whilst a statistically significant difference in rates of *METex14* alteration between differing NSCLC histologies was not identified (Frampton 2015), histology prevalence in this series will have been biased by NSCLC histological sub-types sent to Foundation Medicine for genotyping in routine clinical care. One case of an insertion in an undifferentiated SCLC was also identified. Focussing on adenocarcinomas with *METex14* alterations, amplification at *MET*, *MDM2* and *CDK4* was common. However, co-occurrence with other known classical drivers of adenocarcinoma was rare.

Similar findings have been reported from other much smaller series. A Japanese series of resected lung cancers (all but one being NSCLC) demonstrated *METex14* variants only in adenocarcinomas at a prevalence of 3.3% (7 of 211 cases). These cases were mutually exclusive to *EGFR*, *KRAS*, and *ERBB2 (HER2)* mutations (Onozato *et al.* 2009). A series of 87 resected adenocarcinomas from Korea, identified 3 (3.4%) patients with *METex14* skipping events by transcriptome sequencing (Seo *et al.* 2012).

Case reports of NSCLCs harbouring *METex14* variants have confirmed *in vitro* findings of clinical sensitivity to MET inhibitors (Table 34). The Paik (2015) series identified 8 patients with *METex14* variants, 4 of whom were treated with MET inhibitors (cabozantinib or crizotinib). Whilst all but one of the 8 patients reported had adenocarcinoma sub-type NSCLC, importantly one was a pleomorphic adenocarcinoma with squamous and spindle cell components.

Liu *et al.* (2015) have presented genomic data on a series of 36 patients with sarcomatoid NSCLC; a distinct NSCLC histological variant (Brambilla *et al.* 2001) with poor prognosis, clinical insensitivity to cytotoxic chemotherapy (Vieira *et al.* 2013), and high incidence of MET over-expression (86% sarcomatoid adenocarcinomas, Weingertner 2015). Here, 8 (22%) were

identified to harbour *METex14* variants. Three were 5' splice site deletions, and five were 3' donor splice site point mutations. Data on one patient treated with crizotinib was presented, demonstrating a dramatic response.

Data from limited cases and small case series therefore demonstrates *METex14* variants occurring in a variety of NSCLC histological sub-types, including adenocarcinoma and sarcomatoid carcinomas, with those treated with MET inhibitors generally demonstrating clinical and radiological responses. A larger systematically attained cohort of NSCLC patients with *METex14* is therefore required to robustly evaluate the incidence of *METex14* in an unselected NSCLC population by histological sub-type, to quantify the magnitude of response to therapy in such cases with crizotinib, to evaluate sites and patterns of disease progression, and to characterize biological mechanisms of acquired crizotinib-resistance.

Table 34: NSCLC METEx14 cases reported treated with MET inhibitors with outcomes.

Author	Age	Sex	Smoking status (pack-years)	Histology	MET variant	Other variants	MET therapy	CT Response	Duration of MET therapy (months)
Frampton 2015	82	F	25	Large cell	c.3028G>C	TP53 p.N30fs*14	capmatinib	PR	5+
Frampton 2015	64	F	4	SCC	c.3028+1G>T	NR	capmatinib	PR	13
Jenkins 2015	86	M	0	ADC	c.2887-18_2887-7del12	CDKN2A/B loss, CDK4 amp, MDM2 amp	crizotinib	PR	1.1*
Waqar 2015	71	M	15	ADC	c.3082G>C	NR	crizotinib	PR	6+
Mendenhall 2015	76	F	Ex-light	SCC	D1010H	MDM2 amp	crizotinib	PR	4.4+
Paik 2015	80	F	0	ADC	c.3028G>C	RB1 deletion, DICER1 Q1776X, EPHA5 R853Q, KLF4 M1I, MLL I1929M, MTOR D537N, TERT G225E, FUBP1 amp, GSK3B amp, SDHA amp, TERT amp, MET amp, KDM5A amp, RAD52 amp, MDM2 amp, BCL2L1 amp, NKX3-1 del,ETV6 del	cabozantinib	SD	5.1+
Paik 2015	80	F	20	ADC	c.3024_3028del AGAAGGTATATT	TP53 splice variant, FAT1 R782fs, FBXW7 G539V, MLL E1678K, NF1 E572fs, NTKR2 I191T, YES1 amp	crizotinib	mixed	3.6
Paik 2015	65	M	20	ADC	p.V1001_F1007 del	TP53 R248P, RB1 deletion, BRCA1 E648Q, MYC E137D, NF1 splice variant, NDS1 E1902K, PDGFRB R397W, TERT gain, MET amp, MYC amp, NKX2-1 amp	crizotinib	PR	4.6+

Author	Age	Sex	Smoking status (pack-years)	Histology	MET variant	Other variants	MET therapy	CT Response	Duration of MET therapy (months)
Paik 2015	90	F	0	ADC	c.3028G>T	CDK4 amp, MDM2 amp	crizotinib	PR	3.1+
Liu 2015	74	F	NR	Sarcomatoid	NR	MET amp	crizotinib	PR	NR

ADC, adenocarcinoma; F, female; M, male; NR, not reported; PR, partial response; SCC, squamous cell carcinoma; SD, stable disease; *drug discontinued due to toxicities, +, therapy ongoing at time of reporting

Table 35: Specific aberration rules for Arm D cohorts

Drug	Arm	Molecular Cohort	Specific Aberration	Rule
Crizotinib	D1	MET amplification	Amplification > 3 copies	Focal > 3, otherwise >4
	D2	ROS1 gene fusions	GOPC-ROS1_G8:R35<br>GOPC-ROS1_G4:R36<br>SLC34A2-ROS1_S4:R32<br>CD74-ROS1_C6:R34<br>CD74-ROS1_C6:R32<br>SLC34A2-ROS1_S13del2046:R32<br>SDC4-ROS1_S2:R32<br>EZR-ROS1_E10:R34<br>LRIG3-ROS1 _L16:R35<br>TPM3-ROS1_T8:R35<br>SDC4-ROS1_S4:R32<br>SDC4-ROS1_S4:R34<br>GOPC-ROS1_G7:R35<br>CCDC6-ROS1_C6:R34<br>SLC34A2-ROS1_S4:R34<br>SLC34A2-ROS1_S13del2046:R34	Specific and novel translocations
	D3	MET exon 14 skipping (splice mutation or deletion)	Listed in current version of Technology Hub Manual	N/A

25.2 Specific Eligibility Criteria

25.2.1 Inclusion Criteria

Patients must meet all of the following inclusion criteria to be eligible for enrollment into the trial:

- Patients must fulfil all the core eligibility criteria.
- Technology hub result (or locally obtained result from an approved Laboratory if applicable.
- Eastern Cooperative Oncology Group (ECOG) Performance Status <u><</u>2 with no deterioration over the previous 2 weeks (see Appendix 8: Eastern Cooperative Oncology Group Performance Status Criteria).
- Ability to swallow oral medication.
- Adequate hepatic function in patients **with** liver metastases: AST and/or ALT ≤ 5.0 x ULN.

25.2.2 Exclusion Criteria

Patients should not enter the trial if any of the following exclusion criteria are fulfilled:

- Patients who do not fulfil all the core eligibility criteria.
- Treatment with any of the following:
    - Concomitant use of medications known to prolong QT interval within 6 half-lives plus 1 day prior to the first dose of crizotinib (see Section 25.2.3.1.1).
    - Food or drugs known to be strong CYP3A4 inhibitors, potent CYP3A4 inducers or substrates within the required washout period prior to the first dose of crizotinib:
        - Potent CYP3A4 inducers within 12 days (see Section 25.2.3.2.2).
        - Strong CYP4A4 inhibitors within 7 days (see Section 25.2.3.2.1).
        - CYP3A4 substrates with narrow therapeutic indices within 5 half-lives prior to the first dose of crizotinib (see Section 25.2.3.2.3).
        - The topical use of these medications (if appropriate), such as 2% ketoconazole cream is permitted.
    - Radiotherapy within 4 weeks prior to the first dose of crizotinib (see Section 25.2.3.3).
    - Any other chemotherapy, investigational agents or other anti-cancer therapy within 4 weeks prior to the first dose of crizotinib.
- Any of the following within the 3 months prior to registration (unless otherwise specified): Myocardial infarction, severe/unstable angina, coronary/peripheral artery bypass graft, congestive heart failure or cerebrovascular accident including transient ischemic attack. Appropriate treatment with anticoagulants is permitted.
- Uncontrolled or significant cardiovascular disease including, but not limited to: clinically significant heart disease (e.g. congestive heart failure with New York Heart Association functional class III-IV (see Appendix 10: New York Heart Association Classification – Stages of Heart Failure), pericarditis, pericardial effusion, uncontrolled angina), ongoing cardiac dysrhythmias of NCI CTCAE Grade ≥2, uncontrolled atrial fibrillation of any grade, or machine-read ECG with QTcF interval >470 msec.
- History of extensive disseminated/bilateral or known presence of Grade 3 or 4 interstitial fibrosis or interstitial lung disease, including a history of pneumonitis, hypersensitivity pneumonitis, interstitial pneumonia, interstitial lung disease, obliterative bronchiolitis, and pulmonary fibrosis, but not history of prior radiation pneumonitis.
- Spinal cord compression unless treated with the patient attaining good pain control and stable or recovered neurologic function.
- Carcinomatous meningitis or leptomeningeal disease.
- Severe gastrointestinal conditions such as diarrhoea or ulcer. Patients at risk of gastrointestinal conditions e.g. history of diverticulitis, metastases to the gastrointestinal tract, concomitant use of medications with a recognised risk of gastrointestinal perforation) must be excluded.

25.2.3 Restrictions & Concomitant Medications

Information on any treatment from the date of informed consent until 28 days after the administration of the last treatment dose should be recorded. If medically feasible, patients taking regular medication should be maintained on it throughout the study period. Patients should be advised to inform their treating physicians of all concomitant medications, including prescription medicines, over-the-counter drugs, vitamins, and herbal products.

Nb. These lists are not exhaustive and the absence of a drug from the lists does not imply that its combination with crizotinib is safe.

25.2.3.1 Pharmacodynamic Interactions

25.2.3.1.1 QT interval prolongation

In clinical studies, prolonged QT interval was observed with crizotinib.

Therefore, concomitant medications **known to prolong the QT interval**, or **with any risk of prolonging the QT interval** are prohibited during the active treatment phase. Any patients taking such drugs at or prior to registration should discontinue the drug within 6 half lives plus 1 day prior to commencing trial treatment.

For a list of drugs known to prolong the QT interval, or with any risk of prolonging the QT interval, please refer to the following database: https://www.crediblemeds.org. Appendix 9 Credible Meds List of Drugs that Prolong QT Interval contains a list exported from this database on 2nd March 2018. **It is important to note that this list is a guide – the database will change with time and therefore needs to be checked in real-time when screening and registering a patient, and throughout their treatment. The Trials Office will email all sites when updates are made to the database.**

Please note that for Pfizer trial Arms, all concomitant medications listed on https://www.crediblemeds.org under the following 3 categories will exclude patients from entering the trial as per the eligibility criteria, unless an exception applies:

- **'Known risk of TdP'**
- **'Possible risk of TdP'**
- **'Drugs to Avoid in Congenital Long QT'**

The following exceptions may apply:

- The category '**Drugs to Avoid in Congenital Long QT**' includes drugs that should be avoided in patients with diagnosed or suspected congenital long QT syndrome. Patients without any evidence of these conditions may continue taking drugs allocated to this category alone and would be therefore eligible for inclusion in the trial.
  Important note: 'Drugs to Avoid in Congenital Long QT' category may also be assigned to drugs in parallel with either of the following 2 categories '**Known risk of TdP**' and '**Possible risk of TdP'**; these concomitant medications are still prohibited for all trial patients and therefore patients would be ineligible for inclusion in the trial.
- Patients receiving treatment with a concomitant medication within the risk category **'Conditional Risk of TdP'** are **not** excluded from trial entry into Arm C, regardless of whether they are also assigned the **'Drugs to Avoid in Congenital Long QT'** category in addition. (Medications within this category are not listed within Appendix 9 Credible Meds List of Drugs that Prolong QT Interval but will still appear on https://www.crediblemeds.org).

It is the responsibility of an Investigator (Consultant level) to review and clinically evaluate all concomitant medications. Please contact the National Lung Matrix Trial Office for clarification regarding any drugs that appear on the database that are not listed in Appendix 9 Credible Meds List of Drugs that Prolong QT Interval.

The drugs listed on this website are taken from information provided by The Arizona Centre for Education and Research on Therapeutics and The Critical Path Institute, Tucson, Arizona and Rockville, Maryland. Important Note - If a patient is being treated with such medication or is taking another medication that may affect QT interval which is not on the database, please contact the Trial Office to obtain the recommended withdrawal/ minimum period prior to starting trial treatment.

25.2.3.1.2 Bradycardic agents

Bradycardia has been reported during clinical studies; therefore, crizotinib should be used with caution due to the risk of excessive bradycardia when used in combination with other bradycardic agents (e.g., nondihydropyridine calcium channel blockers such as verapamil and diltiazem, betablockers, clonidine, guanfacine, digoxin, mefloquine, anticholinesterases, pilocarpine) (see Sections 25.3.3.3 and 25.3.3.4).

25.2.3.2 Pharmacokinetic Interactions

Crizotinib is a substrate of CYP3A4/5 and also a moderate inhibitor of CYP3A. *In vitro* studies in human liver microsomes demonstrated that crizotinib is a time-dependent inhibitor of CYP3A.

The concomitant use of crizotinib with strong CYP3A4 inhibitors or with strong and moderate CYP3A4 inducers should be avoided during treatment and are prohibited prior to the first dose of trial treatment. The concomitant use of crizotinib with CYP3A4 substrates with narrow therapeutic indices should be avoided during treatment and are also prohibited prior to the first dose of crizotinib (see below or Section 25.2.2 for relevant washout periods).

25.2.3.2.1 Agents that may increase crizotinib plasma concentrations (strong CYP3A4 inhibitors)

Co-administration of crizotinib with strong CYP3A inhibitors may increase crizotinib plasma concentration. The concomitant use of strong CYP3A inhibitors, including but not limited to certain protease inhibitors, azole antifungals or marcrlides such as atazanavir, clarithromycin, indinavir, itraconazole, ketoconazole, nelfinavir, ritonavir, saquinavir, telithromycin, troleandomycinand voriconazole, should be avoided during treatment and are prohibited within 7 days prior to the first dose of crizotinib. Grapefruit or grapefruit juice may also increase plasma concentrations of crizotinib and should be avoided. The effect of CYP3A inhibitors on steadystate crizotinib exposure has not been established.

25.2.3.2.2 Agents that may decrease crizotinib plasma concentrations (potent CYP3A4 inducers)

Co-administration of crizotinib with strong CYP3A inducers may decrease crizotinib plasma concentrations. The concurrent use of strong CYP3A inducers, including but not limited to carbamazepine, efavirenz, phenobarbital, phenytoin, rifabutin, rifampin, and St. John's Wort, should be avoided during treatment, and are prohibited within 12 days prior to the first dose of crizotinib.

25.2.3.2.3 Agents whose plasma concentrations may be altered by crizotinib (substrates)

Crizotinib has been identified as an inhibitor of CYP3A both *in vitro* and *in vivo* as described in Section 6.1 of the IB. Co-administration of crizotinib with CYP3A4 substrates with narrow therapeutic indices associated with life-threatening arrhythmias including, but not limited to, dihydroergotamine, ergotamine, pimozide, astemizole*, cisapride*, and terfenadine * (*drugs not marketed in all countries) must be avoided during crizotinib treatment and are not permitted within 5 half-lives prior to the first dose of crizotinib.

Caution must be exercised in patients receiving crizotinib in combination with other CYP3A4 substrates, particularly those with narrow therapeutic indices, including but not limited to, alfentanil, cyclosporine, fentanyl, quinidine, sirolimus, and tacrolimus.

In addition to drug interactions due to enzyme inhibition and induction, the possibility of an additive pharmacodynamic interaction of crizotinib and other negatively chronotropic medications (e.g., beta-blockers and non-dihydropyridine calcium-channel blockers) should be considered. Co-administration of crizotinib with these medications may lead to significant decreases in heart rate.

Monitor heart rate and blood pressure regularly. In cases of symptomatic bradycardia (including syncope, dizziness, hypotension) that is not life-threatening, hold crizotinib until recovery to asymptomatic bradycardia or to a heart rate of 60 bpm or above, re-evaluate the use of concomitant medications, and adjust the dose of crizotinib in accordance to the protocol.

The topical use of medications that are potent CYP3A4 inducers, strong CYP3A4 inhibitors or CYP3A4 substrates with narrow therapeutic indices (if appropriate), such as 2% ketoconazole cream is permitted. If in doubt, please check with the Trial Office.

Crizotinib is an inhibitor of CYP2B6 *in vitro*. Therefore, crizotinib may have the potential to increase plasma concentrations of co-administered drugs that are metabolized by CYP2B6. These agents should be administered with caution.

Crizotinib is an inhibitor of P-gp, OCT1 and OCT2 *in vitro*. Therefore, crizotinib may have the potential to increase plasma concentrations of co-administered drugs that are substrates of P-gp, OCT1 or OCT2. These agents should therefore be administered with caution.

25.2.3.2.4 Coadministration with medications that increase gastric pH

The aqueous solubility of crizotinib is pH dependent, with low (acidic) pH resulting in higher solubility. Administration of a single 250 mg crizotinib dose following treatment with esomeprazole 40 mg once daily for 5 days resulted in an approximately 10% decrease in crizotinib total exposure (AUCinf) and no change in peak exposure (Cmax); the extent of the change in total exposure was not clinically meaningful. Therefore, starting dose adjustment is not required when crizotinib is coadministered with agents that increase gastric pH (such as proton pump inhibitors, H2 blockers, or antacids).

25.2.3.3 Concomitant Radiotherapy

Concurrent radiotherapy is not permitted. If palliative radiotherapy is indicated, the patient should stop taking crizotinib 24 hours before radiation commences and should not recommence trial treatment for 24 hours after radiation. This should be decided on consultation with the National Lung Matrix Trial Office.

25.2.3.4 Food Restrictions

Grapefruit or grapefruit juice may increase plasma concentrations of crizotinib and should be avoided during treatment with crizotinib.

25.2.3.5 Other Restrictions

St John's wort should be avoided during treatment with crizotinib.

25.2.3.6 Contraception

Women of childbearing potential should be advised to avoid becoming pregnant while receiving crizotinib. Adequate contraceptive methods should be used during therapy, and for at least 90 days after completing therapy. A definition of females of childbearing potential and

females of non-childbearing potential and acceptable methods of contraception are described in Section 6.3.

Crizotinib can cause foetal harm when administered to a pregnant woman based on its mechanism of action. There are no adequate and well-controlled studies of crizotinib in pregnant women.

It is not known whether crizotinib and its metabolites are excreted in human milk. Because many drugs are excreted in human milk and because of the potential for serious adverse reactions in nursing infants from crizotinib, consider whether to discontinue nursing or to discontinue the drug, taking into account the importance of the drug to the mother.

Based on nonclinical safety findings, male and female fertility may be compromised by treatment with crizotinib. Both men and women should seek advice on fertility preservation before treatment.

25.3 Trial Treatment

25.3.1 Investigational Medicinal Product

Crizotinib will be provided free of charge by Pfizer.

The recommended dose schedule is 250 mg bd taken continuously; a 250 mg capsule is to be taken orally twice daily with or without food.

If a dose is missed, it should be taken as soon as the patient remembers, unless it is less than 6 hours until the next dose, in which case the missed dose should not be taken and dosing should resume at the next prescribed dose. Vomited doses must not be made up. Double doses are not permitted.

Patients experiencing investigational product related toxicity may have their dose modified (see dose modification Section 25.3.4).

Both the Arm D Patient Information Sheet and Arm D Patient Diary contain more specific instructions for patients to follow regarding how to take their medication.

Please note patients who meet RECIST criteria for progressive disease (PD) may be continued on trial treatment if the treatment is tolerable and the Investigator believes it to be of clinical benefit; see Section 9.3.

Please also refer to the Pharmacy Manual for further details.

25.3.2 Schedule of Assessments

Table 36: Crizotinib - Schedule of Assessments

	**Screening**	**Treatment - 250 mg twice daily (21 day cycles)**				**Discontinuation** (+ 7 days)***	**28 Day Follow Up[u]** (+ 7 days)***	**Post-28 day follow up** (± 7 days)****
	Within 28 days of treatment (unless otherwise stated)	Cycle 1, 2 & 3			Cycle 4 onwards[t]			
		Day 1	Day 8 (± 1 day)*	Day 15 (± 2 days)**	Day 1 (± 2 days)**			
Informed consent[a]	X							
Demography & baseline characteristics[b]	X							
Medical history[c]	X							
Inclusion / exclusion criteria[d]	X							
Physical examination[e]	X	X	X	X	X	X		
ECOG performance status	X (within 14 days of treatment)	X			X	X		
Vital signs (inc. weight)[f]	X	X			X	X		
ECG[g]	X (single)	X				X		
MUGA / ECHO[h]		If clinically indicated						
Ophthalomogy Examination[i]	X	If clinically indicated						
Haematology, Clinical chemistry[j]	X (within 7 days of treatment)	X	X (- 2 days)	X (- 2 days)	X (- 2 days)	X		

	Screening	Treatment - 250 mg twice daily (21 day cycles)				Discontinuation (+ 7 days)***	28 Day Follow Up[u] (+ 7 days)***	Post-28 day follow up (± 7 days)****
	Within 28 days of treatment (unless otherwise stated)	Cycle 1, 2 & 3			Cycle 4 onwards[t]			
		Day 1	Day 8 (± 1 day)*	Day 15 (± 2 days)**	Day 1 (± 2 days)**			
Liver Function Tests[k]	X (within 7 days of treatment)	X	X (- 2 days)	X (- 2 days)	X (- 2 days)	X		
Pregnancy test[l]	X	X (Cycle 1 only)				X		
Tumour assessments[m]	X	Every 6 weeks during year 1 (± 7 days) [except 1st scan + 7days only]						X ◊
Adverse events & Concomitant Medications	X	X	X	X	X	X	X	
Dispense study drug		X			X			
Administer study drug[n]		BD Dosing						
Smoking status[o]		X (Cycle 1 only)			X (every 9 weeks)	X		
Germline DNA sample[p]		X (Cycle 1 only)						
ctDNA samples[q]		X (Cycle 1 only)			X (every 9 weeks beginning cycle 4) (- 2 days)	X		X $

	Screening	Treatment - 250 mg twice daily (21 day cycles)				Discontinuation (+ 7 days)***	28 Day Follow Up[u] (+ 7 days)***	Post-28 day follow up (± 7 days)****
	Within 28 days of treatment (unless otherwise stated)	Cycle 1, 2 & 3			Cycle 4 onwards[t]			
		Day 1	Day 8 (± 1 day)*	Day 15 (± 2 days)**	Day 1 (± 2 days)**			
Optional research biopsy[r]		X (post-reg, pre-tx); Cycle 1 only)				X		
Survival status[s]								X

* Visit may occur ± 1 days of the planned visit date. Individual assessments may occur independently of the visit date where indicated in the table above. Where applicable and acceptable in accordance to local practices, visits may be performed by telephone or video call.

** Visit may occur ± 2 days of the planned visit date. Individual assessments may occur independently of the visit date where indicated in the table above

*** Visit may occur + 7 days of the planned visit date

**** Visit may occur ± 7 days of the planned visit date

a Prior to the start of any study specific procedures, each patient must provide signed informed consent.

b Demography must be captured for all patients,. Demographic data and other characteristics will include: date of birth, gender, race /ethnicity.

c A standard medical and surgical history will be obtained, including prior cancer treatment.

d Patients must not be registered unless all eligibility criteria have been fully met.

e Physical examination includes general appearance, respiratory, cardiovascular, skin, head and neck (including ears, eyes, nose and throat), lymph nodes, thyroid, abdomen, musculo-skeletal (including spine and extremities) and neurological systems will be required at screening, pre-dose on day 1 of every cycle, day 8 and day 15 of the first three cycles and at discontinuation.

f Vitals signs are to be recorded are height (at screening only), weight, BP and pulse; required at screening, pre-dose on day 1 of every cycle and at discontinuation.

g 12-lead ECG required at screening (single), pre-dose on day 1 of cycles 1, 2 and 3 and at discontinuation.

Twelve-lead ECGs will be obtained after the patient has been resting semi-supine for at least 10 minutes prior to times indicated. All ECGs should be recorded with the patient in the same physical position. For each time point unless specifically stated above, three ECG recordings should be taken at a minimum of 5 minute intervals (all three ECGs must be collected within 30 minutes). A standardised ECG machine should be used and the patient should be examined using the same machine throughout the study if possible. After paper ECGs have been recorded, the Investigator or designated physician will review each of the ECGs and may refer to a local cardiologist if appropriate. A paper copy should be filed in the patient's medical records. If an abnormal ECG finding at screening or Cycle 1 day 1 is considered to be clinically significant by the Investigator, it should be reported as a concurrent condition. For all ECGs details of rhythm, ECG intervals (R-R, PR, QT and QRS) and an overall evaluation will be recorded.

h Only to be performed if clinically indicated. If abnormalities present, the clinical signs and symptoms associated with the abnormal finding should be reported as the AE. The modality of the cardiac function assessments must be consistent within patient and the same machine operator is to be used where possible.

i Ophthalmology examination includes visual acuity, slit lamp fundoscopy and slit lamp examination and should be performed at screening by an ophthalmologist. Repeat ophthalmologic examination should be repeated during the study when visual disturbances (e.g. visual impairment, photopsia, blurred vision, vitreous floaters) have been observed and persist or worsen in severity.

j Samples to be collected at screening (within 7 days of commencing treatment), day 1, 8 and 15 of cycles 1, 2 & 3, day 1 of subsequent cycles and at discontinuation. Samples can be taken up to 2 days earlier than the actual visit date (where indicated). Clinical Chemistry and Haematology to be performed at screening, pre-dose on day 1 of every cycle and at discontinuation. Where applicable and acceptable in accordance to local practices, blood tests can be performed locally in GP surgeries or in community based clinics.

Clinical Chemistry: calcium (total), creatinine, magnesium, sodium, urea nitrogen, glucose, uric acid, LDH, phosphate, total protein and potassium.

Haematology: FBC, International normalised ratio (INR).

k Liver function tests, including Albumin, AST, ALT, ALP, bilirubin (total) should be monitored once a week during the first 3 cycles of treatment then on day 1 of subsequent cycles until discontinuation. Samples can be taken up to 2 days earlier than the actual visit date (where indicated).

Liver Function Tests should also be repeated within 48 hours if the following is observed and repeated weekly until recovery to baseline levels. During this time, crizotinib treatment must be withheld and recommenced if appropriate after consultation of dose modification table.

- If patient entered the study with AST or ALT baseline values within the normal range, who subsequently present with AST or ALT ≥3 ULN concurrent with total bilirubin ≥2 ULN **OR**
- A patient with pre-existing AST or ALT baseline values above the normal range who subsequently present with AST or ALT ≥2 times the baseline values and ≥3x ULN or ≥8x ULN (whichever is smaller) concurrent with a total bilirubin of ≥2xULN and increased by 1xULN or >3x ULN (whichever is smaller).

In cases of suspected Drug-Induced Liver Injury (DILI) the following tests should be performed: Creatine kinase, indirect bilirubin, direct bilirubin, gamma-glutamyl transferase (GGT) and acetaminophen level.

l Investigator should assess the patient's compliance to contraceptive measures and perform a test if required. Female patients of child-bearing potential only. A serum or urine pregnancy test is to be performed at screening, pre dose on cycle 1 day 1 and at discontinuation. In the event of suspected pregnancy during the study, the test should be repeated and, if positive, the patient discontinued from study treatment immediately.

m CT or MRI scan of head, chest and abdomen to be performed at screening. CT or MRI scans of chest and abdomen to be performed until discontinuation. Following screening, the first tumour assessment should be performed 6 weeks after cycle 1 day 1, then every 6 weeks thereafter for the first year, later reducing to every 12 weeks. Scans should be performed + 7 days (except 1st scan + 7 days only). If brain metastases are identified at Screening or if clinically indicated, head scanning should also be performed throughout treatment at the same time points. **The imaging modality must be used consistently throughout the course of the trial for each patient.**

◊ Tumour assessments will be performed in follow up for patients who discontinue treatment for reasons other than Progressive Disease (e.g. toxicity). These scans should continue to be performed on a 6-weekly basis for the first year relative to the start date of treatment, then every 12 weeks until disease progression or the patient starts a new anti-cancer therapy (unless the patient withdraws consent to do so). Scans should be of the chest and abdomen, and only include the head where brain metastases are identified at screening, or if clinically indicated. All scans to be reported using RECIST 1.1.

n Cycle 1 day 1: Treatment must commence within 7 days of trial registration.

o Smoking status data will be collected through questions and CO monitoring at **pre-dose** cycle 1 day 1, cycle 4 day 1 then every 9 weeks (Day 1 of every 3rd cycle) and at discontinuation. After 12 months of treatment, smoking status collection may be reduced to 12 weekly in line with adjusted visit timing (see footnote 't').

p A whole blood germline DNA sample is to be collected **pre-dose** on Cycle 1 day 1. If the sample is not collected at this timepoint, it should be collected at the next visit. Refer to the Laboratory Manual for sample processing guidelines.

q ctDNA samples to be collected at **pre-dose** cycle 1 day 1, cycle 4 day 1 then every 9 weeks (Day 1 of every 3rd cycle) and at discontinuation. Samples can be taken up to 2 days earlier than the actual visit date (where indicated). After 12 months of treatment, ctDNA sample collection may be reduced to 12 weekly in line with adjusted visit timing (see footnote 't'). Refer to the Laboratory Manual for sample processing instructions.

$ ctDNA samples will be collected in follow up for patients who discontinue treatment for reasons other than Progressive Disease (e.g. toxicity). These samples should be performed at the same visit as follow up CT or MRI scans until disease progression or the patient starts a new anti-cancer therapy (unless the patient withdraws consent to do so). Samples should be collected on a 6-weekly basis for the first year relative to the start date of treatment then every 12 weeks.

r An optional fresh metastatic/recurrent tumour biopsy sample should be collected (if patient consents) post–registration (pre-treatment) and at the end of treatment visit for patients who discontinue treatment for reasons other than disease progression (origin from either the primary tumour of site of metastasis). An optional pre-treatment biopsy should not be performed in cases where the patient has already had a mandatory biopsy for molecular testing (Note - a mandatory repeat SMP2 biopsy will be performed if the patient has had targeted therapy e.g. ALK inhibitor). The discontinuation biopsy must be performed prior to commencing further anti-cancer therapy. A post-treatment biopsy will only be requested from patients with an objective response or stabilisation of disease (PR or CR), or 6 months on treatment with evidence of stabilisation (SD) for patients

who have previously progressed. The tumour tissue will be used to determine possible mechanisms of resistance to study treatment. Refer to the Laboratory Manual for sample processing instructions.

s Survival status will be collected every 12 weeks (± 7 days) post-permanent discontinuation of Crizotinib until death.

t Once a patient has completed 12 months of treatment (approximately 17 cycles), visits may be reduced to 12 weekly at the discretion of the Investigator.

u 28 day follow up visit should be carried out 28 days (+ 7 days) post-permanent discontinuation of Crizotinib.

25.3.3 Toxicity Profile

25.3.3.1 Patient Exposure

As of the database snapshot date of the IB of 08 July 2016, safety data were available for a total of 2635 patients treated with crizotinib on Pfizer-sponsored studies.

25.3.3.2 Treatment-Related Adverse Events – All Studies

The primary Cycle 1 DLT observed in the Phase 1 study of single-agent crizotinib was fatigue, with a MTD of crizotinib on a BID schedule determined to be 250 mg.

A total of 2129 (95.6%) of the 2227 patients with advanced NSCLC who received at least 1 dose of crizotinib 250 mg BID in the single-agent studies (Studies A8081001, A8081005, A8081007, A8081012, A8081014, A8081029 and A8081063) had treatment-related AEs of which the most commonly reported (in ≥20% of patients) were VISION DISORDER (57.6%), Diarrhoea (47.6%), Nausea (46.8%), Vomiting (41.9%), OEDEMA (36.7%), ELEVATED TRANSAMINASES (34.4%), Constipation (30.8%), and NEUTROPENIA (22.4%) (event terms written in ALL CAPITALS represent CLUSTERED TERMS which included multiple Preferred Terms).

A total of 850 (38.2%) patients had treatment-related AEs of Grade 3, 4 or 5 in severity. The maximum severity was Grade 3 treatment-related in 31.8% of patients, Grade 4 treatment-related in 5.2% of patients, and Grade 5 treatment-related in 1.2% of patients. The most common Grade 3 treatment-related AEs (reported in ≥2% patients) were NEUTROPENIA (10.4%), ELEVATED TRANSAMINASES (7.5%), Hypophosphataemia (2.6%), LEUKOPENIA (2.4%), LYMPHOPENIA (2.3%), and Fatigue (2.0%). The most common treatment-related Grade 4 AEs (reported in ≥1% of patients) were NEUTROPENIA (2.1%) and ELEVATED TRANSAMINASES (1.3%). The most common Grade 5 treatment-related AEs (reported in ≥0.1% of patients) were INTERSTITIAL LUNG DISEASE (0.4%), Death (0.2%), Pneumonia, HEPATOTOXICITY, and Lung infection (0.1% each).

Most deaths while on crizotinib treatment were due to progression of the underlying disease. There were 39 treatment-related deaths reported during single-agent crizotinib treatment. Twelve were cases of Death (of unknown cause), Sudden death, or Death not otherwise specified (NOS), and were considered treatment-related because there were no data indicating a different cause of death. Other fatal events reported in more than 1 patient were Pneumonitis (8 patients), Pneumonia and Interstitial lung disease (4 patients each), Hepatic failure (2 patients), Lung infection (2 patients), and Dyspnoea (2 patients). There have not been any treatment-related deaths on crizotinib in combination with an EGFR TKI, a VEGFR TKI nor among patients in the combination studies with immunotherapy. In the expanded access protocol, 1 patient had an event with fatal outcome which was reported to be due to neoplasm progression and was not treatment-related.

Out of the 2227 patients with advanced NSCLC treated with single-agent crizotinib 250 mg BID, a total of 116 (5.2%) permanently discontinued crizotinib associated with treatment-related AEs. The most common treatment-related AEs on single-agent crizotinib that were associated with permanent treatment discontinuation were INTERSTITIAL LUNG DISEASE (1.4%), ELEVATED TRANSAMINASES (0.8%) and HEPATOTOXICITY (0.5%).

25.3.3.3 Specific Adverse Events of Note

25.3.3.3.1 Cardiac Failure

Cases of cardiac failure have been reported, some of which were severe and fatal. Across clinical studies in patients with ALK-positive NSCLC (n = 1669), a total of 19 (1.1%) patients treated with crizotinib had any grade cardiac failure, 8 (0.5%) patients had Grade 3 or 4, and 3 (0.2%) patients had fatal outcome.

Patients with or without pre-existing cardiac disorders, receiving crizotinib, should be monitored for signs and symptoms of heart failure (dyspnoea, oedema, rapid weight gain from fluid retention). If symptoms of cardiac failure are observed, appropriate measures such as dosing interruption, dose reduction, or discontinuation should be considered.

25.3.3.3.2 Gastrointestinal Perforation

Cases of gastrointestinal perforation have been reported, some of which were fatal. There were other possible contributing causes in many of the cases.

25.3.3.3.3 Visual Loss

Cases of severe visual loss have been reported. There were other alternate aetiologies or contributing causes in most cases.

25.3.3.4 Special warnings and precautions for use

25.3.3.4.1 Effects on the Liver

Drug-induced hepatotoxicity with fatal outcome has occurred. These cases have occurred during crizotinib treatment in less than 1% of patients in clinical trials. Most of these cases occurred within the first 2 months after the start of crizotinib treatment.

Concurrent elevations in ALT greater than 3× ULN and total bilirubin greater than 2× ULN without elevated alkaline phosphatase have been observed in less than 1% of patients in clinical trials. Grade 3 and 4 elevations were generally asymptomatic and reversible upon dosing interruption. Patients usually resumed treatment at a lower dose without recurrence; however, a small number of patients required permanent discontinuation from treatment.

Low-grade increases in transaminases were commonly observed, particularly within the first 8 weeks of crizotinib treatment. Liver function tests including ALT and total bilirubin should be monitored every 2 weeks during the first 2 months of treatment, then once a month and as clinically indicated, with more frequent repeat testing for Grades 2, 3, or 4 elevations, or in case of development of signs or symptoms consistent with hepatotoxicity or hepatic failure (e.g., fatigue, weakness, anorexia, nausea, vomiting, right upper quadrant abdominal pain, jaundice, dark urine, and in rare cases, fever, and rash). In such cases, laboratory assessment of transaminases and total bilirubin should be performed, particularly if symptoms are reported in the 3-6-week period after starting crizotinib, when some of the most commonly experienced initial gastrointestinal symptoms (e.g., nausea, vomiting, and diarrhoea) may have abated. Abnormal values in AST and/or ALT concurrent with abnormal elevations in total bilirubin that meet the criteria outlined below in the absence of other causes of liver injury are considered possible cases of drug-induced liver injury (Hy's Law cases) and should always be considered important medical events. The threshold of laboratory abnormalities for a potential case of drug-induced liver injury depends on the patient's individual baseline values and underlying conditions. Patients who present with the following laboratory abnormalities should be evaluated further to definitively determine the aetiology of the abnormal laboratory values:

- Patients with AST or ALT and total bilirubin baseline values within the normal range

who subsequently present with AST or ALT ≥3 times the upper limit of normal (× ULN) concurrent with a total bilirubin ≥2 × ULN with no evidence of haemolysis and an alkaline phosphatase ≥2 × ULN or not available.

- For patients with pre-existing ALT, AST, or total bilirubin values above the upper limit of normal, the following threshold values should be used in the definition mentioned above:

    - With pre-existing AST or ALT baseline values above the normal range: AST or ALT ≥2 times the baseline values and ≥3 × ULN, or ≥8 × ULN (whichever is smaller).
    - With pre-existing values of total bilirubin above the normal range: Total bilirubin increased by 1 × ULN over baseline or ≥3 × ULN (whichever is smaller).

For patients who develop transaminase elevations, the Dose Modification schedule should be followed. All suspected cases of drug-induced liver injury must be expeditiously reported as serious adverse events, and the suspected agent should be discontinued immediately. The patient should return to the investigational site and be evaluated as soon as possible, preferably within 48 hours from awareness of the abnormal results. This evaluation should include laboratory tests, detailed history, and physical assessment; and for oncology studies, the possibility of hepatic neoplasia (primary or secondary) should be considered. In addition to repeating AST and ALT, laboratory tests should include albumin, creatine kinase, total bilirubin, direct and indirect bilirubin, gammaglutamyl transferase (GGT), international normalised ratio (INR), and alkaline phosphatase.

A detailed history, including relevant information, such as review of ethanol, acetaminophen (paracetamol), recreational drug and supplement consumption, family history, sexual history, travel history, history of contact with a jaundiced patient, surgery, blood transfusion, history of liver or allergic disease, and work exposure, should be collected. An acetaminophen (paracetamol) level should be obtained in all patients with suspected drug-induced liver injury at the time of presentation regardless of their history of acetaminophen (paracetamol) use. Further, testing for acute hepatitis A, B, C, or E virus infection, including testing for reactivation of viral infection (HBV), should be considered. Additional serologies (EBV, CMV, HSV, VZV, parvovirus, toxoplasma) should be considered. Liver imaging to rule out biliary obstruction and assess the extent of metastatic liver disease should be considered as well. All cases confirmed on repeat testing as meeting the laboratory criteria defined above, with no other cause for liver function test (LFT) abnormalities yet identified at the time should be considered potential Hy's Law cases irrespective of availability of all the results of the investigations performed to determine aetiology of the abnormal LFTs. Such cases should be reported as SAEs.

25.3.3.4.2 Effects on the Respiratory Tract

Interstitial Lung Disease (Pneumonitis)

Crizotinib has been associated with severe, life-threatening, or fatal treatment-related INTERSTITIAL LUNG DISEASE as described in section 6.2 of the IB. These cases generally occurred within 3 months after initiation of treatment.

Patients should be monitored for pulmonary symptoms indicative of INTERSTITIAL LUNG DISEASE, and NSCLC progression, other pulmonary disease, infection, or radiation effect should be excluded. Other potential causes of interstitial lung disease (ILD)/pneumonitis should be excluded.

Diagnostic measures may include a blood culture, a sputum gram stain and culture, thoracocentesis (with culture of pleural fluid, chemistry analysis, and cytology), bronchoscopy

with bronchoalveolar lavage, lung biopsy, B-natriuretic peptide (to assess for congestive heart failure), as well as testing for mycoplasma, respiratory viruses (RSV, influenza, parainfluenza, adenovirus, human metapneumovirus, etc.), Legionella, and Pneumocystis jiroveci (beta-D-glucan to be considered for Asian patients).

Patients should be permanently discontinued from crizotinib treatment if diagnosed with drug-related INTERSTITIAL LUNG DISEASE.

25.3.3.4.3 Effects on the Cardiovascular System

Crizotinib inhibited hERG potassium currents with an IC_{20} and IC_{50} of 0.3 μM (135 ng/mL) and 1.1 μM (495 ng/mL), respectively. In addition to its effects on the hERG potassium channel, it was also identified as a calcium channel antagonist, suggesting that crizotinib is a mixed ion channel blocker. Decreases in HR, diastolic BP, myocardial contractility and increase in left ventricular end diastolic pressure (LVEDP) were observed in CV study in anesthetized dogs. The increased LVEDP, PR interval prolongation and QRS complex prolongation, and decreased myocardial contractility were likely due to the reduction in HR, though the increase in QRS interval duration could also be related to Nav1.5 sodium channel inhibition. Of note, the actual myocardial contractility values in the treated animals were similar to predose values. The effect on BP could relate to other off-target (secondary pharmacology) activity of crizotinib.

QTc Prolongation
The QTc prolongation potential of crizotinib was assessed in patients who received crizotinib 250 mg twice daily. ECGs in triplicate were collected following a single dose and at steady state to evaluate the effect of crizotinib on QT intervals.

QTc prolongation has been observed. Crizotinib should be avoided in patients with congenital long QT syndrome. In patients with congestive heart failure, bradyarrhythmias, electrolyte abnormalities, or who are taking medications that are known to prolong the QT interval, periodic monitoring with ECGs and electrolytes should be considered. Permanently discontinue crizotinib in patients who develop Grade 4 QTc prolongation. Withhold crizotinib in patients who develop Grade 3 QTc prolongation until recovery to less than or equal to Grade 1, then resume crizotinib at 200 mg twice daily. In case of recurrence of Grade 3 QTc prolongation, withhold crizotinib until recovery to Grade <1, then resume crizotinib at 250 mg once daily. Permanently discontinue crizotinib if Grade 3 QTc prolongation recurs.

Bradycardia
BRADYCARDIA has been reported in patients treated with crizotinib. Most cases were Grade 1 or 2 in severity. Symptomatic bradycardia can occur in patients receiving crizotinib. Syncope can also occur in patients receiving crizotinib.

Avoid using crizotinib in combination with other agents known to cause bradycardia (e.g., beta-blockers, non-dihydropyridine calcium channel blockers, clonidine, and digoxin) to the extent possible. Monitor heart rate and blood pressure regularly. In cases of symptomatic bradycardia (including syncope, dizziness, hypotension) that is not life-threatening, hold crizotinib until recovery to asymptomatic bradycardia or to a heart rate of 60 bpm or above, re-evaluate the use of concomitant medications, and adjust the dose of crizotinib. Permanently discontinue for life-threatening bradycardia due to crizotinib; however, if associated with concomitant medications known to cause bradycardia or hypotension, hold crizotinib until recovery to asymptomatic bradycardia or to a heart rate of 60 bpm or above, and if concomitant medications can be adjusted or discontinued, restart crizotinib at 250 mg once daily with frequent monitoring.

25.3.3.4.4 Effects on Vision

Results from a rat electroretinography (ERG) study suggest that visual effects observed clinically may be related to a direct effect on retinal function. The observed effect during dark adaptation in the rat ERG study was transient, indicating that the rate but not the ability to achieve dark adaptation was affected. Ocular toxicity was not observed from general toxicity studies of up to 3 months duration, which suggests that the effect on retinal function is not associated with a morphological insult.

VISION DISORDER was a commonly reported adverse event in patients with advanced NSCLC treated with single-agent crizotinib. Nearly all of these events were mild in severity. In those patients who had follow-up ophthalmological examinations, there were no reports of any clinically meaningful changes in visual acuity, slit lamp biomicroscopy, or fundoscopy attributed to crizotinib. There were 4 cases in clinical trials of Grade 4 severe visual loss, but underlying medical conditions may have contributed to the occurrence of these events.

Based on the Visual Symptom Assessment Questionnaire (VSAQ-ALK), patients treated with crizotinib in Study 1007 and Study 1014 reported a higher incidence of visual disturbances compared to patients treated with chemotherapy. The onset of vision disorder generally occurred during the first week of drug administration. The majority of patients in the crizotinib arms in Study 1007 and Study 1014 (>50%) reported visual disturbances, which occurred at a frequency of 4 to 7 days each week, lasted up to 1 minute, and had mild or no impact (scores 0 to 3 out of a maximum score of 10) on daily activities as captured by the VSAQ-ALK questionnaire.

Monitor patients for visual effects while on crizotinib, and withhold crizotinib in patients who develop Grade 3 vision disorder until recovery to less than or equal to Grade 1, then resume crizotinib after dose reduction by 1 dose level. Ophthalmologic examination including visual acuity and slit lamp examination should be performed at screening and at the time a visual event is reported, and again if vision disorder persists or worsens in severity.

Permanently discontinue crizotinib in patients who develop Grade 4 vision disorder or vision loss. Any patients with severe visual loss should have an ophthalmological evaluation.

25.3.3.4.5 Effects on the Gastrointestinal Tract

Nausea, diarrhoea, vomiting and constipation

Nausea, vomiting, diarrhoea, and constipation were the most commonly reported gastrointestinal events, each of which was primarily Grade 1 or Grade 2 in severity. The prevalence of these gastrointestinal effects decreased after 3-4 weeks of treatment. Dyspepsia was reported less frequently.

Monitor patients for treatment-related gastrointestinal events, with dosing interruption, dose reduction, and/or standard medical therapy for Grade 3 or 4 toxicity, as appropriate.

Supportive care for gastrointestinal events should include standard antiemetic, antidiarrheal, laxative, or antacid medications. The use of prophylactic antiemetics should be considered.

Gastrointestinal perforation

In clinical studies with crizotinib, events of gastrointestinal perforations were reported. There were reports of fatal cases of gastrointestinal perforation during post-marketing use of XALKORI (see Section 25.3.3.3).

Crizotinib should be used with caution in patients at risk for gastrointestinal perforation (e.g., history of diverticulitis, metastases to the gastrointestinal tract, concomitant use of medications with a recognized risk of gastrointestinal perforation).

Crizotinib should be discontinued in patients who develop gastrointestinal perforation. Patients should be informed of the first signs of gastrointestinal perforations and be advised to consult rapidly in case of occurrence.

25.3.3.4.6 Effects on the Bone Marrow

Most hematologic abnormalities were mild to moderate in severity and were not associated with AEs. Lymphopenia and neutropenia were the most common hematologic Grade 3 or Grade 4 abnormalities. There were 7 cases of febrile neutropenia reported as SAEs in crizotinib-treated patients in single-agent crizotinib studies, 6 of which were considered to be treatment related.

Monitor complete blood counts including differential white blood cell counts monthly and as clinically indicated, with more frequent repeat testing if Grade 3 or 4 abnormalities are observed, or if fever or infection occurs (see section 25.3.4.4). Temporary suspension of crizotinib is recommended for Grade 3 or 4 haematologic toxicity; treatment may be resumed after recovery to Grade ≤2 with or without dose reduction. The use of hematopoietic growth factors is at the discretion of the investigator. Patients with neutropenic fever or infection should be treated promptly and may receive therapeutic colony-stimulating factors, if appropriate.

25.3.3.4.7 Effects on the Kidney

Blood Creatinine Increased (laboratory test abnormality)
Increased blood creatinine levels have been reported in patients with advanced NSCLC treated with crizotinib, but there has not been any clear evidence of a clinically relevant effect of crizotinib on renal function. Data from the 4 clinical studies indicated that the use of crizotinib resulted in an increase in serum creatinine and a decline in estimated Glomerular Filtration rate which was first observed at 2 weeks and remained relatively constant from 12 weeks of treatment through the remainder of the time points examined. There did not appear to be any cumulative toxicity with continued crizotinib treatment. The frequency of Blood creatinine increased was 8.0% in the 4 clinical studies. Renal function should continue to be monitored in patients treated with crizotinib.

Renal Cyst
The development of complex renal cysts has been reported in some patients with NSCLC treated with crizotinib. These cysts are often asymptomatic, and have developed from 1 week to several months after starting crizotinib. The precise nature and significance of these cysts is unclear; however, while no evidence of malignancy has been found based on aspiration of cyst fluid and biopsy in the reported cases, complex renal cysts may be associated with renal malignancy, and thus consultation with urologist or suitable alternate medical expert is recommended. Neither renal impairment nor clinically relevant proteinuria has been observed in these cases. While these renal cysts do not appear to affect renal function, they can become rather large, and in some cases, are associated with extra renal invasion into contiguous anatomical structures, such as the peri-renal space, the abdominal wall, the peritoneal cavity, or the psoas muscle, and while draining procedures have sometimes been employed in such cases, the optimal management is not known, and therefore, the decision to perform drainage of these cysts should be made on a case-by-case basis.

Monitoring with appropriate imaging should be performed per protocol (e.g., every 6 or 8 weeks with contrast-enhanced CT scanning or magnetic resonance imaging assuring full visualization of the kidneys). In addition, dipstick urinalysis should be performed at the time renal cysts are

diagnosed and on Day 1 of each cycle thereafter; in Korea, dipstick urinalysis should be performed in all patients at screening and on Day 1 of each cycle thereafter. Urine reflex microscopy is required whenever urine dipstick is positive for blood or protein.

25.3.3.4.8 Effects on the Nervous System

NEUROPATHY was experienced by NSCLC patients treated with single-agent crizotinib, and was primarily Grade 1 or 2 in severity. It is unclear to what extent prior platinum therapy may have contributed to this risk, as the large majority of patients on these studies had previously received platinum-based regimens.

Monitor for treatment-related neuropathy, with dosing interruption, dose reduction, and/or standard medical therapy for Grade 3 or 4 toxicity as appropriate

25.3.3.4.9 Oedema

EDEMA, including peripheral oedema and localized oedema, has been reported in patients with advanced NSCLC treated with single-agent crizotinib. Nearly all of these cases were Grade 1 or 2 in severity.

Monitor for treatment-related oedema, with dosing interruption, dose reduction, and/or standard medical therapy for Grade 3 or 4 toxicity as appropriate.

25.3.3.4.10 Fatigue

Fatigue has been reported in patients with advanced NSCLC treated with single-agent crizotinib. The majority of these cases were Grade 1 or 2 in severity.

Monitor for treatment-related fatigue, with dosing interruption, dose reduction, and/or standard medical therapy for Grade 3 or 4 toxicity as appropriate.

25.3.3.4.11 Rash

Rash has been reported in patients with advanced NSCLC treated with single-agent crizotinib, and was primarily Grade 1 or 2 in severity. As described in Section 5.3 of the IB, crizotinib was identified with probable phototoxicity potential in vitro, yet few cases of photosensitivity reaction have been reported in patients with advanced NSCLC treated with single-agent crizotinib.

Monitor for treatment-related rash, with dosing interruption, dose reduction, and/or standard medical therapy for Grade 3 or 4 toxicity, as appropriate. Patients treated with crizotinib should avoid sunbathing, prolonged unprotected sun exposure, or tanning for the duration of the study period.

25.3.3.4.12 Overdose

Treatment of overdose with crizotinib should consist of general supportive measures. There is no antidote for crizotinib.

25.3.3.4.13 Effects on Ability to Drive and Use Machines

No studies on the effect of crizotinib on the ability to drive and use machines have been performed. However, advise patients to exercise caution when driving or operating machinery due to the risk of developing vision disorder, dizziness, or fatigue while taking crizotinib.

25.3.4 Dosing Modifications & Toxicity Management

25.3.4.1 Treatment delays and discontinuation

If a treatment delay results from a decline in haematological parameters, the frequency of blood count assessments should be adjusted as clinically indicated.

If the re-treatment parameters (see criteria below) are met within 3 weeks of treatment interruption, crizotinib may be resumed.

If these parameters have not been met after 3 weeks of treatment interruption (including the scheduled 1 week off treatment), permanent discontinuation of trial treatment should be considered in consultation with the National Lung Matrix Trial Office.

In the event of a treatment interruption for reasons other than treatment related toxicity (e.g., non-cancer related surgery) lasting >3 weeks, treatment resumption will be decided on consultation with the National Lung Matrix Trial Office. It is advised that treatment delays should not exceed 6 weeks (2 cycles).

Patients who become pregnant are not allowed to continue treatment.

25.3.4.2 Criteria for recommencement of treatment

The following parameters should be met prior to recommencement of treatment: 1) at the start of every new cycle and; 2) following a treatment interruption for treatment related toxicity:

- Platelet count ≥ 50,000/mm^3;
- ANC ≥ 1000/mm^3 and no fever;
- ≥Grade 3 treatment-related non-haematological AEs considered related to crizotinib recovered to ≤grade 1 or baseline (also see Table 39: Dose modifications for non-haematological toxicity);

- QTcF ≤480 msec and potential reversible causes (e.g. electrolyte imbalance, concomitant medications known to prolong QTcF) corrected. If QTcF remains above 480msec, a cardiologist should be consulted and ECG should be monitored more frequently until QTcF ≤480 msec (see Table 39: Dose modifications for non-haematological toxicity) for guidance on grade 3 toxicities that recover to ≤grade 1).

25.3.4.3 Dose reductions

Dosing interruption and/or dose reduction may be required based on individual safety and tolerability.

In 1722 patients treated with crizotinib with either ALK-positive or ROS1-positive NSCLC across clinical studies, the most frequent adverse reactions (≥3%) associated with dosing interruptions were neutropenia, elevated transaminases, vomiting, and nausea. The most frequent adverse reactions (≥3%) associated with dose reductions were elevated transaminases and neutropenia.

If dose reduction is necessary, then the dose of crizotinib should be reduced to 200 mg taken twice daily, then to 250 mg taken orally once daily if further reduction is necessary.

Table 37: Crizotinib - Available dose levels

Dose level	Crizotinib dose
Starting dose	250mg BD
-1	200mg BD
-2	250mg OD
N/A	Discontinue Trial Treatment

25.3.4.4 Evaluation, Management and Treatment of Toxicities

25.3.4.4.1 Hepatic Impairment

As crizotinib is extensively metabolized in the liver, hepatic impairment is likely to increase plasma crizotinib concentrations. A phase 1 study to evaluate the effect of hepatic impairment on the pharmacokinetics of crizotinib in advanced cancer patients is currently ongoing. Clinical studies A8081001, A8081005 and A8081007 excluded patients with ALT or AST >2.5 × ULN or, if due to underlying malignancy, >5.0 × ULN or with total bilirubin >1.5 × ULN. The popPK analysis using the data from these studies indicated that baseline total bilirubin or AST levels did not have a clinically meaningful effect on the pharmacokinetics of crizotinib. Treatment with crizotinib should be used with caution in patients with hepatic impairment.

Starting dose adjustment is not required for patients with mild hepatic impairment. For patients with moderate hepatic impairment, the mean crizotinib AUCdaily and Cmax at steady state were about 150% and 144%, respectively, to that in patients with normal hepatic function (both groups treated with crizotinib at a dose of 200 mg bi-daily) and a starting dose adjustment to 200 mg bi-daily is recommended for these patients. For patients with severe hepatic impairment: the mean AUCdaily and Cmax at steady state for patients receiving crizotinib 250 mg QD was about 65% and 73%, respectively, to that in patients with normal hepatic function receiving crizotinib 250 mg bi-daily and a starting dose adjustment to 250 mg QD is recommended for these patients.

25.3.4.4.2 Renal Impairment

Patients with mild (60≤ CLcr <90 mL/min) and moderate (30≤ CLcr <60 mL/min) renal impairment were enrolled in single-arm Studies A8081001 and A8081005. The effect of renal function, as measured by baseline CLcr on observed crizotinib steady-state trough concentrations ($C_{trough, ss}$) was evaluated. In Study A8081001, the adjusted geometric mean of plasma $C_{trough, ss}$ in mild (N = 35) and moderate (N = 8) renal impairment patients were 5.1% and 11% higher, respectively, than those in patients with normal renal function. In Study A8081005, the adjusted geometric mean $C_{trough, ss}$ of crizotinib in mild (N = 191) and moderate (N = 65) renal impairment groups were 9.1% and 15% higher, respectively, than those in patients with normal renal function. In addition, the popPK analysis using data from Studies A8081001, A8081005 and A8081007 indicated that CLcr did not have a clinically meaningful effect on the pharmacokinetics of crizotinib PK. Due to the small size of the percentage increases in crizotinib exposure (5%-15%), no starting dose adjustments are recommended for patients with mild or moderate renal impairment.

After a single 250 mg dose in patients with severe renal impairment (CLcr <30 mL/min) not requiring peritoneal dialysis or haemodialysis, crizotinib AUC and C_{max} increased by 79% and 34% respectively, compared to those with normal renal function. An adjustment of the dose of crizotinib to 250 mg taken orally once daily is recommended when administering crizotinib to patients with severe renal impairment not requiring peritoneal dialysis or haemodialysis.

25.3.4.4.3 Dose Reduction Guidelines for Haematological and Non-Haematological Toxicities

Dose reduction guidelines for haematological and non-haematological toxicities are provided in Table 38 and Table 39.

Patients must discontinue crizotinib if they develop gastrointestinal perforation.

Table 38: Dose modifications for haematological toxicity[a,b]

CTCAE[c] grade	Dose Modification
Grade 3	Withhold until recovery to Grade ≤ 2, then resume at the same dose schedule
Grade 4	Withhold until recovery to Grade ≤ 2, then resume at 200 mg twice daily[d]

a) Except lymphopaenia (unless associated with clinical events, e.g., opportunistic infections).
b) For patients who develop neutropaenia and leukopenia, see also Sections 25.3.4.4 and 25.3.3.4
c) National Cancer Institute (NCI) Common Terminology Criteria for Adverse Events
d) In case of recurrence, dosing should be withheld until recovery to Grade ≤ 2, then dosing should be resumed at 250 mg once daily. XALKORI must be permanently discontinued in case of further Grade 4 recurrence.

Table 39: Dose modifications for non-haematological toxicity

CTCAE[a] Grade	Dose Modification
Grade 3 or 4 alanine aminotransferase (ALT) or<br>aspartate aminotransferase (AST) elevation with Grade ≤ 1 total bilirubin	Withhold until recovery to Grade ≤ 1 or baseline, then<br>resume at 250 mg once daily and escalate to 200 mg twice<br>daily if clinically tolerated[b]
Grade 2, 3 or 4 ALT or AST<br>elevation with concurrent Grade 2, 3 or 4 total bilirubin elevation (in the absence of cholestasis or haemolysis)	Permanently discontinue
Any Grade interstitial lung disease (ILD)/pneumonitis	Withhold if ILD/pneumonitis is suspected, and permanently<br>discontinue if treatment-related ILD/pneumonitis is<br>diagnosed[c]
Grade 3 QTc prolongation	Withhold until recovery to Grade ≤ 1, check and if necessary correct electrolytes, then resume at 200 mg twice daily[b]
Grade 4 QTc prolongation	Permanently discontinue
Grade 2, 3 Bradycardia[c,d]<br>Symptomatic, may be severe and medically significant, medical intervention indicated	Withhold until recovery to Grade ≤ 1 or to heart rate 60 or above.<br>Evaluate concomitant medicinal products known to cause bradycardia, as well as anti-hypertensive medications.

CTCAE[a] Grade	Dose Modification
	If contributing concomitant medication is identified and discontinued, or its dose is adjusted, resume at previous dose upon recovery to Grade ≤ 1 or to heart rate 60 or above<br>If no contributing concomitant medicinal product is identified, or if contributing concomitant medicinal product are not discontinued or dose modified, resume at reduced dose upon recovery to Grade ≤1 or to heart rate 60 or above.
Grade 4 Bradycardia[c,d,e]<br>Life-threatening consequences, urgent intervention indicated	Permanently discontinue if no contributing concomitant<br>medication is identified.<br>If contributing concomitant medication is identified and discontinued, or its dose is adjusted, resume at 250 mg once daily upon recovery to Grade ≤ 1 or to heart rate 60 or above, with frequent monitoring.
Grade 4 Ocular Disorder (Visual Loss)	Discontinue during evaluation of severe vision loss

a. NCI Common Terminology Criteria for Adverse Events
b. Crizotinib must be permanently discontinued in case of further Grade ≥3 recurrence. See Sections 25.3.4.4 and 25.3.3.4
c. See Sections 25.3.4.4 and 25.3.3.4
d. Heart rate less than 60 beats per minute (bpm).
e. Permanently discontinue for recurrence.

25.3.4.4.4 Vision Disorder

Table 40: Dose modifications for vision disorder

CTCAE grade	Dose Modification
Grade 3	Withhold until recovery to Grade ≤1 then resume after reduction by 1 dose level. Ophthalmologic examination including visual acuity and slit lamp examination should be performed, and again if disorder persists or worsens in severity.
Grade 4	Permanently discontinue

25.4 Reference List

- Bergethon K, Shaw AT, Ou SH *et al.* (2012) ROS1 rearrangements define a unique molecular class of lung cancers. *J Clin Oncol* **30(8):**863-70.
- Brambilla E, Travis WD, Colby TV *et al.* (2001)The new World Health Organization classification of lung tumours. *Eur Respir J* **15:**1059-68.
- Camidge DR, Ou S-HI, Shapiro GI *et al.* (2014). Efficacy and safety of crizotinib in patients with advanced MET-amplified non-small cell lung cancer (NSCLC). J Clin Oncol, **32:5s:**(suppl; abstr 8001).
- Cappuzzo F, Marchetti A, Skokan M *et al.* (2009). Increased MET gene copy number negatively affects survival of surgically resected non-small-cell lung cancer patients.
- *J Clin Oncol* **27(10):**1667-74.
- Chen YT, Chang JW, Liu HP *et al.* (2011). Clinical implications of high MET gene dosage in non-small cell lung cancer patients without previous tyrosine kinase inhibitor treatment. *J Thorac* Oncol **6(12):**2027-35.
- Frampton GM, Ali SM, Rosenweig M *et al.* (2015). Activation of MET via Diverse Exon 14 Splicing Alterations Occurs in Multiple Tumor Types and Confers Clinical Sensitivity to MET Inhibitors. *Cancer Disc* doi: 10.1158/2159-8290.CD-15-0285 [Epub ahead of print].
- Go H, Jeon YK, Park HJ *et al.* (2010) High MET gene copy number leads to shorter survival in patients with non-small cell lung cancer. *J Thorac Oncol* **5(3):**305-13.
- Jenkins RW, Oxnard GR, Elkin S *et al.* (2015). Response to Crizotinib in a Patient With Lung Adenocarcinoma Harboring a MET Splice Site Mutation. *Clin Lung Cancer* S1525-7304(15)00053-4. doi: 10.1016/j.cllc.2015.01.009. [Epub ahead of print].
- Kong-Beltram M, Seshagiri S, Zha J *et al.* (2006). Somatic mutations lead to an oncogenic deletion of met in lung cancer. *Cancer Res*, **66:** 283-9
- Liu, X, Jia Y, Shen Y *et al.* 2015). Detection of frequent MET Exon 14 skipping events in pulmonary sarcomatoid carcinoma and response to targeted inhibition. *J Clin Oncol* **33** suppl; abstr 8020
- Ma PC, Kijima T, Maulik G *et al.* (2003). c-MET mutational analysis in small cell lung cancer: novel juxtamembrane domain mutations regulating cytoskeletal functions. *Cancer Res.* **63(19):**6272-81.
- Ma PC, Jagadeeswaran R, Jagadeesh S *et al.* (2005). Functional expression and mutations of c-Met and its therapeutic inhibition with SU11274 and small interfering RNA in non-small cell lung cancer. *Cancer Res.* **65(4):**1479-88.
- Mendenhall MA & Goldman JW (2015). MET-Mutated NSCLC with Major Response to Crizotinib. *J Thorac Oncol.* **10(5):**e33-4. doi: 10.1097/JTO.0000000000000491.
- Onozato R, Kosaka T, Kuwano H *et al.* (2009). Activation of MET by gene amplification or by splice mutations deleting the juxtamembrane domain in primary resected lung cancers. *J Thorac Oncol.* **(1):**5-11. doi: 10.1097/JTO.0b013e3181913e0e.
- Ou SH, Kwak EL, Siwak-Tapp C *et al.* (2011). Activity of crizotinib (PF02341066), a dual mesenchymal-epithelial transition (MET) and anaplastic lymphoma kinase (ALK) inhibitor, in a non-small cell lung cancer patient with de novo MET amplification. *J Thorac Oncol* **6(5):**942-6.
- Ou SH, Bang YJ, Camidge DR *et al.* (2013). Efficacy and safety of crizotinib in patients with advanced *ROS1*-rearranged non-small cell lung cancer (NSCLC).*J Clin Oncol* **31**(suppl; abstr 8032).
- Paik PK, Drilon AE, Yu HE *et al.* (2015). Response to crizotinib and cabozantinib in stage IV lung adenocarcinoma patients with mutations that cause MET exon 14 skipping. *J Clin Onc* **33** (suppl; abstr 8021).
- Peschard P, Fournier TM, Lamorte L *et al.* (2001). Mutation of the c-Cbl TKB domain binding site on the Met receptor tyrosine kinase converts it into a transforming protein. *Mol Cell.* **8(5):**995-1004.

- Schwab R, Petak I, Kollar M *et al.* (2014). Major partial response to crizotinib, a dual MET/ALK inhibitor, in a squamous cell lung (SCC) carcinoma patient with de novo c-MET amplification in the absence of ALK rearrangement. *Lung Cancer* **83(1):**109-11.
- Shaw AT, Ou SH, Bang YJ *et al.* (2014). Crizotinib in ROS1-rearranged non-small-cell lung cancer. N Engl J Med **371:**1963–71.
- Seo JS, Ju YS, Lee WC *et al.* (2012). The transcriptional landscape and mutational profile of lung adenocarcinoma. *Genome Res.* **22(11):**2109-19.
- Vieira T, Girard N, Ung M *et al.* (2013). Efficacy of first-line chemotherapy in patients with advanced lung sarcomatoid carcinoma. *J Thorac Oncol.* **8(12):**1574-7.
- Waqar SN, Morgensztern D & Sehn J (2015). MET Mutation Associated with Responsiveness to Crizotinib. *J Thorac Oncol.* **10(5):**e29-31.
- Weingertner N, Meyer N, Voegeli AC *et al.* (2015). Correlation between MET protein expression and MET gene copy number in a Caucasian cohort of non-small cell lung cancers according to the new IASLC/ATS/ERS classification. *Pathology* **47(4):**320-8.

26 ARM E: SELUMETINIB – MEK INHIBITOR IN COMBINATION WITH DOCETAXEL

26.1 Background & Rationale

Lead Investigator: Professor Gary Middleton

26.1.1 Molecular cohorts

Inhibitor: Selumetinib (MEK inhibitor)

Arm	Investigational Medicinal Product	Cohort Number	Histology	Molecular Cohort
E	Selumetinib - MEK Inhibitor & Docetaxel	E1	Squamous cell carcinoma (SCC)	NF1 mutation
		E2	Adenocarcinoma (ADC) or not otherwise specified (NOS) NSCLC	NF1 mutation
		E3	NSCLC	NRAS mutation

Selumetinib (AZD6244, ARRY-142886) is an oral, potent and highly selective allosteric inhibitor of MEK1/2 with a short half-life, currently in development for oncology indications. The intracellular Ras regulated RAF/MEK/ERK protein kinase signal cascade is a key pathway involved in cellular proliferation and there is a strong link between deregulation of this pathway and uncontrolled cell proliferation and survival (Chow *et al.* 2005). Activation of RAF/MEK/ERK signalling pathway is implicated in various cancers, including NSCLC (Khushalani & Adjei 2006). It is anticipated that inhibition of MEK activity should inhibit transduction of the mitogenic and survival signals via RAF/MEK/ERK regardless of the nature of the upstream activation, resulting in an inhibition on tumour proliferation, differentiation and survival.

26.1.2 Pre-Clinical Rationale

The scientific hypothesis for selumetinib is based on inhibition of the RAF/MEK/ERK kinase cascade that when activated plays a pivotal role in cell proliferation and survival and is considered a key pathway for therapeutic intervention in oncology. The RAF/MEK/ERK kinase cascade is activated by Raf binding to activated RAS which itself can be activated either by cell surface receptors, e.g. receptor tyrosine kinases or by mutations in RAS itself. RAF/MEK/ERK signaling can also be activated in cancer by mutation activation of BRAF such as occurs in cutaneous melanoma. Mutated KRAS is constitutively activated compared to wild-type KRAS by virtue of reduced GTPase activity which enables the GTP-bound form of KRAS to recruit the effector Raf proteins which then activate the MEK/ERK kinase pathway. Therefore it is considered that tumours carrying a KRAS mutation will activate MEK-ERK signaling and be more dependent upon this pathway for sustaining the oncogenic phenotype.

Selumetinib has been studied in various pre-clinical models, including studies of both monotherapy and combination therapy in NSCLC and KRAS mutated cancer models. For example *in vivo* studies in KRAS mutation positive human cancer xenografts have demonstrated the potential to improve the therapeutic efficacy of selumetinib in combination with cytotoxic drugs including docetaxel. *In vitro* studies support the hypothesis that dependence upon MEK-ERK signaling is more likely in cells in which mutational activation of the pathway is observed as a consequence of BRAF or RAS gene mutation.

Selumetinib is not currently approved for use in any clinical indication in adults or paediatrics. Clinical testing of the efficacy of selumetinib has focused on indications linked to the RAS-MAPK pathway, with currently ongoing confirmatory studies in adult patients with locally advanced or metastatic non-small cell lung cancer (NSCLC) whose tumours harbour the KRAS mutation, metastatic uveal melanoma, and differentiated thyroid cancer (DTC).

Additional information on selumetinib should be obtained from the latest edition of the IB.

26.1.2.1 Combination of selumetinib with docetaxel - In Vitro Data

The potential for selumetinib to sensitize cells to the effects of docetaxel in the absence of any effect of selumetinib alone has been tested in a small panel of NSCLC cell lines insensitive to the growth inhibitory effects of selumetinib exposed to varying doses of docetaxel and a fixed dose of selumetinib. In all cases, selumetinib treatment did not significantly alter the responses to docetaxel suggesting that in *in vitro* assays, selumetinib does not act as a true sensitizer to docetaxel, regardless of the cell's response to MEK inhibition.

26.1.3 Clinical Data

26.1.3.1 Combination of selumetinib with docetaxel - In Vivo Data

Selumetinib has been combined with docetaxel using human xenograft tumour models (Holt *et. al.* 2012), showing the combination to be more effective than the respective monotherapies. The only exception was the NCI-H1975 model that carries an activating and gatekeeper mutation in EGFR shows no benefit of the selumetinib/docetaxel combination over docetaxel alone.

Table 41: Selumetinib - *In vivo* Combination Effects

Xenograft Model	Treatment[a]	Result (% tumour growth inhibition, compared to control)
SW620 human colorectal cancer xenograft	control<br>selumetinib 25 mg/kg BD<br>docetaxel 15 mg/kg (once weekly)<br>selumetinib + docetaxel	-<br>74<br>12<br>98
HCT-116 human colorectal cancer xenograft	control<br>selumetinib 25 mg/kg BD<br>docetaxel 15 mg/kg (once weekly)<br>selumetinib + docetaxel	-<br>64.9<br>93.4<br>123.2
A549 human non-small cell lung cancer xenograft	control<br>selumetinib 25 mg/kg OD<br>docetaxel 15 mg/kg (once weekly)<br>selumetinib + docetaxel	-<br>50<br>5.8<br>74.8

Xenograft Model	Treatment[a]	Result (% tumour growth inhibition, compared to control)
L004 KRAS/EGFR wild type lung cancer patient-derived NSCLC xenograft mouse model	control<br>selumetinib 25 mg/kg OD<br>docetaxel 15 mg/kg (once weekly)<br>selumetinib + docetaxel	-<br>40<br>>100<br>>100
NCI-H1975 human non-small cell lung cancer xenograft (mutant EGFR L858R/T790M)	control<br>selumetinib 25 mg/kg BD<br>docetaxel 15 mg/kg (once weekly)<br>selumetinib + docetaxel	-<br>46 *<br>114<br>114

a Selumetinib and docetaxel were administered according to their usual monotherapy schedules.
* Not statistically significant compared to control.

An important recent study described a murine 'co-clinical' trial in which mice genetically engineered to develop NSCLC driven by *KRAS* mutation were treated with either docetaxel or the combination of docetaxel plus selumetinib (Chen *et al.* 2012). These studies confirmed findings in cell line derived xenografts that the addition of selumetinib to docetaxel can result in significantly enhanced tumour regression in mut*KRAS* NSCLC.

In mice genetically engineered to conditionally express KrasG12D in the lung epithelium which developed KRAS driven lung adenocarcinomas docetaxel monotherapy resulted in a 30% partial; response rate and no partial responses were seen with selumetinib monotherapy. The combination provided substantial benefit with response rates of 92%. The combination increased apoptosis and reduced proliferation compared to docetaxel alone. Docetaxel alone had no effect on metabolic activity by fludeoxyglucose (FDG) PET but the combination markedly reduced FDG avidity. Docetaxel had no effect on phosphorylated extracellular-signal-regulated kinase (pERK) levels. Selumetinib reduced but did not eliminate pERK but the combination eliminated ERK activation.

In mice with KRAS-driven lung adenocarcinomas docetaxel stabilised disease for several weeks but the addition of selumetinib caused tumour regressions and significantly prolonged progression free survival. These studies also demonstrated that concomitant STK11/LKB1 mutation conferred resistance to the combination therapy and thus patients with STK11/LKB1 loss will be treated in the vistusertib arm (Arm B).

26.1.3.2 Clinical experience

Selumetinib 75 mg bd with docetaxel (75 mg/m^2 every 3 weeks) has been investigated as second-line treatment for patients with *KRAS* mutation-positive locally advanced or metastatic NSCLC in a randomised double-blind Phase II study (Janne *et al.* 2013) with docetaxel alone as the control arm.

The study demonstrated that the addition of selumetinib to docetaxel provided a marked difference in tumour response rate (37% vs 0%, p=0.00001) and PFS (5.3 months vs 2.1 months; HR 0.58; 80% CI 0.42-0.79; 1-sided p=0.0138) and a numerical improvement in median OS from 5.2 months to 9.4 months, (HR 0.80; 80% CI 0.56-1.14; 1-sided p=0.2069). Post hoc analyses of disease-related symptoms based on the Lung Cancer Subscale (LCS), a commonly used endpoint in NSCLC, also showed that significantly more patients treated with the combination of selumetinib and docetaxel experienced clinically important improvements in LCS compared with those who received placebo and docetaxel (LCS

improvement rates: 44% versus 25%, odds ratio 2.50, 80% CI 1.34 to 4.77). The time to worsening of LCS was also in favour of the combination. Patients who received selumetinib and docetaxel experienced a higher incidence of grade >3 AEs (81.8% vs 66.7%), SAEs (59.1% vs 31.0%), and hospitalisation due to AE (48% vs 19%) than in the placebo in combination with docetaxel group, though there was no increase in mortality due to AEs. The most common AE findings that were more frequently experienced by patients receiving selumetinib in combination with docetaxel were as anticipated based on monotherapy profiles of each agent: low neutrophil count, diarrhoea, infections, nausea, vomiting, peripheral oedema, rash (mainly acneiform) and stomatitis. Patients who received selumetinib in combination with docetaxel also experienced febrile neutropenia more frequently (18% versus 0%) and neutropenia was more severe. There was also a greater amount of low grade “intolerable events” on the experimental arm which compromised selumetinib dosing through dose interruptions.

The data above suggests that the combination of selumetinib and docetaxel induces a high frequency and depth of tumour shrinkage which translates into tumour response as assessed by RECIST methodology and an improvement in PFS when compared to that achieved by docetaxel alone. The tumour shrinkage also translates into a reduction in a patient’s disease related symptom burden.

These effects on PFS and tumour shrinkage occurred despite the increased toxicity profile of the combination compared to that of docetaxel alone. A substantial proportion of patients discontinued the combination of selumetinib with docetaxel because of adverse events therefore it may be hypothesised that a greater degree of clinical benefit and in particular a more enduring effect on overall survival may occur with improved tolerability of the combination. This may be achieved by the administration of primary prophylactic G-CSF, use of current toxicity management guidelines and imparting data from other studies within the selumetinib programme that may assist treating physicians make appropriate dose modifications in the event of toxicity.

This landmark study identified the combination of docetaxel and selumetinib as an effective therapy for the treatment of KRAS driven lung cancer. The clinical evaluation of the combination in other contexts in which Ras is activated besides KRAS mutation seems entirely reasonable.

26.1.3.3 Marketing Experience

Selumetinib is in clinical development and is not yet marketed.

Docetaxel (TAXOTERE) is indicated for the treatment of patients with locally advanced or metastatic non-small cell lung cancer after failure of prior chemotherapy.

Docetaxel (TAXOTERE) in combination with cisplatin is indicated for the treatment of patients with unresectable, locally advances or metastatic non-small cell lung cancer, in patients who have no previously received chemotherapy for this condition.

26.1.4 Cohort definition

26.1.4.1 E1 - Squamous cell lung cancer harbouring NF1 mutations

26.1.4.2 E2 - Adenocarcinoma and NOS NSCLC harbouring NF1 mutations

NF1 is a RAS GAP which hydrolyses GTP to guanosine diphosphate (GDP) thus inactivating RAS. When NF1 function is lost as, for example, with mutation this GTPase function is lost with the effect that RAS remains in its active GTP-bound form with subsequent downstream

pathway activation. In a panel of 19 glioblastoma cell lines only 2 demonstrated sensitivity to single agent: both were NF1 deficient. Single agent MEK inhibition suppressed intracranial growth of these sensitive cells and increased survival whereas there was no *in vivo* benefit of MEK inhibition in *in vitro* resistant cell line models. Four NF-deficient cells were resistant to single agent activity. These cells had significant dual signalling down the PI3K/AKT pathway in addition to activated RAS/RAF/MEK signalling. As noted above not all patients with KRAS mutant lung adenocarcinoma responded to the docetaxel/selumetinib combination: it is tempting to assume that resistant tumours have a prominent dual pathway signalling from activated Ras. We intend to retrospectively analyse response in the NF1 cohorts (and the NRAS cohort) according to the presence of the MEK activation/compensatory resistance signatures which predict response to selumetinib (*Dry et al.* 2010). This test can be performed on a single formalin fixed section (personal communication Paul Smith, AZ).

NF1 was first identified as a tumour suppressor gene (TSG) in lung adenocarcinoma in the pivotal paper of Ding and colleagues (Ding *et al.* 2008). In their analysis of 188 samples they found that the most prominent case for a TSG was NF1 with 16 mutations being identified in 13 patients. In the current iteration of the TCGA data the mutation rate in 392 samples is 11.5 %, clearly a not insignificant cohort. This is also very similar to the 11.8% rate in 178 squamous cell carcinomas in the latest TCGA dataset. In the most recent publication of the lung adenocarcinoma TCGA dataset, NF1 mutations were found to be significantly enriched in oncogene-negative tumours and the consortium nominated NF1 mutations as driver events in oncogene-negative lung adenocarcinoma.

26.1.4.3 E3 - NSCLC harbouring NRAS mutations

A recent publication has comprehensively evaluated the characteristics of lung cancer harbouring NRAS mutations. In a series of 4562 patients analysed there was a 0.7% incidence of NRAS mutations, the majority of which were presence in patients with ADC. Critically in 28 of these tumours there was no other obvious driver mutation. 20/21 of the patients for which a smoking history was available were current or former smokers. Importantly the sensitivity of NRAS mutant lung cancer cell lines was tested against a variety of kinase inhibitors. Five of 6 cell lines were sensitive to single agent selumetinib and to no other kinase inhibitors. These cells lines appeared to be signalling principally via MEK as the use of the PI3K inhibitor GDC0941 had no effect on the NRAS mutant cell lines. These data demonstrate whilst this is a small cohort that it is one that would appear to be a particularly attractive one in which to test the combination of selumetinib and docetaxel.

26.2 Specific Eligibility Criteria

26.2.1 Inclusion Criteria

For inclusion in the study, patients must fulfil all of the following criteria:

- Patients must fulfil all the core eligibility criteria.
- Technology hub result (or locally obtained result from an approved Laboratory if applicable.
- Eastern Cooperative Oncology Group (ECOG) Performance Status ≤1 with no deterioration over the previous 2 weeks (see Appendix 8: Eastern Cooperative Oncology Group Performance Status Criteria).
- For patients with EGFRwt tumours, prior therapy must include platinum-based chemotherapy. For patients with tumours harbouring an EGFR mutation known to be associated with EGFR TKI sensitivity, prior therapy must include an EGFR tyrosine kinase inhibitor. For patients with ALK-positive tumours, prior therapy must include an ALK inhibitor.
- Ability to swallow oral medication.

26.2.2 Exclusion Criteria

Patients must not enter the trial if any of the following exclusion criteria are fulfilled:

- Patients who do not fulfil all the core eligibility criteria.
- Treatment with any of the following:
  - Nitrosoureas, mitomycin, or suramin within 6 weeks prior to the first dose of selumetinib & docetaxel.
  - An investigational drug:
    - Within 28 days prior to the first dose of selumetinib & docetaxel OR
    - Within five half-lives of the compound (whichever is the most appropriate is at the discretion of the local Investigator), AND
  - Prior treatment with a MEK, Ras or Raf inhibitor, any treatment that involves the comparator/combination medications or any docetaxel-containing regimen (prior treatment with paclitaxel is acceptable).
  - Radical radiotherapy within 4 weeks prior to the first dose of selumetinib & docetaxel, or limited field of palliative radiotherapy within 7 days of treatment.
  - Any other chemotherapy, investigational agents or other anti-cancer therapy within 4 weeks prior to the first dose of selumetinib & docetaxel.
- Known severe hypersensitivity to any excipient of selumetinib, comparator or combination medications, or history of allergic reactions attributed to compounds of similar chemical or biologic composition to selumetinib or comparator.
- Cardiac conditions as follows:
  - Uncontrolled hypertension (BP ≥150/95 mmHg despite medical therapy).
  - Acute coronary syndrome within 6 months prior to the first dose of selumetinib & docetaxel.
  - Left ventricular ejection fraction below the LLN or <55% measured by echocardiography or institution's LLN for MUGA.
  - Prior or current cardiomyopathy including but not limited to the following:
    - Known hypertrophic cardiomyopathy.
    - Known arrhythmogenic right ventricular cardiomyopathy.
  - Previous moderate or severe impairment of left ventricular systolic function (LVEF <45% on echocardiography or equivalent MUGA) even if full recovery has occurred.
  - Severe valvular heart disease.
  - Uncontrolled angina (Canadian Cardiovascular Society grade II-IV despite medical therapy) (Appendix 11: Canadian Cardiovascular Society Grading of Angina Pectoris.).
  - Symptomatic heart failure NYHA Class II-IV or severe valvular heart disease (Appendix 10: New York Heart Association Classification – Stages of Heart Failure).
  - Atrial fibrillation with a ventricular rate >100 bpm on ECG at rest.
  - QTcF >450msec or other factors that increase the risk of QT prolongation.
- Hepatic function (in patients **with** liver metastasis).
  - Alanine transferase (ALT) and Aspartate transferase (AST) >5 x ULN.
  - Alanine transferase (ALT) and Aspartate transferase (AST) >3.5 x ULN and <5 x ULN with ALP >6 x ULN.
- Ophthalmological conditions as follows:

- Intra-ocular pressure >21 mmHg, or uncontrolled glaucoma (irrespective of intra-ocular pressure).
- Current or past history of retinal pigment epithelial detachment (RPED)/central serous retinopathy (CSR) or retinal vein occlusion.

26.2.3 Restrictions & Concomitant Medications

Information on any treatment from the date of informed consent until 28 days after the administration of the last treatment dose should be recorded. If medically feasible, patients taking regular medication should be maintained on it throughout the study period. Patient should be advised to inform their treating physicians of all concomitant medications, including prescription medicines, over-the-counter drugs, vitamins, and herbal products.

Nb. These lists are not exhaustive and the absence of a drug from the lists does not imply that its combination with selumetinib & docetaxel is safe.

26.2.3.1 CYP Inhibitors and Inducers

Patients should avoid medications that are known to either induce or inhibit the activity of hepatic microsomalisoenzymes, as this may interfere with the metabolism of selumetinib.

In addition, patients should avoid consuming large amounts of grapefruits, Seville oranges, or any other products that may contain these fruits e.g. grapefruit juice, as these may affect selumetinib metabolism. These products inhibit CYP3A4 metabolism of which selumetinib is a substrate, so it would be expected to increase plasma concentrations if taken concurrently.

Changes to, or addition of, the following medications should be avoided, unless clinically indicated, whilst receiving treatment with selumetinib:

Table 42: Inhibitors of CYP1A2, CYP2C19 or CYP3A4

CYP1A2	CYP2C19	CYP3A4
Fluvoxamine, Ciprofloxacin		Indinavir, Nelfinavir, Ritonavir, Clarithromycin, Itraconazole, Ketoconazole, Nefazodone, Saquinavir, Suboxone, Telithromycin, Aprepitant, Erythromycin, Fluconazole, large quantities of grapefruit and Seville oranges (see Section 26.2.3.4), Verapamil, Diltiazem

Table 43: Inducers of CYP1A2, CYP2C19 or CYP3A4

CYP1A2	CYP2C19	CYP3A4
Methylcholanthrene, Modafinil, Nafcillin, Beta-naphthoflavone, Omeprazole	Carbamazepine, Norethindrone, Prednisolone, Rifampicin	Efavirenz, Nevirapine, Barbiturates, Carbamazepine, Glucocorticoids, Modafinil, Oxcarbazepine, Phenobarbital, Phenytoin,

CYP1A2	CYP2C19	CYP3A4
		Pioglitazone, Rifabutin, Rifampicin, St John's wort, Troglitazone

26.2.3.2 Vitamin E

Selumetinib capsules contain vitamin E in the form of D-α-Tacopheryl polyethylene glycol1000 succinate (TPGS), a water-soluble form of vitamin E which acts as a formulation excipient. The maximum daily dose of vitamin E that a study subject may receive from selumetinib is approximately 261.6mg/day. Therefore patients should not take any supplemental vitamin E. High doses of vitamin E have been reported to cause bleeding and interfere with blood coagulation processes.

26.2.3.3 Coumadin Anticoagulant Medications

Selumetinib should be administered with caution in patients who are also receiving concomitant Coumadin anticoagulant medications, e.g. warfarin. These patients should have their INR monitored/anticoagulant assessments conducted more frequently and the dose of the anticoagulant should be adjusted accordingly.

26.2.3.4 Food Restrictions

Patients should avoid consuming large amounts of grapefruits, Seville oranges, or any other products that may contain these fruits e.g. grapefruit juice, as these may affect selumetinib metabolism.

26.2.3.5 Concomitant Radiotherapy

If a patient requires palliative radiotherapy, consideration should be given to suspending trial treatment based on local practice and following discussion with the National Lung Matrix Trial Office.

26.2.3.6 Other Restrictions

- Preliminary data (study D1532C00086) suggests that subjects of Asian descent may experience a higher exposure of selumetinib than the majority of subjects of non-Asian descent receiving an equivalent dose of selumetinib. It is recommended that Investigators take this information into consideration when dosing patients of Asian descent. If, during the course of the selumetinib development programme the dosing regimen being evaluated in this study is found not to be tolerated in a specific ethnic group, this ethnic group may later be excluded from this study. Investigators will be notified, and the protocol will be amended to reflect such findings. The data so far do not suggest a safety concern in any specific population.
- Patients should not donate blood during the study and for at least 12 weeks after receiving the last dose of the study medications.
- Additional information on selumetinib should be obtained from the latest edition of the IB.
- Treatment with St John's Wort should be avoided.

26.2.3.7 Contraception

26.2.3.7.1 <u>Selumetinib:</u>

As reproductive toxicology data indicate that selumetinib has the potential for adverse effects on embryofoetal development and survival at dose levels that do not induce maternal toxicity in mice, the following restrictions apply:

- Selumetinib should not be administered to pregnant or breast-feeding women and conception while on treatment must be avoided.
- Female patients of child-bearing potential will be required to use effective and reliable methods of contraception for the duration of the study and at least 4 weeks after the last dose of selumetinib.
- Male patients with sexual partners who are pregnant or who could become pregnant (i.e. women of child-bearing potential) should use acceptable, effective and reliable methods of contraception for the duration of the study and for at least 12 weeks after the last dose of selumetinib to avoid pregnancy and/or potential adverse effects on the developing embryo.
- A definition of females of childbearing potential and females of non-childbearing potential and effective and reliable methods of contraception are described in Section 6.3.

26.2.3.7.2 Docetaxel:

Docetaxel can have genotoxic effects. Therefore, men being treated with docetaxel are advised not to father a child during and up to 6 months after treatment and to seek advice on conservation of sperm prior to treatment because of the possibility of irreversible infertility due to therapy with docetaxel. Female patients are also advised to use adequate contraception for at least 6 months post discontinuation of docetaxel. Acceptable methods of contraception are described in Section 6.3.

26.3 Trial Treatment

26.3.1 Investigational Medicinal Products

26.3.1.1 Selumetinib

Selumetinib (AZD6244; ARRY 142886) is a potent, selective, uncompetitive inhibitor of MEK, licensed for development by AstraZeneca Pharmaceuticals from Array BioPharma. Array BioPharma was responsible for the first-into-human study; the remainder of the clinical development programme for oncology indications is the responsibility of AstraZeneca.

Selumetinib will be initially administered at a dose of 75 mg BD on a continuous basis on Day 1 to Day 21 of every 21 day cycle. This is the recommended dose for administration as both monotherapy and in combination with docetaxel as defined in phase I multiple ascending dose studies and subsequent phase II and III studies.

Patients should swallow three selumetinib 25 mg capsules twice daily with water, commencing on day 1 after required day 1 assessments are completed. Capsules should be taken whole and with approximately 240 mL water.

All doses of selumetinib should be taken on an empty stomach (no food or drink other than water for 2 hours prior to dosing and 1 hour after dosing).

The doses should be taken approximately 12 hours apart for example at 08:00 and 20:00 or 09:00 and 21:00 at the same time points each day. On docetaxel dosing days there is no need to alter the time points at which selumetinib is taken. Wherever possible, doses should not be missed. If a patient misses taking a scheduled dose, window ± 2 hours, they should take the next dose at the next scheduled time and the missed dose will not be made up. If a patient

vomits after taking their selumetinib medication, they should not make up for this dose, but should take the next scheduled dose. Patients will be provided with dosing instructions for the study. Any deviations from dosing schedule, dose interruptions, dose reductions and dose adjustments should be recorded. This includes details of vomiting after taking study medication e.g. date & time of vomiting in relation to dosing.

If docetaxel is discontinued for reasons other than disease progression, selumetinib should be continued until disease progression (please note patients who meet RECIST criteria for progressive disease (PD) may be continued on trial treatment if the treatment is tolerable and the Investigator believes it to be of clinical benefit; see Section 9.3), intolerable toxicity or the occurrence of another discontinuation criterion. Likewise, if selumetinib is discontinued during the first 6 cycles for reasons other than progressive disease, docetaxel may continue.

Both the Arm E Patient Information Sheet and Arm E Patient Diary contain more specific instructions for patients to follow regarding how to take their medication.

Please refer to the Pharmacy Manual for further details.

26.3.1.2 Docetaxel

Docetaxel is indicated for the treatment of patients with locally advanced or metastatic NSCLC after failure of platinum-based chemotherapy. At a dose of 75 mg/m^2, docetaxel had a significant beneficial effect on OS compared with best supportive care, and a significantly higher 1-year survival compared to the control group of vinorelbine or ifosfamide (Shepherd *et al.* 2000, Fossella *et al.* 2000).

Docetaxel has been selected as the combination and comparator agent for this study as it is one of the most commonly prescribed drugs in second-line NSCLC, and remains a recognised standard of care in this setting (Pfister *et al.* 2004; Azzoli *et al*. 2011). The 75mg/m^2 dose of docetaxel selected is the licensed dose for patients with previously treated NSCLC and was the dose tested in combination with selumetinib in the completed phase I study (D1532C00004) and the completed phase II study (D1532C00016). The docetaxel dosage form and strengths are 20mg/1ml, 80mg/4 ml and 160mg/8ml IV.

Docetaxel should be administered on day 1 of every 21 day cycle at a dose of 75mg/m^2 intravenously over a 30-60 minute infusion. Docetaxel should be prescribed as per local practice. Patients are expected to receive up to 6 cycles of treatment administered on day 1 of every 21 day cycle until objective disease progression, intolerable toxicity or the occurrence of another discontinuation criterion. Further cycles of docetaxel may be administered if the treating Investigator feels it is beneficial and it does not contravene local practice. Investigators may decide to reduce the number of cycles of docetaxel if significant toxicity develops.

If docetaxel is discontinued for reasons other than disease progression, selumetinib should be continued until disease progression (please note patients who meet RECIST criteria for progressive disease (PD) may be continued on trial treatment if the treatment is tolerable and the Investigator believes it to be of clinical benefit; see Section 9.3), intolerable toxicity or the occurrence of another discontinuation criterion. Likewise, if selumetinib is discontinued during the first 6 cycles for reasons other than progressive disease, docetaxel may continue.

Please refer to the Pharmacy Manual for further details.

26.3.2 Non-Investigational Medicinal Products

26.3.2.1 Granulocyte colony stimulating factor (G-CSF)

In order to improve the benefit/risk ratio that may be expected by patients receiving selumetinib and docetaxel in this study G-CSF will be administered after every chemotherapy dose for primary prophylaxis of neutropenia. Dose, duration and timing around chemotherapy should be as per local practice.

Patients receiving 2nd line therapy for NSCLC have been exposed to chemotherapy agents in the 1st line setting. They may therefore already have underlying bone marrow depletion and be at increased risk of haematological toxicity from cytotoxic agents. Patients who received either docetaxel monotherapy or docetaxel in combination with selumetinib in the completed phase II Study D1532C00016 experienced such haematological toxicity. This was more frequent and severe in patients who received the combination with 17% experiencing an episode of febrile neutropenia. ASCO guidelines (ASCO 2006) recommend the administration of primary prophylactic G-CSF if a regimen is likely to induce a 20% or greater incidence of febrile neutropenia. In Study 16 the incidence of febrile neutropenia/neutropenic infection was 18.2% (8/44) for selumetinib+docetaxel vs 0% in placebo+docetaxel group.

Please refer to the Pharmacy Manual for further details.

26.3.3 Schedule of Assessments

Table 44: Selumetinib + Docetaxel - Schedule of Assessments

	**Screening**	**Treatment - Selumetinib 75 mg twice daily, Docetaxel 75mg/m^2 day 1 of cycle (21 day cycles)**						**Discontinuation** (+ 7 days)***	**28 day follow up[w]** (+ 7 days)***	**Post-28 day follow up** (± 7 days)****
	Within 28 days treatment (unless otherwise stated)	Cycle 1			Cycle 2	Cycle 3	Cycle 4 onwards			
		Day 1 # *****	Day 8 (± 1 day)*	Day 15 (± 1 day)*	Day 1 (± 2 days)**	Day 1 (± 2 days)**	Day 1 (± 2 days)**			
Informed consent[a]	X									
Demography & baseline characteristics[b]	X									
Medical history[c]	X									
Inclusion / exclusion criteria[d]	X									
Physical examination[e]	X	X (- 1 day)			X	X	X	X		
ECOG performance status[f]	X (within 14 days of treatment)	X (- 1 day)			X	X	X	X		
Vital signs (inc. weight)[g]	X	X (- 1 day)			X	X	X	X	(X)	
ECG[h]	X	if clinically indicated							(X)	

	Screening	Treatment - Selumetinib 75 mg twice daily, Docetaxel 75mg/m^2 day 1 of cycle (21 day cycles)						Discontinuation (+ 7 days)***	28 day follow up[w] (+ 7 days)***	Post-28 day follow up (± 7 days)****
	Within 28 days treatment (unless otherwise stated)	Cycle 1			Cycle 2	Cycle 3	Cycle 4 onwards			
		Day 1 # *****	Day 8 (± 1 day)*	Day 15 (± 1 day)*	Day 1 (± 2 days)**	Day 1 (± 2 days)**	Day 1 (± 2 days)**			
MUGA / Echocardiogram[i]	X						X (every 12 weeks ± 7 days)		(X)	
Ophthalmology examination[j]	X	if clinically indicated							X (if clinically indicated)	
Haematology, Clinical chemistry, [k]	X (within 7 days of treatment)	X (- 1 day)	X (- 2 days)	X (- 2 days)	X (- 2 days)	X (- 2 days)	X (- 2 days)	X	X ≠	
Urinalysis[l]	X				X (- 2 days)	X (- 2 days)				
Pregnancy test[m]	X	X (- 1 day)						X		
Tumour assessments[n]	X	Every 6 weeks during year 1 (± 7 days) [except 1st scan + 7days only]								X ◊
Adverse events & Concomitant Medications	X	X (- 1 day)	X	X	X	X	X	X	X	
Dispense selumetinib[o]		X (-2 days)			X (-2 days)	X (-2 days)	X (-2 days)			
Administer selumetinib[p]		BD Dosing								

	**Screening**	**Treatment - Selumetinib 75 mg twice daily, Docetaxel 75mg/m^2 day 1 of cycle (21 day cycles)**						**Discontinuation** (+ 7 days)***	**28 day follow up[w]** (+ 7 days)***	**Post-28 day follow up** (± 7 days)****
	Within 28 days treatment (unless otherwise stated)	Cycle 1			Cycle 2	Cycle 3	Cycle 4 onwards			
		Day 1 # *****	Day 8 (± 1 day)*	Day 15 (± 1 day)*	Day 1 (± 2 days)**	Day 1 (± 2 days)**	Day 1 (± 2 days)**			
Administer Docetaxel[p]		X			X	X	X			
Administer G-CSF[q]		X			X	X	X			
Smoking status[r]		X (- 1 day)					X (every 9 weeks)	X		
Germline DNA sample[s]		X (- 1 day)								
ctDNA samples[t]		X (- 1 day)					X (every 9 weeks beginning cycle 4) (- 2 days)	X		X $
Optional research biopsy[u]		X (post-reg, pre-tx)						X		
Survival status[v]										X

If ECG and pregnancy assessments have been performed within 14 days pre-treatment they do not have to be repeated prior to commencing treatment on cycle 1 day 1 if the patient's condition has not changed (no new treatment during this period of time, no new complication or aggravation).

(X) Patients who had a drop in LVEF >10% from baseline and an absolute value <Lower Limit of Normal (LLN) prior to or at the time of discontinuation of selumetinib should, where possible, have a follow up MUGA/ECHO, single ECG, and vital signs (including weight) performed 28 days after permanent discontinuation of selumetinib in order to document reversibility.

*	Visit may occur ± 1 days of the planned visit date. Where applicable and acceptable in accordance to local practices, visits may be performed by telephone or video call.
**	Visit may occur ± 2 days of the planned visit date. Individual assessments may occur independently of the visit date where indicated in the table above
***	Visit may occur + 7 days of the planned visit date
****	Visit may occur ± 7 days of the planned visit date
*****	Some Cycle 1 Day 1 assessments can be carried out -1 day of the Docetaxel infusion to allow flexibility due to Covid-19 associated clinic visit restrictions.

a Prior to the start of any study specific procedures, each patient must provide signed informed consent.

b Demography must be captured for all patients. Demographic data and other characteristics will include: date of birth, gender, race/ethnicity.

c A standard medical and surgical history will be obtained, including prior cancer treatment.

d Patients must not be registered unless all eligibility criteria have been fully met.

e Physical examination includes general appearance, respiratory, cardiovascular, skin, head and neck (including ears, eyes, nose and throat), lymph nodes, thyroid, abdomen, musculo-skeletal (including spine and extremities) and neurological systems and will be required at screening, day 1 of every cycle and at discontinuation. Following the discontinuation of Docetaxel and whilst remaining on selumetinib, assessments of physical exam may decrease in frequency to every 12 weeks (every 4 cycles on day 1).

f ECOG performance status will be assessed at screening, day 1 of every cycle and at discontinuation.

g Vital signs to be performed at screening, day 1 of every cycle and at discontinuation and includes height (screening only), weight, single measurements of supine BP and pulse will be recorded on each occasion after 10 minutes rest.

h ECG to be performed at screening and at any time if clinically indicated. Twelve-lead ECGs will be obtained after the patient has been resting semi-supine for at least 10 minutes prior to times indicated. All ECGs should be recorded with the patient in the same physical position. A standardised

ECG machine should be used and the patient should be examined using the same machine throughout the study if possible. After paper ECGs have been recorded, the Investigator or designated physician will review each of the ECGs and may refer to a local cardiologist if appropriate. A paper copy should be filed in the patient's medical records. If an abnormal ECG finding at screening is considered to be clinically significant by the Investigator, it should be reported as a concurrent condition. For all ECGs details of rhythm, ECG intervals (R-R, PR, QT and QRS) and an overall evaluation will be recorded.

i MUGA/ECHO should be performed every 12 weeks - after approximately the completion of every 4 cycles until discontinuation and as clinically indicated. The modality of the cardiac function assessments must be consistent within patient and the same machine operator is to be used where possible.

j Ophthalmology examination to be performed at screening and throughout study if clinically indicated.

Measurements of best-corrected visual acuity, intraocular pressure and slit-lamp fundoscopy (including photographs if abnormal) to be obtained at screening, and an OCT scan should be performed.

If a patient experiences an adverse event or symptoms of visual disturbance (including blurring of vision) a complete ophthalmological examination, including a slit-lamp examination, must be performed. If an abnormality is detected, fundus photography and an OCT scan can also be performed where required.

Patients with an ongoing retinal abnormality, prior to or at the time of discontinuation of selumetinib, should have a follow-up ophthalmological assessment approximately 28 days after discontinuation. This assessment is recommended to document reversibility, but should be performed only if the patient is fit enough to have the assessment.

k Samples to be collected at screening, day 1, 8 and 15 of cycle 1, day 1 of subsequent cycles and at discontinuation. Samples can be taken up to 2 days earlier than the actual visit date (where indicated). Where applicable and acceptable in accordance to local practices, blood tests can be performed locally in GP surgeries or in community based clinics.

Clinical chemistry: Albumin, AST, ALT, ALP, bilirubin (total), (GGT), calcium (total), creatinine, magnesium, phosphate, sodium, urea nitrogen, potassium, total protein and creatine kinase (CK).

Troponin (I/T - isoform as per institution norm) should be assessed at screening, on occurrence of significant LVEF drop (≥ 10 percentage points from baseline and an absolute value < LLN) or any cardiorespiratory events with no obvious diagnosis

Haematology: FBC. For patients receiving warfarin (coumarin derivatives), INR monitoring should be conducted and recorded.

All patients with AST, ALT or bilirubin ≥1.5 X ULN at the time of the last dose of selumetinib should have a further liver chemistry profile performed 28 days (±7 days) after permanent discontinuation of selumetinib.

≠ Only CK testing performed on 28 day follow up visit (e.g. no other haematology or clinical chemistry bloods taken on this visit).

l Urinalysis: Protein, glucose, blood. If urinalysis abnormal, perform microscopy – red blood cells, white blood cells, bacteria, casts, crystals at screening and pre-Docetaxel on day 1 of cycles 2 & 3. Samples can be taken up to 2 days earlier than the actual visit date (where indicated).

m Investigator should assess the patient's compliance to contraceptive measures and perform a test if required. Female patients of child-bearing potential only. A serum or urine pregnancy test is to be performed at screening, pre dose on cycle 1 day 1 and at discontinuation. In the event of suspected pregnancy during the study, the test should be repeated and, if positive, the patient discontinued from study treatment immediately.

n ◊ CT or MRI scan of head, chest and abdomen to be performed at screening. CT or MRI scans of chest and abdomen to be performed until discontinuation. Following screening, the first tumour assessments should be performed 6 weeks after cycle 1 day 1, then every 6 weeks thereafter for the first year, later reducing to every 12 weeks. Scans should be performed + 7 days (except 1st scan + 7 days only). If brain metastases are identified at Screening or if clinically indicated, head scanning should also be performed throughout treatment at the same time points. **The imaging modality must be used consistently throughout the course of the trial for each patient.** Tumour assessments will be performed in follow up for patients who discontinue treatment for reasons other than Progressive Disease (e.g. toxicity). These scans should be continue to be performed on a 6-weekly basis for the first year relative to the start date of treatment, then every 12 weeks until disease progression or the patient starts a new anti-cancer therapy (unless the patient withdraws consent to do so). Scans should be of the chest and abdomen, and only include the head where brain metastases are identified at screening, or if clinically indicated. All scans to be reported using RECIST 1.1.

o Selumetinib must be dispensed within the IWRS Cenduit system. Refer to the Pharmacy Manual for further details. Selumetinib may be dispensed within the IWRS up to 2 days prior to the actual visit date.

p Cycle 1 day 1: Treatment must commence within 7 days of trial registration

q G-CSF must be administered at least 24 hours after the administration of docetaxel and not within 14 days prior to the next docetaxel administration.

r Smoking status data will be collected through questions and CO monitoring at **pre-dose** cycle 1 day 1, cycle 4 day 1 then every 9 weeks (Day 1 of every third cycle) and at discontinuation.

s A whole blood germline DNA sample is to be collected **pre-dose** on Cycle 1 day 1. If the sample is not collected at this timepoint, it should be collected at the next visit. Refer to the Laboratory Manual for sample processing guidelines.

t ctDNA samples to be collected at **pre-dose** cycle 1 day 1, cycle 4 day 1 then every 9 weeks (Day 1 of every third cycle) and at discontinuation. Samples can be taken up to 2 days earlier than the actual visit date (where indicated). After 12 months of treatment, ctDNA sample collection may be reduced to 12 weekly in line with adjusted visit timing (see footnote 'y'). Refer to lab manual for sample processing instructions.

$ ctDNA samples will be collected in follow up for patients who discontinue treatment for reasons other than Progressive Disease (e.g. toxicity). These samples should be performed at the same visit as follow up CT or MRI scans until disease progression or the patient starts a new anti-cancer therapy (unless the patient withdraws consent to do so). Samples should be collected on a 6-weekly basis for the first year relative to the start date of treatment then every 12 weeks.

u An optional fresh metastatic/recurrent tumour biopsy sample should be collected (if patient consents) post–registration (pre-treatment) and at the end of treatment visit for patients who discontinue treatment for reasons other than disease progression (origin from either the primary tumour of site of metastasis). An optional pre-treatment biopsy should not be performed in cases where the patient has already had a mandatory biopsy for molecular testing (Note - a mandatory repeat SMP2 biopsy will be performed if the patient has had targeted therapy e.g. ALK inhibitor). The discontinuation biopsy must be performed prior to commencing further anti-cancer therapy. A post-treatment biopsy will only be requested from patients with an objective response or stabilisation of disease (PR or CR), or 6 months on treatment with evidence of stabilisation (SD) for patients who have previously progressed. The tumour tissue will be used to determine possible mechanisms of resistance to study treatment. Refer to the Laboratory Manual for sample processing instructions.

v Survival status will be collected every 12 weeks (± 7 days) post-permanent discontinuation of selumetinib or docetaxel (whichever is last) until death.

w 28 day follow up visit should be carried out 28 days (+ 7 days) post-permanent discontinuation of selumetinib or docetaxel (whichever is last).

y Once a patient has completed 12 months of treatment (approximately 17 cycles), visits may be reduced to 12 weekly at the discretion of the Investigator.

26.3.4 Toxicity Profile

26.3.4.1 Exposure

As of 31 January 2016, approximately 3080 patients with cancer have received treatment with selumetinib, including 1439 patients in AZ-sponsored studies, 57 patients in the Array-sponsored Study ARRY-0401, 62 patients in the Merck-sponsored Study D1532C00028, and approximately 1520 patients in Investigator- or collaborative group-sponsored studies. Of the Investigator-sponsored studies, 2 are being conducted in paediatric populations (Studies 8799 [NCT01362803; Phase I] and PBTC-029 [NCT01089101; Phase I/II]). In addition, 354 subjects (healthy volunteers or subjects with renal or hepatic impairment) have been exposed to selumetinib Hyd-Sulfate in clinical pharmacology studies.

26.3.4.2 Expected Adverse Events - Selumetinib

Please refer to the IB for the full AE profile for selumetinib.

26.3.4.2.1 Events regarded as expected for regulatory reporting purposes

- Skin and subcutaneous: rashes (including dermatitis acneiform and exfoliative rash), dry skin, paronychia.
- General: facial and/or peripheral oedema, fatigue/asthenia, pyrexia.
- Respiratory: dyspnoea.
- Eye: blurred vision.
- Physical assessments: increased BP, reduced LVEF.
- Laboratory changes: increases in serum AST or ALT, hypoalbuminaemia, anaemia, hyperphosphataemia, which may be associated with an increase in calcium x phosphate product requiring therapeutic intervention.

In combination with docetaxel:

- Haematological: a higher incidence of febrile neutropenia has been reported in patients receiving selumetinib in combination with docetaxel than in patients receiving placebo plus docetaxel.
- Haematological: a higher incidence of CTCAE Grade 4 low neutrophil counts has been reported in patients receiving selumetinib in combination with docetaxel than in patients receiving placebo plus docetaxel.

It is possible that Asian subjects may experience higher selumetinib plasma exposure (than would be expected in Western subjects receiving the same dose of selumetinib), there could be a potential for a higher risk of adverse events. The number of Asian patients with advanced cancer who have received treatment with selumetinib is very low.

26.3.4.2.2 Expected side effects of selumetinib

The following expected site effects have been reported for patients treated with selumetinib.

Diarrhoea: Diarrhoea is common in patients receiving selumetinib and may occur within days of starting treatment, and therefore early treatment intervention is recommended. Patients should call the study team immediately if experiencing diarrhoea and may be given a treatment to be started immediately if they get diarrhoea. See Figure 19: Selumetinib - Management Guidelines for Patients with Diarrhoea and corresponding section for diarrhoea management guidance.

Nausea and/or vomiting: Nausea/vomiting are common in patients receiving selumetinib and generally start within the first month of treatment with selumetinib. Patients should call the study team immediately if experiencing nausea and/or vomiting.

Fever: This is usually a sign of infection, but in patients receiving selumetinib fever can also occur without any associated infection. Patients should call the study team immediately if experiencing fever. Tests should be conducted to confirm the reason for the fever.

Difficulty breathing or shortness of breath (dyspnoea): If patients get breathless, or experience breathlessness getting worse, patients should call the study team immediately. See Figure 21: Selumetinib – Management Guidelines for Patients with Dyspnoea for safety algorithm for dyspnoea.

Interstitial Lung Disease-type events: A small number of patients have developed inflammation of the lungs causing coughing and difficulty breathing when selumetinib is given in combination with docetaxel in advanced non-small cell lung cancer.

Blockage of blood vessels in the eyes: A small number of patients have experienced a blockage of a blood vessel in the eye, which may temporarily affect vision. See Figure 22: Selumetinib – Management Guidelines for Patients with Visual Symptoms for safety algorithm for visual changes.

Blurring of vision: Some patients have experienced changes in vision. Any visual symptoms should be reported to the study team immediately. See Figure 22: Selumetinib – Management Guidelines for Patients with Visual Symptoms and corresponding section for safety algorithm for visual changes.

Fluid accumulation in the layers at the back of the eye: A small number of patients have experienced changes in the eye such as fluid accumulation in the layers at the back of the eye, which may temporarily affect vision.

Decrease in LVEF: Asymptomatic reductions in left ventricular ejection fraction to below 55% have been recorded in a small number of patients during treatment with selumetinib. Evidence of reversibility on continuing treatment with selumetinib has been demonstrated in some patients.
Swelling of the face or extremities is commonly reported in patients receiving selumetinib.

Rash, dry skin and nail changes: Rash occurs commonly but is usually mild. Acne-like rash, usually on the face and/or upper body is typical with selumetinib treatment. It may also appear elsewhere on the body. Generally, it will appear within the first few weeks of the study treatment. Dry skin can also occur in patients receiving selumetinib. Patients should avoid sun exposure. Some patients may also experience soreness or infection in the skin around fingernails or toenails. See Figure 18: Selumetinib - Guidance for the Management of Patients with Rash for safety algorithm for rash management.

Anaemia may occur in some patients and this requires regular monitoring. Common treatable causes of anaemia (eg, iron, vitamin B12 or folate deficiencies) should be investigated and appropriately managed.

Tiredness occurs commonly in patients receiving selumetinib.

Soreness or inflammation of the mouth can begin within days of starting treatment and usually within the first month in the majority of patients who will experience this AE. Dry mouth

has also been reported by patients taking selumetinib. See Oral Care sub-section in Dose Modifications section for safety algorithm for oral care management.

Abnormal laboratory results: These will be checked during the study.

- From the liver: (increases in some liver proteins called aminotranferases)
- Increase in phosphate level in the blood
- Low blood protein (albumin) level.
- Increase in the blood level of a muscle enzyme creatine phosphokinase (CK).

26.3.4.2.3 Reported events but unknown causality

Effects on muscle: In a small number of patients, weakness of the neck muscles has been reported; this returned to normal after interrupting selumetinib treatment.

Abnormal laboratory results: A small number of patients treated with selumetinib or other drugs that act in the same way as selumetinib have experienced increase in the blood levels of a muscle enzyme called creatine phosphokinase (CPK).

Effects on the lungs: A small number of patients have developed shortness of breath or cough, sometimes with fever, because of inflammation or infection of the lungs.

Effect on large intestine: 2 patients who were receiving selumetinib in combination with gemcitabine (1250mg/m^2) and cisplatin (75mg/m2 q 3 weeks) developed the side effect of large intestine perforation (a hole in the large bowel), and this side effect resulted in death. Because of other concurrent conditions contributing to these events, large intestine perforation was not considered to be related to selumetinib.

26.3.4.3 Expected Adverse Events – Docetaxel

Expected toxicities include hypersensitivity reactions, febrile neutropenia, peripheral neuropathy, and fluid retention. Please refer to the docetaxel product information for a complete listing of adverse events associated with administration of docetaxel. The incidence of severe fluid retention can be reduced by treatment with dexamethasone for 3 days beginning the day before docetaxel administration. Dexamethasone should be given orally. Use of dexamethasone is a recommendation, given at the local Investigator's discretion. It is not mandatory. Additional doses of intravenous dexamethasone can be given at the local Investigator's discretion.

26.3.5 Dose Modifications and Toxicity Management

26.3.5.1 Treatment delays and discontinuation

For all AEs that are considered at least partly due to administration of selumetinib the following dose reduction/adjustment guidance should be applied:

a) Treatment with selumetinib should be temporarily interrupted if one of the following AEs occurs despite optimal supportive care and is considered related to treatment with selumetinib:
    - Any intolerable AE regardless of grade.
    - Any AE ≥ CTCAE Grade 3 (despite optimal supportive care).

b) On improvement of the AE to CTCAE Grade 1 (or to CTCAE Grade 2 for rash, or to >LLN and within 10 percentage points of baseline for LVEF decreases) or baseline within 4 weeks of onset (or within 6 weeks of onset for asymptomatic reductions in LVEF and for

RPED/central serous retinopathy), selumetinib may be restarted at a reduced dose at the discretion of the local Investigator. Step-wise dose reductions of selumetinib (see Table 45: Selumetinib - Available Dose Levels) then permanent discontinuation, are allowed.

- If a further episode of the same AE subsequently requires dose interruption, selumetinib may be restarted at the next dose level down on improvement of the AE.
- If a different AE subsequently requires dose interruption, selumetinib may be restarted at the same dose or at the next dose level down on improvement of the AE.

c) Selumetinib should not be re-escalated to an earlier dose level on improvement of an AE, while a patient remains on combination therapy. The Schedule of Assessments (see section 26.3.3) should continue relative to the date of the first dose (Cycle 1 Day 1) in the event of selumetinib dose interruption of reduction.

d) Treatment with selumetinib should be permanently discontinued for any CTCAE Grade 4 toxicity that is at least partially related to selumetinib. Re-introduction of selumetinib may be considered for episodes of CTCAE Grade 4 neutropenia or Grade 4 thrombocytopenia, or on a case-by-case basis for other Grade 4 AEs in consultation with the National Lung Matrix Trial Office.

e) For patients experiencing treatment delays due to toxicity that exceed 3 weeks, the Investigator should consider permanent treatment discontinuation on consultation with the National Lung Matrix Trial Office, and the patient should be observed until resolution of the toxicity.

In the event of a treatment interruption for reasons other than treatment related toxicity (e.g., non-cancer related surgery) lasting >3 weeks, treatment resumption will be decided on consultation with the National Lung Matrix Trial Office. It is advised that treatment delays should not exceed 6 weeks.

If either drug is discontinued for reasons other than progressive disease during the first 6 cycles, the other drug may be continued. See Trial Treatment Section 26.3 for further details.

Specific guidance for interruption or reduction of treatment with selumetinib may be considered for particular events, as indicated in the algorithms included in the Evaluation, Management and Treatment of Toxicities section below.

26.3.5.2 Dose reductions

Table 45: Selumetinib - Available Dose Levels

**Dose level**	**Selumetinib dose**
Starting dose	75 mg BD
-1	75 mg OD
-2	50 mg BD
-3	50 mg OD
N/A	Discontinue trial treatment

There is no evidence that the majority of adverse drug reactions to selumetinib are related to the maximum concentration (Cmax). An analysis of selumetinib in combination with dacarbazine in a Phase 3 study, indicates a relationship between severity of neutropenia and selumetinib AUC (data on file). This is not unexpected given the mechanism of action. An

analysis of the pharmacodyamic effect, ERK phosphorylation, in an early clinical study indicates that there is a direct relationship between plasma concentration and inhibition of phosphorylation. Therefore activity is not mediated via Cmax, but through attainment and maintenance of plasma selumetinib concentrations sufficient to inhibit MEK1/2. As selumetinib has a short plasma half life of approximately 6-8 hours, the 75 mg dose and BD regimen is recommended to achieve and maintain plasma concentrations sufficient for activity.

AstraZeneca acknowledges that the dose reduction schedule for selumetinib has an empirical component however available data indicates that AUC is important for the severity of neutropenia.. As all efficacy data derived from the program to-date has been with the currently described dose reduction schedule, changing the suggested scheme would carry risk to the development program and to the results of the study as planned. AstraZeneca believes that the evidence of a new dose reduction schedule improving benefit-risk is needed before implementation of a change. Currently available information, including the data that supports conducting this study, has used the existing dose-reduction schedule.

Investigators are encouraged to assess the success of each dose reduction and if tolerability is not improved to change to an alternate schedule with the aim of preserving clinical activity. For example, if tolerability is not improved through the first dose reduction to 75mg OD (which primarily reduces AUC), the second reduction to 50mg BD provides a Cmax reduction while maintaining a higher AUC (with the intention of maintaining efficacy). The intention is to afford patients a favourable benefit-risk profile through the dose-reduction schedule by seeking to maintain AUC-driven efficacy if tolerability has not been improved by the initial reduction.

These steps in dose reduction represent an initial reduction in AUC followed by a reduction in Cmax. As it is anticipated that the majority of AEs are associated with selumetinib AUC, the proposed schedule should maintain a favourable benefit-risk profile.

No dose adjustment is recommended in patients with mild hepatic impairment.

No dose adjustment is also recommended in patients with mild, moderate, or severe renal impairment, or those with ESRD.

26.3.5.3 Missed Doses

Wherever possible, selumetinib doses should not be missed. If a patient misses taking a scheduled dose, window ± 2 hours, they should take the next dose at the next scheduled time and the missed dose is not to be made up. If a patient vomits after taking their selumetinib medication, they should not make up for this dose, but should take the next scheduled dose. It is not acceptable to take the entire daily dose (i.e., 150 mg for adults on continuous dosing) at once.

26.3.5.4 Evaluation, Management and Treatment of Toxicities – Selumetinib

For some specific AEs, recommendations for investigation or management exist in algorithm form:

26.3.5.4.1 Rashes

Table 46: Selumetinib + Docetaxel – Example Topical Steroids and Antibiotics

Topical steroids moderate strength	Triamcinolone acetonide 0.025%<br>Desonide 0.05%

	Fluticasone proprionate 0.05%<br>Aclometasone 0.05%
Topical antibiotics	Clindamycin 1 - 2%<br>Erythromycin 1% - 2%<br>Metronidazole 1%<br>Silver sulphadiazine 1%
Oral antibiotics	Doxycycline 100 mg BD<br>Minocycline 100 mg BD<br>Oxytetracycline 500 mg BD

Figure 18: Selumetinib - Guidance for the Management of Patients with Rash

Recommendations to start on day 1 of treatment with selumetinib/placebo and continue for the whole duration of treatment

- Use skin moisturiser (thick, alcohol-free) at bedtime
- Avoid excessive exposure to sunlight
- Use sunglasses/sunscreen (PABA-free, SPF ≥15; UVA and UVB protection) as needed
- Use of topical retinoids or benzoyl peroxide is not recommended

CTC Grade 1 rash

→ Apply a mild / moderate strength topical steroid and/or topical antibiotic.

CTC Grade 2 rash

→ Apply a moderate strength topical steroid and consider an oral antibiotic

CTC grade ≥3 rash or a CTC grade 2 rash considered by the patient to be intolerable

→ Interrupt selumetinib dose.

Apply moderate strength topical steroid and consider an oral antibiotic. If an infection is suspected, consider other broad spectrum antibiotic cover.
Also consider referral to a dermatologist (manage rash per recommendation)

→ If rash has improved to Grade 2 or less

→ Re-start selumetinib at original or reduced dose (apply investigator's discretion)

Example antibiotics and topical steroids (dosage regimens as per local guidelines):

Topical steroids moderate strength: Triamcinolone, Desonide, Aclometasone
Topical antibiotics: Clindamycin, Erythromycin, Metronidazole, Silver sulphadiazine.
Oral antibiotics: Doxycycline, Minocycline, Oytetracycline

26.3.5.4.2 Recommendations for Diarrhoea Management

Diarrhoea may occur during treatment with selumetinib and action should be taken as soon as symptoms develop. The recommendations for diarrhoea management are based on guidelines from ASCO (J Clin Oncol 2004; 22:2918-26). These guidelines recommend that treatment-induced diarrhoea should be carefully monitored and treated aggressively to ensure that severe complications are avoided and that treatment is not delayed.

- Patients should be made aware that they are likely to experience diarrhoea and be encouraged to record the number of stools and report possible associated symptoms.
- Patients should be given loperamide (in accordance with local regulation and local practice) to take home with them and be advised to start immediately after the first episode of unformed stool.
- Patients should be given dietary advice in case of diarrhoea (e.g. BRAT [bananas, rice, apple sauce, toast, plain pasta] diet; readily digestible food; avoidance of lactose-containing products, fried, fatty or spicy food) and increase fluid intake (8–10 glasses of clear fluids daily, including water and fluids containing salt and sugar, such as sports drinks and clear broth).
- Patients should seek advice early, from their physician or study nurse, if:
    - Persistent Grade 1 or 2 diarrhoea (see Table 47: CTCAE (version 4) grading for diarrhoea) or
    - Grade 3 or 4 diarrhoea.
    - Diarrhoea becomes complicated by associated vomiting or inability to take oral fluids; marked abdominal distension or cramping; bloody stools, fever or symptoms of hypotension.

Table 47: CTCAE (version 4) grading for diarrhoea

CTCAE Grade	Patients without colostomies	Patients with colostomies
Grade 1	Increase in number of stools per day (<4)	Mild increase in loose watery colostomy output compared with pre-treatment
Grade 2	Increase in number of stools per day (4-6) or nocturnal episodes	Moderate increase in loose watery colostomy output compared with pre-treatment, not interfering with normal activity
Grade 3	Increase of more than 7 stools per day or incontinence or needing support for dehydration.	Severe increase in loose watery colostomy output compared with pre-treatment and interfering with normal activity
Grade 4	Life-threatening consequences (eg, hemodynamic collapse)	

Initial management of uncomplicated Grade 1 or 2 diarrhoea

- Patients should immediately start loperamide after the first episode of diarrhoea. The recommended dose is 4 mg initially and loperamide continued at a 2 mg dose every 4 hours after each unformed stool, until the patient is free from diarrhoea for at least 12 hours.

- If after 12 hours of loperamide treatment the diarrhoea is not improving or resolved, the patient should be instructed to contact the study team and to increase to high dose loperamide (2 mg every 2 hours, or 4 mg every 4 hours at night) and continue to take loperamide until they have been free from diarrhoea for at least 12 hours. Additional treatment may be considered according to local practice.

<u>Management of persistent (>24 hours) Grade 1 or 2 diarrhoea despite high dose loperamide</u>

The patient should be seen by the local Investigator or study nurse for full evaluation and the following should be considered:

- Rehydration and electrolytes replacement as appropriate.
- Infectious causes and aetiologies such as Clostridium difficile or viral gastroenteritis.
- Antibiotics if appropriate (for example an oral fluroquinolone for 7 days) particularly if the patient is neutropenic (<1 x 10^9/L) or has a fever.
- Discontinuation of loperamide and start of octreotide (Sandostatin).

It may also be appropriate to consider:

- Addition of other second-line anti-diarrhoeal agents according to local practice.
- Selumetinib interruption until resolution of the diarrhoea.
- Hospitalisation.

<u>Management of any grade uncontrolled or complicated diarrhoea, or Grade 3-4 diarrhoea</u>

- Hospitalisation and full evaluation.
- Intravenous fluids, electrolytes and antibiotics if needed (e.g. fluroquinolone).
- Interrupt selumetinib until diarrhoea and associated symptoms resolve.
- Start octreotide (Sandostatin).

Figure 19: Selumetinib - Management Guidelines for Patients with Diarrhoea

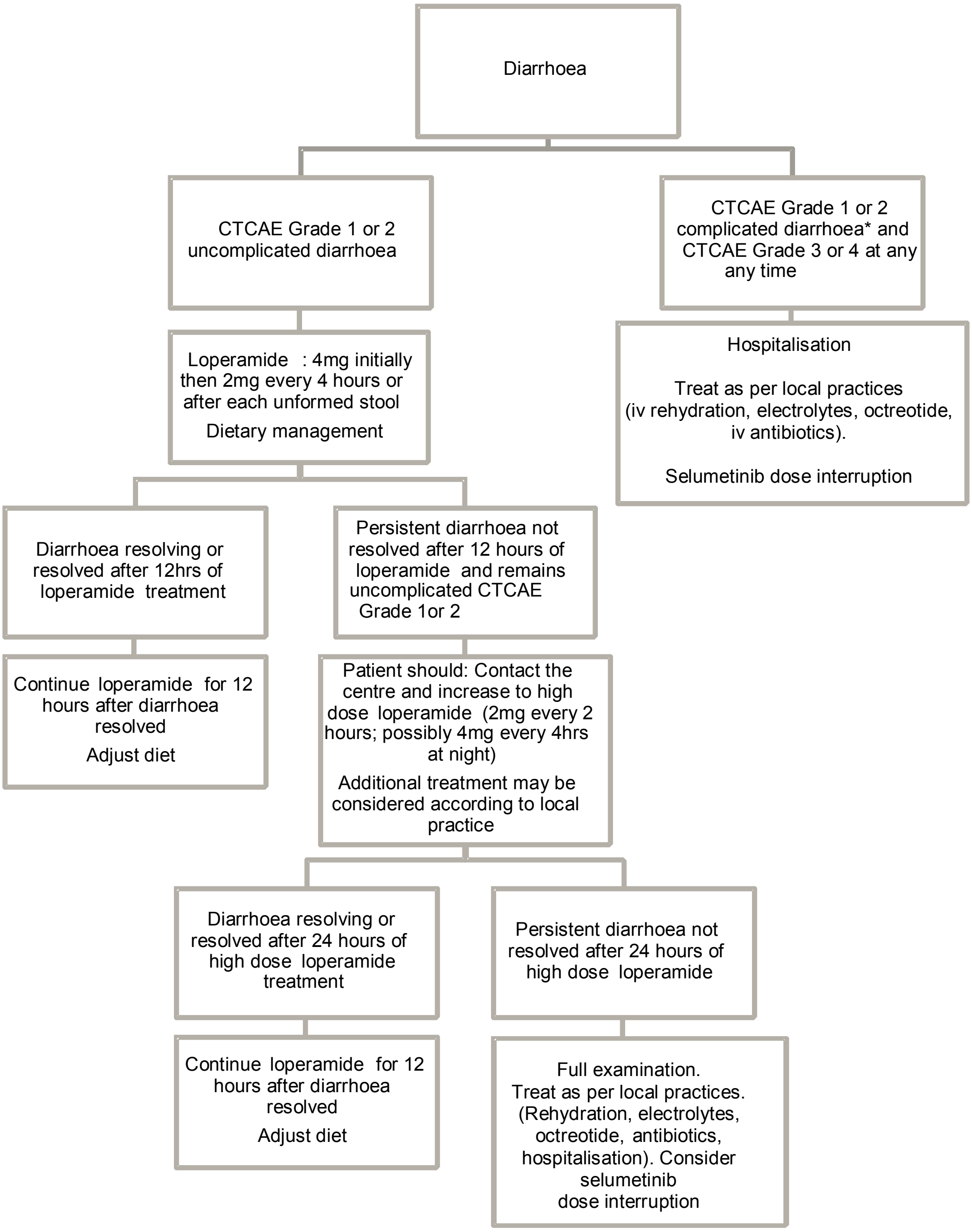

*Diarrhoea becomes complicated by associated vomiting or inability to take oral fluids; marked abdominal distension or cramping; bloody stools, fever or symptoms of hypotension

26.3.5.4.3 Management of Asymptomatic Left Ventricular Ejection Fraction Reduction

Reduction in LVEF: asymptomatic reductions in LVEF have been reported in some patients receiving selumetinib. Treatment may be recommended depending on the magnitude of the LVEF reduction (Figure 20).

An ECHO/MUGA will be conducted at baseline, and every 12 weeks whilst on treatment. A further ECHO/MUGA should be performed as part of the assessment package for any cardio respiratory adverse event with no obvious diagnosis (obvious causes will be managed in accordance with local clinical practice) and additionally at the discretion of the Investigator if clinically indicated.

LVEF, end diastolic and end systolic left ventricular volumes should be recorded at each echocardiogram assessment.Echocardiography/MUGA will also be carried out if a patient develops signs and/or symptoms suggestive of deterioration in left ventricular function/cardiac event.

Patients who had a drop in LVEF of (decrease by ≥ 10 percentage points relative to baseline and to an absolute LVEF below the institution's LLN) prior to or at time of discontinuation of selumetinib should, where possible, have a follow-up ECHO/MUGA (single ECG) and vital signs (including weight) performed 30 days (+/- 7 days) after permanent discontinuation of selumetinib. These assessment are recommended to document reversibility, but should be performed only if the patient is fit enough to have the assessment.

Figure 20: Selumetinib - Management of Asymptomatic LVEF Reduction

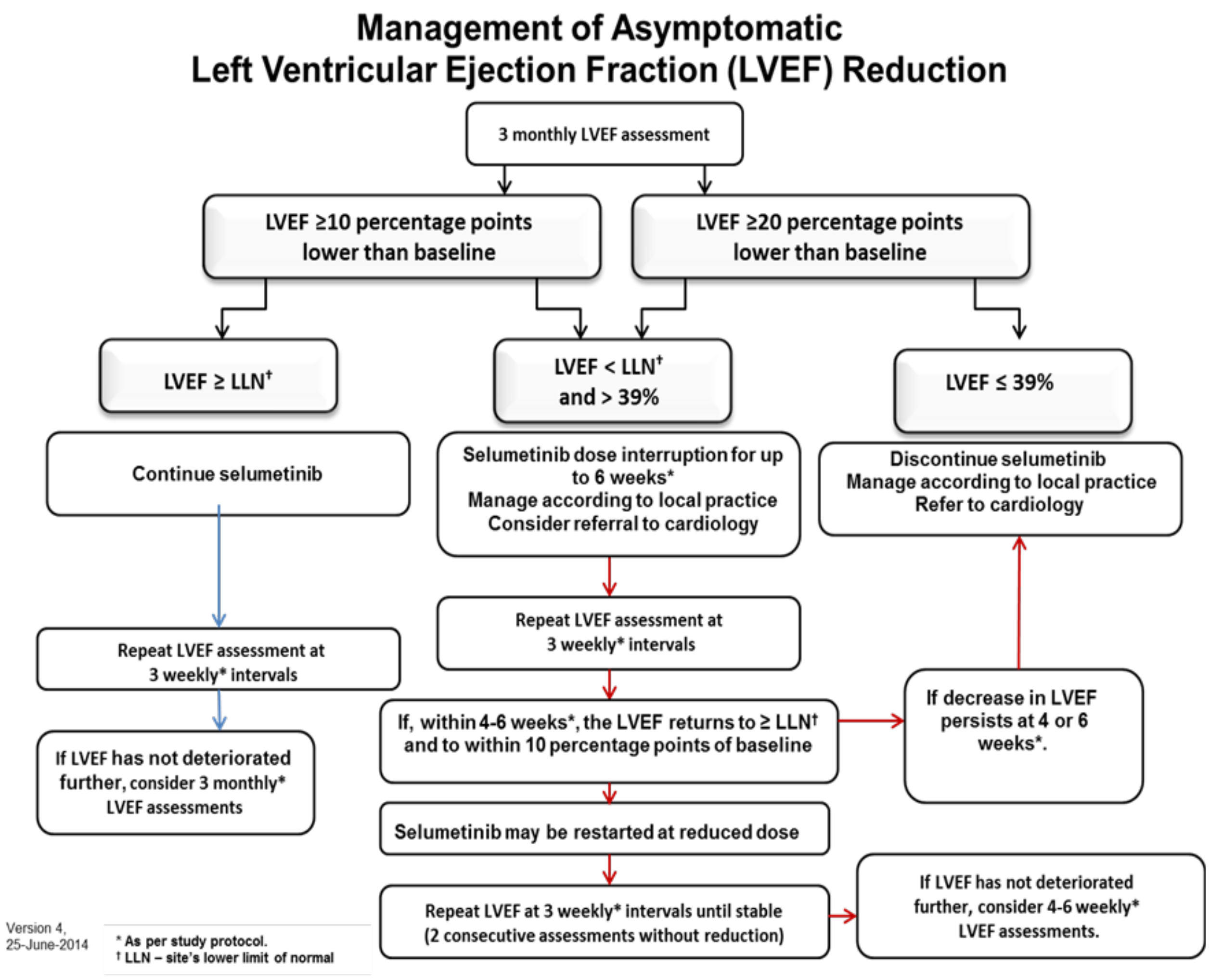

26.3.5.4.4 Dyspnoea

Dyspnoea: new or worsening dyspnoea has been reported commonly during treatment with selumetinib; investigation to determine the underlying cause is recommended (Figure 21).

Figure 21: Selumetinib – Management Guidelines for Patients with Dyspnoea

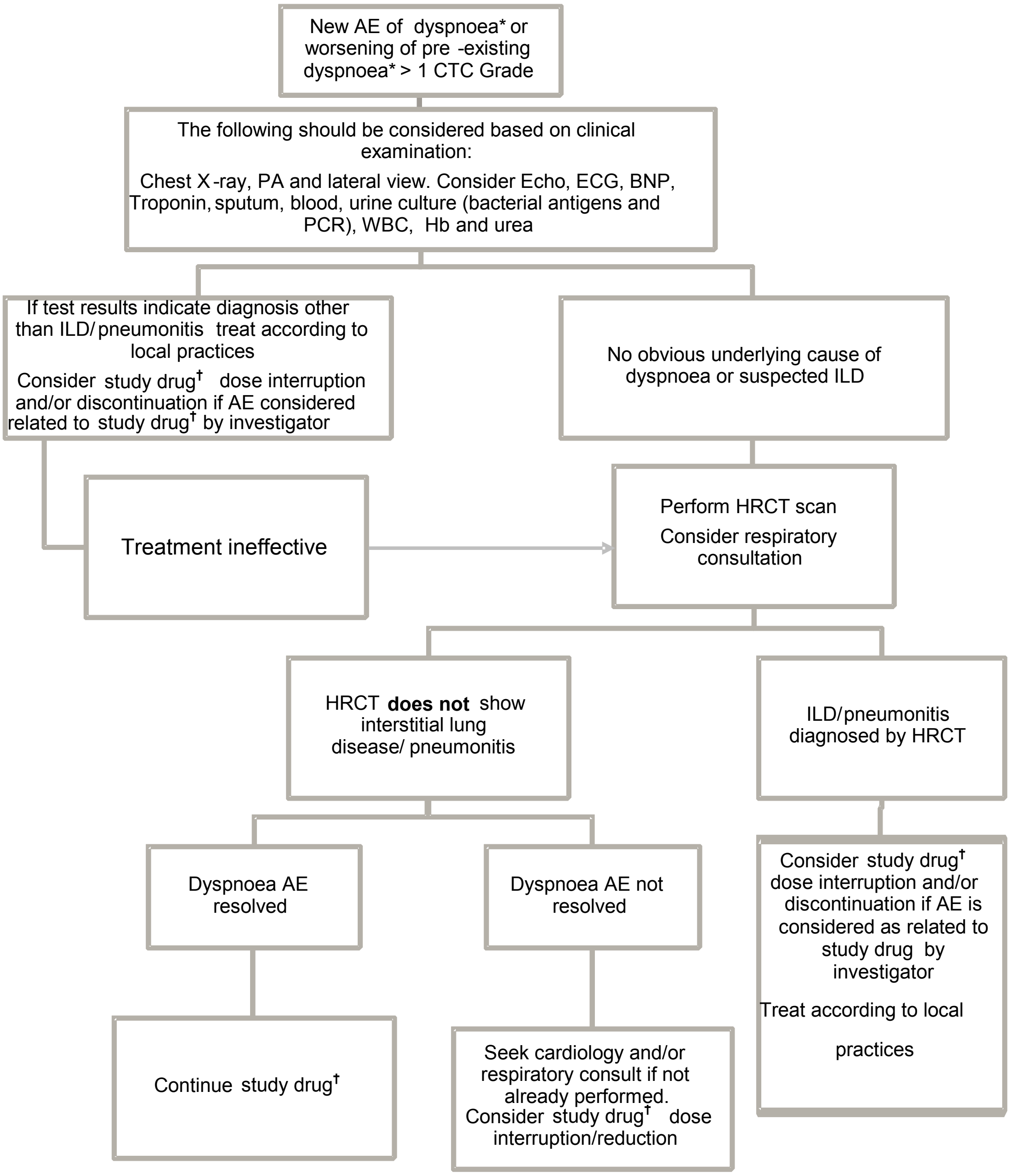

BNP: Brain Natriuretic
CTC: National Cancer Institute common toxicology
Echo: Echocardiography
Hb: Haemoglobin
HRCT: High resolution computerised tomography
ILD: Interstitial Lung
PA: posteroanterior
PCR: Polymerase chain
WBC: White blood cells

*Not considered related to disease under study † Includes selumetinib or combination as appropriate

26.3.5.4.5 Management of Visual Changes/Symptoms

Symptoms including blurred vision occur commonly during treatment with selumetinib, and AEs of central serous retinopathy and retinal vein occlusion have been reported in studies of other MEK inhibitors. Investigation to determine the underlying cause of visual disturbance is recommended (Figure 22: Selumetinib – Management Guidelines for Patients with Visual Symptoms).

An ophthalmological examination should include best corrected visual acuity, intraocular pressure measurement and slit-lamp fundoscopy, and should be performed at screening and as clinically indicated whilst the patient is on study drug. If these examinations indicate retinal abnormality, OCT scan should be considered if clinically appropriate.

If a patient experiences AE/symptoms of visual disturbance (including blurring of vision) a complete ophthalmological examination, including a slit-lamp examination, must be performed. If an abnormality is detected, fundus photography and an OCT scan can also be performed where required.

If retinal abnormality prior to or at time of selumetinib discontinuation has been observed a repeat ophthalmological examination is to be performed 30 days after discontinuation of selumetinib in order to document reversibility.

Figure 22: Selumetinib – Management Guidelines for Patients with Visual Symptoms

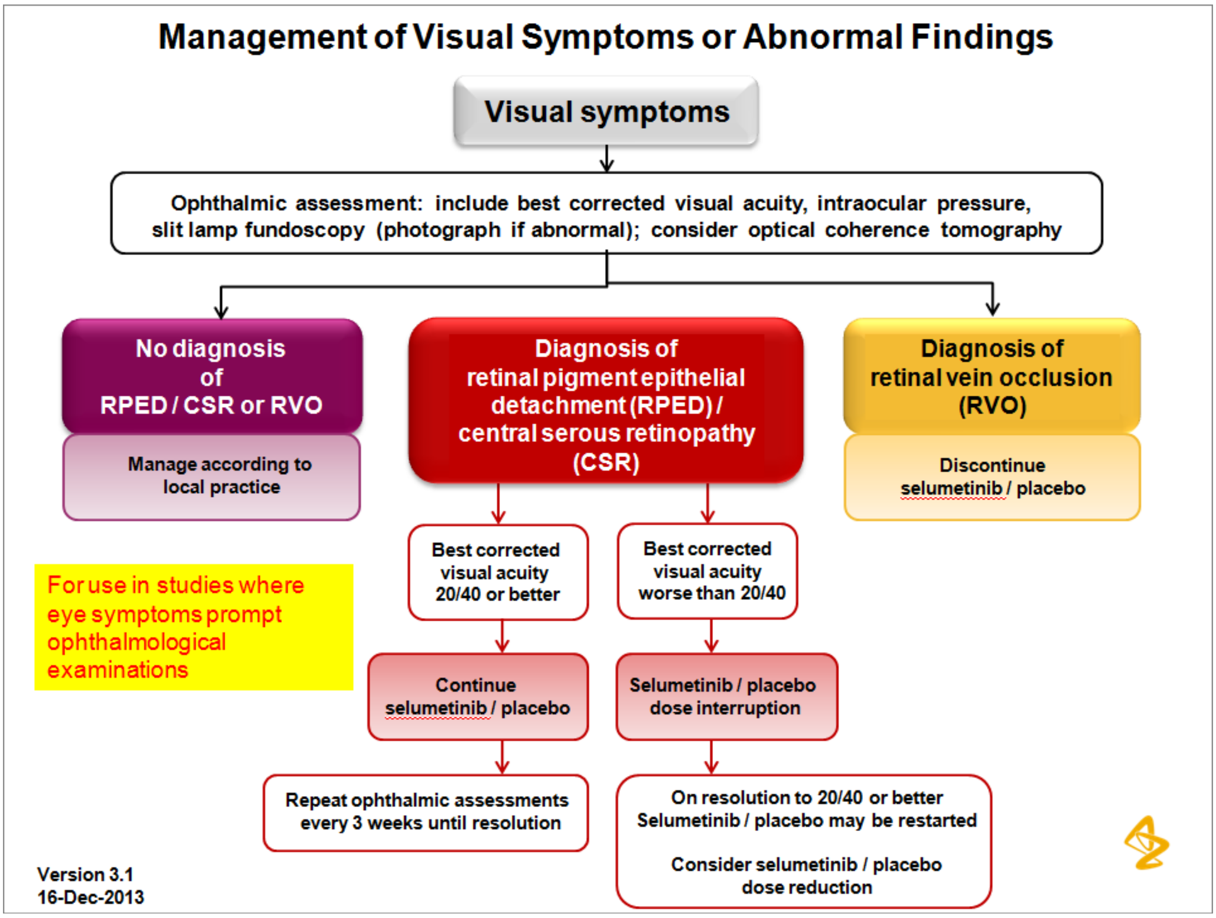

26.3.5.4.6 Oral Care

Oral care guidance is recommended during combination treatment with selumetinib + docetaxel as a higher incidence of stomatitis (oral mucositis) has been reported in patients receiving this combination than would be expected from the monotherapy profiles of either agent.

Patients should be encouraged to take responsibility for their own oral care wherever possible. This may require frequent encouragement and education. The general recommendations of Rubenstein *et al*. (2004) are to maintain a clean and pain-free mouth, which reduces patient discomfort and helps prevent infection and promote dietary intake. Evidence from the literature regarding implementation and efficacy of oral protocols and patient education, suggest that patients who are taught oral care protocols perform oral care more diligently, take more responsibility for their care and may show an improvement in oral symptoms.

Prevention, early diagnosis and management of stomatitis may reduce the need for dose interruption and/or reductions of the study medications due to severe stomatitis and so allow the patient to continue on the study drugs. It is strongly recommended that patients receive advice regarding daily oral health care regimes, both before and during treatment.

Mouthwashes:

- Patients with a healthy mouth may use non-alcoholic mouthwash several times (4 to 6 times daily, or according to the instructions) daily e.g. after each meal during the study.
- Saline mouthwashes (Sodium chloride 0.9%) should be preferred in cases of stomatitis, and should be used at a different time to toothbrushing e.g. after tea.
- Use of a mouthwash immediately after selumetinib intake is recommended.
- The tongue can be gently brushed (if not sore) with a soft toothbrush.
- Patients with, or at risk of stomatitis should not use commercial/over-the-counter mouthwashes because of the alcohol content and astringency. Chlorhexidine mouthwashes are not recommended for the treatment of established stomatitis.
- The mouth should be regularly inspected by the patient and healthcare professionals.

Smoking:
Smoking should be strongly discouraged; patients should be offered help with smoking cessation if necessary in the form of nicotine replacement therapy or referral to smoking cessation services.

Alcohol and food intake:
A high alcohol intake should be discouraged and patients advised to avoid painful stimuli such as spicy foods, hot food and drink.

Dental care:
Dentate patients:

- Patients who are free from dental problems may be at less risk of stomatitis.
- Teeth should be brushed twice daily with a fluoride toothpaste and soft toothbrush, in the morning before breakfast and last thing in the evening before bed, about 30 minutes after eating. Toothbrush should be replaced regularly at least every 3 months but patients with stomatitis should change their toothbrush every 4 - 6 weeks.
- Use of soft toothbrush is recommended.
- Dental floss should be used once daily (caution in patients with coagulopathies including a low platelet count).

Edentulous patients:

- Dentures should be left out whilst at rest.
- Dentures should be cleaned thoroughly twice daily (before and after soaking overnight) and after every meal using a soft toothbrush and denture cleaner water.
- Dentures should be soaked overnight in a mild denture-soaking solution.

In the event of sore mouth or stomatitis:

- Consider treating stomatitis at an early stage (CTCAE grade 1) or as soon as the patient complains of a sore mouth.
- Consider using oral topical analgesic anaesthesia with or without topical steroids, antiviral and/or antifungal medications depending on the patient's clinical condition and the local standard medical practice.

26.3.5.5 Extra assessments

Extra assessments are recommended for investigation of specific adverse events occurring during treatment with selumetinib

Table 48: Selumetinib + Docetaxel - Assessments recommended for specific AEs

Adverse event	Assessment
Clinically significant LVEF reduction (by ≥10 percentage points and to below LLN)	**Single ECG**
Cardio-respiratory AEs of non-obvious cause	**Single ECG**
New or worsening respiratory symptoms (such as dyspnoea, cough)	**Single ECG**
Clinically significant LVEF reduction (by ≥10 percentage points and to below LLN)	**Troponin levels** (isoform according to institutional norm)
Cardio-respiratory AEs of non-obvious cause	**Troponin levels** (isoform according to institutional norm)
Unexplained muscle weakness	**Local diagnostic algorithms**

26.3.5.5.1 Creatine Kinase elevation

Some previous studies of selumetinib have included measurement of **creatine kinase (CK) levels** at baseline and at intervals during study treatment.
Elevation in total Creatine Kinase (CK) levels occur very commonly during treatment with selumetinib and are apparent within the first 2 weeks of starting treatment. CK elevations are predominately of low grade (1 or2 – grade increase from baseline) and are asymptomatic in the majority of patients. The CK-MM fraction accounts for the total CK elevation in the majority of patients, suggesting that the increase in total CK is generally of muscle origin (refer to Section 6.6.1.13 of Selumetinib IB, edition 16, dated 6 January 2016).

CK/CPK elevation has been reported with some MEK inhibitors. CK is known to be released from a number of tissue types, including skin and muscle. The origin of elevations reported in patients receiving MEK inhibitors is unknown.

To reduce possible consequences associated with CK elevation that may be arising from a muscular source, the management guideline described in Figure 23.

Figure 23: Selumetinib - Management Guidelines for patients with CK elevation

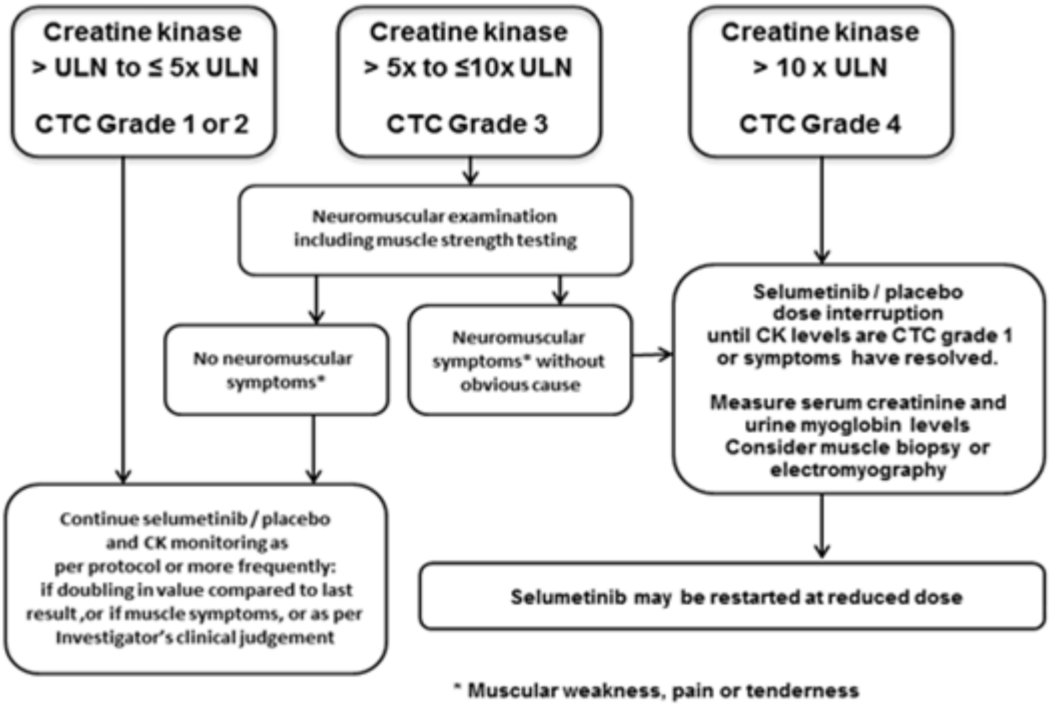

Laboratory values of 'CK elevation' should only be reported as adverse events (AEs) if they fulfil any of the serious AE criteria, or if they are the reason for discontinuation of treatment with selumetinib.

Muscular symptoms concurrent with CK elevation should be reported as AEs. If appropriate, the definitions below should be applied (Table 49).

Table 49: Selumetinib - Muscular symptoms & CK Elevation

**Adverse event**	**Definition**
Myopathy	CK > 10x ULN (CTCAE Grade 4)<br>CK > 5x ULN (CTCAE Grade 3) with muscle symptoms
Rhabdomyolysis	CK > 10x ULN (CTCAE Grade 4) and all of the following:<br>- muscle symptoms<br>- myoglobinuria<br>- renal impairment (serum creatinine 2x baseline, or above ULN)

26.3.5.5.2 Additional On-Study Safety Procedures

Information on the management of AEs for patients on selumetinib: The following situations require additional safety monitoring:

- All cardiorespiratory AEs with no obvious diagnosis should be assessed with a single ECG, ECHO/MUGA, vital signs (resting BP, pulse), weight and blood samples for Troponin (isoform per institution norm) taken at the time of the event.

- Measurements of Troponin (isoform per institution norm) and recording single ECG to be performed when there is a significant drop in LVEF (decrease by > or equal 10 percentage points and to an absolute value < LLN).
- Hyperphosphataemia and increase in the calcium phosphate product should be managed according to local practice.
- If muscle weakness, myalgia (muscle pain) occurs that cannot be explained the patient should have a neuromuscular examination, urine analysis and creatine phosphokinase (CPK) measurement performed (with where possible an additional CPK-MM) and be managed according to local practice.

26.4 References

- 2010 update of EORTC guidelines for the use of granulocytecolony stimulating factor to reduce the incidence of chemotherapy-induced febrile neutropenia in adult patients with lymphoproliferative disorders and solid tumours (2011). *Eur J Cancer* **47:**8-32.
- Azzoli CG, Temin S, Aliff T *et al.* (2012) Focused Update of 2009 American Society of Clinical Oncology Clinical Practice Guideline Update on Chemotherapy for Stage IV Non-Small-Cell Lung Cancer. *J Oncol Pract.* **8(1):** 63–6.
- Chen Z, Cheng K, Walton Z *et al.* (2012). A murine lung cancer co-clinical trial identifies genetic modifiers of therapeutic response. *Nature* **483(7391):**613-7
- Chow LQM, Eckhardt SG, Reid JM *et al.* (2005). A first in human dose-ranging study to assess the pharmacokinetics, pharmacodynamics, and toxicities of the MEK inhibitor ARRY-142886 (AZD6244) in patients with advanced solid malignancies. AACR-NCI-EORTC International Conference on Molecular Targets and Cancer Therapeutics: Abstract C162.
- Dry JR, Pavey S, Pratilas CA *et al.* (2010). Transcriptional pathway signatures predict MEK addiction and response to selumetinib (AZD6244). *Cancer Res.* **70(6):**2264-73.
- Fossella FV, DeVore R, Kerr RN *et al.* (2000). Randomised Phase III Trial of Docetaxel Versus Vinorelbine or Ifosfamide in patients With Advanced Non-Small-Cell Lung Cancer Previously Treated With Platinum-Containing Chemotherapy Regimens. *J Clin Oncol* **18:**2354-62.
- Holt SV, Logié A, Odedra R *et al.* (2012). The MEK1/2 inhibitor, selumetinib (AZD6244; ARRY-142886), enhances anti-tumour efficacy when combined with conventional chemotherapeutic agents in human tumour xenograft models. *Br J Cancer* **106(5):**858-66.
- Jänne PA, Shaw AT, Pereira JR (2013). Selumetinib plus docetaxel for *KRAS*-mutant advanced non-small-cell lung cancer: a randomised, multicentre, placebo-controlled, phase 2 study. *Lancet Oncol.* **14(1):**38-47.
- Khushalani NI & Adjei AA. (2006). Novel Agents in the Treatment of Lung Cancer. *US Oncol Dis* **2:**24-8
- Ohashi K, Sequist LV, Arcila ME *et al.* (2013). Characteristics of Lung Cancers Harboring NRAS Mutations. *Clin Cancer Res.* **19(9):**2584-91.
- Pfister DG, Johnson DH, Azzoli CG *et al.* (2004). American Society of Clinical Oncology treatment of unresectable non-small-cell lung cancer guideline: update 2003. *J Clin Oncol* **22:**330-53.
- Shepherd FA, Dancey J, Ramlau R *et al.* (2000). Prospective Randomised Trial of Docetaxel Versus Best Supportive Care in Patients With Non-Small-Cell Lung Cancer Previously Treated With Platinum-Based Chemotherapy. *J Clin Oncol* **18:**2095-2103.
- Rubenstein *et al.* (2004), Clinical practice guidelines for the prevention and treatment of cancer therapy-induced oral and gastrointestinal mucositis. *Cancer* **100(9):**2026–46
- Peterson *et al.* (2010). Management of oral and gastrointestinal mucositis: ESMO Clinical Practice Guidelines. *Ann Oncol* **21(Suppl 5):**v261–v265.
- Evidence Based Practice Information Sheets for Health Professionals (1998). Prevention and Treatment Of Oral Mucositis In Cancer Patients, Best Practice 2(3):

27 ARM F: AZD5363 - AKT INHIBITOR (CLOSED TO RECRUITMENT)

Lead Investigator: Dr Timothy Yap

27.1 Background & Rationale

27.1.1 Molecular cohorts

Inhibitor: AZD5363

Arm	Investigational Medicinal Product	Cohort Number	NSCLC Histology	Molecular Cohort
	AZD5363 – AKT Inhibitor	**F1**	Squamous cell carcinoma (SCC)	PIK3CA mutation & no aberrations in KRAS, NF1, NRAS (Tiers 1-2), or HRAS or BRAF (Tier 3).
		**F2**	SCC	PIK3CA amplification & no aberrations in KRAS, NF1, NRAS (Tiers 1-2), or HRAS or BRAF (Tier 3) .
		**F3**	NSCLC	PIK3CA mutation or PIK3CA amplification & no aberrations in KRAS, NF1, NRAS (Tiers 1-2), or HRAS or BRAF (Tier 3) (Adenocarcinoma [ADC] or not otherwise specified [NOS] NSCLC);<br><br>PTEN mutation or PTEN loss & no aberrations in KRAS, NF1, NRAS (Tiers 1-2), or HRAS or BRAF (Tier 3) (ADC or NOS NSCLC);<br><br>AKT mutation & no aberrations in KRAS, NF1, NRAS (Tiers 1-2), or HRAS or BRAF (Tier 3) (NSCLC).
		**F4**	SCC	PTEN loss or PTEN mutation & no aberrations in KRAS, NF1, NRAS (Tiers 1-2), or HRAS or BRAF (Tier 3).

27.1.2 Pre-Clinical Data

AZD5363 is a highly potent ATP-competitive AKT inhibitor with IC_{50}<10 nmol/L for all three AKT isoforms (Davies *et al.* 2012). In a panel of 75 kinases, activity was demonstrated against a further 12 kinases (>75% inhibition at 1 µmol/L), 14 of which were members of the AGC kinase family (cAMP-dependent, cGMP-dependent and protein kinase C). The IC_{50} (concentration that results in 50% inhibition) for the Proline rich AKT substrate of 40kDa (PRAS40) and Glycogen Synthase Kinase 3β (GSK3β) inhibition in AKT activated cell lines was between 0.06 and 0.76 µmol/L. AZD5363 induced Forkhead box O3a (FOXO3a) nuclear translocation with an EC_{50} of 0.69 µmol/L. Using a cut off of GI_{50} < 3 µmol/L, 23% of a panel of 182 cell lines were sensitive to inhibition by AZD5363: 14% had a GI_{50} <1 µmol/L. 73% sensitive cell lines carried either PIK3CA mutations, PTEN loss or inactivation through mutation or HER2 amplification, and there was a significant relationship between PIK3CA mutation and PTEN inactivation (loss or mutation) and sensitivity. There was also a significant correlation between the presence of RAS mutations and resistance to AZD5363 and when the

cell lines with concomitant RAS mutations were excluded the relationships of PIK3CA mutation or PTEN inactivation and AZD5363 sensitivity became very highly significant.

Regression was seen in PIK3CA mutant and PTEN inactivated xenograft models in a number of tumour cell backgrounds. There was clear evidence of PD activity in these models with appropriate inhibition of downstream substrates: 50% inhibition of phosphorylated PRAS40 occurred at a total plasma exposure of approximately 0.1 µmol/L.

27.1.3 Clinical Data

Currently 5 AZ-sponsored Phase 1 and Phase 2 studies have been conducted or are ongoing. At the data cut-off of 04 October 2017, approximately 362 patients or healthy volunteers have been enrolled into the clinical development programme, of which 297 have received AZD5363 monotherapy, 38 have received AZD5363 in combination with paclitaxel, and 27 have received AZD5363 in combination with fulvestrant. In addition, 18 Investigator sponsored studies are planned or have recently commenced recruitment. Only limited data from these studies were available for inclusion in the Investigator's Brochure.

27.1.3.1 Safety

The following sections present the current safety data from the AZ-sponsored clinical studies, with all safety data up to the cut-off date of 04 October 2016 shown below. The data are pooled into 4 groups:

- Monotherapy continuous dosing: this group contains data from Studies D3610C00001 and D3610C00004.

- Monotherapy intermittent dosing: this group contains data from Studies D3610C00001 (Parts A, B, C, D)¸ D3610C00003, D3610C00004, and D3610C00007, and includes data from the 4 days on: 3 days off and 2 days on: 5 days off dosing schedules.

- Combination intermittent dosing with paclitaxel: this group contains data from Study D3610C00002 only, and includes dose schedules of 4 days on: 3 days off and 2 days on: 5 days off. Data from Part A, the combination dose-finding part of the study, is included. Part B, dose expansion, is ongoing and therefore data are not included here. An independent safety review committee has reviewed and continues to review safety data from the study in accordance with their charter.

- Combination intermittent dosing with fulvestrant: this group contains data from Study D3610C00001 Parts E and F on the 4 days on: 3 days off schedule.

See Table 19 of the IB for a summary of the design of each of these studies. For each pooled group, the greatest details is provided for the dose within each group that was concluded to be the recommended Phase 2 dose based on tolerability data from the completed studies:

- Monotherapy continuous dosing - AZD5363 320 mg BD (this was the MTD, and this dose regimen is not currently in ongoing clinical development at this time).

- Monotherapy intermittent dosing - AZD5363 480 mg BD (4 days on: 3 days off).

- Combination intermittent dosing with paclitaxel - AZD5363 400 mg BD (4 days on: 3 days off) plus paclitaxel as a weekly IV infusion of 90 mg/m^2 for 3 consecutive weeks followed by 1 week off.

- Combination intermittent dosing with fulvestrant – AZD5363 400 mg BD (4 days on: 3 days off) plus fulvestrant 500 mg intramuscularly (IM) on Days 1, 15, 29, and once monthly thereafter.

A summary of the key findings from these pooled analyses is as follows:

27.1.3.1.1 Monotherapy studies – intermittent dosing (N=229)

In the 480 mg BD intermittent dosing 4 days on; 3 days off (N=183), the most common AEs, irrespective of causality, were diarrhoea (146 [79.8%]), nausea (96 [52.5%]), hyperglycaemia (78 [42.6%]), fatigue (77 [42.1%]), vomiting (70 [38.3%]), decreased appetite (56 [30.6%]) and rash maculo-papular (54 [29.5%]). No obvious dose-related trends were seen for any of the common AEs in this pooled group. A total of 127 patients (69.4%) had AEs of CTCAE Grade 3 or above. AEs leading to discontinuation of AZD5363 treatment occurred in 31 patients (16.9%), 78 patients (42.6%) reported an SAE, and 7 patients died due to an AE (none of which were considered causally related to AZD5363 treatment).

27.1.3.1.2 Combination study with paclitaxel – intermittent dosing (N=38)

Overall (N=38), the most common AEs, irrespective of causality, were diarrhoea (35 [92.1%]), nausea (22 [57.9%]), asthenia (21 [55.3%]), vomiting (15 [39.5%]), anaemia (14 patients [36.8%]), decreased appetite (14 [36.8%]), neurotoxicity (14 [36.8%]), and alopecia (12 [31.6%]). A total of 22 patients had AEs of CTCAE Grade 3 or above. Two patients had an AE that led to discontinuation of AZD5363 treatment. Eleven patients (28.9%) had an SAE. There were no deaths. All patients had an AE considered by the investigator to be causally related to AZD5363 treatment. In the 400 mg BD plys paclitaxel 90 mg/m^2 combination intermittent dosing group (4 days on; 3 days off) (N=7), the most common AEs were diarrhoea (6 [85.7%]),asthenia (6 [85.7%]), neurotoxicity (3 [42.9%]), and myalgia (3 [42.9%]). There were no DLTs in this dosing group

27.1.3.1.3 Combination study with fulvestrant – intermittent dosing (N=26)

The only dosing schedule in this group was 400 mg BD 4 days on; 3 days off plus 500 mg fulvestrant. The most common AEs, irrespective of causality, were nausea (16 patients [61.5%]), diarrhoea (15 [57.7%]), and vomiting (7 [26.9%]). A total of 14 patients (53.8%) had AEs of CTCAE Grade 3 or above. Three patients (11.5%) had an AE that led to discontinuation of AZD5363 treatment. Ten patients (38.5%) had an SAE. There were no deaths. Nineteen patients (73.1%) had an AE considered to be causally related to AZD5363 treatment. The safety profile seen in the investigator sponsored studies is consistent.

27.1.3.1.4 Other Combinations

AZD5363 has also been evaluated in investigator sponsored studies in combination with docetaxel, olaparib, fulvestrant and enzalutamide. The AE profile in all these studies was consistent with the individual AE profiles of each drug and with no additional observations in relation to safety and tolerability.

27.1.3.2 Exposure

Up to the cut-off date of 04 October 2016, 362 patients have been dosed with AZD5363 in the 5 AZ-sponsored clinical trials that are included in the current IB. No exposure information is presented in the IB in relation to patients included in the investigator sponsored studies.

The pooled duration of treatment for key dose schedules are shown in Table 50.

Table 50: Duration of treatment for dosing groups of interest

	320 mg DB continuous[d] N=18	480 mg BD (4 on; 3 off)[a] N=183	400 mg BD (4 on; 3 off) + paclitaxel[b] N=7	400 mg BD (4 on; 3 off) + fulvestrant[c] N=26	Total N=234
**Days of study treatment:**					
**Mean (SD)**	51.8 (31.25)	120.8 (146.97)	217.1 (161.54)	76.3 (56.22)	113.4 (137.00)
**Median (range)**	46 (1 to 108)	81 (1 to 1456)	249 (18 to 477)	50 (15 to 233)	76 (1 to 1456)

[a] The 480 mg BD (4 days on; 3 days off) dose group includes data pooled from the following studies: Study 1 (Parts A, B, C, D), Study 3, Study 4, and Study 7.

[b] The 400 mg BD (4 days on; 3 days off) plus 90 mg/m2 paclitaxel dose group includes data from Study 2 only.

[c] The 400 mg BD (4 days on; 3 days off) plus 500 mg fulvestrant dose group includes data from Study 1 (Parts E, F) only.

[d] The 320 mg BD dose group includes data from Study 1 (Parts A and B) and Study 4.

The mean number of days on treatment for the monotherapy 320 mg BD continuous dosing group was 51.8 days, with a maximum time on treatment of 108 days (approximately 3.5 months) at the time of data cut-off. In the recommended dose schedule for monotherapy intermittent dosing of AZD5363 480 mg BD 4 days on; 3 days off, the mean number of days on treatment was 120.8 days, with a maximum time on treatment of 1456 days (approximately 4 years) at the time of data cut-off. In the recommended dose schedule for combination intermittent dosing with paclitaxel (AZD5363 400 mg BD 4 days on; 3 days off plus paclitaxel 90 mg/m^2), the mean number of days on treatment was 217.1 days, with a maximum time on treatment of 477 days (approximately 1 year and 3 months) at the time of data cut-off. In the recommended dose schedule for combination intermittent dosing with fulvestrant (AZD5363 400 mg BD 4 days on; 3 days off plus fulvestrant 500 mg), the mean number of days on treatment was 76.3 days, with a maximum time on treatment of 233 days (approximately 7.5 months) at the time of data cut-off.

27.1.3.3 Pharmacokinetics

PK data are available from Studies D3610C00001, D3610C00002, D3610C00003, D3610C00004 and D3610C00007. Study D3610C00001 and Study D3610C00004 are equivalent Phase 1 studies in advanced cancer patients performed in Western and Japanese patients, respectively. Study D3610C00002 is a Phase 1/2 study with a safety run-in exploring the combination of AZD5363 with paclitaxel in advanced and metastatic breast cancer and Study D3610C00003 a monotherapy study exploring AZD5363 in mCRPC patients. Study D3610C00007 is a clinical pharmacology study in advanced cancer patients to compare the PK exposure of a tablet formulation with the previously used capsule formulation and the effect of food on the PK of the tablet formulation. Single and multiple dose PK data are available

following BD administration of 80, 160, 240, 400, 480, 600, 640 and 800 mg doses in Study D3610C00001 and following 80, 240, 400 and 480 mg BD doses in Study D3610C00004, all administered as an oral capsule formulation. Multiple dose PK data are available following 480 mg BD dosing in Study D3610C00007.

27.1.3.4 Preliminary efficacy results

The majority of the AZ-sponsored studies reported in the IB have been primarily designed to determine the MTDs and recommended doses for Phase 2 for the various proposed AZD5363 dosing schedules. These studies to date have recruited patients whose malignancy has progressed despite receiving available standard therapies, thus they are from advanced cancer populations that have been heavily pre-treated and who show resistance to a number of prior therapies. Preliminary efficacy results at the time of the cut-off for the current IB are summarised below:

27.1.3.4.1 Studies D3610C00001 (Parts A and B) and D3610C00004:

Neither of these studies preselected for patients harbouring *PIK3CA* or *AKT* mutations. Tumour response data are available from 131 patients. The results show that 3 of the 131 patients had RECIST PRs: 1 whose tumour was positive for *PIK3CA* mutation and 2 where the tumour tested positive for *AKT1* mutation.

27.1.3.4.2 Study D3610C00001 (Parts C and D):

These expansion parts of the study involve an advanced cancer population selected on the basis of tumour molecular biology. In Part C, 31 patients with *PIK3CA*-mutant breast cancer received AZD5363. Of these, 28 had baseline target tumour lesions with measurable disease at baseline, and 1 had a RECIST response of PR in Part C (*PIK3CA* mutation). In addition, 28 patients with *PIK3CA*-mutant gynaecological cancer received AZD5363. Of these, 27 had baseline target tumour lesions and 2 had a RECIST response of PR. This part of the study is now closed to recruitment.

Part D has been closed due to lack of efficacy. Part E and Part F remain open to recruitment. Part E and Part F are designed to explore the combination of AZD5363 plus background fulvestrant in patients with ER positive breast cancer. The closure of Part D (AZD5363 monotherapy) is not considered to impact Parts E and F of Study D3610C00001 or other studies in the programme as Parts E and F test combination hypotheses that are different from the monotherapy used in Parts C and D of the study.

27.1.3.4.3 Study D3610C00007:

Of the 30 patients evaluable for response, no cases of CR or PR have been seen. Sixteen of the 30 patients (53.3%) maintained stable disease for more than 6 weeks. Four patients (13.3%) had disease control at 12 weeks.

27.1.3.5 Marketing Experience

AZD5363 has not been marketed in any country.

Further information on AZD5363 can be found in the IB.

27.1.4 Cohort Definition

The pre-clinical data clearly suggests that the optimal molecular cohorts of patients to test AZD5363 as a monotherapy are those with PIK3CA mutations or PTEN loss *without* concomitant KRAS mutations. In the most recent iteration of TCGA data 7/27

adenocarcinomas harbouring either PIK3CA, AKT or PTEN mutations have concomitant KRAS mutations but only 1/36 squamous cell cancers with such abnormalities have concomitant KRAS mutations. Thus, in squamous cell cancer where there is a particularly high frequency of PIK3CA/PTEN/AKT deregulation there is a very low frequency of concomitant KRAS mutation.

There appears to be a further difference in the presence of important potential resistance mechanisms in PI3K/AKT deregulated squamous cell carcinoma compared with adenocarcinoma. Over-expression of Serum/Glucocorticoid regulated Kinase 1 (SGK1), closely related to AKT and controlled by identical upstream molecules, is an important determinant of resistance to AZD5363 in breast cancer cells (Sommer *et al.* 2013). Interrogation of TCGA data reveals that 65% of lung adenocarcinoma with either PIK3CA, PTEN or AKT mutations over-express SGK1 compared with just 2/36 squamous cell cancers harbouring the same mutational spectrum (unpublished data).

Table 51 documents the frequency of abnormalities in the PI3K/PTEN/AKT pathway in NSCLC by histology. TCGA data is taken from the most recent iteration and includes validated cases not included in the published data.

Table 51: Frequency of abnormalities in the PI3K/PTEN/AKT pathway in NSCLC

	**TCGA Squamous (n=178)**	**TCGA Adeno (n=392)**	**Broad (Imielinski *et al.* 2012) Adeno (n=183)**	**GMI (Seoul) (Seo *et al.* 2012) Adeno (n=87)**
**AKT1 mut**	0.6%	0.3%		
**PIK3CA mut**	15.2% (7.3)*	4.6% (1.5)*	5%	3.6%
**PTEN mut**	7.9% (4.5)*	2% (0.8)*	2.7%	
**PIK3CA amp**	7%	2.4%		
**PTEN inactive**	15%			

**Figures in parenthesis represent the frequency of hotspot mutations.*

It will be appreciated that PIK3CA amplification is a further significant cohort. In other studies the rate of amplification by FISH in squamous cancer is as high as 37% (cf 5% in adenocarcinoma) (Spoerke *et al.* 2012). In this study the impact of PI3K inhibition (using GDC-0941) has been specifically interrogated in PIK3CA amplified NSCLC cell lines: all 3 lines tested were highly sensitive to growth inhibition. Interestingly 2/3 lines harboured concomitant PTEN loss. Cell lines with PTEN loss alone were less sensitive to growth inhibition. There was no co-occurrence of key oncogenic mutations in this series or the TCGA. Importantly, there was discordance between PI3K copy number in primary and metastasis suggesting contemporary biopsy is important in assessing PI3K copy number.

Given the relatively small numbers of patients harbouring the various molecular causes of PI3K/AKT deregulation in adenocarcinoma and the presence of concomitant KRAS mutation in a significant minority it is proposed that we treat a single cohort of KRAS wild type patients with PIK3CA/AKT deregulation and we aim to retrospectively assess the negative predictive value SGK1 status by assessment of pNDRG1 by IHC (in conjunction with Dario Alessi). The size of each discrete squamous cell carcinoma PI3K/PTEN/AKT deregulated cohort allows us to run individual cohorts of each abnormality. This will be much more informative than pooling the various abnormalities in a single cohort as response to AKT inhibition may vary according to the mechanism of pathway deregulation and the mechanisms of resistance are also likely

to vary. These subtle but potentially important clinical differences may be lost in a pooled cohort, with each pathway to deregulation may be numerically under-represented. We will pool the very rare AKT1 mutants in a single cohort.

27.2 Specific Eligibility Criteria

27.2.1 Inclusion Criteria

For inclusion in the study, patients must fulfil all of the following criteria:

- Patients must fulfil all the core eligibility criteria.
- Technology hub result (or locally obtained result from an approved Laboratory is applicable.
- Eastern Cooperative Oncology Group (ECOG) Performance Status ≤2 with no deterioration over the previous 2 weeks (see Appendix 8: Eastern Cooperative Oncology Group Performance Status Criteria).
- For patients with EGFR wt tumours, prior therapy must include platinum- based chemotherapy. For patients with tumours harbouring an EGFR mutation known to be associated with EGFR TKI sensitivity, prior therapy must include an EGFR tyrosine kinase inhibitor. For patients with anaplastic lymphoma kinase (ALK)-positive tumours, prior therapy must include an ALK inhibitor.

27.2.2 Exclusion Criteria

Patients must not enter the trial if any of the following exclusion criteria are fulfilled:

- Patients who do not fulfil all the core eligibility criteria.
- The SMP2 panel shows no result available, or confirmation of any concomitant genetic changes in KRAS, NFI, or NRAS (Tiers 1-2), or HRAS or BRAF (Tier 3).
- Treatment with any of the following:
    - Any concomitant medication known to prolong the QT interval within 6 half-lives plus 1 day prior to the first dose of AZD4547 (see Section 27.2.3).
    - Nitrosourea or mitomycin C within 6 weeks of the first dose of AZD5363.
    - Strong inhibitors, potent inducers or substrates of CYP3A4, or substrates of CYP2D6 within the required washout period prior to the first dose of AZD5363:
        - CYP3A4 inhibitors: see Table 52 in Section 27.2.3.2 for prohibited medications and relevant washout periods (see Table 54 for moderate inhibitors permitted with caution)
        - CYP3A4 inducers: see Table 53 in Section 27.2.3.2 for prohibited medications and relevant washout periods
        - CYP3A4 substrates: see Table 55 & Table 59 in Section 27.2.3.2 for prohibited medications and relevant washout periods (see Table 56 for substrates permitted with caution)
        - CYP2D6 substrates: see Table 57 & Table 59 in Section 27.2.3.2 for prohibited medications and relevant washout periods (see Table 58 for substrates permitted with caution)
    - AZD5363 treatment in any other study i.e. ISS or AZ sponsored study (i.e. any dosing with AZD5363 due to previous participation in any other study).
    - Pathway inhibitors as follows:
        - PIK3CA mutant, PIK3CA amplified and PI3K/AKT deregulated (cohorts F1, F2 & F3) - inhibitors with PI3K and AKT pharmacology.
        - PTEN mutant, PTEN loss, AKT1 mutant (F3 & F4) – inhibitors with AKT pharmacology.
    - Radiotherapy within 4 weeks prior to the first dose of AZD5363.

    - Any other chemotherapy, investigational agents or other anti-cancer therapy within 4 weeks prior to the first dose of AZD5363.
- Clinically significant abnormalities of glucose metabolism as defined by any of the following:
    - Diagnosis of diabetes mellitus type I.
    - Fasting plasma glucose (fasting is defined as no caloric intake for at least 8 hours):
        - ≥7 mmol/L (126 mg/dL) at screening for patients without a pre-existing diagnosis of Type 2 diabetes mellitus.
        - ≥9.3 mmol/L (≥167 mg/dL) for those patients with a pre-existing diagnosis of Type 2 diabetes mellitus.
    - Glycosylated haemoglobin (HbA1C) ≥8.0% (63.9 mmol/mol) at screening (conversion equation for HbA1C [IFCC-HbA1C (mmol/mol) = [DCCT-HbA1C (%) – 2.15] x 10.929).
    - Requirement for insulin for routine diabetic management and control.
    - Requirement for more than two oral hypoglycaemia medications for routine diabetic management and control.
- Any of the following cardiac criteria:
    - Mean resting corrected QT interval (QTcF) >470 msec obtained from 3 consecutive ECGs.
    - Any clinically important abnormalities in rhythm, conduction or morphology of resting ECG e.g., complete left bundle branch block, third degree heart block.
    - Any factors that increase the risk of QTc prolongation or risk of arrhythmic events such as heart failure, hypokalaemia, potential for torsades de pointes, congenital long QT syndrome, family history of long QT syndrome or unexplained sudden death under 40 years of age.
    - Experience of any of the following procedures or conditions in the preceding 6 months: coronary artery bypass graft, angioplasty, vascular stent, myocardial infarction, angina pectoris, congestive heart failure NYHA Grade ≥2.
    - Uncontrolled hypotension – SBP <90 mmHg and/or DBP <50 mmHg.
    - Left ventricular ejection fraction (LVEF) below lower limit of normal for site.
- Hepatic function (in patients **with** liver metastasis).
    - Alanine transferase (ALT) and Aspartate transferase (AST) >5 x ULN.
- Proteinuria 3+ on dipstick analysis followed by >500 mg/24 hours.
- Screening sodium or potassium outside normal reference range for site
- History of hypersensitivity to active or inactive excipients of AZD5363 or drugs with a similar chemical structure or class to AZD5363.

27.2.3 Restrictions & Concomitant Medications

Information on any treatment from the date of informed consent until 28 days after the administration of the last treatment dose should be recorded. If medically feasible, patients taking regular medication should be maintained on it throughout the study period. Patient should be advised to inform their treating physicians of all concomitant medications, including prescription medicines, over-the-counter drugs, vitamins, and herbal products.

Nb. These lists are not exhaustive and the absence of a drug from the lists does not imply that its combination with AZD5363 is safe.

27.2.3.1 QT Interval Prolongation

Concomitant medications **known to prolong the QT interval** are prohibited during the active treatment phase. Any patients taking such drugs at or prior to registration should discontinue the drug 6 half-lives plus 1 day prior to commencing trial treatment.

For a list of drugs known to prolong the QT interval, (or with any risk of prolonging the QT interval), please refer to the following database: https://www.crediblemeds.org. Appendix 9 Credible Meds List of Drugs that Prolong QT Interval contains a list exported from this database on 2nd March 2018. **It is important to note that this list is a guide – the database will change with time and therefore needs to be checked in real-time when screening and registering a patient, and throughout their treatment. The Trials Office will email all sites when updates are made to the database.**

Please note that for AstraZeneca trial Arms, only concomitant medications under the category **'Known risk of TdP'** will exclude patients from entering the trial as per the eligibility criteria.

It is the responsibility of an Investigator (Consultant level) to review and clinically evaluate all concomitant medications. Please contact the National Lung Matrix Trial Office for clarification regarding any drugs that appear on the database that are not listed in Appendix 9 Credible Meds List of Drugs that Prolong QT Interval.

The drugs listed on this website are taken from information provided by The Arizona Centre for Education and Research on Therapeutics and The Critical Path Institute, Tucson, Arizona and Rockville, Maryland. Important Note - If a patient is being treated with such medication or is taking another medication that may affect QT interval which is not on the database, please contact the National Lung Matrix Trial Office to obtain the recommended withdrawal/ minimum period prior to starting trial treatment.

27.2.3.2 Cytochrome P450 (CYP) Related Induction and Inhibition

In vitro experiments indicate that AZD5363 is a time-dependent inhibitor of CYP3A4, which may result in increased exposure of drugs metabolized via CYP3A4 and with the potential to increase the toxicity of these drugs when co-administered with AZD5363. AZD5363 is itself a substrate of CYP3A4 although data available to date suggests that glucuronidation may be the major metabolic route. Co-administration of CYP3A4 inhibitors may increase exposure to AZD5363 and hence potentially affect efficacy/toxicity and hence increase the risk of time-dependent inhibition (and resultant toxicity of CYP3A4 substrates). In addition, co-administration of CYP3A4 inducers may decrease the exposure to AZD5363 and potentially affect efficacy. AZD5363 is also a moderate inhibitor of CYP2D6 *in vitro*. This may increase the exposure of drugs metabolized via CYP2D6 with the potential to increase the toxicity of these drugs when co-administered.

The following restrictions will therefore be put in place in the trial, please see the tables below for listings of relevant drugs. If co-administration is necessary for appropriate clinical care (see Table 54, Table 56 & Table 58 for medications permitted with caution) then additional monitoring for signs of toxicity related to increased exposure to the substrates is required:

27.2.3.2.1 Drugs affecting CYP3A4 metabolism that AstraZeneca strongly recommend are not combined with AZD5363

There are currently no data confirming that there are any PK interactions between any agents and AZD5363. The potential interactions detailed below are considered on the basis of the preclinical data only. The following lists are not intended to be exhaustive, and a similar restriction will apply to other agents that are known to strongly modulate CYP3A4 activity. Appropriate medical judgment is required. Please contact the National Lung Matrix Trial Office with any queries you have on this issue.

Table 52: Prohibited strong CYP3A4 inhibitors may increase exposure to AZD5363 more than 5-fold

Drug	Minimum washout period prior to AZD5363 treatment
Ketoconazole<br>Protease inhibitors (Danoprevir, Ritonavir, Saquinavir, Indanavir, Tapranavir, Telaprevir, Elvitegravir, Lopinavir, Nelfinavir, Bocepravir)<br>Cobicistat<br>ConivaptanNefazodone<br>Mebefradil	Minimum of 2 weeks washout prior to AZD5363 administration and for 2 weeks following discontinuation of AZD5363
Itraconazole<br>Posaconazole<br>Voriconazole<br>Clarithromycin<br>Telithromycin<br>Troleandomycin	Minimum of 1 week washout prior to AZD5363 administration and for 2 weeks following discontinuation of AZD5363

Table 53: Prohibited potent Inducers of CYP3A4 that may reduce exposure to AZD5363 by more than 5-fold

Drug	Minimum washout period prior to AZD5363 treatment
Phenobarbital<br>Carbamazepine<br>Phenytoin<br>Rifampicin<br>Rifabutin<br>Mitotane<br>Enzalutamide	Minimum of 2 weeks washout prior to AZD5363 administration and for 2 weeks following discontinuation of AZD5363
St John's Wort	Minimum of 3 weeks washout prior to AZD5363 administration and for 2 weeks following discontinuation of AZD5363

27.2.3.2.2 Drugs affecting CYP3A4 metabolism that AstraZeneca considers may be allowed with caution.

Table 54: Permitted (with caution) moderate Inhibitors of CYP3A4 that may increase exposure to AZD5363

Drug	Guidance
DiltiazemVerapamil<br>Erythromycin<br>Fluconazole<br>Aprepitant	Drugs are permitted but caution should be exercised and patients monitored closely for possible drug interactions. Please refer to full prescribing information for all drugs prior to co-administration with AZD5363.

Drug	Guidance
Grapefruit juice<br>Seville oranges (and other products containing Seville oranges)	Patients should abstain from eating large amounts of grapefruit and Seville oranges (and other products containing these fruits eg, grapefruit juice or marmalade) during the study (e.g., no more than a small glass of grapefruit juice (120 mL) or half a grapefruit or 1-2 teaspoons (15 g) of Seville orange marmalade daily).

27.2.3.2.3 Medicines that are significantly metabolised by CYP3A4 (substrates) that AstraZeneca strongly recommend are not combined with AZD5363

There are currently no data confirming that there are any pharmacokinetic (PK) interactions between AZD5363 and the following CYP3A4 substrates. The potential interactions detailed below are considered on the basis of the preclinical data only. The following list is not intended to be exhaustive, and a similar restriction will apply to other agents that are known to be sensitive to CYP3A4 inhibitors. Appropriate medical judgment is required. Please contact the National Lung Matrix Trial Office with any queries you have on this issue.

Table 55: Prohibited medicines significantly metabolised by CYP3A4 (substrates)

Exposure, pharmacological action and toxicity that may be increased by inhibition of CYP3A4 by AZD5363.

Drug	Minimum washout period prior to AZD5363 treatment
Alfentanil<br>Cyclosporin<br>Diergotamine<br>Ergotamine<br>Fentanyl<br>Sirolimus<br>Tacrolimus<br>Atorvastatin<br>Lovastatin<br>Simvastatin<br>Cerivastatin	Minimum of 1 week washout prior to AZD5363 administration and for 2 weeks following discontinuation of AZD5363
Carbamazepine	Minimum of 2 weeks washout prior to AZD5363 administration and for 2 weeks following discontinuation of AZD5363.

27.2.3.2.4 Medicines that are significantly metabolised by CYP3A4 (substrates) that AstraZeneca considers may be allowed with caution

Table 56: Permitted (with caution) medicines metabolised by CYP3A4 (substrates)

Exposure, pharmacological action and toxicity that may be increased by inhibition of CYP3A4 by AZD5363

Drug	Guidance
Erythromycin<br>Trazodone<br>Tamoxifen<br>Alprazolam<br>Midazolam<br>Triazolam<br>Felodipine<br>Isradipine<br>Nifedipine<br>Methylprednisolone<br>Pimozide<br>Quinidine<br>Domperidone	Drugs are permitted but caution should be exercised and patients monitored closely for possible drug interactions. Please refer to full prescribing information for all drugs prior to co-administration with AZD5363.

27.2.3.2.5 Agents that are sensitive to CYP2D6 inhibition (substrates) that AstraZeneca strongly recommends are not combined with AZD5363

There are currently no data confirming that there are any PK interactions between AZD5363 and the following CYP2D6 substrates. The potential interactions detailed below are considered on the basis of the preclinical data only. This list is not intended to be exhaustive, and a similar restriction will apply to other agents that are known to be sensitive to CYP2D6 inhibitors. Appropriate medical judgment is required. Please contact the National Lung Matrix Trial Office with any queries you have on this issue.

Table 57: Prohibited medicines metabolised by CYP2D6 (substrates)

Exposure, pharmacological action and toxicity that may be increased by inhibition of CYP2D6 by AZD5363

Drug	Minimum washout period prior to AZD5363 treatment
Amitryptylline<br>Desipramine<br>Trimipramine<br>Doxepin	Minimum of 2 weeks washout prior to AZD5363 administration and for 2 weeks following discontinuation of AZD5363

Drug	Minimum washout period prior to AZD5363 treatment
Atomoxetine<br>Metoprolol<br>Nefazodone<br>Nebivolol<br>Perphenazine<br>Tropisetron<br>Tolterodine	Minimum of 1 week washout prior to AZD5363 administration and for 2 weeks following discontinuation of AZD5363

27.2.3.2.6 Agents that are sensitive to CYP2D6 inhibition that AstraZeneca considers may be allowed with caution

Table 58: Permitted (with caution) medicines metabolised by CYP2D6 (substrates)

Exposure, pharmacological action and toxicity that may be increased by inhibition of CYP2D6 by AZD5363

Drug	Guidance
Venlafaxine<br>Paroxetine<br>Fluoxetine	Drugs are permitted but caution should be exercised and patients monitored closely for possible drug interactions. Please refer to full prescribing information for all drugs prior to co-administration with AZD5363.

27.2.3.2.7 Agents that are sensitive to combined CYP3A4 and CYP2D6 inhibition that AstraZeneca strongly recommend are not combined with AZD5363

There are currently no data confirming that there is a pharmacokinetic (PK) interaction between AZD5363 and the following agents; a potential interaction is considered on the basis of the preclinical data only. This list is not intended to be exhaustive, and a similar restriction will apply to other agents with narrow therapeutic windows that are known to depend on combined CYP3A4 and CYP2D6 metabolism. Appropriate medical judgement is required. Please contact the National Lung Matrix Trial Office with any queries you have on this issue.

Table 59: Prohibited medicines metabolised by combined CYP3A4 and CYP2D6 (substrates)

Exposure, pharmacological action and toxicity that may be increased by inhibition of CYP3A4 and CYP2D6 by AZD5363

Drug	Minimum washout period prior to AZD5363 treatment
Haloperidol	Minimum of 2 weeks washout prior to AZD5363 administration and for 2 weeks following discontinuation of AZD5363
Tramadol	Minimum of 1 week washout prior to AZD5363 administration and for 2 weeks following discontinuation of AZD5363

27.2.3.2.8 Guidance for drugs that are significantly metabolised by CYP2B6, CYP2C9 or CYP2C19 and have a narrow therapeutic margin that AstraZeneca considers may be allowed with caution

Weak signals for competitive inhibition of CYP2B6, CYP2C9 and CYP2C19 cytochrome P450 activities have been demonstrated by *in vitro* laboratory investigations. There are currently no data confirming that there is a pharmacokinetic (PK) interaction between AZD5363 and substrates of these isoforms; a potential interaction is considered on the basis of the preclinical data only. The following list is intended to identify known sensitive substrates of CYP2B6, CYP 2C9 and CYP 2C19 that have a narrow therapeutic margin. The list is not intended to be exhaustive, and a similar restriction should be applied to any other sensitive substrate with narrow therapeutic margin. Appropriate medical judgment is required. Please contact the National Lung Matrix Trial Office with any queries you have on this issue.

Table 60: Permitted (with caution) medicines that are known sensitive substrates of CYP2B6, CYP 2C9 and CYP 2C19

Drug	Guidance
<u>CYP26B</u><br>Bupropion	Drugs are permitted but caution should be exercised and patients monitored closely for possible drug interactions. Please refer to full prescribing information for all drugs prior to co-administration with AZD5363.
<u>CYP2C9</u><br>Warfarin	
<u>CYP2C19</u><br>Clobazam	

27.2.3.3 Metformin

AZD5363 is an in vitro inhibitor of the renal transporters OCT2 and MATE1, which are significantly involved in metformin excretion and partially responsible for creatinine clearance. Metformin is recommended for the management of hyperglycaemia occurring in patients participating in studies of AZD5363. Investigators should exercise caution in the dosing and management of patients receiving the metformin/AZD5363 combination and must be vigilant for signs of renal impairment and metformin toxicity, such as lactic acidosis and hypoglycaemia, namely: lethargy, hypotension, poor urine output, drowsiness, irritation, tachypnoea, sweating, diarrhoea, and vomiting. Metformin should only be given on the days when AZD5363 is also given (the half-life of AZD5363 is approximately 8 to 15 hours), and should be withdrawn when treatment with AZD5363 is withdrawn, unless otherwise clinically indicated. Due to the potential interaction of metformin and AZD5363 due to inhibition of OCT2, patients should attend for clinical assessment when taking both AZD5363 and metformin concurrently, including monitoring of serum creatinine at least once per week for the first 3 weeks after initiation of metformin, then every 3 weeks thereafter.

27.2.3.4 Statin Guidance

27.2.3.4.1 Guidance for statins that are metabolised by CYP3A4 that AstraZeneca considers may be allowed with caution

In vitro data has shown that AZD5363 has a potential to inhibit the OATP1B1 and OATP1B3 transporters. These transporters are implicated in the distribution and clearance of many of the statins. Of the statins that are minimally affected by CYP3A4 inhibition, rosuvastatin and pravastatin (but not fluvastatin) can be affected by OATP1B1/OATP1B3 inhibition. In an assessment of the potential for AZD5363 to inhibit these transporters based on the in vitro

signal, the AUC of these drugs may be increased by 1.3-fold for pravastatin and 1.5-fold for rosuvastatin (static assessment based on maximal free liver inlet concentration of AZD5363).

As a conservative response to this emerging data it is recommended that one of the following options are used for patients who need treatment with a statin whilst receiving AZD5363, and doses of rosuvastatin and pravastatin are capped as follows when combined with AZD5363, and for a 2-week period before and after AZD5363 treatment:

- Rosuvastatin (up to 10 mg once daily).
- Pravastatin (up to 40 mg once daily).
- Fluvastatin

Time-dependent inhibition of cytochrome P450 (CYP) 3A4/5 was observed during the non-clinical *in vitro* evaluation of the metabolism of AZD5363.

The CYP3A4 isozyme is responsible for the metabolism of atorvastatin (ATV), cerivastatin (CRV), lovastatin (LOV), and simvastatin (SMV), including combinations with ezetimibe (SMV/ezetimibe [SMV/EZE]), and their exposure, pharmacological action and toxicity may increase by inhibition of CYP 3A4 and the potential for CYP-mediated Drug-Drug interactions (DDIs) is high.
However, there is minimal metabolism of fluvastatin (FLV), pravastatin (PRV), or rosuvastatin (RSV) by CYP3A4 thus plasma levels are minimally influenced by CYP3A4 inhibitors, conveying a relatively low potential for clinically significant DDIs via this mechanism.

Emerging *in vitro* data has revealed that AZD5363 has a potential to inhibit the OATP1B1 transporter. This transporter is implicated in the distribution and clearance of many of the statins. Of the statins that are minimally affected by CYP3A4 inhibition, RSV and PRV (but not FLV) can be affected by OATP1B1 inhibition. Based on an assessment of the potential for AZD5363 to inhibit OATP1B1 based on the *in vitro* signal the AUC of these drugs may be increased by 1.3-fold for PRV and 1.5-fold for RSV (static assessment based on maximal free liver inlet concentration of AZD5363). As a conservative response to this emerging data it is recommended that doses of RSV be capped to 10 mg once daily and PRV be capped to 40 mg once daily when combined with AZD5363, and for a 2 week period before and after AZD5363 treatment.

In summary, RSV (up to 10 mg once daily), PRV (up to 40 mg once daily) and FLV are appropriate agents to be used in patients included in AZD5363 studies who require statin therapy.

27.2.3.5 Concomitant Radiotherapy

Radiation for palliation at focal sites is permitted. The length of wash out before and after focal radiotherapy is at the discretion of the treating physician and the local radiotherapy team based on the individual patient, the radiotherapy plan, and the tolerability experienced during the study. The available data from the AZ studies so far does not suggest a requirement for interruption of AZD5363 for radiotherapy treatment but this analysis is preliminary and ongoing. Guidance is to consider allowing a wash out of 5 half-lives (3 days) pre and post procedure, and to monitor general tolerability and AEs.

27.2.3.6 Food Restrictions

Patients should abstain from eating large amounts of grapefruit and Seville oranges (and other products containing these fruits eg, grapefruit juice or marmalade) during the study (e.g., no more than a small glass of grapefruit juice (120 mL) or half a grapefruit or 1-2 teaspoons (15 g) of Seville orange marmalade daily).

27.2.3.7 Other Interactions

- Inhibition by AZD5363 was observed *in vitro* for UGT1A1, indicating that there is a potential for absorption-related drug-drug interaction with substrates of UGT1A1 when considering intestinal, but not hepatic concentrations of AZD5363. These drugs should be administered with caution.
- AZD5363 is also a substrate for the P-gp transporter in vitro. There is a potential for increased systemic exposures of AZD5363 by potent P-gp inhibitors. These drugs should also be administered with caution.
- Treatment with St John's Wort is prohibited during treatment with AZD5363. See Table 53 in Section 27.2.3.2.1 for pre-treatment washout period.

27.2.3.8 Contraception

Reproductive toxicology studies have been completed, including an assessment of male fertility in the 6-month rat toxicity study, and embryofoetal development and early postnatal survival/growth in rats. Although AZD5363 causes testicular pathology in rats and dogs, there was no effect on male fertility in the 6-month rat study. AZD5363 had an adverse effect on embryonic survival and early postnatal growth when administered to pregnant rats. Exposure to AZD5363 was confirmed in suckling pups, which may indicate the potential for excretion of AZD5363 in milk.

See Section 6.3 for a definition of females of childbearing potential and females of non-childbearing potential. Females of child-bearing potential should use two forms of highly reliable methods of contraception from the time of screening until at least 4 weeks after discontinuing trial treatment. Acceptable methods of contraception include:

- Established use of oral, injected or implanted hormonal methods of contraception.
- Placement of an intrauterine device or intrauterine system.
- Barrier methods of contraception: condom or occlusive cap (diaphragm or cervical/vault caps) with spermicidal foam/gel/film/cream/suppository.
- Male partner sterilisation (with the appropriate post-vasectomy documentation of the absence of sperm in the ejaculate).
- True abstinence.
- It is not known whether AZD5363 has the capacity to affect the metabolism of hormonal contraceptives, so hormonal contraception should also be combined with a barrier method of contraception.

Male patients should use barrier contraception (i.e. condoms) and refrain from sperm donation throughout the trial and for 16 weeks after the last dose of study drug. Male patients wishing to father children should be advised to arrange for freezing of sperm samples prior to the start of trial treatment.

27.3 Trial Treatment

27.3.1 Investigational Medicinal Product

AZD5363 is available for administration as a tablet for use in clinical trials. The tablet is presented 160 and 200 mg strength tablets. AZD5363 will be provided by AstraZeneca free of charge.

AZD5363 is a potent, selective inhibitor of the kinase activity of the serine/threonine kinase AKT 1, 2 and 3 that is being developed as a potential treatment for solid and haematological malignancies.

AZD5363 will be administered orally, twice daily, on an intermittent weekly dosing schedule. The dosing schedule will be intermittent dosing, 4 days on, 3 days off, 480 mg bd. Continuous dosing of AZD5363 has not been taken forward as it is not tolerable at biologically active doses.

Where possible, all doses of AZD5363 should be taken at approximately the same times each day, with water, and in a fasted state from at least 2 h before dosing to 1 h after dosing.

In the event that a patient vomits, the patient must not retake new tablet(s), but continue to take the next dose 12 hours later.

Should a patient miss a scheduled dose, the patient will be allowed to take the dose up to a maximum of 2 hours after the scheduled dose time. If greater than 2 hours after the scheduled dose time the missed dose should not be taken and the patient should take their allotted dose at the next scheduled time. If a patient needs to take the dose earlier for whatever reason, the patient can take the dose up to 2 hours earlier than the scheduled dose time. The patient should make every reasonable effort to take the AZD5363 tablet(s) on time. Both the Arm F Patient Information Sheet and Arm F Patient Diary contain more specific instructions for patients to follow regarding how to take their medication

Please note patients who meet RECIST criteria for progressive disease (PD) may be continued on trial treatment if the treatment is tolerable and the Investigator believes it to be of clinical benefit; see Section 9.3.

Please also refer to the Pharmacy Manual for further details.

27.3.2 Schedule of Assessments

Table 61: AZD5363 - Schedule of Assessments

	Screening	Treatment - 480 mg twice-daily dose Intermittent 4 days on 3 days off (28 Day Cycles)					Discontinuation (+ 7 days)**	28-day Follow Up[v] (+ 7 days)**	Survival Assessment (± 7 days)***
	Within 28 days of treatment (unless otherwise stated)	Cycle 1				Cycle 2 onwards			
		Day 1	Day 2	Day 4	Day 11	Day 1 (± 2 days)*			
Informed consent[a]	X								
Demography & baseline characteristics[b]	X								
Medical history[c]	X								
Inclusion / exclusion criteria[d]	X								
Physical examination[e]	X	X		X	X	X	X		
ECOG performance status	X (within 14 days of treatment)	X				X	X		
Vital signs (inc. weight)[f]	X	X	X	X	X	X	X		
ECG[g]	X	X	X	X	X	X	X		
MUGA / ECHO[h]	X					X (every 12 weeks ± 7 days)			

	Screening	Treatment - 480 mg twice-daily dose<br>Intermittent 4 days on 3 days off (28 Day Cycles)					Discontinuation<br>(+ 7 days)**	28-day Follow Up[v]<br>(+ 7 days)**	Survival Assessment<br>(± 7 days)***
	Within 28 days of treatment (unless otherwise stated)	Cycle 1				Cycle 2 onwards			
		Day 1	Day 2	Day 4	Day 11	Day 1<br>(± 2 days)*			
Haematology, Clinical chemistry, Urinalysis[i]	X<br>(within 7 days of treatment)	X		X	X	X	X		
Fasted Lipids[j]		X				X (every 6th cycle)	X		
Glucose and Insulin[k]	X (fasted)	X	X	X<br>(glucose only)	X<br>(glucose only)	X			
Glycosylated haemoglobin (HbA1c)[l]	X					X (every 3rd cycle)		X	
Pregnancy test[m]	X	X					X		
Tumour assessments[n]	X	Every 6 weeks during year 1 (± 7 days) [except 1st scan + 7days only]							X□
Adverse events & Concomitant Medications	X	X	X	X	X	X	X	X	
Dispense study drug[o]		X<br>(-2 days)				X<br>(-2 days)			
Administer study drug[p]		X	X	X	X	X			
Smoking status[q]		X				X (every other cycle beginning cycle 3)	X		

	Screening	Treatment - 480 mg twice-daily dose Intermittent 4 days on 3 days off (28 Day Cycles)					Discontinuation (+ 7 days)**	28-day Follow Up[v] (+ 7 days)**	Survival Assessment (± 7 days)***
	Within 28 days of treatment (unless otherwise stated)	Cycle 1				Cycle 2 onwards			
		Day 1	Day 2	Day 4	Day 11	Day 1 (± 2 days)*			
Germline DNA sample[r]		X							
ctDNA samples[s]		X				X (every other cycle beginning cycle 3) (- 2 days)	X		X $
Optional research treatment biopsy[t]		X (post-reg, pre-tx)					X		
Survival status[u]									X

* Visit may occur ± 2 days of the planned visit date. Individual assessments may occur independently of the visit date where indicated in the table Above

** Visit may occur + 7 days of the planned visit date

*** Visit may occur ± 7 days of the planned visit date

a Prior to the start of any study specific procedures, each patient must provide signed informed consent.

b Demography must be captured for all patients, including screen failures. Demographic data and other characteristics will include: date of birth, gender, race and ethnicity.

c A standard medical and surgical history will be obtained, including prior cancer treatment and smoking status.

d Patients must not be registered unless all eligibility criteria have been fully met.

e Physical examination includes general appearance, respiratory, cardiovascular, skin, head and neck (including ears, eyes, nose and throat), lymph nodes, thyroid, abdomen, musculo-skeletal (including spine and extremities) and neurological systems and should be performed at screening, pre-dose on Cycle 1 Days 1, 4 and 11. Physical examination on Day 1 of subsequent cycles and discontinuation can be performed at any time of day.

f Vitals signs are to be recorded are height (at screening only), weight, BP and pulse; required at screening, pre-dose on day 1 of every Cycle and at

Discontinuation.

Single measurements of supine blood pressure and pulse will be recorded on each occasion after 10 minutes rest. Vital signs are to be taken pre-dose and 1 hour, 2 hours and 4-6 hours post dose on Cycle 1 Day 1, pre-dose on Cycle 1 Days 2, 4 and 11, pre-dose and 2 hours post dose on Day 1 of subsequent Cycles. Vital signs can be taken at any time of the day at the discontinuation visit.

g 12-lead ECG to be taken at screening; pre-dose and 1 hour, 2 hours and 4-6 hours post dose on Cycle 1 Day 1; pre-dose on Cycle 1 Days 2, 4 and 11; pre-dose and 2 hours post dose on Cycle 2 Day 1. ECG performed on Day 1 of subsequent cycles and Discontinuation can be performed at any time of day.

Twelve-lead ECGs will be obtained after the patient has been resting semi-supine for at least 10 minutes prior to times indicated. All ECGs should be recorded with the patient in the same physical position. For each time point unless specifically stated above, three ECG recordings should be taken at a minimum of 5 minute intervals (all three ECGs must be collected within 30 minutes). A standardised ECG machine should be used and the patient should be examined using the same machine throughout the study if possible. After paper ECGs have been recorded, the Investigator or designated physician will review each of the ECGs and may refer to a local cardiologist if appropriate. A paper copy should be filed in the patient's medical records. If an abnormal ECG finding at screening or Cycle 1 day 1 is considered to be clinically significant by the Investigator, it should be reported as a concurrent condition. For all ECGs details of rhythm, ECG intervals (R-R, PR, QT and QRS) and an overall evaluation will be recorded.

h A scan carried out within 3 months prior to starting study treatment can be used for eligibility at screening, however, for patients who experience a significant cardiac event or who have had cardiotoxic medication within this interval, the MUGA/ECHO scan should be repeated prior to starting study treatment. MUGA/ECHO should be performed on Cycle 2 day 1 (± 7 days) then every 12 weeks - after approximately completion of every 3 cycles until discontinuation and as clinically indicated. The modality of the cardiac function assessments must be consistent within patient and the same machine operator is to be used where possible.

i Clinical Chemistry, Haematology and Urinalysis to be performed at screening, **pre-dose** whilst on treatment (see table for specific timepoints) and at discontinuation. Discontinuation samples can be collected at any time of the day.

Clinical Chemistry: Albumin, AST, ALT, alkaline phosphatase, bilirubin (total), calcium (total), creatinine, glucose, magnesium, phosphate, sodium, troponin I or T, urea nitrogen, oestradiol (females only), testosterone (males only), potassium, follicule stimulating hormone (FSH), thyroid stimulating hormone (TSH), Free T4 and total protein.

Liver biochemistry results must be reviewed by the Investigator prior to first dose on Cycle 1 day 1, but may be taken up to 48 hours in advance. Laboratory tests do not need to be repeated at Cycle 1 day 1 if within 2 days of the screening sample.

Haematology: FBC.

Urinalysis: Protein, glucose, ketones, blood. If urinalysis abnormal, perform microscopy – red blood cells, white blood cells, bacteria, casts, crystals.

Laboratory values that meet the criteria for CTCAE grade 3 or have changed significantly from baseline and are considered to be of clinical concern will be repeated/confirmed within 7 days and followed up as appropriate. Any AST or ALT result > 8 x ULN in presence of bilirubin increased from

baseline or >10x ULN irrespective of bilirubin result should have the blood test repeated within 48 hours and additional investigations into the aetiology should be initiated as per hepatotoxicity management algorithm.

j Fasted lipids: triglycerides, HDL, LDL and cholesterol. Fasting is defined as no calorific intake for at least 8 hours. Fasted lipids to be measured pre-dose Cycle 1 day 1 then every 6 cycles (Cycle 7 Day 1 etc) and at Discontinuation.

k Screening sample only should be fasted. Fasting is defined as no calorific intake for at least 8 hours. Plasma Glucose and Insulin to be measured 2-4 hours and 4-6 hours post dose: Cycle 1 Day 1 and Cycle 1 Day 2. Glucose and Insulin to be measured 2-4 hours post dose on Cycle 2 Day 1 also and Day 1 of subsequent cycles. **The 2-4 hour and 4-6 hour samples must be taken at least 1 hour apart.**

Glucose alone should also be measured 2-4 hours post dose on Cycle 1 days 4 and 11.

l Glycosylated haemoglobin (HbA1c) to be collected at screening, pre-dose day 1 of every third cycle and at the 28 day follow up visit.

m Investigator should assess the patient's compliance to contraceptive measures and perform a serum or urine test if required. Female patients of child-bearing potential only. Screening, pre dose on Cycle 1 day 1 and at Discontinuation. In the event of suspected pregnancy during the study, the test should be repeated and, if positive, the patient discontinued from study treatment immediately.

n CT or MRI scan of head, chest and abdomen to be performed at screening. CT or MRI scans of chest and abdomen to be performed until discontinuation. Following screening, the first tumour assessments should be performed 6 weeks after cycle 1 day 1, then every 6 weeks thereafter for the first year, later reducing to every 12 weeks. Scans should be performed $\pm$ 7 days (except 1st scan + 7 days only). If brain metastases are identified at Screening or if clinically indicated, head scanning should also be performed throughout treatment at the same time points. All scans to be reported using RECIST 1.1. The imaging modality must be used consistently throughout the course of the trial for each patient.

◊ Tumour assessments will be performed in follow up for patients who discontinue treatment for reasons other than Progressive Disease (e.g. toxicity). These scans should be performed on a 6-weekly basis for the first year relative to the start date of treatment, then every 12 weeks until disease progression or the patient starts a new anti-cancer therapy (unless the patient withdraws consent to do so). Scans should be of the chest and abdomen, and only include the head where brain metastases are identified at screening, or if clinically indicated. All scans to be reported using RECIST 1.1.

o AZD5363 must be dispensed within the IWRS Cenduit system. Refer to the Pharmacy Manual for further details. AZD5363 may be dispensed within the IWRS up to 2 days prior to the actual visit date.

p Cycle 1 Day 1: Treatment must commence within 7 days of trial registration.

q Smoking status data will be collected through questions and CO monitoring at **pre-dose** cycle 1 day 1 then every 8 weeks (day 1 of every other cycle) and at discontinuation.

r A whole blood germline DNA sample is to be collected **pre-dose** on Cycle 1 day 1. If the sample is not collected at this timepoint, it should be collected at the next visit. Refer to the Laboratory Manual for sample processing guidelines.

s ctDNA samples to be collected at Cycle 1 Day 1 then every 8 weeks (Day 1 of every other cycle) beginning cycle 3 and at discontinuation. Samples can be taken up to 2 days earlier than the actual visit date (where indicated). Refer to the Laboratory Manual for sample processing instructions.

$ ctDNA samples will be collected in follow up for patients who discontinue treatment for reasons other than Progressive Disease (e.g. toxicity). These samples should be performed at the same visit as follow up CT or MRI scans until disease progression or the patient starts a new anti-cancer therapy (unless the patient withdraws consent to do so). Samples should be collected on a 6-weekly basis for the first year relative to the start date of treatment then every 12 weeks.

t An optional fresh metastatic/recurrent tumour biopsy sample should be collected (if patient consents) post–registration (pre-treatment) and at the end of treatment visit for patients who discontinue treatment for reasons other than disease progression (origin from either the primary tumour of site of metastasis). An optional pre-treatment biopsy should not be performed in cases where the patient has already had a mandatory biopsy for molecular testing (Note - a mandatory repeat SMP2 biopsy will be performed if the patient has had targeted therapy e.g. ALK inhibitor). The discontinuation biopsy must be performed prior to commencing further anti-cancer therapy. A post-treatment biopsy will only be requested from patients with an objective response or stabilisation of disease (PR or CR), or 6 months on treatment with evidence of stabilisation (SD) for patients who have previously progressed. The tumour tissue will be used to determine possible mechanisms of resistance to study treatment. Refer to the Laboratory Manual for sample processing instructions.

u Survival status will be collected every 12 weeks (± 7 days) post-permanent discontinuation of AZD5363.

v 28 day follow up visit should be carried out 28 days (+ 7 days) post-permanent discontinuation of AZD5363.

27.3.3 Toxicity Profile

27.3.3.1 Expected Adverse Events

The AEs of hyperglycaemia, rash, diarrhoea, hypersensitivity, stomatitis, dry skin, and pruritus are expected for AZD5363. Additional AEs that are commonly reported for AZD5363, (those affecting >30% in the pooled monotherapy intermittent group), irrespective of causality, are decreased appetite, nausea, vomiting, and fatigue. Patients enrolled in the AZD5363 clinical studies have significant comorbidities, multiple concomitant medications, and have been exposed to other anticancer treatment before receiving AZD5363. A summary of the AE profile of AZD5363 is shown in Section 5.1.1 of the IB.

27.3.3.1.1 Hyperglycaemia:

Hyperglycaemia (defined in the clinical studies as at least 1 post-baseline laboratory report of a pooled glucose value [non-fasting and fasting values combined] >ULN) is a frequent clinical observation, but is transient and reversible on cessation of treatment. To date, the overall incidence of hyperglycaemia is 95.6% in the intermittent monotherapy 480 mg BD 4 days on; 3 days off dosing schedule, 100% in the intermittent 400 mg BD 4 days on; 3 days off combination with paclitaxel schedule, and 92.3% in the intermittent 400 mg BD 4 days on; 3 days off combination with fulvestrant schedule. The majority of cases occurred within the first week of study treatment (88.5% in patients treated with AZD5363 monotherapy 480 mg BD intermittent dosing, 100% in patients treated with AZD5363 400 mg BD intermittent with paclitaxel, and 84.6% in patients treated with AZD5363 400 mg BD intermittent with fulvestrant). Across these 3 dosing schedules, the proportion of patients who received metformin for elevated glucose levels ranged from 0% (intermittent dosing with paclitaxel), to 31.7% (AZD5363 intermittent monotherapy).

Consistent with the observed transient hyperglycaemia associated with AZD5363 C_{max}, it has now become apparent that there is a population trend to increasing HbA1c on AZD5363. Examination of all monotherapy, intermittent dosed patients at 400 mg and above reveals an increase in mean HbA1c of 9.27 mmol/mol (90% CI: 7.50, 11.03) at 12 weeks (i.e., an increase to 46.73 mmol/mol from the mean baseline value). A total of 156 patients from Study 1, Parts A to D, Study 3, Study 4 and Study 7, with both baseline and post-baseline measures were included in the analysis. The mean (SD) HbA1c at baseline for the population considered here was 37.5 (5.79) mmol/mol with a range from 24 to 60 mmol/mol. It appears that the majority of any HbA1C rise occurs during the first 12 weeks on treatment and that there is little subsequent elevation in the time period on drug beyond 12 weeks. The clinical relevance of this finding in the context of the populations and diseases under study is unknown. Additional analysis of HbA1c trends will be examined in the placebo-controlled data following unblinding of Study D3610C00002 (BEECH).

27.3.3.1.2 Rash:

Rash is a frequent clinical observation. To date, the overall incidence of rash (a Standardise MedDRA query (SMQ) term including the preferred terms of rash, erythema, rash erythematous, rash macular, rash maculo-papular, rash papular, rash pruritic) is 46.4% in the intermittent monotherapy 480 mg BD 4 days on; 3 days off dosing schedule, 57.1% in the intermittent 400 mg BD 4 days on; 3 days off combination with paclitaxel schedule, and 26.9% in the intermittent 400 mg BD 4 days on; 3 days off combination with fulvestrant schedule. The proportion of patients with an
AE of rash of CTCAE Grade 3 or above was 16.9%, 14.3%, and 11.5%, respectively.

In the intermittent monotherapy 480 mg BD 4 days on; 3 days off dosing schedule, 39.3% of patients had a rash that was considered to be causally related to AZD5363. Seven patients (3.8%) had an SAE of rash, and 7 (3.8%) discontinued AZD5363 treatment due to rash. A total of 51 patients (27.9%) received treatment for rash.

27.3.3.1.3 Diarrhoea:

Diarrhoea is a frequent clinical observation. To date, the overall incidence of diarrhoea (relating to the SMQ term diarrhoea) is 79.8% in the intermittent monotherapy 480 mg BD 4 days on; 3 days off dosing schedule, 85.7% in the intermittent 400 mg BD 4 days on; 3 days off combination with paclitaxel schedule, and 57.7% in the intermittent 400 mg BD 4 days on; 3 days off combination with fulvestrant schedule. The proportion of patients with an AE of diarrhoea of CTCAE Grade 3 or above was 16.9%, 28.6%, and 3.8%, respectively.

In the intermittent monotherapy 480 mg BD 4 days on; 3 days off dosing schedule, 74.3% of patients had an AE of diarrhoea that was considered to be causally related to AZD5363. Twelve patients (6.6%) had an SAE of diarrhoea, and 2 (1.1%) discontinued AZD5363 treatment due to diarrhoea. A total of 108 patients (59.0%) received treatment for diarrhoea.

27.3.3.1.4 Hypersensitivity:

Hypersensitivity is a commonly reported clinical observation being reported in 4 patients on the intermittent monotherapy dose of 480 mg BD 4 days on; 3 days off, and for 1 patient on the intermittent 400 mg BD 4 days on; 3 days off combination with paclitaxel schedule. Symptoms of hypersensitivity included rash in association with 1 or more of AEs of the following AEs: flushing, pruritus, urticaria, throat itchiness, pyrexia, and facial and/or lip oedema. In all patients, hypersensitivity or related AEs were considered causally related to AZD5363, and in 4 patients the reported AEs were considered serious, leading to hospitalisation or prolonged hospitalisation. One patient had a reported history of allergy to heat and cold. In all patients, the symptoms resolved with AZD5363 discontinuation and treatment with antihistamines and steroids.

There were no AEs of hypersensitivity in the intermittent 400 mg BD 4 days on; 3 days off combination with fulvestrant schedule.

27.3.3.1.5 Stomatitis:

Stomatitis is a very common clinical observation, affecting 32 patients (14.0%) in AZD5363 intermittent monotherapy, 10 patients (26.3%) in combination with paclitaxel, and 2 patients (7.7%) in combination with fulvestrant.

In AZD5363 intermittent monotherapy, the majority of stomatitis AEs were Grade 1 or 2 (91.4%), and the maximum severity of AEs experienced by patients was CTCAE Grade 3 (8.6%). There were 3 AEs leading to dose interruption, and of these, all resulted in a positive de-challenge, and in the 2 patients subsequently re-administered the study drug, both received a reduced AZD5363 dose and both experienced negative re-challenge. The median duration of all AEs was 15 days.

27.3.3.1.6 Dry skin:

Dry skin is a very common clinical observation, affecting 27 patients (11.8%) in AZD5363 intermittent monotherapy, 6 patients (15.8%) in combination with paclitaxel, and 1 patient (3.8%) in combination with fulvestrant.

In AZD5363 intermittent monotherapy, the majority of dry skin AEs were Grade 1 (92.9%), and the maximum severity of AEs experienced by patients was CTCAE Grade 2 (7.1%). There

were no AEs leading to dose interruption, reduction, or study discontinuation. The median duration of all AEs was 91.5 days.

27.3.3.1.7 Pruritus:

Pruritus is a common clinical observation, affecting 21 patients (9.2%) in AZD5363 intermittent monotherapy group, 7 patients (18.4%) in combination with paclitaxel, and none in combination with fulvestrant.

In AZD5363 intermittent monotherapy, the majority of pruritus AEs were CTCAE Grade 1 or 2, and 1 AE was Grade 3. There were no AEs leading to dose interruption or study discontinuation. The median duration of all AEs was 37 days.

27.3.4 Dose Modifications and Toxicity Management

27.3.4.1 Treatment delays and discontinuation

If a patient experiences a clinically significant and/or unacceptable toxicity (clinical judgement of local Investigator) dosing will be interrupted or the dose reduced and supportive therapy administered as required. If the toxicity resolves or reverts to ≤ CTCAE Grade 2 within 14 days of onset, treatment with AZD5363 may be restarted at the same or a lower dose (there should be no more than two dose level reductions). With the exception of treatment interruptions due to maculo-papular rash (see Figure 25) for all other events, if the toxicity does not resolve to ≤ CTCAE grade 2 after 14 days, then the local Investigator should consider permanent treatment discontinuation on consultation with the National Lung Matrix Trial Office, and the patient should be observed until resolution of the toxicity (see Figure 24).

In the event of a treatment interruption for reasons other than treatment related toxicity (e.g., non-cancer related surgery) lasting >3 weeks, treatment resumption will be decided on consultation with the National Lung Matrix Trial Office. It is advised that treatment delays should not exceed 6 weeks.

27.3.4.2 Dose reductions

Table 62: AZD5363 available dose levels

Dose level	AZD5363 dose
Starting dose	480 mg BD, intermittent 4 days on, 3 days off
-1	400 mg BD, intermittent 4 days on, 3 days off
-2	320 mg BD, intermittent 4 days on, 3 days off
N/A	Discontinue trial treatment

27.3.4.3 Evaluation, Management and Treatment of Toxicities

Figure 24: Toxicity Management Algorithm

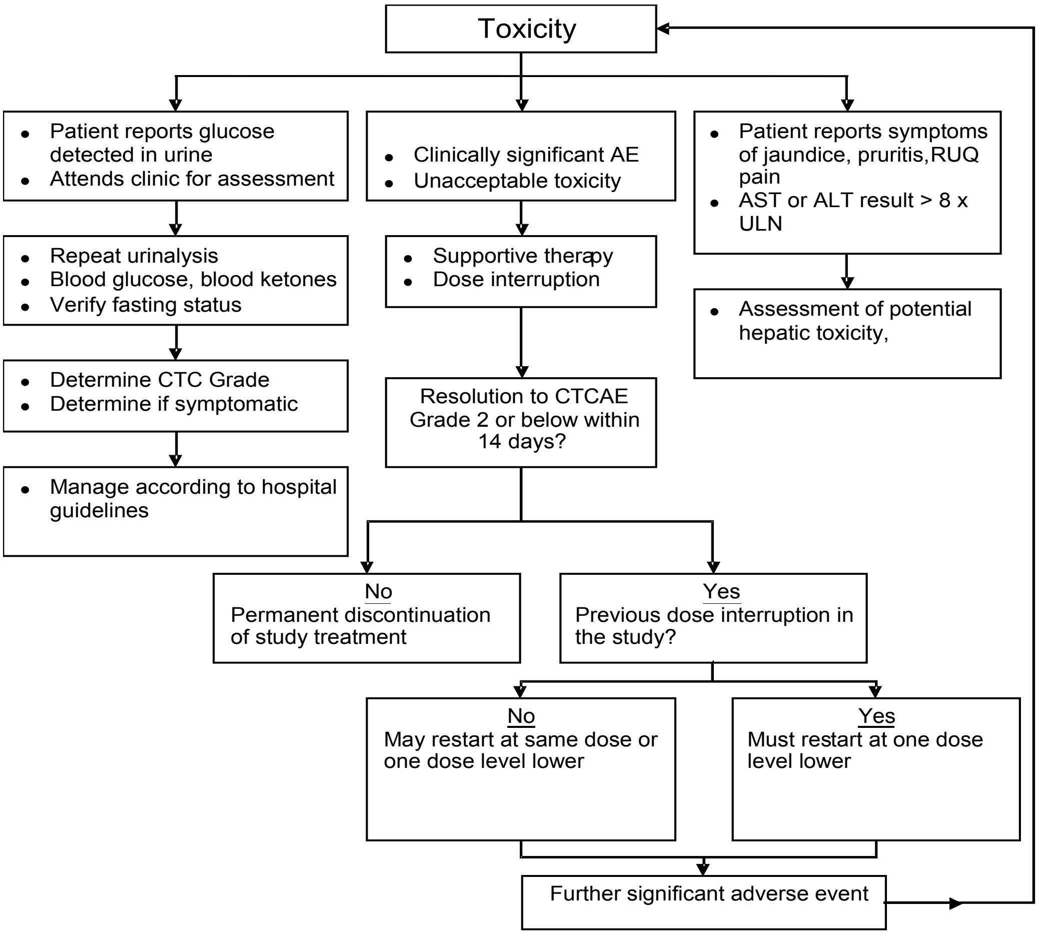

27.3.4.3.1 Rash

The vast majority of patients subjected to rechallenge following resolution of rash suffered a recurrence of the toxicity. Clinical experience indicates that rash can be managed with treatment as outlined in the rash management algorithm (e.g., use of oral or topical steroids, use of oral antihistamine), as well as by interruptions or reductions in AZD5363 dosing. The need for an interruption or dose reduction of AZD5363 should be considered with reference to the Toxicity Management Algorithm (Figure 24).

The algorithm for the management of rash is as detailed in Figure 25.

Figure 25: AZD5363 - Management of Skin Toxicity

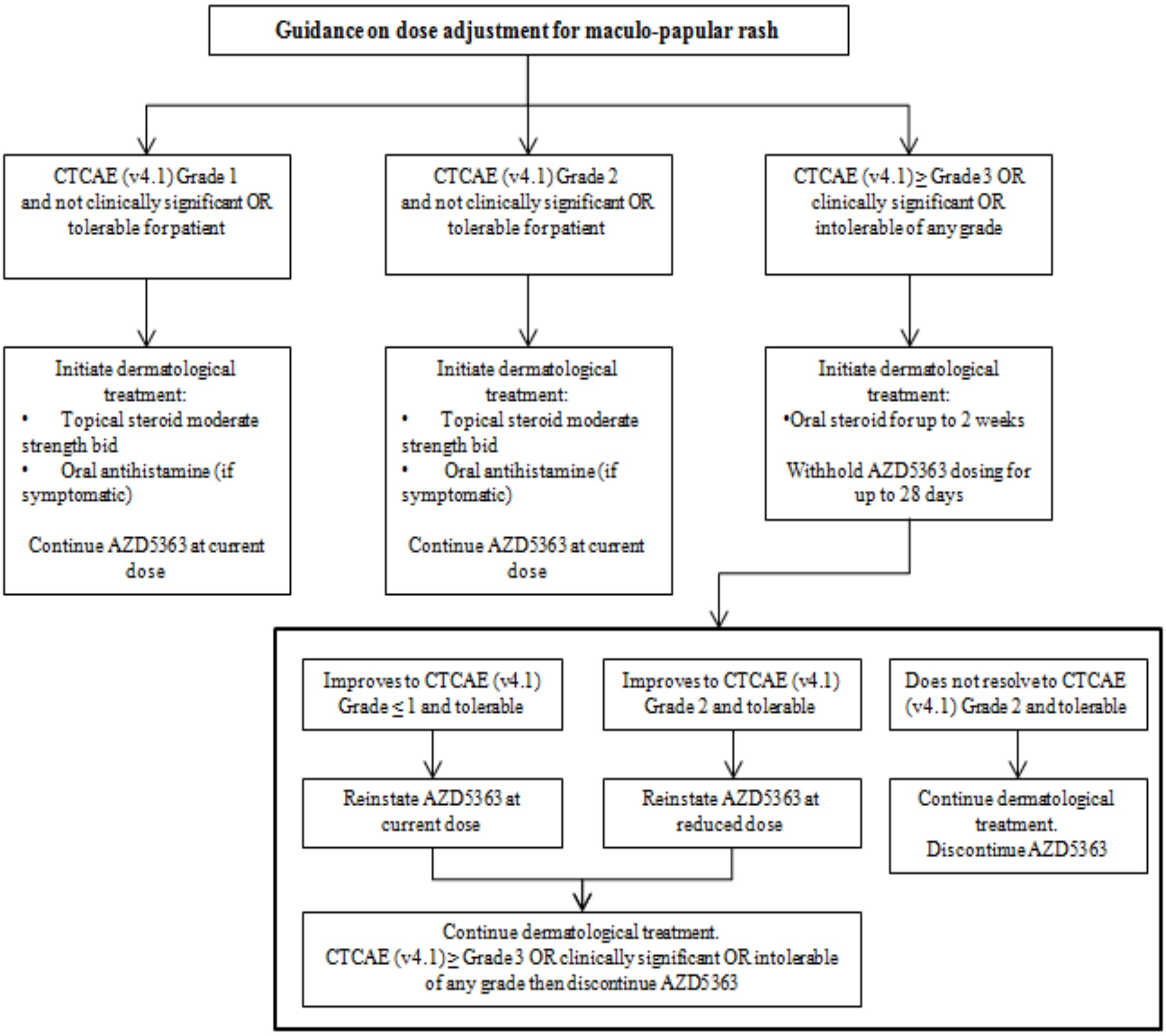

27.3.4.3.2 Diarrhoea

Patient reports of diarrhoea are to be evaluated and treated by investigators according to local practice (e.g., use of medications such as loperamide). The need for an interruption in dosing with AZD5363 should be considered according to the Toxicity Management Algorithm (Figure 24).

27.3.4.3.3 Hypersensitivity

In the case of hypersensitivity reactions, AZD5363 should be discontinued and symptomatic/supportive therapy should be initiated (including with antihistamines and/or steroids) as considered appropriate by the investigator/treating physician. Any subsequent decision on rechallenge with AZD5363 at the same or a lower dose, with its potential for recurrence of such or more severe AEs should be carefully considered against the potential benefits to the individual patient from continuation of AZD5363 treatment. Further management should follow local guidelines on management of hypersensitivity reactions.

27.3.4.3.4 Stomatitis, dry skin, and pruritus

Patient reports of stomatitis, dry skin, and pruritus are to be evaluated and treated by investigators according to local practice. The need for an interruption in dosing with AZD5363 should be considered according to the Toxicity Management Algorithm (Figure 24).

27.3.4.3.5 Glucose Homeostasis

In the period for this IB update AZ has agreed internally to cautiously permit the enrolment of well-controlled non-insulin-dependent diabetic patients in future clinical studies.

Glucose profiles should be performed at the relevant timepoints in all AZD5363 studies to adequately characterise emergent hyperglycaemia and to allow appropriate clinical management of patients. A suggested algorithm for the management of hyperglycaemia is provided below (Figure 26). In addition, because of the pharmacological activity of AZD5363 on glycolysis and insulin signalling, fasting lipid profiles (triglycerides, high density lipoprotein, low density lipoprotein and cholesterol) will also be monitored at appropriate intervals.

Patients will check for urinary glucose using a dipstick prior to breakfast at least twice a week during AZD5363 dosing days. If a patient reports urinary glucose present at home, the patient will be advised to attend the clinic the same day to have repeat urinalysis, confirmation of blood glucose and blood ketone results, and to determine the fasting status and any related symptomatology. Subsequent specific management of the hyperglycaemia will be according to local practice; however, the principles in the blood glucose intervention guideline (Figure 26) should be followed. General advice based upon previous clinical experience with related agents suggests: initial medical intervention should be an oral-antidiabetic agent e.g. metformin 500 mg twice daily on days of AZD5363 dosing.

Figure 26: AZD5363 - Management of Hyperglycaemia Blood Glucose Intervention Guideline

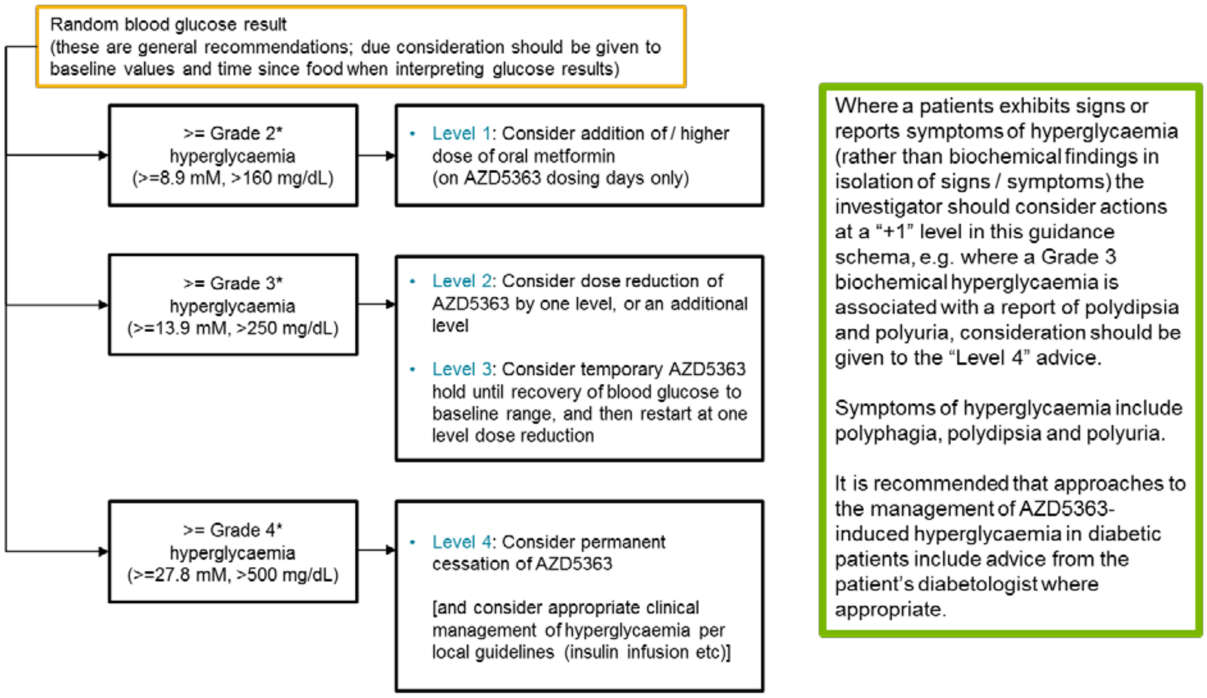

5

*These grade thresholds based on CTCAE cut-offs for fasting glucose, but applied to random glucose here.

27.3.4.3.6 Hepatotoxicity

Figure 27: AZD5363 – Management of hepatotoxicity

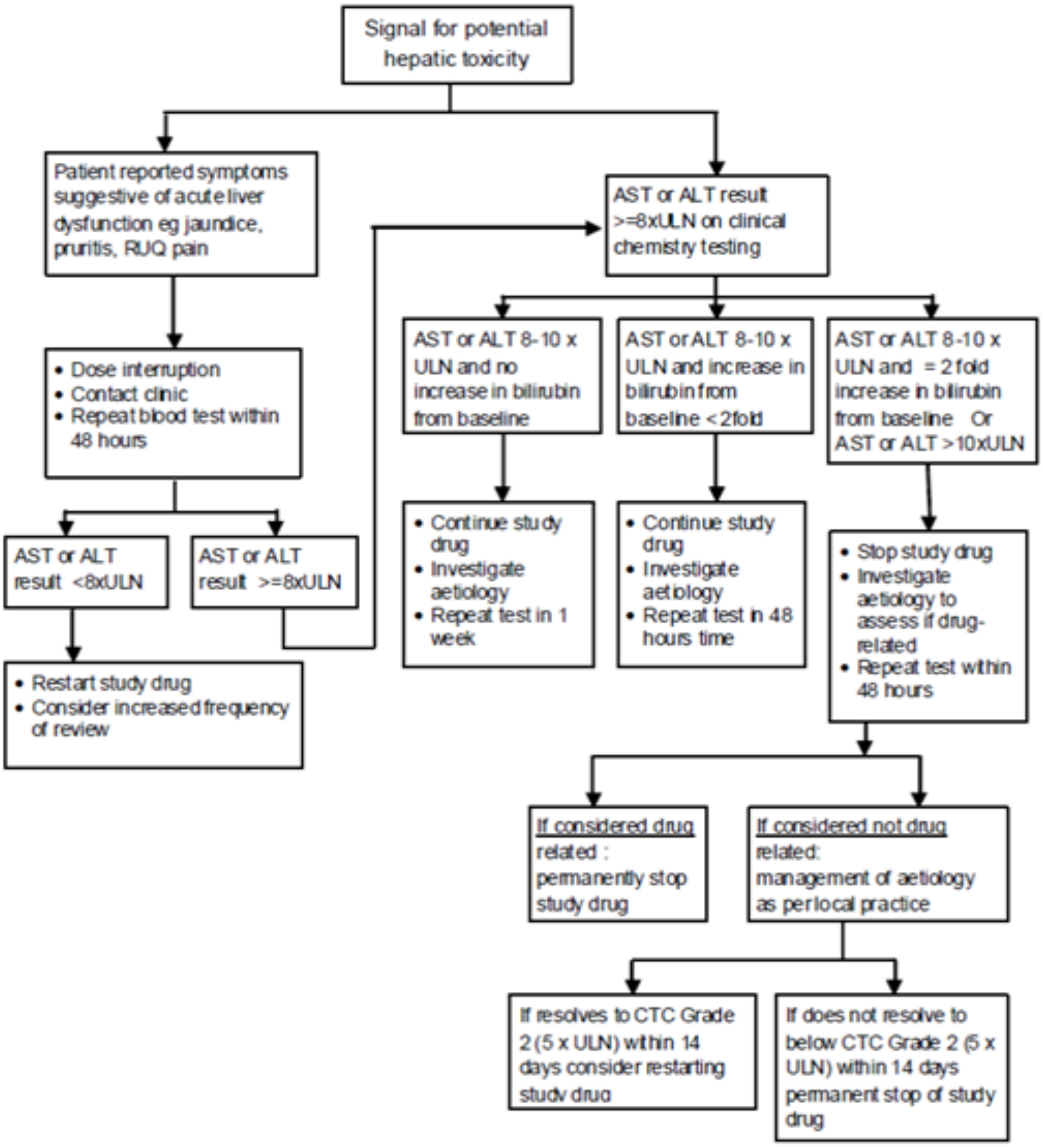

27.4 References

- Davies BR, Greenwood H, Dudley P *et al.* (2012). Preclinical pharmacology of AZD5363, an inhibitor of AKT: pharmacodynamics, antitumour activity, and correlation of monotherapy activity with genetic background. *Mol Cancer Ther* **11(4):**873-87.
- Imielinski M, Berger AH, Hammerman PS *et al.* (2012). Mapping the hallmarks of lung adenocarcinoma with massively parallel sequencing. *Cell* **150(6):**1107-20.
- Seo JS, Ju YS, Lee WC *et al.* (2012). The transcriptional landscape and mutational profile of lung adenocarcinoma. *Genome Res* **22(11):**2109-19.
- Sommer EM, Dry H, Cross D *et al.* (2013). Elevated SGK1 predicts resistance of breast cancer cells to Akt inhibitors. *Biochem J* **452(3):**499-508.
- Spoerke JM, O'Brien C, Huw L *et al.* (2012). Phosphoinositide 3-kinase (PI3K) pathway alterations are associated with histologic subtypes and are predictive of sensitivity to PI3K inhibitors in lung cancer preclinical models. *Clin Cancer Res* **18(24):**6771-83.

28 ARM G: OSIMERTINIB – EGFRM+ AND T790M+ INHIBITOR *(CLOSED TO RECRUITMENT)*

Lead Investigator: Dr Yvonne Summers

28.1 Background & Rationale

28.1.1 Molecular cohorts

Inhibitor: Osimertinib (formerly called AZD9291)

Arm	Investigational Medicinal Product	Cohort Number	NSCLC Histology	Molecular Cohort
**G**	Osimertinib – EGFR mutation positive T790M+ Inhibitor	**G1**	NSCLC	EGFR mutation & T790M mutation

Osimertinib is a potent irreversible small molecule inhibitor of both the single EGFRm+ (TKI-sensitivity conferring mutation) and dual EGFRm+/T790M+ (TKI resistance-conferring mutation) receptor forms of EGFR. Activation of EGFR tyrosine kinase (TKI) triggers a cascade of intracellular downstream signalling events affecting cell proliferation, survival, angiogenesis and potentially metastases.

28.1.2 Pre-Clinical Rationale

Selective inhibition of EGFR tyrosine kinase has demonstrated clinical benefit in approximately 70% of patients with advanced NSCLC harbouring the sensitivity mutations (the most common of which are L858R and deletions in exon 19 (Ex19del), described collectively as EGFRm+). The tumours initially respond to EGFR TKIs, but subsequently develop resistance to therapy, with a median time to progression of 9 months. In at least 50% of these initially EGFR TKI-responsive patients, disease progression is associated with the emergence of a secondary EGFR mutation, T790M in exon 20 of EGFR, which confers resistance to EGFR TKI therapy (Pao *et al.* 2005). The T790M gatekeeper resistance mutation is located in the hinge region of the kinase domain of the ATP-binding pocket of the EGFR protein, where the bulky methionine side chain prevents binding of the EGFR TKIs (Heuckmann *et al.* 2012).

Studies *in vitro* showed osimertinib to be a potent and selective irreversible inhibitor of isolated wild-type and mutant EGFRs (IC50s <10 nM). *In vitro*, cellular EGFR phosphorylation assays demonstrated potent inhibition of single-activated (EGFRm+) and double-T790M mutant (EGFRm+/T790M+) assays, and much weaker inhibition towards wild-type EGFR was observed. *In vitro* wash-out and time dependent cellular kinetic studies demonstrated an irreversible mechanism of action of osimertinib.

Oral treatment of mice bearing EGFRm+ and EGFRm+/T790M+ xenograft tumours with low doses of daily osimertinib (5 mg/kg) led to profound tumour growth regression. In contrast, higher doses of osimertinib (25 mg/kg) were required to achieve significant tumour growth inhibition and did not lead to tumour regression in wildtype EGFR xenograft models, consistent with *in vitro* selectivity margins. Xenograft growth regression with osimertinib was accompanied by dose and time dependent pharmacodynamic inhibition of phospho-EGFR (pEGFR) together with key downstream biomarkers phospho-AKT (pAKT) and phospho-ERK (pERK) across mutant and wild-type EGFR disease models *in vivo*.

Furthermore, chronic longer term oral treatment with osimertinib led to complete and sustained macroscopic disappearance of an EGFRm+ xenograft tumour suggesting the agent may provide an effective first-line treatment (AstraZeneca data on file). In support of this hypothesis, the EGFRm+ cell line showed greater time to resistance in response to osimertinib treatment *in vitro* compared to earlier generation EGFR inhibitors. The active metabolites of osimertinib, AZ5104 and AZ7550, showed similar pharmacological selectivity and activity profiles to parent, although AZ5104 showed a smaller margin of selectivity against wild-type EGFR *in vitro*.

The presence of sensitising mutations in exons 18-24 of EGFR (including L858R and Ex19del, collectively described as EGFRm) in patients with NSCLC tumours confers sensitivity to the EGFR TKI class of drugs in a high percentage of patients. However, the subsequent on-treatment emergence of the T790M gatekeeper mutation in patients treated with an EGFR TKI agent has been described as a major route of development of resistance to this class of therapy. Osimertinib is a potent and specific irreversible inhibitor of both the sensitising EGFRm mutations and the T790M resistance mutation. Non clinical data suggests that dual inhibition can result in anti-proliferative and pro-apoptotic activity in tumour models harbouring one or both of the mutations. Therefore osimertinib has the potential to provide clinical benefit to patients with NSCLC harbouring both the single sensitivity mutations and the resistance mutation following prior therapy with an EGFR TKI. Retreatment of these patients with an EGFR TKI agent is within scope of the current treatment guidelines (NCCN 2012). Osimertinib is anticipated to have a lower level of specificity towards wild-type EGFR, and as a result it is anticipated that there should be a lower level of off target toxicity such as rash and diarrhoea than currently seen with first generation EGFR TKI agents.

28.1.3 Clinical Data

Data from a phase I study of osimertinib in NSCLC patients with EGFR mutation progressing on an EGFR TKI was presented by Jänne *et al* at the 2015 European Lung Cancer Conference (Jänne *et al*, 2015). 283 pre-treated patients received the osimertinib capsule formulation (20 mg to 240 mg doses) once daily, across the dose escalation and dose expansion parts of the study. Osimertinib has a manageable tolerability profile. The proportion of patients with diarrhoea and rash of CTCAE grade ≥ 3 was 2% an1% respectively.

At the Phase II dose of 80 mg once daily, Investigator-assessed and independent centrally reviewed data suggest encouraging ORR (66% and 54%, respectively), duration of response (not calculable and 12.4 months) and progression-free survival (10.9 and 13.5 months) in patients with T790M positive NSCLC.

28.1.3.1 Early evidence of anti-tumour activity

Data pooled from Study D5160C00001 (AURA) and Study D5160C00002 (AURA2) in pre-treated patients with centrally-tested EGFR T790M mutation positive tumours, the confirmed ORR in the evaluable-for-response analysis set based on blinded independent central review (BICR) (primary analysis) was 66.1% (95% CI: 61.2, 70.7); of the 398 patients with measurable disease at baseline based on BICR assessment, 263 had confirmed objective responses to osimertinib: 2 patients (0.5%) had a best overall response of complete response (CR) and 261 (65.6%) had a best overall response of partial response (PR). Overall response rates were similar across lines of therapy (second-line, 66.9% [83/124] vs. all response rates were similar across lines of therapy (second-line, rug based on BICR.

Responses were durable, with median duration of response for the pooled analysis based on Investigator assessment (27.6% maturity) was 8.5 months (95% CI: 8.5, not calculable (NC)). The preliminary estimate of median progression free survival (PFS) for the pooled analysis in the full analysis set based on assessments by both BICR (38.7% maturity) and Investigator (38.4% maturity) was 9.7 months (95% CI: 8.3, not calculable [NC]).

In pre-treated patients with centrally-tested EGFR T790M mutation negative tumours in the AURA Phase 1 component; Investigator assessed analyses, the ORR was 24.6% (95% CI: 15.1, 36.5) in 17 of 69 evaluable for response patients. Median PFS was 3.0 months (95% CI: 2.1, 4.2; 88.4% maturity) in patients with centrally confirmed T790M mutation negative NSCLC.

In treatment naïve patients with an evaluable response in the AURA Phase I component, the ORR (confirmed and awaiting confirmation) by Response Evaluation Criteria In Solid Tumours (RECIST) version 1.1 was 75.0% (45/60 patients; 95% CI: 66.4, 82.3). The disease control rate (complete response [CR] + partial response [PR] + stable disease) was 97% (58/60 patients). There were an insufficient number of progression events to determine the PFS rate; the percentage of patients remaining progression-free at 12 months was 71.7% (95% CI: 58.0, 81.7).

28.1.3.2 Marketing Experience

Osimertinib received marketing approval under the trade name TAGRISSO from the United States Food and Drug Administration on 13th November 2015 Osimertinib 80 mg once-daily tablets are approved for “the treatment of patients with metastatic EGFR T790M+ NSCLC, as detected by an FDA-approved test, who have progressed on or after EGFR TKI therapy”. Osimertinib was granted Fast Track, Breakthrough Therapy, Priority Review and Accelerated Approval status by the FDA.

At the DCO date of 01 October 2015 for the latest Investigator’s Brochure, osimertinib had not been marketed in the US or the EU.

Osimertinib received marketing approval by the European Commission (which includes the MHRA in the UK) on the 3rd February 2016. It was announced in October 2016 that osimertinib is now available (in England) to a sub-group of patients with EGFR T790M+ NSCLC who have not received previous chemotherapy via the Cancer Drugs Fund.

28.2 Specific Eligibility Criteria

28.2.1 Inclusion Criteria

For inclusion in the study, patients must fulfil all of the following criteria:

- Patients must fulfil all the core eligibility criteria.
- Technology hub result (or locally obtained result from an approved Laboratory. See Section 6.4).
- Eastern Cooperative Oncology Group (ECOG) Performance Status $\leq$1 with no deterioration over the previous 2 weeks (see Appendix 8: Eastern Cooperative Oncology Group Performance Status Criteria)
- As a minimum, patients must have failed one or more lines of treatment including an EGFR TKI.

28.2.2 Exclusion Criteria

Patients must not enter the trial if any of the following exclusion criteria are fulfilled:

- Patients who do not fulfil all the core eligibility criteria.
- Treatment with any of the following:
  - An EGFR TKI (e.g. erlotinib or gefitinib) within 8 days or approximately 5x the half-life, whichever is the longer, prior to registration.

    - Osimertinib in the present study (i.e. dosing with osimertinib previously initiated in this study).
    - Radical radiotherapy within 4 weeks of treatment
    - Radiotherapy with a limited field of radiation for palliation within 1 week of treatment, with the exception of patients receiving radiation to more than 30% of the bone marrow or with a wide field of radiation which must be completed within 4 weeks of treatment.
    - Patients currently receiving (or unable to stop use prior to receiving the first dose of study treatment) medications or herbal supplements known to be potent inhibitors of CYP3A4 (at least 1 week prior) and potent inducers of CYP3A4 (at least 3 week prior). All patients must try to avoid concomitant use of any medications, herbal supplements and/or ingestion of foods with known inducer/inhibitory effects on CYP3A4.
    - Any other chemotherapy, investigational agents or other anti-cancer therapy within 4 weeks prior to treatment.
- Any of the following cardiac criteria:
    - Mean resting corrected QT interval (QTcF) > 470 msec obtained from 3 electrocardiograms (ECGs), using the screening clinic ECG machine derived QTcF value.
    - Any clinically important abnormalities in rhythm, conduction or morphology of resting ECG e.g., complete left bundle branch block, third degree heart block, second degree heart block.
    - Any factors that increase the risk of QTc prolongation or risk of arrhythmic events such as heart failure, hypokalaemia, congenital long QT syndrome, family history of long QT syndrome or unexplained sudden death under 40 years of age in first degree relatives, or any concomitant medication known to prolong the QT interval (see Section 28.2.3.1).
- Hepatic function (in patients **with** liver metastasis).
    - Alanine transferase (ALT) and Aspartate transferase (AST) >5 x ULN.
    - Serum bilirubin >3 x ULN.
- Past medical history of interstitial lung disease, drug-induced interstitial lung disease, radiation pneumonitis which required steroid treatment, or any evidence of clinically active interstitial lung disease.
- History of hypersensitivity to active or inactive excipients of osimertinib or drugs with a similar chemical structure or class to osimertinib.
- Any unresolved toxicities from prior therapy greater than CTCAE grade 1 at the time of starting study treatment with the exception of alopecia and grade 2, prior platinum-therapy related neuropathy.
- Refractory nausea and vomiting, chronic gastrointestinal diseases, inability to swallow the formulated product or previous significant bowel resection that would preclude adequate absorption of osimertinib.

28.2.3 Restrictions & Concomitant Medications

Information on any treatment from the date of informed consent until 28 days after the administration of the last treatment dose should be recorded. If medically feasible, patients taking regular medication should be maintained on it throughout the study period. Patient should be advised to inform their treating physicians of all concomitant medications, including prescription medicines, over-the-counter drugs, vitamins, and herbal products.

Nb. These lists are not exhaustive and the absence of a drug from the lists does not imply that its combination with osimertinib is safe.

28.2.3.1 QT Interval Prolongation

Concomitant medications **known to prolong the QT interval** are prohibited during the active treatment phase. Any patients taking such drugs at or prior to registration should discontinue the drug 6 half-lives plus 1 day prior to commencing trial treatment. For a list of drugs known to prolong the QT interval, (or with any risk of prolonging the QT interval), please refer to the following database: https://www.crediblemeds.org. Appendix 9 Credible Meds List of Drugs that Prolong QT Interval contains a list exported from this database on 2nd March 2018. **It is important to note that this list is a guide – the database will change with time and therefore needs to be checked in real-time when screening and registering a patient, and throughout their treatment. The Trials Office will email all sites when updates are made to the database.**

Please note that for AstraZeneca trial Arms, only concomitant medications listed on https://www.crediblemeds.org under the category 'Known risk of TdP' will exclude patients from entering the trial as per the eligibility criteria.

It is the responsibility of an Investigator (Consultant level) to review and clinically evaluate all concomitant medications. Please contact the National Lung Matrix Trial Office for clarification regarding any drugs that appear on the database that are not listed in Appendix 9 Credible Meds List of Drugs that Prolong QT Interval.

The drugs listed on this website are taken from information provided by The Arizona Centre for Education and Research on Therapeutics and The Critical Path Institute, Tucson, Arizona and Rockville, Maryland. Important Note - If a patient is being treated with such medication or is taking another medication that may affect QT interval which is not on the database, please contact the National Lung Matrix Trial Office to obtain the recommended withdrawal/ minimum period prior to starting trial treatment.

28.2.3.2 Cytochrome P450 (CYP) Related Induction and Inhibition

28.2.3.2.1 Drugs inducing CYP3A4 metabolism that AstraZeneca strongly recommend are not combined with osimertinib

Osimertinib is metabolised by CYP3A4 and CYP3A5 enzymes. A drug-drug interaction study of osimertinib evaluated in patients showed that there is potential for osimertinib being a victim when co-administered with strong inducers of CYP3A4 (osimertinib concentrations are decreased when co-dosed with rifampicin).

All patients must try to avoid concomitant use of medications, herbal supplements and/or ingestions of foods with known potent inducer effects on CYP3A4 activity whenever feasible. Such drugs must have been discontinued for an appropriate period before they enter screening and for a period of 2 weeks after the last dose of osimertinib (see Table 63 for required washout period for prohibited drugs).

Patients taking concomitant mediations whose disposition is dependent upon intestinal CYP3A4 and/or transporter proteins and which have a narrow therapeutic index should be closely monitored for signs of changed tolerability as a result of increased exposure of the concomitant medication whilst receiving osimertinib.

The following potent inducers of CYP3A4 must not be used during this study for any patient receiving osimertinib:

Table 63: Prohibited drugs inducing CYP3A4

Contraindicated drugs	Withdrawal period prior to osimertinib start
Carbamazepine, phenobarbital, phenytoin<br>Rifampicin, rifabutin, rifapentin<br>St John's Wort	3 weeks
Phenobarbitone	5 weeks

This list is not intended to be exhaustive, and a similar restriction will apply to other agents that are known to strongly modulate CYP3A4 activity. Appropriate medical judgment is required.

28.2.3.3 BCRP Substrates

Osimertinib may increase the concentration of sensitive BCRP substrates (concentration of the sensitive BCRP substrate, rosuvastatin, is increased). It is recommended that patients taking concomitant medications with disposition dependent upon BCRP and with a narrow therapeutic index (e.g. rosuvastatin) should be closely monitored for signs of changed tolerability as a result of increased exposure of the concomitant medication whilst receiving osimertinib.

Table 64: Permitted (with caution) BCRP substrates

Warning of possible interaction	Advice
Sulfasalazine<br>Doxorubicin<br>Daunorubicin<br>Topotecan	Drugs are permitted but caution should be exercised and patients monitored closely for possible drug interactions. Please refer to full prescribing information for all drugs prior to co-administration with osimertinib.
Rosuvastatin	In addition to the above advice, patients taking rosuvastatin should have creatine kinase (CK) levels monitored due to BCRP-mediated increase in exposure. If the patient experiences any potentially relevant AEs suggestive of muscle toxicity (including unexplained muscle pain, tenderness, or weakness) particularly if accompanied by malaise or fever, rosuvastatin must be stopped and any appropriate further management should be taken.

28.2.3.4 Concomitant Radiotherapy

If a patient requires palliative radiotherapy, consideration should be given to suspending trial treatment based on local practice and following discussion with the National Lung Matrix Trial Office.

28.2.3.5 Other Restrictions

The following restrictions apply while the patient is receiving trial treatment and for the specified times before and after:

- The use of any natural/herbal products or other "folk remedies" should be discouraged, but use of these products, as well as use of all vitamins, nutritional supplements, and all other concomitant medications must be recorded in the CRF. St John's Wort is prohibited.
- Based on *in vitro* studies, osimertinib is a competitive inhibitor of CYP 3A4/5 but not CYP1A2, 2A6, 2B6, 2C8, 2C9, 2C19, 2D6 and 2E1 at clinically relevant concentrations. Based on *in vitro* studies, osimertinib is not an inhibitor of UGT1A1 and UGT2B7 at clinically relevant concentrations hepatically. Intestinal inhibition of UGT1A1 is possible but the clinical impact is unknown.
- Patients taking rosuvastatin should have creatine phosphokinase levels monitored (due to BCRP-mediated increase in exposure). If the patient experiences any potentially relevant AEs suggestive of muscle toxicity including unexplained muscle pain, tenderness, or weakness, particularly if accompanied by malaise or fever, rosuvastatin must be stopped and any appropriate further management should be taken
- *In vitro* studies have shown that osimertinib is not a substrate of OATP1B1 and OATP1B3. *In vitro*, osimertinib does not inhibit P-glycoprotein, OAT1, OAT3, OATP1B1, OATP1B3, MATE1, MATE2K and OCT2 at clinically relevant concentrations. Based on *in vitro* studies, osimertinib is a substrate of P-gp and BCRP, but is unlikely to result in clinically relevant drug interactions with active substances by osimertinib at the clinical doses. Based on *in vitro* data, osimertinib is an inhibitor of BCRP (see Section 28.2.3.3).

28.2.3.6 Contraception

Acceptable methods of contraception include those listed in Section 6.3. Hormonal contraceptives that are not prone to drug-drug interactions should be used (e.g. IUS Levonorgestrel Intra Uterine System (Mirena), Medroxyprogesterone injections (Depo-Provera).

Females of child-bearing potential should use highly reliable methods of contraception from the time of screening until at least 3 months after discontinuing study treatment.

Male patients should be asked to use barrier contraceptives (i.e., by use of condoms) during sex with all partners during the trial and for a washout period of at least 3 months following treatment discontinuation. Patients should avoid procreation for 6 months after completion of trial treatment. Patients should refrain from donating sperm from the start of dosing until 6 months after discontinuing study treatment. If male patients wish to father children they should be advised to arrange for freezing of sperm samples prior to the start of study treatment, and not to donate sperm until 6 months after discontinuation of study treatment.

28.3 Trial Treatment

28.3.1 Investigational Medicinal Product

Osimertinib will be administered orally as one 80 mg tablet once a day. A cycle of treatment is defined as 21 days of once daily osimertinib treatment.

Osimertinib is an oral, irreversible inhibitor of the tyrosine kinase activity of the epidermal growth factor receptor sensitising mutation (EGFRm+) and the resistance mutation (T790M+)in patients with NSCLC. Osimertinib will be supplied free of charge by AstraZeneca. The tablet is presented as 40 mg and 80 mg strength tablets. The dose of osimertinib will be 80 mg once daily.

It is recommended that osimertinib can be taken with or without food.

Doses should be taken approximately 24 hours apart at the same time point each day. If a scheduled dose is delayed within a window of 12 hours, it is still acceptable to take the dose. If longer than 12 hours after the scheduled dose time, the dose should not be taken and the patients should be instructed to take the next dose at the next scheduled time. It is important that there are no patient double doses (i.e. takes 2 doses simultaneously). Vomited doses should not be made up. Both the Arm G Patient Information Sheet and Arm G Patient Diary contain more specific instructions for patients to follow regarding how to take their medication.

Please note patients who meet RECIST criteria for progressive disease (PD) may be continued on trial treatment if the treatment is tolerable and the Investigator believes it to be of clinical benefit; see Section 9.3.

Please also refer to the Pharmacy Manual for further details.

28.3.2 Schedule of Assessments

Table 65: Osimertinib (AZD9291) - Schedule of Assessments

	**Screening**	**Treatment 80 mg once daily (21 day cycles)**				**Discontinuation** (+ 7 days)**	**28 day follow up**[u] (+7 days)**	**Survival Assessments** (± 7 days)***
	Within 28 days of treatment (unless otherwise stated)	Cycle 1			Cycle 2 onwards (± 2 days)*			
		Day 1	Day 8	Day 15	Day 1			
Informed consent[a]	X							
Demography & baseline characteristics[b]	X							
Medical history[c]	X							
Inclusion / exclusion criteria[d]	X							
Physical examination[e]	X	X			X	X		
ECOG performance status[f]	X (within 14 days of treatment)	X			X	X		
Vital signs (inc. weight)[g]	X	X			X	X		
ECG[h]	X	X			X	X		
MUGA / Echocardiogram[i]	X	(If clinically indicated)					(If clinically indicated)	
Ophthalmology examination[j]	X	(If clinically indicated)						

	Screening	Treatment 80 mg once daily (21 day cycles)				Discontinuation (+ 7 days)**	28 day follow up[u] (+7 days)**	Survival Assessments (± 7 days)***
	Within 28 days of treatment (unless otherwise stated)	Cycle 1			Cycle 2 onwards (± 2 days)*			
		Day 1	Day 8	Day 15	Day 1			
Haematology, Clinical chemistry, Urinalysis[k]	X (within 7 days of trial treatment)	X	X	X	X	X		
Pregnancy test[l]	X	X				X		
Tumour assessments[m]	X	Every 6 weeks during year 1 (± 7 days) [except 1st scan + 7days only]						X[illegible]
Adverse events & Concomitant Medications	X	X	X	X	X	X	X	
Dispense study drug[n]		X (-2 days)			X (-2 days)			
Administer study drug[o]		X	X	X	X			
Smoking status[p]		X			X (every 9 weeks beginning cycle 4)	X		
Germline DNA sample[q]		X						
ctDNA samples[r]		X			X (every 9 weeks beginning cycle 4) (-2 days)	X		X $
Optional research biopsy[s]		X (post-reg, pre-tx)				X		

	Screening	Treatment 80 mg once daily (21 day cycles)				Discontinuation (+ 7 days)**	28 day follow up[u] (+7 days)**	Survival Assessments (± 7 days)***
	Within 28 days of treatment (unless otherwise stated)	Cycle 1			Cycle 2 onwards (± 2 days)*			
		Day 1	Day 8	Day 15	Day 1			
Survival status[t]								X

* Visit may occur ± 2 days of the planned visit date. Individual assessments may occur independently of the visit date where indicated in the table above.

** Visit may occur + 7 days of the planned visit date.

*** Visit may occur ± 7 days of the planned visit date.

a Prior to the start of any study specific procedures, each patient must provide signed informed consent.

b Demography must be captured for all patients, including screen failures. Demographic data and other characteristics will include: date of birth, gender, race and ethnicity.

c A standard medical and surgical history will be obtained, including prior cancer treatment and smoking status.

d Patients must not be registered unless all eligibility criteria have been fully met.

e Physical examination includes general appearance, respiratory, cardiovascular, skin, head and neck (including ears, eyes, nose and throat), lymph nodes, thyroid, abdomen, musculo-skeletal (including spine and extremities) and neurological systems will be required at Screening, Day 1 of each Cycle and at Discontinuation.

Physical examination on Day 1 of subsequent cycles and discontinuation can be performed at any time of day.

f ECOG performance status will be assessed at Screening, pre-dose on Cycle 1 day 1, day 1 of every subsequent Cycle and at Discontinuation.

g Vitals signs are to be recorded are height (at screening only), weight, BP and pulse; required at screening, day 1 of every Cycle, on occurrence of any cardiac AE and at Discontinuation.

h	12-lead ECG to be taken at screening, day 1 of each cycle, on occurrence of any cardiac AE, and at Discontinuation. ECG performed on Day 1 of subsequent cycles and Discontinuation can be performed at any time of day.<br>Twelve-lead ECGs will be obtained after the patient has been resting semi-supine for at least 10 minutes prior to times indicated. All ECGs should be recorded with the patient in the same physical position. A standardised ECG machine should be used and the patient should be examined using the same machine throughout the study if possible. After paper ECGs have been recorded, the Investigator or designated physician will review each of the ECGs and may refer to a local cardiologist if appropriate. A paper copy should be filed in the patient's medical records. If an abnormal ECG finding at screening or Cycle 1 day 1 is considered to be clinically significant by the Investigator, it should be reported as a concurrent condition. For all ECGs details of rhythm, ECG intervals (R-R, PR, QT and QRS) and an overall evaluation will be recorded.
i	A scan will be carried out at Screening. Further MUGA/ECHO should be carried out during treatment if a patient develops signs and/or symptoms suggestive of a deterioration in left ventricular function or in case of the pre-specified ECG finding such as T-wave inversions. If LVEF assessment is abnormal at the time of treatment discontinuation, a 28 day follow up assessment should be performed to confirm reversibility of the abnormality. The modality of the cardiac function assessments must be consistent within patient and the same machine operator is to be used where possible.
j	Full ophthalmic assessment, including slit lamp examination, should be performed at screening. Repeat ophthalmologic examination should be considered during the study when visual disturbances (e.g. visual impairment, photopsia, blurred vision, vitreous floaters) have been observed and persist or worsen in severity. If the patient reports any eye symptoms during treatment or if signs are observed during a study visit, a follow up clinical examination should be performed, including a repeat best corrected near and distant visual acuity assessment if appropriate. Any clinically significant findings, including those confirmed by the ophthalmologist must be reported as an AE.

k Blood and urine samples for safety assessment will be collected at Screening, **pre-dose** day 1, 8 and 15 Cycle 1, **pre-dose** day 1 of subsequent Cycles and Discontinuation. Discontinuation samples can be collected at any time of the day.

Clinical Chemistry: Albumin, AST, ALT, Alkaline phosphatase, bilirubin (total), calcium (total), creatinine, glucose, magnesium, sodium, urea nitrogen, potassium.

Haematology: FBC & reticulocytes. Hba1c should be performed day 1 of every cycle only.

Urinalysis: protein, glucose and blood.

All patients with clinically significant abnormal laboratory results at treatment completion or study drug discontinuation visit are to be followed until the results return to normal (or patient's baseline), or until a valid reason, other than a drug-related effect, is identified. Patients with an unresolved AE or SAE event at treatment completion or study drug discontinuation will be contacted by the Investigator or his or her designee to determine the status of the event until the event is resolved or stabilized, the patient is lost to follow up, or it has been determined that the study treatment or participation is not the cause of the event.

Laboratory values that meet the criteria for CTCAEv4 grade 3 or have changed significantly from baseline and are considered to be of clinical concern will be repeated/confirmed within 7 days and followed up as appropriate.

l Investigator should assess the patient's compliance to contraceptive measures and perform a serum or urine test if required. Female patients of child-bearing potential only at Screening, pre dose on Cycle 1 day 1 and at Discontinuation. In the event of suspected pregnancy during the study, the test should be repeated and, if positive, the patient discontinued from study treatment immediately.

m CT or MRI scan of head, chest and abdomen to be performed at Screening. CT or MRI scans of chest and abdomen to be performed until discontinuation. Tumour assessments should be performed relative to the date of start of treatment (cycle 1 day 1) every 6 weeks for the first year, then every 12 weeks. Scans should be performed ± 7 days (except 1st scan + 7 days only). If brain metastases are identified at Screening or clinically indicated, head scanning should also be performed throughout treatment at the same time points. All scans to be reported using RECIST 1.1. **The imaging modality must be used consistently throughout the course of the trial for each patient.**

◊ Tumour assessments will be performed in follow up for patients who discontinue treatment for reasons other than Progressive Disease (e.g. toxicity). These scans should be performed on a 6-weekly basis for the first year relative to the start date of treatment, then every 12 weeks until disease progression or the patient starts a new anti-cancer therapy (unless the patient withdraws consent to do so). Scans should be of the chest and abdomen, and only include the head where brain metastases are identified at screening, or if clinically indicated. All scans to be reported using RECIST 1.1.

n Osimertinib must be dispensed within the IWRS Cenduit system. Refer to the Pharmacy Manual for further details. Osimertinib may be dispensed within the IWRS up to 2 days prior to the actual visit date.

o Cycle 1 (Day 1): Treatment must commence within 7 days of trial registration.

p Smoking status data will be collected through questions and CO monitoring at **pre-dose** cycle 1 day 1, cycle 4 day 1 then every 9 weeks (day 1 of every 3rd cycle) and at discontinuation.

q A whole blood germline DNA sample is to be collected **pre-dose** on Cycle 1 day 1. If the sample is not collected at this timepoint, it should be collected at the next visit. Refer to the Laboratory Manual for sample processing guidelines.

r ctDNA samples to be collected at pre-dose cycle 1 day 1, Cycle 4 Day 1 then every 9 weeks (Day 1 of every third cycle) and at discontinuation. Samples can be taken up to 2 days earlier than the actual visit date (where indicated). Refer to the Laboratory Manual for sample processing instructions.

$ ctDNA samples will be collected in follow up for patients who discontinue treatment for reasons other than Progressive Disease (e.g. toxicity). These samples should be performed at the same visit as follow up CT or MRI scans until disease progression or the patient starts a new anti-cancer therapy (unless the patient withdraws consent to do so). Samples should be collected on a 6-weekly basis for the first year relative to the start date of treatment then every 12 weeks.

s An optional fresh metastatic/recurrent tumour biopsy sample should be collected (if patient consents) post–registration (pre-treatment) and at the end of treatment visit for patients who discontinue treatment for reasons other than disease progression (origin from either the primary tumour of site of metastasis). An optional pre-treatment biopsy should not be performed in cases where the patient has already had a mandatory biopsy for molecular testing (Note - a mandatory repeat SMP2 biopsy will be performed if the patient has had targeted therapy e.g. ALK inhibitor). The discontinuation biopsy must be performed prior to commencing further anti-cancer therapy. A post-treatment biopsy will only be requested from patients with an objective response or stabilisation of disease (PR or CR), or 6 months on treatment with evidence of stabilisation (SD) for patients who have previously progressed. The tumour tissue will be used to determine possible mechanisms of resistance to study treatment. Refer to the Laboratory Manual for sample processing instructions.

t Survival status will be collected every 12 weeks (± 7 days) post-permanent discontinuation of osimertinib.

u 28 day follow up visit should be carried out 28 days (+ 7 days) post-permanent discontinuation of osimertinib.

28.3.3 Toxicity Profile

Detailed information on the non-clinical toxicity studies with osimertinib in rats and dogs is available in the IB.

28.3.3.1 Expected Adverse Events

The monitoring and management of the potential risks based upon the non-clinical toxicity data is discussed below:

28.3.3.1.1 Gastrointestinal tract effects including stomatitis

Diarrhoea is the most commonly reported AE with osimertinib, reported at a Council for International Organisations of Medical Sciences (CIOMS) frequency of very common (>10%). The majority of reported AEs of diarrhoea were considered mild or moderate in severity. AEs of diarrhoea were rarely reported as CTCAE ≥ Grade 3 and did not generally lead to discontinuation of osimertinib. Median time to onset of first event of diarrhoea was 18.0 days (n=174; mean 43.0, range 1 to 251 days). After one month of treatment with osimertinib there is an approximate 20% chance of an adverse event of diarrhoea at any point in time. Where treatment was required the most frequently used class of medication administered were anti-propulsives (e.g., loperamide).

There were no reports of haemorrhagic diarrhoea or GI perforation in Phase I or Phase II studies.

Stomatitis has been reported by NSCLC patients in clinical trials with osimertinib during the reporting period, at a frequency between 5.5% and 13.7% across dose levels, with most events mild in severity and a small number of moderate cases. Only 1 patient experienced a CTCAE Grade 3 or above event. There is an upward inflection in the incidence of stomatitis between 80 mg and 160 mg. The impact of stomatitis on patients receiving osimertinib, as measured by numbers of SAEs (including hospitalisations) and dose modifications or discontinuations is low.

Patients with refractory nausea, vomiting and chronic gastrointestinal diseases are excluded from participating in this study (see arm-specific exclusion criteria). Refer to Section 28.3.4.3 for detailed guidance regarding diarrhoea management.

28.3.3.1.2 Dermatological effects and nail disorders

Dermatological events such as rash, acne and dry skin have occurred in the osimertinib clinical studies at a CIOMS frequency of common (>1% and <10%) to very common (>10%) across dose levels. Most events were CTCAE Grade 1.

Nail effects have previously been identified as a potential risk of treatment with osimertinib as a known class effect for EGFR inhibitors. Nail effects is a group term of closely related events, which includes changes to the nail and nail bed which have been reported at all doses of osimertinib in clinical trials. The highest incidence has been seen at the highest doses of osimertinib and the incidence ranges from 25.1% for the 80 mg dose to 50.0% for the 240 mg dose. Nail effects have been mostly mild (CTCAE Grade 1) or moderate (CTCAE Grade 2) in severity. There has been one severe event (paronychia, CTCAE Grade 3) for which dosing was interrupted. Median time to onset for nail effects (grouped terms) ranges from 1 day to 296 days, with no clear differences between dose levels.

There are no specific dermatological exclusion criteria. However patients with any unresolved adverse event from prior therapy greater than CTCAE Grade 1 will be excluded from participation (see arm-specific exclusion criteria). Dermatological treatment should be instituted for patients with any CTCAE Grade skin reactions, considered by the local Investigator to be causally related to osimertinib. Refer to Section 28.3.4.3 for detailed guidance on management of skin reactions. This information is for guidance but is strongly recommended and is based on experience available with other EGFR TKI and Erb inhibitor agents, which included discussions with an expert dermatologist. However, it should only be implemented if considered by the local Investigator to be clinically appropriate. Local Investigators are also advised to follow the general toxicity management guidelines regarding dose interruption and reduction as included in Section 28.3.4.

Both erythema multiforme (EM) and Stevens-Johnson syndrome (SJS) have been uncommonly and rarely reported, respectively, in association with osimertinib treatment. Before initiating treatment, patients should be advised of signs and symptoms of EM and SJS. If signs and symptoms suggestive of EM develop, close patient monitoring and drug interruption or discontinuation of osimertinib should be considered. If signs and symptoms suggestive of SJS appear, osimertinib should be interrupted or discontinued immediately.

28.3.3.1.3 Ocular surface effects

Keratitis was reported in 0.7% (n=8) of the 1142 patients treated with osimertinib in the AURA and FLAURA studies. Patients presenting with signs and symptoms suggestive of keratitis such as acute or worsening: eye inflammation, lacrimation, light sensitivity, blurred vision, eye pain and/or red eye, should be referred promptly to an ophthalmology specialist. Any clinically significant findings, including those confirmed by the ophthalmologist must be reported as an AE.

28.3.3.1.4 Cardiovascular effects

Cardiac Contractility:
Of the 411 patients in the AURA studies, Left ventricular ejection fraction (LVEF) decreases greater than or equal to 10% and a drop to less than 50% occurred in 3.9% (35/908) of patients treated with osimertinib who had baseline and at least one follow-up LVEF assessment. Based on the available clinical trial data, a causal relationship between effects on changes in cardiac contractility and osimertinib has not been established.

In patients with cardiac risk factors and those with conditions that can affect LVEF, cardiac monitoring, including an assessment of LVEF at baseline and during treatment, should be considered. In patients who develop relevant cardiac signs/symptoms during treatment, cardiac monitoring including LVEF assessment should be considered.

QTc:
Across AURA Phase I and Phase II; and AURA2, an increase in QTcF interval compared with baseline was observed, with a median increase of approximately 15 msec, at steady state on Cycle 3 Day 1 but with no evidence that this is associated with an increased risk of cardiac arrhythmias or TdP.

In AURA2 an intensive assessment of ECG parameter was performed addressing key aspects of a conventional thorough QT/QTc study, including time-matched ECG assessments, to enable evaluation of any pro-arrhythmic risk of osimertinib.

The mean time-matched change from baseline in QTcF at Week 6 across all time points was 14.5 ms (90% CI 14.0, 15.0), with the maximum upper 90% CI limit at any time point being

17.5 ms. In AURA2, based on the time-matched pre-dose baseline and post-dose ECG samples with post-dose PK sampling at the same time points, the mean (90% CI) increase in ΔQTcF interval was estimated to be 0.271 (90% CI: 0.241, 0.301) msec per 10-nM increase in osimertinib plasma levels, based on a linear mixed effects model, with gender not having a significant effect in the model. Based on this concentration QTc analysis, the predicted drug related QTc interval prolongation at the proposed osimertinib therapeutic dose (80 mg) is 14.2 msec (upper bound of 90% CI: 15.8 msec).

This magnitude of effect exerted by osimertinib is considered to be of limited clinical significance as evidenced by the low number of AEs reported under the QT prolongation SMQ in the Phase II studies - 17 (4.1%) patients with ECG QT prolonged: 9 (2.2%) patients had AEs that were maximum CTCAE Grade 1, 3 (0.7%) patients had AEs that were maximum CTCAE Grade 2, and 5 (1.2%) patients had AEs that were maximum CTCAE Grade 3.

At the population level, no clinically significant changes in the PR, RR or QRS intervals were identified during study treatment in any of the clinical trials.

Other Cardiac Disorders:

Across all the AURA Phase I and Phase II components, and the AURA2 study, the nature, frequency and severity of cardiac disorders observed is consistent with the level expected in an advanced lung cancer population.

A total of 36 patients (8.8%) were reported to have cardiac disorders during the Phase II studies, of which 69% (25/36) were Grade 1 or 2. There were less than 5% of patients who experienced Grade 3/4 toxicities. There have been a total of 2 reports with fatal outcome: Acute Congestive Heart Failure and Pulseless Electrical Activity.
Patients who have unstable cardiac conditions and risk factors for QT prolongations will be excluded from participation in this trial (see arm-specific exclusion criteria). Concomitant use of regular medications that may prolong the QT interval will be restricted whenever feasible, but patients may receive any medication that is clinically indicated for the treatment of AEs. Electrolyte and vital sign assessments, including pulse rate and blood pressure, will be monitored regularly throughout the trial. ECG assessments will be performed at baseline and as clinically indicated. Where appropriate, ECGs will be recorded at the beginning of each subsequent treatment cycle. The local Investigator or designated physician will review each ECG prior to discharge from the clinic and may refer to a local cardiologist if appropriate for immediate management of the patient. A paper copy should be filed in the patient’s medical records. If an abnormal ECG finding at screening or baseline is considered to be clinically significant by the local Investigator, it should be reported as a concurrent condition.

28.3.3.1.5 Respiratory effects, including interstitial lung disease (ILD)

Osimertinib is associated with ILD; many EGFR inhibitors are also associated with this type of event. In the Phase II studies (AURA extension and AURA2), at the DCO date of 01 May 2015 ILD (grouped terms) was reported in 2.7% (11/411) of patients during treatment with osimertinib 80 mg in the Phase II studies. The median time to onset for ILD grouped term events in the Phase II studies was 83 days (range 17 to 230 days). The distribution of patients is as follows:

- 7 Asian patients, 4 non-Asian patients
- 8 reports were serious, while 3 reports were non-serious
- There were 4 patients with CTCAE Grade 1 AEs; 3 patients with CTCAE Grade 3 AEs, and 4 patients with fatal AEs
- 5 patients recovered, 1 patient was recovering, 1 patient had not recovered, and for 4 patients the outcome was fatal.

Considering the importance of ILD as an event, an additional analysis has been conducted, which includes other ongoing studies with osimertinib, at a DCO date of 01 June 2015. At this date, osimertinib had been dosed to 1221 patients across the clinical development programme (excluding healthy volunteers who only received single doses of osimertinib, and excluding patients treated with osimertinib in Study D5160C00007, a double-blind randomised controlled trial). A total of 2.9% (35/1221) patients have reported ILD or suspected ILD-like events.

Fatal and life-threatening reports of ILD have been received and are considered as expected.

In the pooled dataset of 1142 patients who received osimertinib at a dose of 80 mg daily in FLAURA and AURA studies (see Section 5.4 of IB), no serious adverse reactions of pneumonitis assessed as life-threatening have been reported; however, more than 15 post-marketing case reports of life-threatening pneumonitis have been received. Therefore, given that cases of fatal pneumonitis have been reported (which is clinically more severe than life-threatening pneumonitis), in order to ensure medical consistency and sound clinical reasoning, life threatening pneumonitis is now considered as expected. The upper limit of the 95% CI for the point estimate of the frequency of life-threatening pneumonitis is not higher than 3/1142 patients (0.3%), which represents the estimated frequency of life-threatening pneumonitis in the pooled population.

The time from initiation of treatment with osimertinib to onset of ILD ranged from 14 days to 230 days (median 54 days).

Treatment with the study drug was discontinued in 33 out of 35 patients with reported ILD, as per protocol. The majority of these patients were treated according to local clinical practice, with corticosteroids and often in association with antibiotics to treat the differential diagnosis of respiratory infection.

Patients with a past medical history of ILD, drug-induced ILD, radiation pneumonitis which required steroid treatment, or any evidence of clinically active ILD will be excluded from participation in this trial (see arm-specific exclusion criteria).

If new or worsening pulmonary symptoms (e.g. dyspnoea) or radiological abnormality suggestive of ILD is observed, an interruption in study treatment dosing is recommended, and the National Lung Matrix Trial Office should be informed. It is strongly recommended to perform a full diagnostic workup, to exclude alternative causes such as lymphangitic carcinomatosis, infection, allergy, cardiogenic oedema, or pulmonary haemorrhage. In the presence of confirmatory HRCT scans where other causes of respiratory symptoms have been excluded, a diagnosis of ILD should be considered and study treatment permanently discontinued.

In the absence of a diagnosis of ILD, study treatment may be restarted following consultation with the National Lung Matrix Trial Office. Refer to Section 28.3.4.3 for more detail on management of ILD and pneumonitis.

28.3.3.1.6 Liver effects

In the Phase II studies, the most commonly reported hepatobiliary-related AEs were ALT increased in 6.6% (27/411) of patients and hyperbilirubinaemia in 2.4% (10/411) of patients. Less than 20% of patients had abnormal hepatic biochemistry values for any individual parameter. Analysis of reported hepatobiliary events/abnormal hepatic biochemistry events

have been considered in conjunction with hepatic laboratory data in the Phase II studies. The overall assessment did not identify an increased risk of drug-induced liver injury.

Patients with any evidence of severe or uncontrolled systemic liver disease, including those with known hepatitis B, hepatitis C, human immunodeficiency virus (HIV) or abnormal liver enzymes (defined as AST or ALT >2.5 x upper limit of normal (ULN), total bilirubin >1.5 x ULN if no evidence of liver metastases; AST or ALT >5 x ULN, total bilirubin >3 x ULN in the presence of liver metastases) at screening are excluded from participating in the study (see core and arm-specific eligibility criteria). During the study, liver function tests will be monitored regularly during the study and at discontinuation. Patients' laboratory results will be assessed against the FDA's Draft Guidance for Drug Induced Liver Injury (FDA Guidance 2009).

28.3.3.1.7 Haematopoietic effects

Patients with inadequate bone marrow reserve as demonstrated by any of the following laboratory values (absolute neutrophil count < 1.5 x 10^9/L; platelet count < 100 x 10^9/L; haemoglobin < 90 g/L) will be excluded from the study (see core exclusion criteria).

In the Phase II studies, the most commonly reported PT was platelet count decreased (11.4% of patients), mainly CTCAE Grade 1 (41/47 patients). Thrombocytopenia was observed in 5.4% (22/411) of patients with the majority of patients having events of CTCAE Grade 1 (14/22 patients). Anaemia was observed in 9.7% (40/411) of patients, and white blood cell count decreased in 7.5% (31/411) of patients. CTCAE Grade 3 events were reported by <2% of patients per PT and there were two patients with CTCAE Grade 4 toxicities. There was one patient with an AE of pancytopenia, which was reported as mild by the investigator.

A decrease in the median value of platelets, neutrophils, lymphocytes and leucocytes has been seen early with osimertinib treatment. At a population level, stabilisation occurs towards the lower limit of normal and the effect is not thought to be of clinical significance. The majority of patients experiencing a change see only a single CTCAE grade shift from baseline. The types and frequency of bleeding or infection events reported are considered consistent with what would be expected in an advanced cancer population.

AEs of leukopenia, lymphopenia, neutropenia and thrombocytopenia have been reported, most of which were mild or moderate in severity and did not lead to dose interruptions. Haematological parameters will be monitored prior to administration of the first dose, weekly during the first cycle, at the start of each subsequent cycle, and at discontinuation.

28.3.3.1.8 Reproductive organ effects

Findings in the repeat dose toxicology studies in rats and dogs indicate that osimertinib could impair male and female fertility. In the 1 month rat study, pathology findings in the ovary and testes were not present following 1 month off-dose, indicating reversibility. It is not possible to make a definitive statement regarding recovery of the testicular pathology seen in the 1 month dog study (due to the absence of testicular pathology in high dose animals at the end of the 1 month dosing period).

Osimertinib was associated with a reversible increase in post-implantation loss in the rat female fertility study. Non-clinical studies have demonstrated that osimertinib has marked adverse effects on embryonic survival plus early postnatal viability and growth when administered to pregnant or lactating rats. This is consistent with the expected effects of an EGFR inhibitor. In addition there was evidence suggestive of osimertinib and its metabolite being excreted in milk. Although there have been no adverse observations related to reprotoxicity potential noted to date in clinical trials, these data indicate the potential for adverse effects if osimertinib was administered to patients who are pregnant or breast feeding.

Therefore women of child bearing potential and all men will be required to use adequate contraceptive measures during the study and for an appropriate period thereafter. Patients will be fully informed of the effects seen on the reproductive organs identified pre-clinically. Women of child bearing potential must have a negative pregnancy test prior to first dose of study treatment. Women who are breast feeding will be excluded from participating in the study. Male patients will be advised to arrange for the freezing of sperm samples prior to the start of the study should they wish to father children, and not to donate sperm until 6 months after discontinuation of study treatment.

28.3.3.1.9 Exposure in the osimertinib programme

As of 01 October 2015, at least 1258 subjects have been exposed to treatment with osimertinib, at dose levels ranging from 20 mg to 240 mg. Of the 1258 subjects, 107 were exposed to a combination of osimertinib plus another treatment (24 patients in combination with savolitinib, 34 patients in combination with durvalumab, and 49 patients in combination with selumetinib). Overall subject exposure included 714 subjects who participated in Phase I studies (this included all Clinical Pharmacology studies conducted in patients) and 544 subjects who participated in the Phase II programme.

In addition to the 1258 subjects, 393 subjects have received treatment (osimertinib or comparator) in Study D5160C00003, a Phase III randomized study of osimertinib versus platinum-based doublet chemotherapy (2:1 randomisation); 251 subjects have received treatment (osimertinib or comparator) in Study D5160C00007, a Phase III randomised study of osimertinib versus a SoC EGFR TKI (1:1 randomisation); 13 subjects have received osimertinib alone or in combination with durvalumab in Study D5165C00001.

Three compassionate use programmes for osimertinib are currently ongoing and, as of 01 October 2015, 372 subjects had received treatment with osimertinib.

28.3.3.1.10 Safety

The most common adverse events (AEs) have been diarrhoea, rash (grouped term), paronychia and dry skin. Most of these events have been of mild to moderate intensity.

At the data cut-off date of 01 May 2015 for AURA Phase 1, AURA extension and AURA2, most of the deaths in the osimertinib Phase II studies were considered to be due to the disease under investigation only.

In total, 13 patients (3.2%) died due to AEs across both Phase II studies (AURA extension and AURA2). In 8 patients, the Investigator considered the death to be due to both NSCLC (disease under investigation) and a fatal AE. In the other 5 patients, the death was considered to be as a consequence of the AEs alone. Of these 13 patients, 4 experienced AEs leading to death that were considered by the Investigator to be possibly causally related to treatment with osimertinib (3 patients had AEs of interstitial lung disease (ILD), and 1 patient had an AE of pneumonitis). In AURA Phase I, there were 12/271 (4.4%) patients who had fatal AEs. The most common was pneumonia (6 patients); no other AE that led to death was reported in more than 1 patient.

The most common fatal AE across the clinical programme was pneumonia, which is not unexpected in an advanced NSCLC population. The total incidence of events and the number of CTCAE grade 5 (fatal) events observed are as expected in this patient population and do not constitute a safety signal.

ILD-like events have been observed in patients receiving osimertinib and 4 cases across the clinical programme have been fatal (see the IB for further discussion of ILD).

The most commonly reported SAEs were as expected in a population of patients with advanced NSCLC. The most common SAEs were pneumonia, pulmonary embolism, pleural effusion and pneumonitis. SAEs that were considered as being possibly related to study drug included: pneumonitis, ILD, diarrhoea, decreased appetite, pulmonary embolism and thrombocytopenia.

The most common AE leading to discontinuation were ILD/pneumonitis. Note that permanent discontinuation was a protocol requirement for all confirmed ILD-like cases.

28.3.4 Dose Modifications & Toxicity Management

28.3.4.1 Treatment delays and discontinuation

If a patient experiences a CTCAE grade 3 or higher and/or unacceptable toxicity (any grade) not attributable to the disease or disease-related processes under investigation, where the local Investigator considers the AE of concern to be specifically associated with osimertinib, dosing will be interrupted and supportive therapy administered as required in accordance with local practice/guidelines.

If the toxicity resolves or reverts to ≤CTCAE grade 2 within 3 weeks of onset, treatment with osimertinib may be restarted at the same dose (80 mg) or a lower dose (40 mg) using the rules below for dose modifications.

If the toxicity does not resolve to ≤CTCAE Grade 2 after 3 weeks, then the Investigator should consider permanent treatment discontinuation on consultation with the National Lung Matrix Trial Office, and the patient should be observed until resolution of the toxicity.

In the event of a treatment interruption for reasons other than treatment related toxicity (e.g., non-cancer related surgery) lasting >3 weeks, treatment resumption will be decided on consultation with the National Lung Matrix Trial Office. It is advised that treatment delays should not exceed 6 weeks. Specific guidance relating to diarrhoea and ophthalmic dose adjustments can be found in Section 28.3.4.3.

28.3.4.2 Dose reductions

There will be no individual modifications to dosing schedule in response to toxicity, only potential dose reduction or dose interruption.

Table 66: Dose intervention for osimertinib

**Intervention**	**Osimertinib Dose**
Starting Dose	80 mg
Reduced Dose –1	40 mg

On resolution of toxicity within 3 weeks:

If an AE subsequently requires dose interruption, osimertinib may restart at the same dose or the reduced dose, on resolution/improvement of the AE at the discretion of the Investigator.

If new or worsening pulmonary symptoms (e.g., dyspnoea) or radiological abnormality suggestive of interstitial lung disease (ILD) is observed, an interruption in study treatment dosing is recommended. It is strongly recommended to perform a full diagnostic workup (including high-resolution computed tomography (HRCT), blood and sputum culture, haematological parameters), to exclude alternative causes such as lymphangitic carcinomatosis, infection, allergy, cardiogenic oedema, or pulmonary haemorrhage. In the presence of confirmatory HRCT scans where other causes of respiratory symptoms have been excluded, a diagnosis of interstitial lung disease should be considered and study treatment permanently discontinued.

In the absence of a diagnosis of interstitial lung disease study treatment may be restarted if felt to be in the best interests of the patient. See Figure 28.

Patients with QTcF prolongation fulfilling the following criteria (i.e. confirmed QTcF prolongation to > 500 msec absolute or a > 60 msec increase from baseline) should have study treatment interrupted and regular ECGs performed until resolution to baseline. If the toxicity does not resolve to < grade 1 within 3 weeks the patient will be permanently withdrawn from study treatment.

No dose adjustment is recommended in patients with mild, moderate, or severe renal impairment. However, as patients with severe renal impairment may have a higher osimertinib mean exposure, cautious use in patients with severe and end stage renal disease continues to be recommended.

Patients experiencing corneal ulceration or ILD will not be permitted to restart study treatment.

No within-patient dose re-escalation will be permitted.

28.3.4.3 Evaluation, Management and Treatment of Toxicities

The purpose of these treatment guidelines is to prevent tolerable AEs becoming intolerable for the patient and leading to discontinuation of treatment, and to promote consistency of treatment for specific adverse events across studies using osimertinib. This section provides advice for the following adverse events:

- Skin effects (rash, dry skin/xerosis, pruritus, paronychia)
- Diarrhoea
- Visual symptoms
- Interstitial lung disease & pneumonitis

28.3.4.3.1 <u>Skin effects</u>

Skin effects may occur at any time, but will most likely start within 2 weeks of study treatment commencement. Patients may consider applying over-the-counter moisturising cream to face, hands and feet twice daily from the start of study.

Table 67: Osimertinib skin effects - rash treatment guidance

CTCAE Grade	AE description	Treatment guidance
1	• <10% body surface area (BSA) papules/pustules<br>• with or without symptoms of pruritus or tenderness	Emollient cream application and/or topical steroid moderate strength bid and/or topical antibiotic bid
2	• 10 to 30% BSA papules/ pustules with or without symptoms of pruritus or tenderness<br>• psychosocial impact<br>• limiting instrumental activities of daily living (ADL)	Treatment same as Grade 1<br>Consider using oral antibiotic for 6 weeks
≥3	• >30% BSA papules/pustules with or without symptoms of pruritus or tenderness<br>• limiting self-care ADL<br>• associated with local superinfection	Topical steroid moderate strength bid and oral antibiotic for 6 weeks<br>Switch to broad spectrum/gram negative cover if infection suspected (yellow crusts, purulent discharge, painful skin / nares)<br>Consider skin swab for bacterial culture

Table 68: Osimertinib skin effects - dry skin/xerosis treatment guidance

CTCAE Grade	AE description	Treatment guidance
1	• <10% body surface area (BSA)<br>• No associated erythema or pruritus	Face/Hands/Feet: over-the-counter moisturising cream or ointment bid<br>Body: ammonium lactate 12% cream bid or salicylic acid 6% cream bid
2	• 10 to 30% BSA<br>• Associated with erythema or pruritus<br>• Limiting instrumental activities of daily living (ADL)	Treatment same as Grade 1
≥3	• >30% BSA<br>• Associated with erythema or pruritus<br>• Limiting self-care ADL	Treatment same as Grade 1/2, plus:<br>Eczematous areas of body: topical steroid moderate strength bid

Table 69: Osimertinib skin effects - pruritus treatment guidance

CTCAE Grade	AE description	Treatment guidance
1	• Mild or localised<br>• Topical intervention indicated	Topical steroid moderate strength bid or topical antipruritic bid
2	• Intense or widespread<br>• Intermittent<br>• Skin changes from scratching (e.g. oedema, papulation, excoriation, lichenification, oozing/crusts)<br>• Oral intervention indicated<br>• Limiting instrumental ADL	Topical steroid moderate strength bid or topical antipruritic bid<br>Oral antihistamine
≥3	• Intense or widespread<br>• Limiting self-care ADL or sleep<br>• Oral corticosteroid or immunosuppressive therapy indicated	Oral antihistamine<br>GABA agonist (gabapentin 300 mg or pregabalin 50-75 mg every 8 hours)

Table 70: Osimertinib skin effects - paronychia treatment guidance

CTCAE Grade	AE description	Treatment guidance
1	• Nail fold oedema or erythema<br>• Disruption of the cuticle	Topical antibiotic bid and vinegar soaks*
2	• Localised intervention indicated<br>• Nail fold oedema or eythema with pain<br>• Associated with discharge or nail plate separation<br>• Limiting instrumental activities of daily living (ADL)	Topical antibiotic bid and vinegar soaks*<br>Topical silver nitrate weekly
≥3	• Surgical intervention or IV antibiotics indicated<br>• Limiting self-care ADL	Topical antibiotic bid and vinegar soaks*<br>Topical silver nitrate weekly<br>Consider nail avulsion / removal

*Soaking fingers or toes in a 1:1 solution of white vinegar in water for 15 minutes every day

<u>Key points for dermatological guidance</u>

- Patients should be encouraged to report any instances of skin reaction as soon as they arise so that appropriate treatment can be promptly initiated.

- Investigators may consider issuing a prescription for topical treatment to patients, however, topical steroids and topical or oral antibiotics should not be implemented prophylactically and treatment should only be started when confirmed with Investigator.
- As soon as an acneiform / papulopustular rash occurs, treatment with moderate strength topical steroids and antibiotics should be implemented.
- Use of topical benzoyl peroxides and other irritating anti-acne agents should be avoided.
- Patients should be instructed to contact the site study team if the skin reaction changes (e.g. if it spreads or becomes painful)
- The occurrence of non-papulopustular skin reactions should be treated appropriately, as defined by the treating physician and in consultation with a dermatologist where necessary.
- Patients may be advised to avoid irritating skin producs (e.g. irritating soaps, products containing retinol or retinoic acid).
- Camouflage makeup (non-comedogenic or non-pore blocking) can be used during study treatment.

28.3.4.3.2 Diarrhoea

Table 71: Osimertinib related diarrhoea: dietetic measures and pharmacological treatment

CTCAE Grade	Dietetic measures	Pharmacological treatment
Uncomplicated CTCAE Grade <2	• Stop all lactose-containing products<br>• Drink 8 to 10 large glasses of clear liquids per day<br>• Eat frequent small meals<br>• Recommend low fat regimen enriched with rice, bananas, and apple sauce	Administer loperamide: initial dose 4mg, followed by 2mg every 4 hours or after every unformed stool.<br>Grade 1 intermittent diarrhoea may not require treatment<br>Consider continuation of loperamide until diarrhoea-free for 12h<br>Consider electrolyte replacement, as appropriate
Grade >3 or any Grade with complications (dehydration, fever and/or Grade >3 neutropenia)	• As per Grade <2 diarrhoea	As per Grade <2 diarrhoea<br>If dehydration is severe, administer octreotide and use intravenous fluids as appropiate.<br>Consider prophylactic antibiotics, especially if diarrhoea is persistent beyond 24h or there is fever or Grade 3-4 neutropenia<br>Consider electrolyte replacement, as appropriate, and consider more frequent measurement of electrolytes until AE resolves

Key points for diarrhoea guidance
Patients should be encouraged to report any instances of diarrhoea as soon as they arise so that appropriate treatment can be promptly initiated.

28.3.4.3.3 Visual symptoms

Patients presenting with signs and symptoms suggestive of keratitis such as acute or worsening: eye inflammation, lacrimation, light sensitivity, blurred vision, eye pain and/or red eye should be referred promptly to an ophthalmology specialist.

28.3.4.3.4 Interstitial lung disease (ILD) & Pneumonitis

Figure 28: Osimertinib - ILD and Pneumonitis Guidance Flowchart

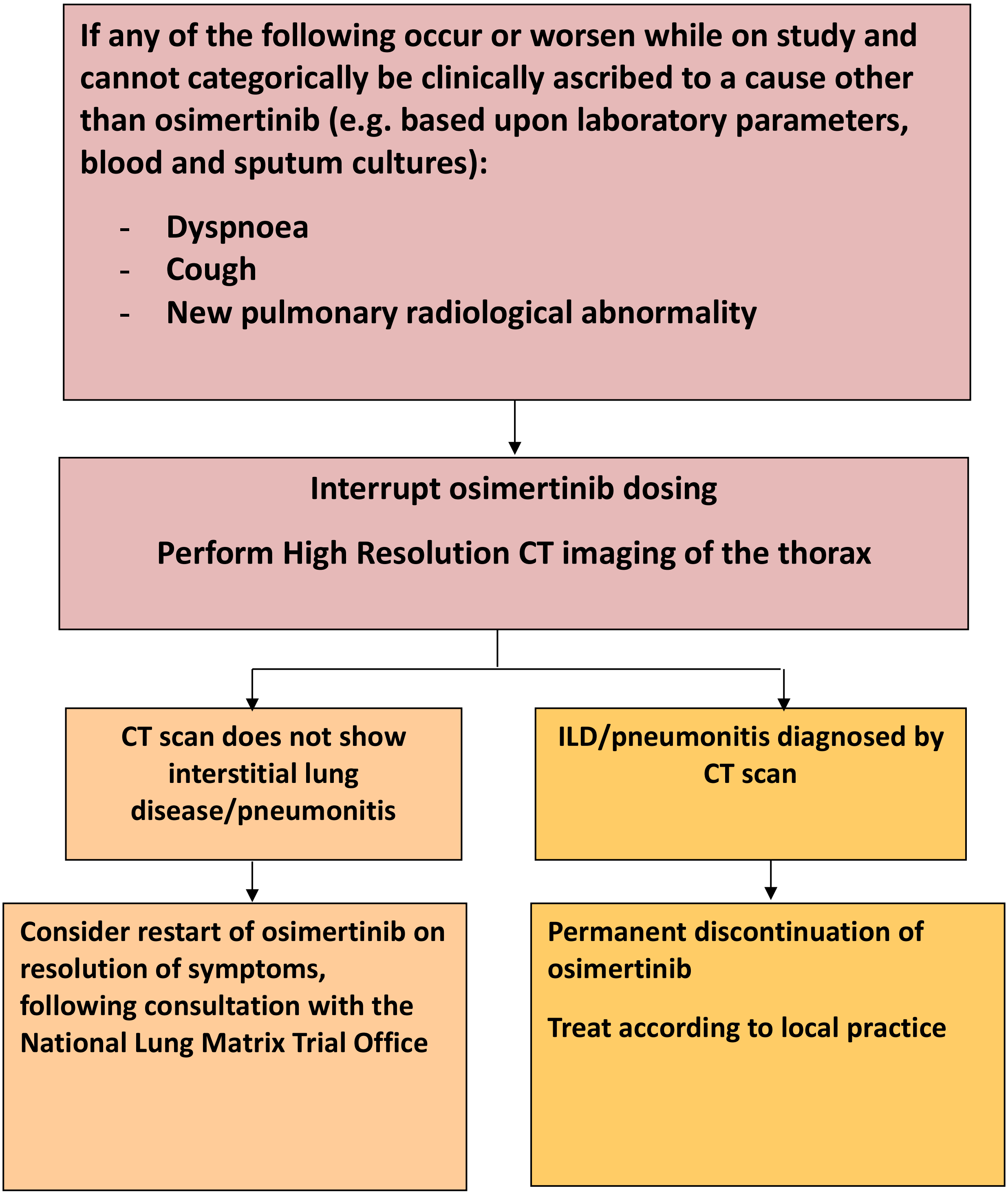

28.4 References

- FDA Guidance for Industry (issued July 2009) 'Drug-induced liver injury: Premarketing clinical evaluation'. Available from URL: http://www.fda.gov/downloads/Drugs/GuidanceComplianceRegulatoryInformation/Guidances/UCM174090.pdf. Accessed 19 June 2015.
- Heuckmann JM, Rauh D, Thomas RK (2012). Epidermal growth factor receptor (EGFR) signaling and covalent EGFR inhibition in lung cancer. *J Clin Oncol*, **30(27):**3417-20.
- Jackman D, Pao W, Riely GJ *et al.* (2010). Clinical definition of acquired resistance to epidermal growth factor receptor tyrosine kinase inhibitors in non-small-cell lung cancer. *J Clin Oncol* **28(2):** 357-360.
- Jänne PA, Yang JC-H, Kim D-W *et al.* (2015). AZD9291 in EGFR Inhibitor–Resistant Non–Small-Cell Lung Cancer. *N Engl J Med* **372:**1689-169
- National Comprehensive Cancer Network Guidelines for Treatment of Cancer by Site. 2012. Available from URL: http://www.nccn.org/professionals/physician_gls/f_guidelines.asp#site
- Pao W, Miller VA, Politi KA *et al.* (2005). Acquired resistance of lung adenocarcinomas to gefitinib or erlotinib is associated with a second mutation in the EGFR kinase domain. PloS Med **2(3):**e73

29 ARM H: SITRAVATINIB (MGCD516) - VEGFR INHIBITOR *(CLOSED TO RECRUITMENT)*

Lead Investigator: Professor Gary Middleton

29.1 Background & Rationale

29.1.1 Molecular Cohorts

Inhibitor: Sitravatinib (MGCD516)

Arm	Investigational Medicinal Product	Cohort Number	NSCLC Histology	Molecular Cohort
H	Sitravatinib	H1	NSCLC	RET Rearrangements

Sitravatinib (MGCD516) is an orally available, potent, small molecular inhibitor of a closely related spectrum of tyrosine kinases including MET, Axl, VEGFR family, platelet-derived growth factor receptor (PDGFR) family, KIT, fms like tyrosine kinase 3 (FLT3), tropomyosin receptor kinase (Trk) family, RET, Discoidin domain-containing receptor 2 (DDR2) and selected Eph family members. This subset of tyrosine kinases is involved in a number of processes implicated in human cancer including regulation of tumour growth and cell survival pathways, tumour invasion and metastatic progression, as well as tumour angiogenesis.

29.1.2 Pre-Clinical Rationale

Sitravatinib has been studied in a variety of *in vitro* and *in vivo* model systems to determine potency for inhibition of RTK targets, kinase selectivity, anti-tumour efficacy, and PK/PD relationships. Sitravatinib demonstrated potent, concentration-dependent inhibition of the kinase activity of MET, Axl family, VEGFR family, PDGFR family, KIT, FLT3, Trk family, RET, DDR2, and selected Eph family members in biochemical assays, and inhibited phosphorylation and kinase dependent function in cell-based assays. Sitravatinib also inhibited oncogenic functions associated with target RTKs including MET-dependent cell viability and migration and endothelial tube formation and angiogenesis. Consistent with this anti-tumour and anti-angiogenic mechanism of action, sitravatinib demonstrated anti-tumour efficacy over a broad spectrum of human tumour xenograft models including robust cytoreductive anti-tumour activity in a subset of models exhibiting genetic alterations in RTK targets including MET, RET, FLT3, platelet-derived growth factor receptor A (PDGFRA), and others.

Sitravatinib also demonstrated inhibition of MET, Axl, RET, and/or ephrin type-A receptor 2 (EPHA2) activity in selected tumour xenograft models *in vivo* and a PK/PD relationship of plasma concentration of sitravatinib to anti-tumour activity was established in defining target plasma exposures utilised for projection of target human efficacious plasma levels and dose.

29.1.3 Clinical Data

29.1.3.1 516-001 Study

The first-in-human study to evaluate sitravatinib is the 516-001 study. This is a multi-centre, phase I/Ib clinical trial characterising the safety, PK, PD and clinical activity of sitravatinib in patients with advanced solid tumour malignancies. Sitravatinib is being evaluated as a single agent administered orally once daily without interruption in cycles of 21 days.

As of 26 June 2017, patient data is available for 86 patients enrolled into the 516-001 study (49 men / 37 women; median age 66 years; range 27-85 years) with advanced solid tumour malignancies (18 renal carcinoma, 16 NSCLC, 13 prostate cancer, 7 soft tissue sarcoma, 6 colon cancer, 26 other). The phase I dose escalation has been completed. Phase Ib enrolment of patients with selected histological diagnoses and/or molecular markers to further assess the safety of sitravatinib at 150 mg QD and to evaluate clinical activity is currently ongoing.

29.1.3.2 MRTX-500 study

Study MRTX-500 is a parallel phase 2 study of glesatinib, sitravatinib or mocetinostat in combination with nivolumab in advanced or metastatic non-squamous NSCLC who have experienced progression of disease on or after treatment with a checkpoint inhibitor. This trial is currently open and enrolling patients in the sitravatinib arm.

As of 26 June 2017, patient data were entered in the clinical trial database for 10 patients (2 men, 8 women, median age 69.5 years, range 49-76 years) with advanced or metastatic NSCLC.

29.1.3.3 AAAQ8661 study

This is an ongoing phase II, single-arm, open-label, Simon optimal 2-stage, Investigator-Sponsored study evaluating the efficacy of sitravatinib in patients with unresectable or metastatic well-differentiated or de-differentiated liposarcoma who have received treatment with at least one prior systemic therapy and demonstrate evidence of disease progression prior to study enrollment. Following the Simon optimal 2-stage design, the study will enrol 13 liposarcoma patients in the first stage. If 3 or more liposarcoma patients meet the Progression Free Rate (PFR) endpoint during the first stage, the study will be expanded to a total of 29 liposarcoma patients. The study is open at two institutions in the USA. All subjects are treated with the same agent and dose of sitravatinib, at 150 mg once per day in continuous 21 day cycles. Patients receive treatment at the discretion of the Principal Investigator until disease progression, unacceptable toxicity or adverse event(s) or withdrawal of consent.

29.1.3.4 2016-0332 study

Study 2016-0332 is a phase I–II dose-finding trial to determine the optimal dose of sitravatinib when combined with immunotherapy nivolumab at fixed dose 240 mg IV every 2 weeks, in patients with metastatic renal cell carcinoma with a clear cell component who have progressed following treatment with anti-VEGF agents. The goal of the study is to determine an optimal oral sitravatinib dose level among the four levels 60, 80, 120 and 150 mg/day given orally daily until toxicity or progressive disease. Dose-finding is using the sequentially adaptive phase I-II late-onset EffTox (Lo-EffTox) trade-off-based design of Jin et al. A maximum of 60 patients will be enrolled in this trial. The study initiated at the 80 mg/day dose level of sitravatinib. As of 30 December 2017, the 120 mg/day dose level of sitravatinib is enrolling. Patients receive treatment at the discretion of the Principal Investigator until disease progression, unacceptable toxicity or adverse event(s) or withdrawal of consent.

29.1.3.5 Efficacy

As of a data cut-off date of 9 December 2016, a total of six NSCLC patients with RET rearrangements had been enrolled into the 516-001 study, four of whom were evaluable for response.

Of the four evaluable patients, one patient with a KIF5B-RET fusion demonstrated a confirmed partial response; one patient with a DSP-RET fusion achieved a partial response on an initial scan, and a third patient with a RET fusion demonstrated tumour reduction of 29%,

representing stable disease. Reduction in tumour size was observed in all four patients. Enrolment of patients with RET rearrangement, chromosome 4q12 amplification, and CBL mutation is ongoing.

As of 08 January 2018, no further efficacy data is available for other studies using sitravatinib.

29.1.3.6 Safety

As of a data cut-off date of 26 June 2017, safety data is available for 86 patients with advanced malignancies in Study 516-001 and 10 patients with NSCLC in Study MRTX-500.

29.1.3.6.1 516-001 study

In Study 516-001, the Phase 1 dose escalation has been completed. At the 80 mg dose level, 1 DLT (Grade 3 palmar-plantar erythrodysesthesia) was observed, and at the 200 mg dose level, 3 DLTs were observed (intolerable Grade 2 neuropathy, intolerable Grade 2 fatigue and intolerable Grade 2 mucositis in 1 patient each), demonstrating 200 mg exceeded the MTD. Phase 1b cohorts were opened with sitravatinib administered at 150 mg once daily in 21-day cycles.

The most commonly observed adverse events (AEs) reported as related to sitravatinib treatment were fatigue (44%), diarrhoea (35%), hypertension (34%), nausea (24%), decreased appetite (22%), vomiting (22%), decreased weight (17%), palmar-plantar erythrodysesthesia (13%) and hypothyroidism (12%). AEs of Grade 3 that were reported as related to treatment in more than one patient included hypertension in 16 patients (19%), diarrhoea in 9 patients (11%), pulmonary embolism, increased lipase and fatigue in 4 patients each (5%), palmar-plantar erythrodysesthesia, decreased ejection fraction, left ventricular dysfunction, mucosal inflammation, and hyponatraemia in 2 patients each (2%). Three Grade 4 AEs, febrile neutropenia, hypotension and tachycardia in 1 patient each (1%), were reported as related to sitravatinib.

Phase 1b enrolment of patients having tumours with selected histological diagnoses and/or molecular markers to further assess the safety of sitravatinib at 150 mg OD and to evaluate clinical activity, is currently ongoing.

As of 26 June 2017, twenty-six deaths were reported in this study, with the primary cause of death being the disease under study (n=17), unknown (n=5), hypoxic respiratory failure, respiratory failure, GI bleed or sepsis (n=1 each).

SUSARs reported for this study as of 19 March 2018 include one fatal cardiac arrest (possibly related to sitravatinib) and one episode of Grade 3 headache (possibly related to sitravatinib).

29.1.3.6.2 MRTX-500 study

Study MRTX-500 began with a lead-in evaluation of sitravatinib in combination with nivolumab in a small cohort of patients. The starting dose of the lead-in cohort was 120 mg QD of sitravatinib. No protocol defined DLTs were reported in the first 6 evaluable patients treated at 120 mg QD which was selected as the recommended Phase 2 dose (RP2D).

As of the data cut-off date, the most commonly observed AEs reported (≥20%) as related to sitravatinib were; fatigue (50%), diarrhoea (40%), aspartate aminotransferase increase (30%), palmar-plantar erythrodysaesthesia (30%), dysphonia (30%), lipase increase (20%), weight decrease (20%), nausea (20%), vomiting (20%), decreased appetite (20%), hyponatremia (20%), hypertension (20%) and hypothyroidism (20%).

Among the 10 patients with available safety data, one patient (10%) experienced at least one serious sitravatinib-related treatment emergent adverse event (SAE) including pulmonary embolism and deep-vein thrombosis.

As of 26 June 2017, no patient deaths have been reported in this study.

SUSARs reported for this study as of 19 March 2018 include one patient with pulmonary embolism and non-occlusive DVT (possibly related to sitravatinib) .

29.1.3.6.3 AAAQ8661 study

SUSARs reported for this study as of 28 November 2017 include decrease in left ventricular ejection fraction (probably related to sitravatinib). No additional safety data is currently available.

29.1.3.6.4 2016-0332 study

SUSARs reported for this study as of 19 March 2018 include one patient with grade 3 pancreatitis (probably related to sitravatinib and nivolumab). No additional safety data is currently available.

29.1.3.7 Pharmacokinetics

The PK profile of sitravatinib has been evaluated in study 516-001 after single and repeated dose administration. Plasma samples for PK analyses were collected over a 168-hour period following single dose administration and over a 24-hour period following repeated dose administration. Plasma drug concentrations were determined using a validated, sensitive, liquid chromatography-mass spectrometry (LC-MS)/MS assay. The pharmacokinetics of sitravatinib was evaluated using non-compartmental analysis methods.

After single dose administration, sitravatinib reaches peak concentration in a median time of 3 to 9 hours. Exposure parameters (maximum concentration [C_{max}] and area under the curve [AUC]) are approximately dose proportional.. Mean elimination half-life varies between 40 and 53 hours after oral administration. The steady state PK is reached in a mean time of 11 to 15 days. Drug accumulation is observed after multiple dose administration and averaged 2.6-fold for C_{max} and 3.1-fold for AUC_{0-24}. The 150 mg dose was determined to be the recommended phase 2 dose (RP2D), which results in a steady state geometric mean C_{avg}, C_{max} and AUC_{0-24} of 87.1ng/mL, 107 ng/mL and 2090 ng•h/mL, respectively. Following determination of the RP2D, an evaluation of patient data suggests that adequate blood levels of sitravatinib can still be maintained with the lower dose, with a potential for better adherence and improved tolerability. Therefore the recommended phase II dose was adjusted to 120 mg which will apply to all ongoing and new trials using sitravatinib.

29.1.3.8 Pharmacodynamics

Pharmacodynamic effects of sitravatinib treatment were examined by analysing vascular endothelial growth factor (VEGF)-A, soluble VEGFR2 and soluble MET ectodomain (sMET) levels in patients' plasma samples collected before and after sitravatinib administration. The assessment of these soluble protein markers in clinical trials of known VEGF or MET pathway inhibitors (e.g., sunitinib, pazopanib, crizotinib, and cabozantinib) has been useful in proof of concept for the targets. PD assessments are still ongoing for Study 516-001, but preliminary PD analysis showed a concentration dependent modulation of each bio-marker.

The mean prediction (95% CI) percent change from baseline in biomarker modulation with exposure, approached: VEGF-A (300% increase), sVEGFR2 (50% decrease) and sMET (35%

increase), consistent with VEGFR and MET inhibition. Average sitravatinib pre-dose steady state concentration values observed in patients receiving 120 mg QD is 70.8 ng/mL. At these sitravatinib plasma exposure levels, near optimal modulation of biomarker levels is expected.

29.1.3.9 Marketing Experience

Sitravatinib is not approved for marketing in any region of the world.

29.1.4 Cohort Definition

29.1.4.1 H1: RET Rearrangement

In cohort H1, NSCLC patients with a tumour RET fusion resulting from gene rearrangements that lead to the juxtaposition of non-RET genetic sequences proximal to the RET kinase domain are eligible. [Identification of RET rearrangements by FISH is also accepted.] These RET fusions include KIF5B-RET, coiled-coil domain-containing protein 6 (CCDC6)-RET, and nuclear receptor coactivator 4 (NCOA4)-RET. Other RET fusions may also be eligible.

RET rearrangement occurs in 1-2% of adenocarcinoma NSCLC cancer patients and results in a RET fusion. The incidence is higher among younger patients, female patients, and non-smokers. All cases of RET fusions were reported in adenocarcinomas with no particular subtype of adenocarcinoma showing a higher incidence of RET fusion (4). The RET gene rearrangement does not appear to be associated with overall survival.

The most common fusion, KIF5B-RET (85%) is formed by pericentric inversion on chromosome 10. This rearrangement leads to juxtaposition of the coiled coil domain of KIF5B to the RET kinase domain, resulting in the former mediating homodimerisation and autophosphorylation and hence constitutive activation of the RET tyrosine kinase. Expression of KIF5B-RET leads to transformation and anchorage-independent growth, which is inhibited by RET inhibition.

Preclinical proof of concept for the activity of sitravatinib in RET fusion disease is using a PDX model developed from a patient harbouring a KIF5B-RET fusion. In this model, treatment with 200 mg/kg daily of sitravatinib resulted in complete cessation of growth (see Figure 29).

Figure 29: Proof of concept anti-tumour efficacy in NSCLC models exhibiting RTK target alterations (10)

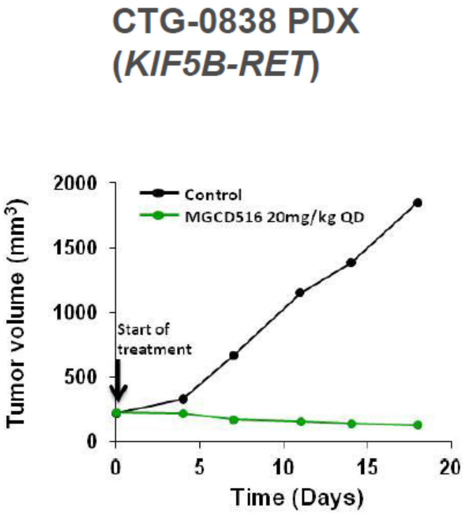

Case series and case reports of patients with NSCLC characterised by RET fusions and who were treated various RET inhibitors have also clearly shown clinical activity. In a study of cabozantinib, the authors enriched a cohort of lifelong non-smokers who had no activating tumour mutations of EGFR and no rearrangement of ALK or ROS to find a RET fusion rate of 16%. Among 3 patients treated, 1 had a partial response (PR) which was ongoing at 5 months; the second patient also had a PR out to 16 weeks; the third patient had stable disease after nearly 8 months of ongoing treatment (5). In another series of 3 patients from the Cleveland Clinic, 1 patient treated with cabozantinib had an impressive metabolic response by positron emission tomography (PET) scanning but needed to stop drug because of severe stomatitis, and after cessation of the drug, rapid progression and death ensued (6). A recent case report of a patient with a CCDC6-RET fusion showed a 76% decrease in the sum of the maximum dimensions of their tumour which remained ongoing after 4 months (7). A patient with a KIF5B fusion treaTed with sunitinib (a multi-tyrosine kinase inhibitor with RET inhibitory activity) also had a good response to treatment but drug discontinuation due to toxicity preceded rapid progression and death (8). Finally in 3 patients treated with sorafenib there was a long term stabilisation of disease for 12 months in a single patient (9).

Sitravatinib is a broad spectrum multi-targeted receptor tyrosine kinase inhibitor that inhibits signalling from several tyrosine kinases, including RET and VEGFR. The in vitro IC50 for RET is 44 nmol/L, which is more potent than the commonly used RET inhibitor vandetanib (1,2), and plasma levels achieved in the phase I dosing of sitravatinib are the same as those causing regression in the PDX model.

29.2 Specific Eligibility Criteria

29.2.1 Inclusion Criteria

- Patients must fulfil all the core eligibility criteria.
- Technology hub result (or locally obtained result from an approved Laboratory if applicable.
- Eastern Cooperative Oncology Group (ECOG) Performance Status $\leq$2 with no deterioration over previous 2 weeks (see Appendix 8).
- Ability to swallow oral medication.
- Adequate hepatic function in patients **with** liver metastasis: AST and/or ALT $\leq$ 5.0 x ULN

29.2.2 Exclusion Criteria

Patients must not enter the trial if any of the following exclusion criteria are fulfilled:

- Patients who do not fulfil all the core eligibility criteria
- Treatment with any of the following:
  - Concomitant use of medications known to prolong QT interval within 6 half-lives plus 1 day prior to the first dose of sitravatinib (see Section 29.2.3.1).
  - Concomitant use of medications known to be sensitive substrates or substrates with a narrow therapeutic index for P-gp and BCRP transporters within 5 half-lives prior to the first dose of sitravatinib (see drugs that are listed as prohibited in Table 73 of Section 29.2.3.3).
  - Drugs that are Cytochrome P450 (CYP) substrates within 5 half-lives prior to the first dose of sitravatinib (listed as prohibited in
  - Table 72 of Section 29.2.3.2)
  - Previous treatment with a therapy targeting the molecular marker of interest in the cohort being considered for enrolment (e.g. treatment with cabozantinib or sunitinib in cohort H1 RET fusions).
  - Any other chemotherapy, radiotherapy, investigational agents or other anti-cancer therapy within 2 weeks prior to trial treatment.
- Significant cardiac abnormalities within the past 6 months, such as myocardial infarction or congestive heart failure ≥ Class 3, and symptomatic or uncontrolled atrial fibrillation.
- Mean resting corrected QT interval (QTcF) >470 msec obtained from 3 consecutive ECGs.
- Left ventricular ejection fraction (LVEF) <40%.
- Squamous cell carcinoma patients with either:
  - tumour lesions near a major blood vessel or large (>2 cm) cavitating lesions *or*
  - A history of significant haemoptysis or haemorrhage within 4 weeks prior to treatment.
- Uncontrolled arterial hypertension (150 mm Hg systolic or > 100 mm Hg diastolic) on at least 2 separate observations despite standard of care treatment. Patients with isolated elevation in blood pressure (e.g. due to anxiety) should **not** be excluded.
- Patients with a sensitivity to any component of the drug:
  - A blend of MGCD516 (sitravatinib) free base drug substance
  - Microcrystalline cellulose (Avicel® PH302)
  - Polysorbate 80 (Tween® 80)
  - Aerosil® 200 Pharma

29.2.3 Restrictions & Concomitant Medications

Information on any treatment from the date of informed consent until 28 days after the administration of the last treatment dose should be recorded. If medically feasible, patients taking regular medication should be maintained on it throughout the study period. Patient should be advised to inform their treating physicians of all concomitant medications, including prescription medicines, over-the-counter drugs, vitamins, and herbal products.

Nb. These lists are not exhaustive and the absence of a drug from the lists does not imply that its combination with sitravatinib is safe.

29.2.3.1 QT Interval Prolongation

Concomitant medications **known to prolong the QT interval** are prohibited at registration, and during the active treatment phase. Any patients taking such drugs at or prior to registration should discontinue the drug 6 half-lives plus 1 day prior to commencing trial treatment.

For a list of drugs known to prolong the QT interval, (or with any risk of prolonging the QT interval), please refer to the following database: https://www.crediblemeds.org. Appendix 10 contains a list exported from this database on 2nd March 2018. **It is important to note that this list is a guide – the database will change with time and therefore needs to be checked in real-time when screening and registering a patient, and throughout their treatment.**

Please note that for this arm, only concomitant medications listed on https://www.crediblemeds.org under the category **'Known risk of TdP'** will exclude patients from entering the trial as per the eligibility criteria.

Concomitant medications listed under the categories **'possible risk of TdP'** or **'Conditional risk of TdP'** may be permitted with caution. Whilst additional monitoring is not mandated, the development of cardiac related signs and symptoms should be closely observed and acted upon.

It is the responsibility of an Investigator (Consultant level) to review and clinically evaluate all concomitant medications. Please contact the National Lung Matrix Trial Office for clarification regarding any drugs that appear on the database that are not listed in Appendix 10.

The drugs listed on this website are taken from information provided by The Arizona Centre for Education and Research on Therapeutics and The Critical Path Institute, Tucson, Arizona and Rockville, Maryland. Important Note - If a patient is being treated with such medication or is taking another medication that may affect QT interval which is not on the database, please contact the National Lung Matrix Trial Office to obtain the recommended withdrawal/ minimum period prior to starting trial treatment.

29.2.3.2 Cytochrome P450 (CYP) Substrates

In vitro experiments indicate that sitravatinib is a potential inducer of CYP 2B6 and 3A4, as well as a potential inhibitor of CYP 2C8, 2D6, and 3A4, though neither time dependent nor metabolism dependent inhibition has been observed. Medications that are substrates for CYP 2B6, 2C8, 2D6 or 3A4 and are either sensitive substrates or have a narrow therapeutic index should be avoided where possible. See Table 72 for those medications that are either prohibited or permitted with caution during treatment with sitravatinib. Any patients taking a drug listed as prohibited in Table 72 prior to registration should discontinue them within 5 half-lives prior to commencing trial treatment.

In vitro experiments in microsomes and recombinant human P450 enzymes suggest that sitravatinib is metabolized by several cytochromes including CYP 3A4, 2B6, 2D6, and with a low risk of any one CYP demonstrating a disproportionate contribution to its metabolism. Caution should therefore be used when administering sitravatinib to patients taking medications that are strong inhibitors or inducers of the cytochrome P450 system.

Table 72: Prohibited & permitted (with caution) sensitive substrates and substrates with a narrow therapeutic index for the indicated CYP enzymes

Enzyme	Prohibited	Permitted (with caution)
CYP2B6	Efavirenz	Bupropion
CYP2C8	Paclitaxel	Repaglinide

Enzyme	Prohibited	Permitted (with caution)
CYP2D6		Atomoxetine, desipramine, dextromethorphan, metoprolol, nebivolol, perphenazine, tolterodine, venlafaxine
CYP3A	Darunavir, dasatinib, everolimusindinavir, lopinavir, maraviroc, saquinavir, tipranavir	Alfentanil, aprepitant, budesonide, buspirone, conivaptan, cyclosporine, darifenacin, dihydroergotamine, eletriptan, eplerenone, ergotamine, felodipine, fentanyl, fluticasone, lovastatin, lurasidone, midazolam, nisoldipine, quetiapine, quinidine sildenafil, simvastatin, tacrolimus, triazolam, vardenafil

29.2.3.3 P-gp and BCRP Transporters

Sitravatinib is an inhibitor of BCRP and P-gp transporters based on *in vitro* studies. Concomitant medications that are sensitive substrates and substrates with a narrow therapeutic index for P-gp and BCRP transporters should be avoided where possible. See Table 73 for those medications that are either prohibited or permitted with caution during treatment with sitravatinib. Any patients taking a drug listed as prohibited in Table 73 prior to registration should discontinue them within 5 half-lives prior to commencing trial treatment.

Table 73: Prohibited and permitted (with caution) sensitive substrates and substrates with a narrow therapeutic index for P-gp and BCRP transporters

Enzyme	Prohibited	Permitted (with caution)
P-gp	Everolimus, imatinib, lapatinib, maraviroc, nilotinib, topotecan.	Aliskiren, ambrisentan, colchicine, dabigatran etexilate, digoxin, fexofenadine, posaconazole, ranolazine, saxagliptin, sirolimus, sitagliptin, talinolol, tolvaptan
BCRP	Mitoxantrone, imatinib, irinotecan, lapatinib, topotecan	Methotrexate, rosuvastatin, sulfasalazine

29.2.3.4 Medications for Gastric pH

The solubility of sitravatinib is pH dependent, therefore medications associated with sustained increase in gastric pH may result in decreased exposure to sitravatinib. Where possible, patients still requiring gastric pH medications should switch from use of proton pump inhibitors (PPI) or H2 antagonists to use of antacids prior to trial registration. Antacids should be avoided 4 hours before and 2 hours after administration of sitravatinib treatment when possible.

Where it is not possible to maintain the patient using antacids, H2 antagonists or PPI may be used. H2 antagonists are preferable over PPI as an alternative medication. H2 antagonists or PPI should be administered 12 hours following dosing with sitravatinib and 12 hours prior to the next dose where possible to minimise the effects on pH dependent solubility.

29.2.3.5 Concomitant Surgery

The impact of treatment with sitravatinib on wound healing has not yet been characterised. Patients requiring major surgery during the trial should discontinue trial treatment 2 weeks

before elective surgery and remain off-treatment during the surgery and for 2 weeks following the surgery when possible.

29.2.3.6 Concomitant Radiotherapy

For patients with bone involvement, any foreseeable need for palliative radiotherapy should be addressed before trial entry, if possible and clinically appropriate (e.g., bone lesions at risk for spontaneous micro-fractures or painful lesions). However, these treatments may be used in cases where it is medically necessary; in these cases, the Investigator should discuss with the National Lung Matrix Trial Office if sitravatinib should be held prior to, during, and after radiation therapy. As a general rule it is advisable to withhold sitravatinib treatment at least 1 day prior and 1 day post radiotherapy, however consideration should be given to the anatomic site, radiation dose and duration, and potent VEGFR inhibition of sitravatinib (e.g. 1-3 fractions to an extremity may only require the minimum, but radiation that encompasses the abdomen may warrant a longer treatment break).

29.2.3.7 Other

Therapeutic colony-stimulating factors should be used in accordance with ASCO guidelines. Packed red blood cell and platelet transfusions may be administered as clinically indicated.

29.2.3.8 Contraception

It is not known whether sitravatinib presents a risk to the embryo or the foetus. Sitravatinib is contraindicated in women who are pregnant or lactating. Women of childbearing potential and men receiving sitravatinib who are sexually active must employ an effective method of contraception throughout their period of treatment and for 6 months after their last treatment with sitravatinib. Acceptable methods of contraception are described in Section 6.3.

29.3 Trial Treatment

29.3.1 Investigational Medicinal Product

Sitravatinib (MGCD516) will be provided free of charge by Mirati Therapeutics as 10 mg and 40 mg unit dose strength capsules. Sitravatinib is not approved for marketing in any region of the world. The dose of sitravatinib will be 120 mg once daily.

Sitravatinib capsules should be taken once daily in a fasted state, at least 2 hours after the previous meal and 1 hour before the next meal. Capsules should be taken during the morning, for instance, at least 1 hour before breakfast or lunch. Sitravatinib capsules should be taken with at least 230 mL (1 cup) of water. Both the Arm H Patient Information Sheet and Arm H Patient Diary contain more specific instructions for patients to follow regarding how to take their medication.

If the morning dose is missed, the dose may be taken up to 12 hours after the usual time as long as the sitravatinib capsules are taken in a fasted state, i.e., at least 2 hours after the previous meal and 1 hour before the next meal. If more than 12 hours have passed from usual administration time, the dose for that day should be missed.

If vomiting occurs after dosing, the sitravatinib dose should not be replaced.
Please refer to the Pharmacy Manual for further details.

29.3.2 Schedule of Assessments

Table 74: Sitravatinib (MGCD516) - Schedule of Assessments (Screening/During Treatment)

	**Screening**	**Treatment 120 mg once daily (21 day cycles)**					**Discontinuation** (+ 7 days)**	**28 day follow up visit**[u] (+ 7 days)**	**Post-28 day follow up** (± 7 days)***
	Within 28 days of treatment (unless otherwise stated)	Cycle 1		Cycle 2		Cycle 3 onwards			
		Day 1	Day 10 (± 2 days)*	Day 1	Day 10 (± 2 days)*	Day 1 (± 2 days)*			
Informed consent[a]	X								
Demography & baseline characteristics[b]	X								
Medical history[c]	X								
Inclusion / exclusion criteria[d]	X								
Physical examination[e]	X	X		X		X	X		
ECOG performance status	X (within 14 days of treatment)	X		X		X	X		
Vital signs (inc. weight)[f]	X	X	X	X	X	X	X		
ECG[g]	X (within 7 days of treatment)	X	X	X	X		X		
Multi-gated acquistion scan (MUGA) / Echocardiogram (ECHO)[h]	X					X (cycle 3 & 5 only) (± 7 days)	X (± 7 days)		

	Screening	Treatment 120 mg once daily (21 day cycles)					Discontinuation (+ 7 days)**	28 day follow up visit[u] (+ 7 days)**	Post-28 day follow up (± 7 days)***
	Within 28 days of treatment (unless otherwise stated)	Cycle 1		Cycle 2		Cycle 3 onwards			
		Day 1	Day 10 (+ 2 days)*	Day 1	Day 10 (+ 2 days)*	Day 1 (+ 2 days)*			
Haematology, Clinical chemistry[i]	X (within 7 days of treatment)	X	X (- 2 days)	X (-2 days)	X (- 2 days)	X (- 2 days)	X (+ 7 days)		
Coagulation[i]	X								
Thyroid function test[j]	X	X				X (every other cycle beginning cycle 3) (- 2 days)	X		
Pregnancy test[k]	X	X					X		
Urinalysis[l]	X	X		X (-2 days)		X (-2 days)	X		
Tumour assessments[m]	X	Every 6 weeks during year 1 (+7 days) [except 1st scan +7 days only]							X ◊
Adverse events & Concomitant Medications	X	X	X	X	X	X	X	X	
Dispense sitravatinib		X		X		X (- 2 days)			
Administer sitravatinib[n]									
Smoking status[o]		X				X (every 9 weeks beginning cycle 4)	X		

	Screening	Treatment 120 mg once daily (21 day cycles)					Discontinuation (+ 7 days)**	28 day follow up visit[u] (+ 7 days)**	Post-28 day follow up (± 7 days)***
	Within 28 days of treatment (unless otherwise stated)	Cycle 1		Cycle 2		Cycle 3 onwards			
		Day 1	Day 10 (± 2 days)*	Day 1	Day 10 (± 2 days)*	Day 1 (± 2 days)*			
Germline DNA sample[p]		X (- 2 days)							
ctDNA samples[q]		X (- 2 days)				X (every 9 weeks beginning cycle 4) (-2 days)	X		X $
Pharmacokinetic (PK) sample[r]		X	X	X					
Optional research biopsy[s]		X (post-reg, pre-tx)					X		
Survival status[t]									X

* Visit may occur ± 2 days of the planned visit date. Individual assessments may occur independently of the visit date where indicated in the table above.

** Visit may occur + 7 days of the planned visit date.

*** Visit may occur ± 7 days of the planned visit date.

a Prior to the start of any trial specific procedures, each patient must provide signed informed consent.

b At screening, demography must be captured for all patients. Demographic data and other characteristics will include: date of birth, gender, race/ethnicity.

c At screening, a standard medical and surgical history will be obtained, including prior cancer treatment.

d Patients must not be registered unless all eligibility criteria have been fully met.

e Physical examination should be performed at screening, prior to dosing on day 1 of every cycle, and at discontinuation. Additional examinations during trial treatment will be symptom directed as required.

Includes general appearance, respiratory, cardiovascular, skin, head and neck (including ears, eyes, nose and throat), lymph nodes, thyroid, abdomen, musculo-skeletal (including spine and extremities) and neurological systems.

f Vital signs (weight, height (at screening), BP and pulse) to be taken at screening, day 1 (pre-dose) & day 10 of cycles 1 & 2, then on day 1 of subsequent cycles, and at discontinuation.

Single measurements of supine blood pressure (BP) and pulse will be recorded on each occasion after 10 minutes rest. Blood pressure should be checked immediately prior to PK sampling.

g ECG to be taken at screening, day 1 and day 10 of cycles 1 & 2, and at discontinuation. Cycle 1 day 1 and day 10 ECGs should be performed prior to dosing and immediately prior to PK sample collection. Additional ECGs may be performed if clinically indicated.

Single twelve-lead ECG will be obtained and should be performed after the patient has been resting semi-supine for at least 10 minutes prior to the recording.

All ECGs should be recorded with the patient in the same physical position. A standardised ECG machine should be used and the patient should be examined using the same machine throughout the trial if possible. After paper ECGs have been recorded, the Investigator or designated physician will review each of the ECGs and may refer to a local cardiologist if appropriate. A paper copy should be filed in the patient's medical records. If an abnormal ECG finding at screening or cycle 1 day 1 is considered to be clinically significant by the Investigator, it should be reported as a concurrent condition. For all ECGs details of rhythm, ECG intervals (R-R, PR, QT and QRS) and an overall evaluation will be recorded.

h MUGA/ECHO should be performed at screening[#], cycle 3 day 1, cycle 5 day 1 and at discontinuation. After the screening scan, assessments may take place up to 7 days earlier or 7 days later than the scheduled visit. Additional assessments may be performed if clinically indicated.

[#]A scan carried out within 3 months prior to starting trial treatment can be used for eligibility at screening, however, for patients who experience a significant cardiac event or who have had cardiotoxic medication within this interval, the MUGA/ECHO scan should be repeated prior to starting trial treatment.

The modality of the cardiac function assessments must be consistent per patient and the same machine operator is to be used where possible.

i Coagulation samples to be collected at screening. Further samples may be collected as clinically indicated.

Haematology & clinical chemistry samples to be collected at the following time points:

- Screening (within 7 days of treatment)
- Cycle 1 day 1 (pre-dose) and day 10 (day 10 samples may be taken up to 2 days earlier)
- Cycle 2 day 1 and day 10 (samples may be taken up to 2 days earlier)
- Day 1 of subsequent cycles (cycle 3 onwards) (samples may be taken up to 2 days earlier)

- Discontinuation (samples may be taken up to 7 days later at discontinuation).

Clinical chemistry: Albumin , ALT, AST, alkaline phosphatase, bicarbonate, chloride, creatinine, glucose, LDH, magnesium, phosphate, potassium, sodium, total bilirubin, total calcium, total protein, urea nitrogen, uric acid.

Haematology: FBC.

Coagulation: International Normalised Ratio (INR), partial thromboplastin time (PTT).

j Thyroid functions tests (TSH) to be performed at screening, pre-dose cycle 1 day 1, then day 1 of every other cycle (cycles 3, 5, 7 etc), and at discontinuation. After cycle 1 day 1, tests during treatment may be performed up to 2 days earlier than the visit date, and up to 7 days later at discontinuation.

TSH will be performed. If TSH is abnormal, free T4 will be collected to assess axis.

k A serum or urine pregnancy test is to be performed at screening, pre dose on cycle 1 day 1 and at discontinuation. At discontinuation, this may be performed up to 7 days later.

Female patients of child-bearing potential only. In the event of suspected pregnancy during the trial, the test should be repeated and, if positive, the patient discontinued from trial treatment immediately.

l Urinalysis to be performed at screening, pre-dose cycle 1 day 1, day 1 of subsequent cycles, and at discontinuation. After cycle 1 day 1, urinalysis may be performed up to 2 days earlier than the visit date and up to 7 days later at discontinuation.

Protein, glucose and blood to be assessed. If urinalysis abnormal, perform microscopy – red blood cells, white blood cells, bacteria, casts, crystals. If urine protein 2+ is observed during treatment, evaluation should include urinalysis with 24-hour urine collection.

m CT or MRI scan of head, chest and abdomen to be performed at screening. CT or MRI scans of chest and abdomen to be performed until discontinuation. Tumour assessments should be performed relative to the date of start of treatment (cycle 1 day 1) every 6 weeks for the first year, then every 12 weeks. Scans should be performed ± 7 days (except 1st scan + 7 days only). If brain metastases are identified at screening or if clinically indicated, head scanning will also be performed at the same time points. The same imaging modality must be used consistently throughout the course of the trial for each patient.

◊ Tumour assessments will be performed in follow up for patients who discontinue treatment for reasons other than Progressive Disease (e.g. toxicity). These scans should continue to be performed on a 6-weekly basis for the first year relative to the start date of treatment, then every 12 weeks until disease progression or the patient starts a new anti-cancer therapy (unless the patient withdraws consent to do so).

Scans should be of the chest and abdomen, and only include the head where brain metastases are identified at screening, or if clinically indicated. All scans to be reported using RECIST 1.1.

n Cycle 1 day 1: Treatment must commence within 7 days of trial registration.

o Smoking status data will be collected through questions and carbon monoxide (CO) monitoring pre-dose cycle 1 day 1, then every 9 weeks (day 1 of every third cycle beginning cycle 4), and at discontinuation.

p A whole blood germline DNA sample is to be collected pre-dose on cycle 1 day 1. The sample can be taken up to 2 days earlier than the actual visit date (commencement of cycle). If the sample is not collected at this time point, it should be collected at the next visit. Refer to the Laboratory Manual for sample processing guidelines.

q ctDNA samples to be collected at pre-dose cycle 1 day 1, then every 9 weeks (day 1 of every third cycle beginning cycle 4), and at discontinuation. Samples can be taken up to 2 days earlier than the actual visit date (commencement of cycle) where indicated. Refer to Laboratory Manual for sample processing guidelines.

$ ctDNA samples will be collected in follow up for patients who discontinue treatment for reasons other than Progressive Disease (e.g. toxicity). These samples should be performed at the same visit as follow up CT scans until disease progression or the patient starts a new anti-cancer therapy (unless the patient withdraws consent to do so). Samples should be collected on a 6-weekly basis for the first year relative to the start date of treatment, then every 12 weeks.

r PK samples should be collected following assessment of vital signs at the following time points:

- Cycle 1 day 1: ≤ 30 minutes pre-dose & 4 hours post-dose
- Cycle 1 day 10: ≤ 30 minutes pre-dose
- Cycle 2 day 1: ≤ 30 minutes pre-dose
- An additional ad hoc sample should be collected within 7 days of a Serious Adverse Reaction or a dose modification

Refer to Laboratory Manual for sample processing guidelines.

s An optional fresh metastatic/recurrent tumour biopsy sample should be collected (if patient consents) post–registration (pre-treatment) and at the end of treatment visit for patients who discontinue treatment for reasons other than disease progression (origin from either the primary tumour of site of metastasis). An optional pre-treatment biopsy should not be performed in cases where the patient has already had a mandatory biopsy for molecular testing (Note - a mandatory repeat SMP2 biopsy will be performed if the patient has had targeted therapy e.g. ALK inhibitor). The discontinuation biopsy must be performed prior to commencing further anti-cancer therapy. A post-treatment biopsy will only be requested from patients with an objective response or stabilisation of disease (PR or CR), or 6 months on treatment with evidence of stabilisation (SD) for patients who have previously progressed. The tumour tissue will be used to determine possible mechanisms of resistance to study treatment. Refer to the Laboratory Manual for sample processing instructions.

t Survival status will be collected every 12 weeks (± 7 days) post-permanent discontinuation of trial treatment until death.

u 28 day follow up visit should be carried out 28 days (+ 7 days) post-permanent discontinuation of trial treatment.

29.3.3 Toxicity Profile

29.3.3.1 Safety issues

The genotoxicity studies on sitravatinib indicate a lack of potential to induce point mutations, chromosomal aberrations, or to interact with or damage DNA, thus the risk for potential genotoxic effects is low.

29.3.3.2 Expected Adverse Events

Based on review of the adverse events reported with sitravatinib in context of the mechanism of action and nonclinical data, frequency, investigator assessment of causality, and medical literature, the adverse events listed in Table 75: Single Agent Sitravatinib Adverse Drug Reactions are considered to be adverse drug reactions (ADRs, i.e. adverse events with at least a reasonable possibility of a causal relationship to investigational product) for single-agent sitravatinib. (Refer to 29.3.4 for toxicity management guidelines).

Table 75: Single Agent Sitravatinib Adverse Drug Reactions

MedDRA System Organ Class	MedDRA Preferred Term	Frequency of Treatment Emergent AE (All grades) n=86	Frequency of Treatment Emergent AE (Grade 3/4) n=86
**General Disorders & Administration Site Conditions**	Fatigue	46 (53.5%)	8 (9.3%)
	Mucosal Inflammation[1]	6 (7.0%)	2 (2.3%)
	Asthenia	10 (11.6%)	0 (0.0%)
**Gastrointestinal Disorders**	Diarrhoea	41 (47.7.0%)	10 (11.6%)
	Nausea	32 (37.2.0%)	2 (2.3%)
	Vomiting	29 (33.7%)	3 (3.5%)
	Stomatitis[1]	8 (9.3%)	0 (0.0%)
**Vascular Disorders**	Hypertension	36 (41.9%)	20 (23.2%)
**Metabolism & Nutritional Disorders**	Decreased Appetite	31 (36.0%)	1 (1.2%)
**Endocrine Disorders**	Thyroid Disorder[2]	20 (23.3%)	0 (0.0%)
**Skin and Subcutaneous Tissue Disorders**	Palmar-Plantar Erythrodysaesthesia Syndrome	12 (14.0%)	2 (2.3%)
**Investigations**	Ejection Fraction Decreased[3]	6 (7.0%)	4 (4.7%)

(Study 516-001 – MGCD516 (Sitravatinib) Investigator Brochure v4.0 11th September 2017 data cut)

1 Mucositis was collectively assessed with MedDRA PTs Mucosal Inflammation and Stomatitis.

2 Thyroid Disorder was collectively assessed with MedDRA PTs Hypothyroidism (n=16), Hyperthyroidism (n=1), and Blood Thyroid Stimulating Hormone Increased (n=3).

3 Ejection Fraction Decreased < 50% includes events reported as adverse events (n=4) and events identified during review of Study 516-001 MUGA/Echocardiogram results (n=2).

29.3.3.2.1 Fatigue/Asthenia

Given the high frequency of treatment emergent fatigue (53.5%) in Study 516-001 versus the background rate (~30%), and because there is some corroboration with preclinical data and other RTKIs, fatigue and asthenia have been assessed as adverse drug reactions (ADR) of sitravatinib.

29.3.3.2.2 Diarrhoea

Diarrhoea has been reported with sitravatinib treatment, though the mechanism remains unclear, as with other small molecule RTKIs. Given the high frequency of treatment emergent diarrhoea (47.7%) in Study 516-001 versus the background rate (7-10%) and because of corroboration with preclinical data and other RTKIs, diarrhoea has been assessed as an ADR of sitravatinib.

29.3.3.2.3 Nausea & Vomiting

Although the frequencies of treatment emergent nausea (37.2%) and vomiting (33.7%) approximate the combined background rate (~ 70%), the frequency is still high and there is corroboration with preclinical data and other RTKIs. Nausea and vomiting have been assessed as ADRs of sitravatinib.

29.3.3.2.4 Hypertension

Hypertension, including Grade 3 events, has been reported with sitravatinib. Although the observed frequency of treatment emergent hypertension (41.9%) is similar to the background rate (~ 40%), the frequency is still high, several subjects who were normotensive at baseline experienced hypertension shortly after the first administration of sitravatinib, and there is corroboration with pre-clinical data and other RTKIs. Hypertension has been assessed as an ADR of sitravatinib.

29.3.3.2.5 Decreased Appetite

Given the high frequency of treatment emergent decreased appetite (36.0%) in Study 516-001 versus the background range (15% to 25%), and corroboration with pre-clinical data and other RTKIs, decreased appetite has been assessed as an ADR of sitravatinib.

29.3.3.2.6 Mucosal Inflammation/Stomatitis

Given the high combined frequency (16.3%), and corroboration with pre-clinical and other RTKIs, mucosal inflammation/stomatitis has been assessed as an ADR of sitravatinib.

29.3.3.2.7 Palmar-Plantar Erythrodysesthesia (PPE) Syndrome

The frequency of treatment emergent palmar-plantar erythrodysaesthesia (14.0%) is within the range observed with other VEGFR inhibitors (9% to 62%); thus, there is corroboration with other RTKIs. Palmar-plantar erythrodysaesthesia has been assessed as an ADR of sitravatinib. Palmar-plantar erythrodyesthesia (PPE) was reported as a dose-limiting toxicity in the Phase 1 study of sitravatinib. Signs and symptoms of PPE include redness, swelling, pain, and less commonly blisters on the palms of the hands and/or the soles of the feet.

29.3.3.2.8 Thrombotic Events

The risk of thrombotic events with sitravatinib is unclear; however, arterial and venous thrombotic events have been described in patients treated with VEGFR inhibitors. In the first-in-human study of sitravatinib, four cases of pulmonary embolism have been reported. The occurrence of the adverse event of pulmonary embolism with sitravatinib is being monitored for further characterisation.

29.3.3.2.9 Decrease in LVEF

Decreased left ventricular ejection fraction (LVEF) has been described in patients treated with various small molecule VEGFR inhibitors. In the first-in-human study of sitravatinib, assessments of LVEF were included in the Schedule of Assessments. Decreased LVEF to < 50% (n=5) and/or a treatment-emergent adverse event of cardiac failure (n=1) has been observed in 6 (7.0%) of subjects administered sitravatinib. Among these 6 patients, cardiac failure (n=1), left ventricular dysfunction (n=2), or ejection fraction decreased (n=3) were reported in 4 patients on study. All 6 patients were diagnosed with confounding factors prior to study enrolment (cardiac tamponade, haemochromatosis, left heart catheterisation, cardiomyopathy and heart disease, hyperlipidaemia, coronary artery disease, hypertension and heart murmur). The events of cardiac failure resolved following interruption of sitravatinib. The occurrence of these adverse events is being monitored for further characterisation. Ejection fraction decrease has been assessed as an ADR for sitravatinib.

29.3.3.2.10 Haemorrhagic Events

The risk of haemorrhagic events with sitravatinib is unknown; however, such events have been reported with inhibitors of VEGFR. Patients with active haemoptysis or gastrointestinal bleeding should not take sitravatinib, and interruption of treatment is recommended for patients developing clinically significant bleeding. Additionally, adrenal haemorrhage and necrosis have been described in animal studies of small molecule RTKIs including sitravatinib. Repeat adrenal imaging as part of the CT or MRI of the chest or abdomen has been included in the first-in-human clinical trial without any reporting of such cases to date.

29.3.3.2.11 Thyroid Dysfunction

Thyroid dysfunction, which includes hypothyroidism, blood thyroid stimulating hormone increased, and hyperthyroidism was observed in 16 (18.6%), 3 (3.5%), and 1 (1.2%) of Study 516-001 subjects, respectively. Given the high combined frequency (23.3%), and corroboration with pre-clinical observations and other receptor tyrosine kinase inhibitors, thyroid dysfunction has been assessed as an adverse drug reaction of sitravatinib.

29.3.3.2.12 Proteinuria

Although the risk with sitravatinib is unclear, proteinuria has been described with other inhibitors of the VEGFR pathway.

29.3.3.2.13 Neutropenic Fever/Infection

Patients with neutropenic fever of infection should be treated promptly as per local practice.

29.3.4 Dose Modifications & Toxicity Management

29.3.4.1 Treatment delays and discontinuation

In the event of adverse events attributed to sitravatinib and deemed intolerable by the Investigator, treatment should be either temporarily or permanently discontinued. For patients who temporarily discontinue treatment, treatment may resume following resolution of treatment-related adverse events to Grade 1 or baseline with the administration of sitravatinib at a reduced dose level as outlined in 29.3.4.3.

Patients temporarily discontinuing study treatment should be considered for permanent discontinuation of trial treatment if toxicity does not recover within 4 weeks or if more than 3 dose reductions are required (see 29.3.4.2 and 29.3.4.3 for recommencement criteria and dose reduction guidance). This should be discussed with the National Lung Matrix Trial Office.

If the administration of sitravatinib is interrupted for reasons other than toxicity, then treatment with the trial drug may be resumed at the same dose. The same applies if treatment is interrupted for an adverse event thought not to be related to protocol therapy by the Investigator.

29.3.4.2 Criteria for recommencement of treatment

The following parameters should be met prior to recommencement of treatment: 1) at the start of every new cycle and; 2) following a treatment interruption for treatment related toxicity:

- Non-haematological treatment-related adverse event reduction to Grade 1 or baseline.
- Neutropenia treatment-related adverse event reduction to Grade 2 or baseline.
- Thrombocytopenia treatment-related adverse event reduction to Grade 1 or baseline.

See Section 29.3.4.3 for dose level reduction guidance.

29.3.4.3 Dose modifications

Table 76: Sitravatinib dose modifications

**Dose level**	**Sitravatinib dose**
Starting dose sitravatinib	120 mg OD
Dose level -1	100 mg OD
Dose level -2	80 mg OD

Dose reductions should be based on the worst preceding treatment-related toxicity.

29.3.4.3.1 Non-Haematological Toxicities

In general, Grade 1 and 2 non-haematological toxicities should be managed at the discretion of the local Investigator and patient. Initially, the dose for patients developing Grade 2 potential ADRs should be maintained in order to assess whether continued treatment is feasible without increasing the severity of the adverse event. Similar adverse events occurring in subsequent patients may be managed in the context of the experience gained in earlier patients or following discussion with other NLMT Investigators.

Treatment-related non-haematological toxicities $\geq$ Grade 3 should be managed with treatment interruption until criteria for recommencement is met (see Section 29.3.4.2). If the toxicity is adequately managed by routine supportive care, treatment may be resumed at the same dose (see Table 77); if not, treatment may be resumed at a reduced dose as described in Table 77.

Recurrence of toxicity may be managed similarly.

Table 77: Dose Modifications for Non-Haematological Drug Related Toxicities

Toxicity	Treatment Delay	Dose Modification
< Grade 3	May be implemented based on Investigator and patient discretion	
Grade 3/4 manageable with routine supportive care	Hold until ≤ Grade 1 or return to baseline	Not required
Grade 3/4 not manageable with routine supportive care	Hold until ≤ Grade 1 or return to baseline	Reduce by one or more levels below dose level inducing the toxicity

29.3.4.3.2 Haematological Toxicity

Initially in the event of Grade 1 or 2 neutropenia or Grade 1 thrombocytopenia, the sitravatinib dose should be maintained in order to assess whether continued treatment is feasible without increasing the severity of the adverse event. Similar adverse events occurring in subsequent patients may be managed in the context of the experience gained in earlier patients or following discussion with other NLMT Investigators, unless the experience indicates that dose interruption and/or reduction are warranted.

Haematological toxicities ≥ Grade 3 should be managed with treatment interruption until resolution as described in Table 4. Recurrence of toxicity may be managed similarly.

Table 78: Dose Modifications for Haematological Drug Related Toxicities

Neutropenia	Treatment Delay / Dose Modification
Grade 1 (ANC: 1.5 x 10^9/L to <LLN)	Maintain dose level
Grade 2 (ANC: 1.0 x 10^9/L to <1.5 x 10^9/L)	
Grade 3 (ANC: 0.5 x 10^9/L to <1.0 x 10^9/L)	Omit dose until resolved to ≤ Grade 2 then:<br>• If resolved in ≤ 7 days maintain dose level<br>• If resolved in > 7 days then reduce by 1 or more levels below dose level inducing the toxicity
Grade 4 (ANC < 0.5 x 10^9/L)	Omit dose until resolved to ≤ Grade 2 then reduce by 1 or more levels below dose level inducing toxicity
**Thrombocytopenia**	**Treatment Delay / Dose Modification**
Grade 1 (PLT: 75 x 10^9/L to <LLN)	Maintain dose level
Grade 2 (PLT: 50 x 10^9/L to <75 x 10^9/L)	Omit dose until resolved to ≤ Grade 1 then:<br>• If resolved in ≤ 7 days maintain dose level<br>• If resolved in > 7 days then reduce by 1 or more levels below dose level inducing the toxicity
Grade 3 (PLT: 25 x 10^9/L to <50 x 10^9/L)	Omit dose until resolved to ≤ Grade 1 then reduce by 1 or more levels below dose level inducing toxicity
Grade 4: (PLT: <25 x 10^9/L)	

29.3.4.4 Evaluation, management & treatment of toxicities

29.3.4.4.1 Stomatitis/Mucosal inflammation

Stomatitis has been described with use of VEGFR inhibitors, but the mechanism remains unknown. Treatment of events of Grade 1-2 severity is generally supportive and includes:

- Avoidance of hot beverages and foods that are salty, spicy, or acidic.
- Use of pain relievers like paracetamol (acetaminophen) or ibuprofen.
- Gargling with cool water or application of ice pops to affected areas.
- Application of a topical anaesthetic such as lidocaine or xylocaine to the affected areas

Drug interruption and/or dose modification is not generally required for Grade 1-2 events, but should generally be made for Grade 3-4 events.

29.3.4.4.2 Nausea/Vomiting

Nausea/vomiting should generally be managed as per institutional guidelines. Modifications to concomitant medications may need to be considered as indicated below:

- Avoided where possible: ondansetron, droperidol, and chlorpromazine.
- Used with caution: diphenhydramine, aprepitant.

Drug interruption and/or dose modification is not generally required for Grade 1-2 events, but should generally be made for Grade 3-4 events that cannot be managed with anti-emetics.

29.3.4.4.3 Hypertension

Patients taking sitravatinib should undergo regular blood pressure monitoring and receive treatment with standard anti-hypertensive therapy if necessary. Dihydropyridine calcium channel blockers such as nifedipine, amlodipine, and nicardipine may be considered if anti-hypertensive therapy is required. In cases of clinically significant increases in blood pressure, temporary suspension of sitravatinib dosing is recommended until blood pressure is controlled. Treatment with sitravatinib may resume at the same or a lower dose at the discretion of the Investigator. If significant hypertension recurs, options include change in medical management of the patient, reduction of sitravatinib dose, or discontinuation of trial treatment, at the discretion of the Investigator.

29.3.4.4.4 Palmar-Plantar Erythrodysesthesia (PPE)

Patients who develop PPE should be counselled on measures to mitigate the effects of PPE. Such measures include avoidance of exposure of hands and feet to hot water when washing dishes or bathing, or to other sources of heat, avoidance of activities that cause unnecessary force or friction (rubbing) on the hands or feet, avoiding contact with harsh chemicals such as cleaning products, use of tools or household items that result in pressure on the hands, such as garden tools, knives, and screwdrivers, and wearing of loose fitting, well-ventilated shoes and clothes. Treatment may include use of topical moisturising agents, topical anaesthetics, or topical anti-inflammatory medications such as corticosteroid creams. In more severe cases, dose interruption and reduction may be warranted.

29.3.4.4.5 Diarrhoea

Patients should be counselled that diarrhoea is a possible side effect and advised to take loperamide or a similar medication as needed if diarrhoea develops. Any patients developing dehydration or clinically significant electrolyte abnormalities should interrupt treatment, but treatment may be restarted once diarrhoea is controlled.

29.3.4.4.6 Thrombotic Events

Precautions should be taken in patients with recent, clinically significant thrombotic events, and treatment should be discontinued in patients who develop clinically significant thromboembolic complications such as acute myocardial infarction or severe pulmonary embolism.

29.3.4.4.7 Decrease in LVEF

In the presence of a new diagnosis of congestive heart failure (CHF) on-trial, interruption of sitravatinib dosing is recommended.

29.3.4.4.8 Haemorrhagic Events

Patients with active haemoptysis or gastrointestinal bleeding should not take sitravatinib, and interruption of treatment is recommended for patients developing clinically significant bleeding.

29.3.4.4.9 Thyroid Dysfunction

Patients should also be observed for potential signs and symptoms of thyroid dysfunction while receiving treatment with sitravatinib. Patients diagnosed with hypothyroidism should be treated with thyroid replacement and may continue treatment with sitravatinib at the Investigator’s discretion.

29.3.4.4.10 Proteinuria

Repeat assessments with urinalysis for urine protein should be performed according to the Schedule of Assessments. Patients who develop ≥ 2+ proteinuria should undergo 24-hour urine collection for assessment of urine protein; treatment with sitravatinib should be discontinued in the presence of ≥ 2 grams of proteinuria/24 hours and may restart when protein levels decrease to < 2 grams/24 hours. Patients who develop nephrotic syndrome should be withdrawn from treatment with sitravatinib.

29.4 Reference List

- An SJ, Chen ZH, Su J, et al. Identification of Enriched Driver Gene Alterations in Subgroups of Non-Small Cell Lung Cancer Patients Based on Histology and Smoking Status. *Plos One,* 7:1-8, 2012.
- Beroukhim R, Mermel CH, Porter D, et al. The landscape of somatic copy-number alteration across human cancers. *Nature* 463: 899-905, 2009.
- Ding L, Getz G, Wheeler DA, et Al. Somatic mutations affect key pathways in lung adenocarcinoma. *Nature* 455:1069-1075, 2008.
- Drilon A. et al. Response to Cabozantinib in patients with RET fusion-positive lung adenocarcinomas. *Cancer Discov.* 2013; 3(6): 630-5
- Falchook G.S. et al. Effect of the RET inhibitor Vandetanib in a patient with RET fusion-positive metastatic non-small-cell lung cancer. *J. Clin. Oncol.* 2016; 34(15): e141-4
- Hammerman PS, Sos ML, Ramos AH, et al. Mutations in the DDR2 kinase gene identify a novel therapeutic target in squamous cell lung cancer. *Cancer Discov*., 1:78-89, 2011.
- Harad T, Yatabe Y, Takeshita M, et. Al.. Role and Relevance of TrkB Mutations and Expression in Non-Small Cell Lung Cancer. *Clin Cancer Res,* 17:2638-2645, 2011.
- Horiike A. et al. Sorafenib treatment for patients with RET fusion-positive non-small cell lung cancer. *Lung Cancer.* 2016; 93: 43-6
- Kohno T. et al. KIF5B-RET fusions in lung adenocarcinoma. *Nat. Med.* 2012; 18(3): 375-7
- Kong-Bletran M, Seskagiri S, Zha J et al. Somatic Mutations Lead to an Oncolgenic Deletion of Met in Lung Cancer. *Cancer Res* ;66:283-289, 2006.
- Lin C. et al. The RET fusion gene and its correlation with demographic and clinicopathological features of non-small cell lung cancer: a meta-analysis. *Cancer Biol. Ther*. 2015; 16(7):1019-28
- Lipson D, Capalletti M, Yelensky R, et. al., Identification of New ALK and RET Gene Fusions from Colorectal and Lung Cancer Biopsies. *Nat Med.* 18:382-384, 2012.
- Marchetti A, Felicioni L, Pelosi G, et al. Frequent Mutations in the Neurotrophic Tyrosine Receptor Kinase Gene Family in Large Cell Neuroendocrine Carcinoma of the Lung. *Hum Mutat*., 29:609-616, 2008.
- Mirati Therapeutics. Proof of concept anti-tumor efficacy in NSCLC models exhibiting RTK target alterations (personal communication).
- Mukhopadhyay S. et al. RET-rearranged lung adenocarcinomas with lymphangitic spread, psammoma bodies, and clinical responses to Cabozantinib. *J. Thorac. Oncol*. 2014; 9(11): 1714-9
- Onozato R, Kosaka T, Kuwano H, et Al. Activation of MET by Gene Amplification or by Splice Mutations Deleting the Justamembrane Domain in Primary Resetced Lung Cancers. *J Thorac Oncol* 4 :5-11, 2009.
- Tsuta K. et al. RET-rearranged non-small-cell lung carcinoma: a clinicopathological and molecular analysis. *Br. J. Cancer.* 2014; 110(6): 1571-8
- Vaishnavi A, Capelletti M, Le AT, et al. Oncogenic and drug-sensitive NTRK1 rearrangements in lung cancer. *Nat Med*. 19:1469-72, 2013.
- Wu H. et al. Rapid response to Sunitinib in a patient with lung adenocarcinoma harboring KIF5B-RET fusion gene. *J. Thorac. Oncol*. 2015; 10(9): e95-6

30 ARM J & COHORT NAJ: AZD6738 (ATR INHIBITOR) & DURVALUMAB (ANTI-PDL1)

Lead Investigator: Professor Gary Middleton

30.1 Background & Rationale

Arm	Investigational Medicinal Product	Cohort Number	NSCLC Histology	Molecular Cohort
J	AZD6738 & Durvalumab	J1	NSCLC	KRAS mutation<br>STK11/LKB1 successful test result
NA		NAJ	NSCLC	No actionable genetic change for other trial arms

Investigators should be familiar with the current AZD6738 and durvalumab (MEDI4736) Investigator's Brochures (IB).

AZD6738 is an orally dosed selective and potent inhibitor of Ataxia Telangiectasis and Rad3 Related (ATR) kinase with good selectivity against other Pi3 kinase family members.

ATR is a serine/threonine protein kinase and member of the phosphatidylinositol 3-kinase related kinase (PIKK) family. During normal replication, ATR is recruited at stalled replication forks which can progress to double strand breaks if left unrepaired. ATR is also recruited to single strand DNA coated with Replication Protein A (RPA) following single strand DNA damage or the resection of double strand breaks. Recruitment and activation of ATR leads to cell cycle arrest in the S phase while the DNA is repaired and the stalled replication fork resolved, or nuclear fragmentation and entry into programmed cell death (apoptosis).

In the clinic, ATR inhibitors are expected to cause growth inhibition in tumour cells dependent upon ATR for DNA repair e.g. ATM-deficient tumours. In addition to monotherapy activity, ATR inhibitors are also predicted to potentiate the activity of cytotoxic DNA damaging agents and radiotherapy (through inhibition of ATR-dependent DNA repair processes) when used in combination. While significant enhancement of anti-tumour activity may be achieved, data with AZD6738 suggest the potential need to reduce the ATR inhibitor dose and intensity (relative to monotherapy dose) and introduce dosing breaks to allow normal tissue recovery when used in combination with systemic DNA damaging chemotherapy agents, in order to maintain tolerable therapeutic margins. There is also a rationale for combining with anti-PD1/PD-L1 agents, such as durvalumab as ATR inhibition may stimulate the production of tumour neo-antigens and directly affect the tumour immune microenvironment but more importantly may positively modulate the tumour microenvironment through the induction of DNA damage.

In addition, in vivo, combination of AZD6738 ceralasertib with radiation, platinum chemotherapy or olaparib (for example) induces a dose-dependent antitumour response, with regressions in ATM-deficient models and growth inhibition in ATM-proficient models. Anti-tumour activity of AZD6738 ceralasertib may, therefore, also be seen more broadly.

Recent data has highlighted the pivotal importance of the cytosolic DNA sensing cGAS-STING pathway in activating a micro-environmental anti-cancer immune response in cells with

unresolved DNA damage. DNA damage repair (DDR) deficient cancers are associated with significant CD4+ and CD8+ T cell infiltration related to enhanced expression of CXCL10 and CCL5 (1). These cells have elevated levels of cytosolic DNA which activate cGAS-STING signalling which drives IRF3 expression which thus up-regulates chemokine production thus attracting T cells into the micro-environment. STING activation also up-regulates tumoural PD-L1 expression. Similar cell-intrinsic immune activation via cGAS with STAT1 up-regulation is seen after ionising radiation which causes DSB formation (2). cGAS was found to localise to micronuclei which arise during mitosis from lagging chromosomal DNA and chromatin bridges as a result of unresolved genomic instability (2,3). After DNA damage up-regulation of interferon stimulated genes was only seen in micronucleated cells and critically DNA damage per se was not enough to cause immune activation after DNA damage: the production of micronuclei at mitosis was essential. Micronuclei have defective nuclear lamina and thus are prone to rupture and it is this rupture that exposes DNA to the cytosol and results in cGAS localisation to micronuclear chromatin. Thus, unresolved DNA damage transmitted into mitosis causes cGAS-activation and activation of local immunity with T cell infiltration and PD-L1 up-regulation. Micronucleated cells are those that drive this cell-intrinsic immune activation, creating a microenvironment that might be optimally enabled with immune checkpoint blockade. Treatment of cancer cells with the ATR inhibitor AZD6738 results in DNA damage and the formation of micronucleated cells (4). Further work by this group has shown that AZD6738 causes an increased number of intra-tumoural CD3+ cells and NK cells and a significant increase in granzyme B and perforin levels (5). There was also increased intratumoural dendritic cell numbers and macrophages but with no evidence of M2 polarisation. PD-L1 expression was increased on these myeloid cells but the impact of this will be limited by co-treatment with durvalumab. AZD6738 also caused a significant increase in tumoural MHC class I expression. Importantly, monotherapy caused an increase in the levels of the key chemotactic chemokine CCL5. The pattern of up-regulation of the cytosolic nucleic acid sensors and the degree of elevation was almost identical for AZD6738 and ionising radiation with up-regulation of ZbP1 a DNA sensor that stimulates type I interferon production and signals via IRF3 and IRF7 (6), DDX60, another cytosolic DNA sensor and the RNA sensor Ifih1. There were also significant increases in Irf7. Thus, AZD6738 drives DNA damage alongside up-regulation of the molecules that sense that damage and activates a pattern of IRF and nucleic acid recognition molecule up-regulation that is very similar to that seen with radiation but where this effect will be systemic. Thus, AZD6738 has the potential to adjuvantise the microenvironment and synergise with checkpoint blockade using durvalumab.

RAS mutation drives chromosomal instability (7). A significant number of RAS transformed cells pass through mitosis with unresolved DNA damage and micronuclei are abundant in fully RAS transformed cells and those early RAS transformants that avoid apoptosis. Importantly oncogenic RAS activates ATR (8). This suggests that ATR activation might serve to limit genomic instability in RAS mutant cells and in particular allow time to thus limit entry into mitosis with unresolved genome damage. When ATR was inhibited in RAS mutant cells □H2AX levels increased as did the sister chromatid exchange rate and there was a significant increase in chromatid breaks in the M phase (8). ATR inhibition lead to a much greater anti-proliferative effects in RAS mutant versus wild type cells suggesting synthetic lethality whereby the level of genomic instability becomes inconsistent with cell survival. Beyond, this effect however, it is clear that the use of AZD6738 in a RAS mutant background will maximise entry into mitosis with unresolved DNA damage and thus maximise the activation of cytosolic DNA sensing with potent local immunostimulatory effects. The resulting local accumulation of T cells can then be enabled by the addition of an immune checkpoint blockade agent. Finally, in KRAS driven mouse models AZD6738 resulted in significant reductions in the levels of both intratumoural and systemic proliferating Tregs (9). Hence, we will test specifically the combination of AZD6738 and the PD-L1 blockade antibody durvalumab in RAS mutant NSCLC. Ongoing dose escalation studies have demonstrated good tolerability of this

combination with clear evidence of activity in biomarker unselected patients. A critical component of this sub-study will be to examine the activity of the combination according to KRAS sub-type (10). Recent data demonstrates that patients with dual KRAS/p53 mutation have twice the PD-L1 Tumour Proportion Score (TPS) and clinical response to immune checkpoint blockade compared with KRAS single mutant lung cancer patients underscoring the impact of the loss of a cell cycle checkpoint on immune reactivity in RAS mutant NSCLC (11). Dual LKB1/KRAS mutants however have low levels PD-L1 reactivity and respond poorly to PD-1/PD-L1 blockade and thus we will specifically examine the durable clinical response rate with the AZD6738/durvalumab combo according to KRAS sub-type.

The background and rationale for the use of durvalumab to treat NSCLC can be found in Section 31.1 COHORT NA1: Durvalumab – Anti-PDL1 *(Closed to Recruitment)*.

30.1.1 Pre-Clinical Experience

The pre-clinical experience is fully described in the current version of the AZD6738 Investigator's Brochure (IB).

30.1.2 Clinical Data

Clinical data for durvalumab can also be found in Section 31.1 COHORT NA1: Durvalumab – Anti-PDL1 *(Closed to Recruitment)*. Clinical experience with AZD6738 in monotherapy and in combination is fully described in the current version of the AZD6738 and durvalumab Investigator's Brochure. All text in this section will refer to AZD6738 or its specific combination with durvalumab:

30.1.2.1 Safety

30.1.2.1.1 AZD6738 safety profile

As of the data cut-off of 13 June 2019, approximately 425 patients have received AZD6738 in ongoing AstraZeneca sponsored open-label clinical studies as monotherapy and in combination. Of these 37 patients received AZD6738 in combination with durvalumab.

Observed toxicities in the clinical setting have been manageable with current clinical practice and none of the events has been fatal. The following have been recognised as a potential risk for AZD6738 alone or in combination, based on toxicology data and the safety profile of the drug:

Bone marrow & haematological effects

Thrombocytopenia and anaemia have been reported during clinical use of AZD6738 as monotherapy, and thrombocytopenia, neutropenia and anaemia when AZD6738 is used in combination with myelosuppressive agents. The timing of observed nadirs, especially of platelets, when AZD6738 is used in combination with myelosuppressive drugs appears to vary by schedule as well as dose of AZD6738. This remains the subject of investigation in ongoing clinical studies and, at least for some schedules, may occur later than might be expected from administration of the myelosuppressive agent alone.

In monotherapy, the incidence of haematological toxicity (anaemia, thrombocytopenia and additionally neutropenia) is comparatively higher when ceralasertib was administered in patients with haematological malignancies (Study D5330C00008 - chronic lymphocytic leukaemia) compared to solid tumours. A SUSAR was reported from the PATRIOT Study (D5330C00002, NCT02223923). A patient in the AZD6738 240 mg bd monotherapy cohort experienced severe pancytopenia towards the end of the third treatment cycle. The Investigators' Brochure quotes bone marrow toxicity in animals, but this is the first case of pancytopenia reported in humans treated with single agent AZD6738.

Adverse events of G≥3 hematologic toxicity including anemia, thrombocytopenia, neutropenia (including febrile neutropenia) have been reported in 4 of 8 patients recruited into AstraZeneca sponsored studies (PLANETTE, D5339C00001, and HUD-SON, D6185C00001), which are using the 240 mg BD dose of AZD6738 for 14 days.

Febrile neutropenia

Febrile neutropenia or fever together with neutropenia (low neutrophil count) has been seen in a small number of subjects receiving AZD6738 in combination with other anticancer drugs that also affect the bone marrow.

Gastrointestinal disorders

In general, GI symptoms were reported more commonly across the combination arms when compared to AZD6738 monotherapy. These events were mostly resolved without modification to study therapy. One patient receiving ceralasertib in combination with olaparib discontinued study treatment due to GI AEs Grade 2 nausea and vomiting. The most frequently reported GI AE across monotherapy and all combinations was nausea. The majority of these AEs were G1 to 2, which required treatment with anti-emetics in approximately half of these patients, with a small number of patients requiring steroid treatment. Vomiting was reported in approximately a third of patients receiving AZD6738 in either of the various combinations. The majority of these AEs were G1 to 2.

Diarrhoea was reported in approximately 25% patients receiving AZD6738 in either of the various combinations. These AEs were G1 to 2.

Cardiovascular disorders

Peripheral vasodilatation with subsequent reflex haemodynamic effects, including lower blood pressure values has been observed in some animal models (for more information please refer to Investigator's Brochure Hypotension which was reported in just under 15% of patients when AZD6738 was administered in combination with durvalumab and just under 5%of patients when AZD6738 was administered in combination with olaparib. In general, these events were mostly unrelated G1 to 2 in severity and did not require any study drug dose modification or treatment for the eventSyncope was reported in 2 patients who were treated with AZD6738 in combination with olaparib). Pre-syncope was reported in 1 patient receiving AZD6738 monotherapy. All of these events were non-serious, considered to be unrelated to the study treatments by the investigators and resolved without treatment. No clinically significant blood pressure/ECG changes were noted at the time of the events.

There have been isolated events of QT prolongation, increased heart rate and palpitations when AZD6738 was used in combination with olaparib or durvalumab. Events were mainly G1 in severity. There was one event of G3 QT prolongation in the olaparib combination, which was considered to be unrelated to the study treatment by the investigator and 2 events of Grade 1 QT prolongation which were considered related to both ceralasertib and olaparib, both requiring a dose interruption. No patients withdrew due to cardiovascular AEs. Additionally, one SAE of QT prolongation (G1) was reported in the externally sponsored study D5330C00002 (PATRIOT) which resulted in a dose reduction of AZD6738. Concurrent administration of domperidone was considered co-suspect by the investigator.

Hepatobiliary effects

Treatment emergent elevations in ALT, aspartate aminotransferase (AST), bilirubin and gamma-glutamyl transpeptidase/transferase(GGT) have been observed when AZD6738 was used in combination. There were no significant hepatic AEs or injury associated with these laboratory changes. No patients withdrew due to hepatic AEs.

Lungs

When AZD6738 was used in combination with olaparib or durvalumab, cough and dyspnoea were seen in approximately 13% of patients, cough were the most commonly reported adverse events. All of these events were G1 to 2 in severity and mostly unrelated. In ongoing Study D5330C00004, 1 patient receiving ceralasertib in combination with olaparib experienced dyspnoea with a reported fatal outcome. The case information is limited however the patient's underlying metastatic malignancy could be a possible contributor for development of the other reported events of thrombotic microangiopathy and veno-occlusive disease, which could all together explain the occurrence of dyspnoea in this case. The event of dyspnoea was considered related to ceralasertib; however, this occurred in the context of disease progression and the other events listed above. In the ongoing ESR Study D5330C00002 (PATRIOT), there has been 1 case of pneumonitis.

Photosensitising potential

Nonclinical evaluation of AZD6738 for phototoxic potential produced positive *in vitro* but negative *in vivo* findings. Two cases of sunburn in patients receiving AZD6738 have been reported. One event was reported as a G3 maculo-papular rash occurring with AZD6738 in combination with olaparib and was treated with oral antihistamines and topical hydrocortisone. The other event was from the externally sponsored Study D5330C00002 (PATRIOT) and was reported as a serious G3 photosensitivity.

30.1.2.1.2 Durvalumab safety profile

As of the data cut-off date of 12 July 2019, an estimated 8817 patients have received durvalumab in AstraZeneca or MedImmune-sponsored interventional studies in multiple tumour types, stages of disease and lines of therapy. Of these, 4067 patients received durvalumab as a monotherapy, 2423 in combination with tremelimumab and 2327 in combination with any investigational and/or approved product. In addition, 2482 patients have participated in the durvalumab Early Access Programme (EAP; Study D4194C00002) for patients with locally advanced, resectable NSCLC whose disease has not progressed following platinum based chemoradiation therapy. The cumulative global post-marketing patient exposure to durvalumab (10 mg/kg) to 30 June 2019 has been estimated to be approximately 12385 patient-years. The safety profile of durvalumab as monotherapy and combined with other anticancer agents is consistent with the pharmacology of the target and other agents in the immune checkpoint inhibitor class. Most adverse drug reactions (ADRs) seen with the immune checkpoint inhibitor class of agents are thought to be due to the effects of inflammatory cells on specific tissues and could occur in any organ system.

30.1.2.2 Efficacy

A total of 37 patients have been dosed to date with AZD6738 in combination with durvalumab in the Study D5330C00004. Five cohorts have been tested and have been declared tolerated, with Cohort 6 ongoing. Responses have been seen across Cohorts 1 to 5 (response assessments in Cohort 6 were awaited at the time of data cut-off). In total, as assessed by RECIST 1.1, there have been three confirmed partial responses (two NSCLC patients and one squamous cell carcinoma of the head and neck [SCCHN]), one unconfirmed response (NSCLC) and one confirmed complete response in a NSCLC patient. In addition, 12 cases of stable disease have been seen (unclean data, data cut-off 13 June 2018).

30.1.2.3 Pharmacokinetics

At the time of writing, preliminary PK data is available from the on-going studies D5330C00004 (Modules 1, 2 and 3) and ESR Study D5330C00002 in the Investigator Brochure for AZD6738. No metabolism data are available in humans. Pharmacokinetic properties of AZD6738 were evaluated in patients with advanced solid tumours in Studies D5330C00002 (PATRIOT) and D5330C00004. PK samples have been analysed for 35 patients in D5330C00004 Module 3 who have received AZD6738 with durvalumab.

AZD6738 was rapidly absorbed following oral dosing in fasting conditions, with a peak plasma concentration observed typically after 1.5 h post dose, and declined in a biphasic manner with a typical elimination half-life of ~11 h. Despite a high inter-individual variability (60% to 70%), there was an apparent dose proportional increase in AZD6738 plasma concentrations across the tested doses. A graphical preliminary exploration of AZD6738 plasma concentrations did not suggest any PK impact by either carboplatin, olaparib or durvalumab. In addition, based on the PK data from the combinatory agents, it is unlikely that AZD6738 will affect the PK of carboplatin or olaparib. Durvalumab levels have not been analysed to date, however, the in vitro DDI predictions did not suggest any DDI between AZD6738 and durvalumab.

In vitro data suggest metabolic clearance of AZD6738 is via CYP3A4 (75%) and CYP2C8 (25%) and it is a substrate for P-gp and BCRP transporters. There is therefore a risk that the PK of ceralasertib may be affected by co-administration with inducers and/or inhibitors of these enzymes and transporters. ADZ6738 is an in vitro inhibitor of P-gp (IC50 200 μM), BCRP (IC50 36.1 μM), OATP1B1 (IC50 3.6 μM), OATP1B3 (IC50 61.2μM), MATE1 (IC50 24.3 μM) and MATE2K (IC50 3.5 μM). Drug interaction assessment based on human PK indicates that at higher doses ceralasertib may influence the PK of P-gp, BCRP, OATP1B1, OATP1B3, MATE1 and MATE2K substrates.

30.1.2.4 Pharmacodynamics

No Pharmacodynamics data are available for AZD6738 or durvalumab.

30.1.2.5 Marketing Experience

AZD6738 has not been approved for marketing purposes anywhere in the world.

Durvalumab has marketing authorisation within EU and USA for treatment of locally advanced, unresectable NSCLC and metastatic urothelial cancer.

30.1.2.6 Cohort Definition

30.1.2.6.1 J1: KRAS mutation

KRAS mutation. Patients must have a successful SMP2 test result available for STK11/LKB1.

30.1.2.6.2 NAJ: No actionable genetic change

Patients must have a successful SMP2 result of ≥90% of core genes (see 4 Molecular Eligibility Rules for core genes list) being reported with no other actionable genetic change currently offered within the protocol.

30.2 Specific Eligibility Criteria

Treatment can potentially be delivered as first or second line therapy, but it is strongly recommended that patients receive all standard of care systemic anti-cancer therapy before entering this arm.

30.2.1 Inclusion Criteria

For inclusion in the trial, patients must fulfil all of the following criteria:

- Patients must fulfil all the core eligibility criteria.
- A successful SMP2 result:
  - An SMP2 result is defined as successful when ≥90% of core genes (see Section 4 Molecular Eligibility Rules) have been successfully reported in the Technology Hub report. For clarity, this can be either the diagnostic test or a repeat biopsy test.
  - Additional molecular inclusion rules apply for cohort J1 (see Section 4 Molecular Eligibility Rules).
- Eastern Cooperative Oncology Group (ECOG) performance status 0 or 1 with no deterioration over the previous 2 weeks and an estimated life expectancy of greater than 12 weeks.
- Body weight of 30.1 Kg or more.
- Ability to swallow and retain oral medication.

30.2.2 Exclusion Criteria

Patients must not enter the trial if any of the following exclusion criteria are fulfilled:

- Patients who do not fulfil all the core eligibility criteria.
- Treatment with any of the following:
  - Blood transfusion within 2 weeks prior to registration.
  - Platelet transfusion or erythropoietin within 4 weeks prior to registration (erythropoietin use >4 weeks is permitted).
  - Cytotoxic treatment within 4 weeks of registration.
  - Corticosteroids (at a dose > 10 mg prednisone/day or equivalent) for any reason within 2 weeks of registration.
  - Hormonal or non-hormonal targeted therapy within 3 weeks of registration.
  - Immunotherapy within 6 weeks of registration.
  - Any small molecule investigational medicinal product (IMP) within 4 weeks of registration.
  - Concomitant medications, herbal supplements and/or foods that significantly modulate CYP3A4 or Pgp activity (wash out periods of 2 weeks, but 3 weeks for St. John's Wort). Note these include common azole antifungals, macrolide antibiotics.
  - Any other non-cytotoxic drugs or IMPs within 4 weeks or five half times, whichever is longest.
  - Radiotherapy with a limited field of radiation for palliation within 1 week of the registration, with the exception of patients receiving radiation to more than 30% of the bone marrow or with a wide field of radiation within 4 weeks of

registration. (The patient can receive a stable dose of bisphosphonates or denosumab for bone metastases, before and during the trial as long as these were started at least 5 days prior to the trial treatment).
    - An additional immunosuppression other than corticosteroids for the management of an AE. Patient must not have experienced recurrence of an AE if rechallenged, or require maintenance doses of > 10 mg prednisone or equivalent per day (patients receiving a stable dose of corticosteroids started at least 4 weeks prior to registration is permitted).
    - Prior exposure to an ATR inhibitor.
    - Live virus and bacterial vaccine within 30 days of registration.
- Patients who have received prior anti-PD-1, anti PD-L1 or anti CTLA-4:
    - Must not have experienced a toxicity that led to permanent discontinuation of prior immunotherapy.
    - Must have complete resolution of all AEs while receiving prior immunotherapy, or resolved to baseline prior to screening for this trial.
    - Must not have experienced a ≥ Grade 3 immune related AE or an immune related neurologic or ocular AE of any grade while receiving prior immunotherapy. NOTE: Patients with endocrine AE of ≤ Grade 2 are permitted to register if they are stably maintained on appropriate replacement therapy and are asymptomatic.
    - Must not have required the use of additional immunosuppression other than corticosteroids for the management of an AE, not have experienced recurrence of an AE if re-challenged, and not currently require maintenance doses of > 10 mg prednisone or equivalent per day.
- A known allergy or hypersensitivity to AZD6738, durvalumab or any excipient of the products.
- Any contraindication to the combination anti-cancer agent as per local prescribing information.
- Cardiac dysfunction as defined as: Myocardial infarction within six months of registration, NYHA Class II/III/IV heart failure, unstable angina, unstable cardiac arrhythmias or reduced LVEF < 55%.
- Any of the following cardiac criteria:
    - Mean resting corrected QT interval (QTc) >470 msec obtained from 3 electrocardiograms (ECGs) in 24 hours.
    - Any clinically important abnormalities in rhythm, conduction or morphology of resting ECG (e.g., complete left bundle branch block, third degree heart block).
    - Any factors that increase the risk of QTc prolongation or risk of arrhythmic events such as heart failure, hypokalaemia, congenital long QT syndrome, immediate family history of long QT syndrome or unexplained sudden death under 40 years of age.
    - Patients at risk of brain perfusion problems, e.g., carotid stenosis.
    - Patients with relative hypotension (< 100/60 mm Hg) or clinically relevant orthostatic hypotension, including a fall in blood pressure of >20mm Hg.
    - Uncontrolled hypertension requiring clinical intervention.
- Any other malignancy which has been active or treated within the past 3 years, with the exception of cervical intra-epithelial neoplasia and non-melanoma skin cancer, ductal Carcinoma in Situ (DCIS), stage 1 grade 1 endometrial carcinoma, or other solid tumours curatively treated with no evidence of disease for ≥ 5 years prior to trial entry (including lymphomas [without bone marrow involvement].
- Refractory nausea and vomiting, chronic gastrointestinal diseases or previous significant bowel resection, with clinically significant sequelae that would preclude adequate absorption of AZD6738.

- As judged by the Investigator, any evidence of severe or uncontrolled systemic diseases that places the patient at unacceptable risk of toxicity or non-compliance. Examples include, but are not limited to:
    - Active bleeding diatheses, renal transplant, uncontrolled seizures, severe COPD, superior vena cava syndrome, extensive bilateral lung disease on High Resolution CT scan, severe Parkinson's disease, refractory nausea or vomiting, irritable bowel syndrome, chronic gastrointestinal disease, significant bowel resection, psychiatric condition, or active infection including any patient known to have tuberculosis, hepatitis B, hepatitis C and human immunodeficiency virus (HIV) or requiring systemic antibiotics, antifungals or antiviral drugs. (Screening for chronic conditions is not required).
- A diagnosis of ataxia telangiectasia.
- Patients with uncontrolled seizures.
- Concurrent severe and/or uncontrolled medical condition (e.g., severe COPD, severe Parkinson's disease, active inflammatory bowel disease) or psychiatric condition (screening for chronic disease is not required).
- History of sarcoidosis.
- Known history of tuberculosis.
- History of non-infectious pneumonitis requiring steroids or has active pneumonitis or significantly reduced transfer coefficient (KCO).

30.2.3 Restrictions & Concomitant Medications

Information on any treatment from the date of informed consent until 28 days after the administration of the last treatment dose should be recorded. If medically feasible, patients taking regular medication should be maintained on it throughout the trial period. Patient should be advised to inform their treating physicians of all concomitant medications, including prescription medicines, over-the-counter drugs, vitamins, and herbal products.

Nb. These lists are not exhaustive and the absence of a drug from the lists does not imply that its combination with AZD6738 and/or durvalumab is safe.

30.2.3.1 Drugs affecting CYP3A4 metabolism AstraZeneca strongly recommend are not combined with AZD6738 trial treatment

- The principal enzyme for metabolising AZD6738 is CYP3A4. Patients should avoid concomitant drugs, herbal supplements and/or ingestion of foods known to modulate CYP3A4 activity, from the time they enter the screening period until 28 days after the last dose of trial medication.
- For patients taking any strong (potent) CYP3A4 inducers the required wash-out periods prior to starting AZD6738 is 2 weeks, except for St. John's Wort, which is 3 weeks.
- Aprepitant (Emend) is a substrate, moderate inhibitor and inducer of CYP4A4 and also an inducer of CYP2C9 therefore should not be used for the treatment of nausea and vomiting induced by the trial drugs.
- If the use of any strong inducers or inhibitors of CYP3A4 are considered necessary for the patient's safety and welfare, the Investigator must contact the Trials Office and a decision to allow the patient to continue in the trial will be made on a case-by-case basis. If the patient is continuing to receive AZD6738 on the basis of clinical benefit, as judged by the Investigator, and in the absence of discontinuation criteria, if the

Investigator feels that concomitant administration of medications, herbal supplements or foods that significantly modulate CYP3A4 activity is necessary based upon medical judgement, such products may be administered with caution following discussion between the Principal Investigator, Trials Office and Lead Investigator.

Table 79: Drugs known to be inhibitors and inducers of CYP3A

Potent CYP3A inhibitors		Potent CYP3A inducers
boceprevir	mibefradil	apalutamide
ceritinib	mifepristone	avasimibe
clarithromycin	nefazodone	carbamazepine
cobicistat (GS-9350)	nelfinavir	enzalutamide
conivaptan	posaconazole	ivosidenib
danoprevir/RIT	ribociclib	lumacaftor
elvitegravir/RIT	ritonavir	mitotane
grapefruit juice[2]	saquinavir	phenobarbital
idelalisib	saquinavir/RIT	phenytoin
indinavir	telaprevir	rifampin
indinavir/RIT	telithromycin	rifapentine
itraconazole	tipranavir/RIT	St John's Wort extract
ketoconazole	troleandomycin	
LCL161	VIEKIRA PAK2[1]	
lopinavir/RIT	voriconazole	

List created using the University of Washington Drug-Drug Interaction Database July 2019.
RIT=Ritonivir. Ritonavir has dual effects of simultaneous CYP3A inhibition and induction, and the net pharmacokinetic outcome during chronic ritonavir therapy is inhibition of CYP3A activity
[1] VIEKIRA PAK = 150/100 mg paritaprevir/ritonavir + 25 mg ombitasvir + 800 mg dasabuvir for 28 days.
[2]. Double-strength grapefruit juice.

30.2.3.2 Drugs affecting CYP3A4 and CYP2B6 metabolism AstraZeneca considers may be allowed with caution

- AZD6738 is a potential inducer of both CYP3A4 and CYP2B6. Caution should be applied with co-administration of drugs that are either completely metabolised by CYP3A4 and/or CYP2B6 or that are substrates of CYP3A4 and/or CYP2B6 and also have a narrow therapeutic index.

Table 80: Drugs known to be metabolised by CYP3A4 and have a narrow therapeutic index

Alfentanil	Quinidine
Cyclosporine	Sirolimus
Dihydroergotamine	Tacrolimus
Ergotamine	Astemizole
Fentanyl	Cisapride
Pimozide	Terfenadine

Table 81: Drugs known to be metabolised by CYP2B6 and have a narrow therapeutic index

Cyclophosphoamide	methadone
Ifosfamide	methoxetamine
Efavirenz	nevirapine
Bupropion	propofol
Propofol	selegiline
Thiotepa	sertraline
Sorafenib	sorafenib
alfentanil	tamoxifen
ketamine	valproic acid

From Flockhart DA (2007). "Drug Interactions: Cytochrome P450 Drug Interaction Table". Indiana University School of Medicine.

30.2.3.3 Concomitant radiotherapy & surgical procedures

- Radiotherapy with a limited field of radiation given palliatively requires a washout period of 1 week prior to registration. Patients who have received radiation to more than 30% of the bone marrow or with a wide field of radiation require a 4 week washout period prior to registration.
- Concurrent radiotherapy (except palliative radiotherapy – see below) should not be given whilst the patient is on trial treatment.
- Palliative radiotherapy may be used for the treatment of pain at the site of bony metastases that were present at baseline, provided the Investigator does not feel that these are indicative of clinical disease progression during the trial period.
- Local treatment of isolated lesions, excluding target lesions, for palliative intent is also acceptable (e.g. by local surgery or radiotherapy).
- Trial treatment should be discontinued for a minimum of 3 days before a patient undergoes therapeutic palliative radiation treatment. Trial treatment should be restarted within 1-4 weeks as long as any bone marrow toxicity has recovered.
- Trial treatment should be discontinued for a minimum of 3 days before a patient undergoes planned surgery. Treatment with durvalumab may resume once the wound has healed. Treatment with AZD6738 should be resumed 10 days post-surgery if the wound has healed. If the wound is not healed, a further 7 days may be allowed and the patient may recommence AZD6738 following discussion with the Trials Office & Lead Investigator.
- No stoppage of trial treatment is required for any needle biopsy procedure.

30.2.3.4 Other anti-cancer treatment

- Hormonal or non-hormonal targeted therapy should not be given within 3 weeks of registration.
- Immunotherapy should not be given whilst the patient is on treatment and within 6 weeks of registration.
- Patients must not receive any other investigational drugs whilst on this trial or within 4 weeks or five half-lives (whichever is greater) of registration.
- Small molecule IMPs must not be given within 4 weeks prior to registration.

- Any other concurrent chemotherapy or biologic for cancer treatment should not be given whilst the patient is on trial treatment and for 4 weeks following discontinuation.
- Patients may receive bisphosphonates or denosumab for bone metastases before and during the trial, as long as they were started at least 5 days prior to trial treatment.

30.2.3.5 Immunosuppressive medications (including steroids)

- Immunosuppressive medications including, but not limited to systemic corticosteroids at doses exceeding 10 mg/day of prednisone or equivalent, methotrexate, azathioprine, and tumour necrosis factor (TNF)-α blockers are not allowed during treatment and for 2 weeks prior to registration.
- Doses $\leq$10mg/day are permitted but should not be given concomitantly, or used for premedication prior to the durvalumab infusions. The following are allowed exceptions:
    - Use of immunosuppressive medications for the management of investigational product-related AEs.
    - Use in subjects with contrast allergies.
- Use of inhaled and intranasal corticosteroids is permitted.
- A temporary period of steroids will be allowed at the discretion of the Principal Investigator, if clinically indicated and considered essential for the management of the patient (e.g., chronic obstructive pulmonary disease, radiation, nausea, etc).

30.2.3.6 Viruses

- Live virus and bacterial vaccines should not be administered 30 days prior to registration, during treatment and for 30 days post discontinuation of treatment. Inactivated viruses, such as those in the influenza vaccine, are permitted.
- An increased risk of infection by the administration of these vaccines has been observed with conventional chemotherapy and the effects with AZD6738 are unknown.
- Live virus and bacterial vaccines include (but are not limited to) yellow fever, measles, influenza, rubella, mumps, typhoid, mycobacterium tuberculosis (BCG), yersinia pestis (EV).
- The administration of killed vaccines is allowed. Examples of killed vaccines are cholera, bubonic plague, polio vaccine, hepatitis A and rabies.

30.2.3.7 Anticoagulant therapy

- Patients who are taking warfarin may participate in this trial; however, it is recommended that prothrombin time (international normalised ratio (INR) and activated partial thromboplastin time (APTT)) be monitored carefully at least once per week for the first month, then monthly if the INR is stable.
- Subcutaneous heparin is permitted.
- Investigators should carefully assess the risk in relation to benefit of using anticoagulation or other medications that might exacerbate bleeding during trial participation. Investigators should consider discontinuation of these agents if the clinical scenario permits it unless discontinuation would be clinically contraindicated. For example, if the clinical condition for which anticoagulation/NSAIDs/aspirin is/are being used may be managed with an alternate agent/s that has a lower risk of bleeding, a substitution should be considered.

30.2.3.8 Pgp inhibitors or inducers

- Patients who have received strong modulators of Pgp activity must have discontinued for at least 2 weeks prior to AZD6738 treatment.
- AZD6738 is also a Pgp substrate. Co-administration of Pgp inhibitors or inducers may affect exposure to AZD6738 and therefore it is strongly recommended these are not co-administered with AZD6738.
- If the use of any inhibitors or inducers of Pgp are considered necessary for the patient's safety and welfare, the Investigator must contact the Trials Office and a decision to allow the patient to continue in the trial will be made on a case-by-case basis. The Investigator will be required to interrupt AZD6738 for the duration of the Pgp inhibitor or inducer and wait for the required washout period of the Pgp modulator (five half-lives or 2 weeks) before dosing AZD6738 again.

30.2.3.9 Other interactions

- AZD6738 is also a substrate of BCRP. Co-administration of BCRP inhibitors or inducers may affect exposure to AZD6738; therefore, it is recommended that the Investigator interrupts AZD6738 for the duration of the BCRP inhibitor or inducer and wait for the required wash-out period of the BRCP modulator (five half-lives or 2 weeks) before dosing AZD6738 again.
- AZD6738 is an inhibitor of OATP1B1. Co-administration of substrates of OATP1B1 may affect exposure to AZD6738; therefore, it is recommended that caution should be applied when such drugs are to be administered with AZD6738.
- The use of any drugs with laxative properties, natural/herbal products or other traditional remedies should be discouraged up to 90 days of taking trial medication.
- Patients should not donate blood or blood components while participating in this trial and for 180 days after receipt of the final dose of trial treatment or until alternate anti-cancer therapy is started.
- Concurrent use of hormones for non-cancer-related conditions (e.g., insulin for diabetes and hormone replacement therapy) is acceptable.
- For all patients, it is prohibited to consume grapefruit juice or Seville oranges (including marmalade, juice, etc.) while participating in the trial.
- AZD6738 may increase the skin's sensitivity to sunlight, which may result in sunburn. Patients should therefore take appropriate precautions when out in the sun (e.g. limit exposure, wear appropriate clothing and sunscreen).
- If no clinically significant blood pressure (BP) changes are seen following the first dose of AZD6738 (cycle 1 day 15), then no restrictions with regard to driving are required. Where significant BP changes are seen, the patient should be instructed not to drive or operate heavy machinery and the Trial Office and Lead Investigator should be consulted for further guidance and reporting to AstraZeneca.
- All other concomitant medications, other than those described above, including vitamins, and nutritional supplements, which are considered necessary for the patient's safety and well-being, may be given at the discretion of the Investigator but must be recorded in the appropriate sections of the CRF.

30.2.3.10 Contraception

A definition of females of childbearing potential and females of non-childbearing potential is available in Section 6.3.1.

Appropriate methods of contraception
Contraceptives that are prone to drug-drug interactions may not be effective due to a potential CYP3A interaction with AZD6738. Contraception used must therefore include a condom with spermicide **and** one of the following:

- Medroxyprogesterione injections (e.g. Depo-provera)
- Intrauterine Device (IUD)
- Levonorgestrol Intrauterine System (e.g. Mirena)
- Tubal occlusion
- Vasectomised partner

The following restrictions apply while the patient is receiving trial treatment and for the specified times before and after:

Female patient of child-bearing potential

- Females of childbearing potential who are sexually active with a non-sterilised male partner must agree to use 2 highly effective methods of contraception in combination from the time of screening and must agree to continue using such precautions for at least 90 days after the last dose of AZD6738 and durvalumab.
- This includes use of a male condom plus spermicide for non-sterilised male partners of a female patient throughout this period.
- Not engaging in sexual activity for the total duration of the drug treatment and the drug washout period is an acceptable practice; however, periodic abstinence, the rhythm method, and the withdrawal method are not acceptable methods of birth control.
- Female subjects should not breastfeed throughout this period.

Male subjects with a female partner of childbearing potential

- Non-sterilised males who are sexually active with a female partner of childbearing potential must use a male condom plus spermicide from screening through 180 days after receipt of the final dose of AZD6738 and durvalumab.
- Not engaging in sexual activity is an acceptable practice; however, occasional abstinence, the rhythm method, and the withdrawal method are not acceptable methods of contraception. Male subjects should refrain from sperm donation throughout this period.
- Female partners (of childbearing potential) of male subjects must also use a highly effective method of contraception throughout this period.

30.3 Trial Treatment

30.3.1 Investigational Medicinal Product

30.3.1.1 AZD6738

AZD6738 (also known as ceralasertib) is available for administration as a film coated tablet for use in clinical trials. The tablet is available in three strengths: 100 mg, 80 mg and 20 mg tablet. AZD6738 80 mg will be provided by AstraZeneca free of charge.

A cycle of trial treatment will be defined as 28 days. AZD6738 will be administered orally at a dose of 240 mg twice daily (BD) on days 15-28 of each cycle. Intermittent dosing is recommended because of improved tolerability with fewer patients stopping treatment as a consequence of toxicity.

Patients must adhere to the following instructions while receiving trial treatment and for the specified times before and after:

- Patients must fast (water to drink only) from at least 2 hours prior to taking a dose to at least 1 hour post-dose for all doses.
- AZD6738 tablets must be taken whole with water.
- Patients may take their medication up to 2 hours late. If later than 2 hours, they should wait until the next scheduled time.
- If a patient vomits after taking tablets, they should not retake the medication unless they can clearly identify all of the drug tablets.

30.3.1.2 Durvalumab

Durvalumab (also known as MEDI4736) is a human monoclonal antibody of the immunoglobulin (Ig) G1 kappa subclass that inhibits binding of PD-L1 (B7-H1, CD274) to PD-1 (CD279) and CD80 (B7-1) and is in development for the treatment of patients with various malignancies including NSCLC. Durvalumab will be supplied free of charge by AstraZeneca in 500 mg vials.

Patients receiving durvalumab will commence treatment (1500 mg IV) on day 1 of every cycle until confirmed disease progression, unless there is unacceptable toxicity, withdrawal of consent, or another discontinuation criterion is met (please note patients who meet RECIST criteria for progressive disease (PD) may be continued on trial treatment if the treatment is tolerable and the Investigator believes it to be of clinical benefit; see Section 9.3 Patient Withdrawal).

Patients who weigh less than 30.1 kg at screening will not be eligible for trial entry. Weight-based dosing should be utilised for patients whose weight falls below 30.1 kg during the trial. The dose will be 20mg/kg for these patients. See Appendix 13: Durvalumab Weight-Based Dose Calculation.

A dose of 1500 mg (for patients $\geq$30.1 kg in weight) will be administered using an IV bag containing 0.9% (w/v) saline or 5% (w/v) dextrose, with a final durvalumab concentration ranging from 1 to 15 mg/mL and delivered through an IV administration set with a 0.2- or 0.22-µm in-line filter. Add 30.0 mL of durvalumab (i.e., 1500mg of durvalumab) to the IV bag. The IV bag size should be selected such that the final concentration is within 1 to 15 mg/mL. Mix the bag by gently inverting to ensure homogeneity of the dose in the bag.

Standard infusion time is 1 hour. In the event that there are interruptions during infusion, the total allowed infusion time should not exceed 8 hours at room temperature. If either preparation time or infusion time exceeds the time limits a new dose must be prepared from new vials. Durvalumab does not contain preservatives, and any unused portion must be discarded.

Do not co-administer other drugs through the same infusion line. The IV line will be flushed with a volume of IV diluent equal to the priming volume of the infusion set used, after the contents of the IV bag are fully administered or complete the infusion according to institutional policy to ensure the full dose is administered.

Durvalumab will be supplied by AstraZeneca R&D supply chain in a 500mg vial solution for infusion. The solution contains 50 mg/mL durvalumab, 26 mM histidine/histidine HCl, 275 mM trehalose dihydrate, 0.02% (w/v) polysorbate 80, at pH 6.0. The nominal fill volume is 10.0 mL. Investigational product vials are stored at 2°C to 8°C (36°F to 46°F) and must not be

frozen. Drug product should be kept in original packaging until use to prevent excessive light exposure.

30.3.2 Schedule of Assessments

Table 82: AZD6738 & Durvalumab – Schedule of Assessments

	**Screening**	**Treatment – AZD6738 240 mg & Durvalumab** 28 day cycle						**Discontinuation** (+ 7 days)**	**28-day Follow Ups**[s] (+ 7 days)**	**Survival Assessment** (± 7 days)***
	Within 28 days of treatment (unless otherwise stated)	Cycles 1 & 2				Cycle 3 onwards				
		Day 1 ****	Cycle 1 Day 8 (± 2 days)*	Day 15 (± 2 days)*	Day 22 (± 2 days)*	Day 1 (± 2 days)*	Day 15 (± 2 days)*			
Informed consent[a]	X									
Demography & baseline characteristics[b]	X									
Medical history[c]	X									
Inclusion / exclusion criteria[d]	X									
Physical examination[e]	X	X (-1 day)	X	X		X	X	X	X	
ECOG performance status	X (within 14 days of treatment)	X (-1 day)				X		X		
Hepatitis B, C and HIV[t]	X									
Vital signs[f]	X	X (-1 day)		X		X	X	X		
Weight	X	X (-1 day)				X		X		
ECG[g]	X	X				X		X		
Haematology, Clinical chemistry[h]	X (within 3 days of treatment)	X (-1 day)	X (-2 days)	X (-2 days)	X (-2 days)	X (-2 days)	X (-2 days)	X	X (-2 days)	
Coagulation[h]	X	If clinically indicated / patient receiving warfarin								

	Screening	Treatment – AZD6738 240 mg & Durvalumab<br>28 day cycle						Discontinuation (+ 7 days)**	28-day Follow Ups[s] (+ 7 days)**	Survival Assessment (± 7 days)***
	Within 28 days of treatment (unless otherwise stated)	Cycles 1 & 2				Cycle 3 onwards				
		Day 1<br>****	Cycle 1 Day 8 (± 2 days)*	Day 15 (± 2 days)*	Day 22 (± 2 days)*	Day 1 (± 2 days)*	Day 15 (± 2 days)*			
Urinalysis[i]	X<br>(within 3 days of treatment)	X<br>(-1 day)	X<br>(-2 days)			X<br>(-2 days)		X	X<br>(-2 days)	
Thyroid Function and Cortisol Level Tests[j]	X	X<br>(-1 day)	X			X<br>(-2 days)		X	X	
Pregnancy test[k]	X	X<br>(-1 day)				X<br>(-2 days)		X		
Tumour assessments[l]	X	Every 8 weeks during year 1 (± 7 days) [except 1st scan + 7 days only]								
Adverse events & Concomitant Medications	X	X<br>(-1 day)	X	X	X	X	X	X	X	
Dispense AZD6738[m]				X			X			
Administer AZD6738[m]				BD orally d15-28			BD orally d15-28			
Dispense & Administer Durvalumab[m]		X				X				
Smoking status[n]		X<br>(every 8 weeks)<br>(-1 day)	Cycle 3 day 1 then every other cycle					X		
Germline DNA sample[o]		X<br>(-1 day)								
ctDNA samples[p]		X<br>(-1 day)	Cycle 3 day 1 then every other cycle					X		X $
Optional research biopsy[q]		X (post-reg, pre-tx)						X		

	Screening	Treatment – AZD6738 240 mg & Durvalumab 28 day cycle						Discontinuation (+ 7 days)**	28-day Follow Ups[s] (+ 7 days)**	Survival Assessment (± 7 days)***
	Within 28 days of treatment (unless otherwise stated)	Cycles 1 & 2				Cycle 3 onwards				
		Day 1 ****	Cycle 1 Day 8 (+ 2 days)*	Day 15 (+ 2 days)*	Day 22 (+ 2 days)*	Day 1 (+ 2 days)*	Day 15 (+ 2 days)*			
Survival status[r]										X

* Visit may occur ± 2 days of the planned visit date. Individual assessments may occur independently of the visit date where indicated in the table above. Where applicable and acceptable in accordance to local practices, visits may be performed by telephone or video call.

** Visit may occur + 7 days of the planned visit date.

*** Visit may occur ± 7 days of the planned visit date.

**** Some Cycle 1 Day 1 assessments can be carried out -1 day of the Docetaxel infusion to allow flexibility due to Covid-19 associated clinic visit restrictions.

a Prior to the start of any trial specific procedures, each patient must provide signed informed consent.

b Demography must be captured for all patients. Demographic data and other characteristics will include: date of birth, gender, race/ethnicity.

c A standard medical and surgical history will be obtained, including cancer diagnosis, staging and prior cancer treatment.

d Patients must not be registered unless all eligibility criteria (core and arm-specific) have been fully met.

e Physical examination includes general appearance, respiratory, cardiovascular, abdomen, skin, head and neck (including ears, eyes, nose and throat), lymph nodes, thyroid, musculo-skeletal (including spine and extremities), and neurological systems and should be performed at screening, day 1 and 15 of every cycle, at discontinuation and at 28-day follow up. A physical examination should also be performed on day 8 of cycle 1.

f Supine BP and pulse will be measured using a semi-automatic BP recording device with an appropriate cuff size, after the patient has rested for at least 5 minutes.

Patients will be monitored at screening, day 1 & day 15 of each cycle and at discontinuation. Screening, day 15 and discontinuation assessments will consist of a single measurement of blood pressure, pulse rate and temperature. During and after the durvalumab infusion, the same assessments of vital signs will take place but at the following times (based on a 60-minute infusion):

- At the beginning of the infusion (at 0 minutes)
- Every 30 minutes during the infusion (±5 minutes)
- At the end of the infusion (at 60 minutes ±5 minutes)
- Thirty and 60 minutes post the infusion (i.e., 90 and 120 minutes from the start of the infusion) (±5 minutes) for the first infusion only and then for subsequent infusions as clinically indicated.

If the infusion takes longer than 60 minutes then the vital signs assessments should follow the principles as described above or more frequently if clinically indicated.

Two or more BP readings should be taken at 2 minutes intervals and averaged. If the first 2 diastolic readings differ by more than 5 mmHg, then an additional reading should be obtained and averaged. After the first dose, the 1-hour observation period will not be required unless a subject experiences an infusion-related reaction.

g ECGs recorded during the screening period will be obtained in triplicate; ECGs recorded during the treatment phase will be single tracing. ECG to be performed at screening, day 1 of each treatment cycle and at discontinuation. Day 1 ECGs should be performed within 1-2 hours prior to durvalumab dosing.

Twelve-lead ECGs will be obtained after the patient has been resting supine for at least 10 minutes prior to times indicated. All ECGs should be recorded with the patient in the same physical position. Where triplicate ECGs are required (screening only), three ECG recordings should be taken at a minimum of 5 minute intervals (all three ECGs must be collected within 30 minutes).

A standardised ECG machine should be used and the patient should be examined using the same machine throughout the trial if possible. After paper ECGs have been recorded, the Investigator or designated physician will review each of the timed 12-lead ECGs on each of the trial days when they are collected and may refer to a local cardiologist if appropriate. A paper copy should be filed in the patient's medical records. If an abnormal ECG finding at screening is considered to be clinically significant by the Investigator, it should be reported as a concurrent condition. For all ECGs details of rhythm, ECG intervals (R-R, PR, QT and QRS) and an overall evaluation will be recorded. At screening, mean QTcF must be <470 msec.

h Coagulation samples (activated partial thromboblastin time (APTT) and international normalised ratio (INR)) to be collected at screening. Further samples to be collected as clinically indicated. If the patient is receiving warfarin, coagulation should be monitored carefully and samples should be collected at least once per week for the first month, then monthly if the INR is stable.

Haematology & clinical chemistry samples to be collected at screening, day 1 & day 15 of each cycle, discontinuation and 28 day follow up. Also at day 22 of cycles 1 and 2. Samples can be taken up to 2 days earlier than the actual visit date (where indicated) with the exception

of Cycle 1 Day 1. Where applicable and acceptable in accordance to local practices, blood tests can be performed locally in GP surgeries or in community based clinics.

Haematology assessments: Haemoglobin, red blood cells (RBC), platelets, mean cell volume (MCV), mean cell haemoglobin concentration (MCHC), mean cell haemoglobin (MCH), white blood cells (WBC), absolute differential white blood count (neutrophils, lymphocytes, monocytes, eosinophils, basophils) and absolute neutrophil count.

Clinical chemistry: Albumin, AST, ALT, ALP, bilirubin (total), bicarbonate, calcium (total), cholesterol, GGT, creatinine, magnesium, sodium, urea nitrogen, glucose, uric acid, LDH, phosphate, chloride, amylase, lipase, total protein, triglycerides and potassium.

Tests for aspartate ALT, AST, ALP, and total bilirubin must be conducted concurrently and assessed concurrently.

All patients with an AST, ALT or bilirubin value (the latter ≥1.5 x ULN) at the time of the last dose of durvalumab should have a further liver chemistry profile (AST, ALT, bilirubin and ALP) performed 28 days (± 7 days) after permanent discontinuation of durvalumab.

Results for urea and electrolytes, full blood count and liver function tests must be available before commencing an infusion. These can be done within 2 days of infusion, with the exception of Cycle 1 Day 1.

i Urinalysis to be performed at screening, day 1 of every cycle, day 8 of cycle 1, at discontinuation and at the 28 day follow up visit: Blood, protein, glucose, ketones, bilirubin, pH, colour & appearance and specific gravity.

Microscopy should be used as appropriate to investigate white blood cells and use the high power field for red blood cells.

j Thyroid functions tests (T3, T4 and TSH), and a cortisol level test to be performed at screening, day 1 of every cycle, day 8 of cycle 1, at discontinuation and at the 28 day follow up visit.

k Female patients of child-bearing potential only. A serum or urine pregnancy test is to be performed at screening, pre dose on day 1 of each cycle and at discontinuation.

l CT or MRI scan of head, chest and abdomen to be performed at screening. CT or MRI scans of chest and abdomen to be performed until discontinuation. Following screening, the first tumour assessment should be performed 8 weeks after cycle 1 day 1, then every 8 weeks thereafter for the first year, later reducing to every 12 weeks. Scans should be performed ± 7 days (except 1st scan + 7 days only). If brain metastases are identified at screening or if clinically indicated, head scanning should also be performed throughout treatment at the same time points. **The same imaging modality must be used consistently throughout the course of the trial for each patient.**

Tumour assessments will be performed in follow up for patients who discontinue treatment for reasons other than Progressive Disease (e.g. toxicity). These scans should continue to be performed on a 8-weekly basis for the first year, then every 12 weeks until disease progression or the patient starts a new anti-cancer therapy (unless the patient withdraws consent to do so). Scans should be of the chest and abdomen,

and only include the head where brain metastases are identified at screening, or if clinically indicated. All scans to be reported using RECIST 1.1.

m Cycle 1 (day 1): Treatment must commence within 7 days of trial registration. AZD6738 and durvalumab must be dispensed within the IWRS Cenduit system. Refer to the Pharmacy Manual for further details. AZD6738 may be dispensed within the IWRS up to 2 days prior to the actual visit date and durvalumab may be dispensed within the IWRS up to 3 days prior to the actual visit date.

n Smoking status data will be collected through questions and CO monitoring **pre-dose** cycle 1 day 1 then every 8 weeks (or day 1 of every other cycle) and at discontinuation.

o A whole blood germline DNA sample is to be collected pre-dose on cycle 1 day 1. The sample can be taken up to 2 days earlier than the actual visit date (commencement of cycle). If the sample is not collected at this time point, it should be collected at the next visit. Refer to the Laboratory Manual for sample processing guidelines.

p ctDNA samples to be collected **pre-dose** cycle 1 day 1 then every 8 weeks (or day 1 of every other cycle) and at discontinuation. Refer to the Laboratory Manual for sample processing instructions. ctDNA samples should be taken **prior** to durvalumab infusion on treatment visits. Samples can be taken up to 2 days earlier than the actual visit date (where indicated). After 12 months of treatment, ctDNA sample collection may be reduced to 12 weekly in line with adjusted visit timing (see footnote ‘u’).

$ ctDNA samples will be collected in follow up for patients who discontinue treatment for reasons other than Progressive Disease (e.g. toxicity). These samples should be performed at the same visit as follow up CT or MRI scans until disease progression or the patient starts a new anti-cancer therapy (unless the patient withdraws consent to do so). Samples should continue to be collected on an 8-weekly basis for the first year relative to the start date of treatment, then every 12 weeks.

q An optional fresh metastatic/recurrent tumour biopsy sample should be collected (if patient consents) post-registration, pre-treatment and at the end of treatment visit for patients who discontinue treatment for reasons other than disease progression (origin from either the primary tumour or site of metastasis). An optional pre-treatment biopsy should not be performed in cases where the patient has already had a mandatory biopsy for molecular testing (Note - a mandatory repeat SMP2 biopsy will be performed if the patient has had targeted therapy e.g. ALK inhibitor). The discontinuation biopsy must be performed prior to commencing further anti-cancer therapy. A post-treatment biopsy will only be requested from patients with an objective response or stabilisation of disease (PR or CR), or 6 months on treatment with evidence of stabilisation (SD) for patients who have previously progressed. The tumour tissue will be used to determine possible mechanisms of resistance to trial treatment. Refer to the Laboratory Manual for sample processing instructions.

r Survival status will be collected every 12 weeks (± 7 days) post-permanent discontinuation of AZD6738 and durvalumab until death.

s 28 day follow up visit should be carried out 28 days (+ 7 days) post-permanent discontinuation of AZD6738 and durvalumab.

t There is no need to test for Hepatitis B, Hepatitis C or HIV unless the investigator is concerned they may test positive for any of these infections.

u Once a patient has completed 12 months of treatment (approximately 13 cycles), visits may be reduced to 12 weekly at the discretion of the Investigator.

30.3.3 Toxicity Profile

The toxicology and safety pharmacology is fully described in the current version of the AZD6738 and durvalumab Investigator's Brochures (IB).

30.3.3.1 Safety Issues

Toxicity with AZD6738 is primarily bone-marrow related. The emerging safety profile is detailed in the Investigator Brochure and will constitute as the Reference Safety Information for AZD6738. Durvalumab safety issues and Adverse Events of Special Interest are detailed below and in the most recent Investigator Brochure.

30.3.3.2 Expected Adverse Events

30.3.3.2.1 AZD6738

As of the data cut-off of 13 June 2018, the most commonly observed AEs with AZD6738 in combination with durvalumab, irrespective of causality, have been thrombocytopenia (20.6% of AEs), anaemia (44.1% AEs), neutropenia (14.7% of AEs), nausea, vomiting, constipation, diarrhoea, decreased appetite, hypokalaemia, fatigue, cough, dizziness and rash.

30.3.3.2.2 Durvalumab

As of the data cut-off of 12 July 2019, the most commonly observed AEs with durvalumab monotherapy (≥15%), irrespective of causality, have been fatigue, decreased appetite, cough, nausea, dyspnoea, constipation and diarrhoea. AEs considered to be related to durvalumab in ≥5% of patients were fatigue, diarrhoea, hypothyroidism, pruritus, nausea, decreased appetite and rash. A total of 44.5% of patients reported AEs of CTCAE Grade 3 or higher: Grade 3, 4, and 5 (fatal) events were reported in 24.5%, 4.7% and 5.2% of patients respectively. Grade 3, 4 and 5 events considered related to durvalumab were reported in 9.9%, 1.0% and 0.6% respectively.

Grade 3 or 4 events occurring in ≥2% of patients were anaemia, dyspnoea, hyponatraemia, fatigue, pneumonia and gamma-glutamyltransferase (GGT) increase. Grade 3 or 4 events considered related to durvalumab occurring in ≥0.5% of patients were fatigue, GGT increase, pneumonitis and aspartate aminotransferase (AST) increase. The most common Grade 5 events were general physical health deterioration (0.5% of patients), respiratory failure, pneumonia and sepsis (0.4% of patients each). The only Grade 5 events considered related to durvalumab occurring in ≥2 patients were pneumonitis and respiratory failure.

Adverse Events of Special Interest

Adverse Events of Special Interest (AESIs) are events of scientific and medical interest specific to the further understanding of the durvalumab safety profile and require close monitoring and rapid communication by the local Investigator to the Trial Office. Durvalumab AESIs may be serious or non-serious. The rapid reporting of these AESIs allows ongoing analysis of these events in order to characterise and understand them in association with the use of this investigational product.

AESIs reported in AstraZeneca or MedImmune-sponsored durvalumab studies and the Investigator Brochure are defined as AEs that include, but are not limited to, events with a potential inflammatory or immune-mediated mechanism that may require more frequent monitoring and/or interventions such as corticosteroids, immunosuppressants, and/or hormone replacement therapy.

The AESIs are grouped under the following categories:

- Dermatitis/rash
- Diarrhoea/colitis
- Hepatic events
- Hypothyroid & hyperthyroid events
- Thyroiditis
- Adrenal insufficiency
- Hypophysitis/hypopituitarism
- Type I diabetes mellitus
- Renal events
- Pneumonitis
- Pancreatic events
- Other rare/miscellaneous events
- Intestinal perforations
- Myocarditis
- Myositis
- Guillain-Barre syndrome
- Myasthenia gravis
- Infusion/hypersensitivity reactions

Immune-Mediated Adverse Events (imAEs)

An immune-mediated adverse event (imAE) is defined as an adverse event that is associated with drug exposure and is consistent with an immune-mediated mechanism of action and where there is no clear alternate aetiology.

In AZ studies where the data is clean and validated, an adjudication process has been conducted to identify imAEs. These are AESIs (excluding infusion related/hypersensitivity/anaphylactic reaction) consistent with an immune-mediated mechanism that required treatment with systemic corticosteroids, other immunosuppressants, or endocrine therapy.

Events with an inflammatory or immune mediated mechanism could occur in nearly all organs. Potential risks with an immune-mediated aetiology that are rare or less frequent include, but are not limited to, Guillain-Barre Syndrome, myasthenia gravis, pericarditis, sarcoidosis, uveitis, and other events involving the eye (eg, keratitis and optic neuritis), skin (eg, scleroderma, vitiligo and pemphigoid), haematological (eg, haemolytic anaemia and immune thrombocytopenic purpura), rheumatological events (polymyalgia rheumatic and autoimmune arthritis), vasculitis, non-infectious meningitis and non-infectious encephalitis.

AESIs and imAEs are manageable by available/established treatment guidelines as described in the Toxicity Management section (30.3.4). In the durvalumab clinical programme the safety profile has been consistent across tumour types. AEs reported with durvalumab monotherapy and durvalumab and tremelimumab combination therapy for the pooled datasets, key clinical studies and safety data from completed studies are summarised in the most recent Investigator Brochure. For the agents that have been used in combination with durvalumab, safety data for these agents when used as monotherapies, can be found in their respective IBs.

30.3.4 Dose Modifications & Toxicity Management

The following general guidance should be followed for management of toxicities:

- Treat each of the toxicities with maximum supportive care, including interruption of the agent suspected of causing the toxicity, if required.
- If an AE is clearly attributed to one drug, the other drug may continue without interruption until the criteria for recommencement for the interrupted drug is met (see Section 30.3.4.7 Criteria for Recommencement of Treatment).
- If the symptoms promptly resolve (CTCAE grade 1 or 2 (depending on toxicity) or to the baseline CTCAE grade) with supportive care, consideration should be given to continuing the same dose of AZD6738 with appropriate continuing supportive care.
- If the toxicity does not resolve to ≤ CTCAEv4 grade 1 or 2 (depending on the toxicity) or the patient is not showing clinical benefit, then the patient should be discontinued from treatment and observed until resolution of the toxicity.
- If medically appropriate, dose modifications are permitted of AZD6738 only (see Section 30.3.4.3). Dose reductions of durvalumab are not permitted, however dose modifications are permitted (see Table 84 to Table 88).
- Dose modifications will not be required for AEs that are clearly not attributed to durvalumab or AZD6738 (such as an accident) or for laboratory abnormalities that are not deemed to be clinically significant.
- All dose modifications should be documented clearly in the CRF.
- All toxicities will be graded according to NCI CTCAE, Version 4.03.

30.3.4.1 AZD6738 haematological toxicity

Complete blood counts will be obtained for all patients at the beginning of each treatment cycle (Day 1).

- If ANC >1 x10^{9}/L and platelets are ≥75 x10^{9}/L no dose modification or interruption is required.
- If ANC <1 x10^{9}/L and/or platelets are <75 x10^{9}/L treatment should be delayed by 1 week intervals until recovery whilst ANC and platelets are monitored weekly (more often as clinically indicated) until recovery.

Any patients with Grade 3 or 4 hematologic toxicity should be identified in cycles 1 and 2. For cycle 3 onwards, recovery of any cytopenias (platelets ≥100x10^{9}/L and neutrophils ≥1.5x10^{9}/L) should be ensured prior to administration of AZD6738 on day 15.

See Section 30.3.4.4 for advice on prolonged haematological toxicities and Section 30.3.4.7 for criteria for recommencement of trial treatment. If haematological parameters do not recover within 28 days, permanent trial discontinuation is strongly recommended and should be discussed with the Trials Office and Lead Investigator.

Patients already receiving erythropoietin at registration may continue, providing they have been receiving it for more than one month. Blood and platelet transfusions are permitted during the trial but are subject to a 28 day washout period before trial registration (see Section 30.2 Specific Eligibility Critieria). Supportive care should be clearly documented as a concomitant medication on the relevant CRF.

30.3.4.1.1 Anaemia

Common treatable causes of anaemia (eg, iron, vitamin B12 or folate deficiencies and hypothyroidism) should be investigated and appropriately managed. In some cases management of anaemia may require blood transfusions.

- For Grade 2 (Hb <10 but ≥8 g/dL), give appropriate supportive treatment and investigate causality. Investigator judgement to continue trial treatment with supportive treatment (e.g. transfusion) or interrupt dose for a maximum of 4 weeks until Hb recovery to ≥10 g/dL.
- For Grade 3 (Hb <8 g/dL) trial treatment should be interrupted for a maximum of 4 weeks until improved to Hb ≥8 g/dL. Upon recovery AZD6738 dose should be reduced (see Section 30.3.4.3).
- For cases where patients develop prolonged haematological toxicity (≥2 week interruption/delay in trial treatment due to CTCAE grade 3 or worse anaemia and/or development of blood transfusion dependence) dose reduce as per Section 30.3.4.3).

30.3.4.1.2 Neutropenia, leukopenia and thrombocytopenia

Adverse events of neutropenia and leukopenia should be managed as deemed appropriate by the Investigator.

- Symptoms should be closely monitored and interruption of trial drug if CTC grade 3 or worse neutropenia occurs for a maximum of 4 weeks until resolved to grade 1 or 2 or better. Appropriate supportive treatment and causality assessment should be carried out.
- If repeat CTCAE grade 3-4 occurrence, dose reduce.
- For toxicity of grades 1-2 especially if more than one toxicity is present then drug can be interrupted at Investigator discretion for a maximum of 4 weeks whilst appropriate supportive treatment is given and causality investigation is carried out.
- For grade 1 neutropenia and /or thrombocytopenia, AZD6738 may continue if neutrophil count is ≥1500/mm^3 and/or platelet count is ≥75,000/mm^3.
- For Grade 2 neutropenia, trial treatment should be interrupted for a maximum of 4 weeks until improved to at least neutrophil count ≥1500/mm^3. Upon recovery AZD6738 dose should be reduced (see Section 30.3.4.3).
- For grade 2-3 thrombocytopenia trial treatment should be interrupted for a maximum of 4 weeks until platelets improve to at least ≥100,000/mm^3. At resolution it is not mandatory to lower the dose as blood counts may recover during the “off period” on the intermittent schedule. If blood counts do not recover by the start of the next dosing period, AZD6738 should be restarted with a dose reduction.
- For Grade 4 thrombocytopenia trial treatment should be interrupted for a maximum of 4 weeks until the platelet count has recovered to ≥100,000/mm^3.
- Colony-stimulating factors including G-CSF, pegylated G-CSF or GM-CSF for neutropenia may be considered after the first cycle of chemotherapy following discussion with the Trials Office and Lead Investigator. Whilst primary prophylaxis with G-CSF is not recommended, if a patient develops febrile neutropenia, it will be advised that trial treatment is stopped and appropriate management, including G-CSF may be given according to local hospital guidelines. G-CSF should not be given within 24 hours (7 days for pegylated G-CSF) of the last dose of trial treatment unless absolutely necessary.

30.3.4.2 AZD6738 non-haematological toxicity

Acute toxicities should be managed as medically indicated, with temporary suspension of trial drug and initiation of supportive care as clinically indicated by the treating clinician.

- Treatment must be interrupted if any CTCAE Grade 3 or 4 non-haematologic AE occurs which the Investigator considers to be related to the administration of the trial treatment(s).
- Treatment should not be restarted until the toxicity reverts to CTCAE grade 1 or 2 (depending on toxicity) or to the baseline CTCAE grade.
  If the required interruption is any longer than 28 days due to toxicity, permanent trial discontinuation is strongly recommended and should be discussed with the Trials Office and Lead Investigator.

30.3.4.2.1 New or worsening pulmonary symptoms

If new or worsening pulmonary symptoms or radiological abnormality suggestive of pneumonitis/ILD is observed, the following assessments (and additional assessments if required) will be performed:

- Physical examination; Signs and symptoms (cough, hypoxia, dyspnoae and pyrexia etc) including auscultation for lung field will be assessed.
- High resolution CT scan looking for interstitial infiltrates in whom infectious, neoplastic or other causes have been excluded.
- SpO2; Saturation of peripheral oxygen (SpO2).
- When pneumonitis (interstitial lung disease [ILD]) is suspected during trial treatment, the folowing markers should be measured where possible:
    - ILD markers (KL-6, SP-D) and β-D-glucan.
- See Table 30: ILD/Pneumonitis Management Grades 1-4 & Table 31: ILD/Pneumonitis Management Following Improvement to Baseline (Arm C Section 24.3.4) for more detailed guidance on the management of suspected ILD/pneumonitis, including investigations and assessment, monitoring, supportive care and action with trial IMP.

30.3.4.2.2 Nausea and vomiting

Nausea and vomiting should be treated aggressively and strong considerations should be given to the administration of prophylactic anti-emetic therapy as per local guidelines. As per international guidance on anti-emetic use in cancer patients (ESMO, NCCN), generally a single agent antiemetic should be considered eg, dopamine receptor antagonist, antihistamines or dexamethasone. Aprepitant (Emend) should not be used (for further details see Restrictions & Concomitant Medications - Section 30.2.3.1).

Patients should be strongly encouraged to maintain liberal oral fluid intake. If vomiting occurs shortly after AZD6738 is swallowed, the dose should only be replaced if all of the intact tablets can be counted. Resume with the following scheduled dose.

30.3.4.2.3 Diarrhoea

Prompt anti-diarrhoeal treatment is required at the first onset of diarrhoea. It is advised that oral loperamide (Imodium) 4 mg should be administered at the first onset of diarrhoea and

then 2 mg every 2 hours until diarrhoea-free for at least 12 hours. The first dose of loperamide could be lowered to 2 mg if the diarrhoea is recurrent and if, in the opinion of the treating physician, the diarrhoea is not severe. Anti-diarrhoeal agents should not be given prophylactically.

Patients should be instructed to begin taking anti-diarrhoeal medication at the first sign of: 1) poorly formed or loose stool, 2) occurrence of more bowel movements than usual in one day or 3) unusually high volume of stool. Anti-diarrhoeal agents should be deferred if blood or mucus is present in the stool or if diarrhoea is accompanied by fever. In this setting, appropriate diagnostic microbiologic specimens should be obtained to exclude an infectious aetiology.

Patients should also be advised to drink liberal quantities of clear fluids to help prevent dehydration. Patients should be instructed to notify the Investigator or research staff of the occurrence of bloody or black stools, symptoms of dehydration, fever, inability to take liquids by mouth, and inability to control diarrhoea within 24 hours of using loperamide or other prescribed anti diarrhoeal medications.

If diarrhoea is severe (ie, requiring intravenous [IV] rehydration) and/or associated with fever or severe neutropenia (Grade 3 or 4), broad-spectrum antibiotics must be prescribed. Patients with severe diarrhoea or any diarrhoea associated with severe nausea or vomiting should be hospitalised for IV hydration and correction of electrolyte imbalances.

30.3.4.2.4 Renal toxicity

If subsequent to trial entry and while still on trial therapy, a patient's estimated CrCl falls below ≥45 mL/min, retesting should be performed promptly.

A one-step dose reduction is recommended for patients who develop moderate renal impairment (calculated CrCl by Cockcroft-Gault equation of between 31 and 45 mL/min) for any reason during the course of the trial (see Section 30.3.4.3 AZD6738 dose modification/reduction recommendations).

30.3.4.2.5 Hepatic toxicity

Drug should be stopped if:

- ALT or AST or ALP* > 5 × ULN
- ALT or AST or ALP* > 3 × ULN with the appearance of symptoms associated with a clinical diagnosis of hepatitis including right upper quadrant pain or tenderness, fever, rash or eosinophilia (>5%)
- ALT or AST > 3 × ULN and total bilirubin > 2 × ULN or INR⁺ >1.5 (unless patient is receiving warfarin) or other evidence of impairment to the synthesis function of the liver]

* In the presence of bone metastasis, assess bone specific isoform of raised ALP in the presence of a raised gamma-GT (to ensure the ALP change is specific to the liver).

⁺ Unless patient is receiving warfarin

Cases where a subject shows elevations in liver biochemistry may require further evaluation, and occurrences of AST or ALT ≥3 x ULN together with total bilirubin ≥2 x ULN may need to be reported as an SAE. Please refer to Appendix 12: Actions required in case of increases in liver biochemistry and evaluation of Hy's Law.

30.3.4.2.6 Cardiovascular abnormalities

Cardiovascular toxicity is potentially associated with AZD6738. Either clinically significant hypotension (defined as an asymptomatic decrease of more than 20 mmHg in systolic blood pressure to below 70 mmHg persisting for at least 10 minutes) or symptomatic orthostatic fall in systolic blood pressure (of more than 20 mmHg compared to resting supine systolic blood pressure) should be monitored for and managed with drug interruptions or reductions as per Investigator's discretion. Other cardiovascular safety measures e.g. ECG readings should also be considered.

30.3.4.3 AZD6738 dose modification/reduction recommendations

At the first occurrence of CTCAE grade 3 and 4 haematological toxicity AZD6738 should be held until resolution of toxicity to CTCAE grade 1 or 2 (depending on parameter affected) or baseline. At the resolution of the first occurrence of a toxicity, a change to dose is not automatically required unless indicated by the specific nature of the AE, the severity or recurrence. At the second occurrence, upon resolution of the toxicity, it is suggested that AZD6738 is reduced by the first dose reduction as indicated below in Table 83.

A second dose reduction for toxicity may also be considered as outlined below, however, no more than two dose reductions are required, and if a third dose reduction is indicated the patient should be discontinued from trial treatment.

Once the dose is reduced, escalation is not permitted, even if toxicities have resolved. All dose modification and interruptions (including any missed doses) and the reasons for the modifications/interruptions are to be recorded on the CRF.

Table 83: AZD6738 dose modifications for toxicity management

Dose level	AZD6738
Initial dose	240 mg BD Days 15-28
1st dose reduction for haematological toxicity	160 mg BD Days 15-28
1st dose reduction for non-haematological toxicity	160 mg BD Days 15-28
2nd dose reduction	80 mg BD Days 15-28
3rd dose reduction	No further dose reduction, withdraw patient and treat as clinically indicated.

30.3.4.4 Prolonged haematological toxicities while on trial treatment

If a va such as:

- ≥2 week interruption/delay in trial treatment due to CTC grade 3 or worse anemia and/or development of blood transfusion dependence
- ≥2 week interruption/delay in trial treatment due to CTC grade 3 or worse neutropenia (ANC < 1 x 10^9/L).
- ≥2 week interruption/delay in trial treatment due to CTC grade 3 or worse thrombocytopenia and/or development of platelet transfusion dependence (Platelets < 50 x 10^9/L).

Check weekly differential blood counts including reticulocytes and peripheral blood smear. If any blood parameters remain clinically abnormal after 4 weeks of dose interruption, the patient should be referred to a haematologist for further investigations. Bone marrow analysis and/or blood cytogenetic analysis should be considered at this stage according to standard haematological practice. It is strongly recommended that trial treatment is discontinued if blood counts do not recover to CTC grade 1 or better within 4 weeks of dose interruption.

Development of a confirmed MDS or other clonal blood disorder should be reported as an SAE. Trial treatment should be discontinued if patient’s diagnosis of MDS and/or AML is confirmed. The effect if any of AZD6738 on the bone marrow in terms of prolonged suppression is unknown.

30.3.4.5 Durvalumab immune-mediated and infusion-related reactions

Comprehensive toxicity management guidelines for immune-mediated reactions, infusion-related reactions, and non-immune-mediated reactions for durvalumab are provided in Table 84 below. These guidelines are to assist Investigators in their clinical judgment in treating these types of toxicities and apply to AEs considered causally related to durvalumab regimen by the reporting Investigator.

Based on the mechanism of action of durvalumab leading to T-cell activation and proliferation, there is the possibility of observing immune-related reactions (imAEs) during the conduct of this trial. Potential imAEs may be similar to those seen with the use of ipilimumab including immune mediated enterocolitis, dermatitis, hepatitis, and endocrinopathies (Brahmer et al. 2010, Hodi et al. 2010).

Patients should be monitored for signs and symptoms of irAEs. In the absence of an alternate aetiology (e.g., infection or PD) signs or symptoms of enterocolitis, dermatitis, hepatitis, and endocrinopathy should be considered to be immune-related.

Patients should be thoroughly evaluated and appropriate efforts should be made to rule out neoplastic, infectious, metabolic, toxin, or other etiologic causes of the AE. Serologic, immunologic, and histologic (biopsy) data, as appropriate, should be used to support an AE diagnosis. In the absence of a clear alternative etiology, events should be considered potentially immune related.

For signs and symptoms suggestive of keratitis or uveitis (such as acute or worsening eye inflammation, lacrimation, light sensitivity, blurred vision, eye pain and /or red eye), subjects should be advised to seek medical advice promptly, and, if required, referred promptly to an ophthalmologist. Adverse events related to the eye may be immune related and should be managed according to local practice.

If the local Investigator has any questions in regards to an adverse event being an imAE, it should be promptly discussed with the Trial Office and Lead Investigator.

Table 84: General Considerations Regarding Immune-Mediated Reactions

Dose Modifications	Toxicity Management
Drug administration modifications of study drug/study regimen will be made to manage potential immune-related AEs based on severity of treatment-emergent toxicities graded per NCI CTCAE v4.03 (unless indicated otherwise).<br>In addition to the criteria for permanent discontinuation of study drug/study regimen based on CTC grade/severity (table below), permanently discontinue study drug/study regimen for the following conditions:<br>• Inability to reduce corticosteroid to a dose of ≤10 mg of prednisone per day (or equivalent) **within 12 weeks** of the start of the immune-mediated adverse event (imAE)<br>• Grade 3 recurrence of a previously experienced treatment-related imAE following resumption of dosing<br>**Grade 1** No dose modification<br>**Grade 2** Hold study drug/study regimen dose until Grade 2 resolution to Grade ≤1.<br>If toxicity worsens, then treat as Grade 3 or Grade 4.<br>Study drug/study regimen can be resumed once event stabilizes to Grade ≤1 after completion of steroid taper.<br>Patients with endocrinopathies who may require prolonged or continued steroid replacement can be retreated with study drug/study regimen on the following conditions:<br>1. The event stabilizes and is controlled.<br>2. The patient is clinically stable as per Investigator or treating physician's clinical judgement.<br>3. Doses of prednisone are at ≤10 mg/day or equivalent.	It is recommended that management of immune-mediated adverse events (imAEs) follows the guidelines presented in this table:<br>– It is possible that events with an inflammatory or immune mediated mechanism could occur in nearly all organs, some of them not noted specifically in these guidelines.<br>– Whether specific immune-mediated events (and/or laboratory indicators of such events) are noted in these guidelines or not, patients should be thoroughly evaluated to rule out any alternative etiology (e.g., disease progression, concomitant medications, and infections) to a possible immune-mediated event. In the absence of a clear alternative etiology, all such events should be managed as if they were immune related. General recommendations follow.<br>– Symptomatic and topical therapy should be considered for low-grade (Grade 1 or 2, unless otherwise specified) events.<br>– For persistent (>3 to 5 days) low-grade (Grade 2) or severe (Grade ≥3) events, promptly start prednisone 1 to 2 mg/kg/day PO or IV equivalent.<br>– Some events with high likelihood for morbidity and/or mortality – e.g., myocarditis, or other similar events even if they are not currently noted in the guidelines – should progress rapidly to high dose IV corticosteroids (methylprednisolone at 2 to 4 mg/kg/day) even if the event is Grade 2, and if clinical suspicion is high and/or there has been clinical confirmation. Consider, as necessary, discussing with the study physician, and promptly pursue specialist consultation.<br>– If symptoms recur or worsen during corticosteroid tapering (28 days of taper), increase the corticosteroid dose (prednisone dose [e.g., up to 2 to 4 mg/kg/day PO or IV equivalent]) until stabilization or improvement of symptoms, then resume corticosteroid tapering at a slower rate (>28 days of taper).

Dose Modifications	Toxicity Management
**Grade 3** Depending on the individual toxicity, study drug/study regimen may be permanently discontinued. Please refer to guidelines below.<br>**Grade 4** Permanently discontinue study drug/study regimen.<br>Note: For asymptomatic amylase or lipase levels of >2.0×ULN, hold study drug/study regimen, and if complete work up shows no evidence of pancreatitis, study drug/study regimen may be continued or resumed.<br>Note: Study drug/study regimen should be permanently discontinued in Grade 3 events with high likelihood for morbidity and/or mortality – e.g., myocarditis, or other similar events even if they are not currently noted in the guidelines. Similarly, consider whether study drug/study regimen should be permanently discontinued in Grade 2 events with high likelihood for morbidity and/or mortality – e.g., myocarditis, or other similar events even if they are not currently noted in the guidelines – when they do not rapidly improve to Grade <1 upon treatment with systemic steroids and following full taper<br>Note: There are some exceptions to permanent discontinuation of study drug for Grade 4 events (i.e., hyperthyroidism, hypothyroidism, Type 1 diabetes mellitus).	– More potent immunosuppressives such as TNF inhibitors (e.g., infliximab; also refer to the individual sections of the imAEs for specific type of immunosuppressive) should be considered for events not responding to systemic steroids. Progression to use of more potent immunosuppressives should proceed more rapidly in events with high likelihood for morbidity and/or mortality – e.g., myocarditis, or other similar events even if they are not currently noted in the guidelines – when these events are not responding to systemic steroids.<br>– With long-term steroid and other immunosuppressive use, consider need for *Pneumocystis jirovecii* pneumonia (PJP, formerly known as *Pneumocystis carinii* pneumonia) prophylaxis, gastrointestinal protection, and glucose monitoring.<br>Discontinuation of study drug/study regimen is not mandated for Grade 3/Grade 4 inflammatory reactions attributed to local tumor response (e.g., inflammatory reaction at sites of metastatic disease and lymph nodes). Continuation of study drug/study regimen in this situation should be based upon a benefit-risk analysis for that patient.

AE Adverse event; CTC Common Toxicity Criteria; CTCAE Common Terminology Criteria for Adverse Events; imAE immune-mediated adverse event; IV intravenous; NCI National Cancer Institute; PO By mouth.

Table 85: Durvalumab Specific Immune-Mediated Reactions

Adverse Events	Severity Grade of the Event	Dose Modifications	Toxicity Management
**Pneumonitis/Interstitial Lung Disease (ILD)**	**Any Grade**<br>(Refer to NCI CTCAE applicable version in study protocol for defining the CTC grade/severity)	**General Guidance**	**For Any Grade:**<br>– Monitor patients for signs and symptoms of pneumonitis or ILD (new onset or worsening shortness of breath or cough). Patients should be evaluated with imaging and pulmonary function tests, including other diagnostic procedures as described below.<br>– Suspected pneumonitis should be confirmed with radiographic and other infectious and disease-relate aetiologies, excluded and managed as described below.<br>– Initial work-up may include clinical evaluation, monitoring of oxygenation via pulse oximetry (resting and exertion), laboratory work-up, and high- resolution CT scan.<br>– Consider Pulmonary and Infectious Diseases consults
	**Grade 1**	No dose modifications required. However, consider holding study drug/study regimen dose as clinically appropriate and during diagnostic work-up for other etiologies.	**For Grade 1 :**<br>– Monitor and closely follow up in 2 to 4 days for clinical symptoms, pulse oximetry (resting and exertion), and laboratory work-up and then as clinically indicated.<br>–
	**Grade 2**	Hold study drug/study regimen dose until Grade 2 resolution to Grade ≤1.<br>• If toxicity worsens, then treat as Grade 3 or Grade 4.<br>• If toxicity improves to Grade ≤1, then the decision to reinitiate study drug/study regimen will be based upon treating physician's clinical	**For Grade 2**<br>– Monitor symptoms daily and consider hospitalization.<br>– Promptly start systemic steroids (e.g., prednisone 1 to 2 mg/kg/day PO or IV equivalent).<br>– Reimage as clinically indicated, consider chest CT with contrast and repeat in 3-4 weeks<br>– If no improvement within 2 to 3 days, additional workup should be considered and prompt treatment with IV methylprednisolone 2 to 4 mg/kg/day started<br>– If no improvement within 2 to 3 days despite IV methylprednisolone at 2 to 4 mg/kg/day, promptly start immunosuppressive therapy such

Adverse Events	Severity Grade of the Event	Dose Modifications	Toxicity Management
		judgment and after completion of steroid taper.	as TNF inhibitors (e.g., infliximab at 5 mg/kg IV once, may be repeated at 2 and 6 weeks after initial dose at the discretion of the treating provider). Caution: It is important to rule out sepsis and refer to infliximab label for general guidance before using infliximab.<br>– Consider, as necessary, discussing with study physician.
	**Grade 3 or 4**	Permanently discontinue study drug/study regimen.	**For Grade 3 or 4:**<br>– Promptly initiate empiric IV methylprednisolone 1 to 4 mg/kg/day or equivalent.<br>– Obtain Pulmonary and Infectious Diseases Consults; consider, discussing with study physician as needed.<br>– Hospitalize the patient.<br>– Supportive care (e.g., oxygen).<br>– If no improvement within 2 to 3 days, additional workup should be considered and prompt treatment with additional immunosuppressive therapy such as TNF inhibitors (e.g., infliximab at 5 mg/kg IV, may be repeated at 2 and 6 weeks after initial dose at the discretion of the treating provider . Caution: rule out sepsis and refer to infliximab label for general guidance before using infliximab.
**Diarrhoea/Colitis**	**Any Grade**	**General Guidance**	**For Any Grade:**<br>– Monitor for symptoms that may be related to diarrhoea/enterocolitis (abdominal pain, cramping, or changes in bowel habits such as increased frequency over baseline or blood in stool) or related to bowel perforation (such as sepsis, peritoneal signs, and ileus).<br>– WHEN SYMPTOMS OR EVALUATION INDICATE A PERFORATION IS SUSPECTED, CONSULT A SURGEON EXPERIENCED IN ABDOMINAL SURGERY IMMEDIATELY WITHOUT ANY DELAY.<br>PERMANENTLY DISCONTINUE STUDY DRUG FOR ANY GRADE OF INTESTINAL PERFORATION.<br>– Patients should be thoroughly evaluated to rule out any alternative etiology (e.g., disease progression, other medications, or infections), including testing for clostridium difficile toxin, etc.

Adverse Events	Severity Grade of the Event	Dose Modifications	Toxicity Management
			– Steroids should be considered in the absence of clear alternative etiology, even for low-grade events, in order to prevent potential progression to higher grade event, including intestinal perforation.<br>– Use analgesics carefully; they can mask symptoms of perforation and peritonitis.
	**Grade 1**	No dose modifications.	**For Grade 1:**<br>– Monitor closely for worsening symptoms.<br>– Consider symptomatic treatment, including hydration, electrolyte replacement, dietary changes (e.g., American Dietetic Association colitis diet), loperamide, and other supportive care measures. Use probiotics as per treating physician's clinical judgment.<br>– If symptoms persist, consider checking lactoferrin; if positive treat as Grade 2 below. If negative and no infection, continue Grade 1 management,
	**Grade 2**<br><br>* "medical intervention" is not invasive	Hold study drug/study regimen until resolution to Grade ≤1<br>• If toxicity worsens, then treat as Grade 3 or Grade 4.<br>• If toxicity improves to Grade ≤1, then study drug/study regimen can be resumed after completion of steroid taper.	**For Grade 2:**<br>– Consider symptomatic treatment, including hydration, electrolyte replacement, dietary changes (e.g., American Dietetic Association colitis diet), and loperamide and/or budesonide.<br>– Promptly start prednisone 1 to 2 mg/kg/day PO or IV equivalent.<br>– If event is not responsive within 2 to 3 days or worsens despite prednisone at 1 to 2 mg/kg/day PO or IV equivalent, consult a GI specialist for consideration of further workup, such as imaging and/or colonoscopy, to confirm colitis and rule out perforation.<br>– If still no improvement within 2 to 3 days despite 1 to 2 mg/kg IV methylprednisolone, promptly start immunosuppressant agentssuch as infliximab at 5 mg/kg IV, may be repeated at 2 and 6 weeks after initial dose at the discretion of the treatment provider. **Caution:** it is important to rule out bowel perforation and refer to infliximab label for general guidance before using infliximab.<br>– Consider, as necessary, discussing with study physician if no resolution to Grade ≤1 in 3 to 4 days.

Adverse Events	Severity Grade of the Event	Dose Modifications	Toxicity Management
	**Grade 3 or 4**<br><br>*This guidance anticipates that Grade 3 operative interventions of perforations are usually not elective	Grade 3<br><br>• For patient treated with PDL-1 inhibitors hold study drug/study regimen until resolution to Grade ≤1; study drug/study regimen can be resumed after completion of steroid taper. Permanently discontinue study drug/study regimen for Grade 3 toxicity does not improve to rade ≤1 within 14 days.<br>• Permanently discontinue study drug for 1) Grade 3 colitis in patients treated with CTLA-4 inhibitors or 2) Any grade of intestinal performtion in any patient treated with ICI<br><br>Grade 4<br>Permanently discontinue study drug/study regimen.	**For Grade 3 or 4:**<br>– Promptly initiate empiric IV methylprednisolone 1 to 2 mg/kg/day or equivalent.<br>– Monitor stool frequency and volume and maintain hydration.<br>– Urgent GI consult and imaging and/or colonoscopy as appropriate.<br>– If still no improvement within 2 to 3 days, promptly add further immunosuppressants (e.g., infliximab at 5 mg/kg IV, may be repeated at 2 and 6 weeks after initial dose at the discretion of the treating provide. **Caution**: Ensure GI consult to rule out bowel perforation and refer to infliximab label for general guidance before using infliximab. If perforation is suspected, consult a surgeon experienced in abdominal surgery immediately without any delay.<br>– If perforation is suspected, consilt a surgeon experienced in abdominal surgery without any delay.

Adverse Events	Severity Grade of the Event	Dose Modifications	Toxicity Management
**Hepatitis (elevated LFTs)**<br>Infliximab should not be used for management of immune-related<br>**PLEASE SEE shaded area immediately below this section to find guidance for management of "Hepatitis (elevated LFTS)" in HCC patients**<br>hepatitis.	**Any Grade**<br>**(Refer to NCI CTCAE apllicable version in study protocol for defining the CTC grade/severity).**	**General Guidance**	**For Any Elevations Described:**<br>– Monitor and evaluate liver function test: AST, ALT, ALP, and TB.<br>– Evaluate for alternative etiologies (e.g., viral hepatitis, disease progression, concomitant medications).
	**Grade 1**	• No dose modifications.<br>• If it worsens, then treat as described for elevations in the row below.	For Grade 1:<br>– Continue LFT monitoring per protocol.
	**Grade 2**	• Hold study drug/study regimen dose until resolution to Grade ≤1. If toxicity worsens, then treat as Grade 3 or Grade 4.<br>• If toxicity improves to Grade ≤1 or baseline, resume study drug/study regimen after completion of steroid taper.<br>• Permanently discontinue study drug/study regimen for any case meeting Hy's law citeria (AST and/or ALT ALT >3 × ULN + bilirubin >2 × ULN without initial findings of cholestasis (i.e., elevated alkaline P04) and in the absence of any alternative cause.[b]	– Regular and frequent checking of LFTs (e.g., every 1 to 2 days) until LFT elevations improve or resolved.<br>– If no resolution to ≤Grade 1 in 1 to 2 days consider discussing with study physician as needed.<br>– If event is persistent (>2 to 3 days) or worsens, promptly start prednisone 1 to 2 mg/kg/day PO or IV equivalent.

<table>
<tr><th>Adverse Events</th><th>Severity Grade of the Event</th><th>Dose Modifications</th><th>Toxicity Management</th></tr>
<tr><td></td><td>Grade 3

Grade 4</td><td>For elevations in transaminases ≤8×ULN and/or in TB ≤5×ULN:
• Hold study drug/study regimen dose until resolution to Grade ≤1 or baseline.
• Resume study drug/study regimen if elevations downgrade to Grade≤1 or baseline within 14 days and after completion of steroid taper.
• Permanently discontinue study drug/study regimen if the elevations do not downgrade to Grade≤1 or baseline within 14 days.
For elevations in transaminases >8×ULN or elevations in bilirubin >5×ULN permanently discontinue study drug/study regimen.

For Grade 4:
Permanently discontinue study drug/study regimen</td><td>For Grade 3 or 4:
– Promptly initiate empiric IV methylprednisolone at 1 to 2 mg/kg/day or equivalent.
– If still no improvement within 2 to 3 days despite 1 to 2 mg/kg/day methylprednisolone IV or equivalent, promptly start treatment with an immunosuppressant therapy (i.e., mycophenolate mofetil 0.5 – 1 g every 12 hours then taper in consultation with hepatology consult). Discuss with study physician if mycophenolate is not available. Infliximab should NOT be used.
– Performt Hepatology Consult, abdominal workup and imaging as appropriate.</td></tr>
<tr><td>Hepatitis (elevated LFTs)</td><td>Any Elevations in AST, ALT or TB as Described Below</td><td>General Guidance</td><td>For Any Elevations Described:
– Monitor and evaluate liver function test: AST, ALT, ALP, and TB.</td></tr>
</table>

Adverse Events	Severity Grade of the Event	Dose Modifications	Toxicity Management
Infliximab should not be used for management of immune-related<br>**THIS shaded area is guidance *only* for management of "Hepatitis (elevated LFTs)" in HCC patients**			– Evaluate for alternative etiologies (e.g., viral hepatitis, disease progression, concomitant medications, worsening of liver cirrhosis [e.g., portal vein thrombosis]).<br>– For HBV+ patients: evaluate quantitative HBV viral load, quantitative HBsAg, or HBeAg<br>– For HCV+ patients: evaluate quantitative HCV viral load<br>– Consider consulting Hepatology or Infectious Diseases specialists regarding changing or starting antiviral HBV medications if HBV viral load is >2000 IU/ml<br>– Consider consulting Hepatology or Infectious Diseases specialist regarding changing or starting antiviral HCV medications if HCV viral load has increased by ≥2-fold<br>– For HCV+ with HBcAb+: Evaluate for both HBV and HCV as above.
hepatitis.<br>See instructions at bottom of shaded area if transaminase rise is not isolated but (at any time) occurs in setting of either **increasing bilirubin or signs of DILI/liver decompensation**	**Isolated AST or ALT >ULN and ≤5.0×ULN, whether normal or elevated at baseline**	• No dose modifications.<br>• If ALT/AST elevations represents significant worsening based on investigator assessment, then treat as described for elevations in the row below.<br>For all transaminase elevations, see instructions at bottom of shaded area if transaminase rise is not isolated but (at any time) occurs in setting of either **increasing bilirubin or signs of DILI/liver decompensation**	

Adverse Events	Severity Grade of the Event	Dose Modifications	Toxicity Management
	**Isolated AST or ALT >5.0×ULN and ≤8.0×ULN, if normal at baseline**<br><br>**Isolated AST or ALT >2.0×baseline and ≤12.5×ULN, if elevated >ULN at baseline**	• Hold study drug/study regimen dose until resolution to AST or ALT ≤5.0×ULN.<br>• If toxicity worsens, then treat as described for elevations in the rows below.<br><br>If toxicity improves to AST or ALT ≤5.0×ULN, resume study drug/study regimen after completion of steroid taper.<br><br>Permanently discontinue study drug/study regimen for any case meeting Hy's law criteria, in the absence of any alternative cause[b]	– Regular and frequent checking of LFTs (e.g., every 1 to 3 days) until elevations of these are improving or resolved.<br>– Recommend consult hepatologist; consider abdominal ultrasound, including Doppler assessment of liver perfusion.<br>– Consider, as necessary, discussing with study physician.<br>– If event is persistent (>2 to 3 days) or worsens, and investigator suspects toxicity to be im AE, start prednisone 1 to 2 mg/kg/day PO or IV equivalent.<br>– If still no improvement within 2 to 3 days despite 1 to 2 mg/kg/day of prednisone PO or IV equivalent, consider additional workup. If still no improvement within 2 to 3 days despite 1 to 2 mg/kg/day of IV methylprednisolone, consider additional abdominal workup (including liver biopsy) and imaging (i.e., liver ultrasound), and consider starting immunosuppressants (i.e., mycophenolate mofetil 0.5 – 1 g every 12 hours then taper in consultation with hepatology consult).[a] Discuss with study physician if mycophenolate mofetil is not available. **Infliximab should NOT be used.**
	**Isolated AST or ALT >8.0×ULN and ≤20.0×ULN, if normal at baseline**<br><br>**Isolated AST or ALT >12.5×ULN and ≤20.0×ULN, if elevated >ULN at baseline**	• Hold study drug/study regimen dose until resolution to AST or ALT ≤5.0×ULN<br>• Resume study drug/study regimen if elevations downgrade to AST or ALT ≤5.0×ULN within 14 days and after completion of steroid taper.<br>• Permanently discontinue study drug/study regimen if the elevations do not downgrade to AST or ALT ≤5.0×ULN within 14 days	– Regular and frequent checking of LFTs (e.g., every 1-2 days) until elevations of these are improving or resolved.<br>– Consult hepatologist (unless investigator is hepatologist); obtain abdominal ultrasound, including Doppler assessment of liver perfusion; and consider liver biopsy.<br>– Considerdiscussing with study physician as needed.<br>– If investigator suspects toxicity to be immune-mediated, promptly initiate empiric IV methylprednisolone at 1 to 2 mg/kg/day or equivalent.<br>– If no improvement within 2 to 3 days despite 1 to 2 mg/kg/day methylprednisolone IV or equivalent, obtain liver biopsy (if it has not been done already) and promptly start treatment with an immunosuppressive therapy (mycophenolate mofetil 0.5 – 1g every 12 hours then taper in consultation with hepatology consult).

Adverse Events	Severity Grade of the Event	Dose Modifications	Toxicity Management
			Discuss with study physician if mycophenolate is not available. **Infliximab should NOT be used.** –
	**Isolated AST or ALT >20×ULN, whether normal or elevated at baseline**	Permanently discontinue study drug/study regimen.	**Same as above**<br>**(except would recommend obtaining liver biopsy early)**
**If transaminase rise is not isolated but (at any time) occurs in setting of either increasing total/direct bilirubin (≥1.5×ULN, if normal at baseline; or 2×baseline, if >ULN at baseline) or signs of DILI/liver decompensation (e.g., fever, elevated INR):**<br>- **Manage dosing for each level of transaminase rise as instructed for the next highest level of transaminase riseFor example, manage dosing for second level of transaminase rise (i.e., AST or ALT >5.0×ULN and ≤8.0×ULN, if normal at baseline, or AST or ALT >2.0×baseline and ≤12.5×ULN, if elevated >ULN at baseline) as instructed for the third level of transaminase rise (i.e., AST or ALT >8.0×ULN and ≤20.0×ULN, if normal at baseline, or AST or ALT >12.5×ULN and ≤20.0×ULN, if elevated >ULN at baseline)**<br>- **For the third and fourth levels of transaminase rises, permanently discontinue study drug/study regimen**			
**Nephritis or renal dysfunction**<br>(elevated serum creatinine)	**Any Grade**<br>(Refer to NCI CTCAE applicable version in study protocol for defining the CTC grade/severity)	**General Guidance**	**For Any Grade:**<br>– Consult a nephrologist.<br>– Monitor for signs and symptoms that may be related to changes in renal function (e.g., routine urinalysis, elevated serum BUN and creatinine, decreased creatinine clearance, electrolyte imbalance, decrease in urine output, or proteinuria).<br>– Patients should be thoroughly evaluated to rule out any alternative etiology (e.g., disease progression, infections, recent IV contrast, medications, fluid status).

Adverse Events	Severity Grade of the Event	Dose Modifications	Toxicity Management
			– Consider using steroids in the absence of clear alternative etiology even for low-grade events (Grade 2), in order to prevent potential progression to higher grade event.
	**Grade 1**	No dose modifications.	**For Grade 1:**<br>– Monitor serum creatinine weekly and any accompanying symptoms.<br>• If creatinine returns to baseline, resume its regular monitoring per study protocol.<br>• If creatinine worsens, depending on the severity, treat as Grade 2, 3, or 4.<br>– Consider symptomatic treatment, including hydration, electrolyte replacement, and diuretics.<br>– If baseline serum creatinine is elevated above normal, and there is a rise to > 1 to 1.5 x baseline, consider following recommendations in this row.
	**Grade 2**	Hold study drug/study regimen until resolution to Grade ≤1 or baseline.<br>• If toxicity worsens, then treat as Grade 3 or 4.<br>• If toxicity improves to Grade ≤1 or baseline, then resume study drug/study regimen after completion of steroid taper.	**For Grade 2:**<br>– Consider symptomatic treatment, including hydration, electrolyte replacement, and diuretics.<br>– Carefully monitor serum creatinine every 2 to 3 days and as clinically warranted.<br>– Consult nephrologist and consider renal biopsy if clinically indicated.<br>– If event is persistent beyond 3 to 5 days or worsens, promptly start prednisone 1 to 2 mg/kg/day PO or IV equivalent.<br>– If event is not responsive within 3 to 5 days or worsens despite prednisone at 1 to 2 mg/kg/day PO or IV equivalent, consider additional workup.<br>– When event returns to baseline, resume study drug/study regimen and routine serum creatinine monitoring per study protocol.

Adverse Events	Severity Grade of the Event	Dose Modifications	Toxicity Management
	**Grade 3 or 4**	Permanently discontinue study drug/study regimen.	**For Grade 3 or 4:**<br>– Carefully monitor serum creatinine..<br>– Consult nephrologist and consider renal biopsy if clinically indicated.<br>– Promptly start prednisone 1 to 2 mg/kg/day PO or IV equivalent.<br>– If event is not responsive within 3 to 5 days or worsens despite prednisone at 1 to 2 mg/kg/day PO or IV equivalent, consider additional workup and prompt treatment with an immunosuppressant in consultation with a nephrologist.
**Rash or Dermatitis (including Pemphigoid)**	**Any Grade**<br>(Refer to NCI CTCAE applicablE version in study protocol for definition of severity/grade depending on type of skin rash)	**General Guidance**	**For Any Grade:**<br>– Monitor for signs and symptoms of dermatitis (rash and pruritus).<br>– **HOLD STUDY DRUG IF STEVENS-JOHNSON SYNDROME (SJS), TOXIC EPIDERMAL NECROLYSIS (TEN), OR OTHER SEVERE CUTANEOUS ADVERSE REACTION (SCAR) IS SUSPECTED.**<br>– **PERMANENTLY DISCONTINUE STUDY DRUG IF SJS, TEN, OR SCAR IS CONFIRMED.**
	**Grade 1**	No dose modifications.	**For Grade 1:**<br>– Consider symptomatic treatment, including oral antipruritics (e.g., diphenhydramine or hydroxyzine) and topical therapy (e.g., emolient lotion or institutional standard).
	**Grade 2**	For persistent (>1 week) Grade 2 events, hold scheduled study drug/study regimen until resolution to Grade ≤1 or baseline.<br>• If toxicity worsens, then treat as Grade 3.<br>• If toxicity improves to Grade ≤1 or baseline, then resume	**For Grade 2:**<br>– Obtain Dermatology consult.<br>– Consider symptomatic treatment, including oral antipruritics (e.g., diphenhydramine or hydroxyzine) and topical therapy.<br>– Consider moderate-strength topical steroid.<br>– If no improvement of rash/skin lesions occurs within 3 days or is worsening despite symptomatic treatment and/or use of moderate strength topical steroid, consider discussing with study physician as

Adverse Events	Severity Grade of the Event	Dose Modifications	Toxicity Management
		drug/study regimen after completion of steroid taper.	needed, and promptly start systemic steroids such as prednisone 1 to 2 mg/kg/day PO or IV equivalent.<br>– Consider skin biopsy if the event persists for >1 week or recurs.
	**Grade 3 or 4**	**For Grade 3:**<br>Hold study drug/study regimen until resolution to Grade ≤1 or baseline.<br>• If toxicity improves to Grade ≤1 or baseline, then resume drug/study regimen after completion of steroid taper.<br>• If toxicity worsens, then treat as Grade 4<br><br>**For Grade 4:**<br>Permanently discontinue study drug/study regimen.	**For Grade 3 or 4:**<br>– Consult Dermatology.<br>– Promptly initiate empiric IV methylprednisolone 1 to 2 mg/kg/day or equivalent.<br>– Consider hospitalization.<br>– Monitor extent of rash [Rule of Nines].<br>– Consider skin biopsy (preferably more than 1) as clinically feasible.<br>– Consider, as necessary, discussing with study physician.
**Endocrinopathy**<br>(e.g., hyperthyroidism, thyroiditis, hypothyroidism, Type 1 diabetes mellitus, hypophysitis, hypopituitarism, and adrenal insufficiency)	**Any Grade**<br>(depending on the typr od endocrinopathy, refer to NCI CTCAE applicable version in study protocol for defining the CTC grade/severity).	**General Guidance**	**For Any Grade:**<br>– Consider consulting an endocrinologist for endocrine events.<br>– Consider discussing with study physician as needed.<br>– Monitor patients for signs and symptoms of endocrinopathies. Non-specific symptoms include headache, fatigue, behaviour changes, mental status changes, photophobia, visual field cuts, vertigo, abdominal pain, unusual bowel habits, polydipsia, polyuria, hypotension, and weakness.<br>– Patients should be thoroughly evaluated to rule out any alternative etiology (e.g., disease progression including brain metastases, or infections).

<table>
<tr><th>Adverse Events</th><th>Severity Grade of the Event</th><th>Dose Modifications</th><th>Toxicity Management</th></tr>
<tr><td></td><td></td><td></td><td>– Depending on the suspected endocrinopathy, monitor and evaluate thyroid function tests: TSH, free T3 and free T4 and other relevant endocrine and related labs (e.g., blood glucose and ketone levels, HgA1c).
– If a patient experiences an AE that is thought to be possibly of autoimmune nature (e.g., thyroiditis, pancreatitis, hypophysitis, or diabetes insipidus), the investigator should send a blood sample for appropriate autoimmune antibody testing.
– Investigators should ask subjects with endocrinopathies who may require prolonged or continued hormonal replacement, to consult their primary care physicians or endocrinologists about further monitoring and treatment after completion of the study.</td></tr>
<tr><td></td><td>Grade 1</td><td>No dose modifications.</td><td>For Grade 1
– Monitor patient with appropriate endocrine function tests.
– For suspected hypophysitis/hypopituitarism, consider consulting an endocrinologist to guide assessment of early-morning ACTH, cortisol, TSH and free T4; also consider gonadotropins, sex hormones, and prolactin levels, as well as cosyntropin stimulation test (though it may not be useful in diagnosing early secondary adrenal insufficiency).
– If TSH < 0.5 × LLN, or TSH >2 × ULN, or consistently out of range in 2 subsequent measurements, include free T4 at subsequent cycles as clinically indicated and consider consultation of an endocrinologist.</td></tr>
<tr><td></td><td>Grade 2, 3 or 4</td><td>For Grade 2-4 endocrinopathies other than hypothyroidism and Type 1 diabetes mellitus, consider holding study drug/study regimen dose until acute symptoms resolve..</td><td>For Grade 2, 3 or 4
– Consult endocrinologist to guide evaluation of endocrine function and, as indicated by suspected endocrinopathy and as clinically indicated, consider pituitary scan.
– For all patients with abnormal endocrine work up, except those with isolated hypothyroidism or type 1 DM, and as guided by an endocrinologist, consider short-term corticosteroids (e.g., 1 to 2 mg/kg/day methylprednisolone or IV equivalent) and prompt</td></tr>
</table>

Adverse Events	Severity Grade of the Event	Dose Modifications	Toxicity Management
		Study drug/study regimen can be resumed once patient stabilizes and after completion of steroid taper.<br>1. Patients with endocrinopathies who may require prolonged or continued steroid replacement (e.g., adrenal insufficiency) can be retreated with study drug/study regimen Patient is clinically stable as per investigator or treating physician's clinical judgement.<br>2. If toxicity worsens, then treat based on severity.	initiation of treatment with relevant hormone replacement (e.g., hydrocortisone, sex hormones).<br>– Isolated hypothyroidism may be treated with replacement therapy, without study drug/study regimen interruption, and without corticosteroids.<br>– Isolated type 1 diabetes mellitus (DM) may be treated with appropriate diabetic therapy, and without corticosteroids. **Only hold study drug/study regimen in setting of hyperglycaemia when diagnostic workup is positive for diabetic ketoacidosis.**<br>– For patients with normal endocrine workup (laboratory assessment or MRI scans), repeat laboratory assessments/MRI as clinically indicated.
**Amylase/Lipase increased**	**Any Grade**<br>(Refer to NCI CTCAE applicable version in study protocol for defining the CTC grade/severity)		– For modest asymptomatic elevations in serum amylase and lipase, corticosteroid treatment is not indicated as long as there are no other signs or symptoms of pancreatic inflammation.<br>– Assess for signs/symptoms of pancreatitis.<br>– Consider appropriate diagnostic testing (e.g., abdominal with contrast, MRCP if clinical suspicion of pancreatitis and no radiologic evidence on CT.<br>– If isolated elevation of enzymes without evidence of pancreatitis, continue immunotherapy. Consider other causes of elevated amylase/lipase.<br>– If evidence of pancreatitis, manage according to pancreatitis recommendations.
	**Grade 1**	No dose modifications	
	**Grade 2, 3, or 4**	**For Grade 2, 3 or 4:**<br>In consultation with relevant pancreatic specialist consider continuing study drug/study regimen if no clinical/radiologic evidence of	

Adverse Events	Severity Grade of the Event	Dose Modifications	Toxicity Management
		pancreatitis ± improvement in amylase/lipase.	
**Acute Pancreatitis** (to include but not be limited to limbic encephalitis and autonomic neuropathy, excluding Myasthenia Gravis and Guillain-Barre)	**Any Grade** (Refer to NCI CTCAE applicable version in study protocol for defining the CTC grade/severity)	**General Guidance**	**For Any Grade:** Consider Gastroenterology referral
	**Grade 1**	No dose modifications	**For Grade 1:**<br>– IV hydration<br>– Manage as per amylase/lipase increased (asymptomatic)
	**Grade 2, 3, or 4**	**For Grade 2:**<br>Hold study drug/study regimen dose until resolution to Grade ≤1<br>**For Grade 3 or 4:**<br>Permanently discontinue study drug/study regimen.	**For Grade 2, 3 or 4:**<br>– Promptly start systemic steroids prenisone 1 to 2 mg/kg/day PO or equivalent<br>– IV hydration
**Neurotoxicity** (to include but not be limited to non-infectious meningitis, non-	**Any Grade** (Depending on the type of neurotoxicity, refer to NCI CTCAE applicable	**General Guidance**	**For Any Grade:**<br>– Patients should be evaluated to rule out any alternative etiology (e.g., disease progression, infections, metabolic syndromes, or medications).

Adverse Events	Severity Grade of the Event	Dose Modifications	Toxicity Management
infectious encephalitis and autonomic neuropathy, excluding Myasthenia Gravis and Guillain-Barre)	version in study protocol for defining the CTC grade/severity)		– Monitor patient for general symptoms (headache, nausea, vertigo, behaviour change, or weakness).<br>– Consider appropriate diagnostic testing (e.g., electromyogram and nerve conduction investigations).<br>– Perform symptomatic treatment with Neurology consult as appropriate.<br>– **FOR TRANSVERSE MYELITIS, PERMANENTLY DISCONTINUE FOR ANY GRADE**
	**Grade 1**	No dose modifications.	**For Grade 1:**<br>– See "Any Grade" recommendations above.<br>– Treat mild signs/symptoms as Grade 1 (e.g. loss of deep tendon reflexes or paresthesia).
	**Grade 2**	For acute motor neuropathies or neurotoxicity, hold study drug/study regimen dose until resolution to Grade ≤1.<br><br>For sensory neuropathy/neuropathic pain, consider holding study drug/study regimen dose until resolution to Grade ≤1.<br>Permanently discontinue study drug/study regimen if Grade 2 imAE does not resolve to Grade <1 within 30 days.<br>If toxicity worsens, then treat as Grade 3 or 4.	**For Grade 2:**<br>– Consider, as necessary, discussing with the study physician.<br>– Obtain Neurology consult.<br>– Sensory neuropathy/neuropathic pain may be managed by appropriate medications (e.g., gabapentin or duloxetine).<br>– Promptly start systemic steroids prednisone 1 to 2 mg/kg/day PO or IV equivalent.<br>– If no improvement within 2 to 3 days despite 1 to 2 mg/kg/day prednisone PO or IV equivalent, consider additional workup and promptly treat with an additional immunosuppressive therapy (e.g., IV IG or other immunosuppressant depening on the specific imAE).

Adverse Events	Severity Grade of the Event	Dose Modifications	Toxicity Management
	**Grade 3 or 4**	**For Grade 3 or 4:**<br>Permanently discontinue study drug/study regimen.	**For Grade 3 or 4:**<br>– Consider, as necessary, discussing with study physician.<br>– Obtain Neurology consult.<br>– Consider hospitalization.<br>– Promptly initiate empiric IV methylprednisolone 1 to 2 mg/kg/day or equivalent.<br>– If no improvement within 2 to 3 days despite IV corticosteroids, consider additional workup and promptly treat with an additional immunosuppressants (e.g., IV IG or other immunospppresant depending on the specific imAE)).<br>– Once stable, gradually taper steroids over ≥28 days.
**Peripheral neuromotor syndromes**<br>(such as Guillain-Barre and myasthenia gravis)	**Any Grade**<br>(Refer to NCI CTCAE applicable version in study protocol for defining the CTC grade/severity)	**General Guidance**	**For Any Grade:**<br>– The prompt diagnosis of immune-mediated peripheral neuromotor syndromes is important, since certain patients may unpredictably experience acute decompensations that can result in substantial morbidity or in the worst case, death. Special care should be taken for certain sentinel symptoms that may predict a more severe outcome, such as prominent dysphagia, rapidly progressive weakness, and signs of respiratory insufficiency or autonomic instability.<br>– Patients should be evaluated to rule out any alternative etiology (e.g., disease progression, infections, metabolic syndromes or medications). It should be noted that the diagnosis of immune-mediated peripheral neuromotor syndromes can be particularly challenging in patients with underlying cancer, due to the multiple potential confounding effects of cancer (and its treatments) throughout the neuraxis. Given the importance of prompt and accurate diagnosis, it is essential to have a low threshold to obtain a Neurology consult.<br>– Neurophysiologic diagnostic testing (e.g., electromyogram and nerve conduction investigations, and “repetitive stimulation” if myasthenia is suspected) are routinely indicated upon suspicion of

Adverse Events	Severity Grade of the Event	Dose Modifications	Toxicity Management
			such conditions and may be best facilitated by means of a Neurology consultation.<br>– It is important to consider that the use of steroids as the primary treatment of Guillain-Barre is not typically considered effective. Patients requiring treatment should be started with IV IG and followed by plasmapheresis if not responsive to IV IG.
	**Grade 1**	No dose modifications.	**For Grade 1:**<br>– Consider discussing with the study physician as needed.<br>– Care should be taken to monitor patients for sentinel symptoms of a potential decompensation as described above.<br>– Consult a neurologist.
	**Grade 2**	Hold study drug/study regimen dose until resolution to Grade ≤1.<br>Permanently discontinue study drug/study regimen if it does not resolve to Grade ≤1 within 30 days or if there are signs of respiratory insufficiency or autonomic instability.	**For Grade 2:**<br>– Consider discussing with the study physician as needed.<br>– Care should be taken to monitor patients for sentinel symptoms of a potential decompensation as described above.<br>– Consult a neurologist.<br>– Sensory neuropathy/neuropathic pain may be managed by appropriate medications (e.g., gabapentin or duloxetine).<br>*MYASTHENIA GRAVIS:*<br>○ Steroids may be successfully used to treat myasthenia gravis. It is important to consider that steroid therapy (especially with high doses) may result in transient worsening of myasthenia and should typically be administered in a monitored setting under supervision of a consulting neurologist.<br>○ Patients unable to tolerate steroids may be candidates for treatment with plasmapheresis or IV IG. Such decisions are best made in consultation with a neurologist, taking into account the unique needs of each patient.<br>○ If myasthenia gravis-like neurotoxicity is present, consider starting AChE inhibitor therapy in addition to steroids. Such

Adverse Events	Severity Grade of the Event	Dose Modifications	Toxicity Management
			therapy, if successful, can also serve to reinforce the diagnosis.<br>o Avoid medications that can worsen myasthenia gravis.<br>*GUILLAIN-BARRE:*<br>o It is important to consider here that the use of steroids as the primary treatment of Guillain-Barre is not typically considered effective.<br>o Patients requiring treatment should be started with IV IG and followed by plasmapheresis if not responsive to IV IG.
	**Grade 3 or 4**	**For Grade 3:**<br>Hold study drug/study regimen dose until resolution to Grade ≤1.<br>Permanently discontinue study drug/study regimen if Grade 3 imAE does not resolve to Grade ≤1 within 30 days or if there are signs of respiratory insufficiency or autonomic instability.<br>**For Grade 4:**<br>Permanently discontinue study drug/study regimen.	**For Grade 3 or 4 (severe or life-threatening events):**<br>– Consider discussing with study physician as needed.<br>– Recommend hospitalization.<br>– Monitor symptoms and obtain Neurology consult.<br>*MYASTHENIA GRAVIS:*<br>o Steroids may be successfully used to treat myasthenia gravis. They should typically be administered in a monitored setting under supervision of a consulting neurologist.<br>o Patients unable to tolerate steroids may be candidates for treatment with plasmapheresis or IV IG.<br>o If myasthenia gravis-like neurotoxicity present, consider starting AChE inhibitor therapy in addition to steroids. Such therapy, if successful, can also serve to reinforce the diagnosis.<br>o Avoid medications that can worsen myasthenia gravis.<br>*GUILLAIN-BARRE:*<br>o It is important to consider here that the use of steroids as the primary treatment of Guillain-Barre is not typically considered effective.

Adverse Events	Severity Grade of the Event	Dose Modifications	Toxicity Management
			o Patients requiring treatment should be started with IV IG and followed by plasmapheresis if not responsive to IV IG.
**Myocarditis**	**Any Grade**<br>(Refer to NCI CTCAE applicable version in study protocol for defining the CTC grade/severity)	**General Guidance**<br>Discontinue drug permanently if biopsy-proven immune-mediated myocarditis.	**For Any Grade:**<br>– The prompt diagnosis of immune-mediated myocarditis is important, particularly in patients with baseline cardiopulmonary disease and reduced cardiac function.<br>– Consider discussing with the study physician as needed.<br>– Monitor patients for signs and symptoms of myocarditis (new onset or worsening chest pain, arrhythmia, shortness of breath, peripheral edema). As some symptoms can overlap with lung toxicities, simultaneously evaluate for and rule out pulmonary toxicity as well as other causes (e.g., pulmonary embolism, congestive heart failure, malignant pericardial effusion). Consult a cardiologist early to promptly assess whether and when to complete a cardiac biopsy, including any other diagnostic procedures.<br>– Initial work-up should include clinical evaluation, BNP, cardiac enzymes, ECG, echocardiogram (ECHO), monitoring of oxygenation via pulse oximetry (resting and exertion), and additional laboratory work-up as indicated. Spiral CT or cardiac MRI can complement ECHO to assess wall motion abnormalities when needed.<br>– Patients should be thoroughly evaluated to rule out any alternative etiology (e.g., disease progression, other medications, or infections)
	**Grade 1**	No dose modifications required unless clinical suspicion is high, in which case hold study drug/study regimen dose during diagnostic work-up for other etiologies. If study drug/study regimen is held, resume	**For Grade 1:**<br>- Monitor and closely follow up in 2 to 4 days for clinical symptoms, BNP, cardiac enzymes, ECG, ECHO, pulse oximetry (resting and exertion), and laboratory work-up as clinically indicated.<br>- Consider using steroids if clinical suspicion is high.

Adverse Events	Severity Grade of the Event	Dose Modifications	Toxicity Management
		after complete resolution to Grade 0.	
	**Grade 2, 3 or 4**<br>(	- If Grade 2 -- Hold study drug/study regimen dose until resolution to Grade 0. If toxicity rapidly improves to Grade 0, then the decision to reinitiate study drug/study regimen will be based upon treating physician's clinical judgment and after completion of steroid taper. If toxicity does not rapidly improve, permanently. discontinue study drug/study regimen.<br>If Grade 3-4, permanently discontinue study drug/study regimen.	**For Grade 2-4:**<br>– Monitor symptoms daily, hospitalize.<br>– Promptly start IV methylprednisolone 2 to 4 mg/kg/day or equivalent after Cardiology consultation has determined whether and when to complete diagnostic procedures including a cardiac biopsy.<br>– Supportive care (e.g., oxygen).<br>– If no improvement within 2 to 3 days despite IV methylprednisolone at 2 to 4 mg/kg/day, promptly start immunosuppressive therapy such as TNF inhibitors (e.g., infliximab at 5 mg/kg IV, may be repeated at 2 and 6 weeks after initial dose at the discretion of the treating provider. **Caution: It is important to rule out sepsis and refer to infliximab label for general guidance before using infliximab.** Infliximab is contraindicated for patients wo have heart failure.<br>–
**Myositis/Polymyositis**	**Any Grade**<br>(Refer to NCI CTCAE applicable version in study protocol for defining the CTC grade/severity)	**General Guidance**	**For Any Grade:**<br>– Monitor patients for signs and symptoms of poly/myositis. Typically, muscle weakness/pain occurs in proximal muscles including upper arms, thighs, shoulders, hips, neck and back, but rarely affects the extremities including hands and fingers; also difficulty breathing and/or trouble swallowing can occur and progress rapidly. Increased general feelings of tiredness and fatigue may occur, and there can be new-onset falling, difficulty getting up from a fall, and trouble climbing stairs, standing up from a seated position, and/or reaching up.<br>– If poly/myositis is suspected, a Neurology consultation should be obtained early, with prompt guidance on diagnostic procedures.

Adverse Events	Severity Grade of the Event	Dose Modifications	Toxicity Management
			Myocarditis may co-occur with poly/myositis; refer to guidance under Myocarditis. Given breathing complications, refer to guidance under Pneumonitis/ILD.<br>Given possibility of an existent (but previously unknown) autoimmune disorder, consider Rheumatology consultation.<br>– Consider, as necessary, discussing with the study physician.<br>– Initial work-up should include clinical evaluation, creatine kinase, aldolase, LDH, BUN/creatinine, erythrocyte sedimentation rate or C-reactive protein level, urine myoglobin, and additional laboratory work-up as indicated, including a number of possible rheumatological/antibody tests (i.e., consider whether a rheumatologist consultation is indicated and could guide need for rheumatoid factor, antinuclear antibody, anti-smooth muscle, antisynthetase [such as anti-Jo-1], and/or signal-recognition particle antibodies). Confirmatory testing may include electromyography, nerve conduction studies, MRI of the muscles, and/or a muscle biopsy. Consider Barium swallow for evaluation of dysphagia or dysphonia.Patients should be thoroughly evaluated to rule out any alternative etiology (e.g., disease progression, other medications, or infections).
	**Grade 1**	- No dose modifications.	**For Grade 1:**<br>– Monitor and closely follow up in 2 to 4 days for clinical symptoms and initiate evaluation as clinically indicated.<br>– Consider Neurology consult.<br>– Consider, as necessary, discussing with the study physician.
	**Grade 2**	Hold study drug/study regimen dose until resolution to Grade ≤1.<br>- Permanently discontinue study drug/study regimen if it does not resolve to Grade ≤1 within 30 days or if there are signs of respiratory insufficiency.	**For Grade 2:**<br>– Monitor symptoms daily and consider hospitalization.<br>– Obtain Neurology consult, and initiate evaluation.<br>– Consider, as necessary, discussing with the study physician.<br>– If clinical course is rapidly progressive (particularly if difficulty breathing and/or trouble swallowing), promptly start IV methylprednisolone 2 to 4 mg/kg/day systemic steroids along with receiving input from Neurology consultant

Adverse Events	Severity Grade of the Event	Dose Modifications	Toxicity Management
			– If clinical course is *not* rapidly progressive, start systemic steroids (e.g., prednisone 1 to 2 mg/kg/day PO or IV equivalent); if no improvement within 2 to 3 days, continue additional work up and start treatment with IV methylprednisolone 2 to 4 mg/kg/day<br>– If after start of IV methylprednisolone at 2 to 4 mg/kg/day there is no improvement within 2 to 3 days, consider starting another immunosuppressive therapy such as TNF inhibitors (e.g., infliximab at 5 mg/kg IV, may be repeated at 2 and 6 weels after initial dose at the discretion of the treating provider). **Caution: It is important to rule out sepsis and refer to infliximab label for general guidance before using infliximab.**
	Grade 3 or 4	**For Grade 3:**<br>Hold study drug/study regimen dose until resolution to Grade ≤1.<br>Permanently discontinue study drug/study regimen if Grade 3 imAE does not resolve to Grade ≤1 within 30 days or if there are signs of respiratory insufficiency.<br><br>**For Grade 4:**<br>- Permanently discontinue study drug/study regimen.	**For Grade 3 or 4 (severe or life-threatening events):**<br>– Monitor symptoms closely; recommend hospitalization.<br>– Obtain Neurology consult..<br>– Consider, discussing with the study physician as needed.<br>– Promptly start IV methylprednisolone 2 to 4 mg/kg/day systemic steroids along with receiving input from Neurology consultant.<br>– If after start of IV methylprednisolone at 2 to 4 mg/kg/day there is no improvement within 2 to 3 days, consider starting another immunosuppressive therapy such as TNF inhibitors (e.g., infliximab at 5 mg/kg IV, may be repeated at 2 and 6 weeks after initial dose at the discretion of the treating provider). **Caution: It is important to rule out sepsis and refer to infliximab label for general guidance before using infliximab.**<br>– Consider whether patient may require IV IG, plasmapheresis.<br>–

[a]ASCO Educational Book 2015 "Managing Immune Checkpoint Blocking Antibody Side Effects" by Michael Postow MD.
[b]FDA Liver Guidance Document 2009 Guidance for Industry: Drug Induced Liver Injury – Premarketing Clinical Evaluation.
[c]NCCN Clinical Practice Guidelines in Oncology "Management of Immunotherapy-Related Toxicities" Version 1.2020 – December 2019
AChE Acetylcholine esterase; ADL Activities of daily living; AE Adverse event; ALP Alkaline phosphatase test; ALT Alanine aminotransferase; AST Aspartate aminotransferase; BUN Blood urea nitrogen; CT Computed tomography; CTCAE Common Terminology Criteria for Adverse Events; ILD Interstitial lung disease; imAE immune-mediated adverse event; IG Immunoglobulin; IV Intravenous; GI Gastrointestinal; LFT Liver function tests; LLN Lower limit of normal; MRI Magnetic resonance imaging; NCI National Cancer Institute; NCCN National Comprehensive Cancer Network; PJP *Pneumocystis jirovecii* pneumonia (formerly known as *Pneumocystis*

carinii pneumonia); PO By mouth; T3 Triiodothyronine; T4 Thyroxine; TB Total bilirubin; TNF Tumor necrosis factor; TSH Thyroid-stimulating hormone; ULN Upper limit of normal.

Table 86: Durvalumab Other-Immune-Mediated Reactions

Severity Grade of the Event (Refer to NCI CTCAE applicable version in study protocol for defining the CTC grade/severity)	Dose Modifications	Toxicity Management
**Any Grade**	Note: It is possible that events with an inflammatory or immune mediated mechanism could occur in nearly all organs, some of them are not noted specifically in these guidelines (e.g. immune thrombocytopenia, haemolytic anaemia, uveitis, vasculitis).	**For Any Grade:**<br>– The study physician may be contacted for immune-mediatedreactions not listed in the "specific immune-mediated reactions" section<br>– Thorough evaluation to rule out any alternative etiology (e.g., disease progression, concomitant medications, and infections)<br>– Consultation with relevant specialist<br>– Treat accordingly, as per institutional standard.
**Grade 1**	No dose modifications	Monitor as clinically indicated
**Grade 2**	**For Grade 2:**<br>– Hold study drug/study regimen until resolution to ≤Grade 1 or baseline.<br>– If toxicity worsens, then treat as Grade 3 or Grade 4.<br>– Study drug/study regimen can be resumed once event stabilizes to Grade ≤1 after completion of steroid taper.<br>– Consider whether study drug/study regimen should be permanently discontinued in Grade 2 events with high likelihood for morbidity and/or mortality when they do not rapidly improve to Grade <1 upon treatment with systemic steroids and following full taper.	**For Grade 2, 3 or 4:**<br>Treat accordingly, as per institutional standard, appropriate clinical practice guidelines, and other society guidelines (e.g. NCCN, ESMO)

Severity Grade of the Event (Refer to NCI CTCAE applicable version in study protocol for defining the CTC grade/severity)	Dose Modifications	Toxicity Management
**Grade 3**	Hold study drug/study regimen.	
**Grade 4**	Permanently discontinue study drug/study regimen	

CTCAE Common Terminology Criteria for Adverse Events; IM intramuscular; IV intravenous; NCI National Cancer Institute.

Table 87: Durvalumab Infusion-Related Reactions

Severity Grade of the Event (NCI CTCAE version 4.03)	Dose Modifications	Toxicity Management
**Any Grade**	General Guidance	**For Any Grade:**<br>– Manage per institutional standard at the discretion of investigator.<br>– Monitor patients for signs and symptoms of infusion-related reactions (e.g., fever and/or shaking chills, flushing and/or itching, alterations in heart rate and blood pressure, dyspnea or chest discomfort, or skin rashes) and anaphylaxis (e.g., generalized urticaria, angioedema, wheezing, hypotension, or tachycardia).
**Grade 1 or 2**	**For Grade 1:**<br>The infusion rate of study drug/study regimen may be decreased by 50% or temporarily interrupted until resolution of the event.<br>**For Grade 2:**<br>The infusion rate of study drug/study regimen may be decreased 50% or temporarily interrupted until resolution of the event.<br>Subsequent infusions may be given at 50% of the initial infusion rate.	**For Grade 1 or 2:**<br>– Acetaminophen and/or antihistamines may be administered per institutional standard at the discretion of the investigator.<br>– Consider premedication per institutional standard prior to subsequent doses.<br>– Steroids should not be used for routine premedication of Grade ≤2 infusion reactions.
**Grade 3 or 4**	**For Grade 3 or 4:**<br>Permanently discontinue study drug/study regimen.	**For Grade 3 or 4:**<br>– Manage severe infusion-related reactions per institutional standards (e.g., IM epinephrine, followed by IV diphenhydramine and famotidine, and IV glucocorticoid).

CTCAE Common Terminology Criteria for Adverse Events; IM intramuscular; IV intravenous; NCI National Cancer Institute.

Table 88: Durvalumab Non - Immune-Mediated Reactions

Severity Grade of the Event (NCI CTCAE version 5.0)	Dose Modifications	Toxicity Management
**Any Grade**	Note: Dose modifications are not required for AEs not deemed to be related to study treatment (i.e., events due to underlying disease) or for laboratory abnormalities not deemed to be clinically significant.	Treat accordingly, as per institutional standard.
**Grade 1**	No dose modifications.	Treat accordingly, as per institutional standard.
**Grade 2**	Hold study drug/study regimen until resolution to ≤Grade 1 or baseline.	Treat accordingly, as per institutional standard.
**Grade 3**	Hold study drug/study regimen until resolution to ≤Grade 1 or baseline.<br>For AEs that downgrade to ≤Grade 2 within 7 days or resolve to ≤Grade 1 or baseline within 14 days, resume study drug/study regimen administration. Otherwise, discontinue study drug/study regimen.	Treat accordingly, as per institutional standard.
**Grade 4**	Discontinue study drug/study regimen (Note: For Grade 4 labs, decision to discontinue should be based on accompanying clinical signs/symptoms, the Investigator's clinical judgment, and consultation with the Sponsor.).	Treat accordingly, as per institutional standard.

Note: As applicable, for early phase studies, the following sentence may be added: "Any event greater than or equal to Grade 2, please discuss with Study Physician."
AE Adverse event; CTCAE Common Terminology Criteria for Adverse Events; NCI National Cancer Institute.

30.3.4.6 Treatment delays and discontinuation

Trial treatment dose interruption for conditions other than toxicity resolution should be kept as short as possible. If a patient cannot restart trial treatment within 4 weeks for resolution of intercurrent conditions not related to disease progression or toxicity, it is strongly recommended the patient is permanently discontinued from trial treatment and the case should be discussed with the National Lung Matrix Trial Office.

All dose reductions and interruptions (including any missed doses), and the reasons for the reductions/interruptions are to be recorded in the CRF.

If an AE is clearly attributed to one drug, the other drug may continue without interruption until the criteria for recommencement for the interrupted drug is met (see below).

If either one of AZD6738 or durvalumab is permanently discontinued, then the other compound should also be permanently discontinued.

30.3.4.7 Criteria for recommencement of treatment

The following parameters should be met prior to recommencement of treatment: 1) at the start of every new cycle following a delay/interruption; 2) following a treatment interruption for treatment related toxicity:

- ANC ≥1.0 × 10^9 /L
- Platelets ≥100 × 10^9 /L
- No drug-related non-haematological toxicity grade >3.

It is recommended that treatment is re-started after recovery to at least grade 1 or baseline for all AEs except for:
1. Anaemia for which treatment can restart after recovery to at least grade 2.
2. Thrombocytopenia for which platelets should recover to at least 100 × 10^9 /L before treatment is restarted.
3. From cycle 3 onwards, Neutropenia for which neutrophils should recover to at least 1.5 × 10^9 /L before treatment is restarted.

30.4 References

1. Parkes EE, Walker SM, Taggart LE, McCabe N, Knight LA, Wilkinson R, McCloskey KD, Buckley NE, Savage KI, Salto-Tellez M, McQuaid S, Harte MT, Mullan PB, Harkin DP, Kennedy RD. Activation of STING-Dependent Innate Immune Signaling By S-Phase-Specific DNA Damage in Breast Cancer. J Natl Cancer Inst. 2016 Oct 5;109(1).
2. Mackenzie KJ, Carroll P, Martin CA, Murina O, Fluteau A, Simpson DJ, Olova N, Sutcliffe H, Rainger JK, Leitch A, Osborn RT, Wheeler AP, Nowotny M, Gilbert N, Chandra T, Reijns MAM, Jackson AP. cGAS surveillance of micronuclei links genome instability to innate immunity. Nature. 2017 Aug 24;548(7668):461-465
3. Harding SM, Benci JL, Irianto J, Discher DE, Minn AJ, Greenberg RA. Mitotic progression following DNA damage enables pattern recognition within micronuclei. Nature. 2017 Aug 24;548(7668):466-470.

4. Dillon MT, Barker HE, Pedersen M, Hafsi H, Bhide SA, Newbold KL, Nutting CM, McLaughlin M, Harrington KJ. Radiosensitization by the ATR Inhibitor AZD6738 through Generation of Acentric Micronuclei. Mol Cancer Ther. 2017 Jan;16(1):25-34.
5. Dillon MT, Bergerhoff KF, Pedersen M, Patin EC, Whittock H, Crespo-Rodriguez E, Pearson A, Paget JT, Smith HG, Patel RR, Foo S, Bozhanova G, Ragulan C, Fontana E, Desai K, Wilkins AC, Sadanandam A, Melcher A, McLaughlin M, Harrington KJ. ATR inhibition potentiates the radiation induced inflammatory tumour microenvironment. Clin Cancer Res. 2019 Feb 15. pii: clincanres.1821.2018
6. Takaoka A, Wang Z, Choi MK, Yanai H, Negishi H, Ban T, Lu Y, Miyagishi M, Kodama T, Honda K, Ohba Y, Taniguchi T. DAI (DLM-1/ZBP1) is a cytosolic DNA sensor and an activator of innate immune response. Nature. 2007 Jul 26;448(7152):501-5.
7. Abulaiti A, Fikaris AJ, Tsygankova OM, Meinkoth JL. Ras induces chromosome instability and abrogation of the DNA damage response. Cancer Res. 2006 Nov 1;66(21):10505-12.
8. Gilad O, Nabet BY, Ragland RL, Schoppy DW, Smith KD, Durham AC, Brown EJ. Combining ATR suppression with oncogenic Ras synergistically increases genomic instability, causing synthetic lethality or tumorigenesis in a dosage-dependent manner. Cancer Res. 2010 Dec 1;70(23):9693-702.
9. Vendetti FP, Karukonda P, Clump DA, Teo T, Lalonde R, Nugent K, Ballew M, Kiesel BF, Beumer JH, Sarkar SN, Conrads TP, O'Connor MJ, Ferris RL, Tran PT, Delgoffe GM, Bakkenist CJ. ATR kinase inhibitor AZD6738 potentiates CD8+ T cell-dependent antitumor activity following radiation. J Clin Invest. 2018 Aug 31;128(9):3926-3940.
10. Skoulidis F, Byers LA, Diao L, Papadimitrakopoulou VA, Tong P, Izzo J, Behrens C, Kadara H, Parra ER, Canales JR, Zhang J, Giri U, Gudikote J, Cortez MA, Yang C, Fan Y, Peyton M, Girard L, Coombes KR, Toniatti C, Heffernan TP, Choi M, Frampton GM, Miller V, Weinstein JN, Herbst RS, Wong KK, Zhang J, Sharma P, Mills GB, Hong WK, Minna JD, Allison JP, Futreal A, Wang J, Wistuba II, Heymach JV. Co-occurring genomic alterations define major subsets of KRAS-mutant lung adenocarcinoma with distinct biology, immune profiles, and therapeutic vulnerabilities. Cancer Discov. 2015 Aug;5(8):860-77.
11. Ferdinandos Skoulidis, Matthew David Hellmann, Mark M. Awad, Hira Rizvi, Brett W. Carter, Warren Denning, Yasir Elamin, Jianjun Zhang, Giulia Costanza Leonardi, Darragh Halpenny, Andrew Plodkowski, Niamh Long, Jeremy J. Erasmus, Vassiliki Papadimitrakopoulou, Kwok-Kin Wong, Ignacio Ivan Wistuba, Pasi A. Janne, Charles M. Rudin, and John Heymach STK11/LKB1 co-mutations to predict for de novo resistance to PD-1/PD-L1 axis blockade in KRAS-mutant lung adenocarcinoma. Journal of Clinical Oncology 2017 35:15_suppl, 9016-9016

31 COHORT NA1: DURVALUMAB – ANTI-PDL1 *(CLOSED TO RECRUITMENT)*

31.1 Background & Rationale

Inhibitor: Durvalumab (formerly called MEDI4736)

Lead Investigator: Professor James Spicer

Arm	Investigational Medicinal Product	Cohort Number	Histology	Molecular Cohort
**NA**	Durvalumab –PD-L1 Inhibitor	**NA1**	NSCLC	No actionable genetic change for other trial arms

Investigators should be familiar with the current durvalumab (MEDI4736) IB.

31.1.1 Pre-Clinical Rationale

Durvalumab is being developed as a potential anticancer therapy for patients with advanced solid tumours. Durvalumab is a human monoclonal antibody (mAb) of the immunoglobulin G1 kappa (IgG1κ) subclass that inhibits binding of programmed cell death ligand 1 (PD-L1) (B7 homolog 1 [B7-H1], cluster of differentiation [CD]274) to programmed cell death 1 (PD-1; CD279) and CD80 (B7-1). Durvalumab is composed of 2 identical heavy chains and 2 identical light chains, with an overall molecular weight of approximately 149 kDa. Durvalumab contains a triple mutation in the constant domain of the immunoglobulin (Ig) G1 heavy chain that reduces binding to complement protein C1q and the fragment crystallisable gamma (Fcγ) receptors involved in triggering effector function.

The immune system can identify tumour-associated antigens and eliminate the cancerous cells expressing them and thus plays an important role in preventing and combating the growth of tumours. This process of tumour immune surveillance is believed to result in a co-evolution of the tumour and immune response termed immunoediting, which is thought to follow 3 stages (Swann and Smyth 2007):

- During the initial phase of elimination, the innate and adaptive immune systems detect and eliminate tumour cells. Elimination can result in complete clearance of tumour cells as is seen in rare cases of spontaneous regression of melanoma (Kalialis *et al.* 2009).
- However, if elimination is incomplete, the immune system and tumour may enter a state of equilibrium. During this second phase of immunoediting, the immune response selectively eliminates susceptible tumour cells and may prevent tumour progression. As the equilibrium phase persists, the tumour may evolve mechanisms to avoid or attenuate the immune response.
- The emergence of tumour cells with reduced immunogenicity or enhanced immunosuppressive mechanisms leads to the escape phase of immunoediting. During the escape phase, many factors may contribute to the failure of the immune system to control tumour growth including the expression of immune-inhibitory molecules, presence of immunosuppressive regulatory T lymphocytes (T cells) or immunosuppressive cytokines within the tumour microenvironment, and down regulation of major histocompatibility molecules and tumour antigens leading to reduced antigen presentation and recognition.

Blockade of negative regulatory signals to T-cells such as cytotoxic T-lymphocyte antigen 4 (CTLA-4) and programmed death ligand 1 (PD-L1) has also shown promising clinical activity. Ipilimumab binds to CTLA-4 and prevents the interaction of CTLA-4 with cluster of differentiation (CD) 80 and CD86, resulting in enhanced T-cell activation and proliferation (Lipson and Drake 2011). Ipilimumab was granted United States (US) Food and Drug Administration (FDA) approval in 2011 for the treatment of metastatic melanoma and is currently under investigation for several other malignancies.

PD-L1 (B7 homolog 1 [B7 H1], CD274) is part of a complex system of receptors and ligands that are involved in controlling T-cell activation. In normal tissue, PD-L1 is expressed on T cells, B lymphocytes (B-cells), dendritic cells, macrophages, mesenchymal stem cells, bone marrow-derived mast cells, as well as various nonhaematopoietic cells (Keir *et al.* 2008). The normal function of PD-L1 is to regulate the balance between T-cell activation and tolerance through interaction with 2 receptors, PD-1 (CD279) and CD80 (B7-1). PD-L1 is also expressed by tumours and acts at multiple sites to help tumours evade detection and elimination by the host immune system tumour response. In the lymph nodes, PD-L1 on antigen-presenting cells (APCs) binds to PD-1 (CD279) or CD80 (B7-1) on activated T-cells and delivers an inhibitory signal to the T-cell (Keir *et al.* 2008, Park *et al.* 2010). Likewise, binding of CD80 on APCs to PD-L1 on T-cells leads to inhibitory signalling in the T-cell. These and bidirectional interactions between CD80 and PD-L1, expressed on both APCs and T-cells, lead to further inhibition of T-cell activation. These interactions result in reduced T-cell activation and fewer activated T-cells in the circulation. In the tumour microenvironment, PD-L1 expressed on tumour cells binds to PD-1 on activated T-cells reaching the tumour. This delivers an inhibitory signal to those T-cells, preventing them from killing the target tumour cells, and thus protecting the tumour from immune elimination (Zou and Chen 2008).

PD-L1 is expressed in a broad range of cancers with a high frequency, up to 88% in some types of cancer. In a number of these cancers, including lung (Mu *et al.* 2011), the expression of PD-L1 is associated with reduced survival and an unfavourable prognosis. In lung cancer, only 20% of patients with tumours expressing PD-L1 survived for more than 3 years, compared with 49% of patients with tumours lacking PD-L1 (Mu *et al.* 2011). Based on these data, and on assessments of expression of PD-L1 on the surface of human tumours using proprietary IHC methods for assessment, durvalumab has the potential to affect multiple types of solid tumours, including those with a high incidence rate and some less common types with limited treatment options and poor outcomes.

The levels of tumour-infiltrating lymphocytes, and more specifically cytotoxic T-cells, have been correlated to improved prognosis in a number of cancers including colorectal, melanoma, and lung cancers (Pagès *et al.* 2010), suggesting that an anti-tumour immune response is beneficial to patients. *In vitro*, an antibody that blocks the interaction between PD-L1 and its receptors can relieve PD-L1-dependent immunosuppressive effects and enhance the cytotoxic activity of anti-tumour T-cells (Blank *et al.* 2006). Based on these findings, an anti-PD-L1 antibody could be used therapeutically to enhance anti-tumour immune responses in patients with cancer. Results of several preclinical studies using mouse tumour models support this hypothesis, where antibodies directed against PD-L1, or its receptor PD-1, showed anti-tumour activity (Hirano *et al.* 2005, Iwai *et al.* 2002, Okudaira *et al*. 2009, Zhang *et al.* 2008).

Blocking PD-L1 is a similar approach to that taken by ipilimumab, but has some potential advantages. Firstly, the expression of CTLA-4 and its ligands is restricted to the haematopoietic system; thus, the site of action for molecules targeting CTLA-4 is solely the peripheral lymphoid organs. In contrast, PD-L1 is expressed not only on cells of the haematopoietic system but also on a range of tumour types. Targeting of PD-L1 could therefore have additional effects within the tumour microenvironment. Secondly, CTLA-4 plays an early and critical role in controlling T-cell activation. This is reflected in the phenotype of CTLA-4

knockout mice, which die at an age of between 3 and 4 weeks due to lymphoproliferative disease and tissue destruction. In contrast PD-L1, via binding to PD-1, acts later in the process of T-cell activation (Fife and Bluestone 2008) and is considered more dispensable for the control of initial T-cell activation. This is reflected in the phenotype of PD-L1 knockout mice, which are viable and have normal T-cell numbers and activation levels, but that have increased T-cell activation in response to antigen and increased susceptibility in certain autoimmunity models (Dong *et al*. 2004, Latchman *et al.* 2004). Based on these data, inhibition of PD-L1 would be expected to have reduced toxicity relative to inhibition of CTLA-4. In support of this, recent Phase 1 clinical studies testing the tolerability of agents targeting PD-1 have shown a more favourable toxicity profile than ipilimumab (Berger *et al.* 2008, Brahmer *et al.* 2010, Wolchok *et al*. 2009).

Durvalumab binds with high affinity and specificity to human PD-L1 and blocks its interaction with PD-1 and CD80. *In vitro* studies demonstrate that durvalumab antagonises the inhibitory effect of PD-L1 on primary human T-cells, resulting in restored proliferation and release of interferon (IFN) gamma. Additionally, durvalumab demonstrates a lack of antibody dependent cellular cytotoxicity and complement-dependent cytotoxicity in cell-based functional assays. *In vivo* studies show that durvalumab inhibits tumour growth in a xenograft model via a T-cell dependent mechanism. An anti-mouse PD-L1 antibody has also demonstrated improved survival in a syngeneic tumour model when durvalumab was given as a monotherapy and resulted in complete tumour regression in >50% of treated mice when given in combination with chemotherapy.

The non-clinical experience is fully described in the current version of the durvalumab IB.

31.1.2 Clinical Data

Current therapies for advanced NSCLC have poor outcomes (low 5-year survival [11%], poor response to systemic treatment for patients with refractory advanced NSCLC [approximately 10%], with median survival of approximately 6 months) (D'Addario *et al.* 2010, Hanna *et al.* 2004). There is still a significant unmet medical need for additional treatment options for use in this patient population

Durvalumab has been given to humans as part of ongoing studies where it is given either as a single drug or in combination with other drugs. For full details of the clinical information, please refer to the current IB.

The majority of the safety data currently available for durvalumab are based on the first-time-in-human (FTIH), single agent study (CD ON MEDI4736-1108) in patients with advanced solid tumours. As of 07 May 2015, a total of 736 patients have entered into this study. Of these subjects, 694 have received durvalumab at 10 mg/kg given every 2 weeks (Q2W), either in the dose-escalation or dose-expansion phase of the study.

Of the 694 subjects treated with 10 mg/kg Q2W, 668 (96.2%) had at least 1 AE (regardless of causality). AEs (all grades), reported in decreasing order of frequency, were fatigue, nausea, decreased appetite, dyspnoea, cough, constipation, diarrhoea, vomiting, back pain, pyrexia, abdominal pain, anaemia, arthralgia, peripheral oedema, headache, rash andpruiritis.

Overall, 456 of 694 subjects treated with durvalumab 10 mg/kg Q2W were evaluable for response (defined as having durvalumab 10 mg/kg Q2W were evaluable for response (defined as follow-up scan, or discontinued due to disease progression or death without any follow-up scan). In PD-L1 unselected subjects, the ORR, based on Investigator assessment per RECIST v1.1, ranged from 0% in uveal melanoma (n = 23) to 20.0% in bladder cancer (n = 15), and disease control rate (DCR)-24w ranged from 4.2% in triple-negative breast cancer (n = 24) to

39.1% in advanced cutaneous melanoma (n = 23). PD-L1 status was known for 383 of the 456 response evaluable subjects. Across the PD-L1-positive tumours, ORR was highest for bladder cancer, advanced cutaneous melanoma, hepatocellular carcinoma (HCC; n = 3 each, 33.3% each), NSCLC (n = 86; 26.7%), and squamous cell carcinoma of the head and neck (SCCHN; n = 22; 18.2%). In the PD-L1-positive subset, DCR-24w was highest in advanced cutaneous melanoma (n = 3; 66.7%), NSCLC (n = 86; 36.0%), HCC and bladder cancer (n = 3 each; 33.3% each), and SCCHN (n = 22; 18.2%).

Other monoclonal antibodies targeting the PD-1/PD-L1 pathway are currently in clinical development. Among the most frequent treatment-related AEs noted of these antibodies are fatigue, rash, diarrhoea and pruritus. Immune-mediated AEs of ≥Grade 3 reported include pneumonitis, diarrhoea, ALT increased and AST increased.

Other relevant risks include those associated with biological and immunotherapy agents. Ipilimumab and tremelimumab are both immunomodulatory antibodies that target CTLA 4 and have been studied extensively across multiple tumour types. Ipilimumab is marketed for the treatment of metastatic or unresectable melanoma based on improvements in OS as a single agent and in combination with chemotherapy. Immune-mediated AEs of ≥Grade 3 observed during Phase 3 studies of ipilimumab occurred in 15% of patients and included enterocolitis (7%), hepatitis (2%), dermatitis (2.5%) and endocrinopathy (1.8%) (YERVOY™ prescribing information). Adverse events similar to those reported with ipilimumab were observed during the clinical development of tremelimumab in melanoma, with diarrhoea (40%), pruritus (23%), rash (22%), nausea (22%) and fatigue (17%) being the most common (Kirkwood et al 2010). Grade 3 or higher AEs observed were diarrhoea (11%), fatigue (2%), rash (1%), nausea (1%), vomiting (1%) and anorexia (1%).

There has been much excitement generated by the emerging efficacy data on therapies targeting the PD-L1/PD-1 axis in NSCLC. In the published phase I data using Nivolumab, outcomes in 18 patients with squamous cell cancer and 56 non-squamous cell carcinoma were reported (Topalian *et al.*, 2012). There were no objective responses at the 1mg/kg dose but 3/6 and 3/7 squamous cell patients treated at the 3mg/kg and 10mg/kg dose respectively achieved an objective response to treatment by modified RECIST v1.0. Again no patient with non-squamous NSCLC responded at the 1mg/kg dose: 3/13 and 4/31 responded at the 3mg/kg and 10mg/kg dose respectively. Progression free survival rate at 24 weeks was 37 and 21% respectively. Responses were durable: 8/14 responders had a response duration of 24 weeks or more and of the 5 responding patients starting therapy a year or more before data analysis, 2 had a response lasting a year or more. Initial data suggested that PD-L1 expression was a useful candidate companion diagnostic for Nivolumab, and whilst there are patients with PD-L1 negative tumours who have responded (personal communication, Bristol Meyers Squib), it is apparent from data presented at the European Cancer Conference 2013 from the phase 1 study of MPDL3280A that there is an association between increasing immunoreactivity for PD-L1 and increasing efficacy with PD-L1 blockade (Horn *et al.* 2013). As in the nivolumab series patients were generally heavily pretreated: 53 patients with PD-L1 expression data were evaluable for response. Overall 23% achieved an objective response by RECIST 1.1: again responses appeared durable with 11/12 responders continuing to respond at the time of data cut-off. In those with >10% tumour infiltrating cells positive for PD-L1 by IHC the response rate was 83%; >5% PD-L1 positivity, response rate 46%; >1% positivity, response rate 31% (8/26) with progressive disease rate as best response in 38%. MPDL3280A was well tolerated with 12.6% of patients experiencing a treatment-related grade 3 or 4 adverse event (fatigue 2%, nausea 1%). There were no DLT and no grade 3/4 pneumonitis. These initial data suggest that blockade of the PD-L1/PD-1 axis will become a key intervention in the management of advanced NSCLC.

Emerging data with anti PD-1/PD-L1 agents suggest that subsets of patients defined by a positive tumour PD-L1 expression may have a higher probability for favourable clinical responses to treatment than patients with PD-L1 negative tumours.

However, at present, the available data sets are small and uncontrolled and suggest that a proportion of patients with PD-L1 negative tumours may still respond to treatment. The reasons for this are likely multifactorial relating to the complex and dynamic nature of the immune system and the robustness of currently available tests. Therefore, it is proposed not to restrict enrolment to the No Actionable Genetic change cohort involving durvalumab to a PD-L1 tumour positive sub-population but to carefully assess these patient characteristics in the context of this study.

The non-clinical and clinical experience with durvalumab is fully described in the most current version of the durvalumab IB.

To date durvalumab has been given to more than 1800 subjects as part of ongoing studies either as monotherapy or in combination with other anti-cancer agents. Details on the safety profile of durvalumab monotherapy are summarized in Section 31.3.3. Refer to the current durvalumab IB for a complete summary of non-clinical and clinical information including safety, efficacy and pharmacokinetics.

31.1.2.1 Marketing Experience

Durvalumab has not been approved for marketing purposes anywhere in the world.

31.2 Specific Eligibility Criteria

31.2.1 Inclusion Criteria

For inclusion in the study, patients must fulfil all of the following criteria:

- Patients must fulfil all the core eligibility criteria.
- For patients with EGFRwt tumours, prior therapy must include platinum-based chemotherapy. For patients with tumours harbouring an EGFR mutation known to be associated with EGFR TKI sensitivity, prior therapy must include an EGFR tyrosine kinase inhibitor. For patients with ALK-positive tumours, prior therapy must include an ALK inhibitor.
- A successful SMP2 result. An SMP2 result is defined as successful when ≥25 out of 28 genes have been successfully reported in the Technology Hub report. For clarity, this can be **either** the diagnostic test or the repeat biopsy test.
- Eastern Cooperative Oncology Group (ECOG) Performance Status ≤1 (see Appendix 8: Eastern Cooperative Oncology Group Performance Status Criteria)
- Sufficient tissue (>100 tumour cells) for PDL1 staining. See the National Lung Matrix Trial Laboratory Manual for further information.
- Nonsterilised males who are sexually active with a female partner of childbearing potential must use 2 acceptable methods of effective contraception (condom or diaphragm plus spermicide) for duration of treatment until 90 days after discontinuation of durvalumab.

31.2.2 Exclusion Criteria

Patients must not enter the trial if any of the following exclusion criteria are fulfilled:

- Patients who do not fulfil all the core eligibility criteria.

- Treatment with any of the following:
  - Monoclonal antibodies 6 weeks prior to registration.
  - Receipt of the last dose of anticancer therapy (chemotherapy, immunotherapy, endocrine therapy, targeted therapy, biologic therapy, or tumour embolisation) ≤28 days prior to registration (≤14 days prior to registration patients who have received prior TKIs and within 6 weeks for nitrosourea or mitomycin C). If sufficient wash-out time has not occurred due to the schedule or PK properties of an agent, a longer wash-out period will be required, as agreed by the National Lung Matrix Trial Office and the local Investigator.
  - Current or prior use of immunosuppressive medication within 14 days of registration, with the following exceptions:
    - Intranasal, inhaled, topical steroids, or local steroid injections (e.g. intra articular injection).
    - Systemic corticosteroids at physiological doses, which are not to exceed 10 mg/day of prednisone, or an equivalent.
    - Steroids as premedication for hypersensitivity reactions (e.g. CT scan premedication)
  - Prior exposure to any anti-PD-1 or anti-PD-L1 antibody including durvalumab.
  - Radiotherapy within 4 weeks prior to treatment.
- Hepatic function (in patients **with** liver metastasis).
  - Alanine transferase (ALT) and Aspartate transferase (AST) >5 x ULN.
- Any prior Grade ≥3 immune-related adverse event (irAE) while receiving any previous immunotherapy agent, or any unresolved irAE >Grade 1.
- Mean resting corrected QT interval (QTcF) >470 msec obtained from 3 consecutive ECGs.
- As judged by the local Investigator, any evidence of severe or uncontrolled systemic diseases (e.g., severe hepatic impairment, interstitial lung disease [bilateral, diffuse, parenchymal lung disease], uncontrolled chronic renal diseases (glomerulonephritis, nephritic syndrome, Fanconi Syndrome or Renal tubular acidosis), or current unstable or uncompensated respiratory or cardiac conditions (angina pectoris, cardiac arrhythmia), or uncontrolled hypertension, active bleeding diatheses, active peptic ulcer disease or gastritis, or psychiatric illness/social situations that would limit compliance with study requirements or compromise the ability of the subject to give written informed consent.
- Active infection including tuberculosis (clinical evaluation that includes clinical history, physical examination and radiographic findings, and TB testing in line with local practice), hepatitis B (known positive HBV surface antigen (HBsAg) result), hepatitis C, or human immunodeficiency virus.
- Active or prior documented autoimmune disease or inflammatory disorders (including inflammatory bowel disease e.g., Crohn’s disease or colitis), diverticulitis (with the exception of diverticulosis), systemic lupus, erythematosus, Sarcoidosis syndrome, or Wegener syndrome (granulomatosis with polyangiitis, Grave’s disease, rheumatoid arthritis, hypophysitis, uveitis, etc.). The following are exceptions to this criterion:
  - Subjects with vitiligo or alopecia.
  - Subjects with hypothyroidism (e.g. following Hashimoto syndrome) stable of hormone replacement.
  - Any chronic skin condition that does not require systemic therapy.
  - Subjects without active disease in the last 5 years may be included but only after consultation with the National Lung Matrix Trial Office.
  - Subjects with celiac disease controlled by diet alone.
- History of active primary immunodeficiency.

- History of allogeneic organ transplant.
- Known allergy or hypersensitivity to durvalumab or any excipient.
- Receipt of live attenuated vaccination within 30 days prior to registration.

31.2.3 Restrictions & Concomitant Medications

Information on any treatment from the date of informed consent until 28 days after the administration of the last treatment dose should be recorded. If medically feasible, patients taking regular medication should be maintained on it throughout the study period. Patient should be advised to inform their treating physicians of all concomitant medications, including prescription medicines, over-the-counter drugs, vitamins, and herbal products.

Nb. These lists are not exhaustive and the absence of a drug from the lists does not imply that its combination with durvalumab is safe.

- Any concurrent chemotherapy, radiotherapy (except palliative radiotherapy), immunotherapy, biologic or hormonal therapy for cancer treatment should not be given whilst the patient is on trial treatment. Concurrent use of hormones for non-cancer-related conditions (e.g., insulin for diabetes and hormone replacement therapy) is acceptable. Local treatment of isolated lesions, excluding target lesions, for palliative intent is acceptable (e.g. by local surgery or radiotherapy).
- Immunosuppressive medications including, but not limited to systemic corticosteroids at doses not exceeding 10 mg/day of prednisone or equivalent, methotrexate, azathioprine, and tumour necrosis factor (TNF)-α blockers should not be given concomitantly, or used for premedication prior to the durvalumab infusions. The following are allowed exceptions:
    - Use of immunosuppressive medications for the management of investigational product-related AEs.
    - Use in subjects with contrast allergies.
    - In addition, use of inhaled and intranasal corticosteroids is permitted.
    - A temporary period of steroids will be allowed if clinically indicated, at the discretion of the principal investigator, and considered to be essential for the management of the patient (e.g., chronic obstructive pulmonary disease, radiation, nausea, etc).
- Drugs with laxative properties, and herbal or natural remedies should be used with caution through to 90 days after the last dose of trial treatment.
- Live attenuated vaccines within 30 days of durvalumab dosing (ie, 30 days prior to the first dose, during treatment with durvalumab and for 30 days post discontinuation of durvalumab) are not permitted. Inactivated viruses, such as those in the influenza vaccine, are permitted.

31.2.3.1 Contraception

A definition of females of childbearing potential and females of non-childbearing potential is available in Section 6.3.1.

The following restrictions apply while the patient is receiving study treatment and for the specified times before and after:

Female patient of child-bearing potential:

- Females of childbearing potential who are sexually active with a non-sterilized male partner must use at least 1 **highly** effective method of contraception (see
- Table 88) from the time of screening and must agree to continue using such precautions for 180 days after the last dose of durvalumab + any drug combination

therapy or 90 days after the last dose of durvalumab monotherapy. Non-sterilized male partners of a female patient must use male condom plus spermicide throughout this period. Cessation of birth control after this point should be discussed with a responsible physician. Not engaging in sexual activity for the total duration of the drug treatment and the drug washout period is an acceptable practice; however, periodic abstinence, the rhythm method, and the withdrawal method are not acceptable methods of birth control. Female subjects should also refrain from breastfeeding throughout this period.

Male subjects with a female partner of childbearing potential:

- Non-sterilized males who are sexually active with a female partner of childbearing potential must use a male condom plus spermicide from screening through 180 days after receipt of the final dose of durvalumab + any drug combination therapy or 90 days after receipt of the final dose of durvalumab monotherapy. Not engaging in sexual activity is an acceptable practice; however, occasional abstinence, the rhythm method, and the withdrawal method are not acceptable methods of contraception. Male subjects should refrain from sperm donation throughout this period.

Female partners (of childbearing potential) of male subjects must also use a highly effective method of contraception throughout this perod (see Table 88).

Highly effective methods of contraception, defined as one that results in a low failure rate (i.e. less than 1% per year) when used consistently and correctly are described in Table 88. Note that some contraception methods are not considered highly effective (eg male or female condom with or without spermicide; female cap, diaphragm, or sponge with or without spermicide; non-copper containing intrauterine device; progestogen-only oral hormonal contraceptive pills where inhibition of ovulation is not the primary mode of action [excluding Cerazette/desogestrel which is considered highly effective]; and triphasic combined oral contraceptive pills).

Table 89: Highly Effective Methods of Contraception (<1% Failure Rate)

**Barrier/Intrauterine methods**	**Hormonal Methods**
• Copper T intrauterine device<br>• Levonorgestrel-releasing intrauterine system (eg, Mirena®)	• Etonogestrel implants: eg Implanon or Norplant<br>• Intravaginal device: eg ethinylestradiol and etonogestrel<br>• Medroxyprogesterone injection: eg Depo-Provera<br>• Normal and low dose combined oral contraceptive pill<br>• Norelgestromin/ethinylestradiol transdermal system<br>• Cerazette (desogestrel)

31.3 Trial Treatment

31.3.1 Investigational Medicinal Product

Durvalumab is a human monoclonal antibody of the immunoglobulin (Ig) G1 kappa subclass that inhibits binding of PD-L1 (B7-H1, CD274) to PD-1 (CD279) and CD80 (B7-1) and is in development for the treatment of patients with various malignancies including NSCLC. Durvalumab will be supplied free of charge by AstraZeneca.

Patients receiving durvalumab will commence treatment (10 mg/kg IV) on Day 1 and continue every 2 weeks (Q2W) until confirmed disease progression, unless there is unacceptable toxicity, withdrawal of consent, or another discontinuation criterion is met (please note patients who meet RECIST criteria for progressive disease (PD) may be continued on trial treatment if the treatment is tolerable and the Investigator believes it to be of clinical benefit; see Section 9.3).

Patient weight at baseline (screening visit) should be used for dosing calculations unless there is a ≥10% change in weight since baseline where the dose should be adjusted accordingly. Dose rounding is permitted for ease of measurement, provided the dose remains within 5% of the calculated dose.

If a patient experiences a weight change of ≥10% since baseline, and their dose is recalculated, this dose should be used going forward, and subsequently the weight of the patient at each visit should be compared to the patient's weight at the visit where the dose was last changed. Only if there is another ≥10% weight change since the visit of the previous dose change, should the dose be adjusted again.

Following preparation of durvalumab, the entire contents of the IV bag should be administered as an IV infusion over approximately 60 minutes (±5 minutes), using a 0.2-μm in-line filter. The IV line will be flushed with a volume of normal saline equal to the priming volume of the infusion set used after the contents of the IV bag are fully administered, or complete the infusion according to institutional policy to ensure the full dose is administered and document if the line was not flushed. The durvalumab solution should not be infused through an IV line in which other solutions or medications are being administered.
Please note patients who meet RECIST criteria for progressive disease (PD) may be continued on trial treatment if the treatment is tolerable and the Investigator believes it to be of clinical benefit; see Section 9.3.

Please refer to the Pharmacy Manual for further details.

31.3.2 Schedule of Assessments

Table 90: Durvalumab (MEDI4736) - Schedule of Assessments

Study procedures at Screening and during the Treatment Period **for all patients.** Follow-up for patients have discontinued durvalumab treatment due to confirmed PD **only**.

	**Screening**	**Treatment 10 mg/kg IV every 2 weeks**										**Discontinuation** (+ 7 days)	**28 day follow up[v]** (+ 7 days)*	**Months 2 & 3 post-discontinuation** (± 7 days)**	**Survival Assessments** (± 7 days)**
	Within 28 days of treatment (unless otherwise stated)	W0 (Day 1)	W2	W4	W6	W8	2 wkly 10,12, 14,16, etc.	4 wkly 12,16, 20,24, etc.	6 wkly 12,18, 24,30, etc.	8 wkly 16,24, 32,40 etc.	16 wkly 16,32 48, etc.				
			± 3 days*						± 7 days**						
Informed consent[a]	X														
Mandatory pre-treatment biospy[b]	X (can be collected >28 days before treatment)														
Demography & baseline characteristics[c]	X														
Medical history[d]	X														
Inclusion / exclusion criteria[e]	X														

	Screening<br><br>Within 28 days of treatment (unless otherwise stated)	Treatment 10 mg/kg IV every 2 weeks										Discontinuation (+ 7 days)	28 day follow up[v] (+ 7 days)*	Months 2 & 3 post-discontinuation (± 7 days)**	Survival Assessments (± 7 days)**
		W0 (Day 1)	W2	W4	W6	W8	2 wkly<br>10,12, 14,16, etc.	4 wkly<br>12,16, 20,24, etc.	6 wkly<br>12,18, 24,30, etc.	8 wkly<br>16,24, 32,40 etc.	16 wkly<br>16,32 48, etc.				
			± 3 days*						± 7 days**						
Physical examination[f]	X	X<br>(-1 day)	X	X	X	X	X					X	X		
ECOG performance status	X<br>(within 14 days of treatment)	X		X		X		X				X			
Hepatitis B, C and HIV	X														
Vital signs[g]	X	X	X	X	X	X	X					X			
Weight	X	X<br>(- 2 days)		X<br>(- 2 days)		X<br>(- 2 days)		X<br>(- 2 days)				X			
ECG[h]	X										X	X			
Haematology, Clinical chemistry[i]	X<br>(within 3 days of treatment)	X<br>(-1 day)	X<br>(- 2 days)	X<br>(- 2 days)	X<br>(- 2 days)	X<br>(- 2 days)	X<br>(- 2 days)					X	X<br>(- 2 days)	X<br>(- 2 days)	
Urinalysis[j]	X<br>(within 3 days of treatment)	X		X<br>(- 2 days)		X<br>(- 2 days)		X<br>(- 2 days)				X	X<br>(- 2 days)		

	Screening<br>Within 28 days of treatment (unless otherwise stated)	Treatment 10 mg/kg IV every 2 weeks										Discontinuation (+ 7 days)	28 day follow up[v] (+ 7 days)*	Months 2 & 3 post-discontinuation (± 7 days)**	Survival Assessments (± 7 days)**
							2 wkly	4 wkly	6 wkly	8 wkly	16 wkly				
		W0 (Day 1)	W2	W4	W6	W8	10,12, 14,16, etc.	12,16, 20,24, etc.	12,18, 24,30, etc.	16,24, 32,40 etc.	16,32 48, etc.				
			± 3 days*						± 7 days**						
Thyroid Function and Cortisol Level Tests[k]	X	X (-1 day)		X		X		X				X	X		
Pregnancy test[l]	X	X	X	X	X	X	X					X			
Tumour assessments[m]	X				X (+7 days)				Every 6 weeks (± 7 days) [except 1st scan + 7days only]						
Adverse events & Concomitant Medications	X	X	X	X	X	X	X					X	X	X	
Dispense durvalumab[n]		X (-3 days)	X (-3 days)	X (-3 days)	X (-3 days)	X (-3 days)	X (-3 days)								
Administer durvalumab[o]		X	X	X	X	X	X								
Smoking status[p]		X				X				X		X			

	Screening<br><br>Within 28 days of treatment (unless otherwise stated)	Treatment 10 mg/kg IV every 2 weeks										Discontinuation (+ 7 days)	28 day follow up[v] (+ 7 days)*	Months 2 & 3 post-discontinuation (± 7 days)**	Survival Assessments (± 7 days)**
							2 wkly	4 wkly	6 wkly	8 wkly	16 wkly				
		W0 (Day 1)	W2	W4	W6	W8	10,12, 14,16, etc.	12,16, 20,24, etc.	12,18, 24,30, etc.	16,24, 32,40 etc.	16,32 48, etc.				
			± 3 days*						± 7 days**						
Germline DNA sample[q]		X (-1 day)													
ctDNA samples[r]		X (-1 day)				X (- 2 days)				X (- 2 days)		X			
PBMC samples[s]		X (-1 day)	X (- 2 days)		X (- 2 days)							X			
Optional research biopsy[t]		X										X			
Survival status[u]															X

* Visit may occur ± 3 days of the planned visit date. Individual assessments may occur independently of the visit date where indicated in the table above

** Visit may occur ± 7 days of the planned visit date. Individual assessments may occur independently of the visit date where indicated in the table above

*** Visit may occur + 7 days of the plannet visit date. Individual assessments may occur independently of the visit date where indicated in the table above

a Prior to the start of any study specific procedures, each patient must provide signed informed consent.

b A mandatory fresh tumour biopsy sample for molecular testing should be collected at screening. Repeat molecular testing biopsies are run on the Next Generation Sequencing panel with germline blood DNA. These germline samples will have previously been collected at patient consent to SMP2. It is important to check that there is sufficient blood DNA remaining from this original germline sample, by checking if there is stored material with the Technology Hub and then at the Clinical Hub, so that it can be re-run on the panel with mandatory repeat biopsies for Matrix. If the sample quantity is insufficient, the patient should be consented again using SMP2 consent, and a further sample should be taken from the patient under the consent of SMP2.

Once part of the biopsy sample has been sent for repeat SMP2 testing, the remaining sample should be shipped to the HBRC for storage and subsequent PD-L1 testing.

See Section 6.4 Definition of sample adequate to submit for testing, and refer to the Laboratory Manual for sample processing instructions.

c Demography must be captured for all patients. Demographic data and other characteristics will include: date of birth, gender, race/ethnicity.

d A standard medical and surgical history will be obtained, including prior cancer treatment.

e Patients must not be registered unless all eligibility criteria (core and arm-specific) have been fully met.

f Physical examination includes general appearance, respiratory, cardiovascular, abdomen, skin, head and neck (including ears, eyes, nose and throat), lymph nodes, thyroid, musculo-skeletal (including spine and extremities), and neurological systems and should be performed at screening, every 2 weeks, at discontinuation and at 28-day follow up.

g Supine BP and pulse will be measured using a semi-automatic BP recording device with an appropriate cuff size, after the patient has rested for at least 5 minutes.

Patients will be monitored at screening, day 1 of each infusion and at discontinuation. Screening and discontinuation assessments will consist of a single measurement of blood pressure, pulse rate, weight and temperature. During and after the infusion, the same assessments of vital signs will take place but at the following times (based on a 60-minute infusion):

- At the beginning of the infusion (at 0 minutes)
- Every 30 minutes during the infusion (±5 minutes)
- At the end of the infusion (at 60 minutes ±5 minutes)
- Thirty and 60 minutes post the infusion (i.e., 90 and 120 minutes from the start of the infusion) (±5 minutes) for the first infusion only and then for subsequent infusions as clinically indicated.

If the infusion takes longer than 60 minutes then the vital signs assessments should follow the principles as described above or more frequently if clinically indicated.

Two or more BP readings should be taken at 2 minutes intervals and averaged. If the first 2 diastolic readings differ by more than 5 mmHg, then an additional reading should be obtained and averaged. After the first dose, subsequent doses (at dose levels of 10 mg/kg or less), the 1-hour observation period will not be required unless a subject experiences an infusion-related reaction.

h ECGs recorded during the screening period will be obtained in triplicate; ECGs recorded during the treatment phase will be single tracing. ECG to be performed at screening, every 16 weeks on treatment beginning at week 16, and at discontinuation. Twelve-lead ECGs will be obtained after the patient has been resting supine for at least 10 minutes prior to times indicated. All ECGs should be recorded with the patient in the same physical position. For each time point unless specifically stated above, three ECG recordings should be taken at a minimum of 5 minute intervals (all three ECGs must be collected within 30 minutes). A standardised ECG machine should be used and the patient should be examined using the same machine throughout the study if possible. After paper ECGs have been recorded, the Investigator or designated physician will review each of the timed 12-lead ECGs on each of the study days when they are collected and may refer to a local cardiologist if appropriate. A paper copy should be filed in the patient's medical records. If an abnormal ECG finding at screening is considered to be clinically significant by the Investigator, it should be reported as a concurrent condition. For all ECGs details of rhythm, ECG intervals (R-R, PR, QT and QRS) and an overall evaluation will be recorded. At screening, mean QTcF must be <470 msec. On Week 16, ECGs will be recorded within an hour prior to start of infusion and at least one time point 0 to 3 hours after the infusion.

i Samples to be collected at screening, week 0 (day 1), every 2 weeks during treatment, discontinuation, 28 day follow up, month 2 and month 3 follow up visits. Samples can be taken up to 2 days earlier than the actual visit date (where indicated).

Clinical chemistry, haematology and urinalysis tests to be performed at every visit and if clinically indicated.

Clinical chemistry: Albumin, AST, ALT, ALP, bilirubin (total), bicarbonate, calcium (total), cholesterol, GGT, creatinine, magnesium, sodium, urea nitrogen, glucose, uric acid, LDH, phosphate, chloride, amylase, lipase, total protein, triglycerides and potassium.

Tests for aspartate ALT, AST, ALP, and total bilirubin must be conducted concurrently and assessed concurrently.

All patients with an AST, ALT or bilirubin value (the latter ≥1.5 x ULN) at the time of the last dose of durvalumab should have a further liver chemistry profile (AST, ALT, bilirubin and ALP) performed 30 days (±7days) after permanent discontinuation of durvalumab.

Results for urea and electrolytes, full blood count and liver function tests must be available before commencing an infusion. Can be done within 72 hours of infusion. Screening test results obtained within 3 days of dosing may be used prior to Week 0 Day 1 infusion if Week 0 Day 1 results are not available prior to dosing.

Haematology: FBC, Prothrombin time, fibrinogen.

APTT and INR will be performed at screening only unless clinically indicated.

j Urinalysis to be performed at screening, every 4 weeks whilst on treatment and at discontinuation and at the 28 day follow up visit: Blood, protein, glucose, ketones, bilirubin, pH, colour & appearance and specific gravity.

Microscopy should be used as appropriate to investigate white blood cells and use the high power field for red blood cells.

k Thyroid functions tests (T3, T4 and TSH), and a cortisol level test to be performed at screening, every 4 weeks whilst on treatment, at discontinuation and at the 28 day follow up visit.

l Female patients of child-bearing potential only. A serum or urine pregnancy test is to be performed at screening, pre dose on week 0 (day 1), every 2 weeks whilst on treatment and at discontinuation.

m CT or MRI scan of head, chest and abdomen to be performed at screening. CT or MRI scans of chest and abdomen to be performed until discontinuation. Tumour assessments should be performed relative to the date of start of treatment (week 0 day 1) every 6 weeks for the first year, then every 12 weeks. Scans should be performed ± 7 days (except 1st scan + 7 days only). If brain metastases are identified at screening or if clinically indicated, head scanning will also be performed throughout treatment at the same time points. All scans to be reported using RECIST 1.1. **The imaging modality must be used consistently throughout the course of the trial for each patient.**

n Durvalumab must be dispensed within the IWRS Cenduit system. Refer to the Pharmacy Manual for further details. Durvalumab may be dispensed within the IWRS up to 3 days prior to the actual visit date.

o Week 0 (Day 1): Treatment must commence within 7 days of trial registration.

p Smoking status data will be collected through questions and CO monitoring **pre-dose** week 0 day 1 then every 8 weeks and at discontinuation.

q A whole blood germline DNA sample is to be collected **pre-dose** on week 0 day 1. If the sample is not collected at this timepoint, it should be collected at the next visit. Refer to the Laboratory Manual for sample processing guidelines.

r ctDNA samples to be collected **pre-dose** week 0 day 1 then every 8 weeks and at discontinuation. Refer to the Laboratory Manual for sample processing instructions. ctDNA samples should be taken **prior** to durvalumab infusion on treatment visits. Samples can be taken up to 2 days earlier than the actual visit date (where indicated).

s Peripheral blood mononuclear cells (PBMC) samples are to be collected **pre-dose** week 0 day 1, week 2, week 6 and at discontinuation. Samples can be taken up to 2 days earlier than the actual visit date (where indicated). Please note PBMC samples can only be collected and shipped Monday-Thursday. Refer to the Laboratory Manual for sample processing instructions.

t An optional fresh metastatic/recurrent tumour biopsy sample should be collected at discontinuation for patients who discontinue treatment for reasons other than disease progression (origin from either the primary tumour or site of metastasis). The discontinuation biopsy must be performed prior to commencing further anti-cancer therapy. A post-treatment biopsy will only be requested from patients with an objective response or stabilisation of disease (PR or CR), or 6 months on treatment with evidence of stabilisation (SD) for

patients who have previously progressed. The tumour tissue will be used to determine possible mechanisms of resistance to study treatment. Refer to the Laboratory Manual for sample processing instructions.

u Survival status will be collected every 12 weeks (± 7 days) post-permanent discontinuation of durvalumab until death.

v 28 day follow up visit should be carried out 28 days (+ 7 days) post-permanent discontinuation of durvalumab.

Table 91: Durvalumab - Schedule of Assessments: follow up in absence of PD.

Follow up for patients who have discontinued durvalumab due to toxicity in the absence of confirmed PD.

	**Time since last dose of durvalumab**	**Follow Up Investigations**			
	Day (± 2 days)	Months (± 7 days)			
	28	2	3	4	Post 4 months
Physical examination	X				
Vital signs inc. weight	X				
ECG	X				
Adverse events & Concomitant Medications	X	X	X		
ECOG performance status	X	X	X	X	
Haematology	X	X	X	X	
Clinical chemistry	X	X	X		
Urinalysis	X				
Thyroid Function Tests (TSH and T3 and T4)	X				
Tumour assessments[1]	(See footnote for frequency)				
ctDNA sample[2]	(See footnote for frequency)				
Survival assessment[3]		Every 12 weeks (±14 days)			

1 **For patients who have discontinued durvalumab for reasons other than progressive disease (e.g. due to toxicity)**, tumour assessments should continue to be performed relative to the date of start of treatment (week 0) as follows: every 6 weeks for the first year, then every 12 weeks, until confirmed objective progression by RECIST 1.1, or the patient starts a new anti-cancer therapy (unless the patient withdraws consent to do so).

Scans should be of the chest and abdomen, and only include the head where brain metastases are identified at screening, or if clinically indicated.

2 ctDNA samples will be collected in follow up for patients who discontinue treatment for reasons other than progressive disease (e.g. toxicity). These samples should be performed at the same visit as follow up CT or MRI scans until disease progression or the patient starts a new anti-cancer therapy (unless the patient withdraws consent to do so). Samples should be collected on a 6-weekly basis for the first year relative to the start date of treatment then every 12 weeks.

3 Survival status will be collected every 12 weeks (± 7 days) post-permanent discontinuation of durvalumab until death.

31.3.3 Toxicity Profile

Detailed information on the non-clinical toxicity studies with durvalumab in cynomolgus monkeys is available in the IB.

31.3.3.1 Adverse Events of Special Interest

31.3.3.2 Adverse events of special interest (AESI) can be found in Arm J Toxicity Profile Section 30.3.3Immune-Mediated Adverse Events

Immune-mediated adverse event (imAE) details can be found in Arm J Toxicity Profile Section 30.3.3.

If the local Investigator has any questions in regards to an adverse event (AE) being an irAE, the local Investigator should promptly contact the Chief Investigator.

31.3.3.3 Adverse Events Reported with Durvalumab

31.3.3.4 Expected Adverse Events for Durvalumab can be found in Arm J Toxicity Profile Section 30.3.3. Overdose

An overdose is defined as a subject receiving a dose of durvalumab in excess of that specified in the Investigator's Brochure, unless otherwise specified in this protocol.
Any overdose of a study subject with durvalumab, with or without associated AEs/SAEs, is required to be reported within 24 hours of knowledge of the event to the National Lung Matrix Trial Office. If the overdose results in an AE, the AE must also be recorded as an AE using the eRDC. Overdose does not automatically make an AE serious, but if the consequences of the overdose are serious, for example death or hospitalisation, the event is serious and must be recorded and reported as an SAE (see section 11.2.1.2). There is currently no specific treatment in the event of an overdose of durvalumab.

The Investigator will use clinical judgment to treat any overdose.

If the overdose does not qualify as an SAE, please contact the National Lung Matrix Trial Office within 24 hours of knowledge of the event by either phone or email.

31.3.4 Dosing Modifications & Toxicity Management

31.3.4.1 Doses and Treatment Regimens

Patients receiving durvalumab will commence treatment (10 mg/kg IV) on Day 1 and continue every 2 weeks (Q2W) until confirmed disease progression, unless there is unacceptable toxicity, withdrawal of consent, or another discontinuation criterion is met. Patients who meet RECIST criteria for progressive disease (PD) may be continued on trial treatment if the treatment is tolerable and the Investigator believes it to be of clinical benefit; see Section 9.3.

31.3.4.2 Monitoring of dose administration

Subjects will be monitored during and after the infusion with assessment of vital signs at the times specified in the Schedule of Assessments. Monitor subjects for signs and symptoms of infusion-related reactions (e.g., fever and/or shaking chills, flushing and/or itching, alterations in heart rate and blood pressure, dyspnea or chest discomfort, skin rashes etc.) and anaphylaxis (e.g., generalized urticaria, angioedema, wheezing, hypotension, tachycardia, etc.)

In the event of a ≤ Grade 2 infusion-related reaction the infusion rate of durvalumab may be decreased by 50% or temporarily interrupted until resolution of the event (up to 4 hours).

In the event of a Grade 2 infusion-related reaction, the infusion rate of durvalumab may be decreased by 50% or temporarily interrupted until resolution of the event (up to 4 hours) and re-initiated at 50% of the initial rate until completion of the infusion. For patients with a Grade 2 infusion related reaction, subsequent infusions may be administered at 50% of the initial rate. Acetaminophen and/orantihistamines may be administered per institutional standard at the discretion of the local Investigator. Consider premedication per institutional standard prior to subsequent doses

If the infusion related reaction is ≥ Grade 3 or higher in severity, treatment with durvalumab will be permanently discontinued. Manage severe infusion-related reactions per institutional standards (e.g., intramuscular epinephrine, followed by IV diphenhydramine and ranitidine, and IV glucocorticoid).

As with any antibody, allergic reactions to dose administration are possible. Appropriate drugs and medical equipment to treat acute anaphylactic reactions must be immediately available, and study personnel must be trained to recognize and treat anaphylaxis. The study site must have immediate access to emergency resuscitation teams and equipment in addition to the ability to admit patients to an intensive care unit if necessary.

31.3.4.3 Dose Modifications

For AEs that are considered at least partly due to administration of durvalumab the following dose adjustment guidance may be applied:

- Treat each of the toxicities with maximum supportive care (including holding the agent suspected of causing the toxicity where required).
- If the symptoms promptly resolve with supportive care, consideration should be given to continuing the same dose of durvalumab along with appropriate continuing supportive care. If medically appropriate, dose modifications are permitted for durvalumab.

All dose modifications should be documented in the CRF with clear reasoning and documentation of the approach taken.

In addition, there are certain circumstances in which durvalumab should be permanently discontinued.

Following the first dose of durvalumab, subsequent administration of durvalumab can be modified based on toxicities observed. Dose reductions are not permitted.

Dose modifications will not be required for AEs that are clearly not attributed to durvalumab (such as an accident) or for laboratory abnormalities that are not deemed to be clinically significant. Dosing may continue despite concurrent vitiligo of any AE grade.

Based on the mechanism of action of durvalumab leading to T-cell activation and proliferation, there is the possibility of observing irAEs during the conduct of this study. Potential irAEs may

be similar to those seen with the use of ipilimumab including immune mediated enterocolitis, dermatitis, hepatitis, and endocrinopathies (Brahmer *et al.* 2010, Hodi *et al.* 2010). Patients should be monitored for signs and symptoms of irAEs. In the absence of an alternate aetiology (e.g., infection or PD) signs or symptoms of enterocolitis, dermatitis, hepatitis, and endocrinopathy should be considered to be immune-related.

Dose modification recommendations and toxicity management guidelines for immune-mediated reactions, for infusion-related reactions, and for non-immune-mediated reactions are detailed in Arm J Section 30.3.4 Dose Modifications & Toxicity Management in the following tables:
Table 84: General Considerations Regarding Immune-Mediated Reactions
Table 85: Durvalumab Specific Immune-Mediated Reactions
Table 86: Durvalumab Other Immune-Mediated Reactions
Table 87: Durvalumab Infusion-Related Reactions
Table 88: Durvalumab Non - Immune-Mediated Reactions.

REFERENCES

- Berger R, Rotem-Yehudar R, Slama G *et al.* (2008). Phase I safety and pharmacokinetic study of CT-011, a humanized antibody interacting with PD-1, in patients with advanced hematologic malignancies. *Clin Cancer Res* **14(10):**3044-51.
- Blank C, Kuball J, Voelkl S, *et al.* (2006). Blockade of PD-L1 (B7-H1) augments human tumour-specific T cell responses *in vitro*. *Int J Cancer* **119(2):**317-27.
- Brahmer JR, Drake CG, Wollner I *et al.* (2010). Phase I study of single-agent anti-programmed death-1 (MDX-1106) in refractory solid tumors: safety, clinical activity, pharmacodynamics, and immunologic correlates. *J Clin Oncol* **28(19):**3167-75.
- Brahmer JR, Tykodi SS, Chow LQ, *et al.* (2012). Safety and activity of anti-PD-L1 antibody in patients with advanced cancer. *N Engl J Med* **(26):**2455-65.
- D'Addario G, Früh M, Reck M *et al* (2010). ESMO Guidelines Working Group. Metastatic non-small-cell lung cancer: ESMO Clinical Practice Guidelines for diagnosis, treatment and follow-up. *Ann Oncol* **21**(Suppl 5):v116-9.
- Dong H, Zhu G, Tamada K *et al.* (2004). B7-H1 determines accumulation and deletion of intrahepatic CD8(+) T lymphocytes. *Immunity* **20(3):**327 36.
- Fairman D, Narwal R, Liang M, Robbins P, Schneider A, Chavez C, Lu H, Pak M, Blake-Haskins A, Vasselli J, Ibrahim R, and Lorin Roskos. Pharmacokinetics of MEDI4736, a Fully Human Anti-PDL1 Monoclonal Antibody, in Patients with Advanced Solid Tumours. Presented at American Society of Clinical Oncology, 2014. Poster.
- FDA Guidance for Industry (issued July 2009) 'Drug-induced liver injury: Premarketing clinical evaluation'. Available from URL: http://www.fda.gov/downloads/Drugs/GuidanceComplianceRegulatoryInformation/Guidances/UCM174090.pdf. Accessed 10 December 2013.
- Fife BT, Bluestone JA (2008). Control of peripheral T-cell tolerance and autoimmunity via the CTLA-4 and PD-1 pathways. *Immunol Rev* **224:**166-82.
- Hanna N, Shepherd FA, Fossella FV *et al.* (2004). Randomized phase III trial of pemetrexed versus docetaxel in patients with non-small-cell lung cancer previously treated with chemotherapy. *J Clin Oncol* **22(9):**1589-97.
- Hirano F, Kaneko K, Tamura H *et al.* (2005) Blockade of B7-H1 and PD-1 by monoclonal antibodies potentiates cancer therapeutic immunity. *Cancer Res* **65(3):**1089-96.
- Hodi FS, O'Day SJ, McDermott DF *et al.* (2010). Improved survival with ipilimumab in patients with metastatic melanoma [published erratum appears in N Engl J Med 2010 Sep 23;363(13):1290*]. N Engl J Med* **363(8):**711-23.
- Horn L, Herbst RS, Spigel D *et al.* (2013). MO18.01 An analysis of the relationship of clinical activity to baseline EGFR status, PDl1 expression and prior treatment history in patients with non-small cell lung cancer (NSCLC) folloeing PDL1 blockade with MPDL3280A (anti-PDL1). *J Thorac Oncol* 8: Suppl 2; Abstract
- Iwai Y, Ishida M, Tanaka Y (2002). Involvement of PD-L1 on tumor cells in the escape from host immune system and tumor immunotherapy by PD-L1 blockade. *Proc Natl Acad Sci USA* **99(19):**12293-7.
- Kalialis LV, Drzewiecki KT, Klyver H (2009). Spontaneous regression of metastases from melanoma: review of the literature. *Melanoma Res* **19(5):**175-82.
- Keir ME, Butte MJ, Freeman GJ *et al.* (2008). PD-1 and its ligands in tolerance and immunity. *Annu Rev Immunol* **26:**677-704.
- Latchman YE, Liang SC, Wu Y *et al.* (2004). PD-L1-deficient mice show that PD-L1 on T cells, antigen-presenting cells, and host tissues negatively regulates T cells. *Proc Natl Acad Sci USA* **101(29):**10691-6.
- Lipson EJ, Drake CG (2011). Ipilimumab: an anti-CTLA-4 antibody for metastatic melanoma. *Clin Cancer Res* **17(22):**6958-62.

- Mu CY, Huang JA, Chen Y *et al.* (2011). High expression of PD-L1 in lung cancer may contribute to poor prognosis and tumor cells immune escape through suppressing tumor infiltrating dendritic cells maturation. *Med Oncol* **28(3):**682-8.
- Nishimura H, Minato N, Nakano T *et al.* (2009). Immunological studies on PD-1 deficient mice: implication of PD-1 as a negative regulator for B cell responses. *Int Immunol* **10(10):**1563-72.
- Okudaira K, Hokari R, Tsuzuki Y *et al.* (2009). Blockade of B7-H1 or B7-DC induces an anti-tumor effect in a mouse pancreatic cancer model. *Int J Oncol* **35(4):**741-9.
- Pagès F, Galon J, Dieu-Nosjean MC (2010). Immune infiltration in human tumors: a prognostic factor that should not be ignored. *Oncogene* **29(8):**1093-102.
- Park JJ, Omiya R, Matsumura Y *et al.* (2010). B7-H1/CD80 interaction is required for the induction and maintenance of peripheral T-cell tolerance. *Blood* **116(8):**1291-8.
- Topalian SL, Hodi FS, Brahmer JR *et al.* (2012) Safety, activity, and immune correlates of anti-PD-1 antibody in cancer. *N Engl J Med* **366:**2443-54
- Weber JS, Kähler KC, Hauschild A (2012). Management of immune-related adverse events and kinetics of response with ipilimumab. *J Clin Oncol* **30(21):**2691-7.
- Wolchok JD, Kluger H, Callahan MK et al. (2013), Nivolumab plus Ipilimumab in advanced melanoma," *N Engl J Med*, **369(2):**122–33.
- Wolchok JD, Hoos A, O'Day S *et al.* (2009). Guidelines for the Evaluation of Immune Therapy Activity in Solid Tumors: Immune-Related Response Criteria. *Clin Cancer Res* **15:** 7412.Swann JB & Smyth MJ (2007). Immune Surveillance of Tumours. *J Clin Invest* **117(5):**1137-46.
- Yervoy™ [prescribing information]. Princeton, NJ, IN: Bristol-Myers Squibb Company; 2011 [cited 2011 March]. Available from: http://www.accessdata.fda.gov/drugsatfda_docs/label/2011/125377s0000lbl.pdf. Accessed 31 October 2013.
- Zhang C, Wu S, Xue X *et al.* (2008). Anti-tumor immunotherapy by blockade of the PD-1/PD-L1 pathway with recombinant human PD-1-IgV. *Cytotherapy* **10(7):**711-9.
- Zou W, Chen L. (2008). Inhibitory B7-family molecules in the tumour microenvironment. *Nat Rev Immunol* **8(6):**467-77.

32 APPENDIX 1: RESPONSE EVALUATION CRITERIA IN SOLID TUMOURS VERSION 1.1

The following contains excerpts from the RECIST version 1.1 plus study specific instructions. A free copy of the revised guidelines is available from http://www.eortc.be/recist/RECIST_EORTC_NCI_AACR_OCtober_2008.pdf (Eisenhauer *et al.*, 2009).

Measurability of Tumour Lesions at Baseline

Only patients with measurable disease at baseline should be included. Measurable disease is defined by the presence of at least one measurable lesion. At baseline, tumour lesions will be categorised as follows:

- Measurable
- Non-measurable

Measurable lesions are those that can be accurately measured in at least one dimension (longest diameter to be recorded) with a minimum size of 10 mm by CT scan (CT scan slice thickness no greater than 5 mm), 10 mm caliper measurement by clinical exam (lesions which cannot be accurately measured with callipers should be recorded as non-measurable) and 20 mm by chest X-ray. For malignant lymph nodes to be considered pathologically enlarged and measurable, a lymph node must be ≥15 mm in short axis when assessed by a CT scan (at baseline and during treatment, only the short axis will be measured and followed).

Non-measurable lesions are all other lesions, including small lesions (longest diameter <10 mm or pathological lymph nodes with ≥10 to <15 mm short axis) and truly non-measurable lesions.

Lesions considered to be truly non-measurable include the following: bone lesions, leptomeningeal disease, ascites, pleural/pericardial effusion, inflammatory breast disease, lymphangitis cutis/pulmonis, abdominal masses that are not confirmed and followed by imaging techniques, and cystic lesions.

Tumour lesions that are situated in a previously irradiated area are not considered measurable.

The term "evaluable" in reference to measurability is not recommended and will not be used because it does not provide additional meaning or accuracy.

All measurements should be recorded in metric notation using callipers (or a ruler) if clinically assessed. All baseline evaluations should be performed as closely as possible to the beginning of treatment and never more than 4 weeks before the beginning of treatment.

Specifications by Methods of Measurements

The same method of assessment and the same technique should be used to characterise each identified and reported lesions at baseline, during treatment and at the post-treatment assessment. Image-based evaluation is preferred to evaluation by clinical examination when both methods have been used to assess the anti-tumour effect of a treatment. CT is the best currently available and reproducible method for measuring target lesions selected for response assessment. Investigators should utilize the best available CT imaging technique available to them for determining response and PFS of patients participating in the National Lung Matrix Trial.

Tumour Response Evaluation

Baseline Documentation of "Target" and "Non-target" Lesions
All measurable lesions up to a maximum of 2 lesions per organ and 5 lesions in total, representative of all involved organs, should be identified as “target” lesions and recorded and measured at baseline.

Target lesions should be selected on the basis of their size (those with the longest diameter) and their suitability for accurate, reproducible, repeated measurements.

A sum of the longest diameter (LD) for all target lesions will be calculated and reported as the baseline sum LD. The baseline sum LD will be used as the reference by which to characterise the objective tumour response.

All other lesions (or sites of disease) should be identified as “non-target” lesions and should also be recorded at baseline. Measurements of these lesions are not required but these lesions should be followed as ‘present’, ‘absent’ or in rare cases ‘unequivocal progression’ and recorded.

Response Criteria

A. Evaluation of Target Lesions

Response Category	Description
Complete Response (CR)	Disappearance of all target lesions
Partial Response (PR)	At least a 30% decrease in the sum of the LD of target lesions, taking as reference the baseline sum LD
Progressive Disease (PD)	At least a 20% increase in the sum of the LD of target lesions, taking as reference the smallest sum LD recordedon study (this includes the baseline sum if that is the smallest on study). In addition to this, the sum must also demonstrate an absolute increase of at least 5 mm.
Stable Disease (SD)	Neither sufficient shrinkage to qualify for PR nor sufficient increase to qualify for PD, taking as reference the smallest sum LD whilst on study.

B. Evaluation of Non-target Lesions

Response Category	Description
Complete Response (CR)	Disappearance of all non-target lesions
Incomplete Response/ Stable Disease (SD)	Persistence of one or more non-target lesion(s)
Progressive Disease (PD)	Appearance of one or more new lesions and/or unequivocal progression of existing non-target lesions [1]

1. To achieve “unequivocal progression” on the basis of the non-target disease, there must be an overall level of substantial worsening in non-target disease such that, even in presence of SD or PR

in target disease, the overall tumour burden has increased sufficiently to merit discontinuation of therapy.

C. Evaluation of New Lesions

Response Category	Description
**Progressive Disease (PD)**	The appearance of one or more lesion is also considered progression.

D. Overall Responses for all Possible Combinations of Tumour Responses in Target and Non-target Lesions With or Without the Appearance of New Lesions

Target Lesions	Non-target Lesions	New Lesions	Overall Response
Complete response (CR)	CR	No	CR
Complete response (CR)	Non-CR/non-PD	No	PR
Complete response (CR)	Not evaluated	No	PR
Partial response (PR)	Non-PD	No	PR
Stable disease (SD)	Non-PD	No	SD
Not all evaluated	Non-PD	No	Not evaluable (NE)
Progressive disease (PD)	Any	Yes or no	PD
Any	PD	Yes or no	PD
Any	Any	Yes	PD

Patients with a global deterioration of health status requiring discontinuation of treatment without objective evidence of disease progression at that time should be classified as having "symptomatic deterioration." Every effort should be made to document the objective disease progression, even after discontinuation of treatment.

E. Evaluation of Best Overall Response

The best overall response is the best response recorded from the start of treatment until disease progression In general, the patient's best response assignment will depend on the achievement of both measurement and confirmation criteria.

Frequency of Tumour Re-evaluations

For the National Lung Matrix Trial, clinical response rate, disease control rate (CR+PR+SD), PFS and duration of response will be evaluated radiologically by a CT scan of the head, chest

and abdomen and other clinically relevant areas at baseline, and by a CT scan of the chest and abdomen at 6 weekly intervals relative to the date of start of treatment, reducing to 12 weekly intervals after the 1st year and continuing until disease progression.. The head will only be scanned throughout treatment if there is evidence of brain metastases at baseline, or if clinically indicated.

For patients who discontinue treatment prior to progression CT scans should, where possible, continue to be performed in accordance with the relevant schedule of assessments until disease progression.

33 APPENDIX 2: DEFINITION OF SITES INVOLVED IN SMP2 AND THE NATIONAL LUNG MATRIX TRIAL

SMP2 Feeder Hospital

Hospital with R&D approval to consent patients to pre-screening and send diagnostic samples into their paired SMP2 Clinical Hub under SMP2 Governance.

SMP2 Clinical Hub

Experimental Cancer Medicine Centre where patients are consented to pre-screening under SMP2 Governance, and diagnostic samples are also received for SMP2 feeder hospitals. The SMP2 clinical hubs send samples from local patients and feeder hospitals to their paired Technology Hub.

Technology Hub

Laboratory that performs the NGS testing for both SMP2 and the National Lung Matrix Trial. All SMP2 clinical hubs are paired with one of 3 laboratories – Birmingham, Cardiff and ICR.

National Lung Matrix Trial Participating Site/Clinical Hub

Hospital treating patients in the National Lung Matrix Trial and performing all other protocol defined duties. These may be either SMP2 Clinical Hubs or SMP2 Feeder Hospitals.

National Lung Matrix Trial Feeder Site

SMP2 Feeder Hospital that have R&D approval to perform repeat molecular testing biopsies for the National Lung Matrix Trial, and perform the screening registration process in order to obtain a slot reservation with the National Lung Matrix Trial Office.

34 APPENDIX 3: COMMON TOXICITY CRITERIA GRADINGS

Toxicities will be recorded according to the Common Terminology Criteria for Adverse Events (CTCAE), version 4.0. The full CTCAE document is available on the National Cancer Institute (NCI) website, the following address was correct when this version of the protocol was approved:
http://ctep.cancer.gov/protocolDevelopment/electronic_applications/ctc.htm

35 APPENDIX 4: COCKCROFT GAULT FORMULA – CREATININE CLEARANCE

Cockcroft and Gault creatinine clearance (CrCl):

$$\text{CrCl (mL/min)} = \frac{\text{N x [140-age (years)] x weight* (kg)}}{\text{Serum creatinine (micromol/L)}}$$

(Where N = 1.23 males, 1.04 females)

36 APPENDIX 5: INCIDENTAL GENETIC FINDINGS POLICY

In the National Lung Matrix Trial plasma and whole blood samples will be genetically sequenced by various sequencing methods to detect circulating tumour DNA (ctDNA). The aim is to identify specific tumour mutations and compare these with germline. The aim is not to identify variants in germline and these will be excluded at an early stage of analysis if possible. However there is a small possibility that some germline mutations will be identified, and that these mutations will have implications for the patient and family risk of suffering from a disease.

In the event that a germline mutation is found, the Laboratory Research Team will ask the National Lung Matrix Trial Office to review the Informed Consent Form signed by the patient upon entry to the trial. If the patient in question has consented to be informed of the findings, the relevant Principal Investigator will be notified and will discuss the results with the NHS Clinical Genetics Team. During a routine visit the affected patient will be offered referral to the NHS Genetic Services if all of the following points are applicable:

- If the mutation is known to be associated with certain cancer risk or response to therapy. Variants of unknown significance or moderate risk genes (those without suficient evidence to alter management) will not be reported back to the patient.
- If findings are in relation to genes under investigation (the anaylsis and interpretation of the whole genome of the germline will not be possible, as the investigation is focused on certain regions of certain genes).
- Since some of the analysis will be performed years after collection, returning findings will only be considered if the patient is still alive.

The National Lung Matrix Trial Management Group will review any issues relating to incidental genetic findings and amend this policy as necessary.

This policy is applicable to all National Lung Matrix Trial participating sites.

Disclosure with Insurance Companies in the United Kingdom:
The Concordat and Moratorium allows patients who have taken a predicitive genetic test to obtain significant levels of insurance cover without disclosing the results of that predictive genetic test. This agreement between the UK Government and the Insurance Industry was established from 2001 until 2017, and is reviewed every 3 years. A new review was held in 2014.

For further information:

https://www.gov.uk/government/uploads/system/uploads/attachment_data/file/216821/Concordat-and-Moratorium-on-Genetics-and-Insurance-20111.pdf

www.geneticalliance.org.uk/insurance.htm

37 APPENDIX 6: DEFINITION OF ADVERSE EVENTS

Adverse Event
Any untoward medical occurrence in a patient or clinical trial subject administered a medicinal product and which does not necessarily have a causal relationship with this treatment.

Comment:
An AE can therefore be any unfavourable and unintended sign (including abnormal laboratory findings), symptom or disease temporally associated with the use of an investigational medicinal product, whether or not related to the investigational medicinal product.

Adverse Reaction
All untoward and unintended responses to an IMP related to any dose administered.

Comment:
An AE judged by either the reporting Investigator or the National Lung Matrix Trial Office as having causal relationship to the IMP qualifies as an AR. The expression reasonable causal relationship means to convey in general that there is evidence or argument to suggest a causal relationship.

Serious Adverse Event
Any untoward medical occurrence or effect that at any dose:

a) Results in death (unrelated to original cancer)
b) Is life-threatening*
c) Requires hospitalisation** or prolongation of existing inpatients' hospitalisation
d) Results in persistent or significant disability or incapacity
e) Is a congenital anomaly/birth defect
f) Or is otherwise considered medically significant by the Investigator***
g) For the purposes of the National Lung Matrix Trial, Hy's law cases, and potential sight-threatening and severe vision loss adverse events (Arm D patients only), are considered medically significant and should be reported as an SAE.****

Comments:
The term severe is often used to describe the intensity (severity) of a specific event. This is not the same as serious, which is based on patients/event outcome or action criteria.
*Life threatening in the definition of an SAE refers to an event in which the patient was at risk of death at the time of the event; it does not refer to an event that hypothetically might have caused death if it were more severe.
**Hospitalisation is defined as an unplanned, formal inpatient admission, even if the hospitalisation is a precautionary measure for continued observation. Thus, hospitalisation for protocol treatment (e.g. line insertion), elective procedures (unless brought forward because of worsening symptoms) or for social reasons (e.g. respite care) are not regarded as an SAE.
***Medical judgement should be exercised in deciding whether an AE is serious in other situations. Important AEs that are not immediately life threatening or do not result in death or hospitalisation but may jeopardise the subject or may require intervention to prevent one of the other outcomes listed in the definition above, should be considered serious.
****Refer to Sections 11.1.2.2 and 11.1.2.3 for further details.

Serious Adverse Reaction
An Adverse Reaction which also meets the definition of a Serious Adverse Event.

Suspected Unexpected Serious Adverse Reaction
A SAR that is unexpected i.e. the nature, or severity of the event is not consistent with the applicable product information.
A SUSAR should meet the definition of an AR, UAR and SAR.

Unexpected Adverse Reaction
An AR, the nature or severity of which is not consistent with the applicable product information (e.g. IB for an unapproved IMP or (compendium of) Summary of Product Characteristics (SPC) for a licensed product).

When the outcome of an AR is not consistent with the applicable product information the AR should be considered unexpected.

38 APPENDIX 7: WMA DECLARATION OF HELSINKI

WORLD MEDICAL ASSOCIATION DECLARATION OF HELSINKI

Recommendations guiding physicians in biomedical research involving human subjects

Adopted by the 18th World Medical Assembly
Helsinki, Finland, June 1964
and amended by the
29th World Medical Assembly, Tokyo, Japan, October 1975
35th World Medical Assembly, Venice, Italy, October 1983
41st World Medical Assembly, Hong Kong, September 1989
and the
48th General Assembly, Somerset West, Republic of South Africa, October 1996

INTRODUCTION

It is the mission of the physician to safeguard the health of the people. His or her knowledge and conscience are dedicated to the fulfilment of this mission.
The Declaration of Geneva of the World Medical Association binds the physician with the words, "The Health of my patient will be my first consideration," and the International Code of Medical Ethics declares that, "A physician shall act only in the patient's interest when providing medical care which might have the effect of weakening the physical and mental condition of the patient."
The purpose of biomedical research involving human subjects must be to improve diagnostic, therapeutic and prophylactic procedures and the understanding of the aetiology and pathogenesis of disease.
In current medical practice most diagnostic, therapeutic or prophylactic procedures involve hazards. This applies especially to biomedical research.
Medical progress is based on research which ultimately must rest in part on experimentation involving human subjects.
In the field of biomedical research a fundamental distinction must be recognized between medical research in which the aim is essentially diagnostic or therapeutic for a patient, and medical research, the essential object of which is purely scientific and without implying direct diagnostic or therapeutic value to the person subjected to the research.
Special caution must be exercised in the conduct of research which may affect the environment, and the welfare of animals used for research must be respected.
Because it is essential that the results of laboratory experiments be applied to human beings to further scientific knowledge and to help suffering humanity, the World Medical Association has prepared the following recommendations as a guide to every physician in biomedical research involving human subjects. They should be kept under review in the future. It must be stressed that the standards as drafted are only a guide to physicians all over the world. Physicians are not relieved from criminal, civil and ethical responsibilities under the laws of their own countries.

I. BASIC PRINCIPLES

1. Biomedical research involving human subjects must conform to generally accepted scientific principles and should be based on adequately performed laboratory and animal experimentation and on a thorough knowledge of the scientific literature.

2. The design and performance of each experimental procedure involving human subjects should be clearly formulated in an experimental protocol which should be transmitted for consideration, comment and guidance to a specially appointed committee independent of

the Investigator and the sponsor provided that this independent committee is in conformity with the laws and regulations of the country in which the research experiment is performed.

3. Biomedical research involving human subjects should be conducted only by scientifically qualified persons and under the supervision of a clinically competent medical person. The responsibility for the human subject must always rest with a medically qualified person and never rest on the subject of the research, even though the subject has given his or her consent.

4. Biomedical research involving human subjects cannot legitimately be carried out unless the importance of the objective is in proportion to the inherent risk to the subject.

5. Every biomedical research project involving human subjects should be preceded by careful assessment of predictable risks in comparison with foreseeable benefits to the subject or to others. Concern for the interests of the subject must always prevail over the interests of science and society.

6. The right of the research subject to safeguard his or her integrity must always be respected. Every precaution should be taken to respect the privacy of the subject and to minimize the impact of the study on the subject's physical and mental integrity and on the personality of the subject.

7. Physicians should abstain from engaging in research projects involving human subjects unless they are satisfied that the hazards involved are believed to be predictable. Physicians should cease any investigation if the hazards are found to outweigh the potential benefits.

8. In publication of the results of his or her research, the physician is obliged to preserve the accuracy of the results. Reports of experimentation not in accordance with the principles laid down in this Declaration should not be accepted for publication.

9. In any research on human beings, each potential subject must be adequately informed of the aims, methods, anticipated benefits and potential hazards of the study and the discomfort it may entail. He or she should be informed that he or she is at liberty to abstain from participation in the study and that he or she is free to withdraw his or her consent to participation at any time. The physician should then obtain the subject's freely-given informed consent, preferably in writing.

10. When obtaining informed consent for the research project the physician should be particularly cautious if the subject is in a dependent relationship to him or her or may consent under duress. In that case the informed consent should be obtained by a physician who is not engaged in the investigation and who is completely independent of this official relationship.

11. In case of legal incompetence, informed consent should be obtained from the legal guardian in accordance with national legislation. Where physical or mental incapacity makes it impossible to obtain informed consent, or when the subject is a minor, permission from the responsible relative replaces that of the subject in accordance with national legislation. Whenever the minor child is in fact able to give consent, the minor's consent must be obtained in addition to the consent of the minor's legal guardian.

12. The research protocol should always contain a statement of the ethical considerations involved and should indicate that the principles enunciated in the present Declaration are complied with.

II. MEDICAL RESEARCH COMBINED WITH PROFESSIONAL CARE (Clinical Research)

1. In the treatment of the sick person, the physician must be free to use a new diagnostic and therapeutic measure, if in his or her judgement it offers hope of saving life, re-establishing health or alleviating suffering.

2. The potential benefits, hazards and discomfort of a new method should be weighed against the advantages of the best current diagnostic and therapeutic methods.

3. In any medical study, every patient - including those of a control group, if any - should be assured of the best proven diagnostic and therapeutic method. This does not exclude the use of inert placebo in studies where no proven diagnostic or therapeutic method exists.

4. The refusal of the patient to participate in a study must never interfere with the physician-patient relationship.

5. If the physician considers it essential not to obtain informed consent, the specific reasons for this proposal should be stated in the experimental protocol for transmission to the independent committee (I, 2).

6. The physician can combine medical research with professional care, the objective being the acquisition of new medical knowledge, only to the extent that medical research is justified by its potential diagnostic or therapeutic value for the patient.

III. NON-THERAPEUTIC BIOMEDICAL RESEARCH INVOLVING HUMAN SUBJECTS (Non-Clinical Biomedical Research)

1. In the purely scientific application of medical research carried out on a human being, it is the duty of the physician to remain the protector of the life and health of that person on whom biomedical research is being carried out.

2. The subject should be volunteers - either healthy persons or patients for whom the experimental design is not related to the patient's illness.

3. The Investigator or the investigating team should discontinue the research if in his/her or their judgement it may, if continued, be harmful to the individual.

4. In research on man, the interest of science and society should never take precedence over considerations related to the wellbeing of the subject.

39 APPENDIX 8: EASTERN COOPERATIVE ONCOLOGY GROUP PERFORMANCE STATUS CRITERIA

0	Fully active, able to carry on all pre-disease performance without restriction
1	Restricted in physically strenuous activity but ambulatory and able to carry out work of a light or sedentary nature, e.g., light house work, office work
2	Ambulatory and capable of all selfcare but unable to carry out any work activities. Up and about more than 50% of waking hours
3	Capable of only limited selfcare, confined to bed or chair more than 50% of waking hours
4	Completely disabled. Cannot carry on any selfcare. Totally confined to bed or chair
5	Dead

As published in Am. J. Clin. Oncol: Oken, M.M., Creech, R.H., Tormey, D.C., Horton, J., Davis, T.E., McFadden, E.T., Carbone, P.P.: Toxicity And Response Criteria Of The Eastern Cooperative Oncology Group. Am J Clin Oncol 5:649-655, 1982.

40 APPENDIX 9 CREDIBLE MEDS LIST OF DRUGS THAT PROLONG QT INTERVAL

This list was exported from https://www.crediblemeds.org on 6th December 2019.

It is important to note that this list is a guide – the database will change with time and therefore needs to be checked in real time when screening and registering a patient, and throughout their treatment. Please contact the National Lung Matrix Trial Office for clarification regarding any drugs that appear on the database but not in the list below.

Arm A:

Drugs known to cause QT interval prolongation are prohibited at registration and during the active treatment phase for patients receiving AZD4547 (Arm A). The Arm-specific Exclusion Critiera should be referred to for the required washout period for patients taking prohibited concomitant medications prior to commencement of trial treatment.

All concomitant medications listed on https://www.crediblemeds.org under the following risk category will exclude patients from entering the trial:

- **Known Risk of TdP**

In addition, **a number of permitted drugs with a possible TdP risk have an associated minimum treatment period on medication prior to commencing AZD4547 (see Section 22.2.3.1.2).**

Arms B, F and G:

Drugs known to cause QT interval prolongation are prohibited at registration and during the active treatment phase for patients receiving either vistusertib (Arm B), AZD5363 (Arm F) or osimertinib (Arm G). The Arm-specific Exclusion Critiera should be referred to for the required washout period for patients taking prohibited concomitant medications prior to commencement of trial treatment.

All concomitant medications listed on https://www.crediblemeds.org under the following risk category will exclude patients from entering the trial:

- **Known Risk of TdP**

Arms C and D:

Drugs known to cause QT interval prolongation, or with any risk of prolonging the QT interval, are prohibited at registration and during the active treatment phase for patients receiving either palbociclib (Arm C) or crizotinib (Arm D). The Arm-specific Exclusion Critiera should be referred to for the required washout period for patients taking prohibited concomitant medications prior to commencement of trial treatment.

All concomitant medications listed on https://www.crediblemeds.org under the following 3 categories will exclude patients from entering the trial, unless an exception applies:

- **Known Risk of TdP**
- **Possible Risk of TdP**
- **Drugs to Avoid in Congenital Long QT**

The following exceptions may apply:

- The category '**Drugs to Avoid in Congenital Long QT**' includes drugs that should be avoided in patients with diagnosed or suspected congenital long QT syndrome.

Patients without any evidence of these conditions may continue taking drugs allocated to this category alone and would be therefore eligible for inclusion in the trial.

- <u>**Important note**</u>: '**Drugs to Avoid in Congenital Long QT**' category may also be assigned to drugs in parallel with either of the following 2 categories '**Known risk of TdP**' and '**Possible risk of TdP**'; these concomitant medications are still prohibited for all trial patients and therefore patients would be ineligible for inclusion in the trial.

- Patients receiving treatment with a concomitant medication within the risk category **'Conditional Risk of TdP'** are **not** excluded from trial entry into Arms C and D, regardless of whether or not they are also assigned the 'Drugs to Avoid in Congenital Long QT' category. (Medications within this category are not listed below but will still appear on https://www.crediblemeds.org.).

<u>Arm H:</u>

Drugs known to prolong the QT interval are prohibited at registration and during the active treatment phase for patients receiving sitravatinib (Arm H). Any patients taking such drugs at or prior to registration should discontinue the drug 6 half-lives plus 1 day prior to commencing trial treatment.

All concomitant medications listed on https://www.crediblemeds.org under the following risk category will exclude patients from entering the trial:

- **Known Risk of TdP**

Concomitant medications listed under the categories **'possible risk of TdP'** or **'Conditional risk of TdP'** may be permitted **with caution**. Whilst additional monitoring is not mandated, the development of cardiac related signs and symptoms should be closely observed and acted upon.

<u>Definitions:</u>

- **Known Risk of TdP:** These drugs prolong the QT interval **<u>AND</u>** are clearly associated with a known risk of TdP, even when taken as recommended.
- **Possible Risk of TdP:** These drugs can cause QT prolongation **<u>BUT</u>** currently lack evidence for a risk of TdP when taken as recommended.
- **Conditional Risk of TdP:** These drugs are associated with TdP **<u>BUT</u>** only under certain conditions of their use (e.g. excessive dose, in patients with conditions such as hypokalaemia, or when taken with interacting drugs) **<u>OR</u>** by creating conditions that facilitate or induce TdP (e.g. by inhibiting metabolism of a QT-prolonging drug or by causing an electrolyte disturbance that induces TdP).
- **Drugs to Avoid in Congenital Long QT:** These drugs pose a high risk of TdP for patients with CLQTS and include all those in the above three categories (KR, PR & CR) **<u>PLUS</u>** additional drugs that do not prolong the QT interval per se but which have a Special Risk (SR) because of their other actions.

Generic Name	Drug Class	Therapeutic Use	Risk Category	Route
**Abarelix**	Gonadotropin-releasing hormone antagonist	Prostate	Possible Risk, Drugs to Avoid in Congenital Long QT	injection
**Abiraterone**	Anti-cancer	Prostate cancer	Conditional Risk, Drugs to Avoid in Congenital Long QT	oral
**Aclarubicin**	Anti-cancer	Cancer	Known Risk, Drugs to Avoid in Congenital Long QT	injection

Generic Name	Drug Class	Therapeutic Use	Risk Category	Route
**Albuterol (salbutamol)**	Bronchodilator	Asthma	Drugs to Avoid in Congenital Long QT	oral, injection, inhaled
**Alfuzosin**	Alpha1-blocker	Benign prostatic hyperplasia	Possible Risk, Drugs to Avoid in Congenital Long QT	oral
**Alimemazine (trimeprazine)**	Antihistamine, antiemetic, sedative	Urticaria, pruritus, sedative (paediatrics)	Possible risk TdP, Drug to avoid in congenital long QT	oral
**Amiodarone**	Anti-arrhythmic	Abnormal heart rhythm	Known Risk, Drugs to Avoid in Congenital Long QT	oral, injection
**Amphetamine (Amfetamine)**	CNS stimulant	ADHD	Drugs to Avoid in Congenital Long QT	oral, injection
**Amsacrine**	Anti-cancer	Lymphoma, acute adult leukaemia	Conditional Risk, Drugs to Avoid in Congenital Long QT	injection
**Anagrelide**	Phosphodiesterase 3 inhibitor	Thrombocythemia	Known Risk, Drugs to Avoid in Congenital Long QT	oral
**Apalutamide**	Anti-cancer	Prostate	Possible Risk, Drugs to Avoid in Congenital Long QT	oral
**Apomorphine**	Dopamine agonist	Parkinson's disease	Possible Risk, Drugs to Avoid in Congenital Long QT	oral, injection
**Arformoterol**	Bronchodilator	Bronchoconstriction in COPD	Drugs to Avoid in Congenital Long QT	inhaled
**Aripiprazole**	Anti-psychotic, atypical	Schizophrenia, depression (adjunct)	Possible Risk, Drugs to Avoid in Congenital Long QT	oral, injection
**Arsenic trioxide**	Anti-cancer	Cancer (leukemia)	Known Risk, Drugs to Avoid in Congenital Long QT	injection
**Artemether + lumefantrine**	Anti-malarial	Malaria	Possible Risk, Drugs to Avoid in Congenital Long QT	oral
**Artenimol + piperaquine (Only on Non US Market)**	Anti-malarial	Malaria	Possible Risk, Drugs to Avoid in Congenital Long QT	oral
**Asenapine**	Antipsychotic, atypical	Schizophrenia	Possible Risk, Drugs to Avoid in Congenital Long QT	sublingual
**Astemizole (Removed from Market)**	Antihistamine	Allergic rhinitis	Known Risk, Drugs to Avoid in Congenital Long QT	oral
**Atomoxetine**	Norepinephrine reuptake inhibitor	ADHD	Possible Risk, Drugs to Avoid in Congenital Long QT	oral
**Azithromycin**	Antibiotic	Bacterial infection	Known Risk, Drugs to Avoid in Congenital Long QT	oral, injection
**Bedaquiline**	Antibiotic	Tuberculosis, Multi-drug resistant	Possible Risk, Drugs to Avoid in Congenital Long QT	oral
**Bendamustine**	Alkylating agent	Leukemia, lymphoma	Possible Risk, Drugs to Avoid in Congenital Long QT	injection
**Benperidol (Only on Non US Market)**	Anti-psychotic	Antipsychotic	Possible Risk, Drugs to Avoid in Congenital Long QT	oral
**Bepridil (Removed from Market)**	Anti-anginal	Angina Pectoris (heart pain)	Known Risk, Drugs to Avoid in Congenital Long QT	oral
**Betrixaban**	Anticoagulant	Anticoagulant	Possible Risk, Drugs to Avoid in Congenital Long QT	oral

Generic Name	Drug Class	Therapeutic Use	Risk Category	Route
**Bortezomib**	Proteasome inhibitor	Cancer (multiple myeloma,lymphoma)	Possible Risk, Drugs to Avoid in Congenital Long QT	injection
**Bosutinib**	Tyrosine kinase inhibitor	Cancer (leukemia)	Possible Risk, Drugs to Avoid in Congenital Long QT	oral
**Buprenorphine**	Opioid receptor modulator	Narcotic additction and pain	Possible Risk, Drugs to Avoid in Congenital Long QT	sublingual, topical, injection
**Cabozantinib**	Anti-cancer	Renal cell carcinoma	Possible Risk, Drugs to Avoid in Congenital Long QT	oral
**Capecitabine**	Anticancer	Cancer (GI, Breast)	Possible Risk, Drugs to Avoid in Congenital Long QT	oral
**Ceritinib**	Kinase inhibitor	Cancer (Lung)	Possible Risk, Drugs to Avoid in Congenital Long QT	oral
**Chloroquine**	Anti-malarial	Malaria	Known Risk, Drugs to Avoid in Congenital Long QT	oral
**Chlorpromazine**	Anti-psychotic / Anti-emetic	Schizophrenia, nausea, many others	Known Risk, Drugs to Avoid in Congenital Long QT	oral, injection, suppository
**Cilostazol**	Phosphodiesterase 3 inhibitor	Intermittent claudication	Known Risk, Drugs to Avoid in Congenital Long QT	oral
**Cimetidine**	Histamine H2 receptor antagonist	Heartburn, peptic ulcers	Conditional Risk, Drugs to Avoid in Congenital Long QT	oral
**Ciprofloxacin**	Antibiotic	Bacterial infection	Known Risk, Drugs to Avoid in Congenital Long QT	oral, injection
**Cisapride (Removed from Market)**	GI stimulant	Increase GI motility	Known Risk, Drugs to Avoid in Congenital Long QT	oral
**Citalopram**	Anti-depressant, SSRI	Depression	Known Risk, Drugs to Avoid in Congenital Long QT	oral
**Clarithromycin**	Antibiotic	Bacterial infection	Known Risk, Drugs to Avoid in Congenital Long QT	oral
**Clofazimine (Only on Non US Market)**	Antibiotic	Antimycobacterial	Possible Risk, Drugs to Avoid in Congenital Long QT	oral
**Clomipramine**	Anti-depressant, Tricyclic	Depression	Possible Risk, Drugs to Avoid in Congenital Long QT	oral, injection
**Clotiapine**	Antipsychotic	Schizophrenia	Possible Risk, Drugs to Avoid in Congenital Long QT	oral
**Clozapine**	Anti-psychotic, atypical	Schizophrenia	Possible Risk, Drugs to Avoid in Congenital Long QT	oral
**Cobimetinib**	Anti-cancer	Cancer	Possible risk TdP, Drug to avoid in congenital long QT	oral
**Cocaine**	Local anesthetic	Anesthesia (topical)	Known Risk, Drugs to Avoid in Congenital Long QT	oral, nasal
**Crizotinib**	Kinase inhibitor	Cancer (Non-small cell lung cancer, metastatic)	Possible Risk, Drugs to Avoid in Congenital Long QT	oral
**Cyamemazine (cyamepromazine) (Only on Non US Market)**	Antipsychotic	Schizophrenia, sedation	Possible Risk, Drugs to Avoid in Congenital Long QT	oral, injection
**Dabrafenib**	Kinase inhibitor	Cancer (melanoma)	Possible Risk, Drugs to Avoid in Congenital Long QT	oral

Generic Name	Drug Class	Therapeutic Use	Risk Category	Route
**Dasatinib**	Tyrosine kinase inhibitor	Cancer (leukemia)	Possible Risk, Drugs to Avoid in Congenital Long QT	oral
**Degarelix**	Gonadotropin Releasing Hormone Agonist/antagonis t	Cancer (prostate)	Possible Risk, Drugs to Avoid in Congenital Long QT	injection, suppository
**Delaminid (only on Non US Market)**	Antibiotic	Tuberculosis, drug resistant	Possible Risk, Drugs to Avoid in Congenital Long QT	oral
**Desipramine**	Anti-depressant, Tricyclic	Depression	Possible Risk, Drugs to Avoid in Congenital Long QT	oral
**Deutetrabenazin e**	Vesicular monamine transporter 2 inhibitor	Chorea (Huntington's disease)	Possible Risk, Drugs to Avoid in Congenital Long QT	oral
**Dexmedetomidin e**	Sedative	Sedation	Possible Risk, Drugs to Avoid in Congenital Long QT	injection
**Dexmethylpheni date**	CNS stimulant	ADHD	Drugs to Avoid in Congenital Long QT	oral
**Dextroampheta mine (Dexamfetamine)**	CNS stimulant	ADHD, obesity	Drugs to Avoid in Congenital Long QT	oral
**Dextromethorph an + Quinidine**	Sedative/Anti-arrhythmic agent	Pseudobulbar affect (PBA	Possible Risk, Drugs to Avoid in Congenital Long QT	oral
**Disopyramide**	Anti-arrhythmic	Abnormal heart rhythm	Known Risk, Drugs to Avoid in Congenital Long QT	oral, injection
**Dobutamine**	Inotrope	Heart failure, shock (low blood pressure)	Drugs to Avoid in Congenital Long QT	injection
**Dofetilide**	Anti-arrhythmic	Abnormal heart rhythm	Known Risk, Drugs to Avoid in Congenital Long QT	oral
**Dolasetron**	Anti-emetic	Nausea, vomiting	Possible Risk, Drugs to Avoid in Congenital Long QT	oral, injection
**Domperidone (Only on Non US Market)**	Anti-nausea	Nausea, vomiting	Known Risk, Drugs to Avoid in Congenital Long QT	oral, injection, suppository
**Donepezil**	Cholinesterase inhibitor	Dementia (Alzheimer's Disease)	Known Risk, Drugs to Avoid in Congenital Long QT	oral
**Dopamine**	Inotrope	Heart failure, shock (low blood pressure)	Drugs to Avoid in Congenital Long QT	injection
**Dronedarone**	Anti-arrhythmic	Abnormal heart rhythm	Known Risk, Drugs to Avoid in Congenital Long QT	oral
**Droperidol**	Anti-psychotic / Anti-emetic	Anesthesia (adjunct), nausea	Known Risk, Drugs to Avoid in Congenital Long QT	injection
**Droxidopa**	Adrenergic pro-drug	Neurogenic orthostatic hypotension	Drugs to Avoid in Congenital Long QT	oral
**Efavirenz**	Anti-retroviral	HIV	Possible Risk, Drugs to Avoid in Congenital Long QT	oral
**Eliglustat**	Glucosylceramide synthase inhibitor	Gaucher's disease	Possible Risk, Drugs to Avoid in Congenital Long QT	oral
**Encorafenib**	Anti-cancer	Melanoma	Possible Risk, Drugs to Avoid in Congenital Long QT	oral

Generic Name	Drug Class	Therapeutic Use	Risk Category	Route
**Entrectinib**	Anti-cancer	Non-Small Cell Lung Cancer	Possible Risk, Drugs to Avoid in Congenital Long QT	oral
**Eperisone**	Anti-spasmodic	Spasticity	Conditional Risk, Drugs to Avoid in Congenital Long QT	oral
**Ephedrine**	Bronchodilator, decongestant	Allergic reaction, allergic rhinitis, asthma	Drugs to Avoid in Congenital Long QT	oral, injection
**Epinephrine (Adrenaline)**	Catecholamine, vasoconstrictor	Allergic reaction, anaphylaxis, cardiac arrest	Drugs to Avoid in Congenital Long QT	injection, inhaled
**Epirubicin**	Anti-cancer	Cancer	Possible Risk, Drugs to Avoid in Congenital Long QT	injection
**Eribulin mesylate**	Microtubule inhibitor	Cancer (breast, metastatic)	Possible Risk, Drugs to Avoid in Congenital Long QT	injection
**Erythromycin**	Antibiotic	Bacterial infection, increase GI motility	Known Risk, Drugs to Avoid in Congenital Long QT	oral, injection
**Escitalopram**	Anti-depressant, SSRI	Depression (major), anxiety disorders	Known Risk, Drugs to Avoid in Congenital Long QT	oral
**Ezogabine (Retigabine)**	Anti-convulsant	Seizures, Partial	Possible Risk, Drugs to Avoid in Congenital Long QT	oral
**Felbamate**	Anti-convulsant	Epilepsy	Possible Risk, Drugs to Avoid in Congenital Long QT	oral
**Fenfluramine (Removed from Market)**	Appetite suppressant	Obesity	Drugs to Avoid in Congenital Long QT	oral
**Fenoterol**	Bronchodilator	Asthma	Drugs to Avoid in Congenital Long QT	oral, inhaled
**Fingolimod**	Sphingosine phospate receptor modulator	Multiple Sclerosis	Possible Risk, Drugs to Avoid in Congenital Long QT	oral
**Flecainide**	Anti-arrhythmic	Abnormal heart rhythm	Known Risk, Drugs to Avoid in Congenital Long QT	oral
**Fluconazole**	Anti-fungal	Fungal infection	Known Risk, Drugs to Avoid in Congenital Long QT	oral, injection
**Fluorouracil (5-FU)**	Anti-cancer	Cancer	Possible Risk, Drugs to Avoid in Congenital Long QT	injection
**Flupentixol (Only on Non US Market)**	Dopamine 2 and 5HT2a antagonist	Schizophrenia	Possible Risk, Drugs to Avoid in Congenital Long QT	oral, injection
**Fluticasone (when used in combination with Salmetarol)**	Corticosteroid	Asthma	Drugs to Avoid in Congenital Long QT	oral, inhaled
**Formoterol**	Bronchodilator	Asthma	Drugs to Avoid in Congenital Long QT	injection, inhaled
**Gatifloxacin (Removed from Market)**	Antibiotic	Bacterial infection	Known Risk, Drugs to Avoid in Congenital Long QT	oral, injection
**Gemifloxacin**	Antibiotic	Bacterial infection	Possible Risk, Drugs to Avoid in Congenital Long QT	oral
**Gilteritinib**	Anti-cancer	Cancer	Possible risk TdP, Drug to avoid in congenital long QT	oral
**Glasdegib (Daurismo)**	Anti-cancer	Acute myeloid leukemia (AML)	Possible risk TdP, Drug to avoid in congenital long QT	oral

Generic Name	Drug Class	Therapeutic Use	Risk Category	Route
**Granisetron**	Anti-emetic	Nausea, vomiting	Possible Risk, Drugs to Avoid in Congenital Long QT	oral, injection, topical
**Grepafloxacin (Removed from Market)**	Antibiotic	Bacterial infection	Known Risk, Drugs to Avoid in Congenital Long QT	oral
**Halofantrine (Only on Non US Market)**	Anti-malarial	Malaria	Known Risk, Drugs to Avoid in Congenital Long QT	oral
**Haloperidol**	Anti-psychotic	Schizophrenia, agitation	Known Risk, Drugs to Avoid in Congenital Long QT	oral, injection
**Hydrocodone – ER**	Analgesic	Pain, severe	Possible Risk, Drugs to Avoid in Congenital Long QT	oral, suppository
**Hydroquinidine**	Class Ia antiarrhythmic	Arrhythmia	Known risk TdP, Drug to avoid in congenital long QT	oral
**Hydroxychloroquine**	Antimalarial	Malaria	Known Risk, Drugs to Avoid in Congenital Long QT	oral
**Ibogaine (Only on Non US Market)**	Psychedelic	Narcotic addiction, unproven	Known Risk, Drugs to Avoid in Congenital Long QT	oral
**Ibutilide**	Anti-arrhythmic	Abnormal heart rhythm	Known Risk, Drugs to Avoid in Congenital Long QT	injection
**Iloperidone**	Anti-psychotic, atypical	Schizophrenia	Possible Risk, Drugs to Avoid in Congenital Long QT	oral, injection
**Imipramine (melipramine)**	Anti-depressant, Tricyclic	Depression	Possible Risk, Drugs to Avoid in Congenital Long QT	oral
**Indacaterol**	Bronchodilator	Chronic obstructive lung disease	Drugs to Avoid in Congenital Long QT	inhaled
**Inotuzumab ozogamicin**	Antineoplastic Agent	Acute Lymphocytic Leukemia	Possible Risk, Drugs to Avoid in Congenital Long QT	injection
**Isoproterenol**	Bronchodilator	Allergic reaction	Drugs to Avoid in Congenital Long QT	injection, inhaled
**Isradipine**	Anti-hypertensive	Hypertension	Possible Risk, Drugs to Avoid in Congenital Long QT	oral
**Ivosidenib**	Anti-cancer	Cancer	Possible risk TdP, Drug to avoid in congenital long QT	oral
**Ketanserin (Only on Non US Market)**	Antihypertensive	Hypertension	Possible Risk, Drugs to Avoid in Congenital Long QT	oral
**Lacidipine**	Antihypertensive	Hypertension	Possible Risk, Drugs to Avoid in Congenital Long QT	oral
**Lapatinib**	Kinase inhibitor	Cancer (breast, metastatic)	Possible Risk, Drugs to Avoid in Congenital Long QT	oral
**Lefamulin**	Antibiotic	Community acquired pneumonia	Possible Risk, Drugs to Avoid in Congenital Long QT	oral, injection
**Lenvatinib**	Anticancer	Cancer (Thyroid)	Possible Risk, Drugs to Avoid in Congenital Long QT	oral
**Leuprolide**	Gonadotropin receptor agonist/antogist	Cancer (prostate)	Possible Risk, Drugs to Avoid in Congenital Long QT	injection
**Levalbuterol (levsalbutamol)**	Bronchodilator	Asthma	Drugs to Avoid in Congenital Long QT	oral, inhaled
Levetiracetam	Anti-convulsant	Epilepsy	Possible Risk, Drugs to Avoid in Congenital Long QT	oral, injection

Generic Name	Drug Class	Therapeutic Use	Risk Category	Route
**Levmetampheta mine**	Decongestant	Nasal Congestion	Drugs to Avoid in Congenital Long QT	inhaled
**Levofloxacin**	Antibiotic	Bacterial infection	Known Risk, Drugs to Avoid in Congenital Long QT	oral, injection
**Levomepromazi ne (Only on Non US Market)**	Antipsychotic	Schizophrenia	Known Risk, Drugs to Avoid in Congenital Long QT	oral, injection
**Levomethadone**	Opioid agonist	Narcotic dependence	Possible Risk, Drugs to Avoid in Congenital Long QT	oral
**Levomethadyl acetate (Removed from Market)**	Opioid agonist	Narcotic dependence	Known Risk, Drugs to Avoid in Congenital Long QT	oral
**Levosulpiride (Only on Non US Market)**	Antipsychotic	Schizophrenia	Known Risk, Drugs to Avoid in Congenital Long QT	oral, injection
**Lisdexamfetami ne**	CNS stimulant	ADHD	Drugs to Avoid in Congenital Long QT	oral
**Lithium**	Anti-mania	Bipolar disorder	Possible Risk, Drugs to Avoid in Congenital Long QT	oral, injection
**Lopinavir and ritonavir**	Viral protease inhibitor	HIV/AIDS	Possible Risk, Drugs to Avoid in Congenital Long QT	oral
**Lofexidine (Lucemyra)**	α_{2A} adrenergic receptor agonist	Hypertension, opiod withdrawal	Possible risk TdP, Drug to avoid in congenital long QT	oral
**Maprotiline**	Anti-depressant	Depression	Possible Risk, Drugs to Avoid in Congenital Long QT	oral, injection
**Meglumine antimoniate**	Antiprotozoal	Parasitic Infection	Known Risk, Drugs to Avoid in Congenital Long QT	injection
**Melperone (Only on Non US Market)**	Antipsychotic, atypical	Schizophrenia	Possible Risk, Drugs to Avoid in Congenital Long QT	oral, injection
**Memantine**	NMDA receptor blocker	Dementia	Possible Risk, Drugs to Avoid in Congenital Long QT	oral
**Mesoridazine (Removed from Market)**	Anti-psychotic	Schizophrenia	Known Risk, Drugs to Avoid in Congenital Long QT	oral
**Metaproterenol (orciprenaline)**	Bronchodilator	Asthma	Drugs to Avoid in Congenital Long QT	oral, inhaled
**Methadone**	Opioid agonist	Narcotic dependence, pain	Known Risk, Drugs to Avoid in Congenital Long QT	oral, injection
**Methamphetami ne (metamfetamine)**	CNS stimulant	Obesity, ADHD	Drugs to Avoid in Congenital Long QT	oral, injection, inhaled
**Methylphenidate**	CNS stimulant	ADHD	Drugs to Avoid in Congenital Long QT	oral, injection, inhaled
**Mianserin (Not on US market)**	Atypical antidepressant	Depression	Possible Risk, Drugs to Avoid in Congenital Long QT	oral
**Midodrine**	Vasoconstrictor	Hypotension	Drugs to Avoid in Congenital Long QT	oral
**Midostaurin**	Anti-cancer	Avute myeloid leukemia	Possible Risk, Drugs to Avoid in Congenital Long QT	oral
**Mifepristone**	Progesterone antagonist	Pregnancy termination	Possible Risk, Drugs to Avoid in Congenital Long QT	oral
**Mirabegron**	Beta3 adrenergic antagonist	Bladder spasm	Possible Risk, Drugs to Avoid in Congenital Long QT	oral

Generic Name	Drug Class	Therapeutic Use	Risk Category	Route
**Mirtazapine**	Anti-depressant, Tetracyclic	Depression	Possible Risk, Drugs to Avoid in Congenital Long QT	oral
**Moexipril/HCTZ**	Anti-hypertensive	Hypertension	Possible Risk, Drugs to Avoid in Congenital Long QT	oral
**Moxifloxacin**	Antibiotic	Bacterial infection	Known Risk, Drugs to Avoid in Congenital Long QT	oral, injection
**Necitumumab**	Anti-cancer	Lung cancer	Possible Risk, Drugs to Avoid in Congenital Long QT	injection
**Nicardipine**	Anti-hypertensive	Hypertension	Possible Risk, Drugs to Avoid in Congenital Long QT	oral, injection
**Nifekalant (marketed in Japan)**	Class II antiarrhythmic agent	Arrhythmias	Known Risk, Drugs to Avoid in Congenital Long QT	intravenous
**Nilotinib**	Kinase inhibitor	Cancer (leukemia)	Possible Risk, Drugs to Avoid in Congenital Long QT	oral
**Norepinephrine**	Vasconstrictor, Inotrope	Heart failure, shock (low blood pressure)	Drugs to Avoid in Congenital Long QT	oral, injection
**Norfloxacin**	Antibiotic	Bacterial infection	Possible Risk, Drugs to Avoid in Congenital Long QT	oral
**Nortriptyline**	Anti-depressant, Tricyclic	Depression	Possible Risk, Drugs to Avoid in Congenital Long QT	oral
**Nusinersen**	Antisense oligonucleotide	Spinal muscular atrophy	Possible Risk, Drugs to Avoid in Congenital Long QT	injection
**Ofloxacin**	Antibiotic	Bacterial infection	Possible Risk, Drugs to Avoid in Congenital Long QT	oral, injection
Oliceridine	Opioid	Pain	Possible Risk, Drugs to Avoid in Congenital Long QT	injection
**Olodaterol**	Bronchodilator	Asthma	Drugs to Avoid in Congenital Long QT	inhaled
**Ondansetron**	Anti-emetic	Nausea, vomiting	Known Risk, Drugs to Avoid in Congenital Long QT	oral, injection, suppository
**Osimertinib**	Tyrosine kinase inhibitor	Cancer (EGFR pos. NSC Lung cancer)	Possible Risk, Drugs to Avoid in Congenital Long QT	oral
**Oxaliplatin**	Antineoplastic Agent	Cancer	Known Risk, Drugs to Avoid in Congenital Long QT	injection
**Oxymetazoline**	**Sympathomimetic**	Nasal congestion	Drugs to Avoid in Congenital Long QT	intranasal
**Oxytocin**	Oxytocic	Labor stimulation	Possible Risk, Drugs to Avoid in Congenital Long QT	injection
**Ozanimod**	Sphingosine 1-Phosphate (S1P) receptor Modulator	Multiple Sclerosis	Possible Risk, Drugs to Avoid in Congenital Long QT	oral
**Paliperidone**	Anti-psychotic, atypical	Schizophrenia	Possible Risk, Drugs to Avoid in Congenital Long QT	oral, injection
**Palonosetron**	Antiemetic	Nausea	Possible Risk, Drugs to Avoid in Congenital Long QT	injection
**Panobinostat**	Histone deacetylase inhibitor	Multiple myeloma (Part of a 3 drug regimen)	Possible Risk, Drugs to Avoid in Congenital Long QT	oral
**Papaverine HCl (Intra-coronary)**	Vasodilator, Coronary	Diagnostic adjunct	Known Risk, Drugs to Avoid in Congenital Long QT	injection
**Pasireotide**	Somatostatin analog	Cushings Disease	Possible Risk, Drugs to Avoid in Congenital Long QT	injection, topical

Generic Name	Drug Class	Therapeutic Use	Risk Category	Route
**Pazopanib**	Tyrosine kinase inhibitor	Cancer (renal cell, sarcoma)	Possible Risk, Drugs to Avoid in Congenital Long QT	oral
**Pentamidine**	Antifungal	Fungal infection (Pneumocystis pneumonia)	Known Risk, Drugs to Avoid in Congenital Long QT	injection, inhaled
**Perflutren lipid microspheres**	Imaging contrast agent	Echocardiography	Possible Risk, Drugs to Avoid in Congenital Long QT	injection
**Perphenazine**	Antipsychotic	Schizophrenia	Possible Risk, Drugs to Avoid in Congenital Long QT	oral, injection
**Phentermine**	Appetite suppressant	Obesity	Drugs to Avoid in Congenital Long QT	oral
**Phenylephrine**	Vasoconstrictor	Shock (low blood pressure), allergic rhinitis, asthma	Drugs to Avoid in Congenital Long QT	oral, injection
**Phenylpropanolamine**	Appetite suppressant	Obesity	Drugs to Avoid in Congenital Long QT	oral
**Pilsicainide (Only on Non US Market)**	Anti-arrhythmic	Arrythmia	Possible Risk, Drugs to Avoid in Congenital Long QT	oral, injection
**Pimavanserin**	Antipsychotic, atypical	Psychosis, Parkinson's disease	Possible Risk, Drugs to Avoid in Congenital Long QT	oral
**Pimozide**	Anti-psychotic	Tourette's Disorder	Known Risk, Drugs to Avoid in Congenital Long QT	oral
**Pipamperone (Only on Non US Market)**	Antipsychotic	Schizophrenia	Possible Risk, Drugs to Avoid in Congenital Long QT	oral
**Pitolisant**	Histamine receptor antagonist	Narcolepsy / daytime sleepiness	Possible Risk, Drugs to Avoid in Congenital Long QT	oral
**Pretomanid**	Anti-bacterial	Drug-resistant TB	Possible Risk, Drugs to Avoid in Congenital Long QT	oral
**Primaquine phosphate**	Anti-malarial	Malaria	Possible Risk, Drugs to Avoid in Congenital Long QT	oral
**Probucol (Removed from Market)**	Antilipemic	Hypercholesterolemia	Known Risk, Drugs to Avoid in Congenital Long QT	oral
**Procainamide**	Anti-arrhythmic	Abnormal heart rhythm	Known Risk, Drugs to Avoid in Congenital Long QT	injection
**Promethazine**	Anti-psychotic / Anti-emetic	Nausea, vomiting	Possible Risk, Drugs to Avoid in Congenital Long QT	oral, injection, suppository
**Propofol**	Anesthetic, general	Anesthesia	Known Risk, Drugs to Avoid in Congenital Long QT	injection
**Prothipendyl (Only on Non US Market)**	Antipsychotic	Schizophrenia	Possible Risk, Drugs to Avoid in Congenital Long QT	oral, injection
**Pseudoephedrine**	Decongestant	Allergic reaction, allergic rhinitis, asthma	Drugs to Avoid in Congenital Long QT	oral, inhaled
**Quinidine**	Anti-arrhythmic	Abnormal heart rhythm	Known Risk, Drugs to Avoid in Congenital Long QT	oral, injection
Remimazolam	Sedative	Anasthesia or Sedation	Possible Risk, Drugs to Avoid in Congenital Long QT	injection
**Ribociclib**	Cyclin dependent kinase inhibitor	Breast cancer	Possible Risk, Drugs to Avoid in Congenital Long QT	oral

Generic Name	Drug Class	Therapeutic Use	Risk Category	Route
**Rilpivirine**	Anti-viral	Viral infection (HIV/AIDS)	Possible Risk, Drugs to Avoid in Congenital Long QT	oral
**Risperidone**	Anti-psychotic, atypical	Schizophrenia	Conditional Risk, Drugs to Avoid in Congenital Long QT	oral, injection
**Ritodrine (Removed from Market)**	Muscle relaxant	Premature labor	Drugs to Avoid in Congenital Long QT	oral, injection
**Romidepsin**	Histone deacetylase inhibitor	Lymphoma	Possible Risk, Drugs to Avoid in Congenital Long QT	injection
**Roxithromycin (Only on Non US Market)**	Antibiotic	Bacterial infection	Known Risk, Drugs to Avoid in Congenital Long QT	oral
**Salmeterol**	Bronchodilator	Asthma	Drugs to Avoid in Congenital Long QT	inhaled
**Saquinavir**	Anti-viral	Viral infection (HIV/AIDS)	Possible Risk, Drugs to Avoid in Congenital Long QT	oral
**Sertindole (Only on Non US Market)**	Anti-psychotic, atypical	Schizophrenia, anxiety	Possible Risk, Drugs to Avoid in Congenital Long QT	oral
**Sevoflurane**	Anesthetic, general	Anesthesia	Known Risk, Drugs to Avoid in Congenital Long QT	inhaled
**Sibutramine (Removed from Market)**	Appetite suppressant	Obesity	Drugs to Avoid in Congenital Long QT	oral
**Siponimid**	Mutliple Sclerosis therapy	Multiple Sclerosis	Possible Risk, Drugs to Avoid in Congenital Long QT	oral
**Sorafenib**	Tyrosine kinase inhibitor	Cancer (liver, renal cell, metastatic thyroid)	Possible Risk, Drugs to Avoid in Congenital Long QT	oral
**Sotalol**	Anti-arrhythmic	Abnormal heart rhythm	Known Risk, Drugs to Avoid in Congenital Long QT	oral
**Sparfloxacin (Removed from Market)**	Antibiotic	Bacterial infection	Known Risk, Drugs to Avoid in Congenital Long QT	oral
**Sulpiride (Only on Non US Market)**	Anti-psychotic, atypical	Schizophrenia	Known Risk, Drugs to Avoid in Congenital Long QT	oral, inhaled
**Sultopride (Only on Non US Market)**	Antipsychotic, atypical	Schizophrenia	Known Risk, Drugs to Avoid in Congenital Long QT	oral, injection
**Sunitinib**	Kinase inhibitor	Cancer (GIST, renal cell, pNET)	Possible Risk, Drugs to Avoid in Congenital Long QT	oral
**Tacrolimus**	Immunosuppressant	Immune suppression	Possible Risk, Drugs to Avoid in Congenital Long QT	oral, injection
**Tamoxifen**	Anti-cancer	Cancer (breast)	Possible Risk, Drugs to Avoid in Congenital Long QT	oral
**Telavancin**	Antibiotic	Bacterial infection	Possible Risk, Drugs to Avoid in Congenital Long QT	injection
**Telithromycin**	Antibiotic	Bacterial infection	Possible Risk, Drugs to Avoid in Congenital Long QT	oral
**Terbutaline**	Bronchodilator	Asthma, premature labour	Drugs to Avoid in Congenital Long QT	oral, injection, inhaled

Generic Name	Drug Class	Therapeutic Use	Risk Category	Route
**Terfenadine (Removed from Market)**	Antihistamine	Allergic rhinitis	Known Risk, Drugs to Avoid in Congenital Long QT	oral
**Terlipressin (Only on Non US Market)**	Vasoconstrictor	Septic shock	Known Risk, Drugs to Avoid in Congenital Long QT	injection
**Terodiline (Only on Non US Market)**	Muscle relaxant	Bladder spasm	Known Risk, Drugs to Avoid in Congenital Long QT	oral
**Tetrabenazine**	Monoamine Transporter Inhibitor	Chorea (Huntington's disease)	Possible Risk, Drugs to Avoid in Congenital Long QT	oral
**Thioridazine**	Anti-psychotic	Schizophrenia	Known Risk, Drugs to Avoid in Congenital Long QT	oral
**Tiapride (Only on Non US Market)**	Selective D2, D3 dopamine antagonist	Alcoholism, withdrawal	Possible Risk, Drugs to Avoid in Congenital Long QT	oral, injection
**Tipiracil and Trifluridine**	Anti-cancer	Metastatic colorectal cancer	Possible Risk, Drugs to Avoid in Congenital Long QT	oral
**Tizanidine**	Muscle relaxant	Muscle spasticity	Possible Risk, Drugs to Avoid in Congenital Long QT	oral
**Tolterodine**	Muscle relaxant	Bladder spasm	Possible Risk, Drugs to Avoid in Congenital Long QT	oral
**Toremifene**	Estrogen agonist/antagonist	Cancer (breast, metastatic)	Possible Risk, Drugs to Avoid in Congenital Long QT	oral
**Tramadol**	Analgesic	Pain	Possible Risk, Drugs to Avoid in Congenital Long QT	oral, injection, suppository
**Trimethoprim-Sulfamethoxazole**	Antibiotic	Bacterial infection	Drugs to Avoid in Congenital Long QT	oral
**Trimipramine**	Anti-depressant, Tricyclic	Depression	Possible Risk, Drugs to Avoid in Congenital Long QT	oral, injection
**Tropisetron (Only on Non US Market)**	Anti-emetic	Nausea, vomiting	Possible Risk, Drugs to Avoid in Congenital Long QT	oral, injection
**Valbenazine**	Vesicular monamine transporter 2 inhibitor	Tardive Dyskinesia	Possible Risk, Drugs to Avoid in Congenital Long QT	oral
**Vandetanib**	Anti-cancer	Cancer (thyroid)	Known Risk, Drugs to Avoid in Congenital Long QT	oral
**Vardenafil**	Phosphodiesterase 5 inhibitor	Erectile dysfunction	Possible Risk, Drugs to Avoid in Congenital Long QT	oral, injection
**Vemurafenib**	Kinase inhibitor	Cancer (melanoma)	Possible Risk, Drugs to Avoid in Congenital Long QT	oral
**Venlafaxine**	Anti-depressant, SNRI	Depression	Possible Risk, Drugs to Avoid in Congenital Long QT	oral
**Vilanterol/fluticasone furoate**	Bronchodilator	Asthma	Drugs to Avoid in Congenital Long QT	inhaled
**Vorinostat**	Histone deacetylase inhibitor	Cancer (lymphoma)	Possible Risk, Drugs to Avoid in Congenital Long QT	oral
**Xylometazoline**	Alpha-adrenergic agonist	Nasal congestion, allergic rhinitis, and sinusitis	Drug to avoid in congenital long QT	intranasal

Generic Name	Drug Class	Therapeutic Use	Risk Category	Route
**Zotepine (Only on Non US Market)**	Antipsychotic, atypical	Schizophrenia	Possible Risk, Drugs to Avoid in Congenital Long QT	oral
**Zuclopenthixol**	Antipsychotic	Schizophrenia	Possible Risk, Drugs to Avoid in Congenital Long QT	oral

41 APPENDIX 10: NEW YORK HEART ASSOCIATION CLASSIFICATION – STAGES OF HEART FAILURE

Class	Patient Symptoms
Class I (mild)	No limitation of physical activity. Ordinary physical activity does not cause undue fatigue, palpitation, or dyspnoea (shortness of breath).
Class II (mild)	Slight limitation of physical activity. Comfortable at rest, but ordinary physical activity results in fatigue, palpitation, or dyspnoea.
Class III (moderate)	Marked limitation of physical activity. Comfortable at rest, but less than ordinary activity causes fatigue, palpitation, or dyspnoea.
Class IV (severe)	Unable to carry out any physical activity without discomfort. Symptoms of cardiac insufficiency at rest. If any physical activity is undertaken, discomfort is increased.

American Heart Association (2016) *Classes of Heart Failure,* Available at:*http://www.heart.org/HEARTORG/Conditions/HeartFailure/AboutHeartFailure/Classes-of-Heart-Failure_UCM_306328_Article.jsp#.WA8RGvkrJhG* (Accessed: 25th October 2016).

42 APPENDIX 11: CANADIAN CARDIOVASCULAR SOCIETY GRADING OF ANGINA PECTORIS.

Grade	Description
I	Ordinary physical activity does not cause angina, such as walking and climbing stairs. Angina with strenuous or rapid or prolonged exertion at work or recreation.
II	Slight limitation of ordinary activity. Walking or climbing stairs rapidly, walking uphill, walking or stair climbing after meals, or in cold, or in wind, or under emotional stress, or only during the few hours after awakening. Walking more than two blocks on the level and climbing more than one flight of ordinary stairs at a normal pace and in normal conditions.
III	Marked limitation of ordinary physical activity. Walking one or two blocks on the level and climbing one flight of stairs in normal conditions and at normal pace.
IV	Inability to carry on any physical activity without discomfort, anginal syndrome may be present at rest.

Campeau Lucien. Grading of angina pectoris
Circulation 1976;54:5223 Available on the Canadian Cardiovascular Society Website at www.ccs.ca

43 APPENDIX 12: ACTIONS REQUIRED IN CASE OF INCREASES IN LIVER BIOCHEMISTRY AND EVALUATION OF HY'S LAW

43.1 Introduction

This Appendix describes the process to be followed in order to identify and appropriately report cases of Hy's Law. It is not intended to be a comprehensive guide to the management of elevated liver biochemistries.

As per FDA guidance, discontinuation of treatment and further evaluation of drug induced liver injury should be considered if not otherwise explained by underlying malignant disease:

- ALT or AST > 8x ULN
- ALT or AST > 5x ULN for more than 2 weeks
- ALT or AST > 3x ULN and (TBL >2x ULN or INR >1.5)
- ALT or AST > 3x ULN with the appearance of fatigue, nausea, vomiting, right upper quadrant pain or tenderness, fever, rash, and/or eosinophilia (>5%).

During the course of the study the Investigator will remain vigilant for increases in liver biochemistry. The Investigator is responsible for determining whether a patient meets potential Hy's Law (PHL) criteria at any point during the study.

The Investigator participates, together with the National Lung Matrix Trial Office, in review and assessment of cases meeting PHL criteria to agree whether Hy's Law (HL) criteria are met. HL criteria are met if there is no alternative explanation for the elevations in liver biochemistry other than Drug Induced Liver Injury (DILI) caused by the Investigational Medicinal Product (IMP).

The Investigator is responsible for recording data pertaining to PHL/HL cases and for reporting Adverse Events (AE) and Serious Adverse Events (SAE) according to the outcome of the review and assessment in line with standard safety reporting processes.

43.2 Definitions

43.2.1 Potential Hy's Law (PHL):

Aspartate Aminotransferase (AST) or Alanine Aminotransferase (ALT) ≥ 3x Upper Limit of Normal (ULN) together with Total Bilirubin (TBL) ≥ 2x ULN at any point during the study following the start of study medication irrespective of an increase in Alkaline Phosphatase (ALP).

43.2.2 Hy's Law (HL):

AST or ALT ≥ 3 x ULN together with TBL ≥ 2xULN, where no other reason, other than the IMP, can be found to explain the combination of increases, e.g., elevated ALP indicating cholestasis, viral hepatitis, another drug.

NOTE: For PHL and HL the elevation in transaminases must precede or be coincident with (i.e. on the same day as) the elevation in TBL, but there is no specified timeframe within which the elevations in transaminases and TBL must occur.

43.3 Identification of Potential Hy's Law (PHL) cases

In order to identify cases of PHL it is important to perform a comprehensive review of laboratory data for any patient who meets any of the following identification criteria in isolation or in combination:

- ALT ≥ 3x ULN

- AST ≥ 3x ULN
- TBL ≥ 2x ULN

The Investigator will also remain vigilant for any local laboratory reports where the identification criteria are met, where this is the case the Investigator will:
- Notify the National Lung Matrix Trial Office.
- Request a repeat of the test (new blood draw) by the central laboratory.
- Complete the appropriate unscheduled laboratory CRF module(s) with the original local laboratory test result.

When the identification criteria are met from central or local laboratory results the Investigator will without delay:
- Determine whether the patient meets PHL criteria (see definition) by reviewing laboratory reports from all previous visits (including both central and local laboratory results).
- The Investigator will without delay review each new laboratory report and if the identification criteria are met will:
    - Notify the National Lung Matrix Trial Office.
    - Promptly enter the laboratory data into the Laboratory Test forms on the eRDC.

43.4 Follow-up

1. Potential Hy's Law Criteria not met:
If the patient does not meet PHL criteria the Investigator will:
- Inform the National Lung Matrix Trial Office that the patient has not met PHL criteria.
- Perform follow-up on subsequent laboratory results according to the guidance provided in the Protocol.

2. Potential Hy's Law Criteria met:
If the patient does meet PHL criteria the Investigator will:
- Notify the National Lung Matrix Trial Office who will then inform AstraZeneca.

Then the National Lung Matrix Trial Office contacts the Investigator, to provide guidance, discuss and agree an approach for the study patients' follow-up and the continuous review of data. Subsequent to this contact the Investigator will:
- Monitor the patient until liver biochemistry parameters and appropriate clinical symptoms and signs return to normal or baseline levels, or as long as medically indicated.
- Investigate the aetiology of the event and perform diagnostic investigations as discussed with the Study Physician. This includes investigation of whether the PHL criteria were met at any trial visit prior to starting trial treatment in the presence of liver metastases.
- Complete the three Liver CRF Modules as information becomes available.
- If at any time (in consultation with the the National Lung Matrix Trial Office) the PHL case meets serious criteria, report it as an SAE using standard reporting procedures.

43.5 Review and assessment of Potential Hy's Law (PHL) cases

The instructions in this Section should be followed for all cases where PHL criteria are met.

No later than 3 weeks after the biochemistry abnormality was initially detected, the National Lung Matrix Trial Office contacts the Investigator in order to review available data and agree on whether there is an alternative explanation for meeting PHL criteria other than DILI caused by the IMP. The AstraZeneca Medical Science Director (MSD) and Global Safety Physician

(GSP) will also be involved in this review together with other subject matter experts as appropriate.

According to the outcome of the review and assessment, the Investigator will follow the instructions below:

If there is an agreed alternative explanation for the ALT or AST and TBL elevations, a determination of whether the alternative explanation is an AE will be made and subsequently whether the AE meets the criteria for a SAE:

- If the alternative explanation is not an AE, record the alternative explanation within a Note to File.
- If the alternative explanation is an AE/SAE, record the AE /SAE in the CRF accordingly and follow the AZ standard processes.

If it is agreed that there is no explanation that would explain the ALT or AST and TBL elevations other than the IMP:

- Report an SAE (report term 'Hy's Law') according to standard processes.
- The 'Medically Important' serious criterion should be used if no other serious criteria apply.
- As there is no alternative explanation for the HL case, a causality assessment of 'possibly, probably or definitely related' should be assigned.

If, there is an unavoidable delay, of over 3 weeks, in obtaining the information necessary to assess whether or not the case meets the criteria for HL, then it is assumed that there is no alternative explanation until such time as an informed decision can be made:

- Report an SAE (report term 'Potential Hy's Law') applying serious criteria and causality assessment as per above
- Continue follow-up and review according to agreed plan. Once the necessary supplementary information is obtained, repeat the review and assessment to determine whether HL criteria are met. Update the SAE report according to the outcome of the review

1. Actions required when potential Hy's Law Criteria are met before and after starting trial treatment

This section is applicable to patients who meet PHL criteria on trial treatment having previously met PHL criteria at a study visit prior to starting trial treatment.

At the first on trial treatment occurrence of PHL criteria being met the Investigator will:

- Determine if there has been a significant change* in the patients' condition compared with the last visit where PHL criteria were met
    - If there is no significant change no action is required
    - If there is a significant change notify the Trials Office, who will inform the Lead Investigator, Chief Investigator and AstraZeneca, then follow the subsequent process described in Section 'Potential Hy's Law Criteria not met' of this Appendix.

*A 'significant' change in the patient's condition refers to a clinically relevant change in any of the individual liver biochemistry parameters (ALT, AST or total bilirubin) in isolation or in combination, or a clinically relevant change in associated symptoms. The determination of whether there has been a significant change will be at the discretion of the Investigator, this may be in consultation with the Trials Office if there is any uncertainty.

2. Actions required for repeat episodes of potential Hy's Law

This section is applicable when a patient meets PHL criteria on study treatment and has already met PHL criteria at a previous on study treatment visit. The requirement to conduct follow-up, review and assessment of a repeat occurrence(s) of PHL is based on the nature of the alternative cause identified for the previous occurrence.

The Investigator should determine the cause for the previous occurrence of PHL criteria being met and answer the following question:

- Was the alternative cause for the previous occurrence of PHL criteria being met chronic or progressing malignant disease or did the patient meet PHL criteria prior to starting trial treatment and at their first on trial treatment visit as described in Section 'Actions required when potential Hy's law criteria are met before and after starting trial treatment' of this Appendix?
  - If No: follow the process described in Section 'Potential Hy's Law Criteria not met' of this Appendix.
  - If Yes: Determine if there has been a significant change* in the patient's condition compared with when PHL criteria were previously met
    - If there is no significant change no action is required
    - If there is a significant change follow the process described in Section 'Potential Hy's Law Criteria not met' of this Appendix 'significant' change in the patient's condition refers to a clinically relevant change in any of the individual liver biochemistry parameters (ALT, AST or total bilirubin) in isolation or in combination, or a clinically relevant change in associated symptoms. The determination of whether there has been a significant change will be at the discretion of the Investigator, this may be in consultation with the Trials Office if there is any uncertainty.

43.6 References

The above guidance is the main part of the Hy's law appendix. Further information, including additional sections that may be required for specific protocols, can be found in the FDA Guidance for Industry (issued July 2009) 'Drug-induced liver injury: Premarketing clinical evaluation':
http://www.fda.gov/downloads/Drugs/GuidanceComplianceRegulatoryInformation/Guidances/UCM174090.pdf

Aithal et al 2011, Clinical Pharmacology and Therapeutics 89(6):806-815

44 APPENDIX 13: DURVALUMAB WEIGHT-BASED DOSE CALCULATION

For patients whose weight falls below 30.1 kg during the trial, the following weight-based calculation should be utilised:

1. Dose: X mg/kg
2. Subject weight: Y kg (rounded to 1 decimal point)
3. Dose for patient: XY mg = X (mg/kg) × Y (kg)
4. Dose to be added into infusion bag:

   Dose (mL) = XY mg / 50 (mg/mL)

   where 50 mg/mL is durvalumab nominal concentration.

   The corresponding volume of durvalumab should be rounded to the nearest tenth mL (0.1 mL).
5. The number of vials required for dose preparation is the next greatest whole number of vials from the following formula:

Number of vials = Dose (mL) / 10.0 (mL/vial)

Example:

1. Dose: 20 mg/kg
2. Subject weight: 30 kg
3. Dose for patient: 600 mg = 20 (mg/kg) × 30 (kg)
4. Dose to be added into infusion bag:

Dose (mL) = 600 mg / 50 (mg/mL) = 12.0 mL

5. The number of vials required for dose preparation:

Number of vials = 12.0 (mL) / 10.0 (mL/vial) = 2 vials

Coordinating Trial Office

National Lung Matrix Trial Office
Early Drug Development Team
Cancer Research UK Clinical Trials Unit
Institute of Cancer and Genomic Sciences
University of Birmingham
Edgbaston
B15 2TT

☎ 0121 414 7611
✉ lungmatrix@trials.bham.ac.uk
www.birmingham.ac.uk/lungmatrix

Patient Registration

☎ 0121 414 7611 or 0121 414 6788
✉ lungmatrix@trials.bham.ac.uk
Monday to Friday, 9:00am to 5:00pm

Serious Adverse Event Reporting

Please email SAE Forms to:
reg@trials.bham.ac.uk

CONSORT 2010 checklist of information to include when reporting a randomised trial*

Section/Topic	Item No	Checklist item	Reported on page No
**Title and abstract**			
	1a	Identification as a randomised trial in the title	N/A (not randomised)
	1b	Structured summary of trial design, methods, results, and conclusions (for specific guidance see CONSORT for abstracts)	1
**Introduction**			
Background and objectives	2a	Scientific background and explanation of rationale	3-5
	2b	Specific objectives or hypotheses	3
**Methods**			
Trial design	3a	Description of trial design (such as parallel, factorial) including allocation ratio	5
	3b	Important changes to methods after trial commencement (such as eligibility criteria), with reasons	N/A
Participants	4a	Eligibility criteria for participants	13
	4b	Settings and locations where the data were collected	14
Interventions	5	The interventions for each group with sufficient details to allow replication, including how and when they were actually administered	14
Outcomes	6a	Completely defined pre-specified primary and secondary outcome measures, including how and when they were assessed	14
	6b	Any changes to trial outcomes after the trial commenced, with reasons	N/A
Sample size	7a	How sample size was determined	14
	7b	When applicable, explanation of any interim analyses and stopping guidelines	14
Randomisation:			
Sequence generation	8a	Method used to generate the random allocation sequence	N/A (not randomised)
	8b	Type of randomisation; details of any restriction (such as blocking and block size)	N/A (not randomised)
Allocation concealment mechanism	9	Mechanism used to implement the random allocation sequence (such as sequentially numbered containers), describing any steps taken to conceal the sequence until interventions were assigned	N/A (not randomised)

Implementation	10	Who generated the random allocation sequence, who enrolled participants, and who assigned participants to interventions	N/A (not randomised)
Blinding	11a	If done, who was blinded after assignment to interventions (for example, participants, care providers, those assessing outcomes) and how	N/A (not randomised)
	11b	If relevant, description of the similarity of interventions	N/A (not randomised)
Statistical methods	12a	Statistical methods used to compare groups for primary and secondary outcomes	14
	12b	Methods for additional analyses, such as subgroup analyses and adjusted analyses	N/A
**Results**			
Participant flow (a diagram is strongly recommended)	13a	For each group, the numbers of participants who were randomly assigned, received intended treatment, and were analysed for the primary outcome	Figure 1
	13b	For each group, losses and exclusions after randomisation, together with reasons	Figure 1
Recruitment	14a	Dates defining the periods of recruitment and follow-up	5
	14b	Why the trial ended or was stopped	5
Baseline data	15	A table showing baseline demographic and clinical characteristics for each group	Supplemental table 1
Numbers analysed	16	For each group, number of participants (denominator) included in each analysis and whether the analysis was by original assigned groups	5-7
Outcomes and estimation	17a	For each primary and secondary outcome, results for each group, and the estimated effect size and its precision (such as 95% confidence interval)	5-7
	17b	For binary outcomes, presentation of both absolute and relative effect sizes is recommended	5-7
Ancillary analyses	18	Results of any other analyses performed, including subgroup analyses and adjusted analyses, distinguishing pre-specified from exploratory	N/A
Harms	19	All important harms or unintended effects in each group (for specific guidance see CONSORT for harms)	Supplemental table 2
**Discussion**			
Limitations	20	Trial limitations, addressing sources of potential bias, imprecision, and, if relevant, multiplicity of analyses	12
Generalisability	21	Generalisability (external validity, applicability) of the trial findings	12
Interpretation	22	Interpretation consistent with results, balancing benefits and harms, and considering other relevant evidence	8-12
**Other information**			
Registration	23	Registration number and name of trial registry	12
Protocol	24	Where the full trial protocol can be accessed, if available	Supplemental

Funding	25	Sources of funding and other support (such as supply of drugs), role of funders	18

Citation: Schulz KF, Altman DG, Moher D, for the CONSORT Group. CONSORT 2010 Statement: updated guidelines for reporting parallel group randomised trials. BMC Medicine. 2010;8:18.
© 2010 Schulz et al. This is an Open Access article distributed under the terms of the Creative Commons Attribution License (http://creativecommons.org/licenses/by/2.0), which permits unrestricted use, distribution, and reproduction in any medium, provided the original work is properly cited.

*We strongly recommend reading this statement in conjunction with the CONSORT 2010 Explanation and Elaboration for important clarifications on all the items. If relevant, we also recommend reading CONSORT extensions for cluster randomised trials, non-inferiority and equivalence trials, non-pharmacological treatments, herbal interventions, and pragmatic trials. Additional extensions are forthcoming: for those and for up-to-date references relevant to this checklist, see www.consort-statement.org.
